# Reactivities of tertiary phosphines towards allenic, acetylenic, and vinylic Michael acceptors†

**DOI:** 10.1039/d4sc04852k

**Published:** 2024-10-14

**Authors:** Feng An, Jan Brossette, Harish Jangra, Yin Wei, Min Shi, Hendrik Zipse, Armin R. Ofial

**Affiliations:** a Department Chemie, Ludwig-Maximilians-Universität München Butenandtstr. 5-13 81377 München Germany zipse@cup.uni-muenchen.de ofial@lmu.de; b State Key Laboratory of Organometallic Chemistry, Center for Excellence in Molecular Synthesis, University of Chinese Academy of Sciences, Shanghai Institute of Organic Chemistry, Chinese Academy of Sciences 345 Lingling Road Shanghai P. R. China; c Key Laboratory for Advanced Materials and Institute of Fine Chemicals, School of Chemistry & Molecular Engineering, East China University of Science and Technology Meilong Road No. 130 200237 Shanghai P. R. China

## Abstract

The addition of phosphines (PR_3_) to Michael acceptors is a key step in many Lewis-base catalysed reactions. The kinetics of the reactions of ten phosphines with ethyl acrylate, ethyl allenoate, ethyl propiolate, ethenesulfonyl fluoride, and ethyl 2-butynoate in dichloromethane at 20 °C was followed by photometric and NMR spectroscopic methods. The experimentally determined second-order rate constants *k*_2_ show that electronic effects in sterically unencumbered phosphines affect their nucleophilicity towards different classes of Michael acceptors in the same ordering. Michael acceptors with sp-hybridised electrophilic centres, however, are less susceptible to changes in the PR_3_ nucleophilicity than those with sp^2^-hybridised reactive sites. Linear correlations of lg *k*_2_ from this work with published rate constants for S_N_2 and S_N_1 reactions as well as with Brønsted basicities and fugalities for PR_3_ demonstrate the generality of the detected reactivity trends. Computed reaction barriers (Δ*G*^‡^_calc_) as well as reaction energies (Δ*G*_add_) for Michael adduct formations show excellent correlations with experimentally obtained reaction barriers (Δ*G*^‡^_exp_) corroborating the interpretation of the kinetic data and revealing the philicity/fugality features of the reactants in phospha-Michael additions.

## Abstract 2

Combined experimental and quantum-chemical studies were used to characterise the philicity/fugality features of tertiary phosphines and Michael acceptors in phospha-Michael reactions, which are key steps in phosphine-catalysed organic reactions.

## Graphical abstract

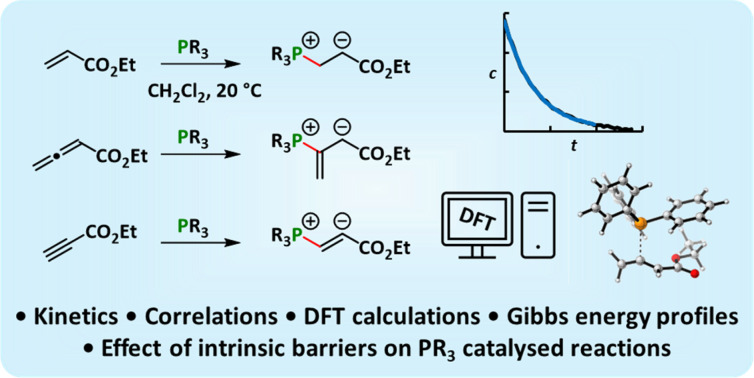

## Introduction

Addition of tertiary phosphines to electron-deficient π-systems generates zwitterionic intermediates which can be trapped directly or after isomerisation with various types of electrophilic reagents for carbon–carbon bond-formation. Thus, Horner's anionic acrylonitrile polymerisation,^1^ Rauhut–Currier reactions, Morita–Baylis–Hillman reactions,^2–4^ Lu's (3 + 2) cycloadditions, Kwon's [4 + 2] annulations^5^ and many other useful Lewis-base catalysed reactions^6^ share phospha-Michael additions^7^ as initiating steps in their catalytic cycles toward complex, functionalised products.^8^ Chiral phosphine catalysts have enabled enantioselective versions of these transformations.^9^ Though some phospha-Michael additions have recently been exploited for bioorthogonal reactions to detect α,β-unsaturated carbonyl groups in biomolecular targets,^10^ the reversibility of the endergonic phosphine additions to Michael acceptors has remained a challenge for kinetic studies.

**Scheme 1:**
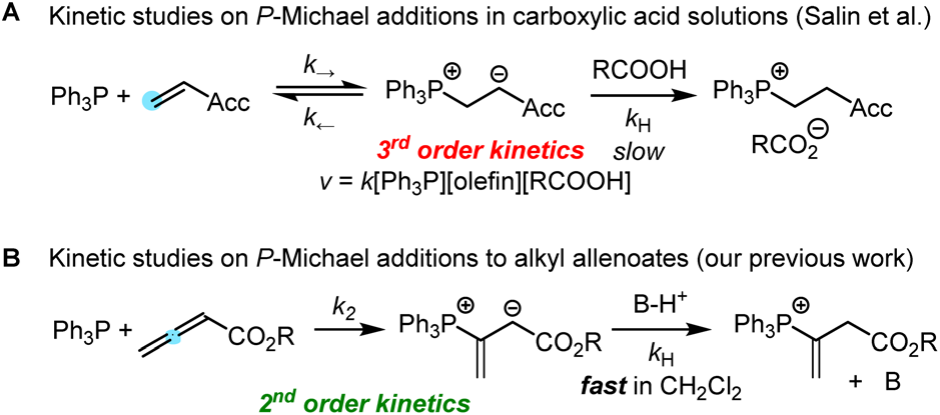
Kinetics of P-Michael additions.

Protonation of the zwitterionic intermediate is a straightforward approach to render the phospha-Michael addition irreversible. The kinetics of phospha-Michael additions in protic and aprotic solvents with carboxylic acids as the proton sources were carefully investigated by Salin and co-workers (Scheme 1A).^11–14^ Generally, rate-determining proton transfer from carboxylic acids to the intermediate zwitterions gave rise to third-order kinetics (Scheme 1A).

Recently, we studied the kinetics of the adduct formation of PBu_3_ and PPh_3_ with alkyl and phenyl allenoates in dichloromethane solution.^15^ By utilising collidinium triflate and trialkylphosphonium triflates as the proton sources (BH^+^), the intermediate zwitterions were efficiently trapped in fast reactions. It, thus, became possible to determine second-order rate constants *k*_2_ for phosphine additions to allenoates, which allowed us to identify the impact of structural variation on the reactivity of these electrophiles (Scheme 1B).

**Scheme 2:**
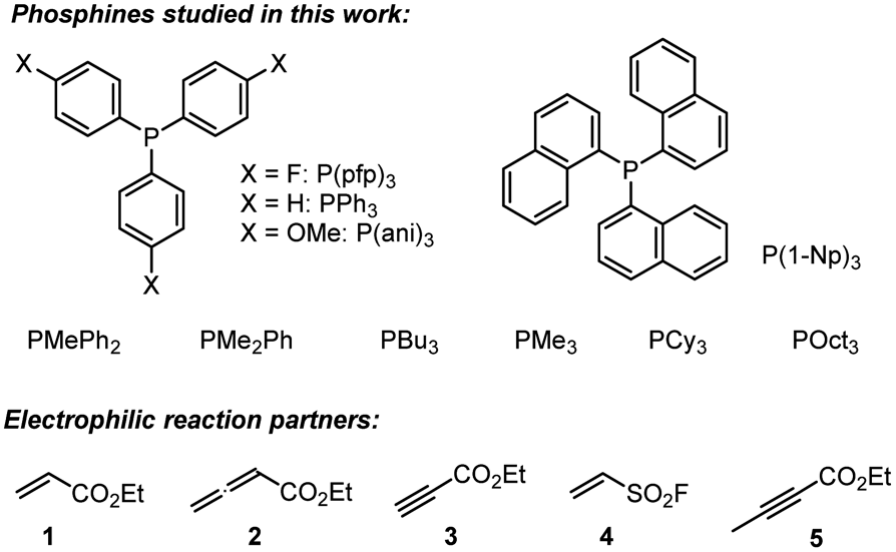
Phosphines PR_3_ and electrophiles used in this work (Cy = cyclohexyl).

A complementary, systematic comparison of PR_3_ reactivities in phospha-Michael additions across different types of Michael acceptors is not available to date.^16^ We, therefore, set out to investigate the kinetics of PR_3_ additions to allenic, acetylenic and vinylic Michael acceptors. Herein, we present the analysis of the addition kinetics of ten phosphines to ethyl acrylate (1), ethyl allenoate (2), ethyl propiolate (3), ethenesulfonyl fluoride (4), and ethyl 2-butynoate (5) in dichloromethane at 20 °C (Scheme 2), which were followed by photometric and NMR spectroscopic methods. Given that these phosphine additions are generally considered to be the first step in PR_3_-catalysed reactions, kinetic investigations will also help to gain further insight into the key factors that control a manifold of organocatalytic reactions. Furthermore, quantum-chemical methods were employed to rationalise the reactivity ordering observed in the kinetic experiments.

## Results and discussion

### Products of the reactions of phosphines with the electrophiles 1–3

Horner and co-workers showed that stable, zwitterionic phospha-Michael adducts are obtained when PEt_3_ or PPh_3_ are combined with highly reactive Michael acceptors, such as 1,1-dicyanoethene.^1^ They noted, however, that the less Lewis acidic benzylidenemalononitrile (BMN) only forms Lewis adducts with trialkylphosphines (PMe_3_, PEt_3_, and PBu_3_) but not with PPh_3_. Photometric studies of the isolated adduct of benzylidenemalononitrile and PEt_3_ surprisingly showed that the UV-vis spectrum was identical to benzylidenemalononitrile alone. Horner explained this experimental observation by the dissociation of the Michael adduct in solution.^1^

Efficient trapping of the initially formed zwitterionic phospha-Michael adducts is, therefore, a prerequisite to get to observable reaction products in solution and to generate conditions for reproducible kinetic measurements. Hence, we started our preparative investigations by NMR characterisations of relevant protonated Michael adducts.

**Scheme 3:**
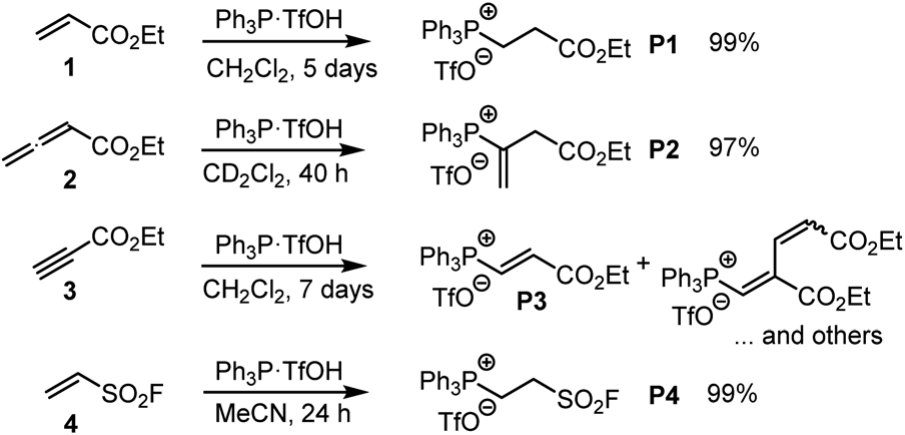
Generation of phosphonium triflates P1–P4 by reaction of PPh_3_·TfOH (TPPT) with the Michael acceptors 1–4 (at ambient temperature).

The Michael addition of PPh_3_ to acrylate 1 was first reported by Hoffmann, who used triphenylphosphine hydrobromide that reacted with ethyl acrylate (1) within 15 min in acetonitrile.^17^ We used pre-formed triphenylphosphonium triflate (TPPT) to characterise the products of its reactions with the electrophiles 1–4 by NMR spectroscopic and HRMS methods (Scheme 3).

TPPT reacted slowly but selectively with ethyl acrylate (1) to furnish within five days almost quantitatively the phosphonium triflate P1, which was isolated in a yield of 99%.^18^ The analogous reaction of TPPT with ethyl allenoate (2) in CD_2_Cl_2_ generated the vinylphosphonium triflate (P2) in a yield of 97%.^15^ The reaction of TPPT with Michael acceptor 3 mainly produced the acceptor-substituted vinyl phosphonium salts P3. The NMR spectra and in particular the HRMS analytical data showed that also 2 : 1 products (as a mixture of *E*- and *Z*-isomers) were formed in significant amounts, which could not be separated from the 1 : 1 adduct P3. Ethenesulfonyl fluoride (ESF, 4) is a considerably stronger electrophile than 1–3. Accordingly, 4 reacted already within 24 h quantitatively with TPPT to yield the phosphonium triflate P4 (99% yield of isolated product).

**Scheme 4:**
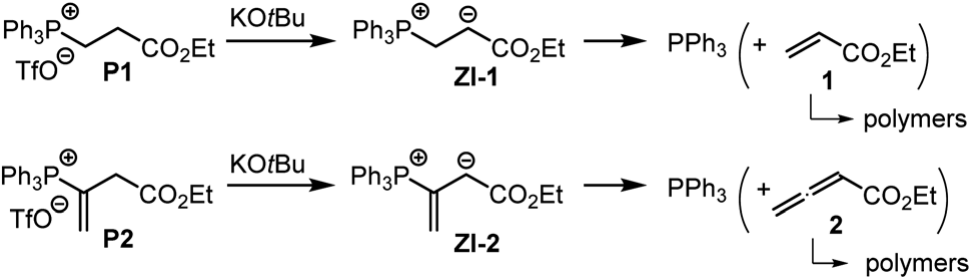
Retro-Michael additions of P1 and P2 under basic reaction conditions (in *d*_6_-DMSO).

The associations of triphenylphosphine with Michael acceptors 1–4 are highly reversible if the reactions are performed without an appropriate proton source that efficiently traps the incipient, zwitterionic Lewis adducts. Attempts to generate the zwitterions ZI-1 and ZI-2 by deprotonation of the phosphonium salts P1 and P2, respectively, with potassium *t*-butoxide in *d*_6_-DMSO gave rise to rapid retro-Michael additions (Scheme 4). The release of free PPh_3_ from P1 and P2 was unequivocally detected by ^1^H, ^13^C, and ^31^P NMR spectroscopic analysis of the reaction mixtures (ESI, Fig. S19–S24†). The electrophiles 1 and 2 cannot be recovered under these reaction conditions. However, owing to a lack of NMR signals in the olefinic region and the occurrence of various new resonances in the aliphatic region, we assume that 1 and 2 rather undergo anionic polymerisations. These observations indicate that Lewis acid/base adduct formation between triphenylphosphine and the Michael acceptors 1 or 2 are endergonic.

To investigate the kinetics of the first step of the phosphine-catalyzed reactions with Michael acceptors, we have, therefore, decided to combine phosphines PR_3_ with the relevant electrophiles in the presence of proton sources that are able to intercept the initially formed zwitterions by fast protonation. The next section, therefore, shows how we identified Brønsted acids that reliably trapped the zwitterionic adducts but did not influence the reactivities of two reaction partners in the phospha-Michael addition.

### Choice of proton sources as trapping reagents for the intermediate zwitterions

Ohmori and colleagues showed that the reaction of PPh_3_ with 1 (in CH_2_Cl_2_) can be performed under neutral conditions when 2,6-lutidinium perchlorates or tetrafluoroborates are used as proton sources.^19^ Lutidinium ions are only weak acids (p*K*_a_ = 14.16 in MeCN)^20^ and it can be expected that they are neither able to protonate PPh_3_ (p*K*_aH_ = 7.62 in MeCN)^20^ nor the Michael acceptor 1.

**Fig. 1:**
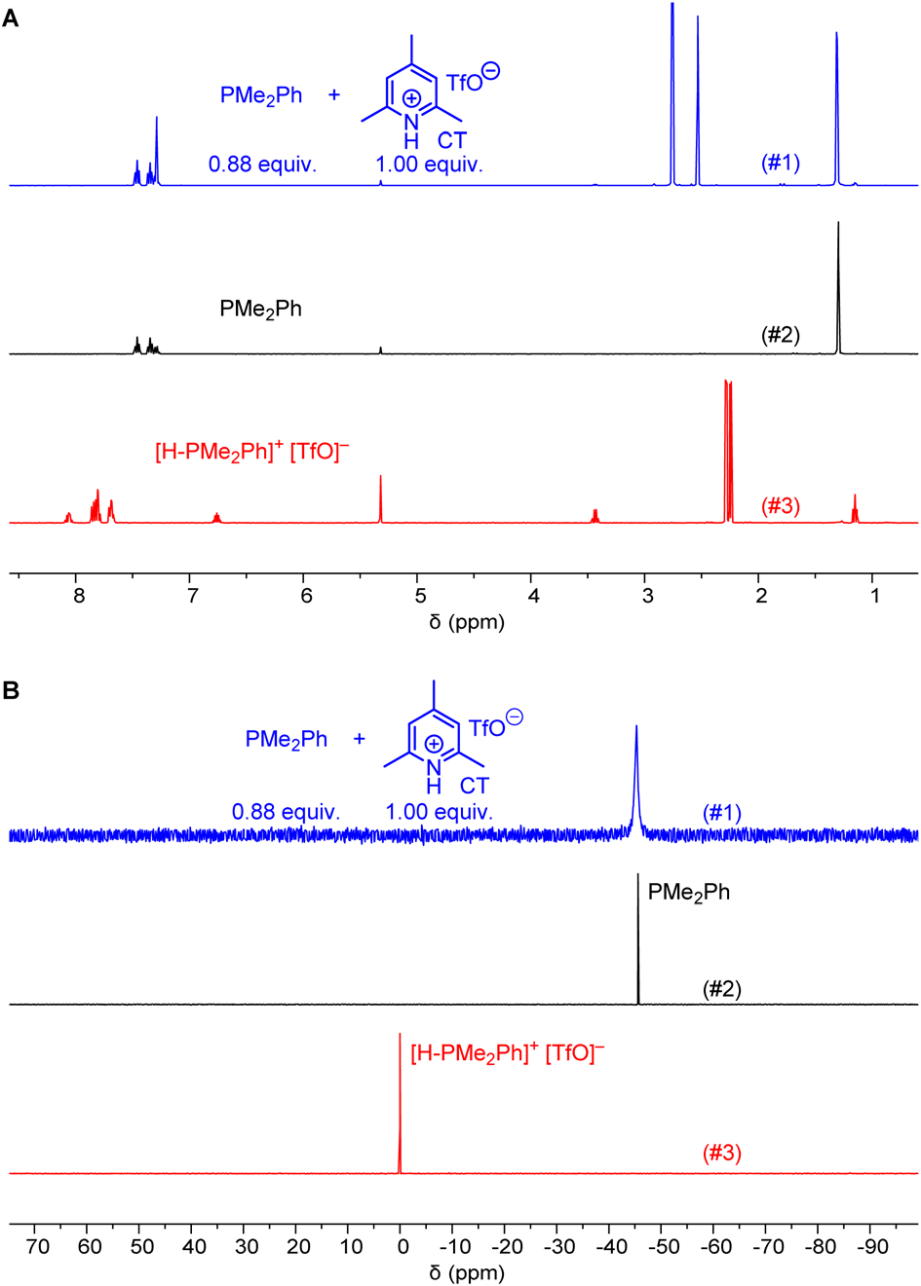
(A) 400 MHz ^1^H and (B) 162 MHz ^31^P{^1^H} NMR spectra of a mixture of collidinium triflate (CT) and PMe_2_Ph in CD_2_Cl_2_ (#1 in blue) compared to the ^1^H and ^31^P{^1^H} NMR spectra of PMe_2_Ph (#2 in black) and dimethylphenylphosphonium triflate (#3 in red).

Given that we needed a proton source to cover the Brønsted basicity range from P(pfp)_3_ to PMe_2_Ph (p*K*_aH_ = 12.64 in MeCN) without affecting the reactivity of the phosphines, we expected that the even less acidic 2,4,6-collidinium triflate (p*K*_a_ = 15.00 in MeCN)^20^ would be a practical trapping reagent for kinetic measurements. NMR spectroscopic studies in CD_2_Cl_2_ were carried out to assess whether the known relative acidities in acetonitrile are transferable to those in dichloromethane solution. The ^1^H NMR spectrum of a mixture of PMe_2_Ph with a slight excess of 2,4,6-collidinium triflate (CT) showed only resonances that could be assigned to both individual components in the mixture. Resonances for the phenyl group in [H-PMe_2_Ph]^+^ at *δ* > 7.5 ppm were not detected, and also the CH_3_ resonance of PMe_2_Ph at *δ* = 1.31 ppm did not shift when mixed with CT (Fig. 1A). Accordingly, the ^31^P NMR spectrum of a mixture of CT with PMe_2_Ph (0.9 equiv.) in CD_2_Cl_2_ showed that the detected chemical shift (*δ*_P_ = −45.6 ppm) corresponds to free PhMe_2_P and is not shifted towards the resonance for the protonated form (*δ*_P_ = 0.0 ppm, Fig. 1B). An analogous ^1^H and ^31^P NMR study for a mixture of the less basic PMePh_2_ (p*K*_aH_ = 9.97 in MeCN)^20^ and CT is presented in the ESI (Fig. S3 and S4†) and shows, accordingly, that CT does not protonate PMePh_2_ in dichloromethane.

Further NMR studies were carried out to elucidate possible interactions of CT additives with the Michael acceptors 1–3. Fig. S7–S9† (ESI) illustrate that the ^1^H NMR chemical shifts of the acrylate 1, the allenoate 2, and the propiolate 3, respectively, did not undergo changes when mixed with CT in CD_2_Cl_2_. The resonances assigned to CT remained unchanged, which indicates that this proton source does not interact with the electrophiles. Given that interactions of CT were neither observable with the electrophilic Michael acceptors 1–3 nor with those phosphines PR_3_ with basicities lower than that of PMe_2_Ph (p*K*_aH_ < 12.64 in MeCN), it can be expected that CT will be a suitable zwitterion intercepting reagent for kinetic experiments in dichloromethane solution.

Kinetic measurements for reactions of electrophiles with the more basic trialkylphosphines PMe_3_, PCy_3_ and POct_3_ were carried out by *in situ* liberation of a certain amount of free PR_3_ from trialkylphosphonium triflates by adding known amounts of the strong Brønsted base triethylamine (TEA) to the solutions in dichloromethane. The detection of only a single ^31^P NMR signal (in CD_2_Cl_2_) indicates quantitative deprotonation of [H-PR_3_]^+^ by TEA (ESI, Fig. S16–S18†). By using the thus generated solutions, the reversibly formed adducts of the reactions of the electrophiles 1–3 with PMe_3_, PCy_3_ and POct_3_, respectively, were efficiently trapped by the conjugate Brønsted acids of the studied PR_3_ nucleophiles. Because handling and further dilution steps of the trialkylphosphine stock solutions were avoided by this procedure, also oxidation prone PR_3_ could be studied under reliable conditions and delivered reproducible kinetic data.

Comparing the heats of formation for the parent allene (Δ_f_*H* = +192.1 kJ mol^−1^) with that for propyne (Δ_f_*H* = +185.4 kJ mol^−1^) shows that the alkyne is the thermodynamically favored isomer.^21^ We, therefore, tested whether the allene derivative 2 can isomerise to the acetylene derivative 5 under the conditions of the kinetic experiments. The ^1^H NMR spectra of TEA + 2 and TEA + 5 mixtures in CD_2_Cl_2_, which were stored at ambient temperature overnight (ESI, Fig. S11 and S12†), remained unchanged, however, indicating that free TEA is not able to equilibrate the allenoate 2 with the corresponding alkynoate tautomer 5, or *vice versa*.

Furthermore, comparing ^1^H NMR spectra of the mixtures of triethylammonium triflate (TEAT), which is generated during the *in situ* liberation of PR_3_, and Michael acceptors 1, 2, and 5 (in CD_2_Cl_2_) with those of the individual compounds in the same solvent showed that TEAT (possible proton source) does not interact with the electrophiles 1, 2, and 5 (ESI, Fig. S13–S15†). Interestingly, also mixtures of tributylphosphonium triflate (TBPT) with ethyl acrylate (1) show ^1^H NMR spectra, which reflect the resonances of the individual compounds (ESI, Fig. S10†), thus excluding significant electrophile activation by the presence of the TBPT proton source.

### Kinetics

Depending on the spectroscopic properties of the reagents, the kinetics of PR_3_ additions to the electrophiles 1–3 were monitored by using either photometry or NMR spectroscopy.

The majority of the kinetics of reactions of 2 and 3 with phosphines in dichloromethane at 20 °C were determined by following absorption changes in the UV-range. For a straightforward evaluation of the absorption decay curves, we used one of the reaction partners in at least 10-fold excess relative to the initial concentration of the minor compound. This made it possible that first-order rate constants *k*_obs_ (s^−1^) could be derived from fitting the mono-exponential function *A* = *A*_0_ exp(−*k*_obs_*t*) + *C* to the experimentally observed decrease of the absorption of the minor compound. Determination of *k*_obs_ at four different concentrations of the excess reaction partner enabled us to calculate the second-order rate constants *k*_2_ (M^−1^ s^−1^) from the slope of the linear regression line of *k*_obs_*vs.* [PR_3_]_0_ or [electrophile]_0_. Furthermore, the linearity of both types of plots, that is, *k*_obs_*vs.* [PR_3_]_0_ and *k*_obs_*vs.* [electrophile]_0_, indicates the operation of a rate law for the overall reaction, which is first order in [PR_3_] and first order in [electrophile].

**Fig. 2:**
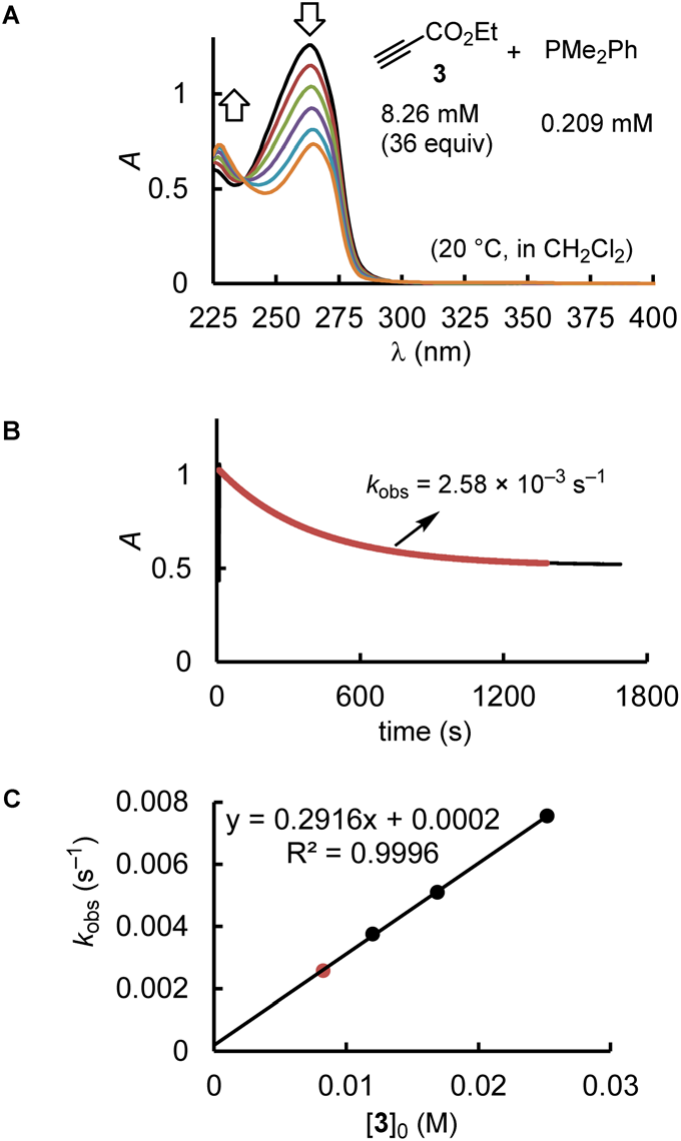
(A) The decay of the UV absorption of PMe_2_Ph was used to monitor the kinetics of the reaction of PMe_2_Ph with ethyl propiolate (3) in CH_2_Cl_2_ under pseudo first-order conditions (proton source: CT, [CT]_0_ = 0.232 mM). (B) Exponential decay of the absorption *A* at 252 nm during the reaction. (C) Determination of the second-order rate constant *k*_2_ (M^−1^ s^−1^) from the slope of a linear correlation of *k*_obs_ (s^−1^) *vs.* [3]_0_.

Fig. 2 uses the relatively slow reaction of 3 with PMe_2_Ph to illustrate the workflow for kinetic measurements by conventional photometric equipment and their subsequent evaluation. The kinetics of faster reactions (*t*_1/2_ < 40 s) were followed by using stopped-flow spectrophotometer systems and analysed analogously. The sequential mixing option of the stopped-flow instrument was used to study the kinetics of the fast reactions of PMe_3_ with the electrophiles 1, 2, and 4. At mixer 1, PMe_3_ was liberated by deprotonation of trimethylphosphonium tetrafluoroborate with a substoichiometric amount of TEA. The thus prepared nucleophile solution was then mixed at mixer 2 with the solution of 1, 2, or 4. Details for the individual kinetics are given in the ESI.†

**Fig. 3:**
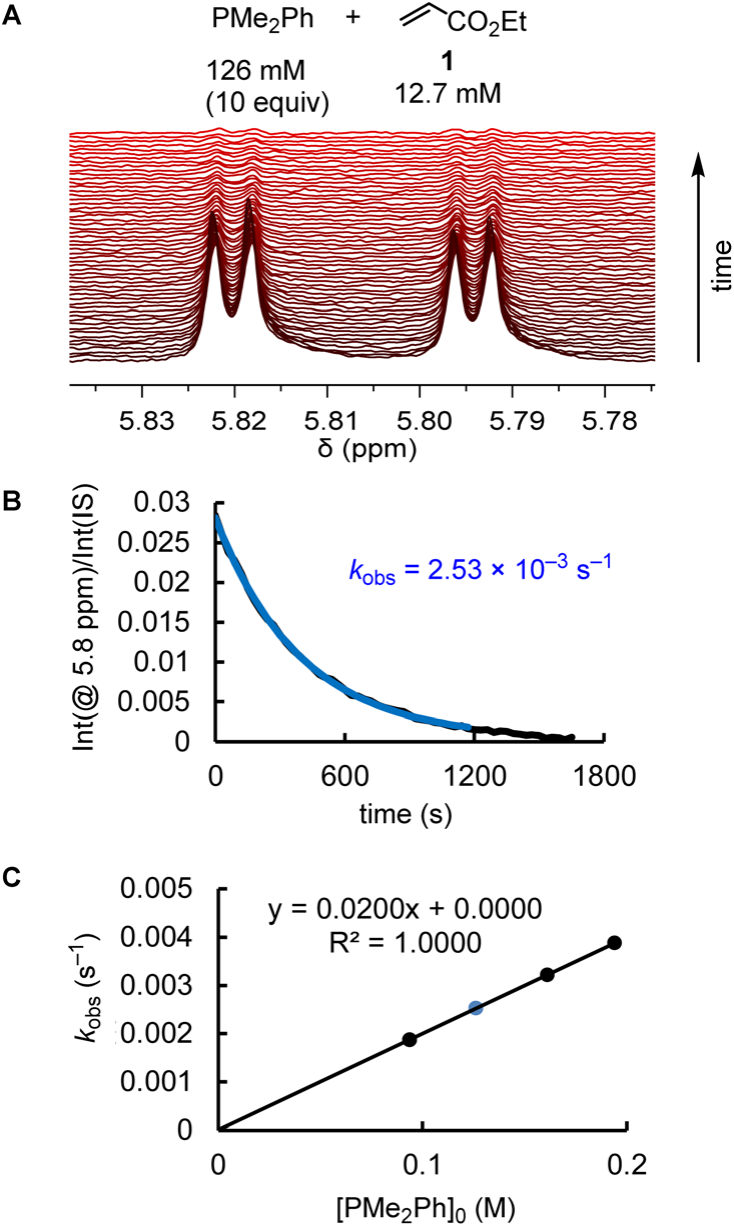
(A) Monitoring the kinetics of the PMe_2_Ph addition to ethyl acrylate (1) in CD_2_Cl_2_ (20 °C) by ^1^H NMR spectroscopy (proton source: CT, [CT]_0_ = 21.4 mM). (B) Exponential decay of the integrals for the olefinic protons at 5.8 ppm during the progress of the reaction (mesitylene was used as an internal integration standard, IS). (C) Determination of the second-order rate constant *k*_2_ (M^−1^ s^−1^) from the slope of a linear correlation of *k*_obs_ (s^−1^) *vs.* [PMe_2_Ph]_0_.

The kinetics of further phosphine–electrophile reactions, in particular those which involved phosphines with aryl groups, were more accessible through the use of NMR techniques.^22^ Tracing the time-dependent changes in the ^1^H NMR spectra was used, for example, to follow the kinetics of the PMe_2_Ph addition to ethyl acrylate (1) (Fig. 3). CT trapped the intermediate zwitterions. Added mesitylene served as the internal integration standard. The experiment shown in Fig. 3A was repeated at different CT concentrations at otherwise identical conditions. For [CT] = 21.4, 37.1, and 73.3 mM, the observed first-order rate constants *k*_obs_ were 2.53 × 10^−3^, 2.47 × 10^−3^, and 2.55 × 10^−3^ s^−1^, respectively (ESI, Table S10†). An analogous independency of *k*_obs_ in the reaction of PBu_3_ + 1 was observed when enhancing the TBPT concentration (ESI, Table S11†). Thus, in accord with the previous NMR investigations on binary CT (or TBPT) mixtures with either phosphines or electrophiles, the observed rate constant *k*_obs_ remained unchanged within the experimental error limit, which corresponds to a zeroth order kinetics with regard to the concentration of the proton source. This observation underpins again that the nature of the proton sources selected in this work did not affect the kinetics of the phospha-Michael additions we aimed to investigate.

Phosphines PR_3_ were used as the excess compounds when the decay of the electrophile was followed by ^1^H NMR spectroscopy (Fig. 3). An inverse concentration ratio, that is, with the electrophiles as the excess compounds, was employed for ^31^P NMR kinetic measurements. The kinetics for the combinations of PMePh_2_ with 1 gave *k*_2_ = 1.66 × 10^−3^ M^−1^ s^−1^ by ^1^H NMR (ESI, Table S9†) and *k*_2_ = 2.36 × 10^−3^ M^−1^ s^−1^ by ^31^P NMR spectroscopy (ESI, Table S8†), which agree within a factor of 1.4. Reaction monitoring of the kinetics of P(pfp)_3_ with ethyl allenoate (2) gave a *k*_2_(^31^P)/*k*_2_(^1^H) ratio of 1.1 (ESI, Tables S16 and S17†). For the reactions of PPh_3_ with ethyl propiolate 3 (ESI, Tables S28 and S29†), ^1^H NMR spectroscopy delivered a slightly higher *k*_2_ value than the ^31^P NMR spectroscopic reaction tracing [*k*_2_ = 1.04 × 10^−2^ M^−1^ s^−1^ (^1^H) *vs.* 7.05 × 10^−3^ M^−1^ s^−1^ (^31^P)]. In general, we considered rate constants determined by time-resolved ^1^H NMR spectroscopy to be more reliable than results from ^31^P NMR spectroscopic reaction monitoring because ^1^H NMR spectra were recorded with an internal integration reference. In subsequent correlations we, therefore, preferred to use *k*_2_ from ^1^H NMR kinetics if *k*_2_ for a given PR_3_ + electrophile pair was determined by both ^1^H and ^31^P NMR kinetics.

To further test the influence of the zwitterion trapping on the kinetics, we used two different proton sources (CT and TEAT) when following the kinetics of the reaction of PPh_3_ with ethyl acrylate (1) by ^31^P NMR spectroscopy (Table 1, entries 1 & 2). The individual linear correlations of *k*_obs_ with [1]_0_ agreed so surprisingly well that we used the first-order rate constants from both series of kinetic measurements jointly to determine the second-order rate constant *k*_2_ for the PPh_3_ + 1 reaction (ESI, Table S6†).

**Table 1:** Comparison of the kinetics of phospha-Michael additions (dichloromethane, 20 °C) with variable zwitterion trapping reagents

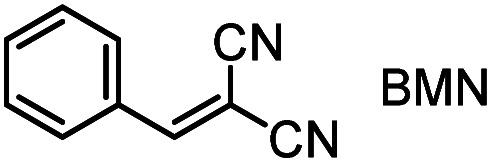			
Entry	Reactions	Trapping reagents	*k*_2_ a (M^−1^ s^−1^)
1	PPh_3_ + 1	CT	1.55 × 10^−4^^a^
2	PPh_3_ + 1	TEAT	1.56 × 10^−4^^a^
3	PBu_3_ + 1	TBPT	5.83 × 10^−2^^b^
4	PBu_3_ + 1	BMN	5.47 × 10^−2^^b^
5	PMe_2_Ph + 3	CT	0.292^b^
6	PMe_2_Ph + 3	BMN	0.257^b^

a Determined by time-resolved ^31^P NMR spectroscopy.

b Determined by photometric methods.

In a next step, we sought to replace the proton source by an olefinic Michael acceptor, that is, a neutral carbon-centred electrophile. The highly electrophilic BMN (Mayr *E* = −9.42)^23^ was used by Lu and coworkers as a reaction partner for alkyl allenoates 2 in PPh_3_-catalysed cyclopentene syntheses.^24^ Recently, we could demonstrate that BMN reacts fast yet reversibly with PBu_3_.^15^ Generation of cycloadducts, therefore, requires initial PBu_3_ attack at the allenoate electrophile to be productive. Entries 3 and 4 in Table 1 show that the second-order rate constants *k*_2_ for reactions of ethyl acrylate (1) with PBu_3_ are identical (within an error margin of ±10%) and independent of whether proton (TBPT) or BMN trapping was used. Similar rate constants *k*_2_ (within ±10%) were also derived for the reaction of ethyl propiolate (3) with PMe_2_Ph when CT and BMN were compared as trapping reagents (entries 5 & 6). The results in Table 1, thus, corroborate that the proton sources used in the kinetic standard procedure to generate the data for Table 2 neither attenuated the reactivity of the PR_3_ nucleophiles nor enhanced the electrophilicity of the esters 1 and 3 by protonation.

**Table 2:** Second-order rate constants *k*_2_ for the reactions of phosphines PR_3_ with the Michael acceptors 1–5 (in dichloromethane, at 20 °C)

Phosphines PR_3_	*k*_2_ a (M^−1^ s^−1^)				
	Ethyl acrylate (1)	Ethyl allenoate (2)	Ethyl propiolate (3)	ESF (4)	Ethyl 2-butynoate (5)
P(pfp)_3_	2.22 × 10^−5^^b^	3.43 × 10^−3^^c^	2.86 × 10^−3^^c^	4.23 × 10^−1^	n.d.
PPh_3_	1.55 × 10^−4^^b^	7.67 × 10^−3^^c^^,^^d^	1.04 × 10^−2^^c^	3.38	2.05 × 10^−5^^b^
P(ani)_3_	9.32 × 10^−4^^b^	3.76 × 10^−2^	5.45 × 10^−2^	n.d.	n.d.
PMePh_2_	1.66 × 10^−3^^c^	6.12 × 10^−2^	6.28 × 10^−2^	8.76	n.d.
PMe_2_Ph	2.00 × 10^−2^^c^	2.68 × 10^−1^	2.92 × 10^−1^	4.26 × 10^2^	n.d.
PBu_3_	5.83 × 10^−2^	6.35 × 10^−1^	9.61 × 10^−1^	7.99 × 10^2^	5.71 × 10^−3^^c^
PMe_3_	1.24 × 10^−1^	9.69 × 10^−1^	9.56 × 10^−1^	2.07 × 10^3^	n.d.
PCy_3_	3.82 × 10^−2^^c^	1.20 × 10^−1^	1.43	n.d.	n.d.
POct_3_	5.45 × 10^−2^	7.24 × 10^−1^	2.69	n.d.	n.d.
P(1-Np)_3_	Too slow^b^	8.00 × 10^−6^^b^	8.46 × 10^−5^^b^	n.d.	n.d.

a In CH_2_Cl_2_, kinetics followed by photometric methods if not mentioned otherwise.

b In CD_2_Cl_2_, kinetics followed by online ^31^P NMR spectroscopy.

c In CD_2_Cl_2_, kinetics followed by online ^1^H NMR spectroscopy.

d For the reaction of 2 with PPh_3_ in benzene activation parameters Δ*H*^‡^ = 14.8 kcal mol^−1^ and Δ*S*^‡^ = −19.6 cal mol^−1^ K^−1^ were reported in ref. 25, which correspond to a second-order rate constant of *k*_2_ = 2.9 × 10^−3^ M^−1^ s^−1^ (20 °C).

Table 2 gathers the second-order rate constants *k*_2_ for the reactions of the phosphines PR_3_ with the electron-deficient π-systems in 1, 2, and 3.^25^ In addition, the kinetics of PR_3_ reactions with ESF (4) were included in the study. ESF (4) is a Michael acceptor that is known to be considerably more electrophilic than ethyl acrylate 1.^26^ Furthermore, we investigated the reactivity of ethyl 2-butynoate (5), an isomer of 2, towards PBu_3_ and PPh_3_.

**Fig. 4:**
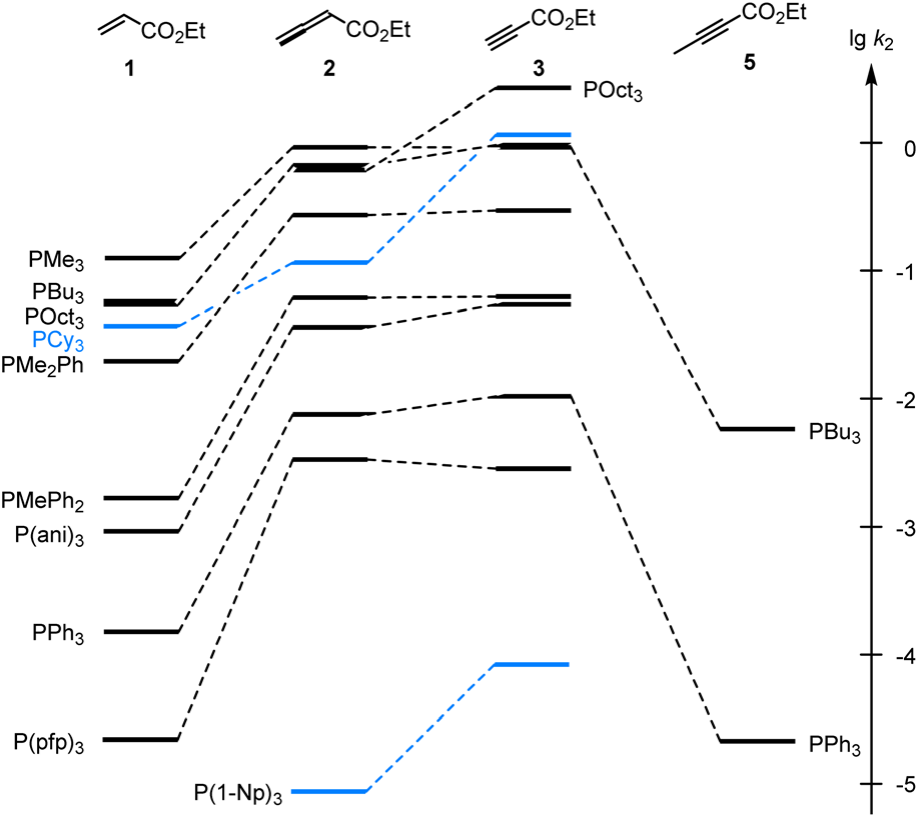
Reactivities of PR_3_ towards the Michael acceptors 1, 2, 3, and 5 compared by the second-order rate constants (lg *k*_2_) for the formation of Michael adducts in dichloromethane at 20 °C (data from Table 2).

As illustrated in Fig. 4, the second-order rate constants *k*_2_ in Table 2 show that ethyl acrylate (1) is a relatively weak electrophile towards phosphines PR_3_. The reactivities of PR_3_ towards ethyl allenoate (2) and ethyl propiolate (3) are at almost the same levels and generally exceed those towards ethyl acrylate (1) by one to two orders of magnitude. The reactivity of the terminal alkynyl π-system in the ethyl ester 3 is reduced by 2.5 to 3 orders of magnitude if a methyl substituent is added, as shown by the entries for ethyl 2-butynoate (5), which is even by a factor of 10 less reactive towards phosphines PR_3_ than ethyl acrylate (1).

Significant changes in the relative order of PR_3_ reactivities are only observed for the sterically demanding phosphine PCy_3_,^27^ which catches up in reactivity with other trialkylphosphines when it adds to the terminal electrophilic carbons in 1 or 3. However, PCy_3_ reacts considerably slower than other trialkylphosphines with 2, in which the electrophilic reaction centre is the central carbon in the allene π-system and thus more difficult to access for the bulky PCy_3_ than for the sterically less demanding phosphines PMe_3_, PBu_3_ or POct_3_. As a consequence, rate constants for reactions of PCy_3_ were generally excluded in the subsequent correlation analyses, which were performed to gain further quantitative insight in structure–reactivity relationships for Michael additions of phosphines PR_3_. The same reasons that explain the *k*_2_(3)/*k*_2_(2) = 12 for PCy_3_ can be applied to rationalise the by one order of magnitude higher reactivity of P(1-Np)_3_ towards 3 (terminal electrophilic centre) than towards 2.

### Correlation analysis

#### Reactivities of Michael acceptors

**Fig. 5:**
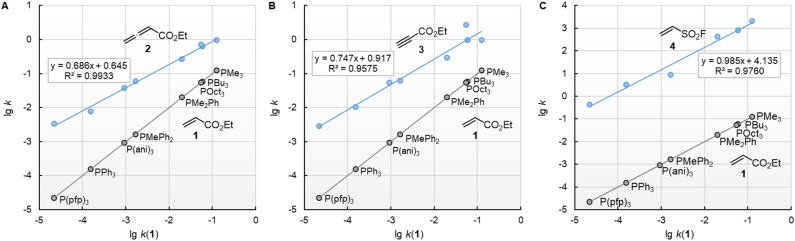
Relative reactivities of PR_3_ towards (A) ethyl allenoate (2), (B) ethyl propiolate (3), and (C) ESF (4) referenced towards lg *k*(PR_3_ + 1). With rate constants *k*_2_ from Table 2, data for the sterically encumbered PCy_3_ was excluded when constructing the correlation lines.

The decadic logarithm of the second-order rate constants (lg *k*_2_) of the reactions with ethyl acrylate (1) can be used as a reference to compare the susceptibilities of the different types of electrophiles for variation of the phosphine reactivities. Fig. 5 shows that the relative trends are identical when changing from the sp^2^-hybridised electrophilic centre in 1 to the sp-hybridised reactive positions in 2 or 3. The slopes of 0.686 and 0.747 for the correlations with 2 and 3, respectively, illustrate however, that the increase in phosphine reactivity towards Michael acceptor 1 is only partially found in the faster reactions with the electron-deficient π-systems in 2 and 3. The lower susceptibility is not a consequence of the Reactivity-Selectivity-Principle, a concept that has been criticised several times before.^28^ This can be demonstrated through reactions of the phosphines PR_3_ with ESF (4), which are much faster than those with 1, 2, or 3. Yet, the linear plot of lg *k*_2_(4) *vs.* lg *k*_2_(1) shows constant selectivity (slope = 0.985). Rather, we interpret the different slopes in Fig. 5A–C to be a result of the different hybridisation of the electrophilic centres, which differentiate the sp^2^-hybridised reactive sites in 1 and 4 from those in the sp-hybridised 2 and 3.

#### Correlation with Brønsted basicities of phosphines

Comparison with published physico-chemical data (Table 3) or reactivity descriptors for PR_3_ shows that the relative reactivity ordering for tertiary phosphines derived from reactions with the Michael acceptors 1–3 (*cf.*Table 2) is also reflected by other reaction series.^20,29–32^

**Table 3:** Comparison of the reactivity of phosphines PR_3_ towards Michael acceptors 1–3 (in dichloromethane, at 20 °C) with their basicity (p*K*_aH_), nucleophilicities in reactions with ethyl iodide (EtI) and iron-complex stabilised carbocations (*N*_Fe_), ligand exchange rate constants at borane (log *k*^F^_B_), and methyl cation affinities (MCA)

PR_3_	lg *k*_2_			p*K*_aH_^a^	lg *k*_2_(EtI)^b^	*N*_Fe_ c	lg *k*^F^_B_^d^
	1	2	3				
P(pfp)_3_	−4.65	−2.46	−2.54	1.97	—	1.3	−2.17
PPh_3_	−3.81	−2.12	−1.98	2.73 (7.62)	−4.42	1.95	−2.59
P(ani)_3_	−3.03	−1.42	−1.26	4.57 (10.06)	−3.57	2.9	−3.47
PMePh_2_	−2.78	−1.21	−1.20	4.65 (9.97)	—	—	−3.46
PMe_2_Ph	−1.70	−0.57	−0.53	6.49 (12.64)	−3.12	(3.3)^e^	−4.46
PBu_3_	−1.23	−0.20	−0.02	8.43	−2.79	3.6	−5.59
PMe_3_	−0.91	−0.01	−0.02	8.65 (15.48)	−2.65	—	−5.44
PCy_3_	−1.42	−0.92	+0.16	9.70	−2.68	—	−5.60
POct_3_	−1.26	−0.14	+0.43	9.03^f^	—	—	—

a Acidities of R_3_P^+^H refer to H_2_O as reported in ref. 29a and 30, values in parentheses are acidities of R_3_P^+^H in MeCN as reported in ref. 20.

b Calculated from second-order rate constants *k*_2_ (M^−1^ s^−1^) for reactions of PR_3_ with ethyl iodide in acetone at 35 °C reported in ref. 29b.

c Phosphine nucleophilicities *N*_Fe_ towards iron-complex stabilised carbocations from ref. 31.

d Calculated from the rate constants *k*^F^_B_ (M^−1^ s^−1^) for the ligand exchange of R_3_P in R_3_P→BH_3_ complexes by quinuclidine in toluene at 30 °C reported in ref. 32.

e *N*_Fe_ of PEt_2_Ph is used because *N*_Fe_ for PMe_2_Ph has not been determined.

f Calculated by DFT methods (ESI).

**Fig. 6:**
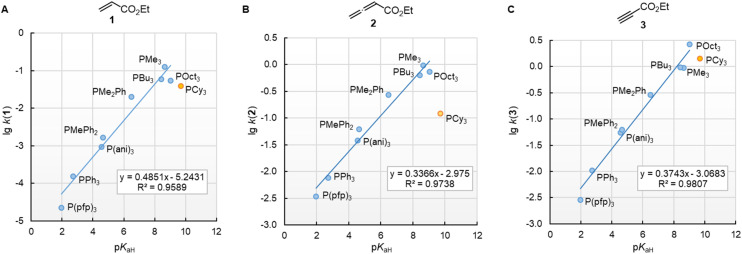
Linear relationships of the second-order rate constants (lg *k*_2_, at 20 °C in dichloromethane) for reactions of R_3_P with the Michael acceptors (A) ethyl acrylate (1), (B) ethyl allenoate (2), and (C) ethyl propiolate (3) and the Brønsted basicities p*K*_aH_(H_2_O) of the phosphines R_3_P (with data from Table 3, data for the sterically encumbered PCy_3_ excluded when constructing the correlation lines).

The nucleophilic reactivities of amines towards C-centred electrophiles have repeatedly been reported to correlate only poorly with their Brønsted basicities (p*K*_aH_).^33,34^ In contrast, the second-order rate constants for the attack of phosphines PR_3_ at Michael acceptors 1–3 in dichloromethane are linearly related to the phosphine basicities in water (Fig. 6). PCy_3_ deviates negatively from the correlation lines constructed for the remaining PR_3_ nucleophiles, and the reaction of PCy_3_ with the allenoate 2 is by more than a factor of 10 slower than expected based on its p*K*_aH_. The deviation of PCy_3_ from the correlation lines is less prominent for both electrophiles with a terminal reaction centre. Because available data for p*K*_aH_(MeCN) and p*K*_aH_(H_2_O) of PR_3_ correlate linearly (*r*^2^ = 0.9997, *n* = 5, ESI, Fig. S1†), we can assume that correlations of our reactivity data with p*K*_aH_(H_2_O) will also hold in aprotic polar solvents and will allow chemists to predict the reactivities of further sterically unencumbered phosphines towards neutral electrophiles. The slopes in the range of 0.49 to 0.34 indicate that only a part of the thermodynamic driving force of the protonation reactions is seen in the kinetics of PR_3_ additions to Michael acceptors.

#### Correlation with nucleophilicities of phosphines in S_N_1 and S_N_2 reactions

**Fig. 7:**
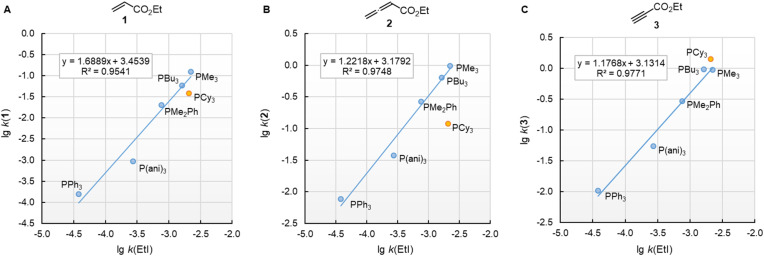
Reactivities (lg *k*_2_) of PR_3_ towards (A) ethyl acrylate (1), (B) ethyl allenoate (2), and (C) ethyl propiolate (3) correlate linearly with the S_N_2 reactivity of PR_3_ towards ethyl iodide (in acetone at 35 °C, ref. 29b). With rate constants *k*_2_ from Table 3, data for the sterically encumbered PCy_3_ excluded when constructing the correlation lines.

The nucleophilicity of PR_3_ phosphines was previously characterised by investigating the kinetics of ethylation reactions (with ethyl iodide) in acetone at 35 °C (Table 3).^29*b*^ The rate constants that we determined in this work for addition reactions of PR_3_ to electron-deficient neutral π-systems correlate linearly with the S_N_2 reactivities of tertiary phosphines towards ethyl iodide, lg *k*(EtI) (Fig. 7). For the electrophiles 1 and 3, the data point of PCy_3_ is close to the respective correlation line, which illustrates the similar steric demand for reactions of PR_3_ at terminal –CH_2_X, =CH_2_, and ≡CH groups. The slope of the correlation line is 1.7 for the olefinic Michael acceptor 1 (Fig. 7A), close to the typical slope of 2 observed when comparing nucleophile reactivities in S_N_1 reactions with those in S_N_2 reactions.^35–37^ The correlation lines for the sp-hybridised electrophiles 2 and 3 are more shallow. Their slopes in the range of 1.2 (Fig. 7B/C) are caused by the higher degree of reorganisation required to change the hybridisation at the reaction centre from a linear to a trigonal planar geometry.

**Fig. 8:**
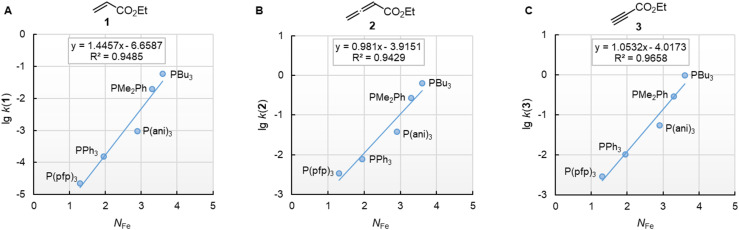
Reactivities (lg *k*_2_) of PR_3_ towards (A) ethyl acrylate (1), (B) ethyl allenoate (2), and (C) ethyl propiolate (3) correlate linearly with *N*_Fe_, which are nucleophilicity parameters for phosphines derived from reactions of PR_3_ with cationic electrophiles structurally similar to [Fe(CO)_3_(C_6_H_7_)]^+^, from ref. 31 and 38 With rate constants *k*_2_ from Table 3. For the PMe_2_Ph entries, the *N*_Fe_ of PEt_2_Ph was used. The correlation for ESF (4) is shown in Fig. S2 (ESI).†

Furthermore, rate constants of addition reactions of phosphines PR_3_ to iron-complex stabilised carbocations, such as [Fe(CO)_3_(C_6_H_7_)]^+^, were reported.^31,38^ These kinetic data were used by Kane-Maguire, Honig, and Sweigart to derive *N*_Fe_ parameters (Table 3), which describe the averaged nucleophilicity of a PR_3_ reagent towards such cationic complexes.^31^ The graphs in Fig. 8 demonstrate that the phosphine reactivities determined for reactions with 1, 2, and 3 are linearly related with the *N*_Fe_ descriptors.

Thus, the rate constants determined in this work for the reactions of PR_3_ phosphines with neutral Michael acceptors correlate both with reported phosphine reactivities towards S_N_2 and S_N_1 substrates. Given that the molecular structures of ethyl iodide and [Fe(CO)_3_(C_6_H_7_)]^+^ ion are unlike the Michael acceptors studied in this work, we can conclude that the reactivities of the PR_3_ nucleophiles determined towards Michael acceptors 1–3 are generally applicable.

#### Correlation with borane-nucleofugalities of phosphines

There is no general relationship between nucleophilicity and nucleofugality (or Lewis basicity).^39^ Several classes of nucleophiles, such as DABCO,^40,41^ other tertiary alkylamines,^34^ thioethers,^42^ or iodide and cyanide ions, have been reported to be good nucleophiles and excellent nucleofuges owing to their little need for reorganisation and low Marcus intrinsic barriers.^43^ However, other classes of compounds are good nucleophiles but weak nucleofuges.^44^ Quite often, only a few experimentally determined data exist for either of the two reaction directions. As a consequence, assessing Marcus intrinsic barriers is impossible and predictions of philicity/fugality relationships become infeasible.

The isoelectronic relation between H_3_C-X and [H_3_B-X]^−^ triggered our interest to compare the reactivities of the phosphines PR_3_ at carbon with those at boron centres. Recently, Lloyd-Jones and colleagues studied the rate constants of quinuclidine displacement of R_3_P–BH_3_ adducts in toluene at 30 °C.^32^ They reported mechanistic evidence consistent with an S_N_2-like process at the boron-centre. The Lloyd-Jones group also derived an increment system of ‘ligand nucleofugality values 
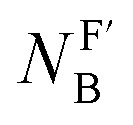
 for phosphines PR_3_ to describe the structural factors that influence the leaving group abilities. The *N*^F^_B_ values correlate excellently with the p*K*_aH_ values of PR_3_ in water. The linear relationship spans over a range of 11 p*K*_a_ units (*n* = 12, *r*^2^ = 0.9956) and comprises P(pfp)_3_ as the least basic and PCy_3_ as the most basic phosphine.^32^

**Fig. 9:**
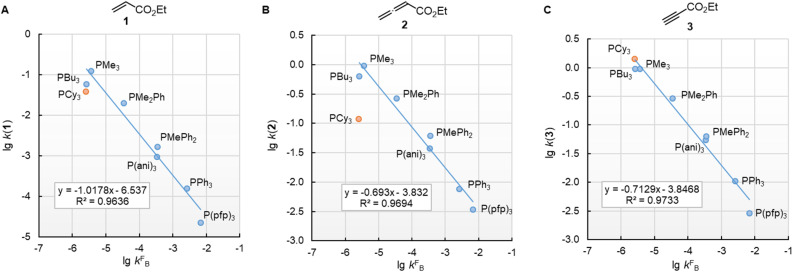
Correlation of PR_3_ reactivity (lg *k*_2_ in dichloromethane) towards (A) ethyl acrylate (1), (B) ethyl allenoate (2), and (C) ethyl propiolate (3) with the ligand nucleofugality of R_3_P from R_3_P→BH_3_ complexes (external nucleophile: quinuclidine, in toluene at 30 °C) from ref. 32. With rate constants *k*^F^_B_ from Table 3, data for PCy_3_ excluded when constructing the correlation lines.

In this work, we found that also the reactivities of phosphines towards Michael acceptors correlate linearly with their p*K*_aH_ values (*cf.*Fig. 6). Thus, the stage was set for establishing a relationship between nucleophilicities and nucleofugalities of PR_3_ by combining the rate constants for adduct formation of PR_3_ with Michael acceptors with the rate constants for the quinuclidine displacement of PR_3_ in R_3_P-borane complexes (*k*^F^_B_).^32^Fig. 9 shows an inverse linear correlation for the phosphines in both reaction series. The weakest nucleophile P(pfp)_3_ is the most reactive nucleofuge, and the relation is *vice versa* for the highly nucleophilic PMe_3_ or PBu_3_. Depending on the steric environment at the electrophilic centres of the Michael acceptors, PCy_3_ is close to the linear correlation for the sterically unencumbered PR_3_ species (as for 1 and 3) or has been determined to be a weaker nucleophile than expected on the basis of its nucleofugality lg *k*^F^_B_ (as for 2).

### Quantum-chemical analysis

Previous quantum-chemical studies of the PMe_3_/methyl allenoate addition in benzene gave significantly different results: the addition was reported to be almost thermoneutral^45*b*^ or endergonic (Δ*G*_add_ = +40.6 kJ mol^−1^).^25,45*a*^ This ambiguity in calculating the driving force of a relatively simple model reaction is an indication of the importance of the computational methods used. Hence, we started by investigating the influence of quantum-chemical methods on the thermodynamics of the PMe_3_ addition to ethyl allenoate (2) by using different basis sets, electronic structure methods, and solvation models (see ESI† for details). We found that the combination of the MN15 functional with the triple-ζ basis set def2-TZVPP and the implicit solvation model SMD showed reliable performance. Hence, this combination was used for all quantum-chemical calculations performed in this work.^46^

**Scheme 5:**
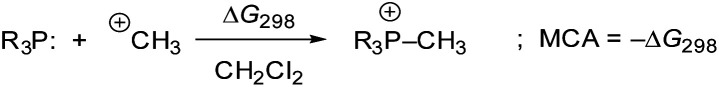
Reaction scheme for the calculation of PR_3_ methyl cation affinities (MCA) in dichloromethane.

Methyl cation affinities (MCA)^47,48^ often characterise the reactivity of nucleophiles (Nuc:) towards C-centred electrophiles^49^ better than p*K*_aH_ values, which reflect the thermodynamics of ^+^Nuc–H bond formations. We, therefore, calculated MCAs for phosphines PR_3_, as shown in Scheme 5, from the Gibbs reactions energies of methylation reactions in dichloromethane (MCA = −Δ*G*_298_) (see Table 4 and ESI† for details).

**Table 4:** Methyl cation affinities (MCA), experimentally determined reaction barriers (Δ*G*^‡^_exp_) as well as quantum-chemically calculated reaction barriers (Δ*G*^‡^_calc_) and reaction energies (Δ*G*_add_) for the addition of phosphines PR_3_ to the Michael acceptors 1, 2 and 3 in dichloromethane (all energies in kJ mol^−1^)

PR_3_	MCA^a^	Ethyl acrylate (1)			Ethyl allenoate (2)			Ethyl propiolate (3)		
		Δ*G*^‡^_exp_^b^	Δ*G*^‡^_calc_^c^	Δ*G*_add_^d^	Δ*G*^‡^_exp_^b^	Δ*G*^‡^_calc_^c^	Δ*G*_add_^d^	Δ*G*^‡^_exp_^b^	Δ*G*^‡^_calc_^c^	Δ*G*_add_^d^
P(pfp)_3_	402.8	97.9	90.8	73.0	85.6	88.6	7.7	86.0	89.8	29.3
PPh_3_	418.6	93.1	89.9	68.5	83.6	87.7	5.5	82.9	87.9	27.7
P(ani)_3_	425.7	88.8	86.2	61.1	79.8	87.5	−0.8	78.9	84.6	18.6
PMePh_2_	434.5	87.4	86.5	51.8	78.6	87.8	−10.8	78.5	86.7	18.1
PMe_2_Ph	448.6	81.3	80.6	33.9	75.0	83.7	−23.3	74.8	82.2	4.6
PBu_3_	459.7	78.7	79.2	32.2	72.9	83.6	−31.8	71.9	79.7	−3.6
PMe_3_	466.1	76.8	79.2	22.0	71.8	82.7	−33.6	71.9	80.2	−5.9
PCy_3_	473.0	79.7	81.5	34.7	76.9	81.9	−7.1	70.9	71.9	−7.2

a MCA (= −Δ*G*_298_) calculated according to Scheme 5 at the SMD(DCM)/MN15/def2-TZVPP level of theory at 298.15 K.

b Gibbs activation energies Δ*G*^‡^_exp_ calculated from the experimentally determined second-order rate constants *k*_2_ (20 °C) in Table 2 by using the Eyring equation.

c Gibbs activation energies Δ*G*^‡^_calc_ of the reactions in Scheme 6 calculated at the SMD(DCM)/MN15/def2-TZVPP level of theory at 298.15 K.

d Gibbs reaction energies Δ*G*_add_ of the reactions in Scheme 6 calculated at the SMD(DCM)/MN15/def2-TZVPP level of theory at 298.15 K.

**Fig. 10:**
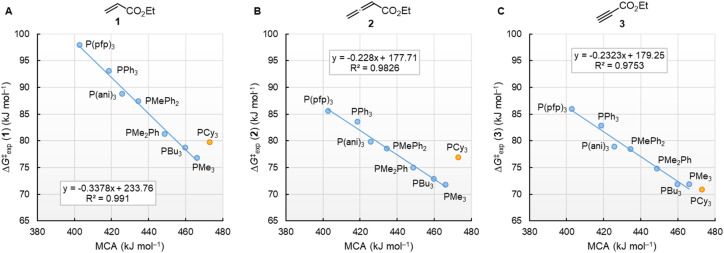
Correlation of experimental Gibbs activation energies Δ*G*^‡^_exp_ with computed MCA values of PR_3_ additions to (A) ethyl acrylate (1), (B) ethyl allenoate (2), and (C) ethyl propiolate (3) in dichloromethane. With energies from Table 4, data for the sterically encumbered PCy_3_ excluded when constructing the correlation lines.

Fig. 10 illustrates that the experimentally determined Gibbs activation energies Δ*G*^‡^_exp_ of PR_3_ additions to Michael acceptors 1, 2, and 3 (20 °C, CH_2_Cl_2_), except for PCy_3_, correlate linearly with the quantum-chemically calculated MCAs. Thus, we can conclude that the easily calculated thermodynamics of methylation reactions can be used to predict relative nucleophilicities of sterically unencumbered PR_3_ also towards other classes of C-electrophiles, such as electron-deficient π-systems.

By using the same DFT level of theory as for the MCA calculations, we then analysed the energetics of PR_3_ additions to Michael acceptors 1, 2, and 3 (Scheme 6) by calculating the reaction barriers Δ*G*^‡^_calc_ and the Gibbs reaction energies for the addition step Δ*G*_add_ (Table 4).

**Scheme 6:**
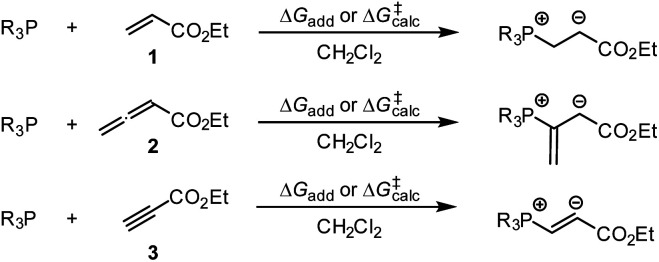
Gibbs activation (Δ*G*^‡^_calc_) and reaction energies (Δ*G*_add_) of PR_3_ additions to Michael acceptors 1–3.

**Fig. 11:**
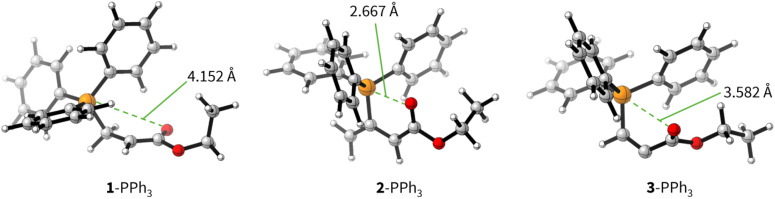
Molecular structures of the zwitterionic adducts of PPh_3_ with 1, 2, and 3 optimised at SMD(DCM)/MN15/def2-TZVPP level of theory. Green dashed lines indicate relevant P–O distances in the adducts.

The positive Δ*G*_add_ values for PR_3_ additions to 1 (Table 4) are in accord with the experimentally observed reversibility of these reactions. For 2 and particularly 3 only the most reactive and Lewis basic phosphines react exergonically. In general, PR_3_ additions to the allenoate 2 are energetically more favourable than the corresponding reactions of phosphines with 1 or 3. We rationalise the differences in the stability of the zwitterionic PR_3_-adducts derived from 1, 2, and 3 by the variable extent of attractive P⋯O interactions in the adducts.^25^Fig. 11 depicts the optimised geometries of the adducts of 1, 2, or 3 with PPh_3_, the most relevant phosphine in organocatalysis. The computed P–O distances in the PPh_3_ adducts of 2, 3, and 1 follow the trend seen in Δ*G*_add_: the shorter the P–O distance the more stable is the adduct.

**Fig. 12:**
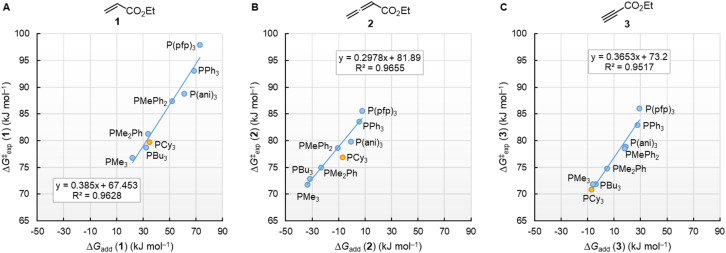
Correlations of Δ*G*^‡^_exp_ (20 °C) for PR_3_ additions to (A) 1, (B) 2, and (C) 3 in dichloromethane with the respective reaction energies Δ*G*_add_. With energies from Table 4, data for the sterically encumbered PCy_3_ excluded when constructing the correlation lines.

The correlation lines in Fig. 12 indicate that the activation barriers (Δ*G*^‡^_exp_) of phosphine additions to the vinylic, allenic, and acetylenic electrophiles decrease systematically as the thermodynamic driving forces (Δ*G*_add_) increase. However, the slopes in Fig. 12A–C reflect that only 39%, 30%, and 37% of the product stabilising effects are found in the transition states (TS) of these phospha-Michael additions.

**Fig. 13:**
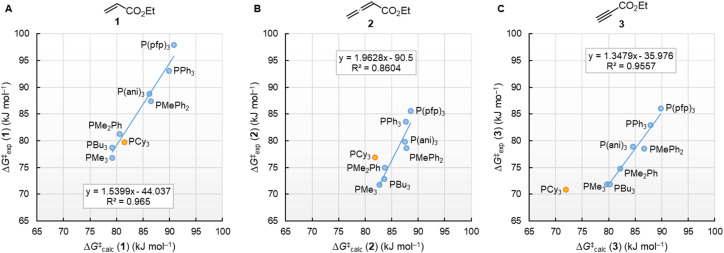
Correlation of experimentally determined Δ*G*^‡^_exp_ (20 °C) for PR_3_ additions to (A) 1, (B) 2, and (C) 3 in dichloromethane with quantum-chemically calculated Gibbs activation energies (Δ*G*^‡^_calc_). Results for PCy_3_ were excluded when calculating the regression lines.

Neglecting the effect of the small temperature difference between experimental and calculated energies (20 °C *vs.* 25 °C), the quantum-chemically calculated reaction barriers (Δ*G*^‡^_calc_) for phosphine additions to 1, 2, and 3 are within a range of ±10 kJ mol^−1^ of the experimentally determined Gibbs activation energies Δ*G*^‡^_exp_ (Table 4). The excellent linear correlations of Δ*G*^‡^_exp_ with Δ*G*^‡^_calc_ in Fig. 13 corroborate the interpretation that the experimentally measured second-order rate constants *k*_2_ reflect the initial phosphine addition to the electron-deficient reaction partners. We note, however, that the regression lines for all three Michael acceptors show slopes significantly larger than unity, which implies that the 20 kJ mol^−1^ wide range for Δ*G*^‡^_exp_ is compressed to a width of only 10 kJ mol^−1^ in the DFT calculations.

**Fig. 14:**
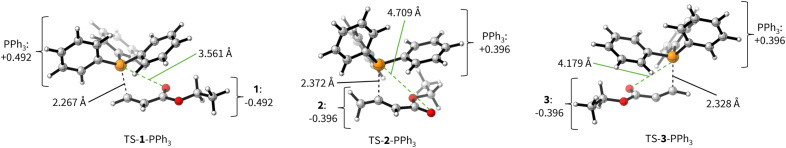
TS geometries for the addition of PPh_3_ to 1 (TS-1-PPh_3_), 2 (TS-2-PPh_3_), and 3 (TS-3-PPh_3_) with selected P–O (green dashed line) and P–C (black dashed line) distances as well as charge separation (based on cumulated NBO charges on the fragments) of PPh_3_ and the corresponding Michael acceptor (level of theory: SMD(DCM)/MN15/def2-TZVPP).

Transition state (TS) geometries for the addition of PPh_3_ to Michael acceptors 1, 2, and 3 are shown in Fig. 14. In contrast to the PPh_3_ adduct with 2, where the attractive P⋯O interaction was identified as a key stabilising factor, the P⋯O distance in the TS geometries of PPh_3_ reactions with 1, 2, and 3 are generally >3.5 Å and exceed the sum of the van der Waals radii of oxygen and phosphorus (3.22 Å, with O: 1.52 Å and P: 1.80 Å).^50^ The P–C bond formation is slightly more advanced in TS-1-PPh_3_ (P–C distance: 2.267 Å) than in TS-2-PPh_3_ (2.372 Å) or TS-3-PPh_3_ (2.328 Å), which indicates a later TS for the addition of PPh_3_ to 1 than for the analogous reaction with 2 and 3. Likewise, charge separation (NBO analysis) between PPh_3_ and the respective electrophile in the TS is found to be already larger for acrylate 1 than for 2 or 3: the cumulative partial charges in TS-1-PPh_3_ are ±0.492 and amount to only ±0.396 in TS-2-PPh_3_ and TS-3-PPh_3_, respectively. The origin of the variations in the reaction barrier (Δ*G*^‡^) were subsequently investigated by using the Marcus eqn (1) to calculate intrinsic barriers (Δ*G*^‡^_0_).^43^1
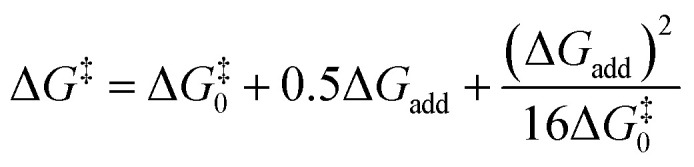


The intrinsic barriers Δ*G*^‡^_0_ (Table 5) obtained by combining experimental or theoretically calculated reaction barriers with the DFT-calculated Δ*G*_add_ show identical trends. The Δ*G*^‡^_0_ for reactions with the Michael acceptor 1, which changes hybridisation from sp^2^ to sp^3^ at the reaction centre, are significantly lower than those for analogous PR_3_ additions to 2 and 3, which involve the need for a higher degree of reorganisation owing to the change from sp- to sp^2^-hybridisation at the electrophilic centre (see Table 5). Nevertheless, the more favourable reaction energies Δ*G*_add_ for PR_3_ additions to 2 and 3 give rise to the overall lower reaction barriers (Δ*G*^‡^) and thus faster reaction rates, despite higher intrinsic barriers than for PR_3_ additions to the acrylate 1.

**Table 5:** Computed intrinsic barriers for addition of phosphines to 1, 2 and 3 according to eqn (1). Δ*G*^‡^_0,calc_ refers to intrinsic barriers calculated with computed reaction barriers (SMD(DCM)/MN15/def2-TZVPP data). Δ*G*^‡^_0,exp_ refers to the use of experimentally determined reaction barriers in eqn (1)

PR_3_	Ethyl acrylate (1)		Ethyl allenoate (2)		Ethyl propiolate (3)	
	Δ*G*^‡^_0,exp_	Δ*G*^‡^_0,calc_	Δ*G*^‡^_0,exp_	Δ*G*^‡^_0,calc_	Δ*G*^‡^_0,exp_	Δ*G*^‡^_0,calc_
P(pfp)_3_	55.4	47.3	81.7	84.7	70.6	74.4
PPh_3_	53.4	49.8	80.8	84.9	68.3	73.4
P(ani)_3_	53.9	51.1	80.2	87.9	69.3	75.0
PMePh_2_	58.6	57.7	83.9	93.1	69.2	77.4
PMe_2_Ph	63.2	62.5	86.3	95.0	72.5	79.9
PBu_3_	61.6	62.1	88.1	98.9	73.7	81.5
PMe_3_	65.3	67.8	87.8	98.8	74.8	83.1
PCy_3_	61.1	63.0	80.4	85.4	74.5	75.5

**Fig. 15:**
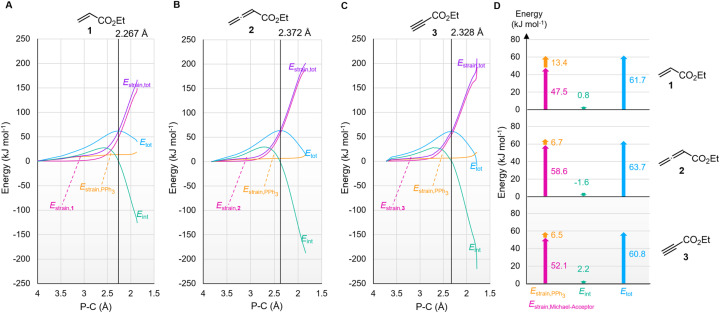
Activation strain analyses for the addition of PPh_3_ to 1 (A), 2 (B) and 3 (C). Deformation energies of the Michael acceptor (magenta) and PPh_3_ (orange) and the total deformation (purple) as well as interaction energies (green) and relative energy of a molecular complex along the reaction coordinate (blue) are depicted. The TS is highlighted by the vertical grey line. (D) Distortion–interaction analysis of the TS corresponding to (A)–(C) (level of theory: SMD(DCM)/MN15/def2-TZVPP).

The origin of the characteristic differences in the intrinsic barriers in the reaction series for 1 (Δ*G*^‡^_0,exp_ = 53 to 65 kJ mol^−1^), 2 (80 to 88 kJ mol^−1^), and 3 (68 to 75 kJ mol^−1^) were further scrutinised by analysing deformation energies according to the activation strain model.^51^ The P–C distance (highlighted in the TS geometries in Fig. 14) was used as the reaction coordinate to analyse the activation strain energetics for the addition of PPh_3_ to Michael acceptors 1, 2, and 3 (Fig. 15). The calculated overall deformation energies (*E*_strain,tot_) are dominated by the deformation energies (*E*_strain_) of the electrophiles 1–3, while the deformation energies of PPh_3_ are comparatively small. The deformation energy for Michael acceptor 1 (47.5 kJ mol^−1^) is significantly smaller than *E*_strain_ for Michael acceptors 2 (58.6 kJ mol^−1^) or 3 (52.1 kJ mol^−1^), in accord with the ordering of the Marcus intrinsic barriers in Table 5 (53.4 kJ mol^−1^ for 1 + PPh_3_, 68.3 kJ mol^−1^ for 3 + PPh_3_, and 80.8 kJ mol^−1^ for 2 + PPh_3_). For all reactions in Fig. 15, the interaction energies (*E*_int_) are destabilising at early stages of the P⋯C bond-formation and only become stabilising when approaching the TS region.

## Conclusion

Phosphine additions to electron-deficient π-systems play a key role in many Lewis-base catalysed organic reactions. In this work, we determined second-order rate constants *k*_2_ for the additions of differently substituted tertiary phosphines PR_3_ to ethyl acrylate, ethyl allenoate, and ethyl propiolate in dichloromethane at 20 °C. The reactivities of PR_3_ quantified in this way correlate linearly with a range of PR_3_ properties, for example their S_N_2 and S_N_1 reactivities towards other types of electrophilic reaction partners or their Brønsted and Lewis basicities. In addition, the experimentally determined Gibbs activation energies correlate with theoretically calculated barriers for the phospha-Michael additions as well as with theoretically calculated reaction energies in dichloromethane (SMD solvent model) suggesting the potential to anchor future quantum-chemical modeling of PR_3_ reactions to experiments.

**Fig. 16:**
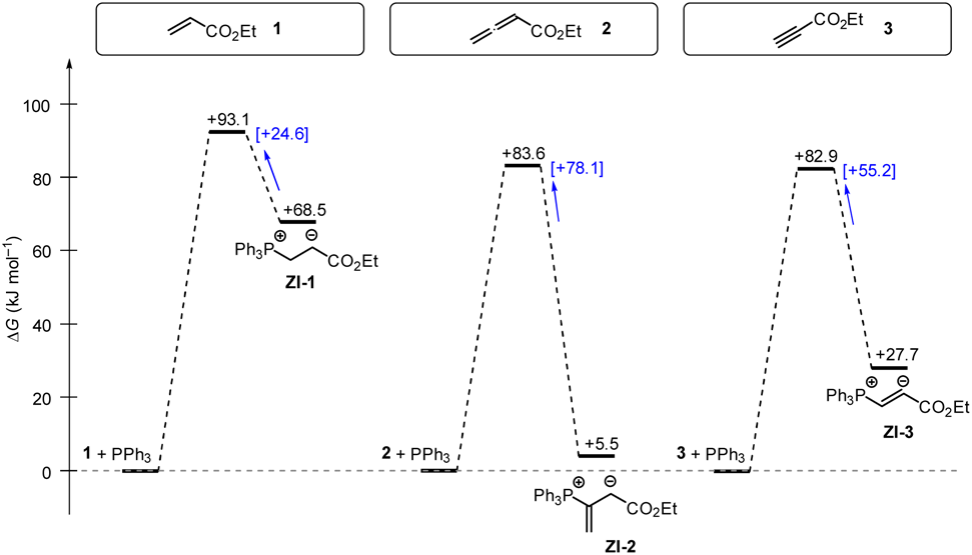
Gibbs energy profiles for PPh_3_ additions to Michael acceptors 1, 2 and 3 in dichloromethane solution. Experimentally determined Gibbs activation energies Δ*G*^‡^_exp_ are combined with quantum-chemically calculated reaction energies Δ*G*_add_ (data from Table 4). Reaction barriers for the retro-additions [Δ*G*^‡^_retro_] are given in square brackets.

Gibbs energy profiles for the phospha-Michael addition reactions can be constructed from the experimental Gibbs activation energies (Δ*G*^‡^) and the DFT-calculated Gibbs reaction energies (Δ*G*_add_). Fig. 16 shows the energy profiles for reactions of 1, 2, or 3 with PPh_3_, which is the most frequently used phosphine catalyst in organocatalytic transformations. The energy profiles for PPh_3_ additions to acrylate 1 and allenoate 2 (or propiolate 3) immediately reveal that the addition barriers are surprisingly similar, while the reaction energies are largely different (Fig. 16). The by 27.4 kJ mol^−1^ higher intrinsic barrier Δ*G*^‡^_0,exp_ for the PPh_3_ addition to 2 than to 1 (*cf.*Table 5) largely compensates the effect of the higher thermodynamic driving force for the adduct formation with 2 (0.5ΔΔ*G*_add_ = 31.5 kJ mol^−1^). As a consequence, the energetic barrier for the retroaddition of the endergonic 1+PPh_3_ reaction is only 24.6 kJ mol^−1^. In contrast, the analogous dissociation of the 2 + PPh_3_-adduct proceeds over an energetic barrier of 78.1 kJ mol^−1^.

**Fig. 17:**
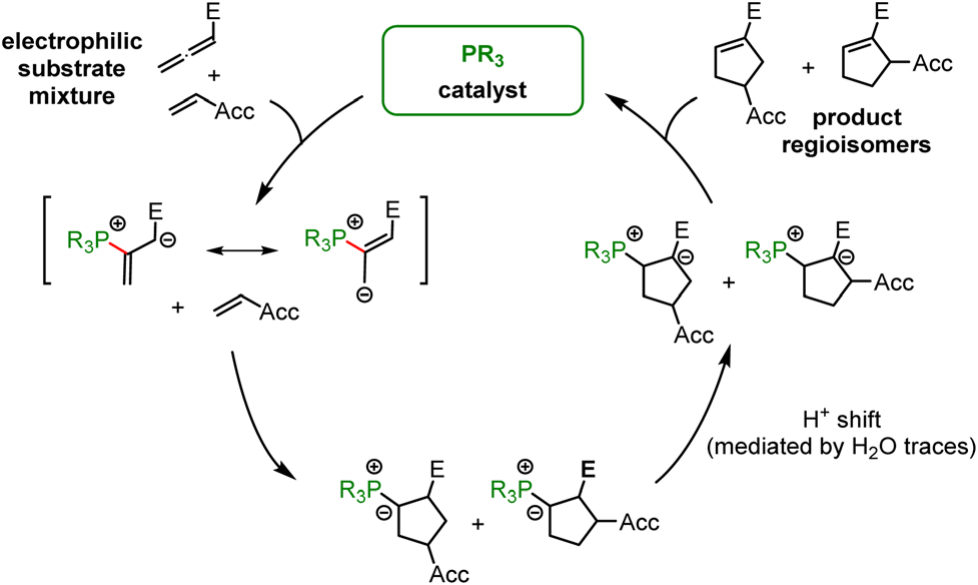
PR_3_-catalysed Lu cycloaddition (Acc = electron-accepting group, E = ester group).

In the context of multicomponent reactions, such as the Lu reaction (Fig. 17), which starts with a phosphine catalyst in a mixture of competing electrophiles, higher effective concentrations of zwitterionic PR_3_-allenoate adducts than for the analogous PR_3_-acrylate adducts may be one of the origins for the chemoselectivity of this cycloaddition.

**Fig. 18:**
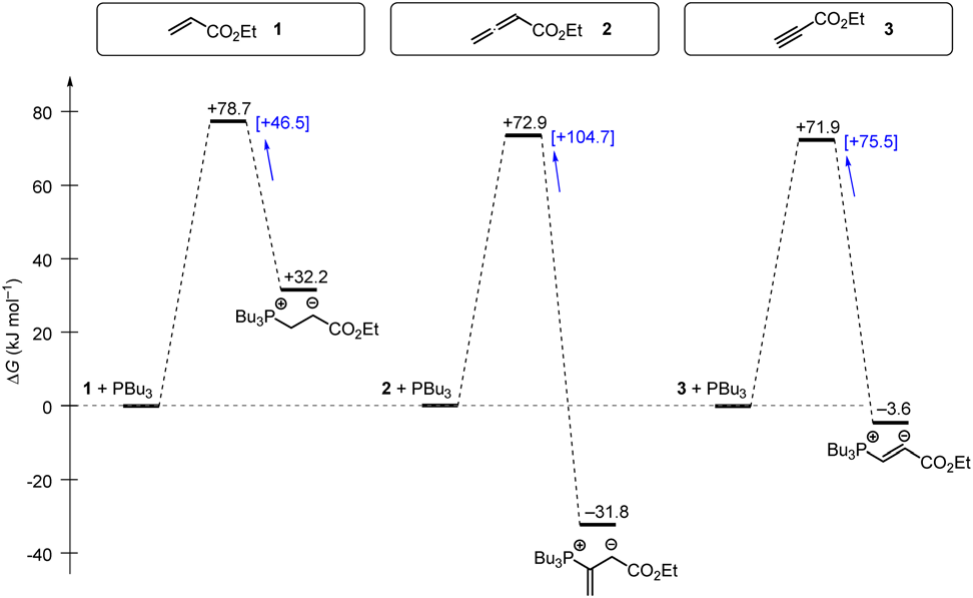
Gibbs energy profiles for PBu_3_ additions to Michael acceptors 1, 2 and 3 in dichloromethane solution. Experimentally determined Gibbs activation energies Δ*G*^‡^_exp_ are combined with quantum-chemically calculated reaction energies Δ*G*_add_ (data from Table 4). Reaction barriers for the retro-additions [Δ*G*^‡^_retro_] are given in square brackets.

Tributylphosphine PBu_3_ is more nucleophilic and Lewis basic than PPh_3_. At first glance and neglecting the practical challenges associated with handling air-sensitive catalysts, PBu_3_ might therefore appear to be a generally more effective Lewis base catalyst than PPh_3_. The reaction profile of PBu_3_ addition to allenoate 2 in Fig. 18 reveals, however, that the favourable thermodynamics for the zwitterionic adduct formations is linked to a rather large barrier for the heterolytic P–C bond cleavage. In phosphine catalysis this may imply that the final step of the catalytic cycle (*e.g.* in Fig. 17), that is the release of the PR_3_ catalyst, may become unfavourably slow. As a consequence, optimisation of reaction conditions regularly requires to keep a delicate balance between formation of a sufficient concentration of PR_3_ adducts by using highly reactive (nucleophilic) and Lewis basic phosphines and the antagonistic necessity of installing good PR_3_ nucleofuges that allow for the efficient release of the catalyst in the final step of the catalytic cycle. The combination of experimental and quantum-chemical data to characterise the philicity/fugality features of tertiary phosphines in this work may therefore be helpful to guide future attempts to use phosphine catalysis in organic synthesis.

Not all steps of phosphine-catalysed reactions are well accessible by experiment, *e.g.* in the Lu reaction. Further quantum-chemical investigations of the full cycle of phosphine-catalysed reactions are, therefore, ongoing to gain further insight in the relevant factors that need to be understood for a systematic improvement of these versatile reactions.

## Author contributions

Project conceptualisation and funding acquisition were done jointly by Y. W., M. S., H. Z. and A. R. O. Experimental methodology development and kinetic investigations were carried out by F. A. and supervised by A. R. O. Results of the kinetic measurements were formally analysed and visualised by F. A. and A. R. O. Quantum-chemical investigations, supervised by H. Z., were performed, analysed and visualised by H. J. and J. B. Results were discussed with Y. W. and M. S. The manuscript was written jointly by H. Z., J. B. and A. R. O. with input from all authors.

## Conflicts of interest

There are no conflicts to declare.

## Supplementary Material

SC-015-D4SC04852K-s001

## Data Availability

The data supporting this article have been included as part of the ESI.†
