# Supplementary material for: Reactivities of tertiary phosphines towards allenic, acetylenic, and vinylic Michael acceptors
Source: Chem Sci. 2024 Oct 14;15(43):18111–26. doi: 10.1039/d4sc04852k (PMC11474661; doi:10.1039/d4sc04852k)
Supplement: SC-015-D4SC04852K-s001 [file SC-015-D4SC04852K-s001.pdf]

Supplementary Information (SI) for Chemical Science.
This journal is © The Royal Society of Chemistry 2024

Electronic Supplementary Information

Reactivities of tertiary phosphines towards allenic, acetylenic, and vinylic Michael acceptors

Feng An,[+a] Jan Brosette,[+a] Harish Jangra,[a] Yin Wei,[b] Min Shi,[bc] Hendrik Zipse*[a] and Armin R. Ofial*[a]

[+] These authors contributed equally to this work.

[a] *Department Chemie, Ludwig-Maximilians-Universtität München, Butenandtstr. 5–13, 81377 München (Germany)*

E-Mail: zipse@cup.uni-muenchen.de (H.Z.), ofial@lmu.de (A.R.O.)

[b] *State Key Laboratory of Organometallic Chemistry, Shanghai Institute of Organic Chemistry, Chinese Academy of Sciences, 354 Fenglin Road, Shanghai 200032 (P. R. China)*

[c] *Key Laboratory for Advanced Materials and Institute of Fine Chemicals, East China University of Science and Technology, 130 MeiLong Road, Shanghai 200237 (P. R. China)*

Table of Contents

1 Additional Figures and Tables S4
2 Synthesis and Analytics S6
2.1 General S6
2.2 Syntheses and Purifications of Educts S7
2.3 Copies of NMR Spectra for Educts S14
2.3.1 Ethyl acrylate (**1**) S14
2.3.2 Ethyl 2,3-butadienoate (**2**) S15
2.3.3 Ethyl propiolate (**3**) S16
2.3.4 Ethenesulfonylfluoride (ESF, **4**) S17
2.3.5 Ethyl 2-butynoate (**5**) S18
2.3.6 Tris(4-fluorophenyl)phosphine $P(pfp)_3$ S19
2.3.7 Triphenylphosphine PPh_3 ..... S21
2.3.8 Triphenylphosphonium triflate (TPPT) S22
2.3.9 Tris(4-methoxyphenyl)phosphine $P(ani)_3$ S24
2.3.10 Methyldiphenylphosphine $PMePh_2$ S25
2.3.11 Methyldiphenylphosphonium triflate S27
2.3.12 Dimethyl(phenyl)phosphine S29
2.3.13 Dimethyl(phenyl)phosphonium triflate S31
2.3.14 Tributylphosphine S33
2.3.15 Tributylphosphonium triflate (TBPT) S34
2.3.16 Trimethylphosphonium tetrafluoroborate S36
2.3.17 Tricyclohexylphosphonium triflate S37
2.3.18 Trioctylphosphonium triflate S39
2.3.19 Tri(naphthalen-1-yl)phosphane S41
2.3.20 Triphenylphosphine oxide S42
2.3.21 Triethylamine S44
2.3.22 Triethylammonium triflate (TEAT) S45
2.3.23 2,4,6-Collidinium triflate (CT) S46
2.4 Product Studies S47
2.4.1 Reaction of triphenylphosphonium triflate (TPPT) with ethyl acrylate (**1**) S47
2.4.2 Reaction of triphenylphosphonium triflate (TPPT) with ethyl buta-2,3-dienoate (**2**) S49
2.4.3 Reaction of triphenylphosphonium triflate (TPPT) with ethyl propiolate (**3**) S53
2.4.4 Reaction of triphenylphosphonium triflate with ethenesulfonyl fluoride (**4**) S56
3 Studies on the Interactions Between Electrophiles, Nucleophiles and Proton Sources in CD_2Cl_2 ..... S60
3.1 Brønsted Basicity of PMe_2Ph and $PMePh_2$ vs 2,4,6-Collidine S60
3.1.1 $PMePh_2$ and 2,4,6-collidinium triflate (CT) S60
3.1.2 PMe_2Ph and 2,4,6-collidinium triflate (CT) S62
3.2 Interactions of Proton Sources with Electrophiles S64
3.2.1 Ethyl acrylate (**1**) with 2,4,6-collidinium triflate (CT) S64
3.2.2 Ethyl allenoate (**2**) with CT S65
3.2.3 Ethyl propiolate (**3**) with CT S66
3.2.5 Ethyl acrylate (**1**) with tributylphosphonium triflate (TBPT) S67
3.3 Stability of the Electrophiles **2** and **5** in the Presence of Triethylamine S68
3.3.1 Ethyl 2,3-butadienoate (**2**) + trimethylamine (TEA) S68
3.3.2 Ethyl 2-butynoate (**5**) + trimethylamine (TEA) S69
3.4 NMR Studies of Mixtures of TEAT with the Electrophiles **1**–**3** S70
3.4.1 Ethyl acrylate (**1**) with triethylammonium triflate (TEAT) S70

3.4.2 Ethyl 2,3-butadienoate (**2**) with triethylammonium triflate (TEAT) S71
3.4.3 Ethyl 2-butynoate (**5**) with triethylammonium triflate (TEAT) S72
3.4.4 In situ generation of trimethylphosphine PMe_3 in CD_2Cl_2 ..... S73
3.4.5 Generation of tricyclohexylphosphine PCy_3 in CD_2Cl_2 ..... S75
3.4.6 Generation of trioctylphosphine $POct_3$ in CD_2Cl_2 ..... S76
3.5 Deprotonation of Phosphonium Salts **P1** and **P2** S77
3.5.1 Deprotonation of **P1** with KO*t*Bu in d_6-DMSO S77
3.5.2 Deprotonation of **P2** with KO*t*Bu in d_6-DMSO S79
4 UV-Vis Absorption Spectra in Dichloromethane S81
4.1 General S81
4.2 UV-Vis Absorption Spectra of Phosphines PR_3 ..... S82
4.3 UV-Vis Absorption Spectra of Triethylamine, TEAT, 2,4,6-Collidine and CT S83
4.4 UV-Vis Absorption Spectra of Electrophiles S84
5 Kinetic Measurements S85
5.1 General S85
5.2 Kinetic Experiments in Dichloromethane S86
5.2.1 Kinetics of the reactions of ethyl acrylate (**1**) with PR_3 ..... S86
5.2.2 Kinetics of the reactions of ethyl 2,3-butadienoate (**2**) with PR_3 ..... S98
5.2.3 Kinetics of the reactions of ethyl propiolate (**3**) with PR_3 ..... S108
5.2.4 Kinetics of the reactions with ethenesulfonyl fluoride (ESF, **4**) with PR_3 ..... S119
5.2.5 Kinetics of the reactions with ethyl 2-butynoate (**5**) with PR_3 ..... S125
6 Theoretical Analysis S127
6.1 Prelude: Quantum-Chemical Method Validation S127
6.2 Computational Methods S132
6.3 Computational Results S133
6.3.1 Michael acceptors S133
6.3.2 Phosphines S134
6.3.3 Protonated phosphines S136
6.3.4 Methylated phosphines S137
6.3.5 Zwitterions formed by the addition of PR_3 to ethyl acrylate (**1**) S138
6.3.6 Zwitterions formed by the addition of PR_3 to ethyl allenoate (**2**) S139
6.3.7 Zwitterions formed by the addition of PR_3 to ethyl propiolate (**3**) S140
6.3.8 Transition state (TS) properties for additions of PR_3 to acrylate **1** S141
6.3.9 Transition state (TS) properties for additions of PR_3 to allenoate **2** S142
6.3.10 Transition state (TS) properties for additions of PR_3 to propiolate **3** S143
6.3.11 NBO analysis S144
6.3.12 Thermodynamics of allenoate and butynoate isomerisations S146
6.3.13 Theoretical pK_{aH} value prediction S147
6.3.14 Activation strain analysis for reactions of PPh_3 with **1**, **2**, or **3** S148
6.3.15 Intrinsic barriers S156
6.4 Cartesian Coordinates S157
7 References S213

1 Additional Figures and Tables

Table S1. Basicities (pK_{aH}) of phosphines PR_3 in water and in acetonitrile

Phosphine PR_3	pK_{aH}(H_2O)[a]	pK_{aH}(MeCN)[b]
P(ani)$_3$	4.57	10.06
PPh_3	2.73	7.62
$PMePh_2$	4.65	9.97
PMe_2Ph	6.49	12.64
PMe_3	8.65	15.48

[a] From ref. S1. [b] From ref. S2.

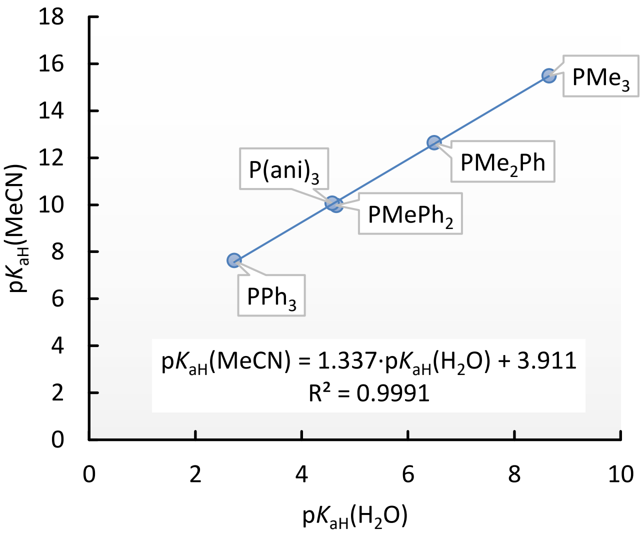

Fig. S1. Linear correlation of pK_{aH}(MeCN) with pK_{aH}(H_2O) for phosphines PR_3 (with data from Table S1).

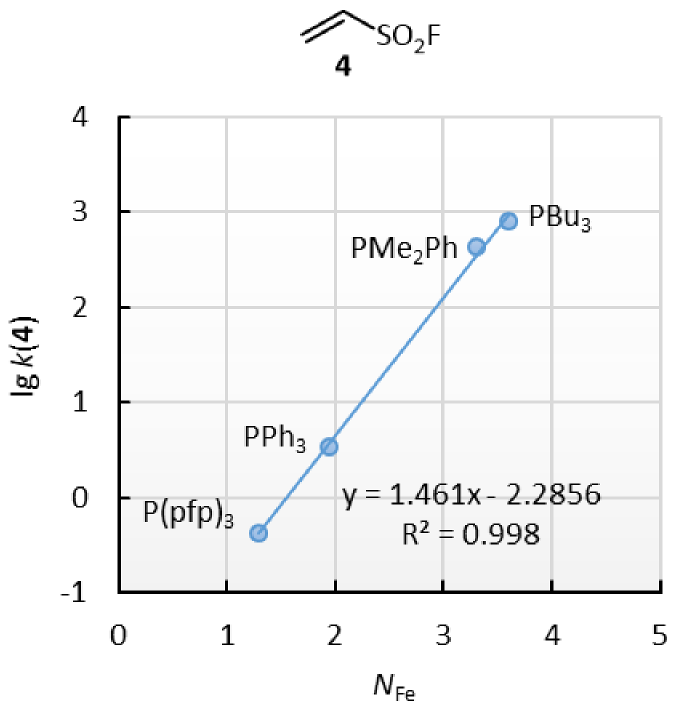

Fig. S2. Reactivities (lg k_2) of PR_3 towards ESF (**4**) correlate linearly with N_{Fe}, which are nucleophilicity parameters for phosphines derived from reactions of PR_3 with cationic electrophiles structurally similar to $[Fe(CO)_3(C_6H_7)]^+$, from ref. S3. With rate constants lg k_2(**4**) from Table 2 (main text). For the PMe_2Ph entry, the N_{Fe} of PEt_2Ph was used.

2 Synthesis and Analytics

2.1 General

Analytics. ^{1}H, ^{13}C, ^{19}F, and ^{31}P NMR spectra were measured on 400 MHz, 600 MHz, or 800 MHz NMR spectrometers. The phosphines samples in CD_2Cl_2 were prepared in a glove box under an atmosphere of dry argon gas and measured immediately after complete mixing. ^{1}H and ^{13}C NMR spectra were calibrated to residual solvent peaks.[S4] Chemical shifts are given in ppm and coupling constants *J* are reported in Hz. Multiplicities are abbreviated as follows: s = singlet, d = doublet, t = triplet, q = quartet, hept = heptet, m = multiplet, and br = broad. $^{13}C\{^{1}H\}$ NMR spectra were acquired with broad band proton decoupling. Numbers of C-attached hydrogen atoms were obtained from gHSQC experiments and are reported as C, CH, CH_2, or CH_3. The assignments of individual NMR signals were based on additional 2D-NMR experiments (gHSQC, gHMBC, and COSY). HRMS spectra were determined on a Finnigan MAT 95 mass spectrometer. IR spectra were recorded on a FTIR Spectrometer SPECTRUM BX II (Perkin Elmer). Melting points were obtained on a BÜCHI M-560 melting point apparatus and are uncorrected.

Synthesis: Flash column chromatography was performed on Merck silica gel 60 (0.040–0.063 mm) using compressed air. Thin layer chromatography (TLC) was carried out by using Merck silica gel 60 F254 aluminum plates. Compounds on eluted plates were visualised using a 254 nm UV lamp and/or by treatment with a suitable stain followed by heating. Concentration under reduced pressure was performed on rotary evaporators with a water bath temperature of 40 °C. Starting materials and reagents were purchased from Sigma-Aldrich or ABCR and were used as supplied or, in the case of some liquids, distilled. Solvents were distilled or dried prior to use over appropriate drying agents: dichloromethane (calcium hydride) and diethyl ether (sodium/benzophenone). Solvents for filtration, chromatography, and recrystallisation were purchased from Fisher and used as received.

2.2 Syntheses and Purifications of Educts

Ethyl acrylate (1) was purified by fresh distillation (bp 99.4 °C).

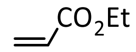

1

^{1}H NMR (400 MHz, CD_2Cl_2) δ 6.36 (dd, *J* = 17.4, 1.6 Hz, 1 H), 6.11 (dd, *J* = 17.3, 10.4 Hz, 1 H), 5.80 (dd, *J* = 10.4, 1.6 Hz, 1 H), 4.18 (q, *J* = 7.1 Hz, 2 H), 1.28 (t, *J* = 7.1 Hz, 3 H). **^{13}C{^{1}H} NMR** (101 MHz, CD_2Cl_2) δ 166.4, 130.5, 129.1, 60.8, 14.4.

Ethyl buta-2,3-dienoate (2)

Method 1: Commercially available **2** (Aldrich) was purified by column chromatography on silica gel (pentane/diethyl ether 25:1 to 15:1).

Method 2: Ethyl 2,3-butadienoate (**2**) was synthesised by a modified literature procedure reported by Hansen.[S5] For details and further analytical data see the procedure described in ref. S6.

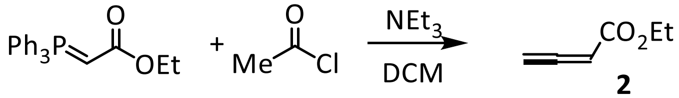

2

^{1}H NMR (400 MHz, CD_2Cl_2) δ 5.62 (t, *J* = 6.6 Hz, 1 H), 5.21 (d, *J* = 6.6 Hz, 2 H), 4.16 (q, *J* = 7.1 Hz, 2 H), 1.26 (t, *J* = 7.1 Hz, 3 H). **^{13}C{^{1}H} NMR** (101 MHz, CD_2Cl_2) δ 216.0, 165.8, 88.2, 79.3, 61.3, 14.4.

Ethyl propiolate (3) was purchased (Aldrich) and used as received.

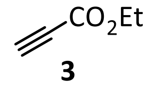

3

^{1}H NMR (400 MHz, CD_2Cl_2) δ 4.23 (q, *J* = 7.1 Hz, 2 H), 2.93 (s, 1 H), 1.29 (t, *J* = 7.1 Hz, 3 H). **^{13}C{^{1}H} NMR** (101 MHz, CD_2Cl_2) δ 153.0, 75.1, 74.5, 62.8, 14.1.

Ethenesulfonyl fluoride (ESF, 4) was purified freshly by distillation (bp. 115–116 °C) prior to use in kinetic or product studies.

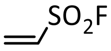

4

^{1}H NMR (400 MHz, CD_2Cl_2) δ 6.75 (ddd, $^2J_{H,H}$ = 16.6 Hz, $^3J_{H,H}$ = 9.4 Hz, $^4J_{H,F}$ = 2.1 Hz, 1 H), 6.66 (ddd, $^2J_{H,H}$ = 16.6 Hz, $^4J_{H,F}$ = 1.1 Hz,$^3J_{H,H}$ = 0.6 Hz, 1 H), 6.41 (ddd, $^3J_{H,H}$ = 9.4 Hz, $^3J_{H,F}$ = 5.3 Hz, $^3J_{H,H}$ = 0.6 Hz, 1 H). **^{13}C{^{1}H} NMR** (101 MHz, CD_2Cl_2) δ 135.4 (d, $J_{C,F}$ = 2.8 Hz), 130.3 (d, $J_{C,F}$ = 27.9 Hz). **^{19}F NMR** (377 MHz, CD_2Cl_2) δ 57.3 (ddd, $J_{F,H}$ = 5.3, 2.0, 1.0 Hz).

Ethyl 2-butynoate (5) was purchased (Aldrich) and used as received.

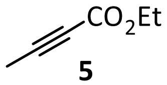

5

^{1}H NMR (400 MHz, CD_2Cl_2) δ 4.16 (q, *J* = 7.1 Hz, 2 H), 1.97 (s, 3 H), 1.26 (t, *J* = 7.1 Hz, 3 H). **^{13}C{^{1}H} NMR** (101 MHz, CD_2Cl_2) δ 153.9, 85.5, 72.6, 62.1, 14.2, 3.9.

Tris(4-fluorophenyl)phosphine P(pfp)₃ was purchased (Aldrich) and used as received.

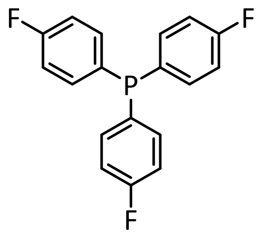

^{1}H NMR (400 MHz, CD_2Cl_2) δ 7.30–7.25 (m, 6 H), 7.10–7.05 (m, 6 H). **^{13}C{^{1}H} NMR** (101 MHz, CD_2Cl_2) δ 163.9 (d, $J_{C,F}$ = 248.7 Hz), 136.0 (dd, J = 21.4, 8.1 Hz), 133.1 (dd, J = 11.3, 3.5 Hz), 116.2 (dd, J = 21.1, 7.6 Hz). **^{31}P NMR** (162 MHz, CD_2Cl_2) δ −9.2 (q, J = 4.5 Hz). **^{19}F NMR** (377 MHz, CD_2Cl_2) δ −112.9 to −113.0 (m).

Triphenylphosphine PPh_3

PPh_3 was purified by a quick distillation (3 × 10^{-3} mbar, 140–165 °C, distillate condensed under liquid nitrogen cooling bath).[S6]

^{1}H NMR (400 MHz, CD_2Cl_2) δ 7.39–7.32 (m, 15 H). **^{13}C{^{1}H} NMR** (101 MHz, CD_2Cl_2) δ 137.8 (d, $J_{C,P}$ = 11.4 Hz), 134.1 (d, $J_{C,P}$ = 19.6 Hz), 129.1, 128.9 (d, $J_{C,P}$ = 6.9 Hz). **^{31}P NMR** (162 MHz, CD_2Cl_2) δ −5.5.

Triphenylphosphonium Triflate (TPPT) was prepared as previously reported.[S6]

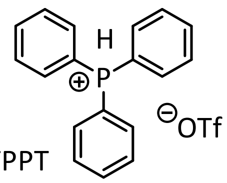

TPPT

^{1}H NMR (400 MHz, CD_2Cl_2) δ 9.47 (d, $^{1}J_{H,P}$ = 533.2 Hz, 1 H), 7.88–7.65 (m, 15 H). **^{13}C{^{1}H} NMR** (101 MHz, CD_2Cl_2) δ 135.9 (d, $J_{C,P}$ = 3.0 Hz), 134.5 (d, $J_{C,P}$ = 11.4 Hz), 130.9 (d, $J_{C,P}$ = 13.5 Hz), 121.2 (q, $J_{C,F}$ = 320.7 Hz), 116.0 (d, $J_{C,P}$ = 87.1 Hz). **^{31}P NMR** (162 MHz, CD_2Cl_2) δ 2.9 (d, $^{1}J_{P,H}$ = 533.4 Hz). **^{19}F NMR** (377 MHz, CD_2Cl_2) δ −79.0.

Tris(4-methoxyphenyl)phosphine P(ani)₃ was purchased (ABCR) and used as received.

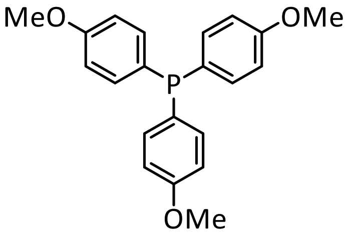

^{1}H NMR (400 MHz, CD_3CN) δ 7.22–7.17 (m, 6 H), 6.94–6.90 (m, 6 H), 3.78 (s, 9 H). **^{13}C{^{1}H} NMR** (101 MHz, CD_3CN) δ 161.3, 135.8 (d, $J_{C,P}$ = 21.1 Hz), 129.9 (d, $J_{C,P}$ = 8.6 Hz), 115.2 (d, $J_{C,P}$ = 7.6 Hz), 55.9. **^{31}P NMR** (162 MHz, CD_3CN) δ −10.7 to −10.8 (m).

Methyldiphenylphosphine $PMePh_2$ was purchased (Aldrich) and used as received.

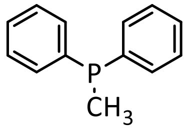

^{1}H NMR (400 MHz, CD_2Cl_2) δ 7.43–7.29 (m, 10 H), 1.62 (d, $J_{H,P}$ = 3.7 Hz, 3 H). **^{13}C{^{1}H} NMR** (101 MHz, CD_2Cl_2) δ 140.8 (d, $J_{C,P}$ = 12.7 Hz), 132.4 (d, $J_{C,P}$ = 18.5 Hz), 128.7 (d, $J_{C,P}$ = 6.4 Hz), 128.7, 12.5 (d, $J_{C,P}$ = 13.8 Hz). **^{31}P{^{1}H} NMR** (162 MHz, CD_2Cl_2) δ −27.1.

Methyldiphenylphosphonium triflate

Under an atmosphere of dry nitrogen triflic acid (323 mg, 2.15 mmol) was dissolved in anhydrous diethyl ether (20 mL) and cooled with an ice bath. Then $PMePh_2$ (429 mg, 2.14 mmol) was added dropwise. A suspension formed, which was filtered. The collected solids were washed with anhydrous diethyl ether under inert gas atmosphere. Volatiles were removed under vacuum to provide a colourless solid (587 mg, 78%).

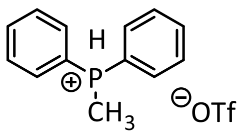

^{1}H NMR (400 MHz, CD_2Cl_2) δ 9.05–9.03 (m, 0.5 H), 7.85–7.48 (m, 10.5 H), 2.54 (dd, J = 15.1, 4.1 Hz, 3 H). **^{13}C{^{1}H} NMR** (101 MHz, CD_2Cl_2) δ 135.8 (d, $J_{C,P}$ = 3.1 Hz), 133.4 (d, $J_{C,P}$ = 11.2 Hz), 130.8 (d, $J_{C,P}$ = 13.4 Hz), 121.1 (q, $J_{C,F}$ = 320.3 Hz), 116.8 (d, $J_{C,P}$ = 87.2 Hz), 6.6 (d, $J_{C,P}$ = 54.5 Hz). **^{31}P NMR** (162 MHz, CD_2Cl_2) δ 1.6 (d, J = 527.6 Hz). **^{19}F NMR** (377 MHz, CD_2Cl_2) δ −79.0.

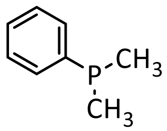

Dimethyl(phenyl)phosphine PMe_2Ph was purchased (ABCR) and used as received.

^{1}H NMR (400 MHz, CD_2Cl_2) δ 7.48–7.44 (m, 2 H), 7.37–7.33 (m, 2 H), 7.31–7.26 (m, 1 H), 1.30 (d, $J_{H,P}$ = 2.8, 6 H). **^{13}C{^{1}H} NMR** (101 MHz, CD_2Cl_2) δ 143.4 (d, $J_{C,P}$ = 13.8 Hz), 130.9 (d, $J_{C,P}$ = 17.3 Hz), 128.7 (d, $J_{C,P}$ = 5.9 Hz), 128.3, 14.7 (d, $J_{C,P}$ = 13.1 Hz). **^{31}P NMR** (162 MHz, CD_2Cl_2) δ −45.6.

Dimethyl(phenyl)phosphonium triflate

Under an atmosphere of dry nitrogen triflic acid (763 mg, 5.08 mmol) was dissolved in anhydrous diethyl ether (15 mL) and cooled with an ice bath. Then PMe_2Ph (695 mg, 5.03 mmol) was added dropwise. A suspension formed, which was filtered. The collected solids were washed with anhydrous diethyl ether under inert gas atmosphere. Volatiles were removed under vacuum to provide a colourless solid (1.34 g, 92%).

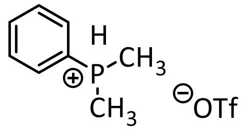

^{1}H NMR (400 MHz, CD_2Cl_2) δ 7.86–7.78 (m, 3 H), 7.71–7.66 (m, 2 H), 7.41 (dhept, $^1J_{H,P}$ = 520 Hz, $J_{H,H}$ = 5.7 Hz, 1 H), 2.26 (dd, $J_{H,P}$ = 15.4, $J_{H,H}$ = 5.7 Hz, 6 H). **^{13}C{^{1}H} NMR** (101 MHz, CD_2Cl_2) δ 135.6 (d, $J_{C,P}$ = 3.2 Hz), 132.2 (d, $J_{C,P}$ = 11.0 Hz), 130.8 (d, $J_{C,P}$ = 13.1 Hz), 121.2 (q, $J_{C,F}$ = 320.3 Hz), 117.6 (d, $J_{C,P}$ = 86.7 Hz), 6.7 (d, $J_{C,P}$ = 54.6 Hz). **^{31}P NMR** (162 MHz, CD_2Cl_2) δ 0.0 (dhept, $^1J_{P,H}$ = 520 Hz, $^2J_{P,H}$ = 15.4 Hz). **^{19}F NMR** (377 MHz, CD_2Cl_2) δ −79.0.

Tributylphosphine PBu_3 was purified by vacuum distillation (83.5 °C, 0.75 mbar).[S6]

^{1}H NMR (400 MHz, CD_2Cl_2) δ 1.41–1.33 (m, 18 H), 0.93–0.87 (m, 9 H). **^{13}C{^{1}H} NMR** (101 MHz, CD_2Cl_2) δ 28.6 (d, $J_{C,P}$ = 12.8 Hz), 27.4 (d, $J_{C,P}$ = 12.6 Hz), 25.0 (d, $J_{C,P}$ = 10.9 Hz), 14.1. **^{31}P NMR** (162 MHz, CD_2Cl_2) δ −31.3.

Tributylphosphonium triflate (TBPT) was prepared as reported previously.[S6]

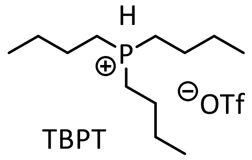

^{1}H NMR (400 MHz, CD_2Cl_2) δ 6.13 (dhept, $^1J_{H,P}$ = 480.4 Hz, $J_{H,H}$ = 5.6 Hz, 1 H), 2.30–2.21 (m, 6 H), 1.67–1.55 (m, 6 H), 1.55–1.44 (m, 6 H), 0.96 (t, J = 7.3 Hz, 9 H). **^{13}C{^{1}H} NMR** (101 MHz, CD_2Cl_2) δ 121.2 (q, $J_{C,F}$ = 320.8 Hz), 25.0 (d, $J_{C,P}$ = 4.8 Hz), 24.0 (d, $J_{C,P}$ = 15.4 Hz), 16.8 (d, $J_{C,P}$ = 46.8 Hz), 13.4. **^{31}P NMR** (162 MHz, CD_2Cl_2) δ 13.2 (dtt, $^1J_{P,H}$ = 480.9 Hz, $^2J_{P,H}$ = 25.2 Hz, $^3J_{P,H}$ = 12.0 Hz).

Trimethylphosphonium tetrafluoroborate was purchased (Aldrich) and used as received. $[HPMe_3]^+$ $[BF_4]^-$ is poorly soluble in dichloromethane at room temperature.

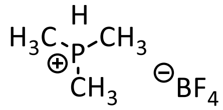

^{1}H NMR (400 MHz, CD_2Cl_2) δ 6.45 (dm, $^1J_{H,P}$ = 509.4 Hz, $J_{H,H}$ = 5.6 Hz, 1 H), 1.97 (dd, $^2J_{H,P}$ = 15.5 Hz, $J_{H,H}$ = 5.6 Hz, 9 H). **^{13}C{^{1}H} NMR** (101 MHz, CD_2Cl_2) δ 6.1 (d, $J_{C,P}$ = 55.0 Hz). **^{31}P NMR** (162 MHz, CD_2Cl_2) δ −3.5 (dm, $^1J_{P,H}$ = 509.5 Hz, $^2J_{P,H}$ = 15.6 Hz). The NMR data are consistent with those reported for $[HPMe_3]^+$ $[BF_4]^-$ in CD_3CN.[S7]

Tricyclohexylphosphonium triflate

Under an atmosphere of dry nitrogen triflic acid (466 mg, 3.11 mmol) was dissolved in anhydrous diethyl ether (3 mL) and cooled with an ice bath. Then, a solution of tricyclohexylphosphine (845 mg, 3.01 mmol) in anhydrous diethyl ether (15 mL) was added dropwise. A suspension formed, which was filtered. The collected solids were washed with anhydrous diethyl ether under inert gas atmosphere. Volatiles were removed under vacuum to provide a colourless solid (1.28 g, 99%).

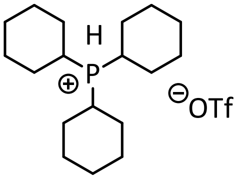

^{1}H NMR (400 MHz, $CDCl_3$) δ 6.04 (dq, $J_{H,P}$ = 474.1 Hz, $J_{H,H}$ = 4.1 Hz, 1 H), 2.49 (tdd, $J_{H,P}$ = 15.9 Hz, $J_{H,H}$ = 8.1, 3.5 Hz, 3 H), 2.05–1.76 (m, 15 H), 1.64–1.52 (m, 6 H), 1.45–1.25 (m, 9 H). **^{13}C{^{1}H} NMR** (101 MHz, $CDCl_3$) δ 120.8 (q, $J_{C,F}$ = 320.7 Hz), 28.3 (d, $J_{C,P}$ = 39.4 Hz, CH), 28.0 (d, $J_{C,P}$ = 3.6 Hz, CH_2), 26.3 (d, $J_{C,P}$ = 12.8 Hz, CH_2), 25.2 (d, $J_{C,P}$ = 1.6 Hz, CH_2). **^{31}P NMR** (162 MHz, $CDCl_3$) δ 27.6 (d, $J_{P,H}$ = 474.4 Hz). **^{19}F NMR** (377 MHz, $CDCl_3$) δ −78.3.

Trioctylphosphonium triflate

Under an atmosphere of dry nitrogen triflic acid (556 mg, 3.70 mmol) was dissolved in anhydrous diethyl ether (10 mL) and cooled with an ice bath. Then, trioctylphosphine (1.31 g, 3.53 mmol) was added dropwise. The volatiles were removed under vacuum. The residue was mixed with anhydrous pentane (10 mL) under inert gas protection and kept at −10 °C. A suspension formed, which was filtered. The collected solids were quickly washed (cold anhydrous pentane) under inert gas atmosphere. Volatiles were removed under vacuum to provide a colourless solid (521 mg, 28%).

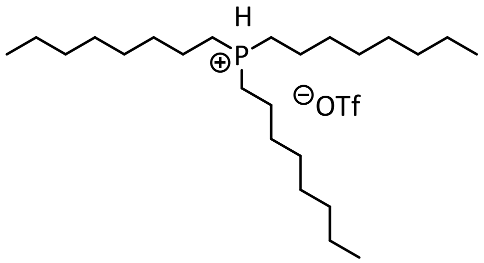

^{1}**H NMR** (400 MHz, $CDCl_3$) δ 6.19 (dm, $J_{H,P}$ = 484 Hz, 1 H), 2.30–2.21 (m, 6 H), 1.65–1.57 (m, 6 H), 1.48–1.40 (m, 6 H), 1.32–1.24 (m, 24 H), 0.86 (t, $J_{H,H}$ = 6.7 Hz, 9 H). ^{13}**C{^{1}H} NMR** (101 MHz, $CDCl_3$) δ 120.8(q, $J_{C,F}$ = 320 Hz), 31.8, 30.6 (d, $J_{C,P}$ = 14.7 Hz), 29.0, 28.9, 23.0 (d, $J_{C,P}$ = 4.8 Hz), 22.7, 16.9 (d, $J_{C,P}$ = 46.5 Hz), 14.1. ^{31}**P NMR** (162 MHz, $CDCl_3$) δ 13.0 (dtt, $J_{P,H}$ = 485.1, 25.6, 12.2 Hz).

Tri(naphthalen-1-yl)phosphane $P(1\text{-}Np)_3$ was purchased (Aldrich) and used as received.

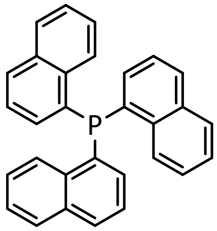

^{1}**H NMR** (400 MHz, CD_2Cl_2) δ 8.54 (ddd, J = 8.4, 4.6, 1,1 Hz, 3 H), 7.93 (dd, J = 8.0, 1.2 Hz, 3 H), 7.88 (d, J = 8.3 Hz, 3 H), 7.55-7.50 (m, 3 H), 7.49-7.39 (m, 3 H), 7.27 (ddd, J = 8.0, 7.1, 0.7 Hz, 3 H), 6.96 (ddd, J = 7.0, 4.8, 1.3 Hz, 3 H). ^{13}**C{^{1}H} NMR** (101 MHz, CD_2Cl_2) δ 136.1 (d, $J_{C,P}$ = 23.8 Hz), 134.1 (d, $J_{C,P}$ = 4.8 Hz), 133.8, 133.2 (d, $J_{C,P}$ = 11.0 Hz), 130.1, 129.1 (d, $J_{C,P}$ = 2.0 Hz), 126.7, 126.6 (d, $J_{C,P}$ = 24.8 Hz), 126.5 (d, $J_{C,P}$ = 1.5 Hz), 126.2 (d, $J_{C,P}$ = 1.5 Hz). ^{31}**P{^{1}H} NMR** (162 MHz, CD_2Cl_2) δ −34.1.

Triphenylphosphine Oxide ($Ph_3P{=}O$) was purchased (Aldrich) and used as received.

^{1}**H NMR** (400 MHz, CD_2Cl_2) δ 7.68–7.62 (m, 6 H), 7.59–7.54 (m, 3 H), 7.50–7.45 (m, 6 H).

^{13}**C{^{1}H} NMR** (101 MHz, CD_2Cl_2) δ 133.4 (d, J = 103.4 Hz), 132.3 (d, J = 9.8 Hz), 132.2 (d, J = 2.8 Hz), 128.9 (d, J = 12.0 Hz). ^{31}**P NMR** (162 MHz, CD_2Cl_2) δ 27.2.

Triethylamine was purified by fresh distillation over lithium aluminium hydride.

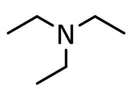

^{1}H NMR (400 MHz, CD_2Cl_2) δ 2.46 (q, *J* = 7.1 Hz, 6 H), 0.97 (t, *J* = 7.1 Hz, 9 H). **^{13}C{^{1}H} NMR** (101 MHz, CD_2Cl_2) δ 46.8, 12.2.

Triethylammonium Triflate (TEAT)

Under an atmosphere of dry nitrogen triflic acid (820 mg, 5.46 mmol) was dissolved in anhydrous diethyl ether (10 mL) and cooled with an ice bath. Then, trimethylamine (580 mg, 5.73 mmol) was added dropwise. The volatiles were removed under vacuum to provide TEAT, which was kept in the fridge. In the cool, it slowly crystallised to a white solid (1.37 g, 99%).

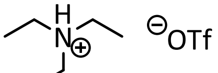

TEAT

^{1}H NMR (400 MHz, CD_2Cl_2) δ 8.54 (s, br 1H), 3.14 (q, *J* = 7.3 Hz, 6 H), 1.34 (t, *J* = 7.3 Hz, 9 H). **^{13}C{^{1}H} NMR** (101 MHz, CD_2Cl_2) δ 120.8 (q, $J_{C,F}$ = 319.4 Hz), 47.3, 8.9.

2,4,6-Collidinium Triflate (CT) was prepared as reported previously.[S6]

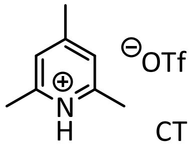

CT

^{1}H NMR (400 MHz, CD_2Cl_2) δ 14.38 (br t, $J_{H,N}$ = 53.3 Hz, 1 H), 7.28 (s, 2 H), 2.77 (s, 6 H), 2.53 (s, 3 H). **^{13}C{^{1}H} NMR** (101 MHz, CD_2Cl_2) δ 160.0, 153.3, 125.7, 120.8 (q, $J_{C,F}$ = 319.5 Hz), 22.3, 19.6.

2.3 Copies of NMR Spectra for Educts

2.3.1 Ethyl acrylate (1)

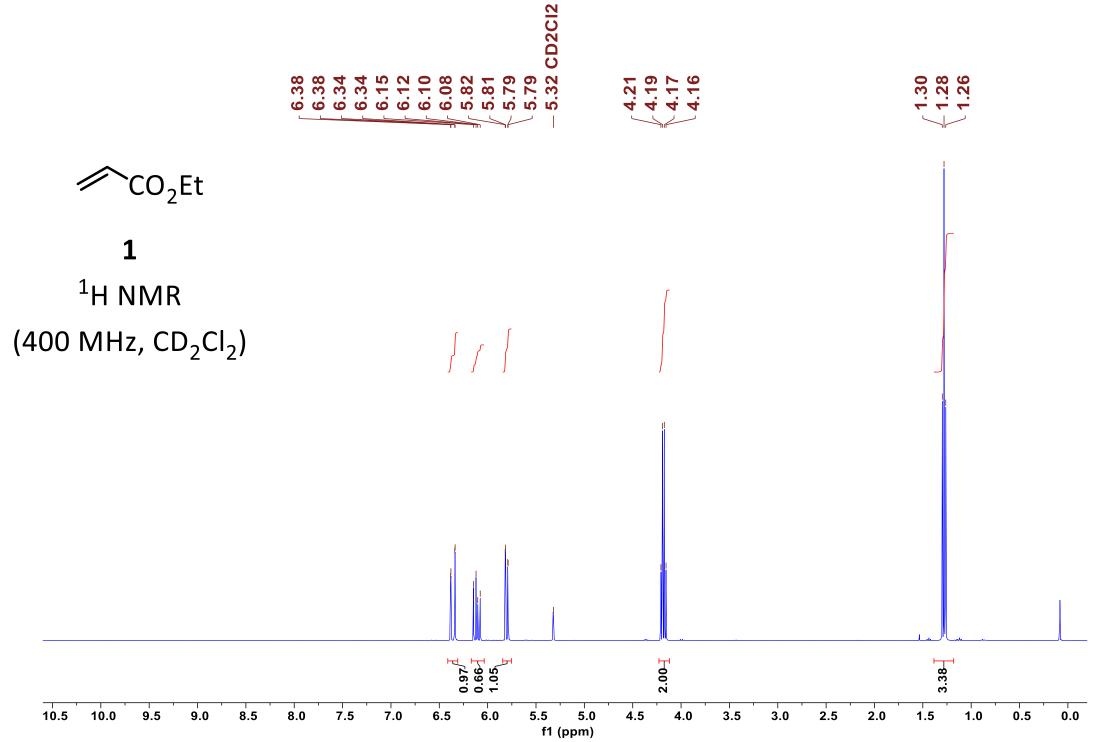

f379

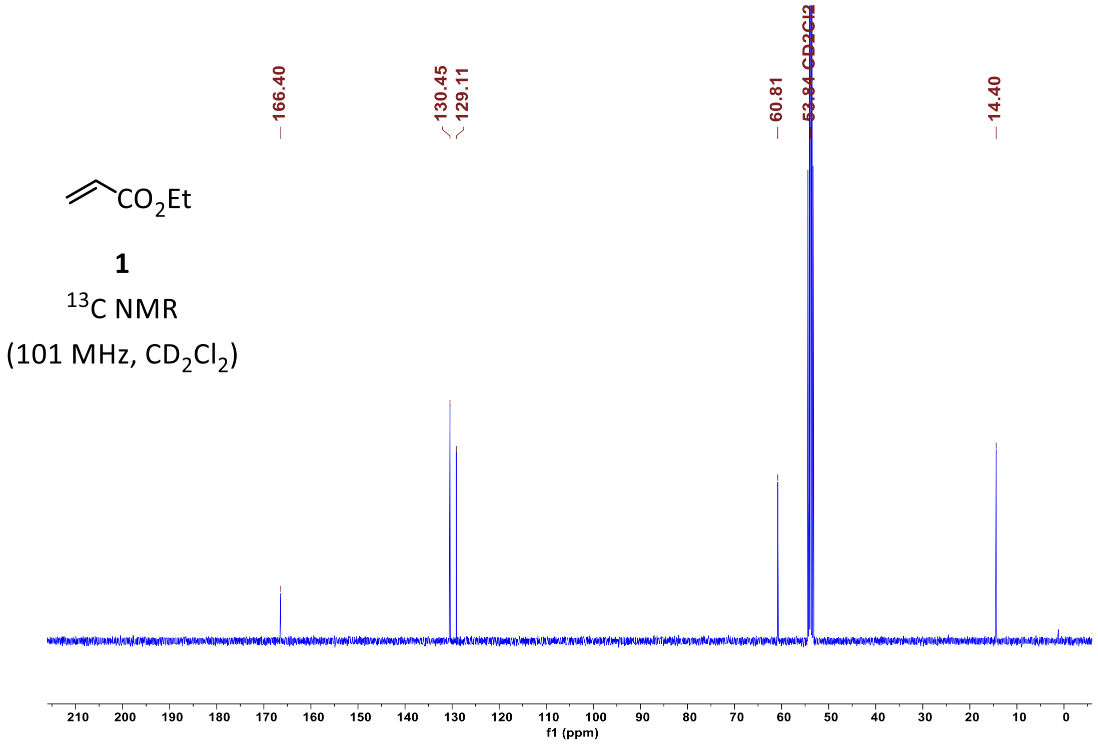

2.3.2 Ethyl 2,3-butadienoate (2)

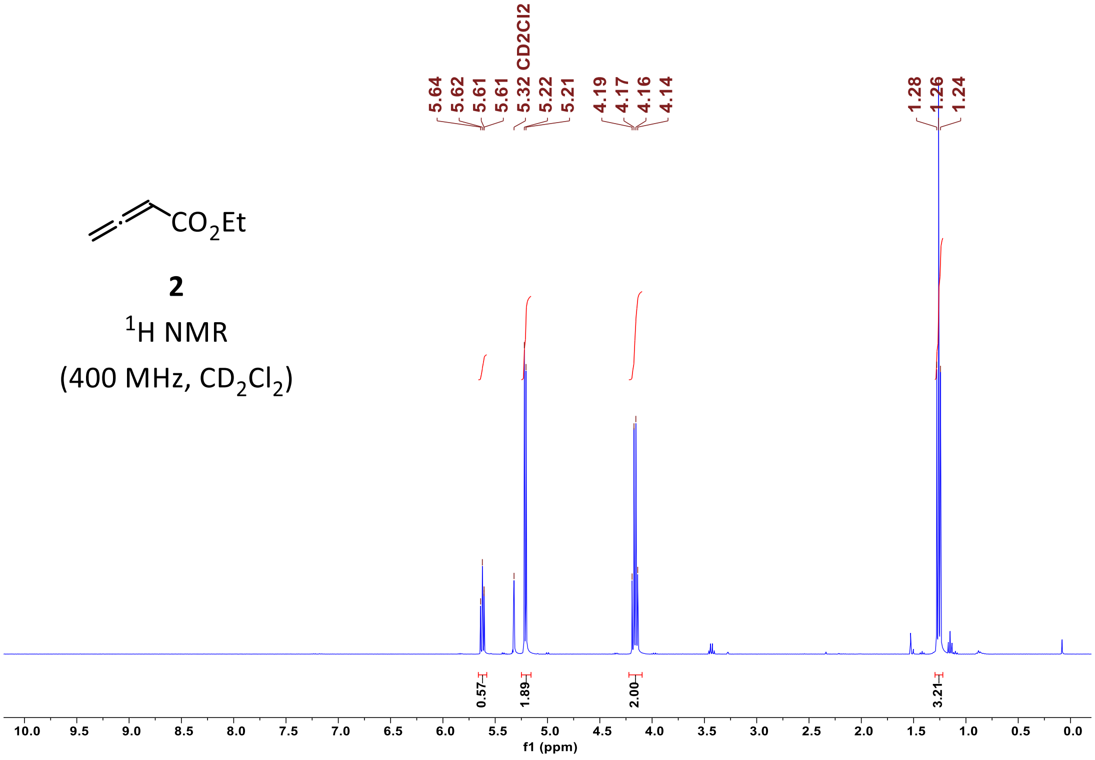

f450-02

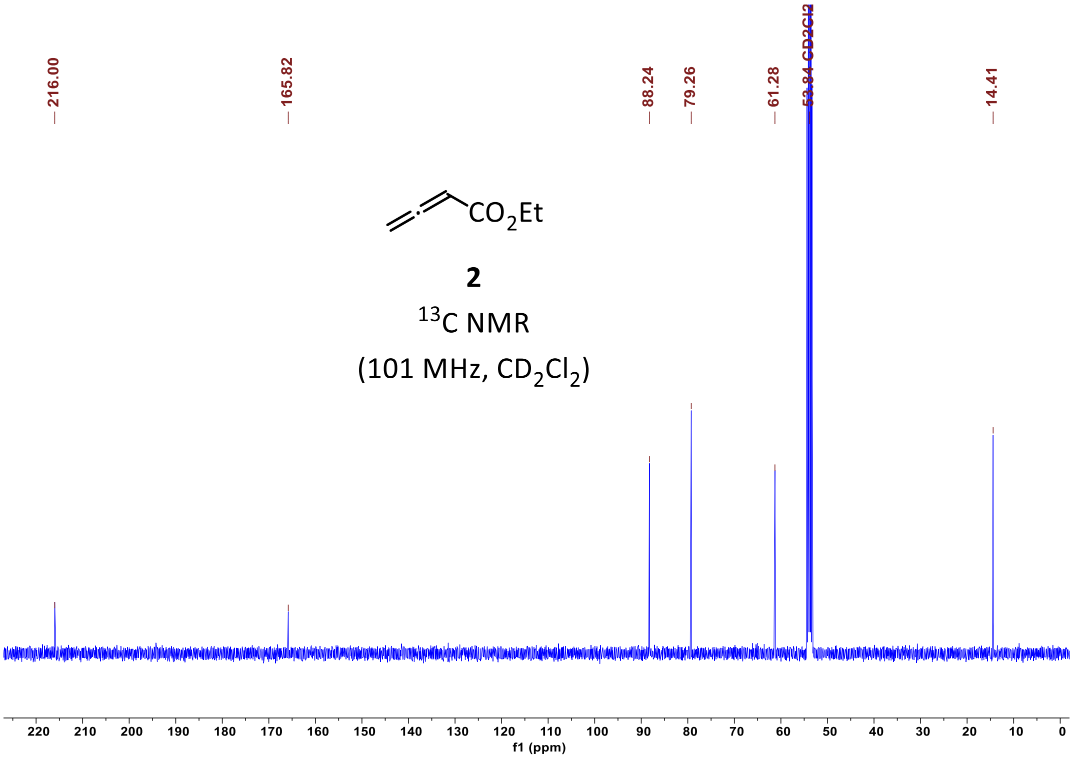

2.3.3 Ethyl propiolate (3)

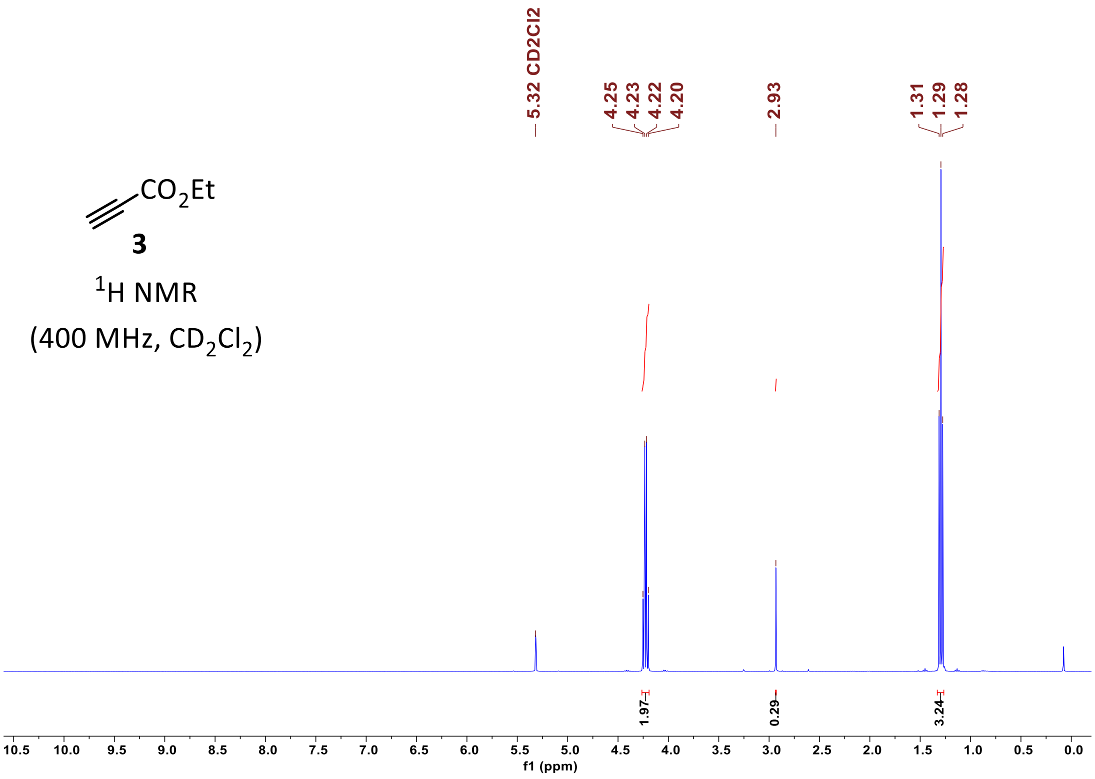

f500

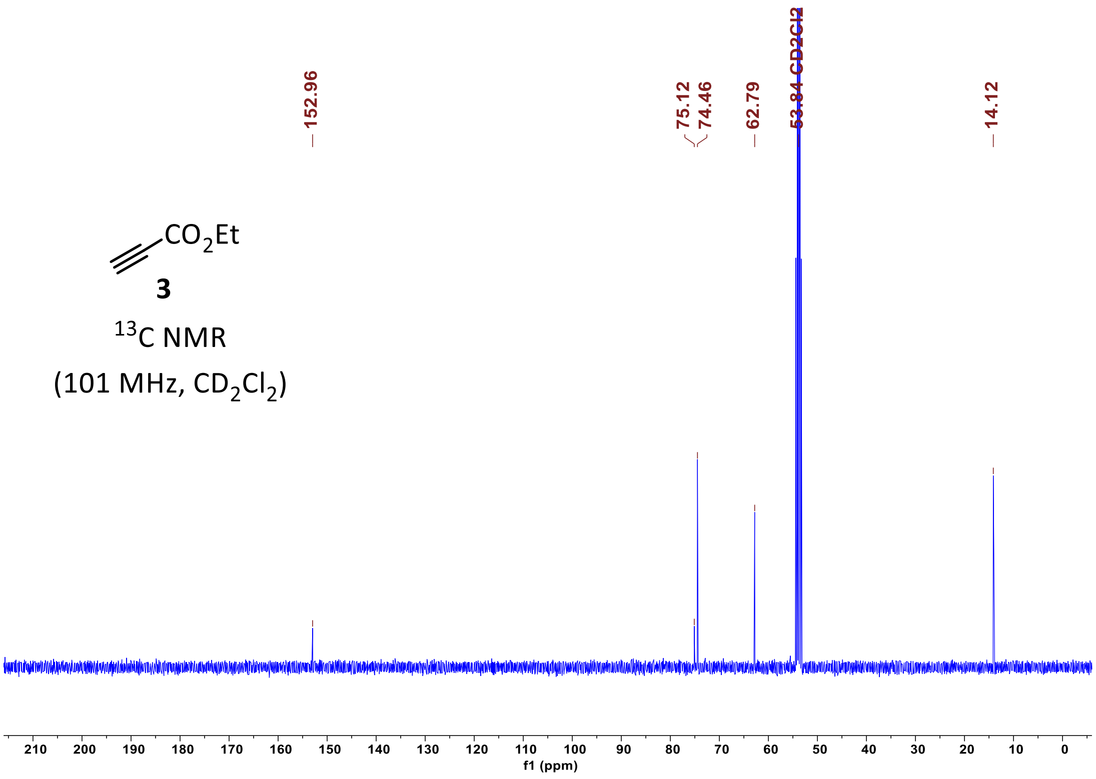

2.3.4 Ethenesulfonylfluoride (ESF, 4)

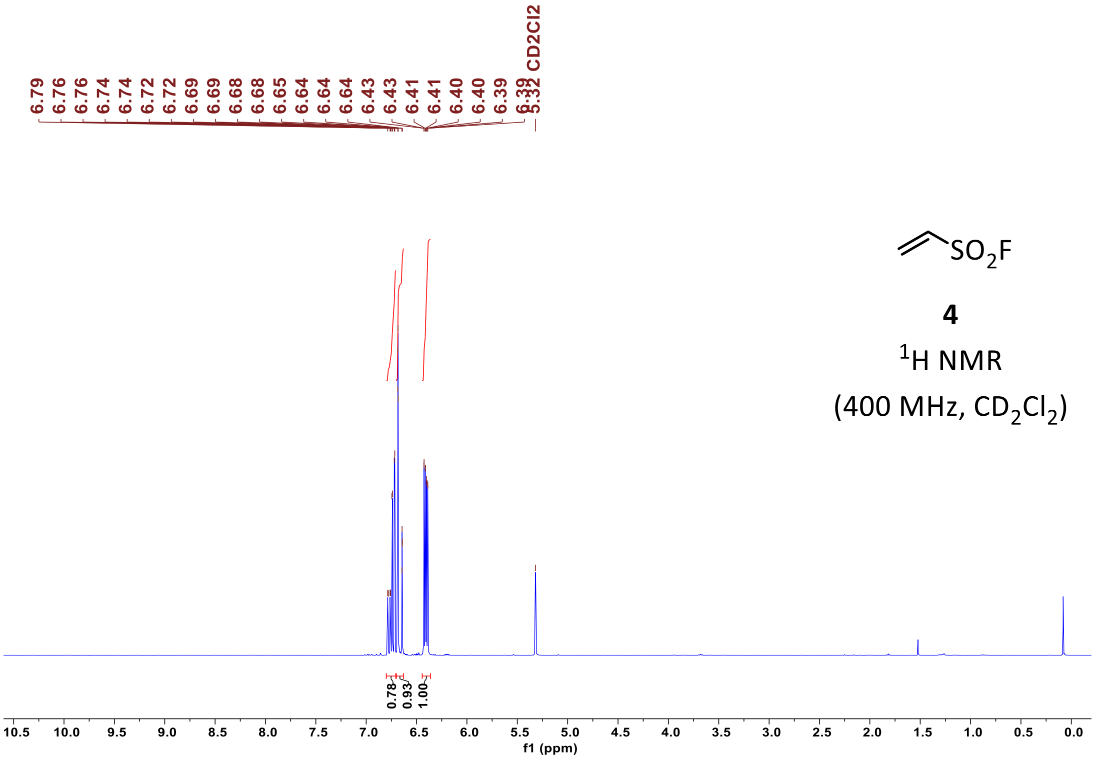

f606

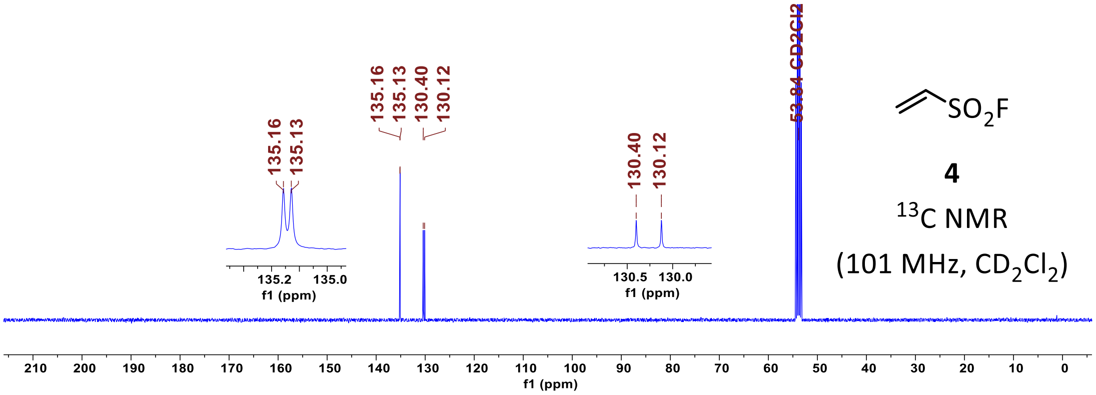

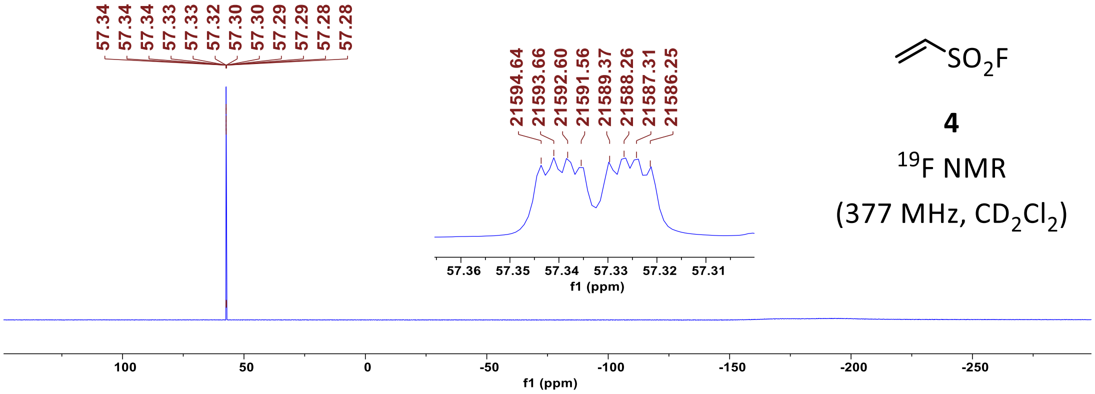

2.3.5 Ethyl 2-butynoate (5)

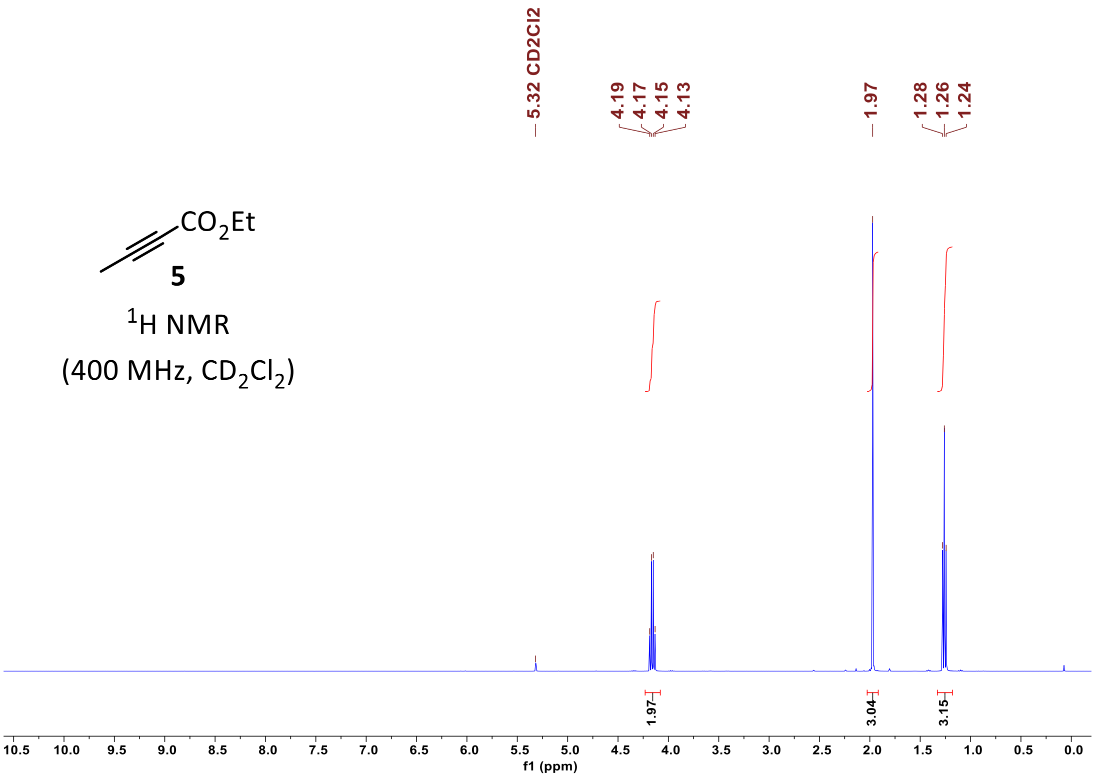

f444-2

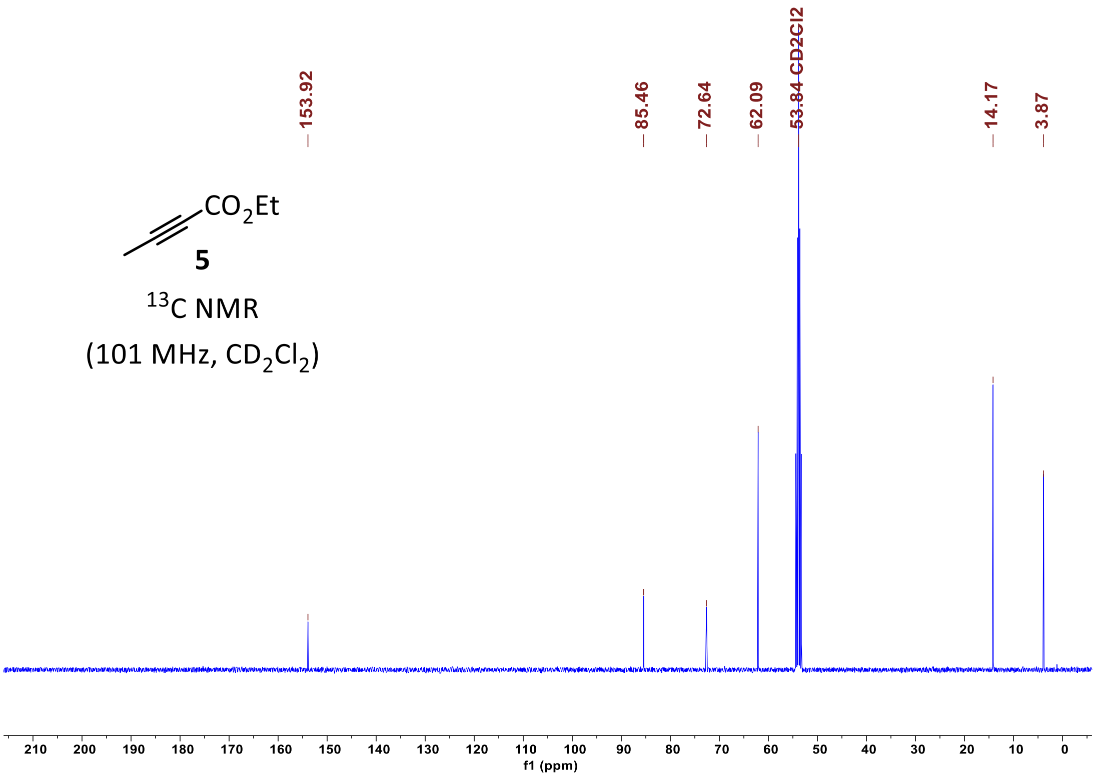

2.3.6 Tris(4-fluorophenyl)phosphine P(pfp)$_3$

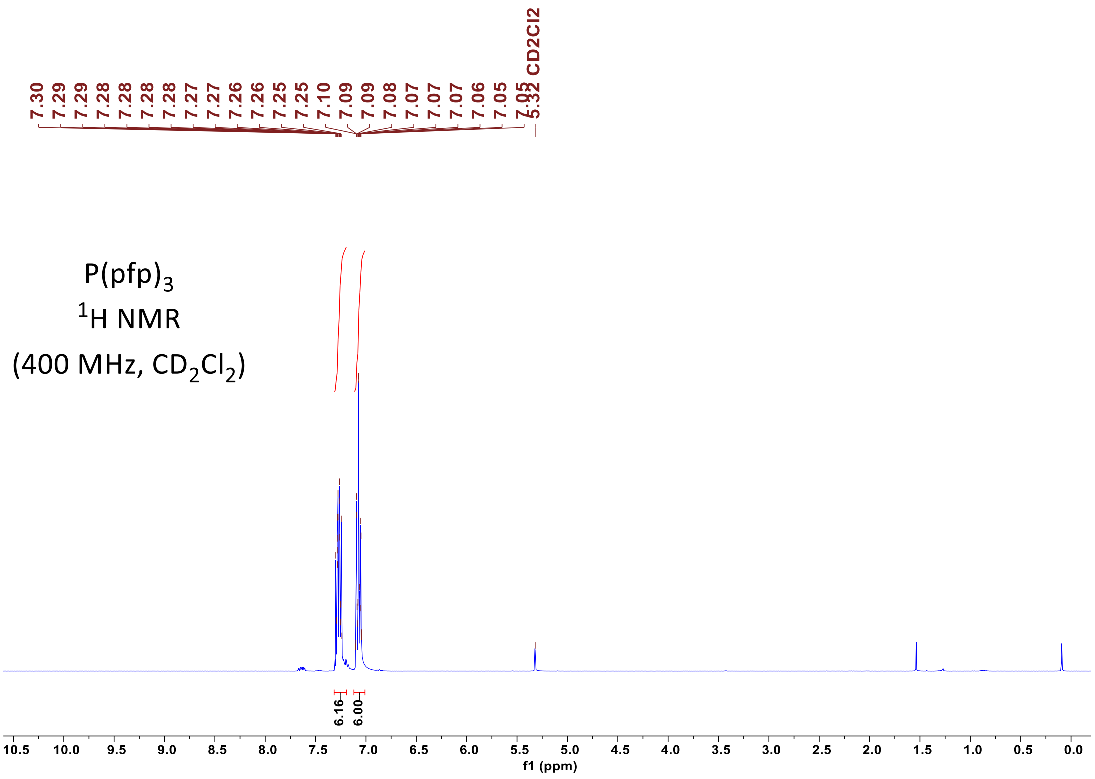

f409

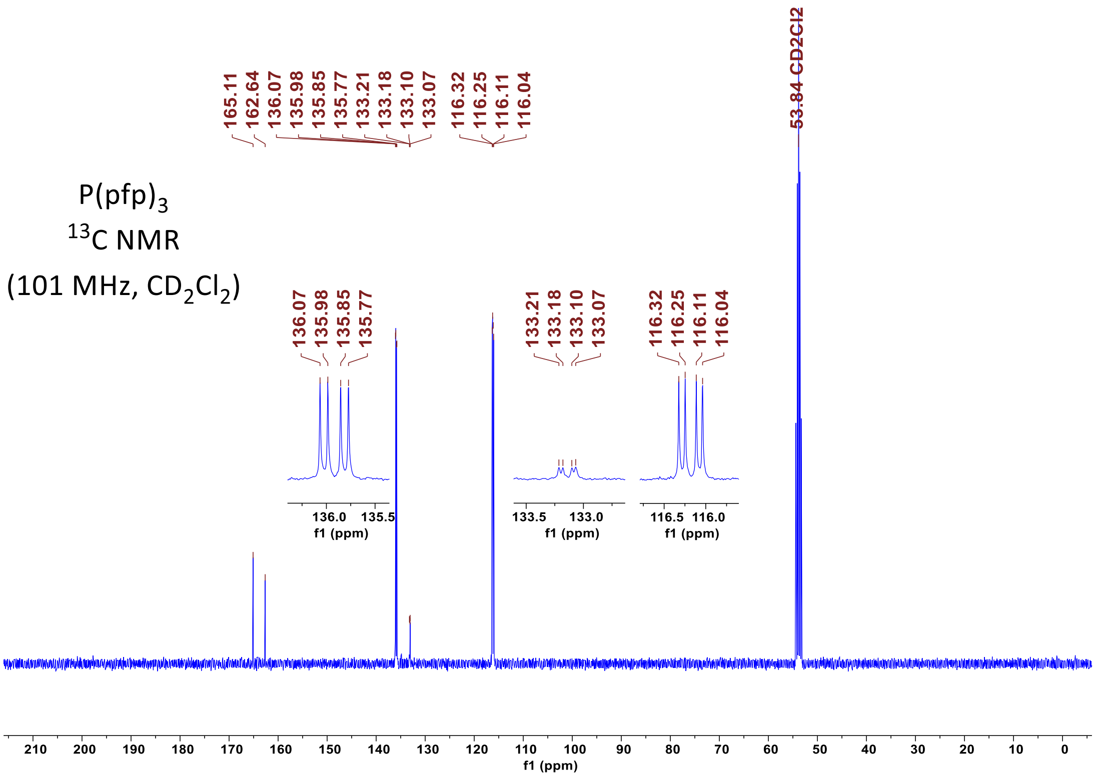

P(pfp)$_3$
^{31}P NMR
(162 MHz, CD_2Cl_2)

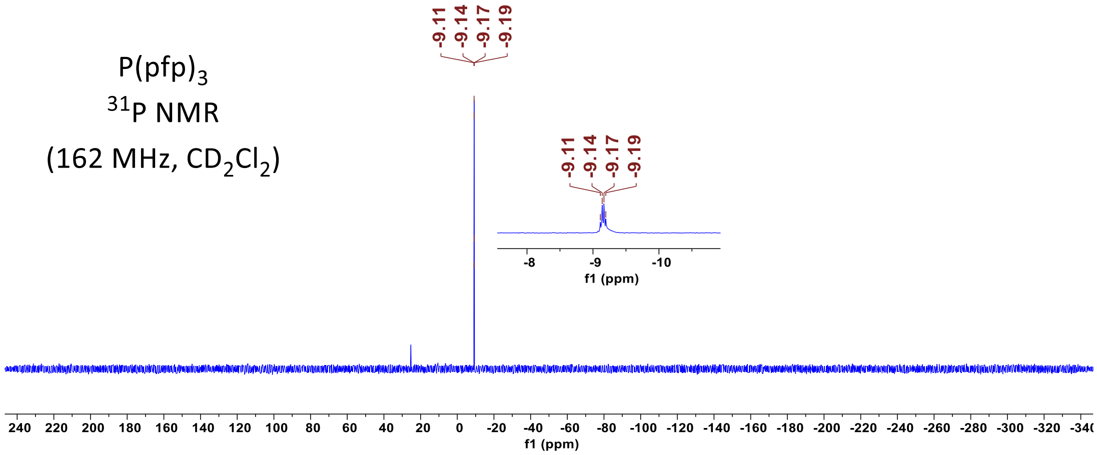

P(pfp)$_3$
^{19}F NMR
(377 MHz, CD_2Cl_2)

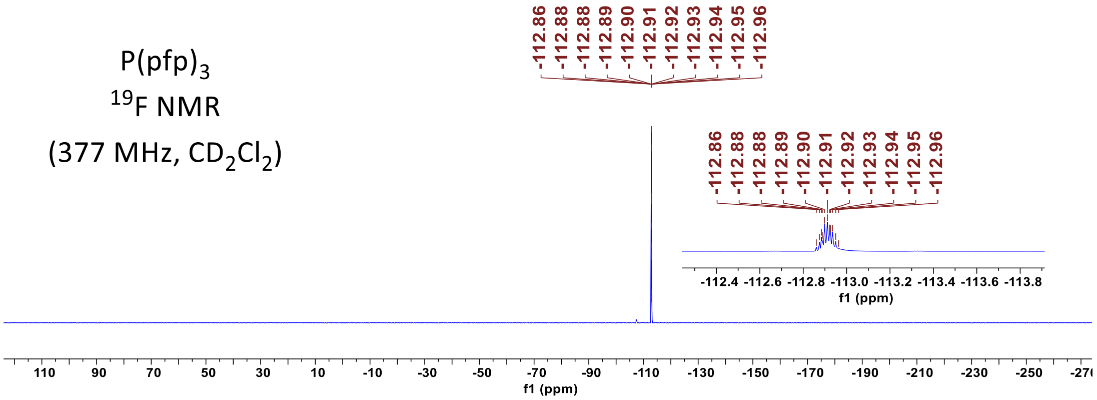

2.3.7 Triphenylphosphine PPh_3

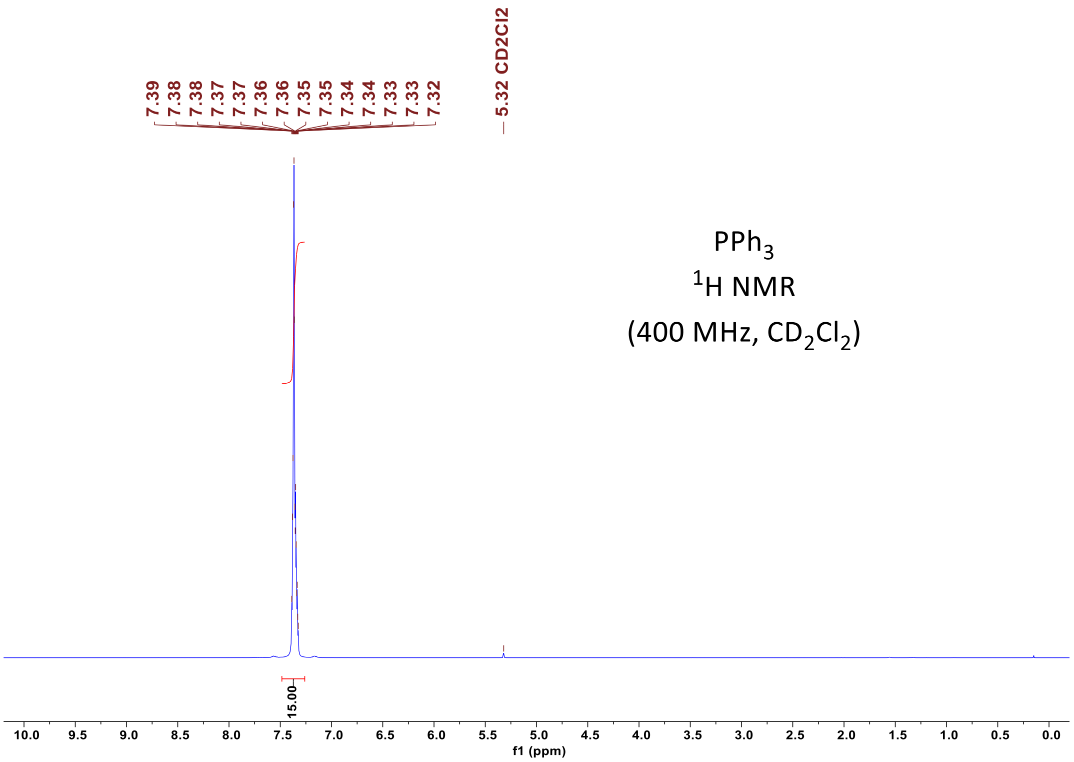

f363

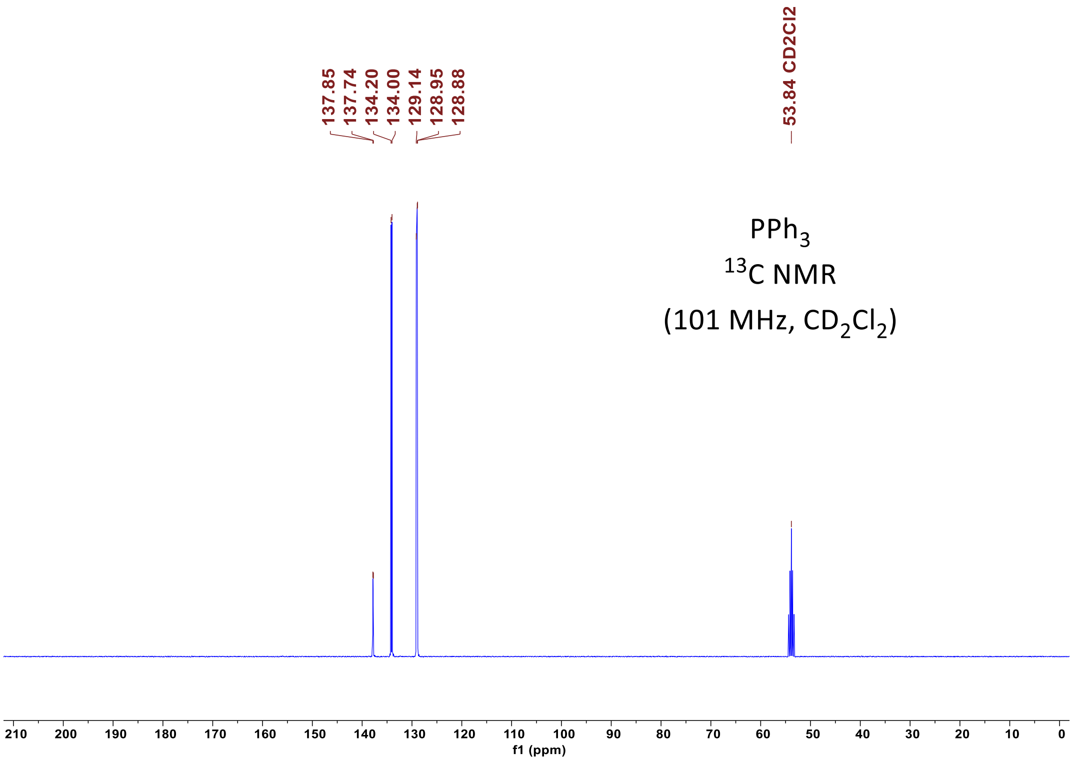

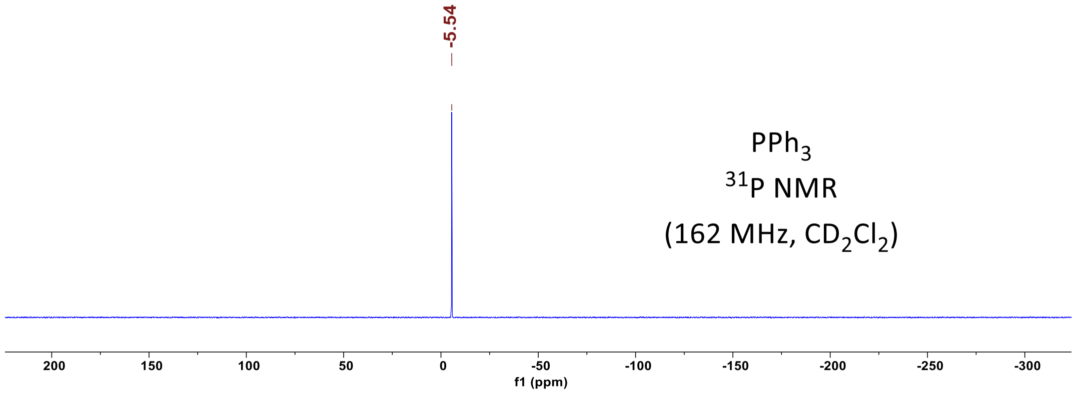

2.3.8 Triphenylphosphonium triflate (TPPT)

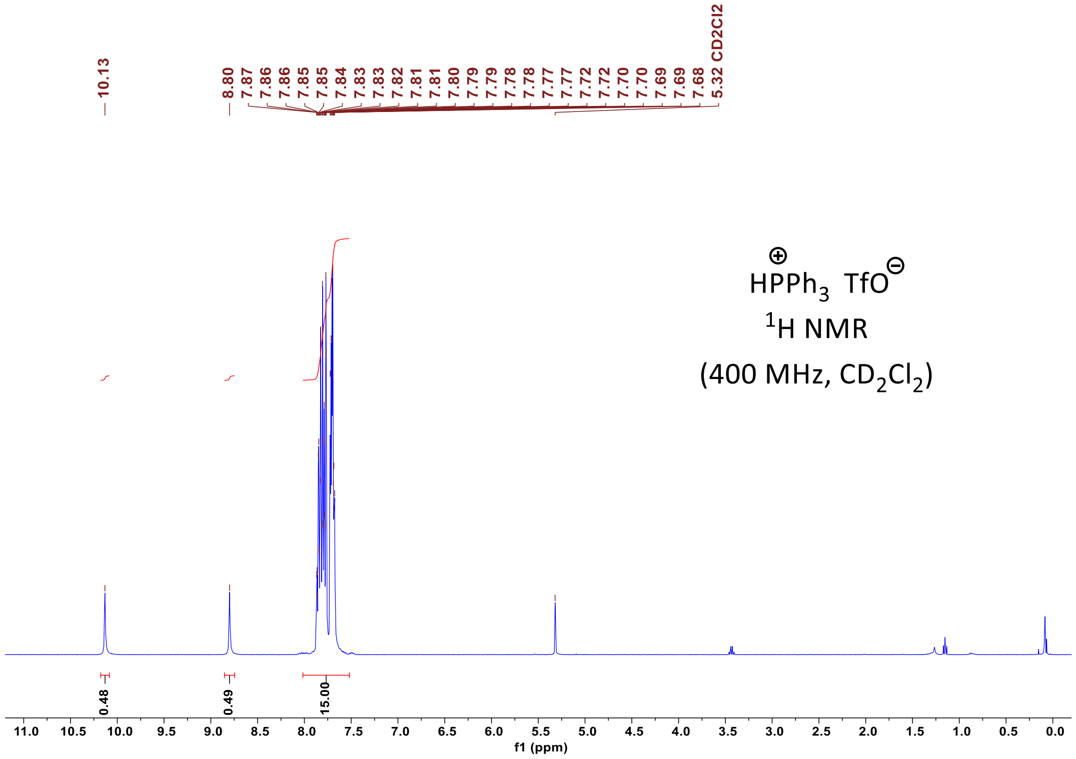

f372

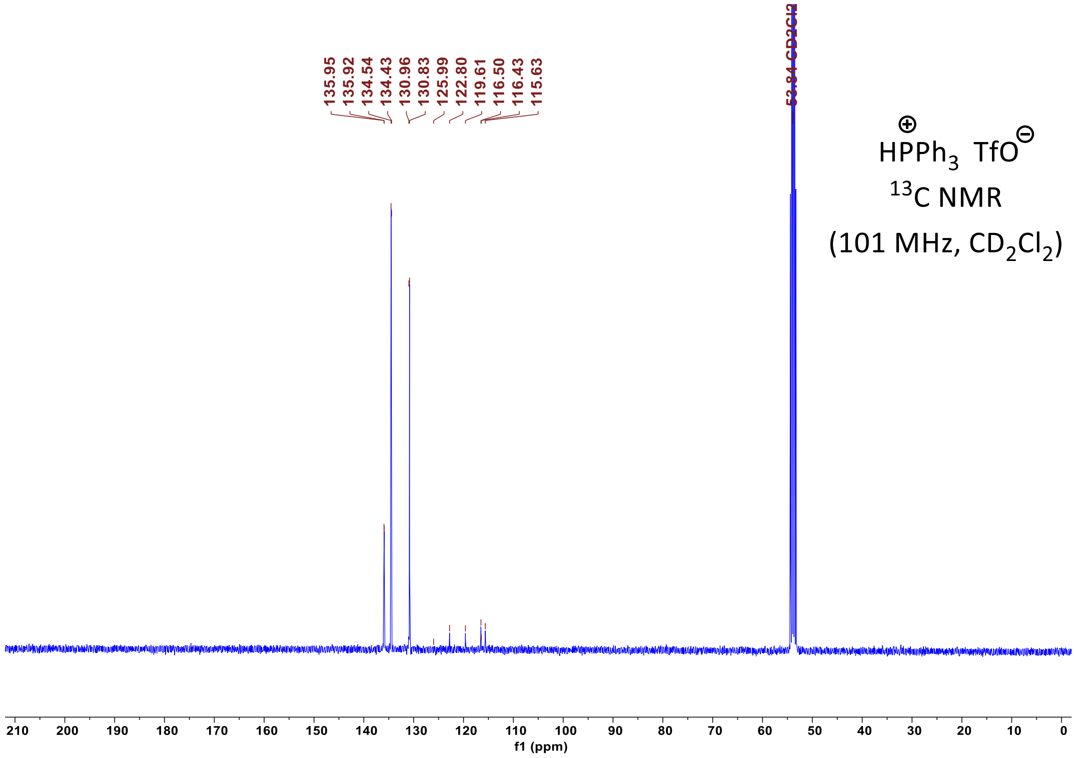
135.95
135.92
134.54
134.43
130.96
130.83
125.99
122.80
119.61
116.50
116.43
115.63
53.84 CD2Cl2
⊕
HPPh$_3$ TfO⊖
^{13}C NMR
(101 MHz, CD$_2$Cl$_2$)
f1 (ppm)

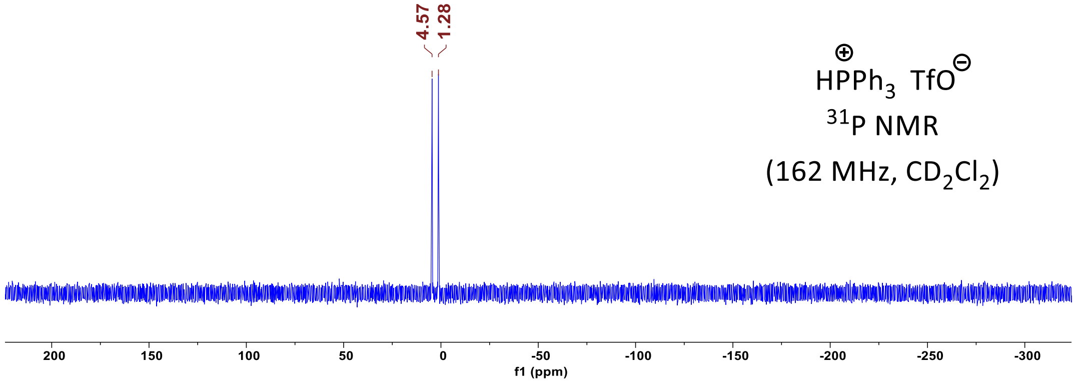
4.57
1.28
⊕
HPPh$_3$ TfO⊖
^{31}P NMR
(162 MHz, CD$_2$Cl$_2$)
f1 (ppm)

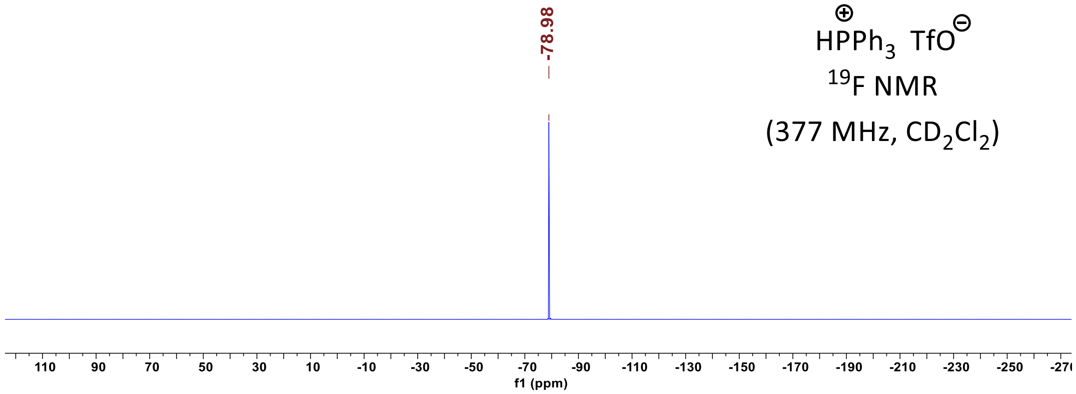
-78.98
⊕
HPPh$_3$ TfO⊖
^{19}F NMR
(377 MHz, CD$_2$Cl$_2$)
f1 (ppm)

2.3.9 Tris(4-methoxyphenyl)phosphine P(ani)$_3$

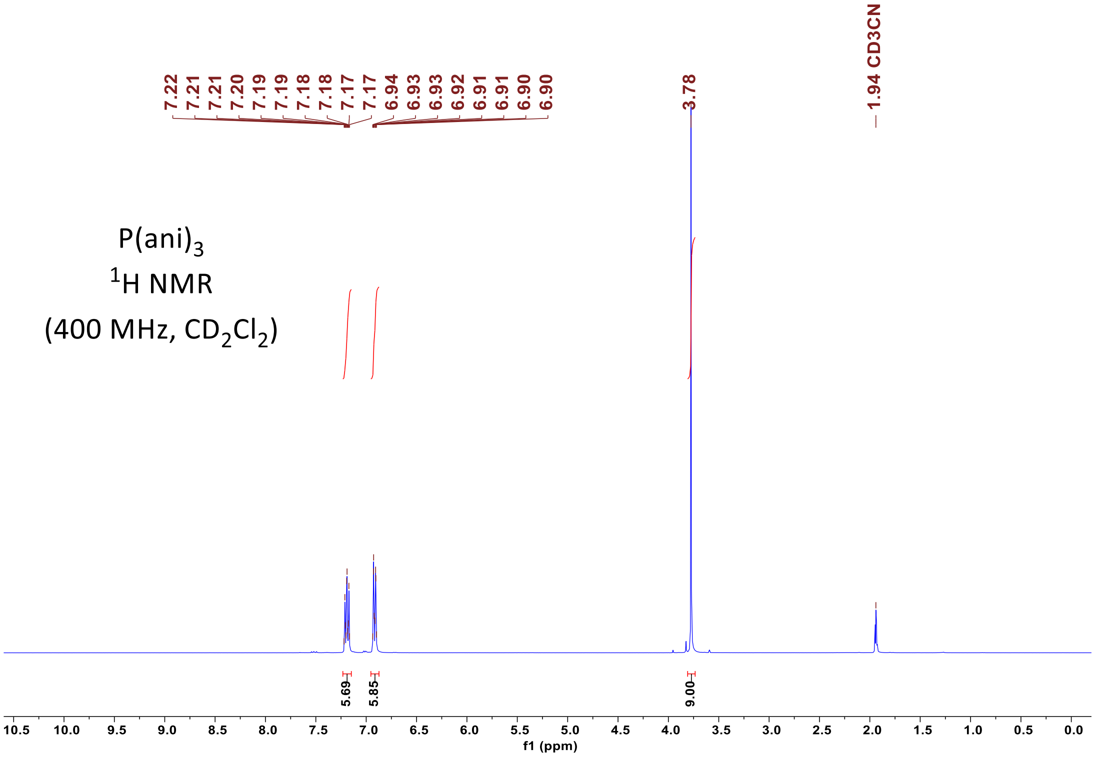

f419

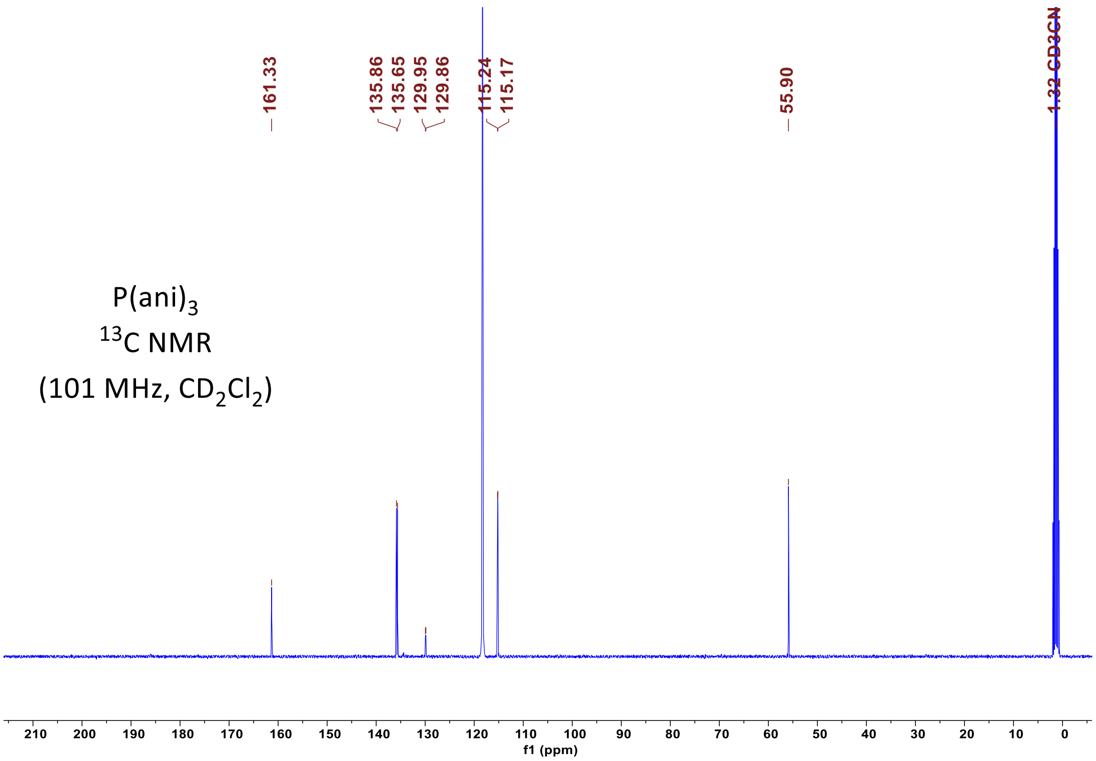

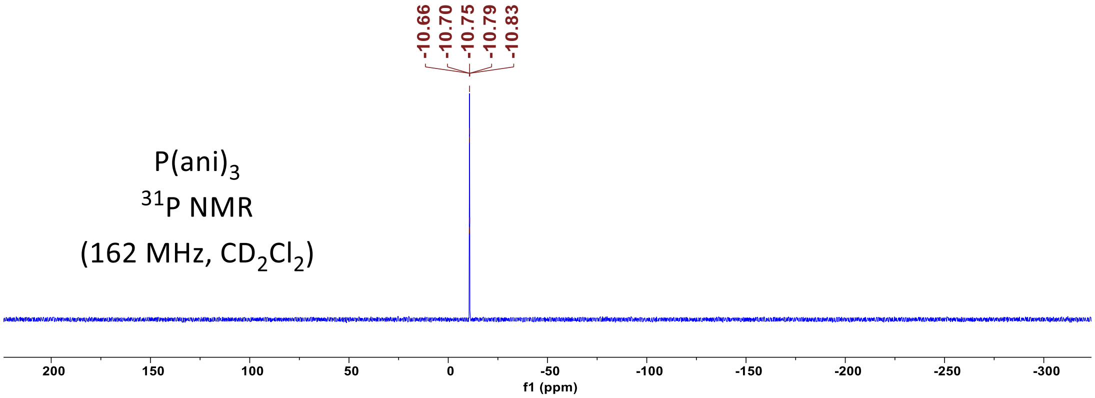

2.3.10 Methyldiphenylphosphine $PMePh_2$

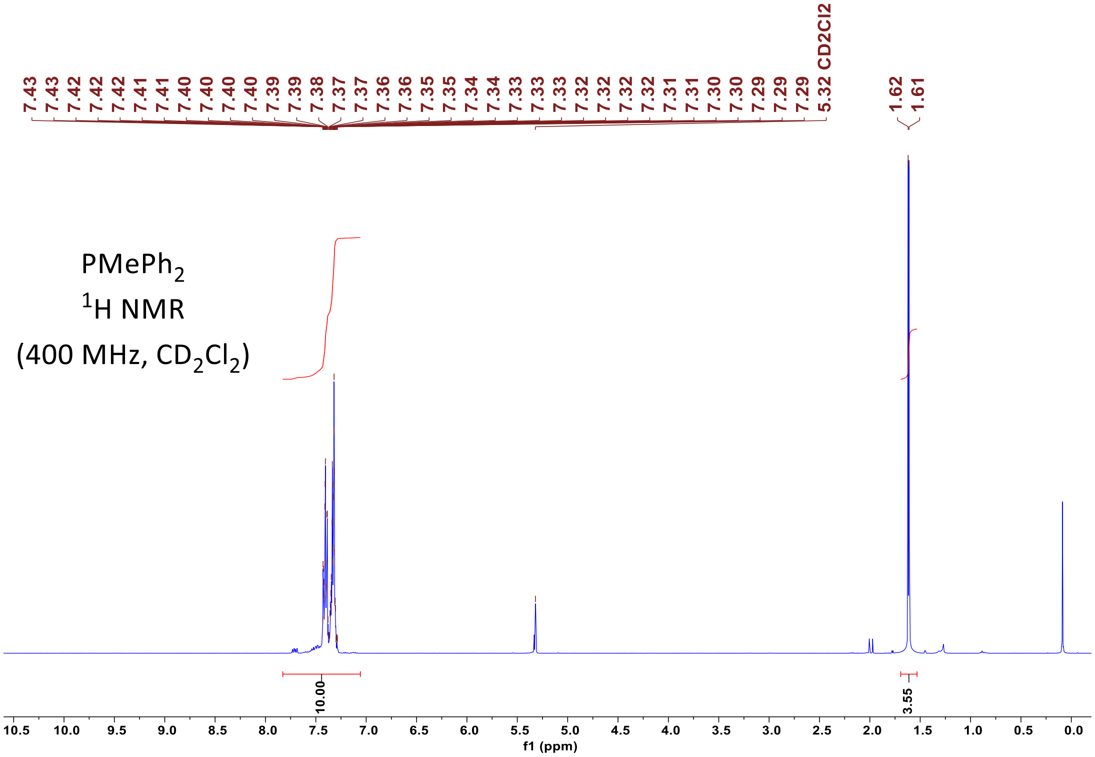

f390

PMePh$_2$
^{13}C NMR
(101 MHz, CD_2Cl_2)

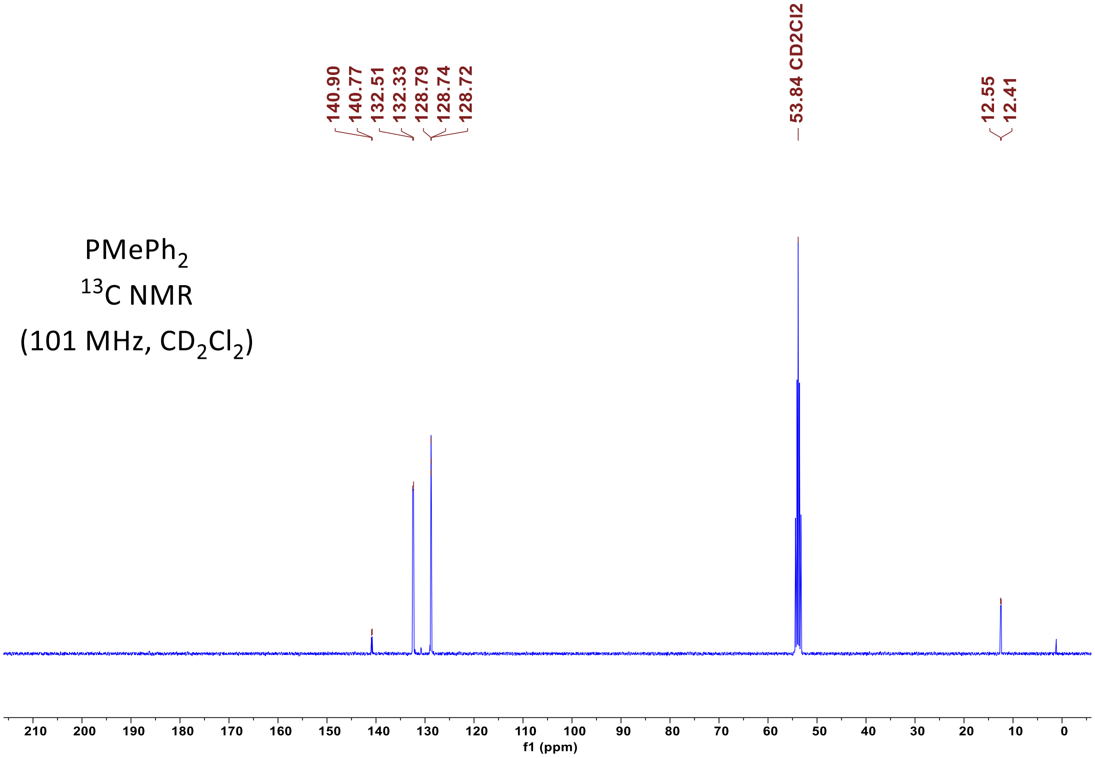

PMePh$_2$
^{31}P{^{1}H} NMR
(162 MHz, CD_2Cl_2)

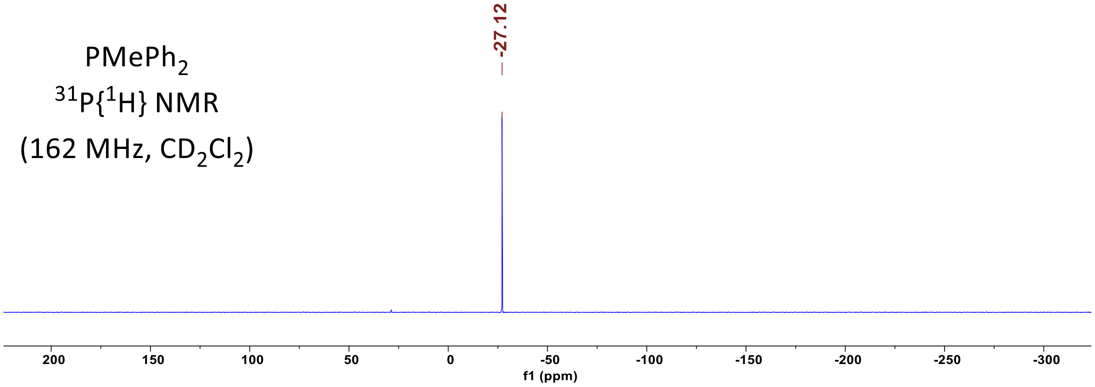

2.3.11 Methyldiphenylphosphonium triflate

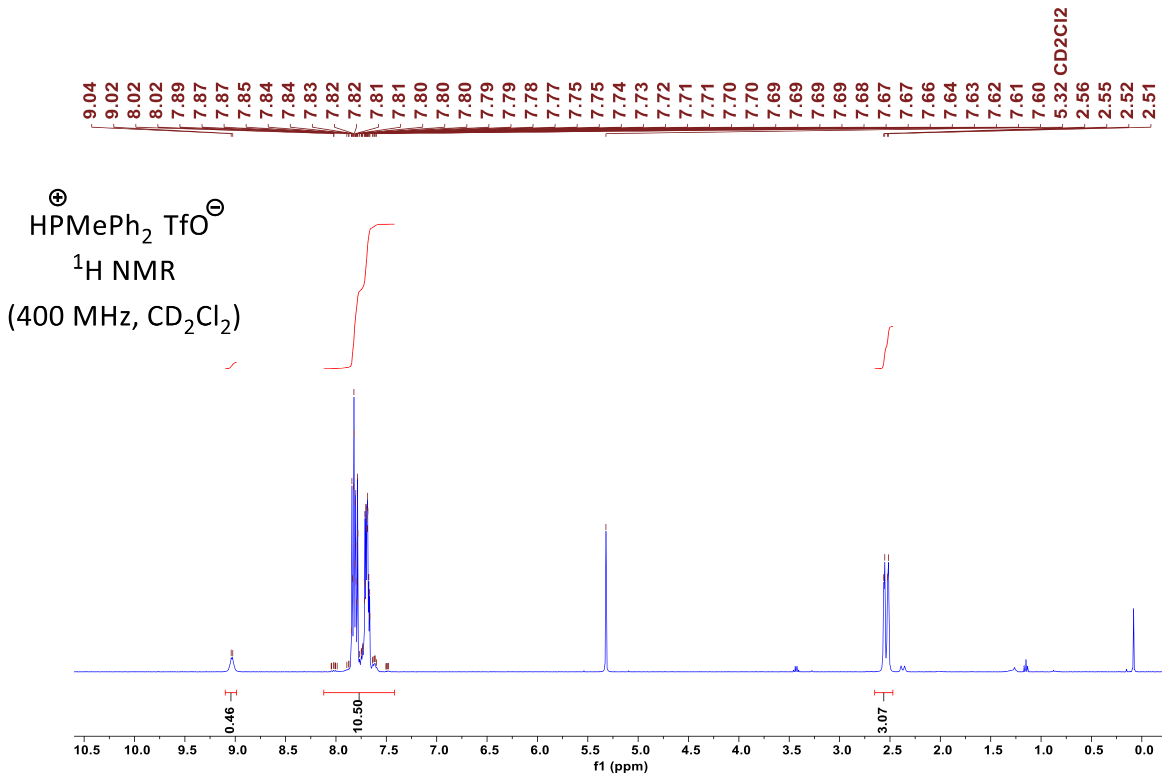

f518

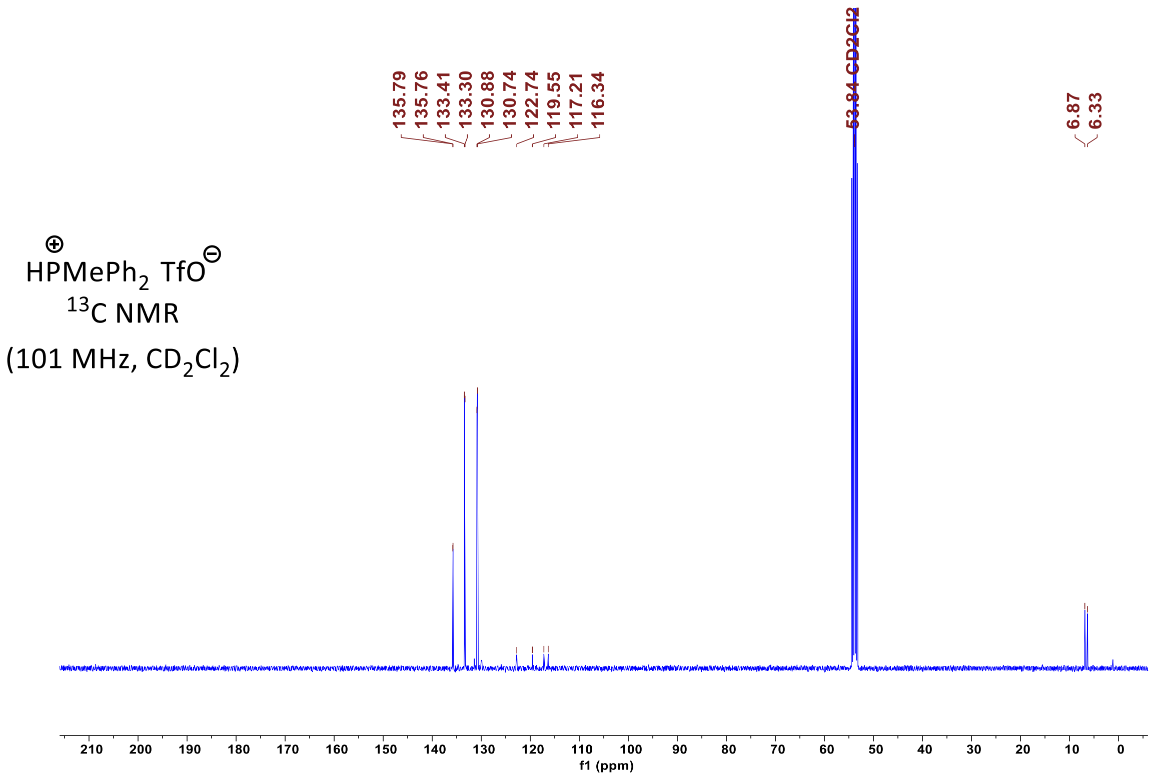

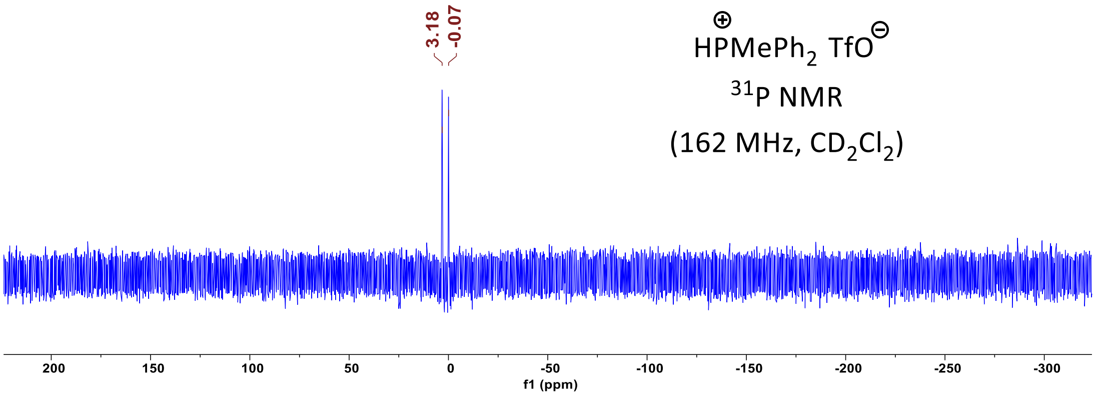
3.18
-0.07
HPMePh2 TfO
31P NMR
(162 MHz, CD2Cl2)
200
150
100
50
0
-50
-100
-150
-200
-250
-300
f1 (ppm)

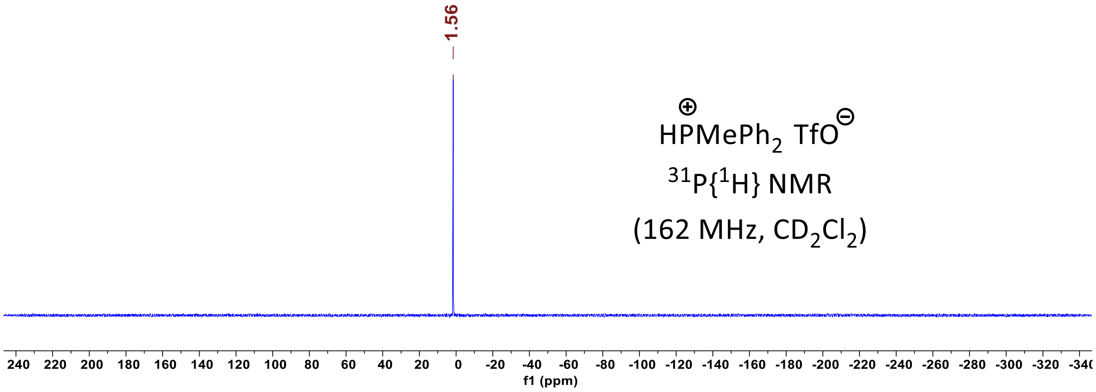
1.56
HPMePh2 TfO
31P{1H} NMR
(162 MHz, CD2Cl2)
240
220
200
180
160
140
120
100
80
60
40
20
0
-20
-40
-60
-80
-100
-120
-140
-160
-180
-200
-220
-240
-260
-280
-300
-320
f1 (ppm)

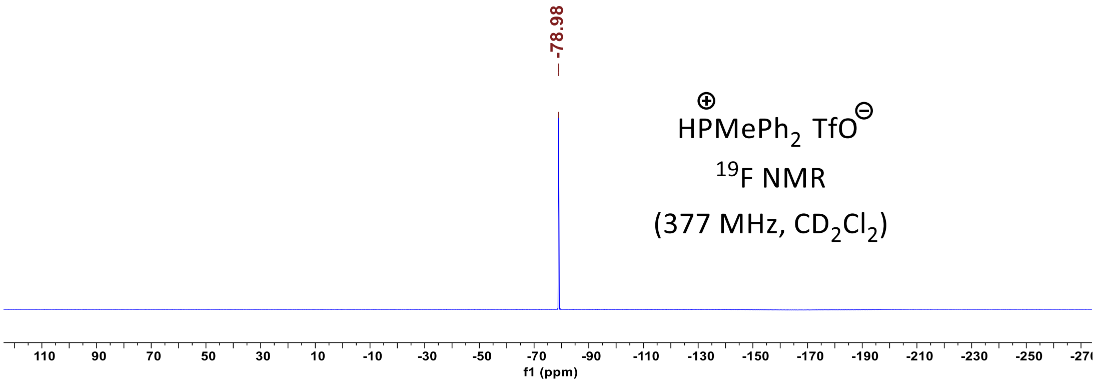
-78.98
HPMePh2 TfO
19F NMR
(377 MHz, CD2Cl2)
110
90
70
50
30
10
-10
-30
-50
-70
-90
-110
-130
-150
-170
-190
-210
-230
-250
f1 (ppm)

2.3.12 Dimethyl(phenyl)phosphine

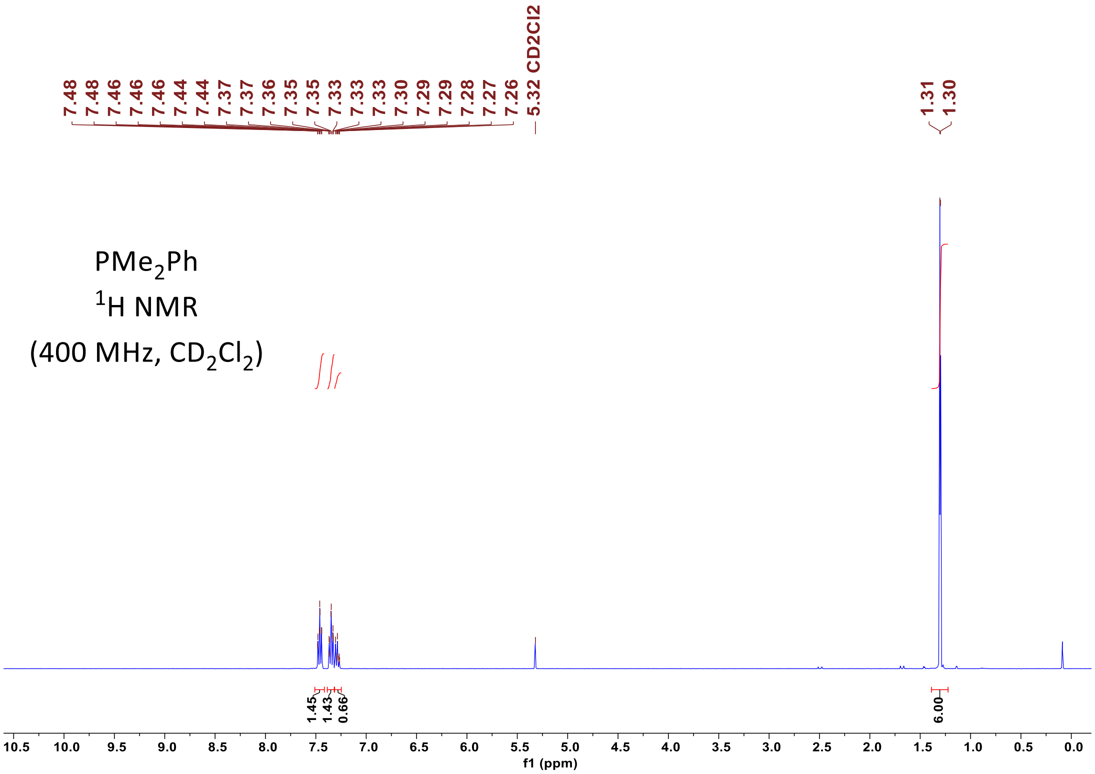

f393

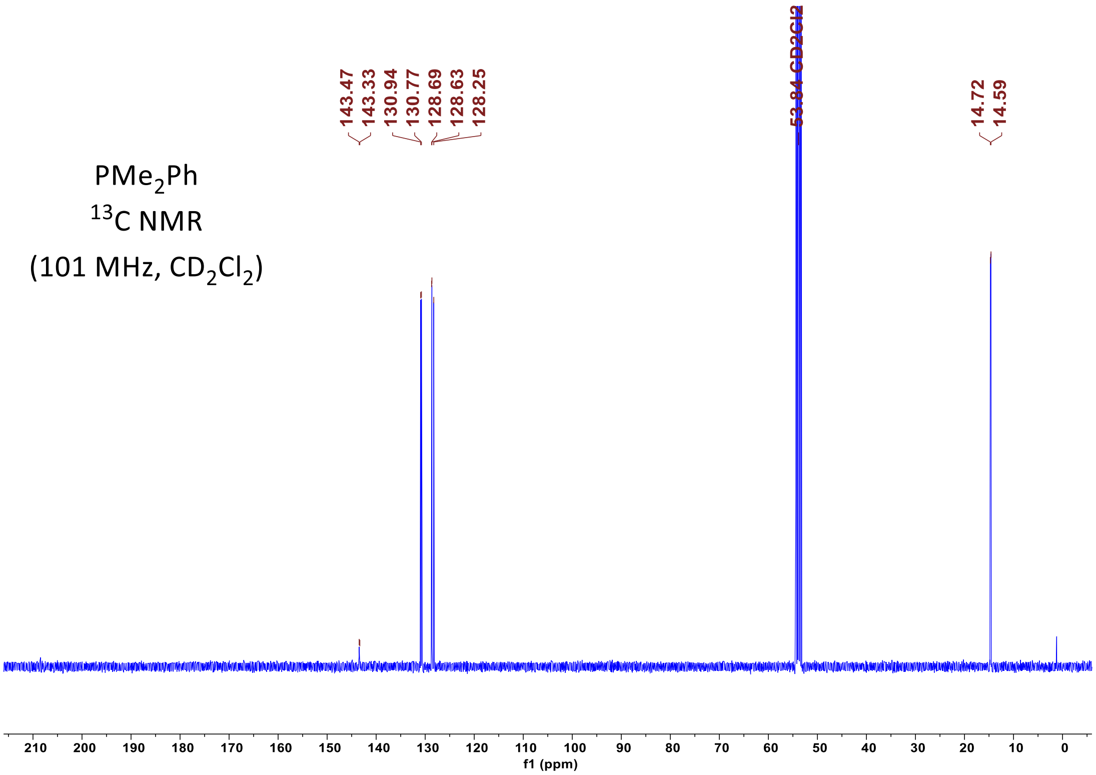

PMe$_2$Ph
$^{31}P\{^1H\}$ NMR
(162 MHz, CD_2Cl_2)

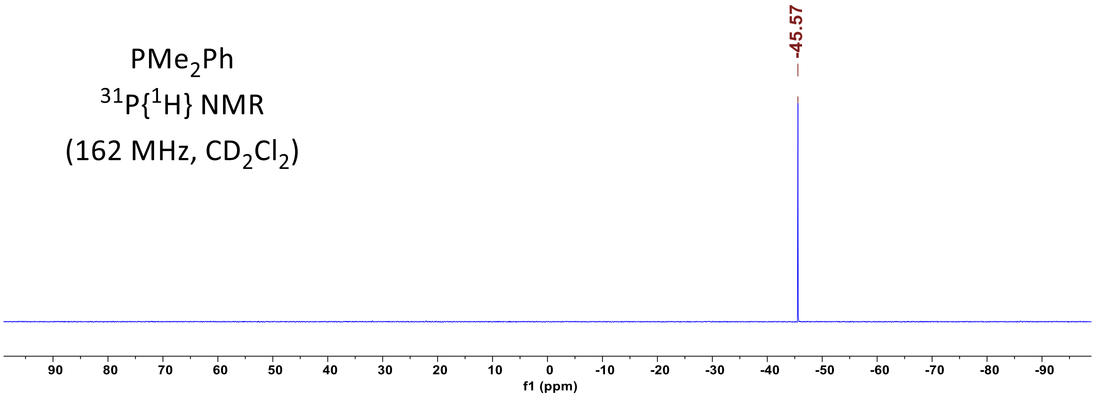

2.3.13 Dimethyl(phenyl)phosphonium triflate

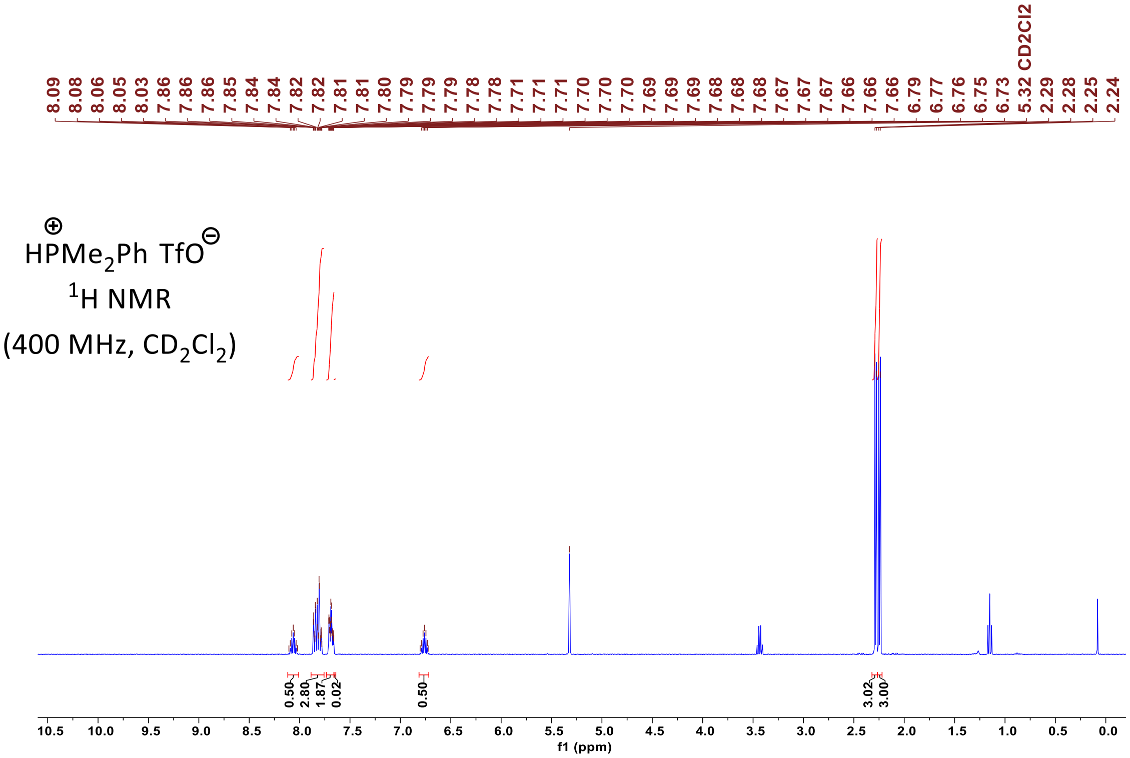

⊕
HPMe$_2$Ph TfO⊖
^{1}H NMR
(400 MHz, CD_2Cl_2)

f519

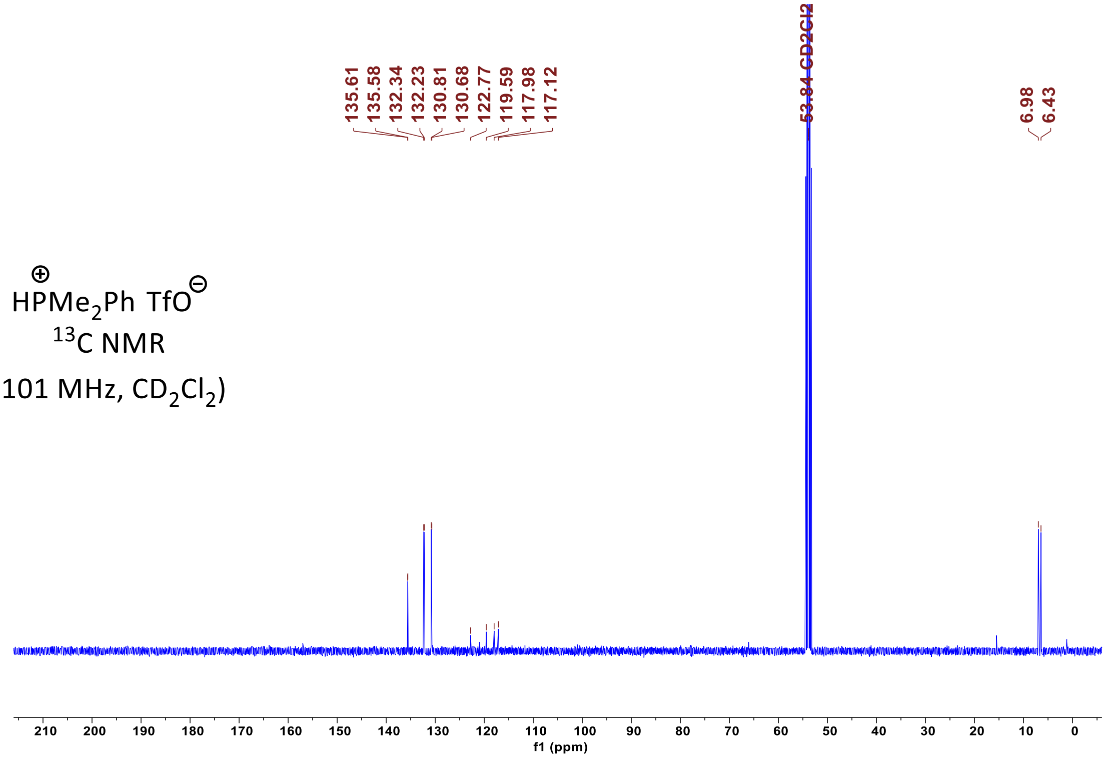

⊕
HPMe$_2$Ph TfO⊖
^{13}C NMR
(101 MHz, CD_2Cl_2)

⊕
HPMe$_2$Ph TfO⊖
^{31}P NMR
(162 MHz, CD_2Cl_2)

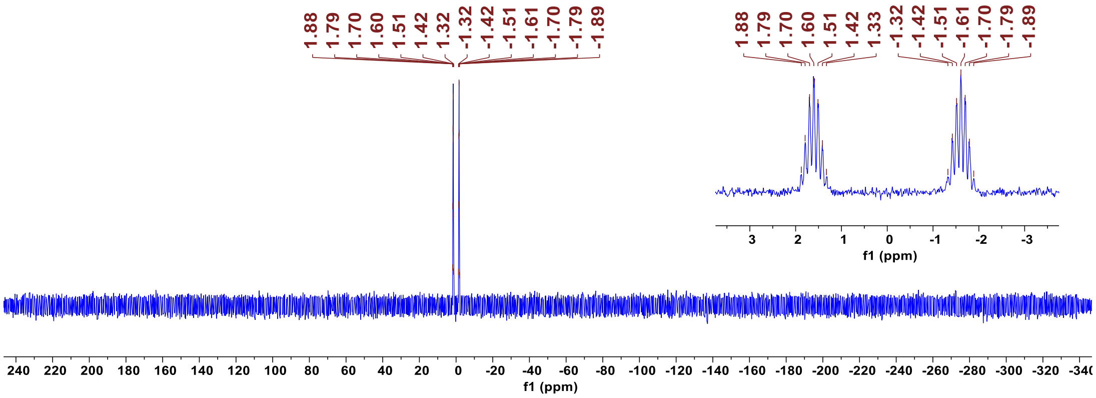

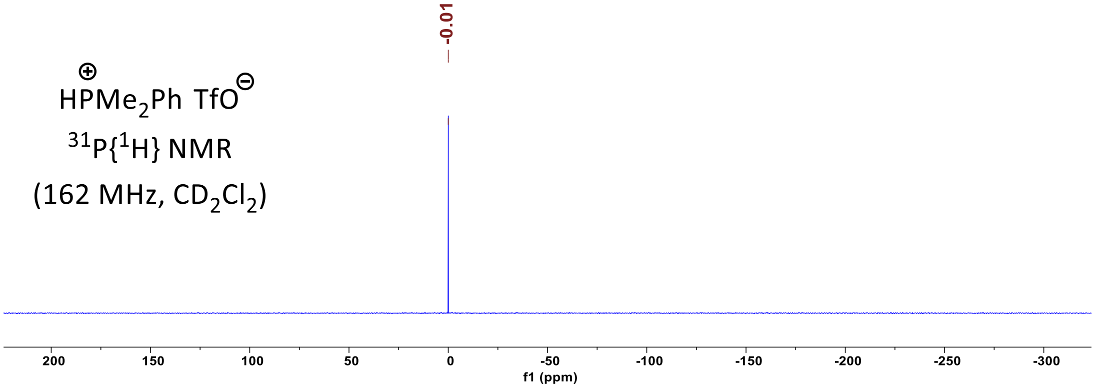

$\overset{\oplus}{H}PMe_2Ph\ TfO^{\ominus}$

$^{31}P\{^{1}H\}$ NMR

(162 MHz, CD_2Cl_2)

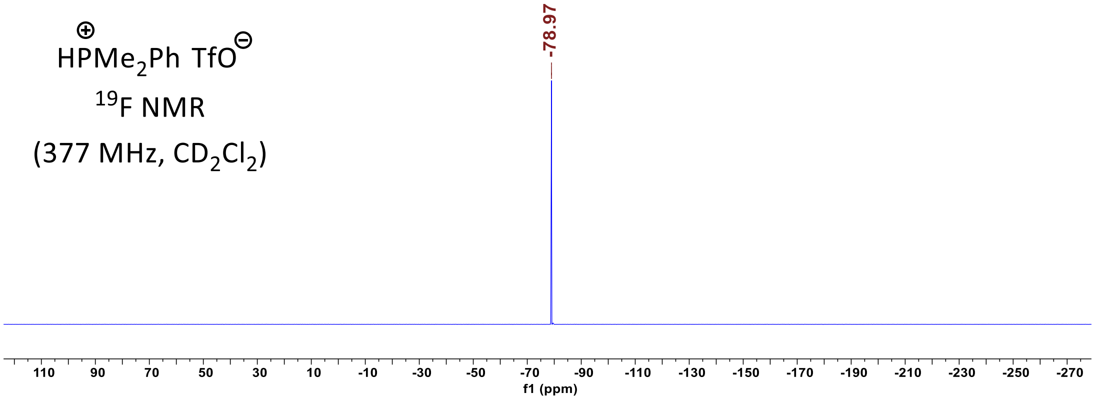

$\overset{\oplus}{H}PMe_2Ph\ TfO^{\ominus}$

^{19}F NMR

(377 MHz, CD_2Cl_2)

2.3.14 Tributylphosphine

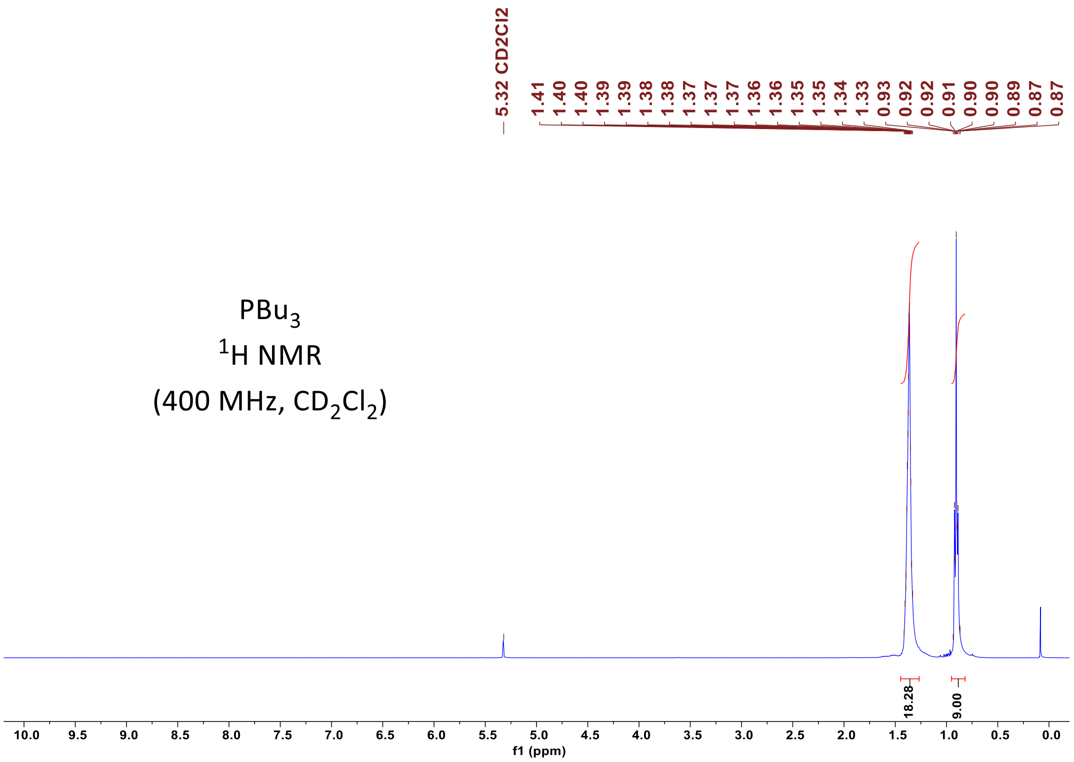

f416

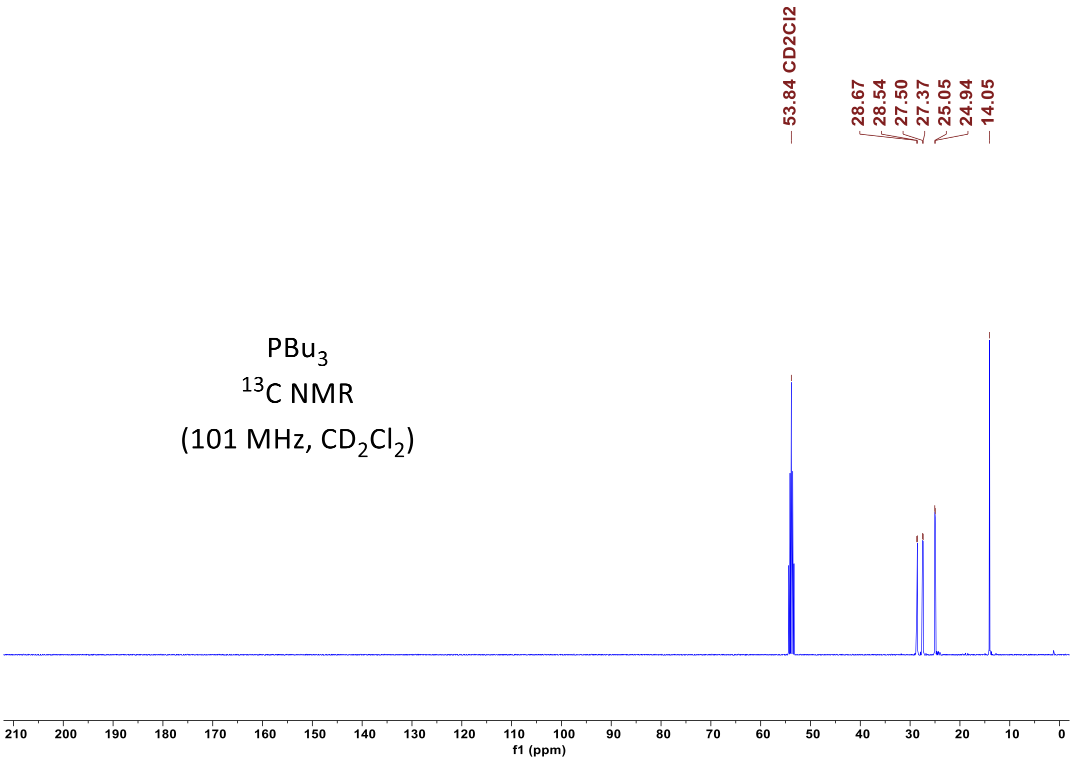

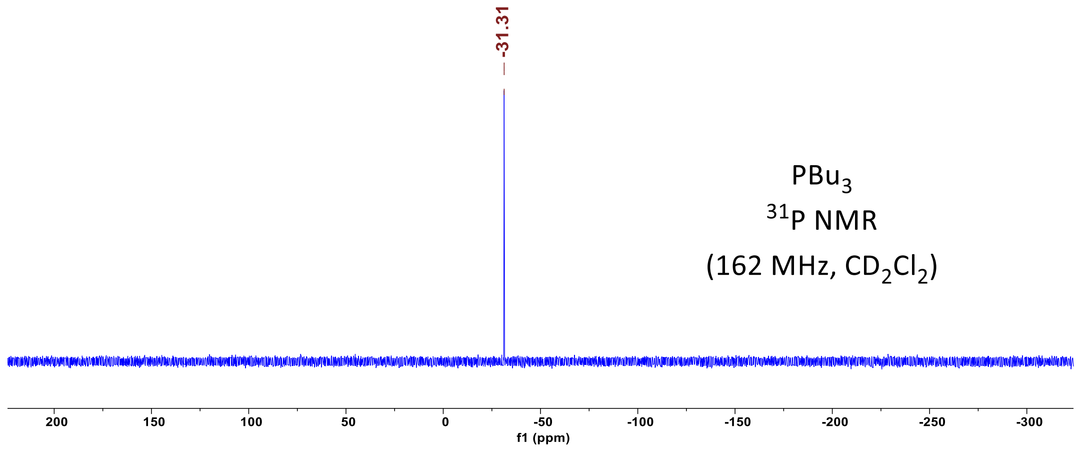

2.3.15 Tributylphosphonium triflate (TBPT)

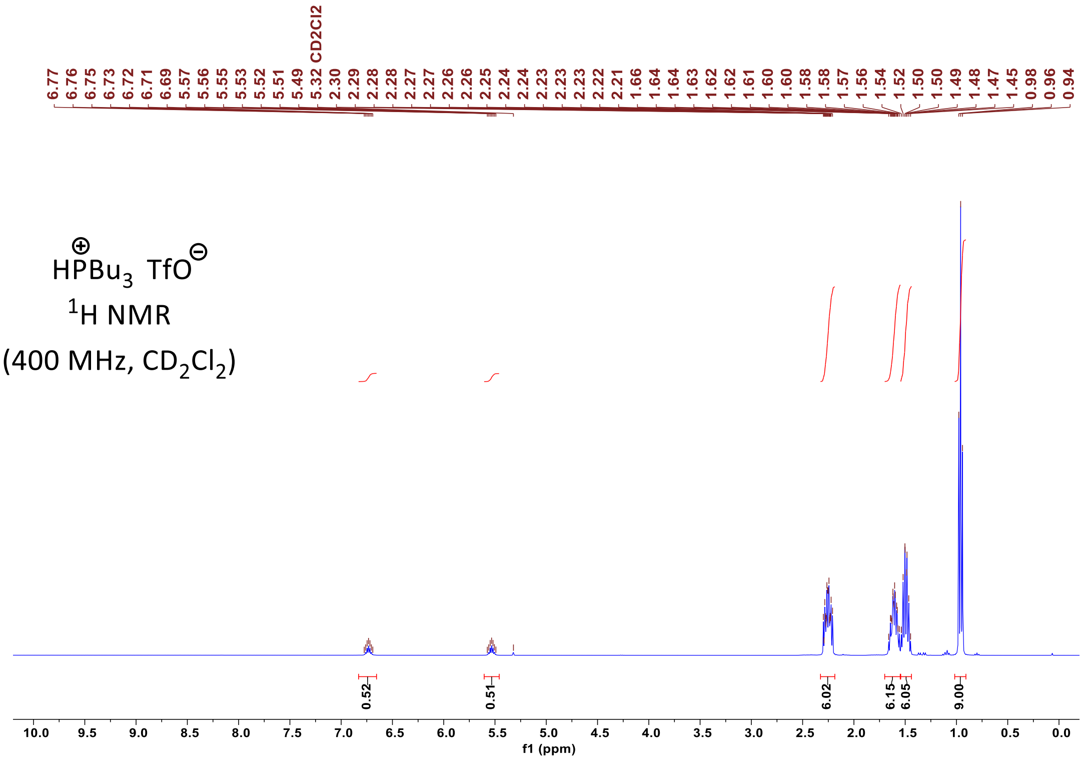

f214

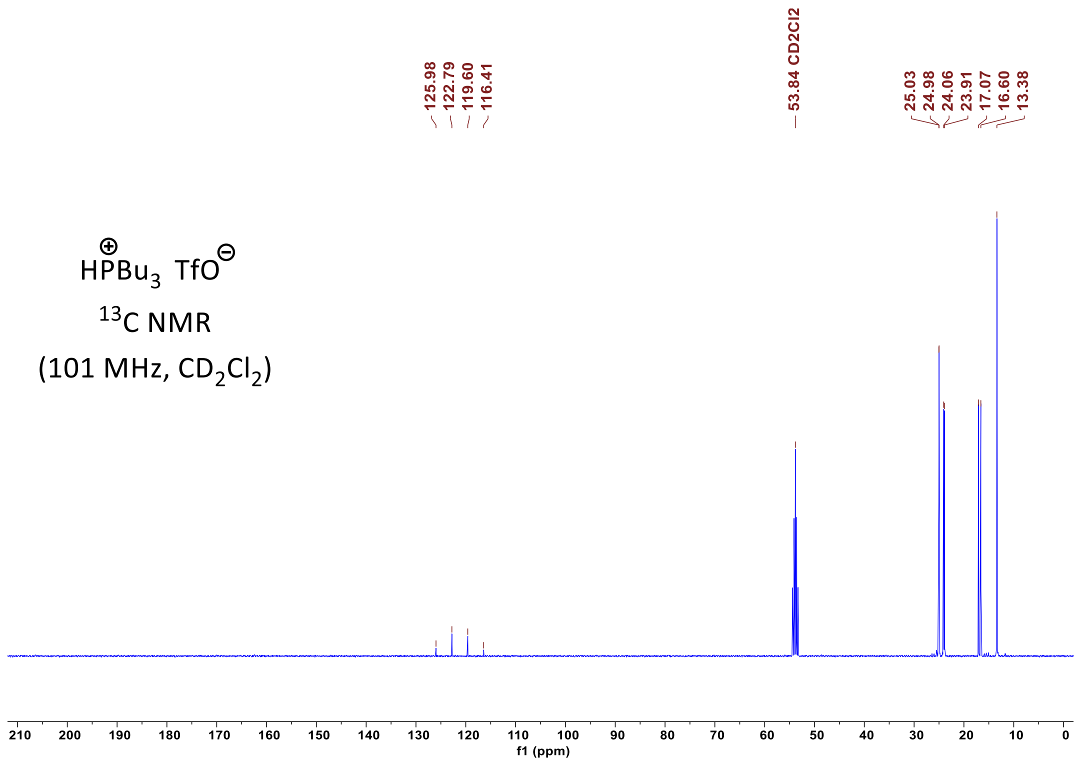

125.98
122.79
119.60
116.41
53.84 CD2Cl2
25.03
24.98
24.06
23.91
17.07
16.60
13.38
HPBu3 TfO
13C NMR
(101 MHz, CD2Cl2)
210 200 190 180 170 160 150 140 130 120 110 100 90 80 70 60 50 40 30 20 10 0
f1 (ppm)

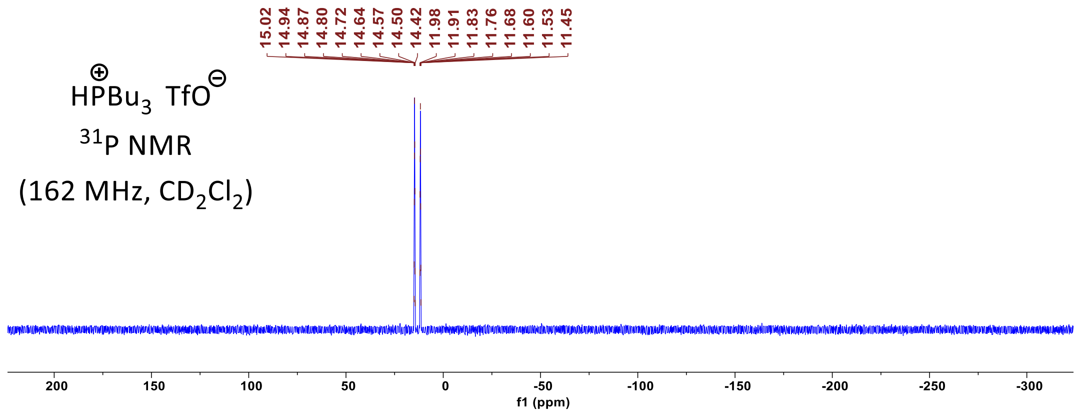

15.02
14.94
14.87
14.80
14.72
14.64
14.57
14.50
14.42
11.98
11.91
11.83
11.76
11.68
11.60
11.53
11.45
HPBu3 TfO
31P NMR
(162 MHz, CD2Cl2)
200 150 100 50 0 -50 -100 -150 -200 -250 -300
f1 (ppm)

2.3.16 Trimethylphosphonium tetrafluoroborate

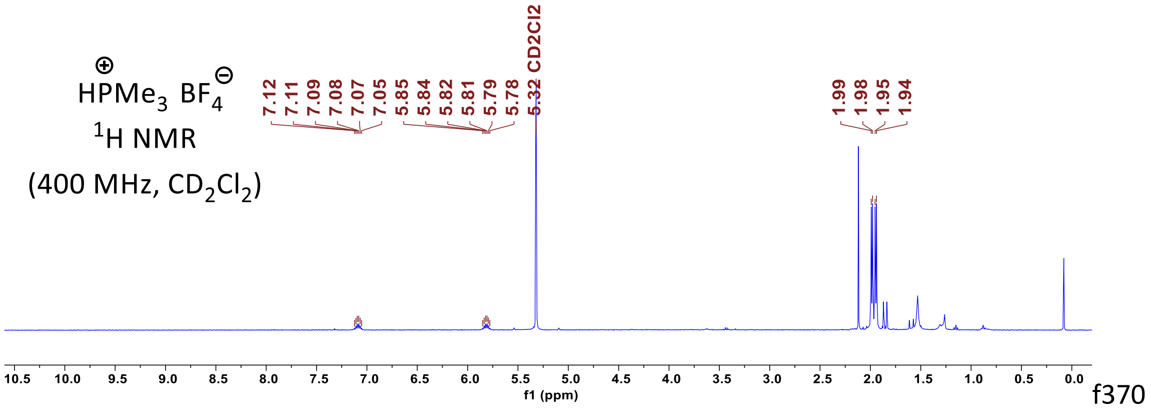

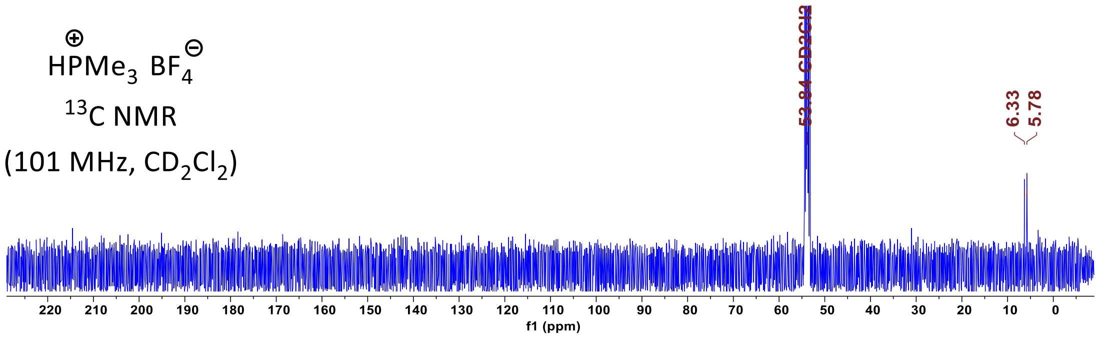

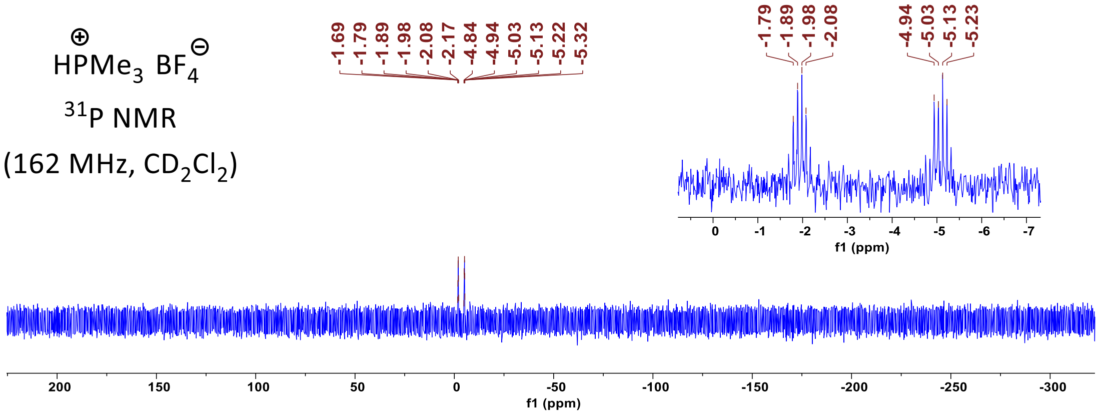

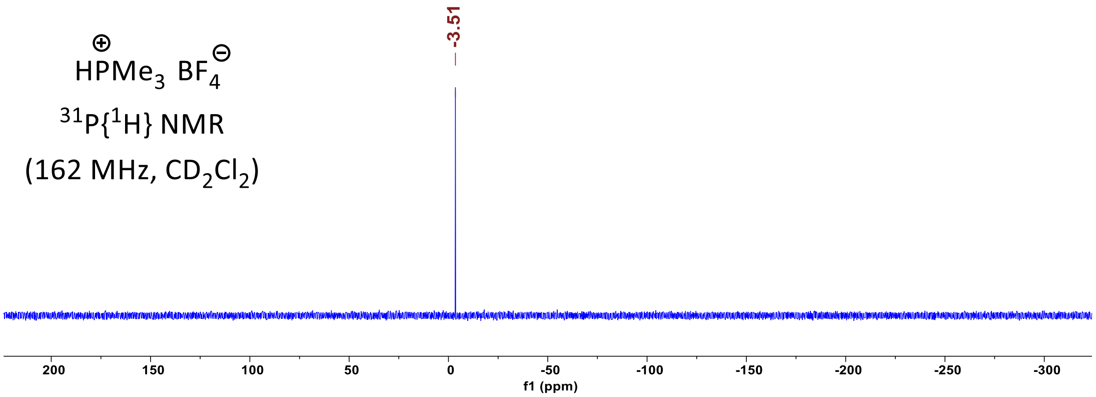

2.3.17 Tricyclohexylphosphonium triflate

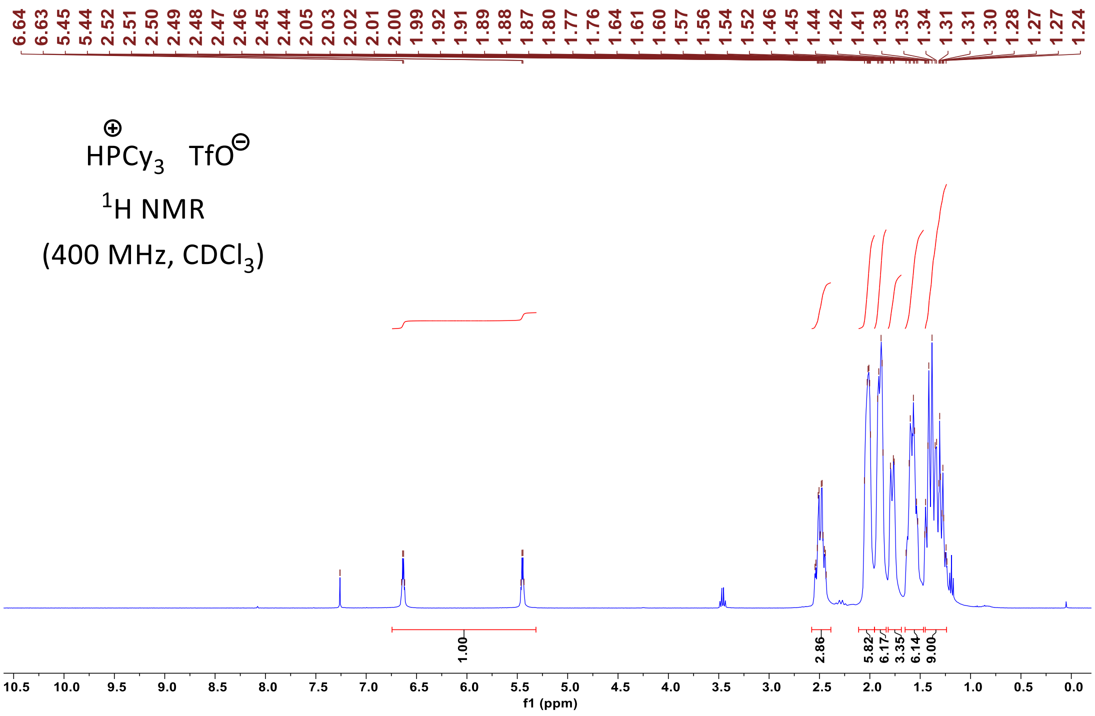

f414

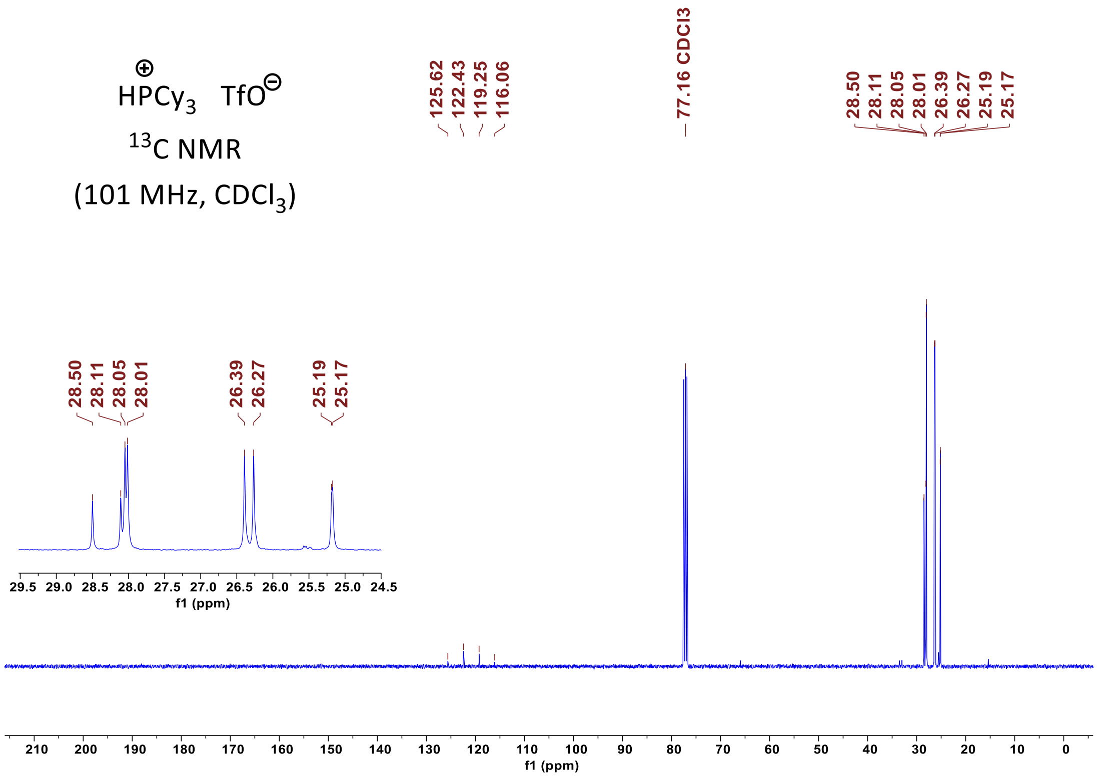

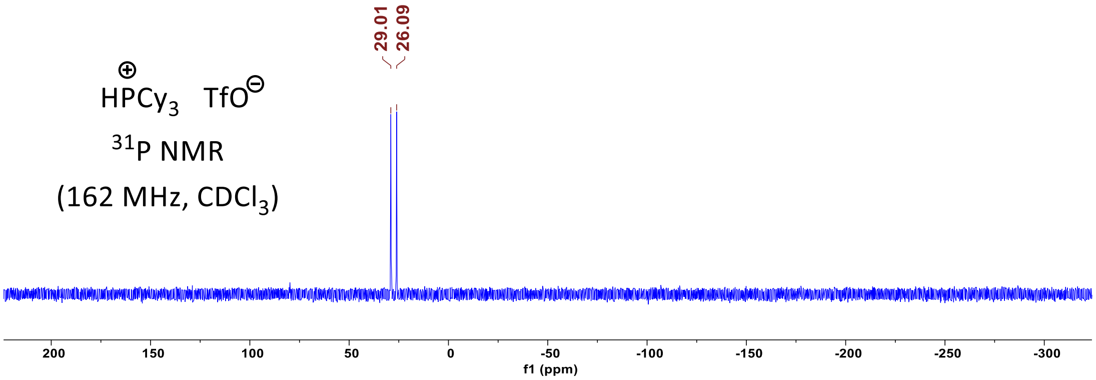
HPCy3 TfO
31P NMR
(162 MHz, CDCl3)
29.01
26.09
200 150 100 50 0 -50 -100 -150 -200 -250 -300
f1 (ppm)

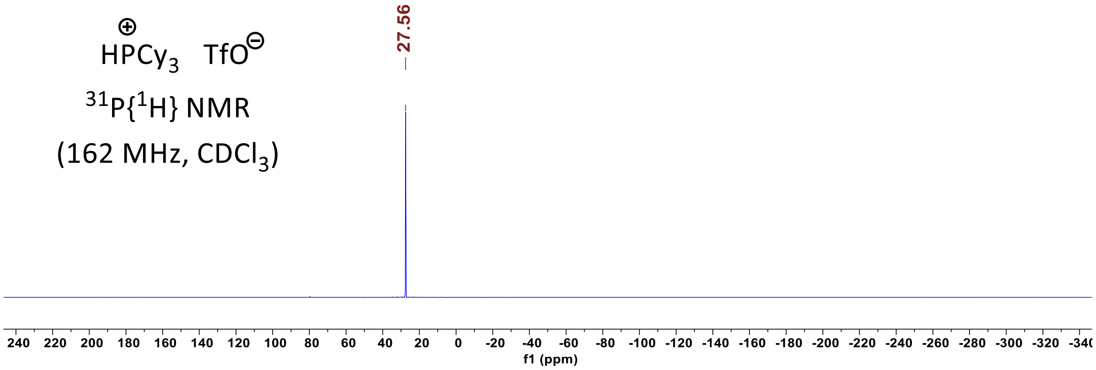
HPCy3 TfO
31P{1H} NMR
(162 MHz, CDCl3)
27.56
240 220 200 180 160 140 120 100 80 60 40 20 0 -20 -40 -60 -80 -100 -120 -140 -160 -180 -200 -220 -240 -260 -280 -300 -320
f1 (ppm)

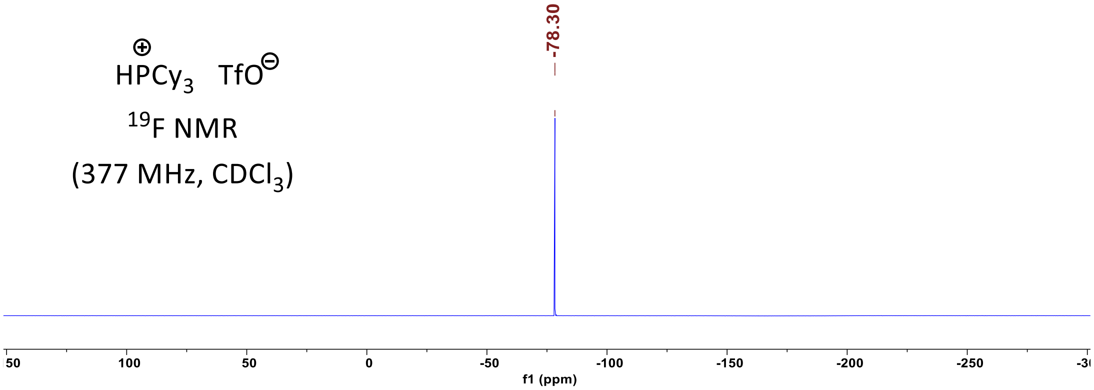
HPCy3 TfO
19F NMR
(377 MHz, CDCl3)
-78.30
50 100 50 0 -50 -100 -150 -200 -250
f1 (ppm)

2.3.18 Trioctylphosphonium triflate

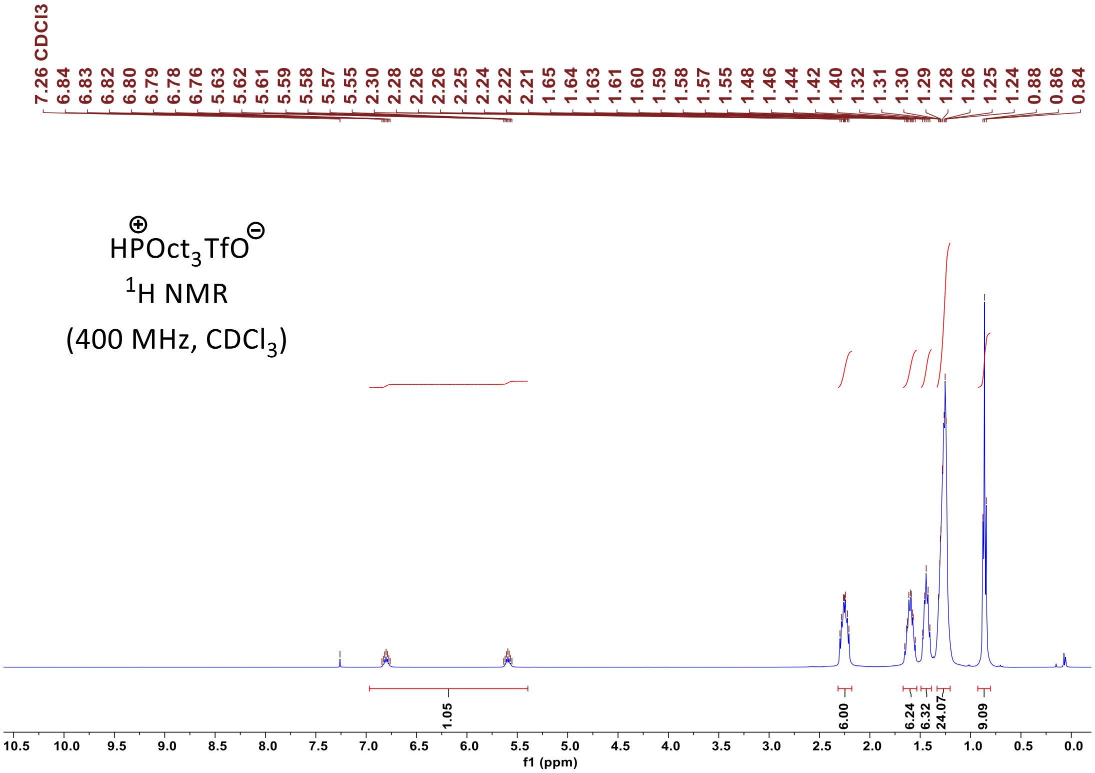

f415

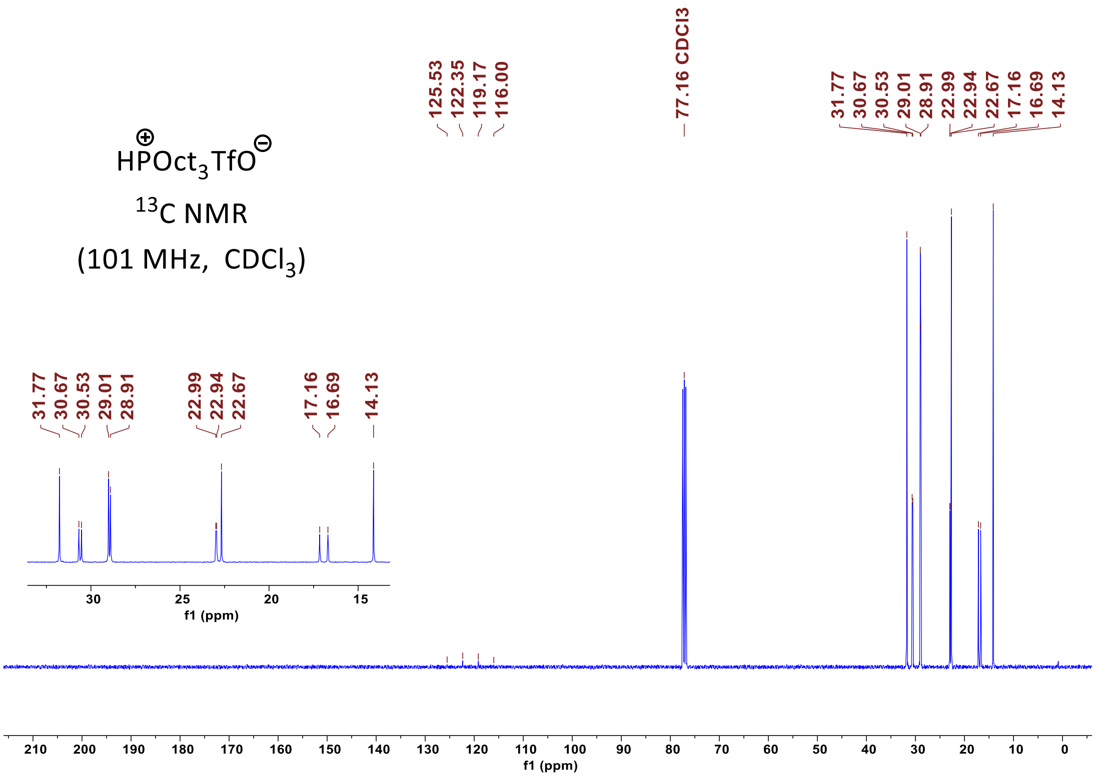

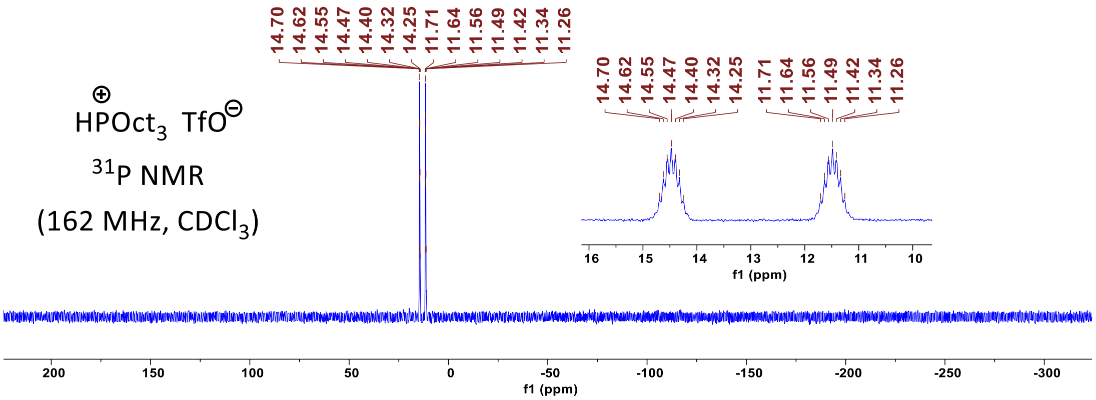
HPOct3 TfO
31P NMR
(162 MHz, CDCl3)
14.70
14.62
14.55
14.47
14.40
14.32
14.25
11.71
11.64
11.56
11.49
11.42
11.34
11.26
f1 (ppm)

2.3.19 Tri(naphthalen-1-yl)phosphane

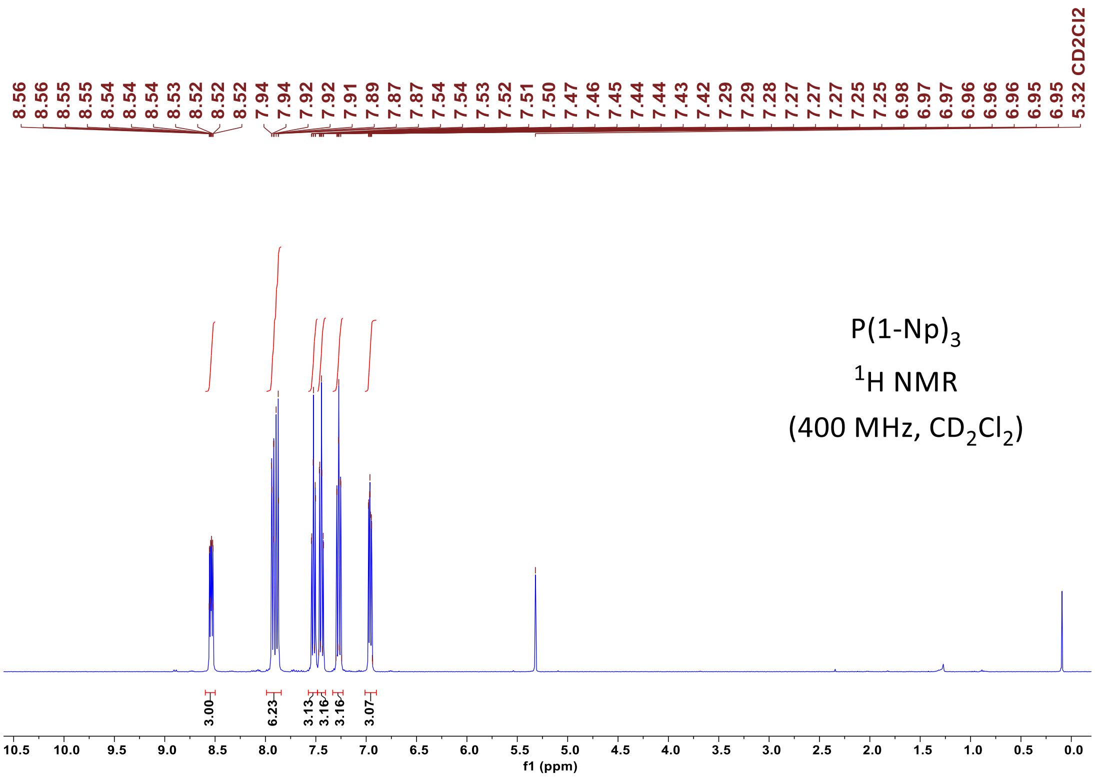

f505

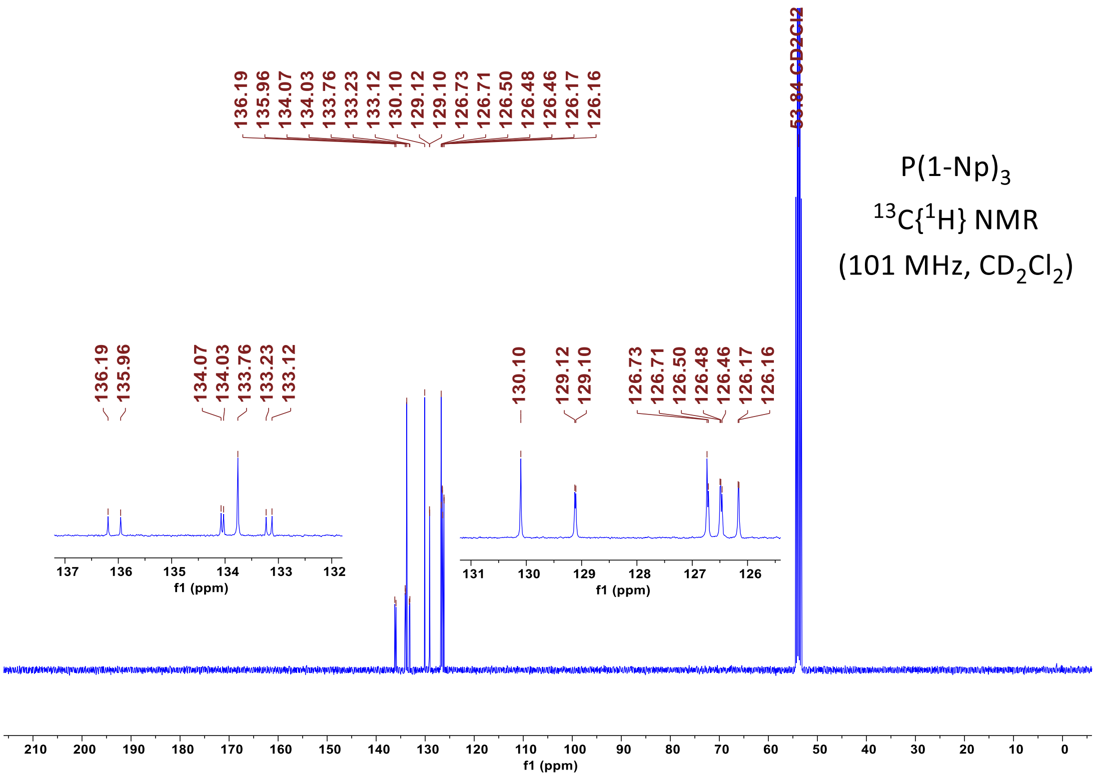

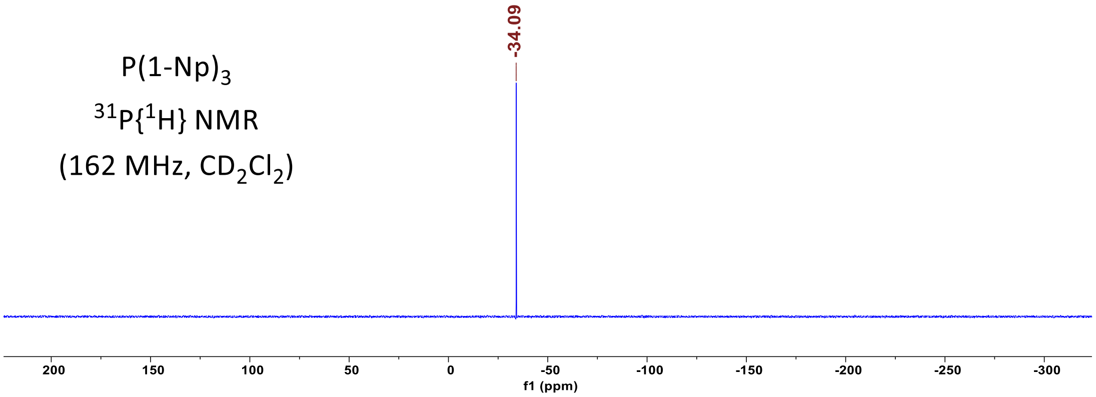

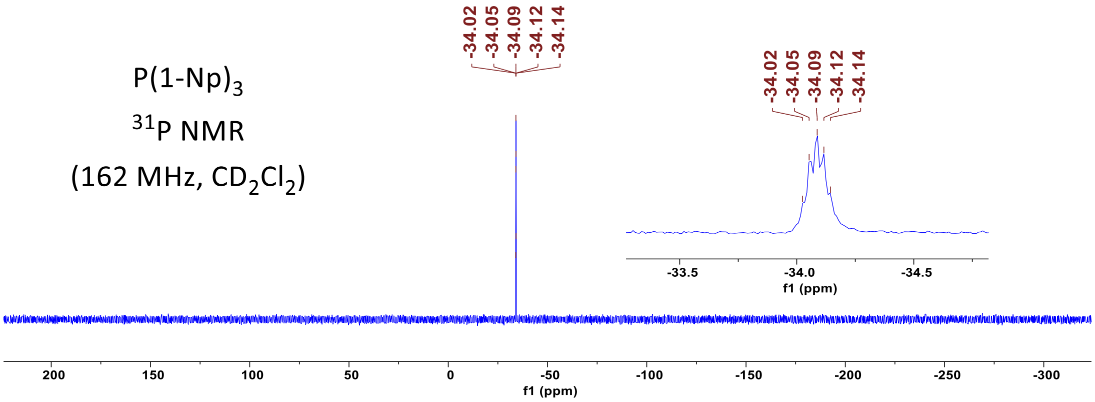

2.3.20 Triphenylphosphine oxide

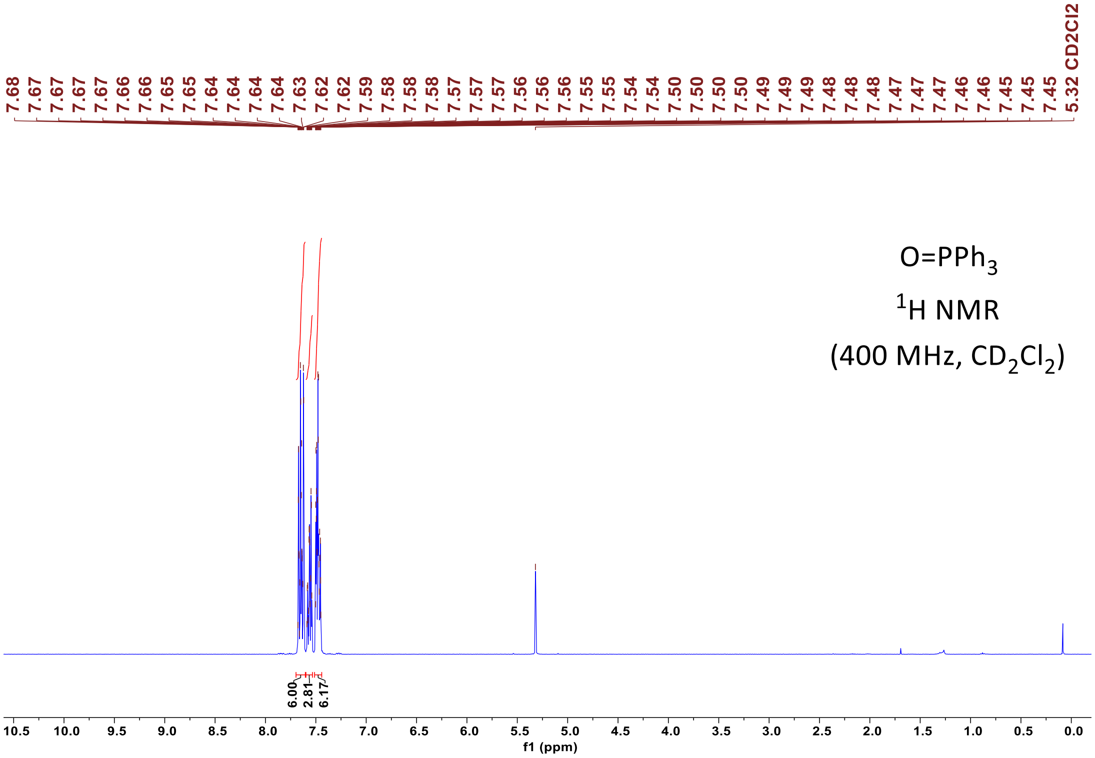

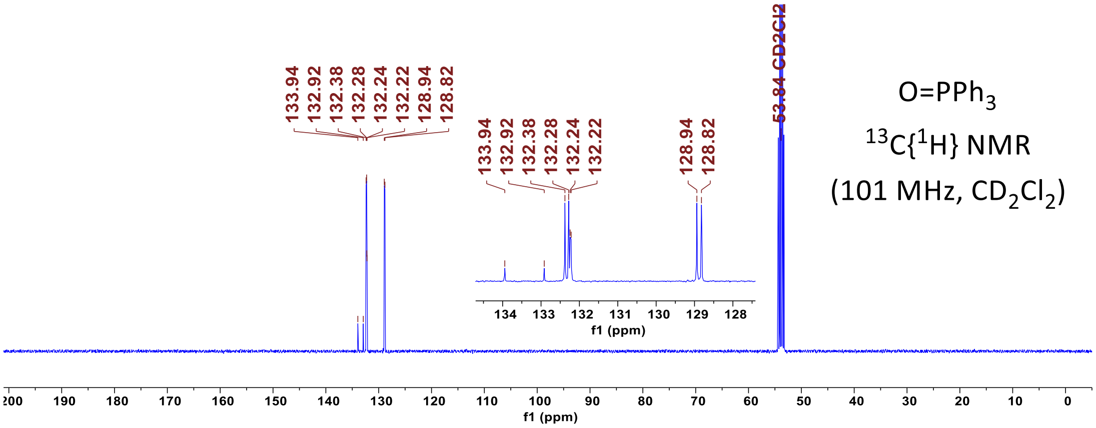
133.94
132.92
132.38
132.28
132.24
132.22
128.94
128.82
53.84 CD2Cl2
f1 (ppm)
O=PPh3
13C{1H} NMR
(101 MHz, CD2Cl2)

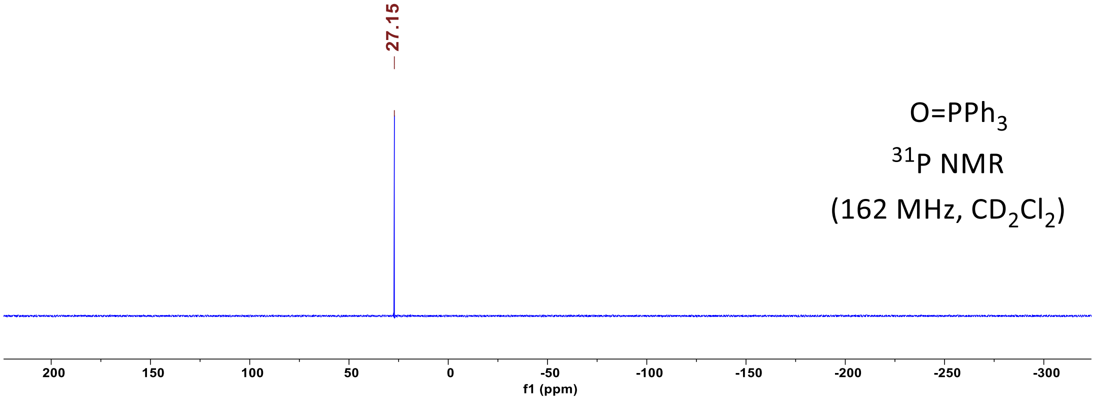
27.15
f1 (ppm)
O=PPh3
31P NMR
(162 MHz, CD2Cl2)

2.3.21 Triethylamine

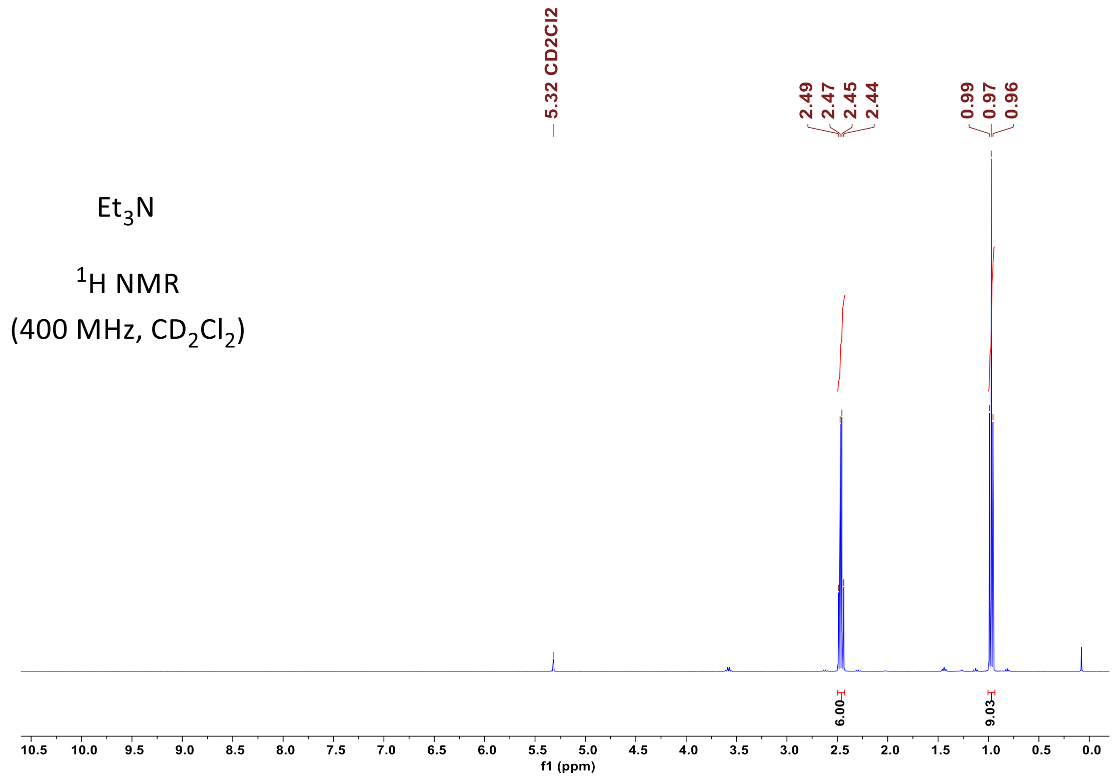

f428

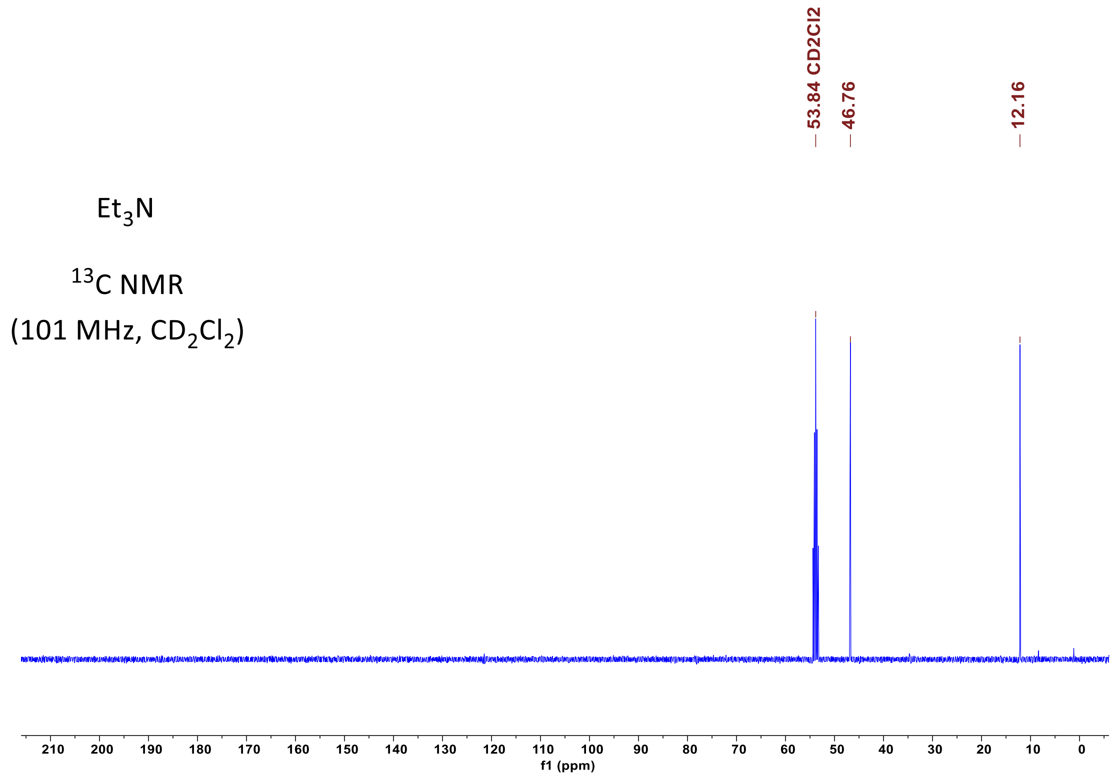

2.3.22 Triethylammonium triflate (TEAT)

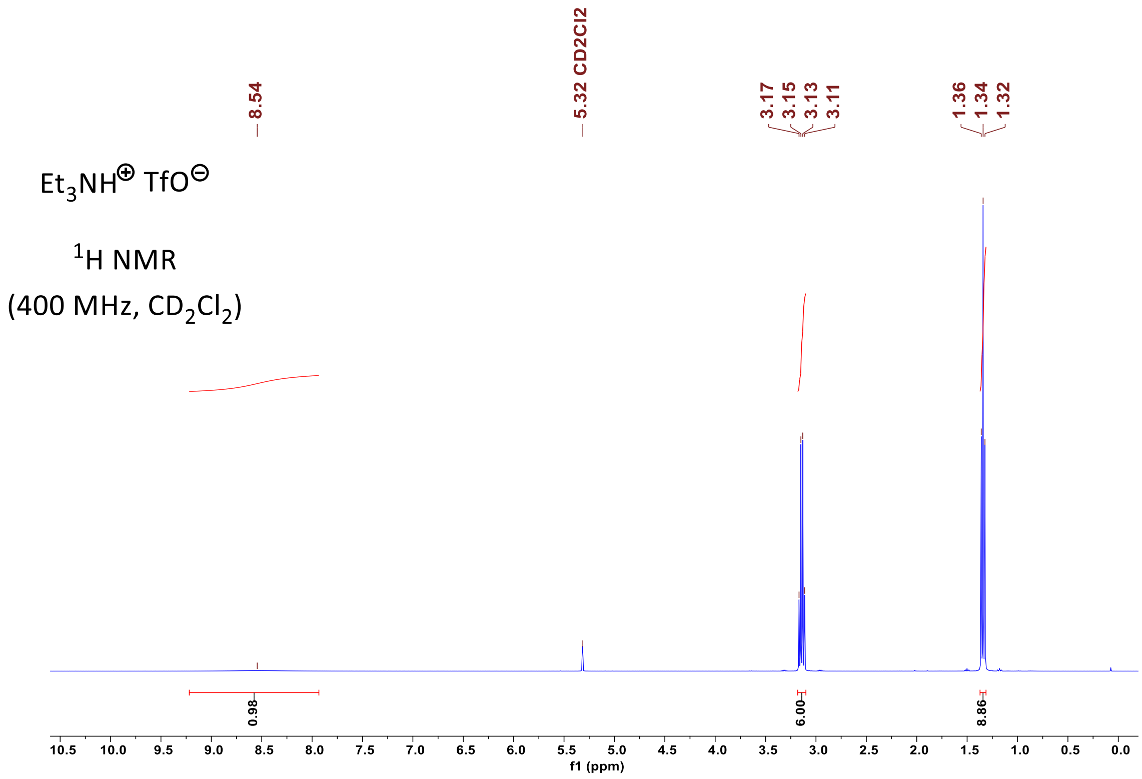

f422

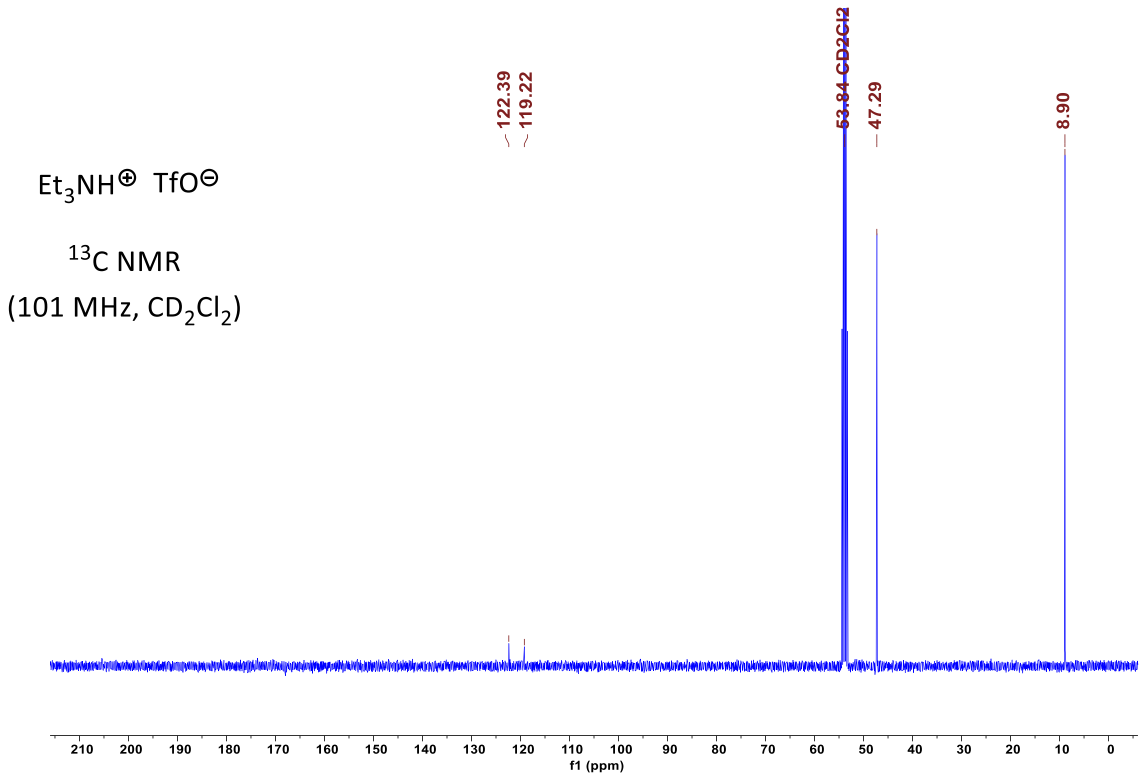

2.3.23 2,4,6-Collidinium triflate (CT)

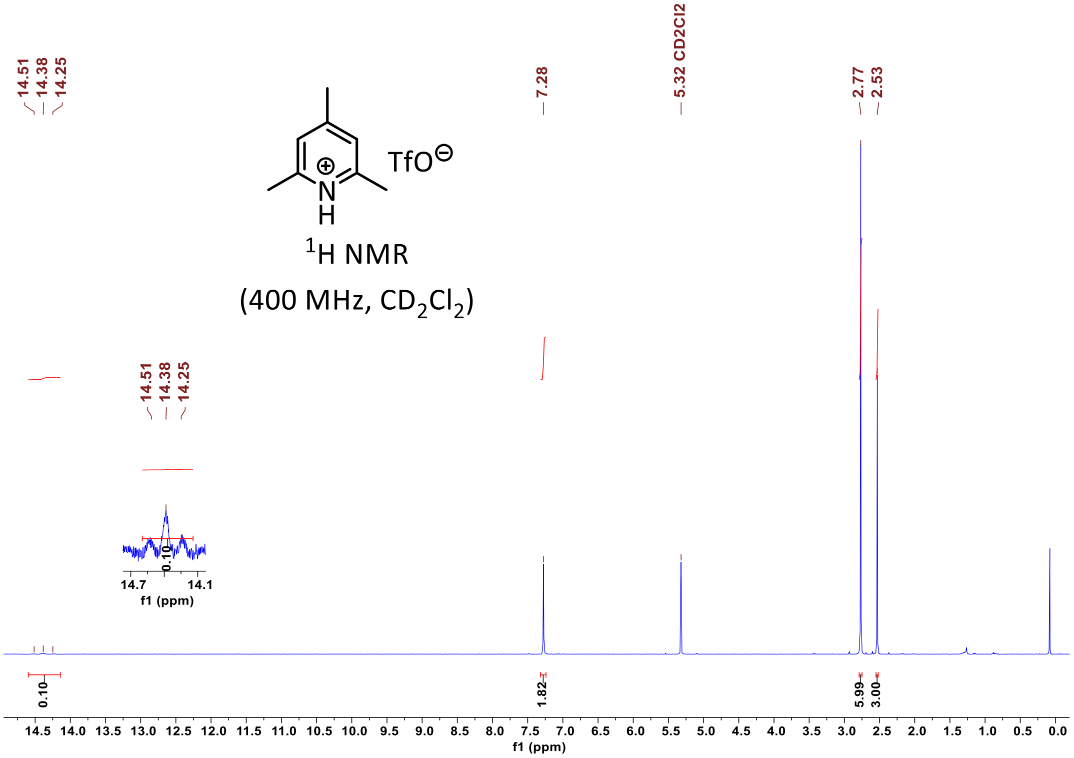

f499

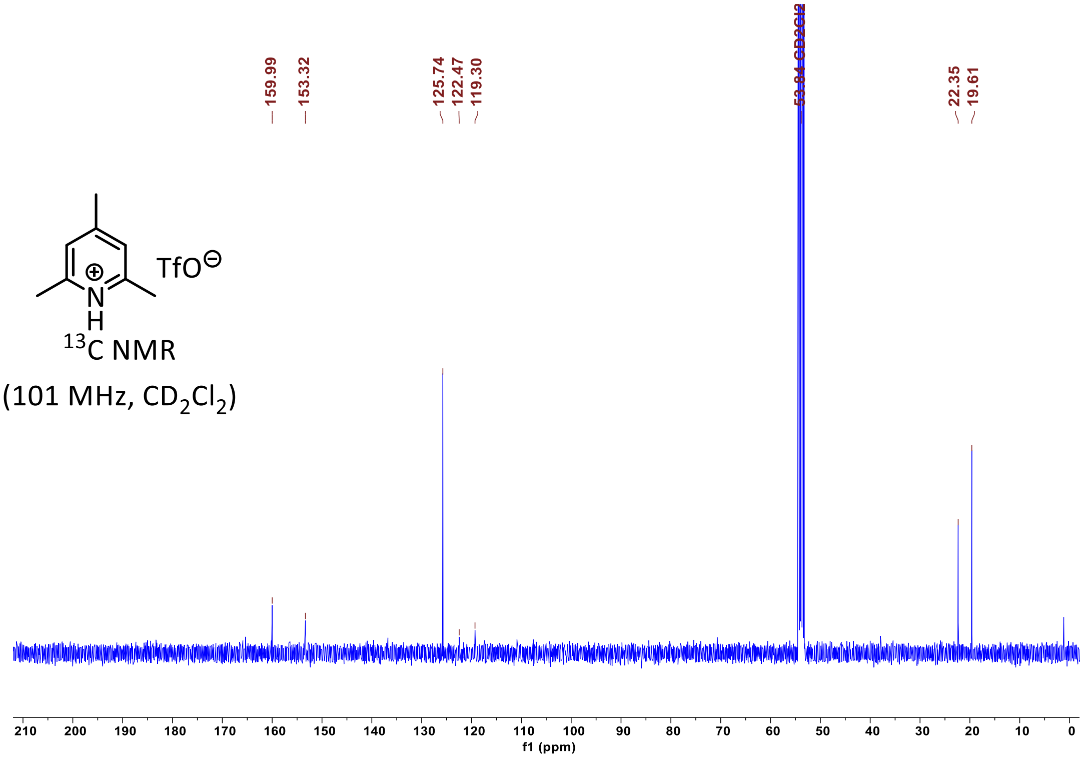

2.4 Product Studies

2.4.1 Reaction of triphenylphosphonium triflate (TPPT) with ethyl acrylate (1)

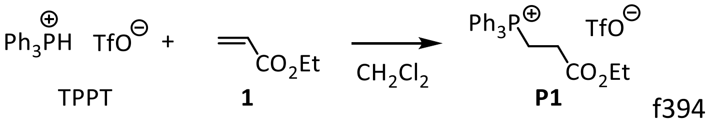

TPPT (200 mg, 0.485 mmol) and **1** (63.4 mg, 0.633 mmol) were mixed in dichloromethane (5 mL). The mixture was kept under inert gas atmosphere at ambient temperature for 5 days. The volatiles were removed by reduced pressure. The residue was washed by diethyl ether and dried under vacuum to provide (3-ethoxy-3-oxopropyl)triphenylphosphonium triflate **P1** as a white solid (247 mg, 99%); mp 121.5–122.8 °C.

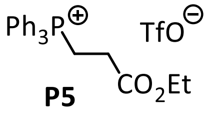

^{1}H NMR (400 MHz, CD_2Cl_2) δ 7.90–7.85 (m, 3 H), 7.76–7.67 (m, 12 H), 4.02 (q, J = 7.1 Hz, 2 H), 3.59 (dt, J = 12.7, 7.5 Hz, 2 H), 2.78 (dt, J = 14.0, 7.5 Hz, 2 H), 1.16 (t, J = 7.2 Hz, 3 H). **^{13}C{^{1}H} NMR** (101 MHz, CD_2Cl_2) δ 170.3 (d, $J_{C,P}$ = 13.2 Hz, C_q), 136.0 (d, $J_{C,P}$ = 3.1 Hz, CH), 133.9 (d, $J_{C,P}$ = 10.0 Hz, CH), 131.1 (d, $J_{C,P}$ = 12.7 Hz, CH), 121.4 (q, $^{1}J_{C,F}$ = 321.2 Hz, CF_3), 117.6 (d, $^{1}J_{C,P}$ = 87.0 Hz, C_q), 62.3 (CH_2), 27.3 (d, $^{2}J_{C,P}$ = 2.8 Hz, CH_2), 18.6 (d, $^{1}J_{C,P}$ = 55.9 Hz, CH_2), 14.2 (CH_3). **^{31}P{^{1}H} NMR** (162 MHz, CD_2Cl_2) δ 24.2. **^{19}F NMR** (377 MHz, CD_2Cl_2) δ −78.9. **IR** (neat, ATR): $\tilde{\nu}$ = 2915, 1730, 1588, 1485, 1441, 1417, 1391, 1375, 1263, 1238, 1225, 1182, 1137, 1110, 1055, 1031, 996, 745, 724, 690 cm^{-1}. **HRMS** (ESI): m/z calcd for $C_{23}H_{24}O_2P^+$ (M – TfO$^-$): 363.15084, found: 363.15075.

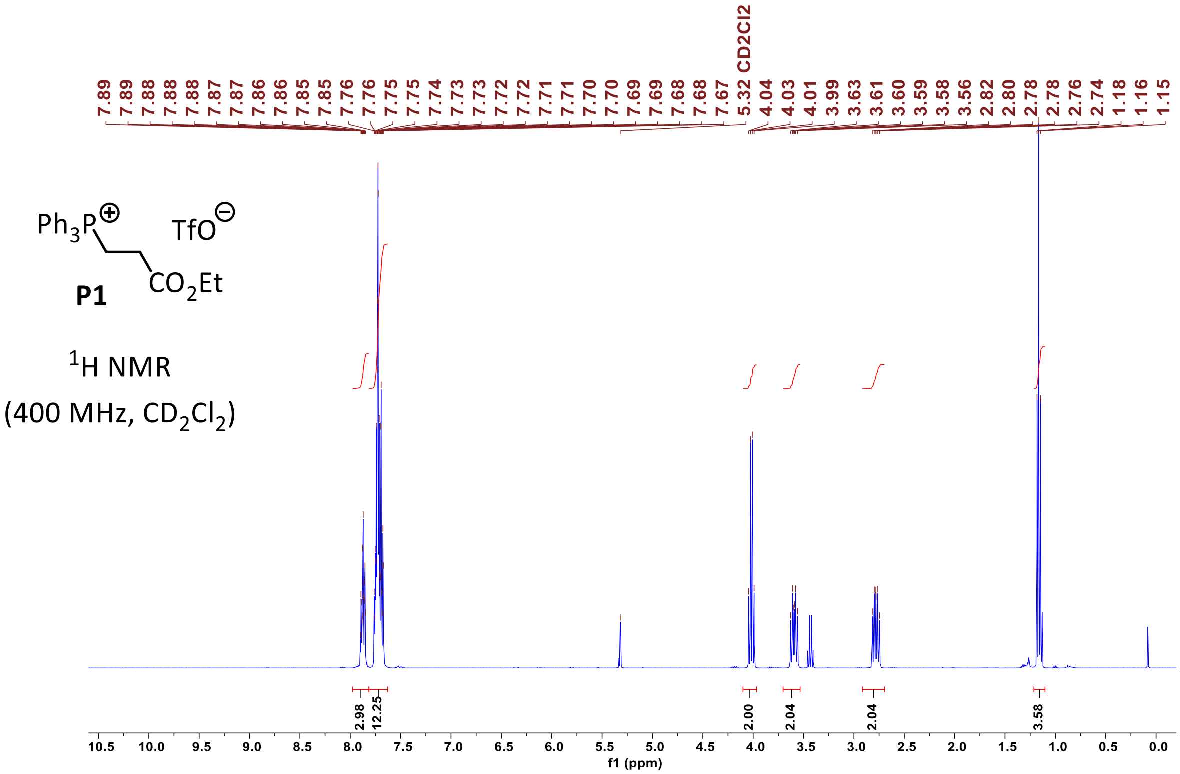

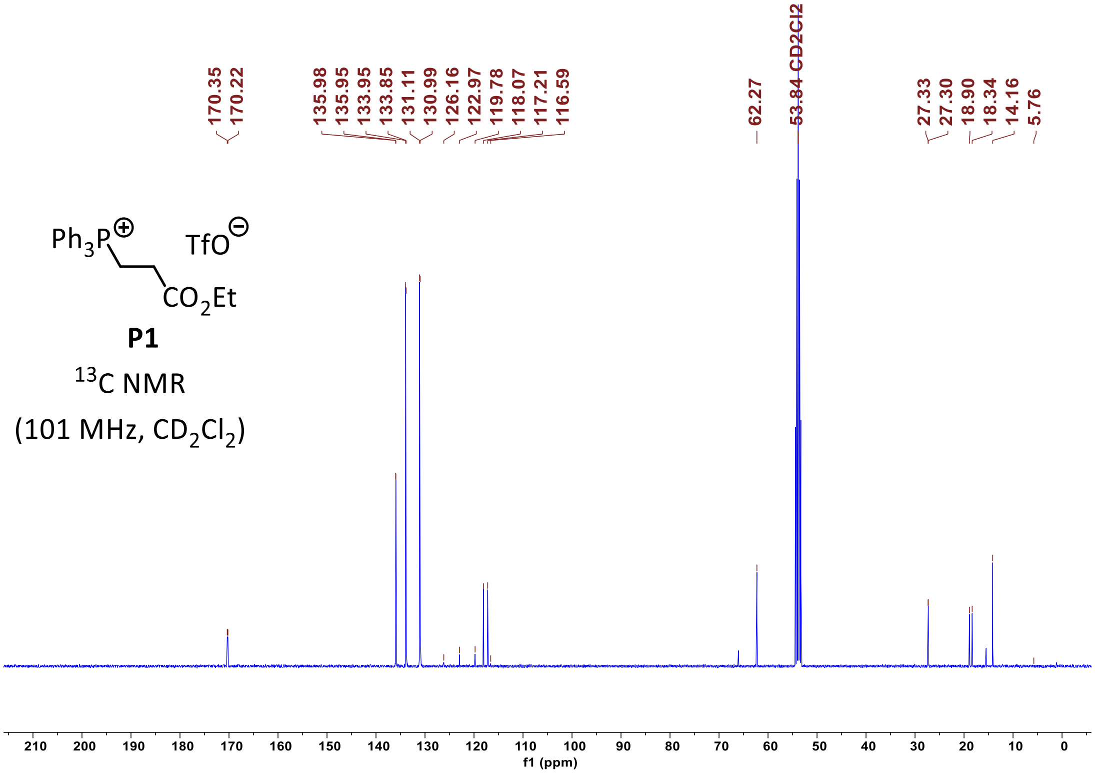
170.35
170.22
135.98
135.95
133.95
133.85
131.11
130.99
126.16
122.97
119.78
118.07
117.21
116.59
62.27
53.84 CD2Cl2
27.33
27.30
18.90
18.34
14.16
5.76
Ph3P⊕ TfO⊖
CO2Et
P1
13C NMR
(101 MHz, CD2Cl2)
210 200 190 180 170 160 150 140 130 120 110 100 90 80 70 60 50 40 30 20 10 0
f1 (ppm)

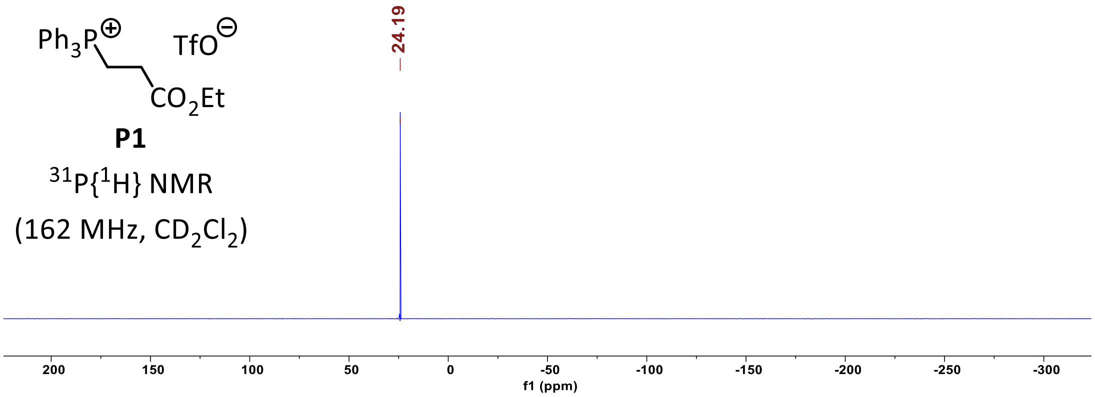
24.19
Ph3P⊕ TfO⊖
CO2Et
P1
31P{1H} NMR
(162 MHz, CD2Cl2)
200 150 100 50 0 -50 -100 -150 -200 -250 -300
f1 (ppm)

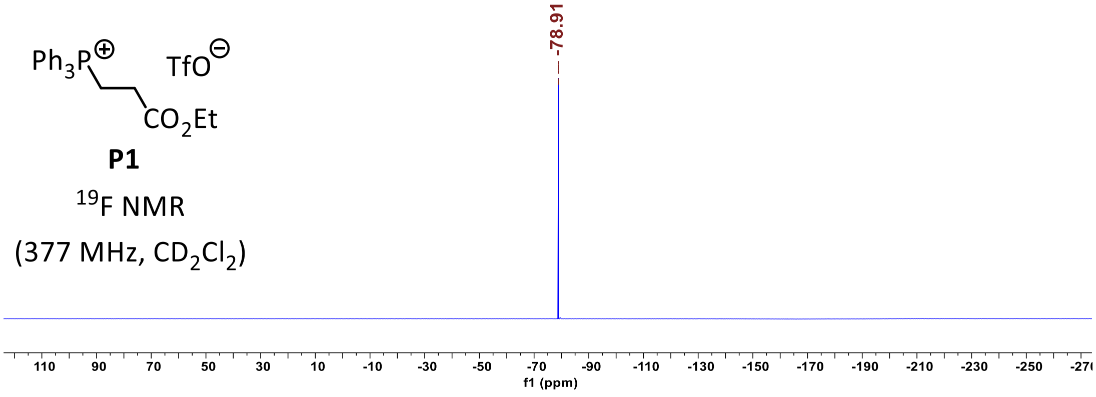
-78.91
Ph3P⊕ TfO⊖
CO2Et
P1
19F NMR
(377 MHz, CD2Cl2)
110 90 70 50 30 10 -10 -30 -50 -70 -90 -110 -130 -150 -170 -190 -210 -230 -250 -270
f1 (ppm)

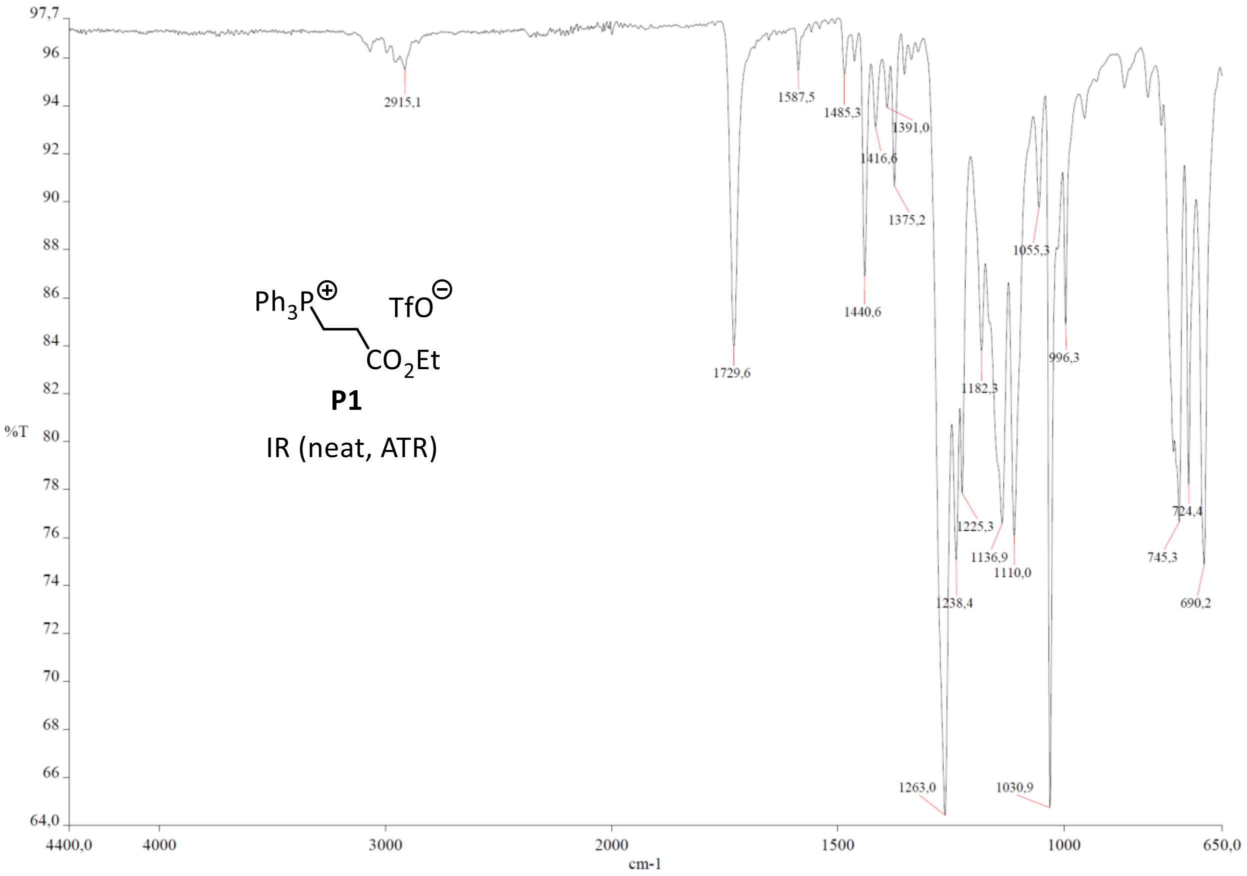

2.4.2 Reaction of triphenylphosphonium triflate (TPPT) with ethyl buta-2,3-dienoate (2)

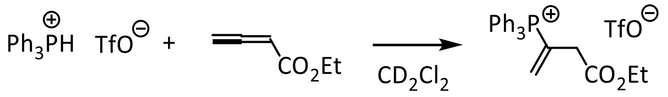

TPPT **2** **P2** f396

As originally reported in ref S6, TPPT (186 mg, 451 mmol) and **2** (54.5 mg, 486 mmol) were mixed in CD_2Cl_2 (1.5 mL). The mixture was kept under inert gas atmosphere at ambient temperature for 40 h. The volatiles were removed under reduced pressure. The residue was washed with pentane and dried under high vacuum to provide **P2** (230 mg, 97%) as a white solid; mp 97.3–98.3 °C.[S6]

^{1}H NMR (600 MHz, CD_2Cl_2) δ 7.92–7.89 (m, 3 H), 7.76–7.68 (m, 12 H), 6.94 (dd, $J_{H,P}$ = 45.6 Hz, $J_{H,H}$ = 1.2 Hz, 1 H), 6.29 (dd, $J_{H,P}$ = 21.0 Hz, $J_{H,H}$ = 1.2 Hz, 1 H), 3.86 (q, $J_{H,H}$ = 7.1 Hz, 2 H), 3.56 (dd, $J_{H,P}$ = 15.4 Hz, $J_{H,H}$ = 1.1 Hz, 2 H), 1.06 (t, $J_{H,H}$ = 7.1 Hz, 3 H). **^{13}C{^{1}H} NMR** (151 MHz, CD_2Cl_2) δ 169.2 (d, $^3J_{C,P}$ = 3.1 Hz, C_q), 145.6–145.5 (m, =CH_2), 136.1 (d, $J_{C,P}$ = 3.1 Hz, CH), 134.9 (d, $J_{C,P}$ = 10.3 Hz, CH), 130.9 (d, $J_{C,P}$ = 12.9 Hz,

CH), 125.3 (d, $^{1}J_{C,P}$ = 78.1 Hz, C_q), 121.4 (q, $^{1}J_{C,F}$ = 321.5 Hz, CF_3), 117.1 (d, $^{1}J_{C,P}$ = 88.6 Hz, C_q), 62.3 (CH_2), 39.2 (d, $^{2}J_{C,P}$ = 11.6 Hz, CH_2), 14.1 (CH_3). **^{31}P NMR** (162 MHz, CD_2Cl_2) δ 25.3.[S8] **^{19}F NMR** (377 MHz, CD_2Cl_2) δ −78.9. **IR** (neat, ATR) $\tilde{\nu}$ = 2983, 1732, 1586, 1484, 1441, 1334, 1258, 1223, 1204, 1154, 1107, 1027, 996, 970, 948, 861, 755, 724, 712, 690, 658 cm^{-1}. **HRMS** (ESI): *m/z* calcd for $C_{24}H_{24}O_2P^+$ (M – TfO^-): 375.15084, found: 375.15072.

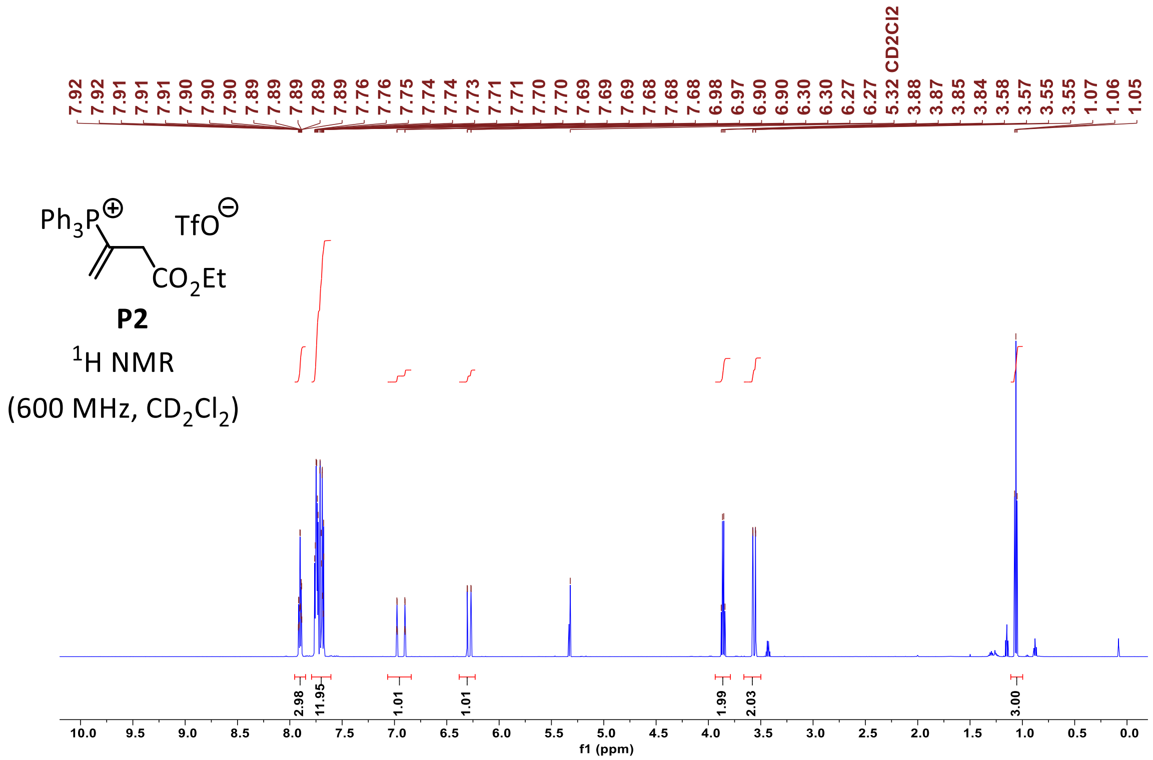

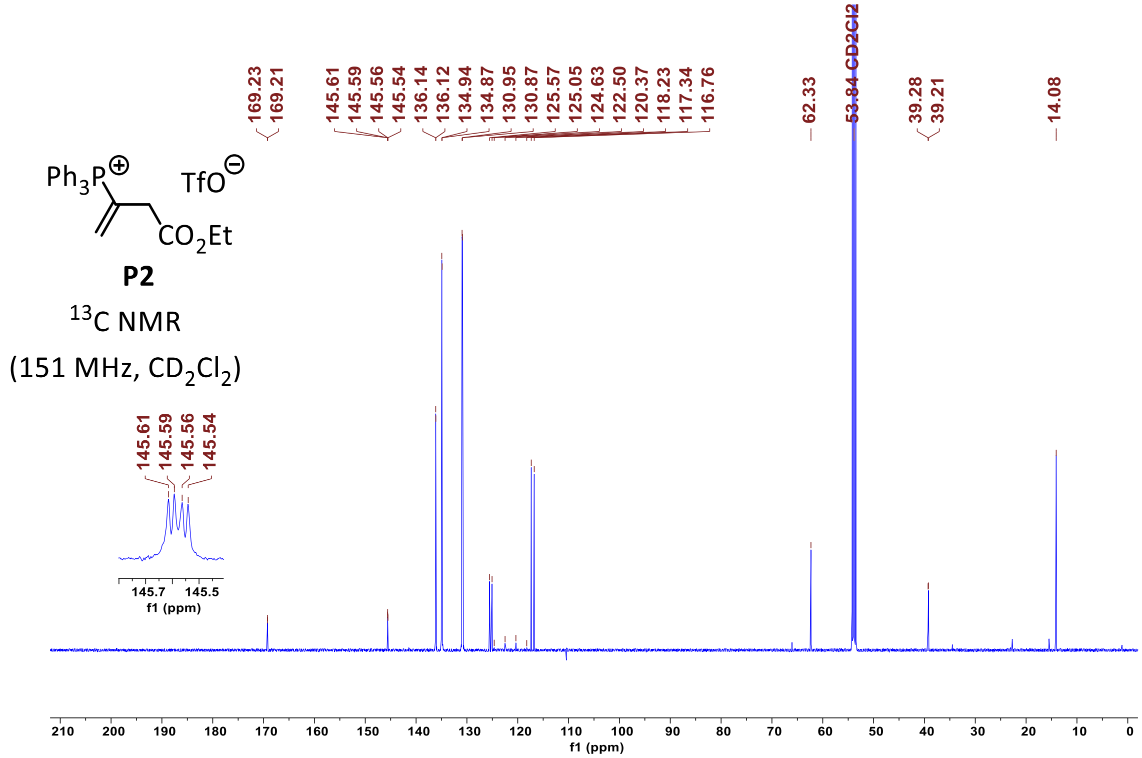
Ph3P⊕ TfO⊖
CO2Et
P2
13C NMR
(151 MHz, CD2Cl2)
169.23
169.21
145.61
145.59
145.56
145.54
136.14
136.12
134.94
134.87
130.95
130.87
125.57
125.05
124.63
122.50
120.37
118.23
117.34
116.76
62.33
53.84 CD2Cl2
39.28
39.21
14.08
145.7
145.5
f1 (ppm)
210 200 190 180 170 160 150 140 130 120 110 100 90 80 70 60 50 40 30 20 10 0
f1 (ppm)

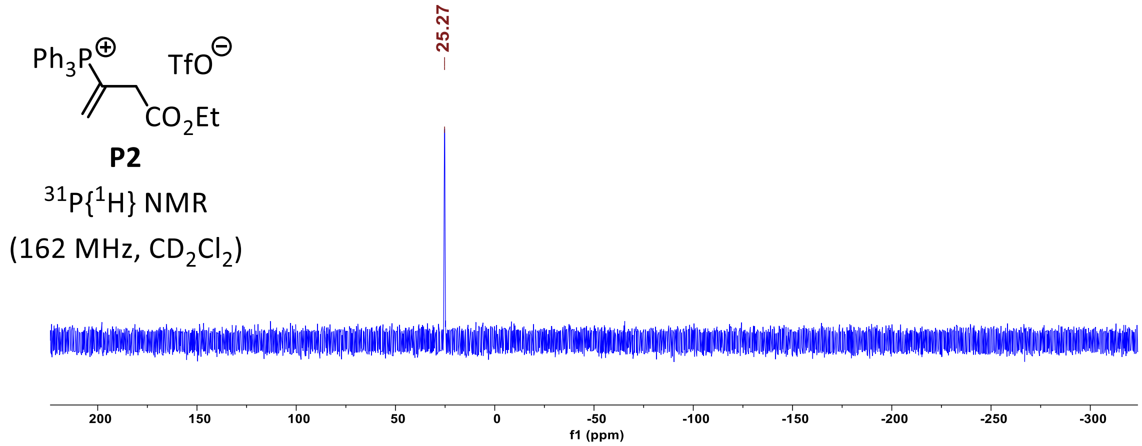
Ph3P⊕ TfO⊖
CO2Et
P2
31P{1H} NMR
(162 MHz, CD2Cl2)
25.27
200 150 100 50 0 -50 -100 -150 -200 -250 -300
f1 (ppm)

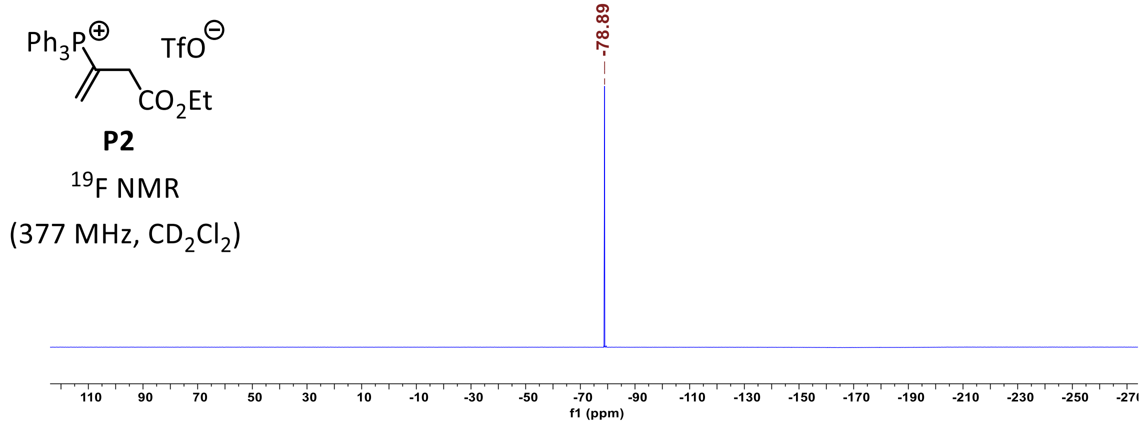
Ph3P⊕ TfO⊖
CO2Et
P2
19F NMR
(377 MHz, CD2Cl2)
-78.89
110 90 70 50 30 10 -10 -30 -50 -70 -90 -110 -130 -150 -170 -190 -210 -230 -250 -270
f1 (ppm)

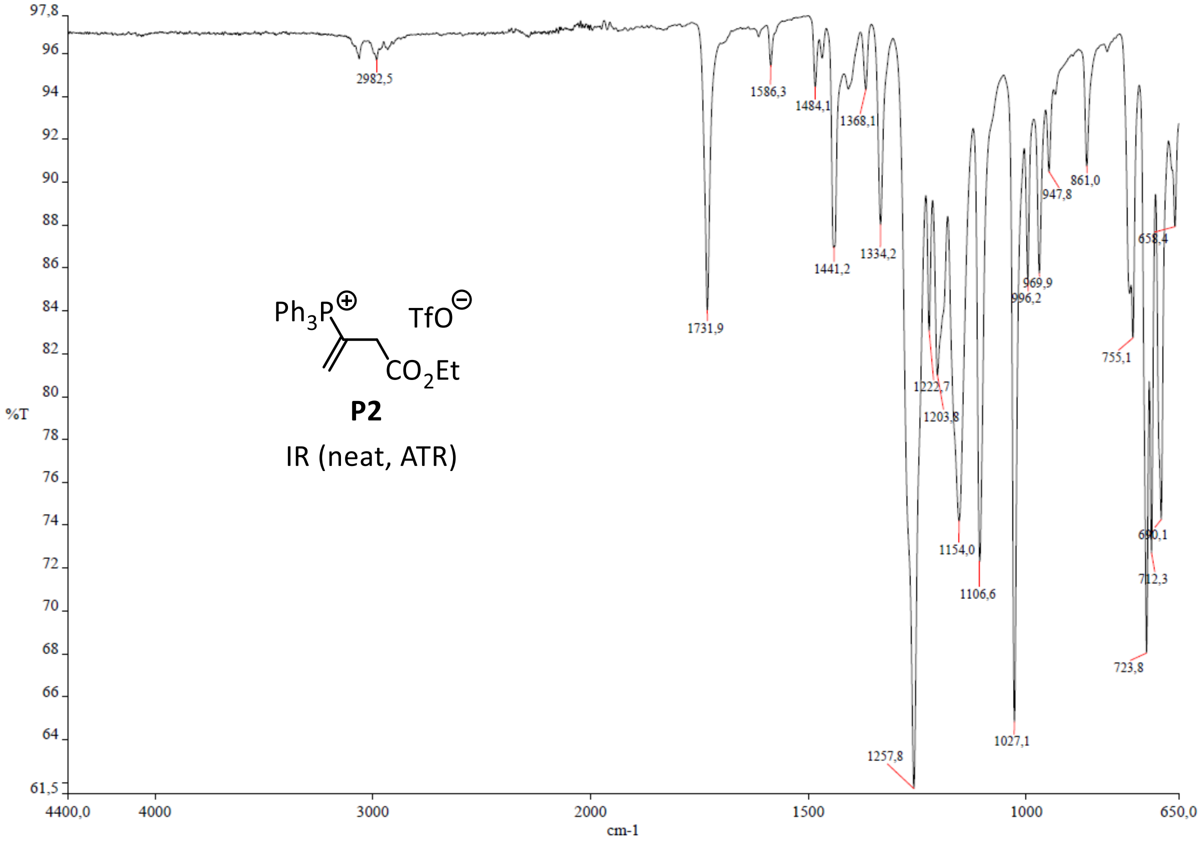
Ph3P⊕ TfO⊖
CO2Et
P2
IR (neat, ATR)
%T
cm-1
2982,5
1731,9
1586,3
1484,1
1441,2
1368,1
1334,2
1257,8
1222,7
1203,8
1154,0
1106,6
1027,1
996,2
969,9
947,8
861,0
755,1
723,8
712,3
690,1
658,4

2.4.3 Reaction of triphenylphosphonium triflate (TPPT) with ethyl propiolate (3)

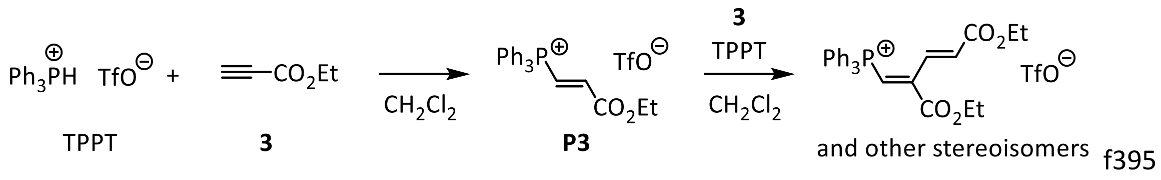

TPPT (205 mg, 0.497 mmol) and **3** (63.0 mg, 0.642 mmol) were mixed in dichloromethane (5 mL). The mixture was kept under inert gas atmosphere at ambient temperature for 7 days. The volatiles were removed under reduced pressure. The residue was washed by diethyl ether and dried under vacuum to provide a white solid, which consists of **P3** (approx. .50 wt%) and 2:1 products (mixture of stereoisomers).

^{1}H NMR (400 MHz, CD_2Cl_2) δ 7.97–7.59 (m, 16 H), 6.63 (dd, *J* = 19.8, 16.8 Hz, 1 H), 4.34 (q, *J* = 7.1 Hz, 2 H), 1.34 (t, *J* = 7.1 Hz, 3 H). **^{13}C NMR** (101 MHz, CD_2Cl_2) δ 162.7, 146.1 (d, *J* = 4.4 Hz), 136.7 (d, *J* = 3.1 Hz), 134.4 (d, *J* = 10.8 Hz), 131.4 (d, *J* = 13.3 Hz), 124.4 (d, *J* = 83.8 Hz), 121.3 (q, $^1J_{C,F}$ = 321.1 Hz, CF_3), 116.1 (d, *J* = 91.4 Hz), 63.3, 14.2. **^{31}P NMR** (162 MHz, CD_2Cl_2) δ 19.2 (m). **HRMS** (ESI): *m/z* calcd for $C_{23}H_{22}O_2P^+$ (M – TfO^-): 361.13519, found: 361.13553; *m/z* calcd for the 2:1-product $C_{28}H_{28}O_4P^+$ (M – TfO^-): 459.17197, found: 459.17234.

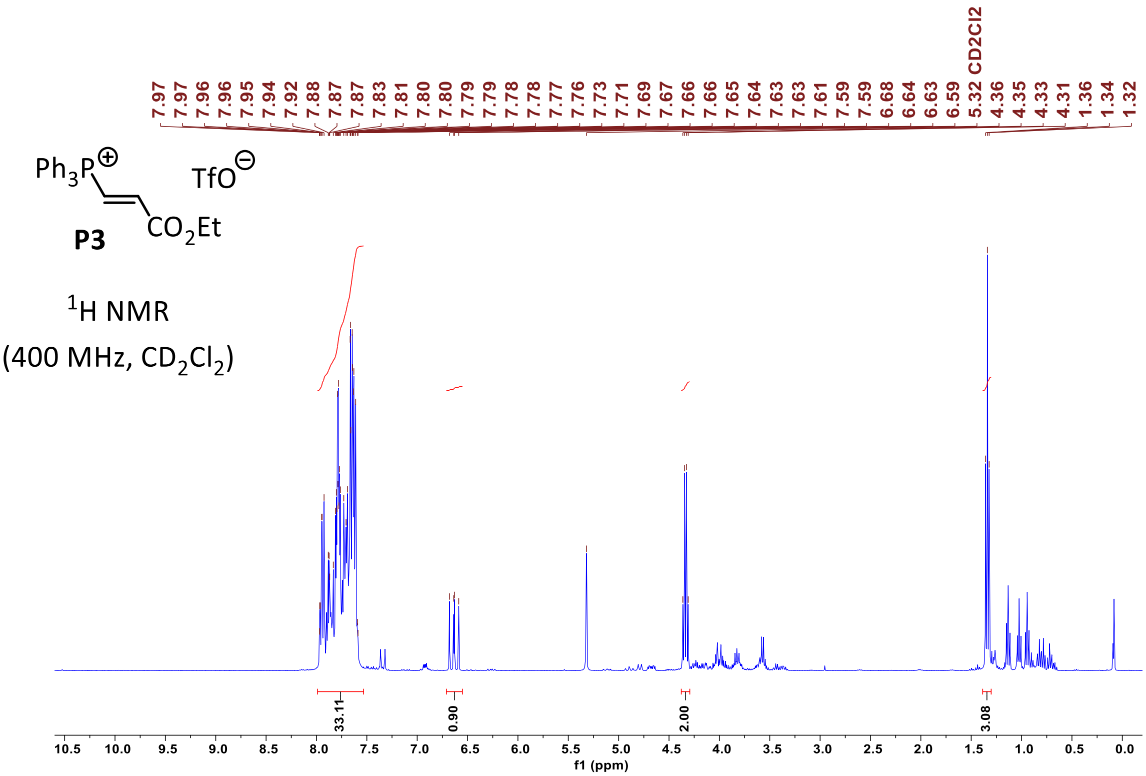

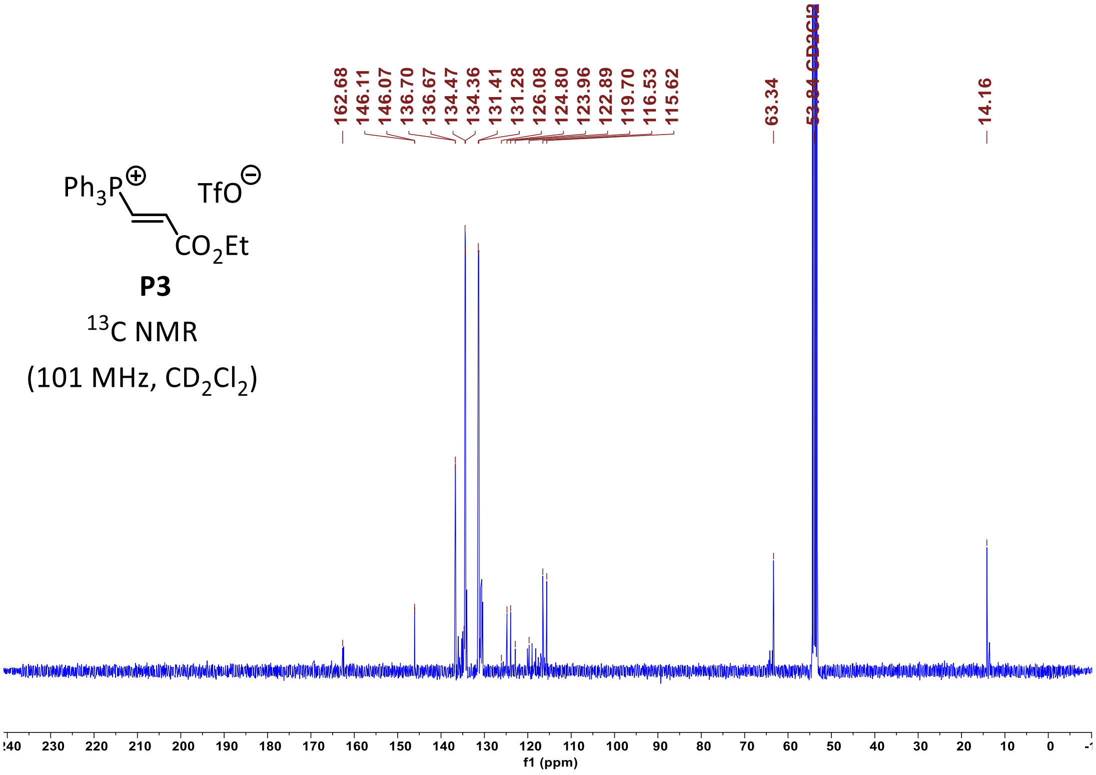
Ph3P⊕ TfO⊖
CO2Et
P3
13C NMR
(101 MHz, CD2Cl2)
162.68
146.11
146.07
136.70
136.67
134.47
134.36
131.41
131.28
126.08
124.80
123.96
122.89
119.70
116.53
115.62
63.34
53.84 CD2Cl2
14.16
240 230 220 210 200 190 180 170 160 150 140 130 120 110 100 90 80 70 60 50 40 30 20 10 0
f1 (ppm)

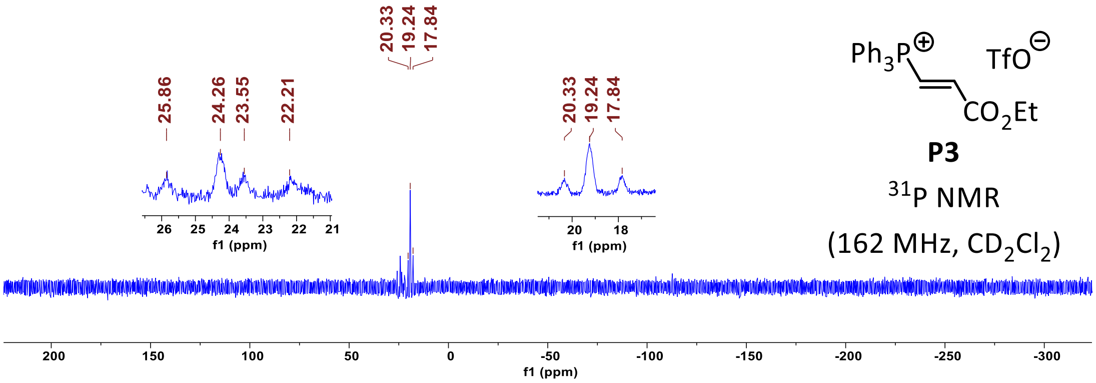
20.33
19.24
17.84
25.86
24.26
23.55
22.21
26 25 24 23 22 21
f1 (ppm)
20.33
19.24
17.84
20 18
f1 (ppm)
Ph3P⊕ TfO⊖
CO2Et
P3
31P NMR
(162 MHz, CD2Cl2)
200 150 100 50 0 -50 -100 -150 -200 -250 -300
f1 (ppm)

Probenname: C:\Data\09-feanch-f395 Probe: 361, c23h22o2p, fest, Chloroform Inj Vol: 1.000000
Auftraggeber: An, Offial Methode: 100 ul/min Acetonitril/Wasser, FIA/ESI, LTQ FT, Spahl Zeit:

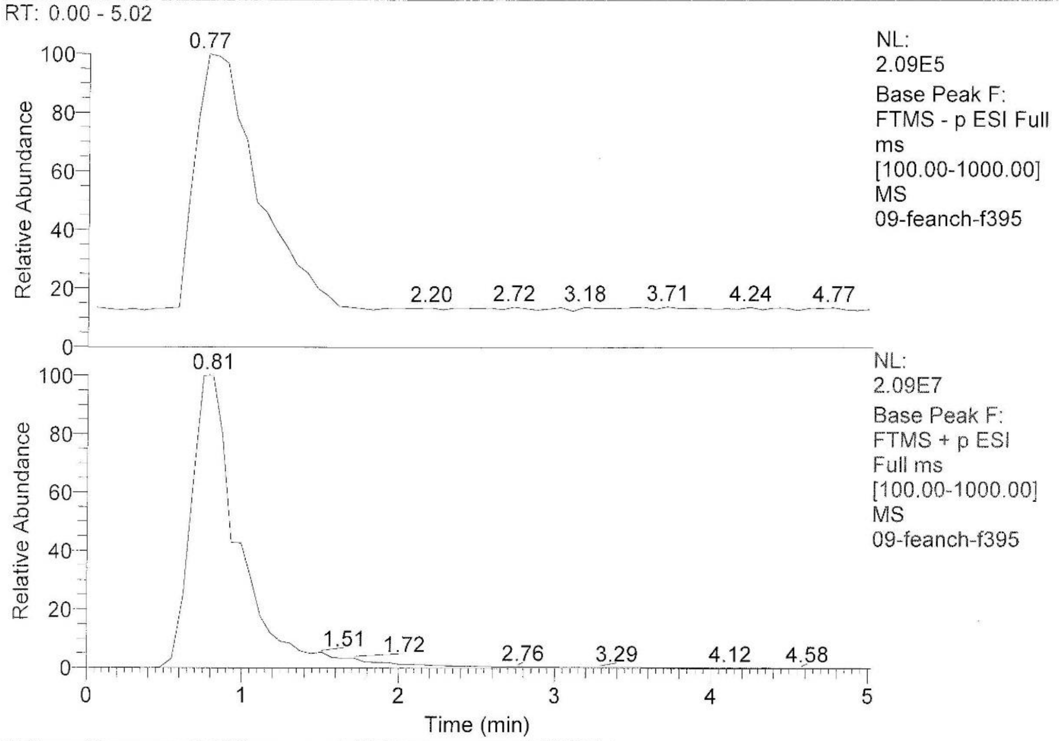

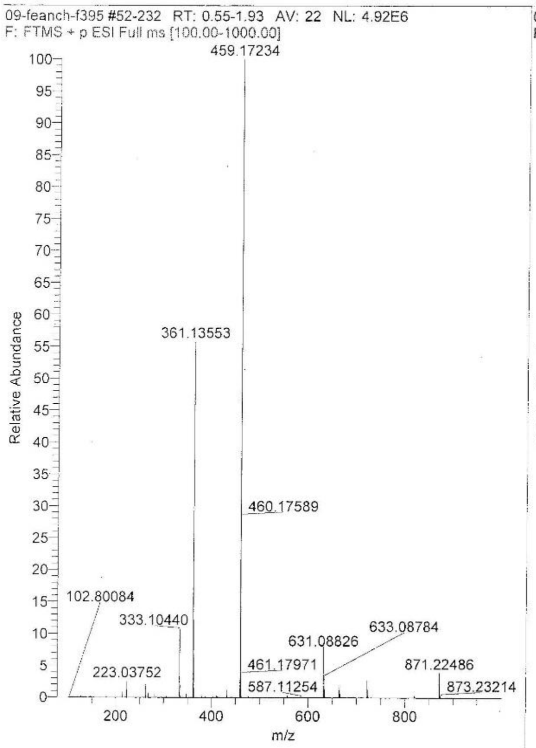

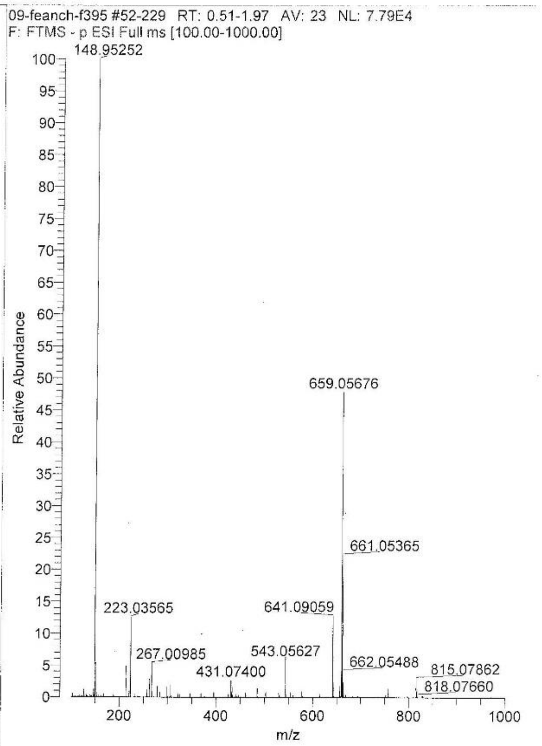

2.4.4 Reaction of triphenylphosphonium triflate with ethenesulfonyl fluoride (4)

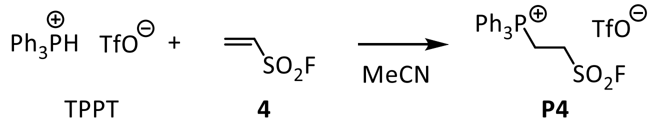

f603/f610

TPPT (37.4 mg, 0.0907 mmol) and **4** (17.8 mg, 0.162 mmol) were mixed in acetonitrile (1.0 mL). The reaction mixture was kept under inert gas atmosphere at ambient temperature for 1 day. Then, the volatiles were removed under reduced pressure. The residue was washed with pentane and dried under vacuum to provide **P4** as a white solid (46.9 mg, 99%).

^{1}H NMR (800 MHz, CD_2Cl_2) δ 7.93–7.91 (m, 3 H), 7.79–7.72 (m, 12 H), 3.98–3.88 (m, 4 H). **^{13}C NMR** (101 MHz, CD_2Cl_2) δ 136.5 (d, $J_{C,P}$ = 3.1 Hz, CH), 134.1 (d, $J_{C,P}$ = 10.4 Hz, CH), 131.4 (d, $J_{C,P}$ = 13.1 Hz, CH), 116.2 (d, $^1J_{C,P}$ = 87.8 Hz, C_q), 44.4 (d, $^2J_{C,P}$ = 20.7 Hz, CH_2), 18.9 (d, $^1J_{C,P}$ = 55.6 Hz, CH_2; the ^{13}C resonance of the CF_3 group could not be detected. **^{31}P{^{1}H} NMR** (162 MHz, CD_2Cl_2) δ 23.5. **^{19}F NMR** (377 MHz, CD_2Cl_2) δ 54.2 (m, 1 F, SO_2F), −78.9 (s, 3 F, CF_3). **IR** (neat, ATR) $\tilde{\nu}$ = 3065, 2926, 1589, 1486, 1440, 1414, 1259, 1225, 1199, 1155, 1113, 1030, 998, 784, 741, 690 cm^{-1}. **HRMS** (ESI): *m*/*z* calcd for $C_{20}H_{19}FO_2PS^+$ (M − TfO^-): 373.08219, found: 373.08217.

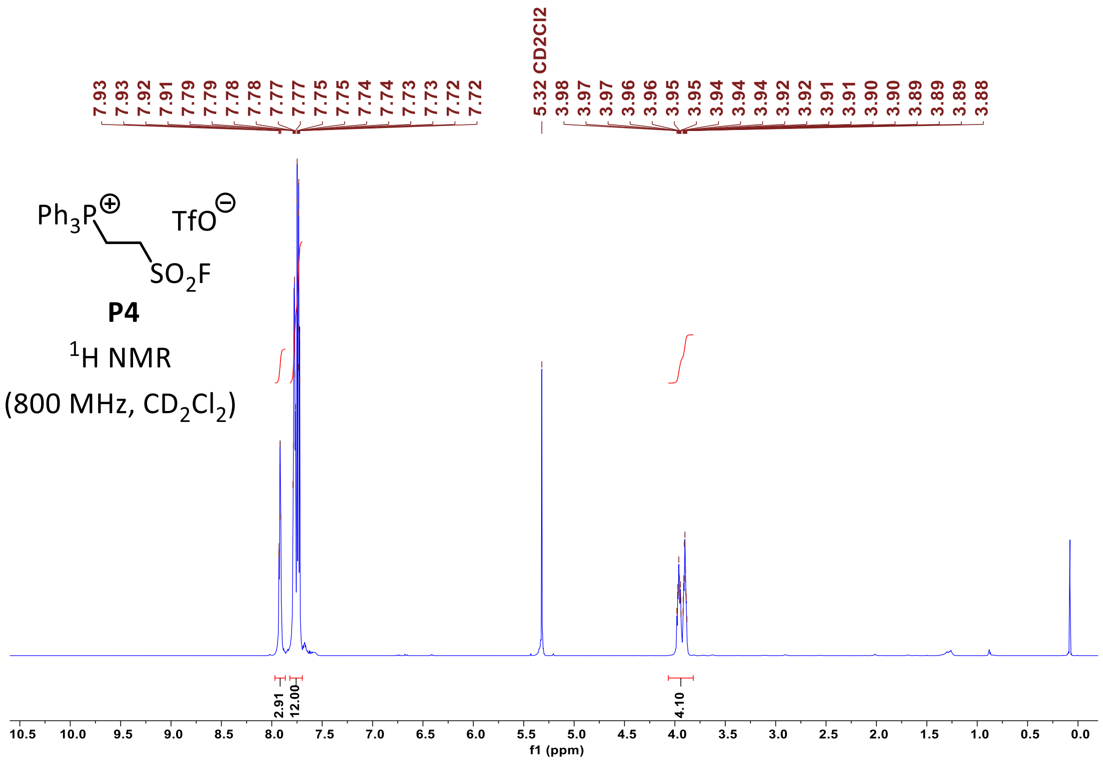

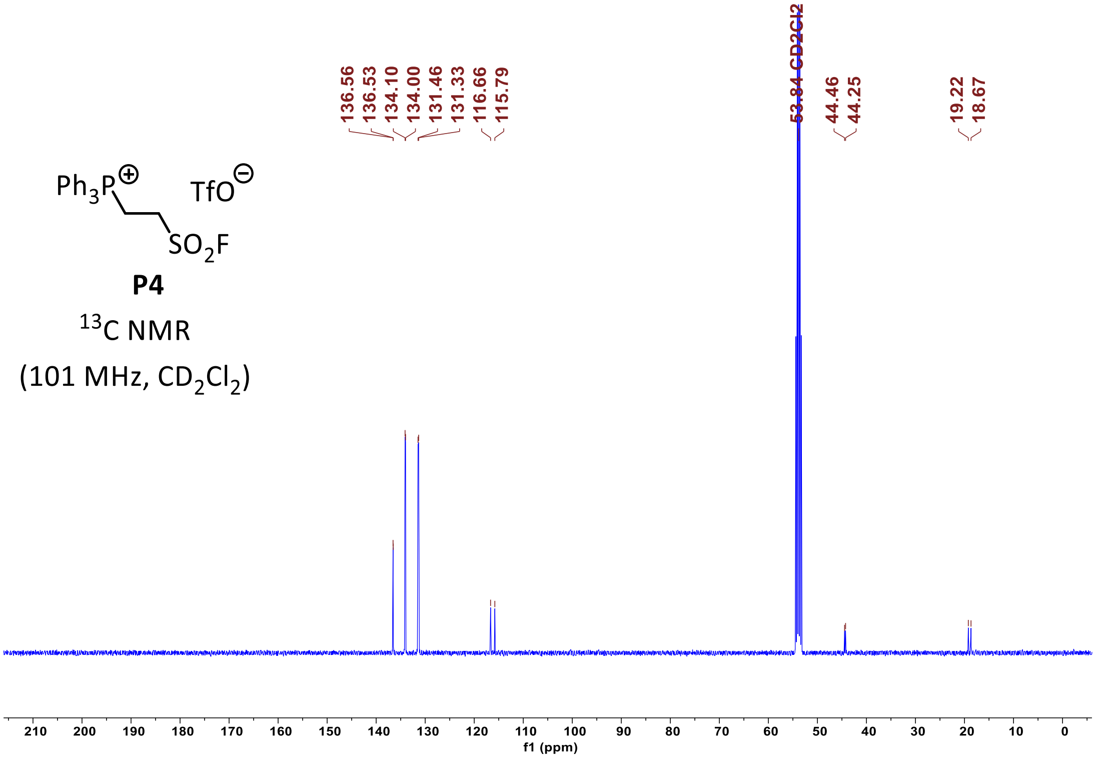
136.56
136.53
134.10
134.00
131.46
131.33
116.66
115.79
53.84 CD2Cl2
44.46
44.25
19.22
18.67
Ph3P
TfO
SO2F
P4
13C NMR
(101 MHz, CD2Cl2)
210 200 190 180 170 160 150 140 130 120 110 100 90 80 70 60 50 40 30 20 10 0
f1 (ppm)

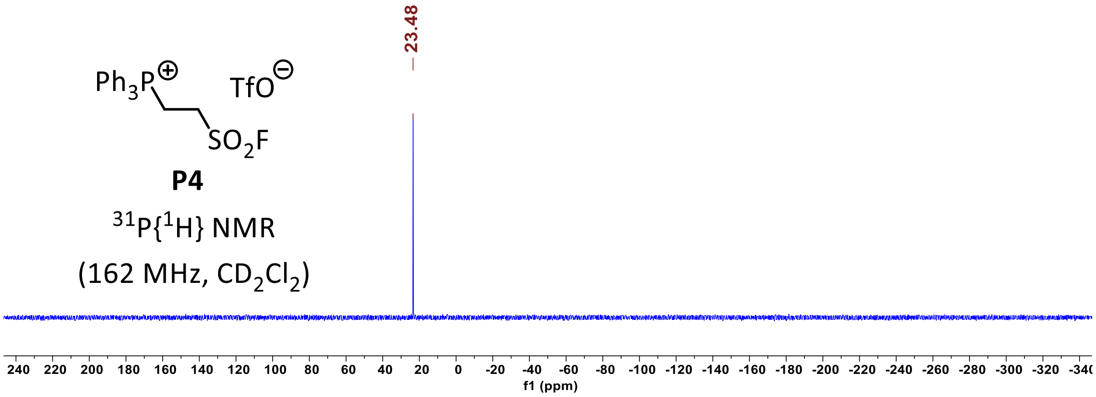
23.48
Ph3P
TfO
SO2F
P4
31P{1H} NMR
(162 MHz, CD2Cl2)
240 220 200 180 160 140 120 100 80 60 40 20 0 -20 -40 -60 -80 -100 -120 -140 -160 -180 -200 -220 -240 -260 -280 -300 -320
f1 (ppm)

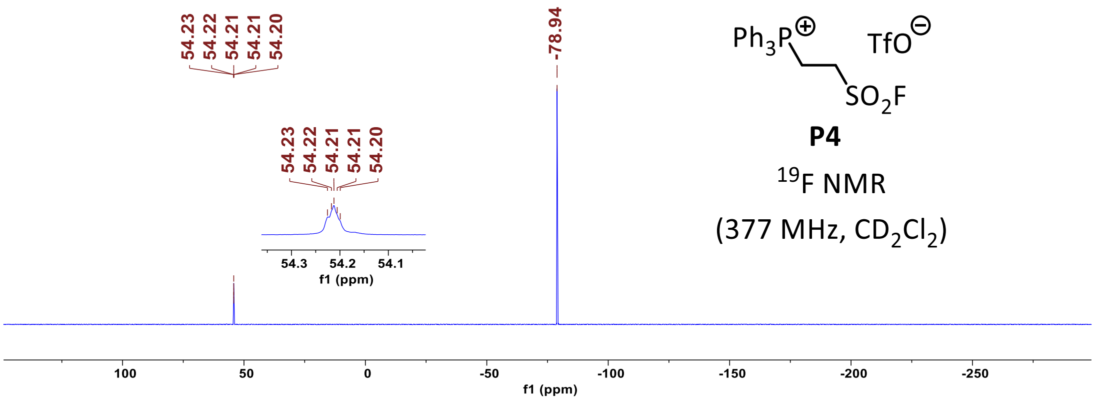
54.23
54.22
54.21
54.21
54.20
-78.94
54.3 54.2 54.1
f1 (ppm)
Ph3P
TfO
SO2F
P4
19F NMR
(377 MHz, CD2Cl2)
100 50 0 -50 -100 -150 -200 -250
f1 (ppm)

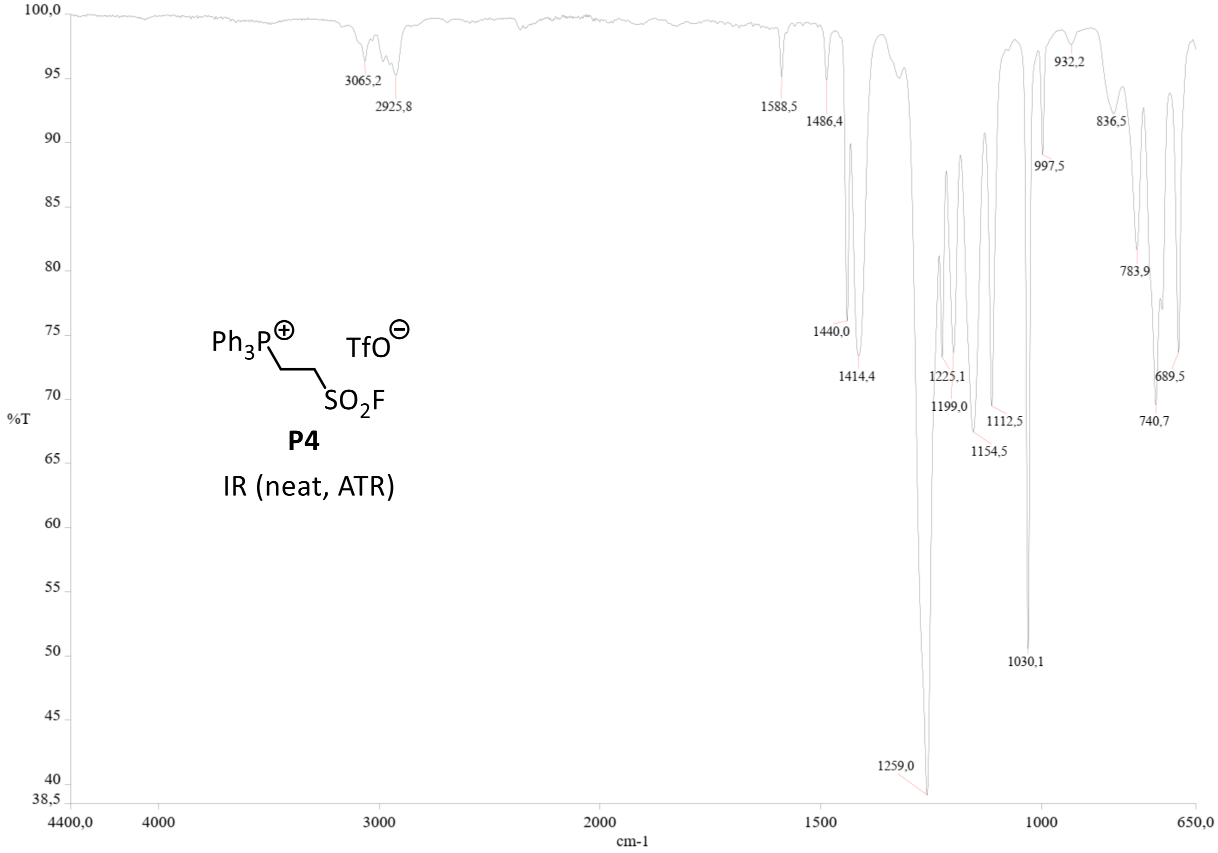
100,0
95
90
85
80
75
70
65
60
55
50
45
40
38,5
%T
4400,0
4000
3000
2000
1500
1000
650,0
cm-1
3065,2
2925,8
1588,5
1486,4
1440,0
1414,4
1259,0
1225,1
1199,0
1154,5
1112,5
1030,1
997,5
932,2
836,5
783,9
740,7
689,5
P4
IR (neat, ATR)

Probenname: C:\Data\12-feanch-f603
Auftraggeber: An, Offial

Probe: 522, c21h19f4o5ps2, fest, Aceton
Methode: 100 ul/min Acetonitril/Wasser, FIA/ESI, LTQ FT, Spahl

Inj Vol: 1.000000
Zeit:

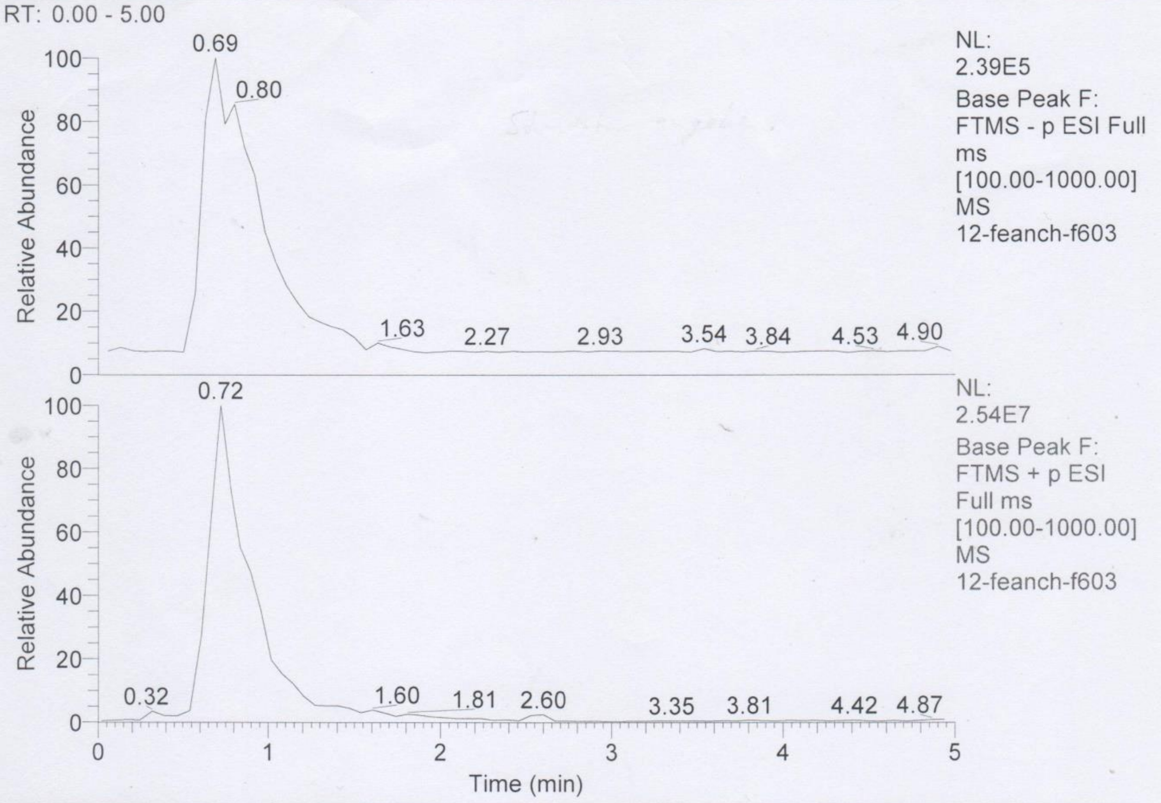

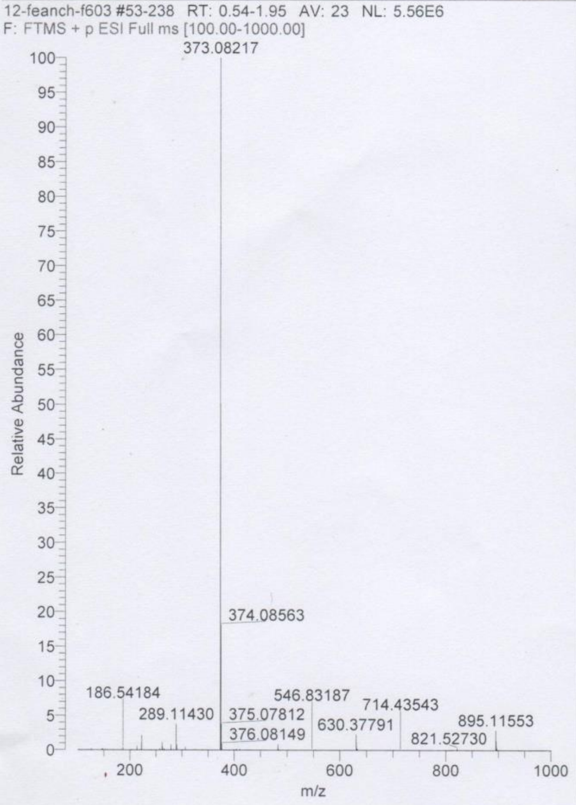

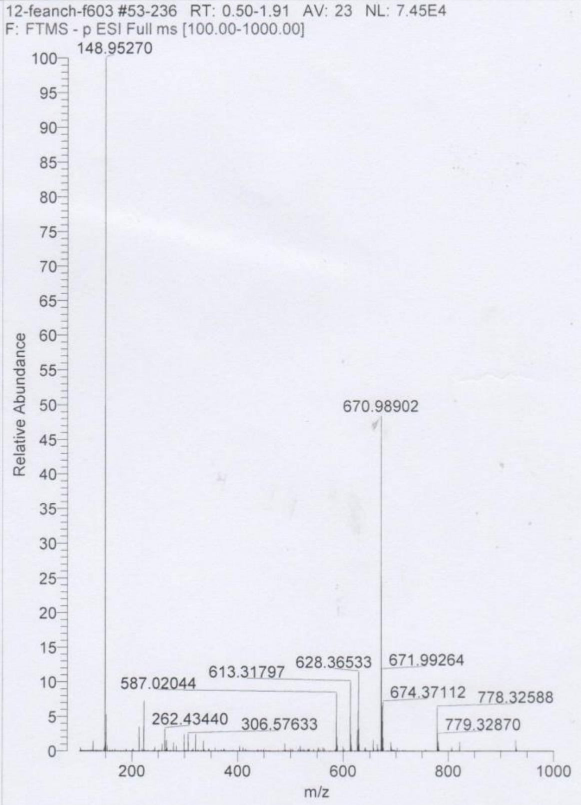

3 Studies on the Interactions Between Electrophiles, Nucleophiles and Proton Sources in CD_2Cl_2

3.1 Brønsted Basicity of PMe_2Ph and $PMePh_2$ vs 2,4,6-Collidine

3.1.1 $PMePh_2$ and 2,4,6-collidinium triflate (CT)

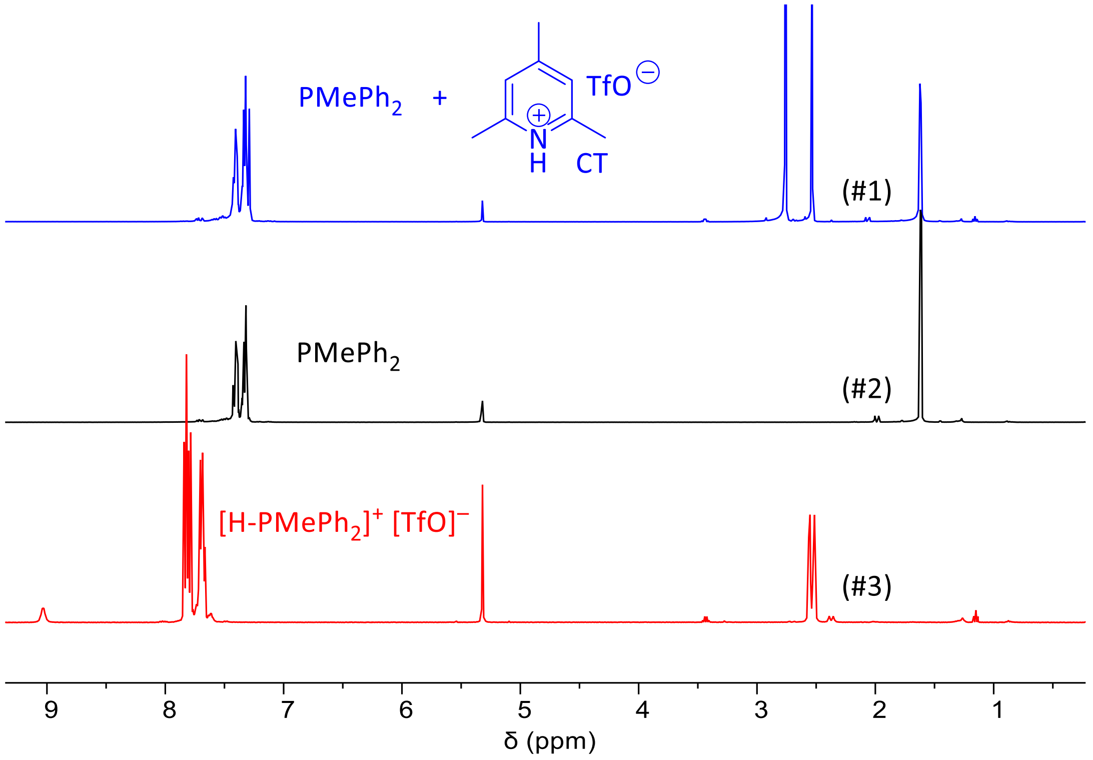

Fig. S3. Comparison of the ^{1}H NMR spectra (400 MHz, CD_2Cl_2) of $PMePh_2$ (#2) and $[H\text{-}PMePh_2]^+$ $[TfO]^-$ (#3) with that of a mixture of $PMePh_2$ and CT (#1).

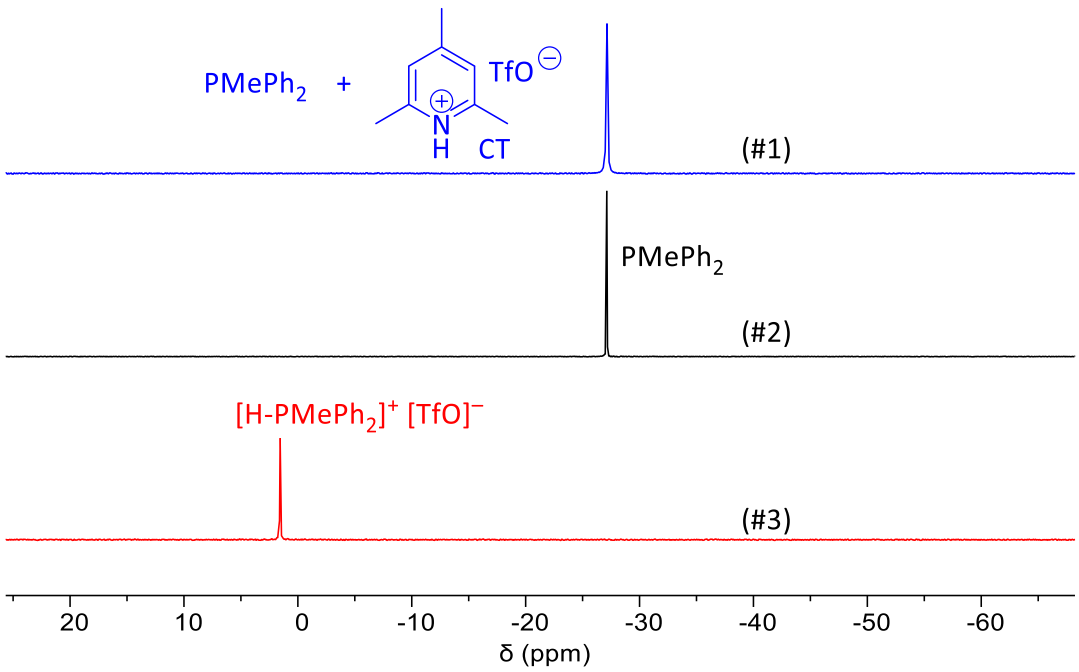

Fig. S4. Comparison of the ^{31}P NMR spectra (162 MHz, CD_2Cl_2) of $PMePh_2$ (#2) and $[H\text{-}PMePh_2]^+$ $[TfO]^-$ (#3) with that of a mixture of $PMePh_2$ and CT (#1).

3.1.2 PMe_2Ph and 2,4,6-collidinium triflate (CT)

Figures S5 and S6 indicate that PMe_2Ph is not protonated by CT in dichloromethane.

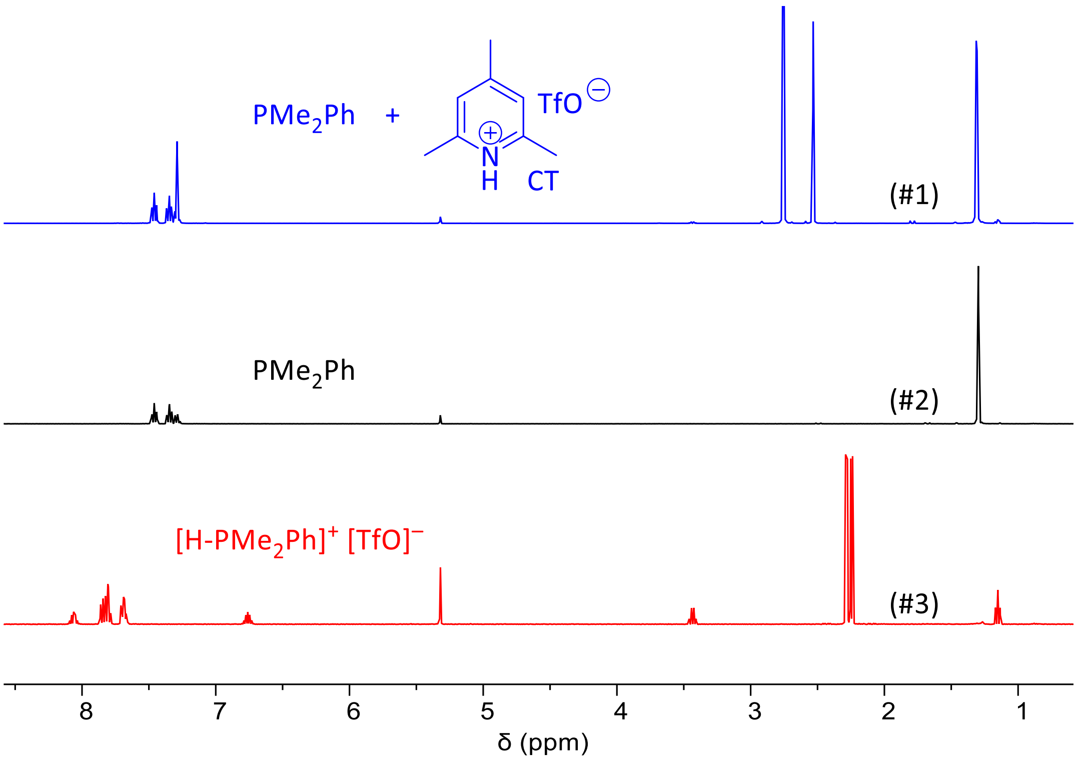

Fig. S5. Comparison of the ^{1}H NMR spectra (400 MHz, CD_2Cl_2) of PMe_2Ph (#2) and $[H\text{-}PMe_2Ph]^+$ $[TfO]^-$ (#3) with that of a mixture of PMe_2Ph and CT (#1).

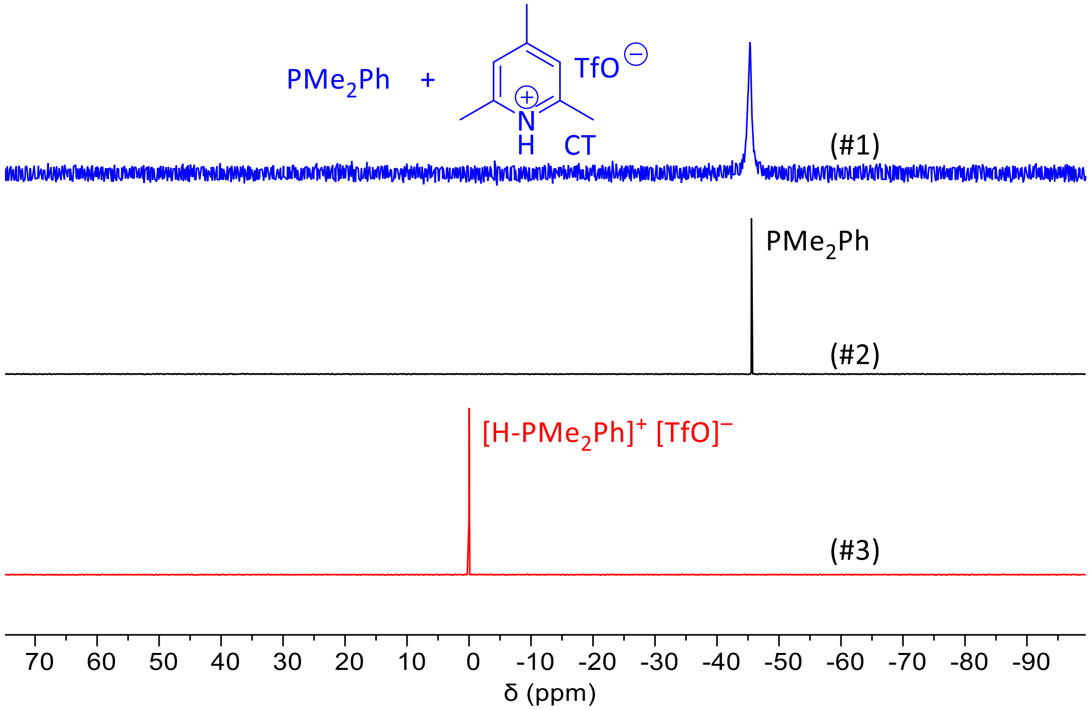

Fig. S6. Comparison of the ^{31}P NMR spectra (162 MHz, CD_2Cl_2) of PMe_2Ph (#2) and $[H\text{-}PMe_2Ph]^+$ $[TfO]^-$ (#3) with that of a mixture of PMe_2Ph and CT (#1).

3.2 Interactions of Proton Sources with Electrophiles

3.2.1 Ethyl acrylate (1) with 2,4,6-collidinium triflate (CT)

Figure S7 indicates that CT does not change the ^{1}H NMR spectrum of ethyl acrylate **1** (in CD_2Cl_2).

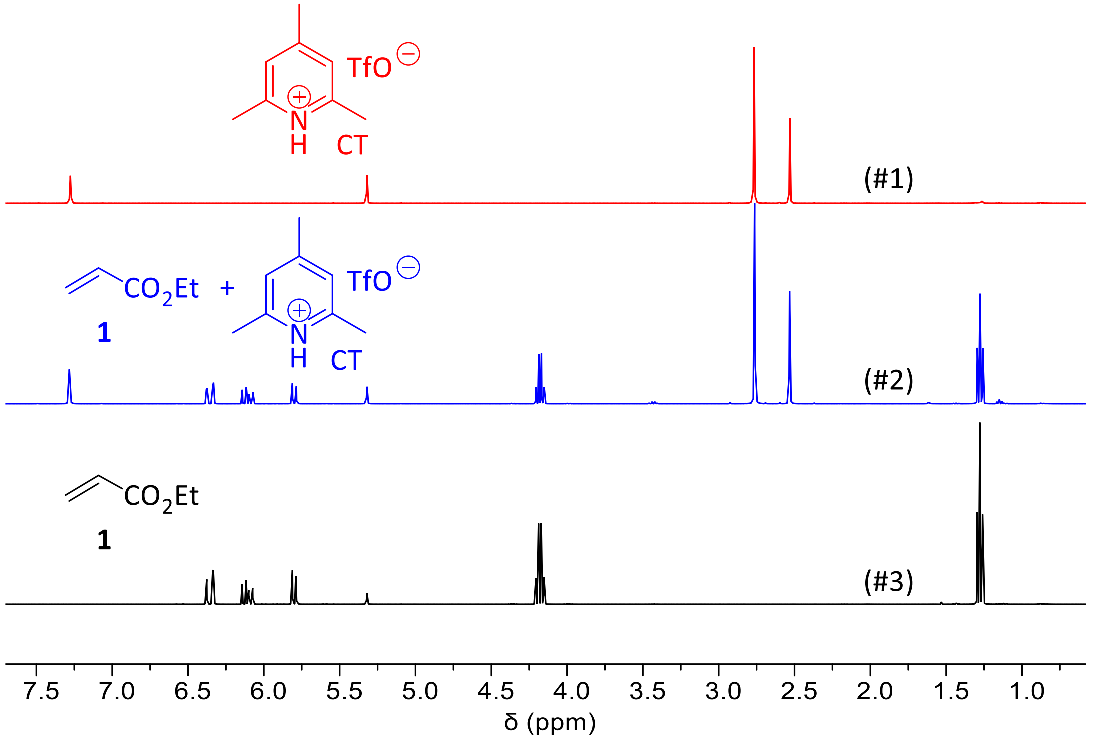

Fig. S7. Comparison of the ^{1}H NMR spectra (400 MHz, CD_2Cl_2) of CT (#1) and **1** (#3) with that of a mixture of CT and **1** (#2).

3.2.2 Ethyl allenoate (2) with CT

Figure S8 shows that CT does not change the ^{1}H NMR spectrum of ethyl allenoate **2** (in CD_2Cl_2).[S6]

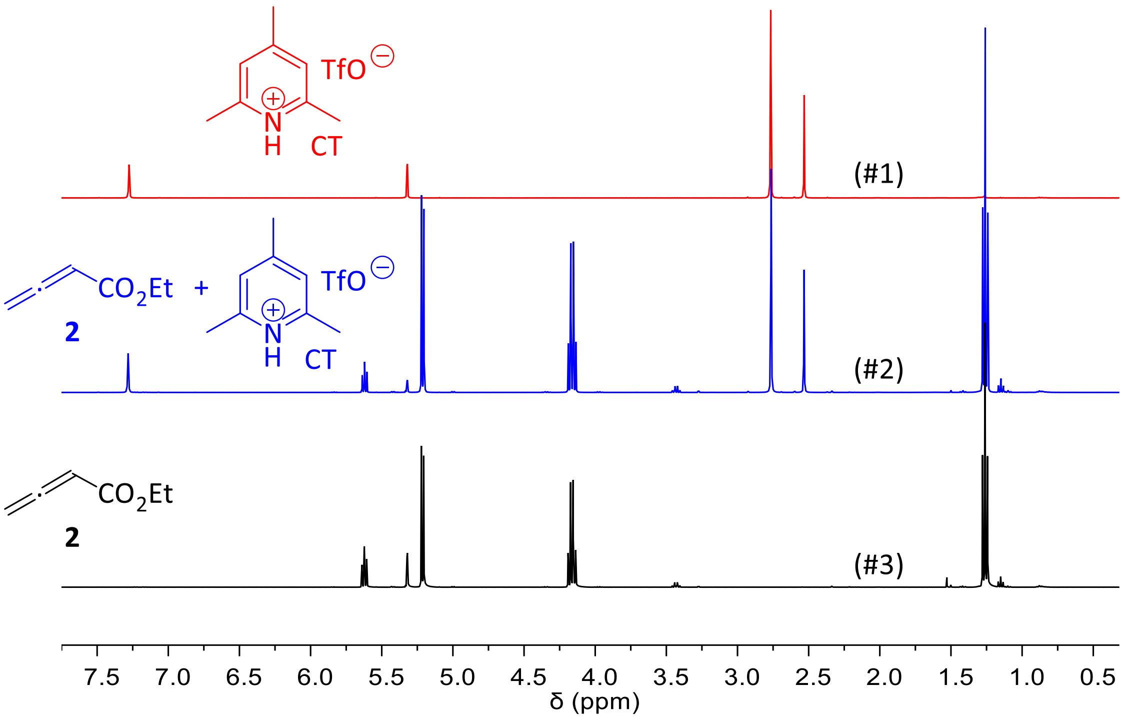

Fig. S8. Comparison of the ^{1}H NMR spectra (400 MHz, CD_2Cl_2) of CT (#1) and **2** (#3) with that of a mixture of CT and **2** in CD_2Cl_2 (#2).

3.2.3 Ethyl propiolate (3) with CT

Figure S9 indicates that CT does not change the ^{1}H NMR spectrum of ethyl propiolate **3** (in CD_2Cl_2).

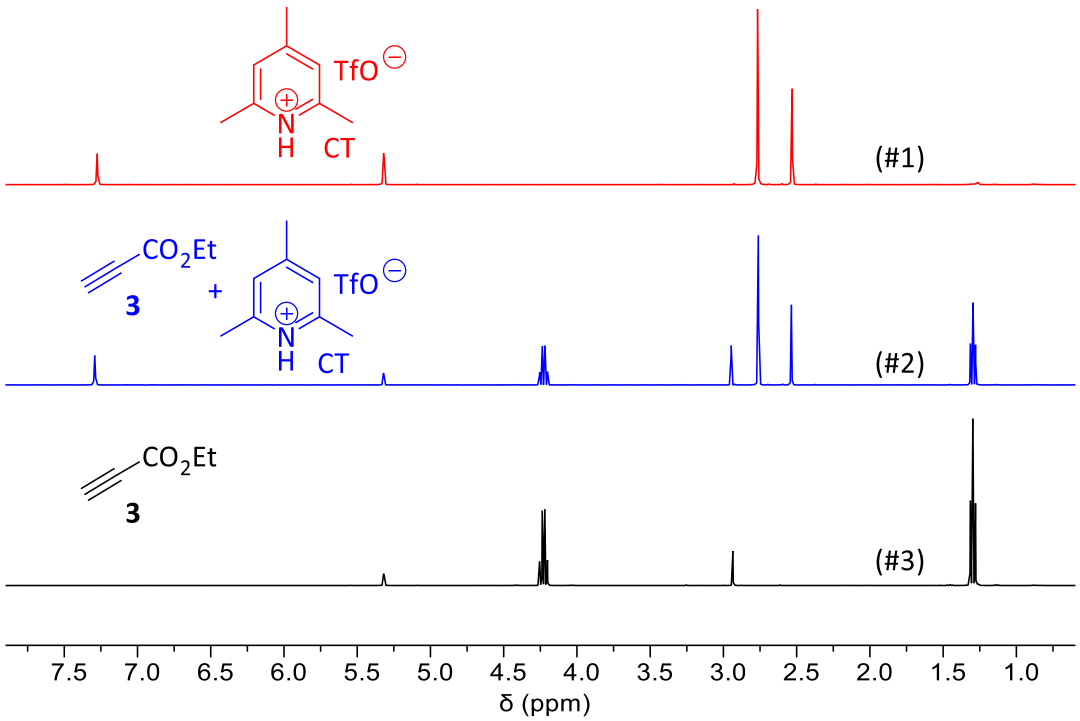

Fig. S9. Comparison of the ^{1}H NMR spectra (400 MHz, CD_2Cl_2) of CT (#1) and **3** (#3) with that of a mixture of **3** and CT (#2).

3.2.5 Ethyl acrylate (1) with tributylphosphonium triflate (TBPT)

Figure S10 shows that TBPT does not change the ^{1}H NMR spectrum of ethyl acrylate **1** (in CD_2Cl_2).

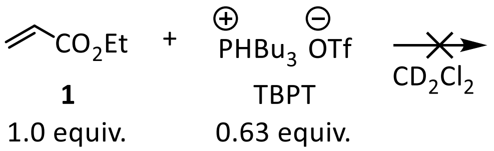

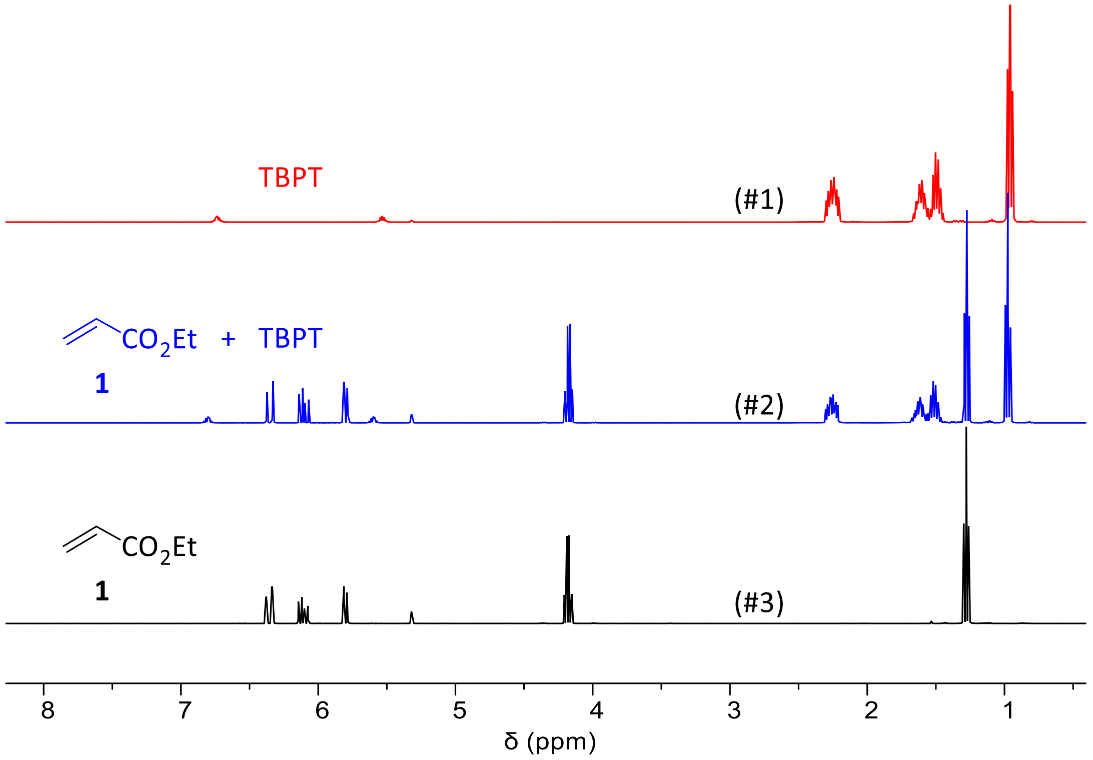

Fig. S10. Comparison of the ^{1}H NMR spectra (400 MHz, CD_2Cl_2) of TBPT (#1) and **1** (#3) with that of a mixture of **1** and TBPT (#2).

3.3 Stability of the Electrophiles 2 and 5 in the Presence of Triethylamine

3.3.1 Ethyl 2,3-butadienoate (2) + trimethylamine (TEA)

Ethyl-2,3-butadieneoate (**2**) does not tautomerise (within 16 h) to ethyl 2-butynoate (**5**) when mixed with TEA in dichloromethane (Figure S11).

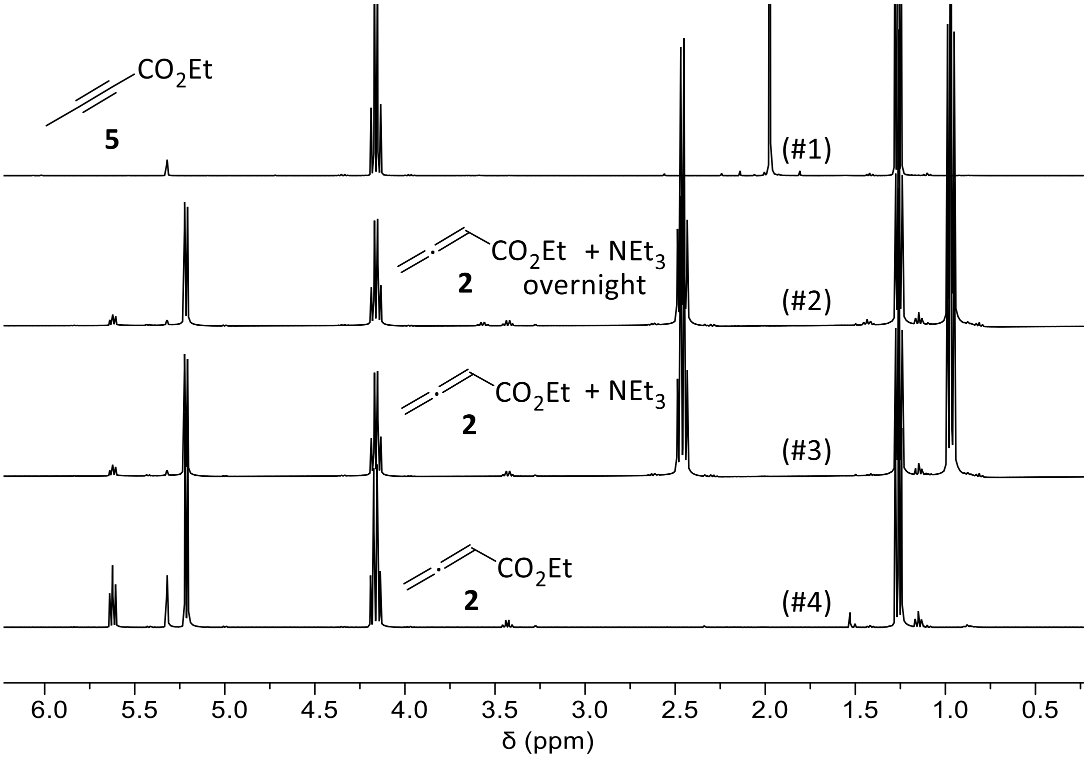

Fig. S11. Comparison of the ^1H NMR spectra (400 MHz, CD_2Cl_2) of **5** (#1) and **2** (#4) with those of a mixture of **2** with triethylamine (after mixing: #3, overnight: #2).

3.3.2 Ethyl 2-butynoate (5) + trimethylamine (TEA)

Ethyl 2-butynoate (**5**) does not tautomerise (within 16 h) to ethyl-2,3-butadieneoate (**2**) when mixed with TEA in dichloromethane (Figure S12).

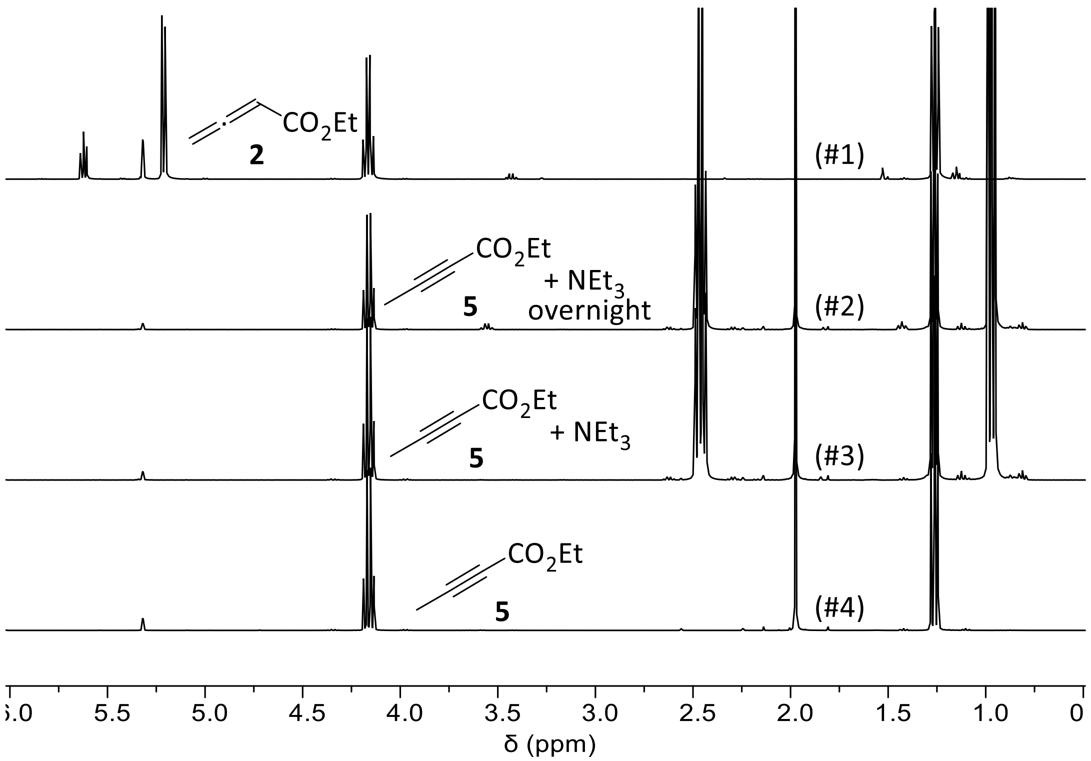

Fig. S12. Comparison of the ^{1}H NMR spectra (400 MHz, CD_2Cl_2) of **2** (#1) and **5** (#4) with those of a mixture of **5** with triethylamine (after mixing: #3, overnight: #2).

3.4 NMR Studies of Mixtures of TEAT with the Electrophiles 1–3

3.4.1 Ethyl acrylate (1) with triethylammonium triflate (TEAT)

Figure S13 indicates that TEAT does not change the ^1H NMR spectrum of ethyl acrylate (**1**) in CD_2Cl_2.

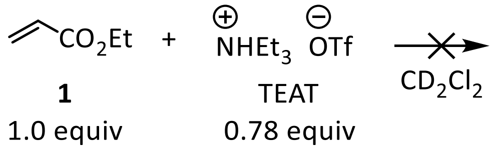

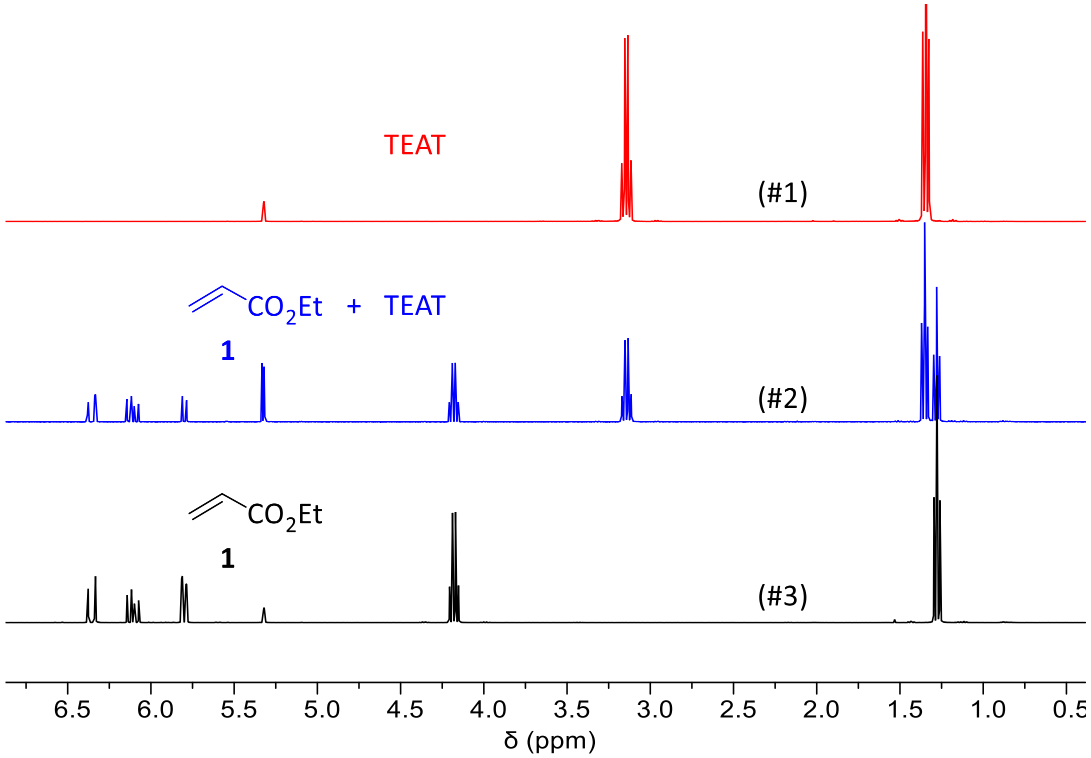

Fig. S13. Comparison of the ^1H NMR spectra (400 MHz, CD_2Cl_2) of TEAT (#1) and **1** (#3) with that of a mixture of **1** and TEAT (#2).

3.4.2 Ethyl 2,3-butadienoate (2) with triethylammonium triflate (TEAT)

Ethyl 2,3-butadieneoate (**2**) does not tautomerise to ethyl 2-butynoate (**5**) when mixed with TEAT in dichloromethane. Mixtures of **2** and TEAT show identical ^{1}H NMR chemical shifts that agree with the NMR resonances of the individual compounds and excludes significant interactions between TEAT and the electrophile **2**. Only after long reaction times (overnight) additional resonances of potential reaction products become detectable (Figure S14).

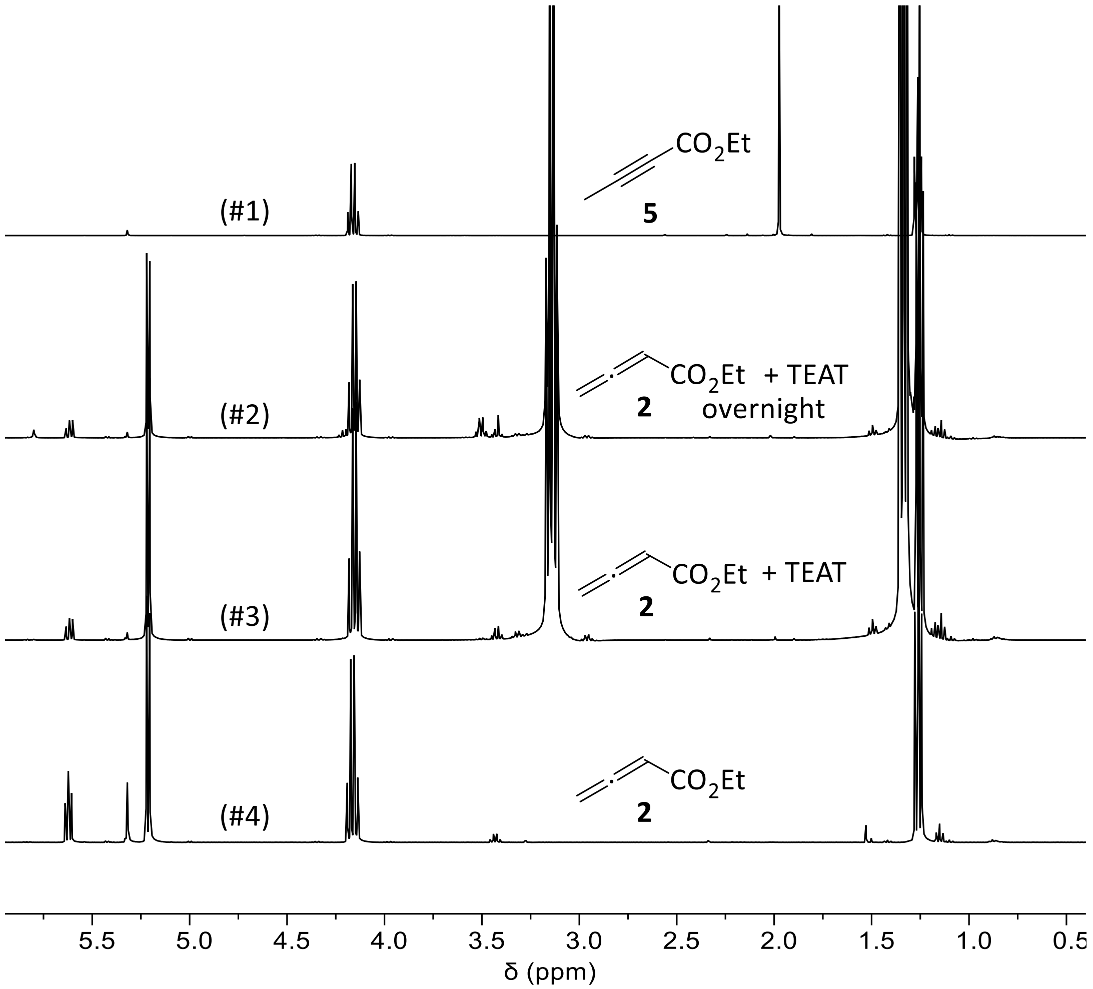

Fig. S14. Comparison of the ^{1}H NMR spectra (400 MHz, CD_2Cl_2) of **5** (#1) and its tautomer **2** (#4) with those of a mixture of **2** with TEAT (after mixing: #3, overnight: #2).

3.4.3 Ethyl 2-butynoate (5) with triethylammonium triflate (TEAT)

Ethyl 2-butynoate (**5**) does not tautomerise to ethyl-2,3-butadieneoate (**2**) when mixed with trimethylammonium triflate in dichloromethane (Figure S15).

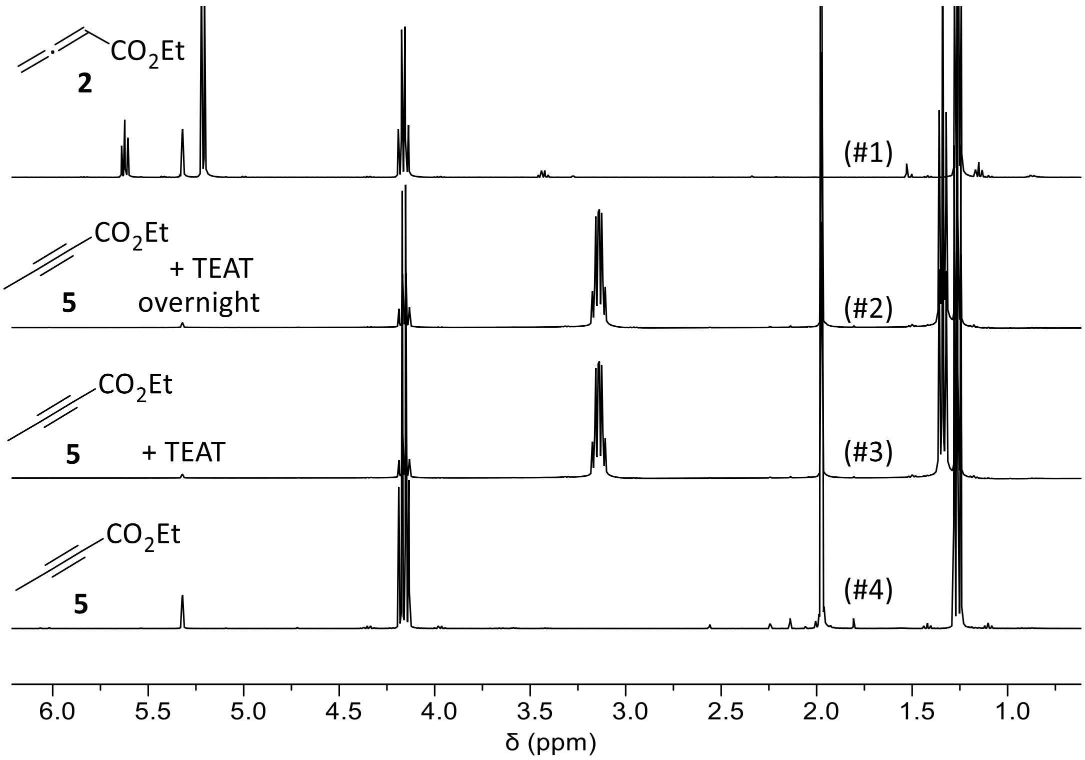

Fig. S15. Comparison of the ^{1}H NMR spectra (400 MHz, CD_2Cl_2) of **2** (#1) and its tautomer **5** (#4) with those of a mixture of **5** and TEAT (after mixing: #3, overnight: #2).

3.4.4 In situ generation of trimethylphosphine PMe_3 in CD_2Cl_2

The ^{1}H, ^{13}C, and ^{31}P NMR spectra in Figure S16 show that NEt_3 is a stronger Brønsted base than PMe_3 (in CD_2Cl_2). Thus, a quantitative amount of PMe_3 is liberated when $[HPMe_3]^+$ $[BF_4]^-$ is mixed with NEt_3. The NMR spectroscopic data for resonances assigned to PMe_3 (in the mixtures with triethylammonium tetrafluoroborate) agree with those reported for pure PMe_3.[S9]

$$^{\oplus}PHMe_3\ BF_4^{\ominus} + NEt_3 \xrightarrow{CD_2Cl_2} PMe_3 + {}^{\oplus}NHEt_3\ BF_4^{\ominus} \qquad \text{(f406)}$$

For PMe_3:

^{1}H NMR (400 MHz, CD_2Cl_2) δ 0.99–0.98 (m, 9 H). **^{13}C{^{1}H} NMR** (101 MHz, CD_2Cl_2) δ 16.4 (d, *J* = 11.5 Hz). **^{31}P NMR** (162 MHz, CD_2Cl_2) δ −61.5.

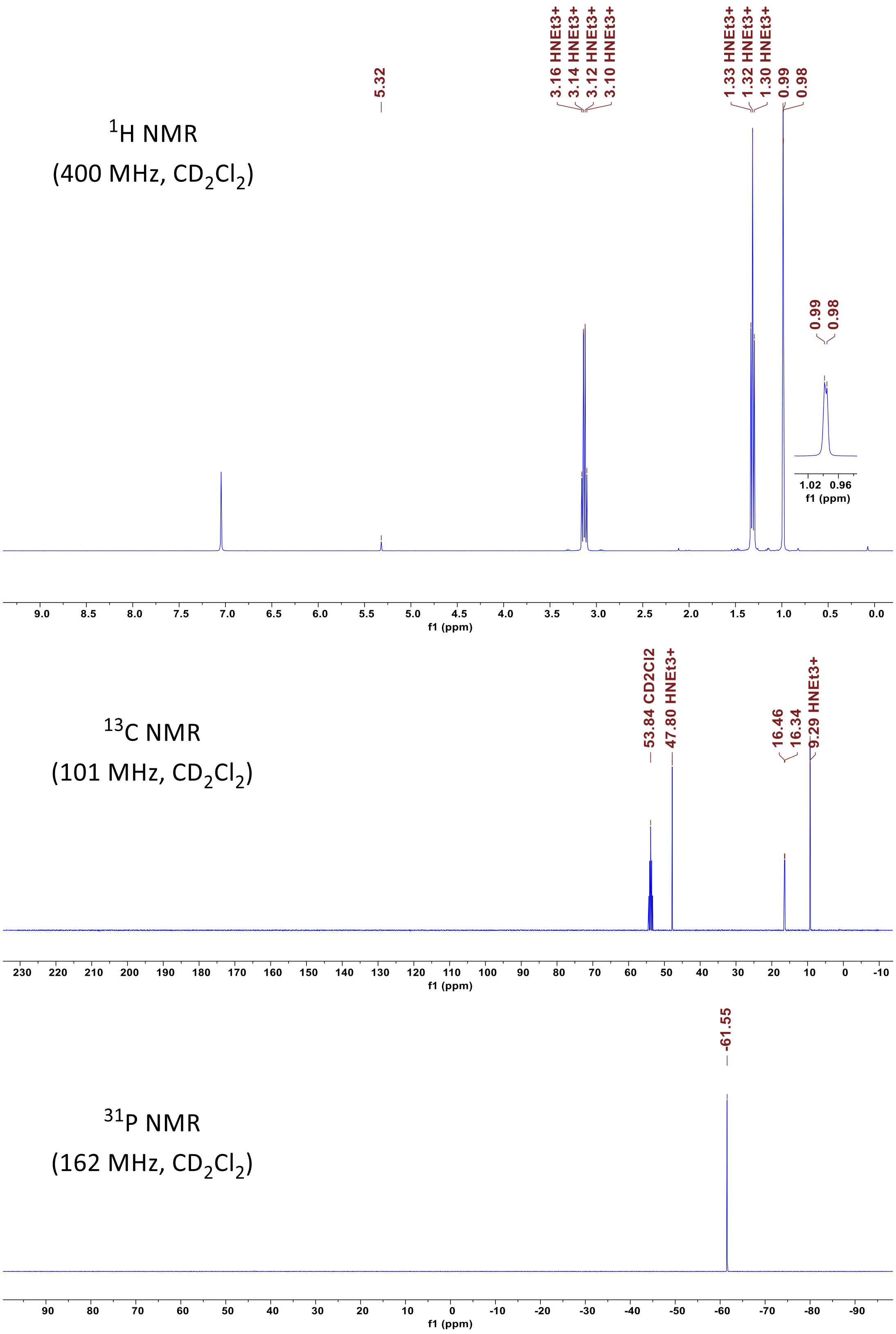

Fig. S16. ^{1}H, ^{13}C, and ^{31}P NMR spectra of a mixture of $[HPMe_3]^+$ $[BF_4]^-$ with NEt_3 (1.0 equiv) in CD_2Cl_2.

3.4.5 Generation of tricyclohexylphosphine PCy_3 in CD_2Cl_2

The ^{31}P NMR spectrum in Figure S17 shows that NEt_3 is a stronger Brønsted base than PCy_3 (in CD_2Cl_2). Thus, a quantitative amount of PCy_3 is liberated when $[HPCy_3]^+$ $[TfO]^-$ is mixed with NEt_3. The NMR spectroscopic data for the ^{31}P resonance assigned to PCy_3 (in a mixture with TEAT) agrees with that reported for pure PCy_3.[S10]

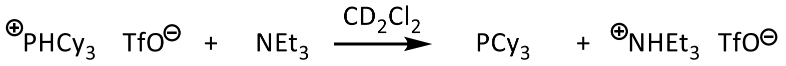

(f417)

For PCy_3: **^{31}P NMR** (162 MHz, CD_2Cl_2) δ 10.5.

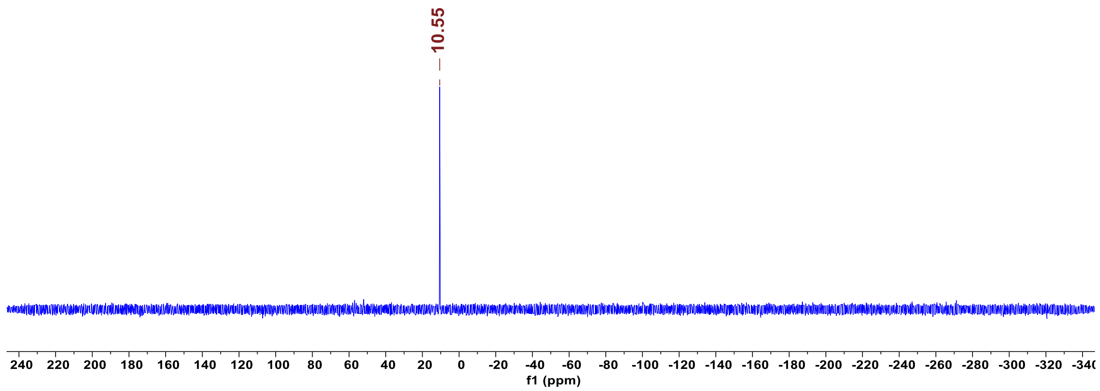

Fig. S17. ^{31}P NMR spectrum (162 MHz) of a mixture of $[HPCy_3]^+$ $[TfO]^-$ with NEt_3 (1 equiv) in CD_2Cl_2.

3.4.6 Generation of trioctylphosphine $POct_3$ in CD_2Cl_2

The ^{31}P NMR spectrum in Figure S18 shows that NEt_3 is a stronger Brønsted base than $POct_3$ (in CD_2Cl_2). Thus, a quantitative amount of $POct_3$ is liberated when $[HPOct_3]^+$ $[TfO]^-$ is mixed with NEt_3. The NMR spectroscopic data for the ^{31}P resonance assigned to $POct_3$ (in a mixture with TEAT) agrees with that reported for pure $POct_3$.[S11]

$$^{\oplus}PHOct_3\ TfO^{\ominus} + NEt_3 \xrightarrow{CD_2Cl_2} POct_3 + {}^{\oplus}NHEt_3\ TfO^{\ominus} \qquad \text{(f418)}$$

For $POct_3$: **^{31}P NMR** (162 MHz, CD_2Cl_2) δ −31.3 (t, *J* = 6.6 Hz)

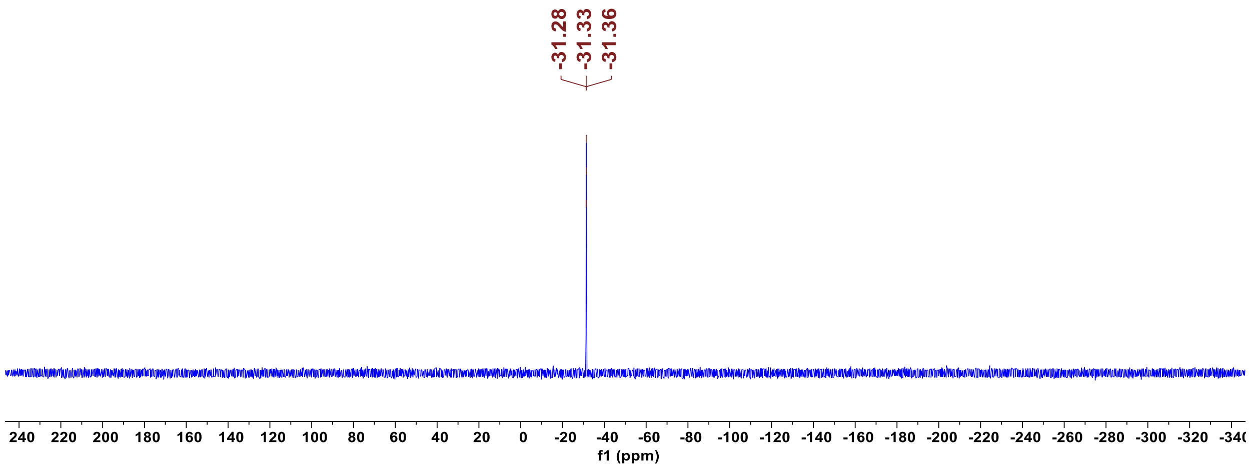

Fig. S18. ^{31}P NMR spectrum (162 MHz) of a mixture of $[HPCy_3]^+$ $[TfO]^-$ with NEt_3 (1.0 equiv) in CD_2Cl_2.

3.5 Deprotonation of Phosphonium Salts P1 and P2

3.5.1 Deprotonation of P1 with KO*t*Bu in d_6-DMSO

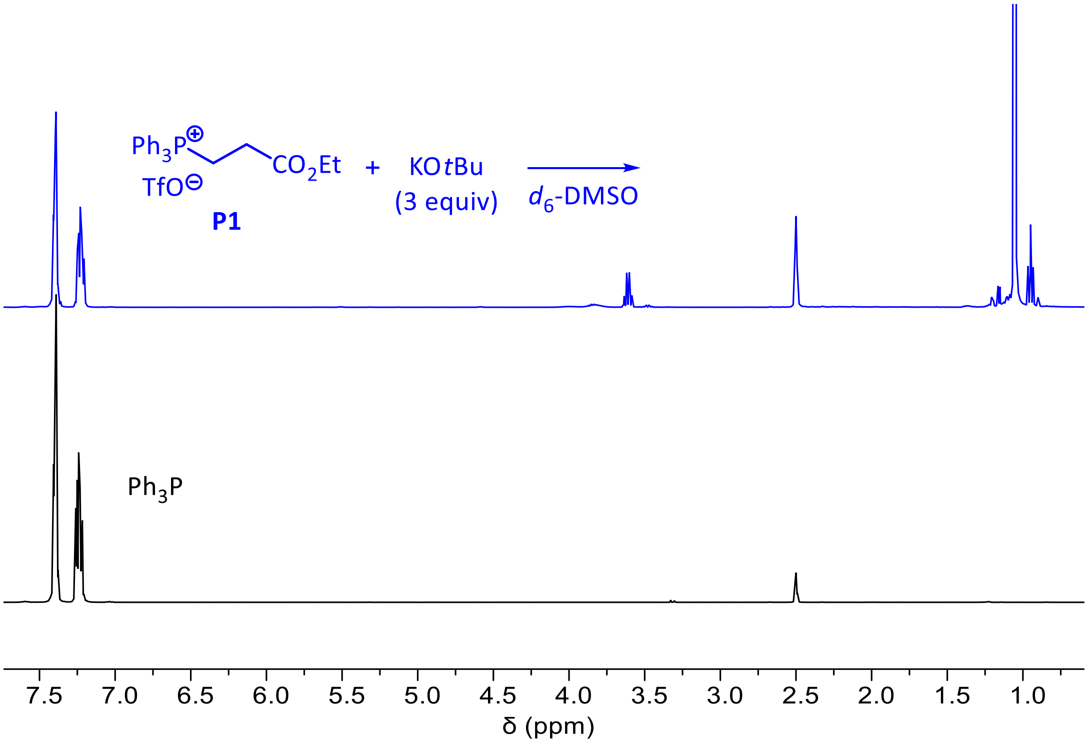

Fig. S19. Comparison of the ^{1}H NMR spectrum (400 MHz, d_6-DMSO) of PPh_3 (bottom, black) with that of a **P1**/KO*t*Bu mixture (top, blue).

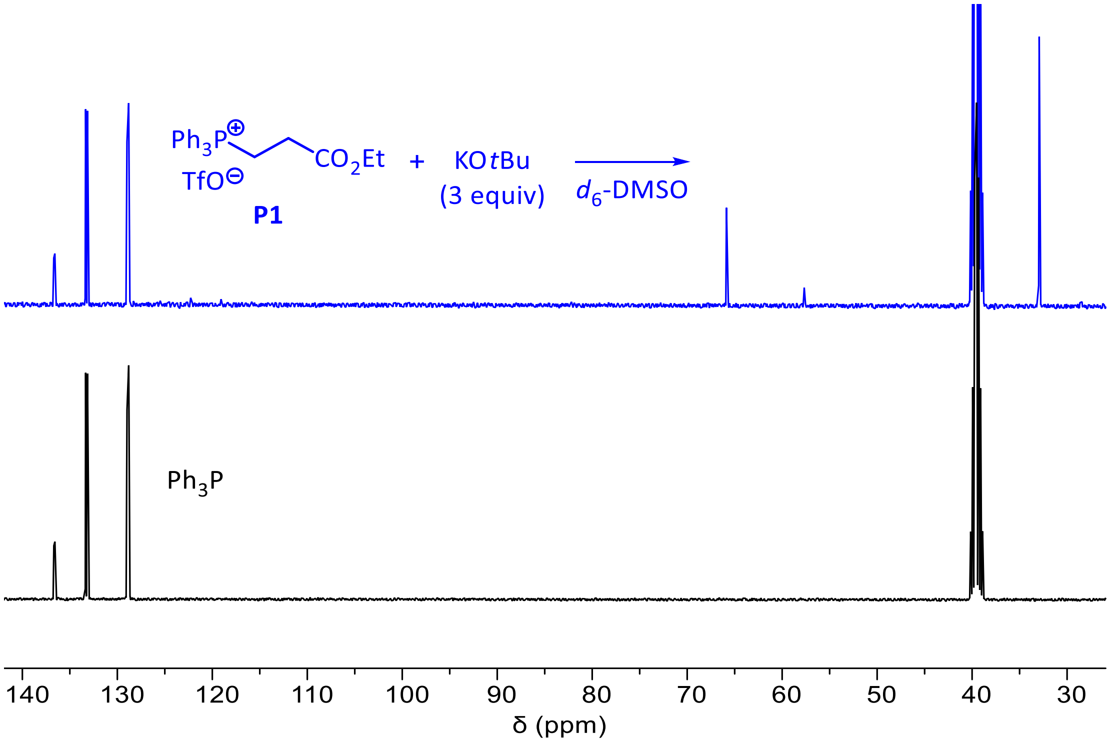

Fig. S20. Comparison of the ^{13}C NMR spectrum (101 MHz, d_6-DMSO) of PPh_3 (bottom, black) with that of a **P1**/KO*t*Bu mixture (top, blue).

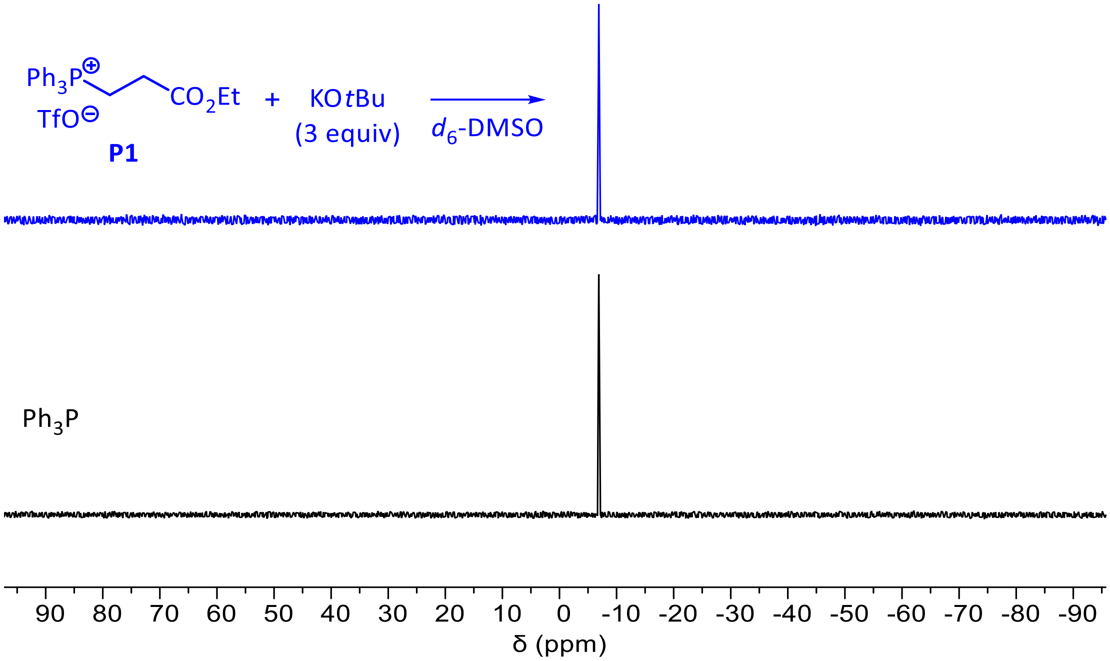

Fig. S21. Comparison of the ^{31}P NMR spectrum (162 MHz, d_6-DMSO) of PPh_3 (bottom, black) with that of a **P1**/KO*t*Bu mixture (top, blue).

3.5.2 Deprotonation of P2 with KO*t*Bu in *d*$_6$-DMSO

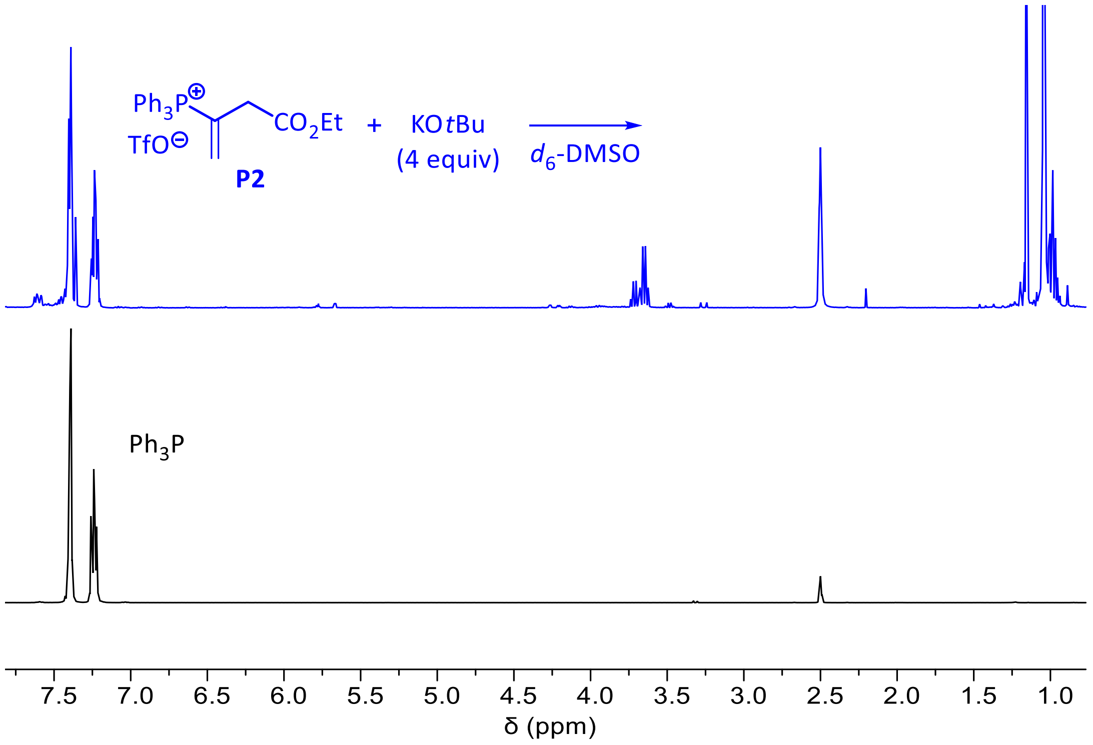

Fig. S22. Comparison of the ^{1}H NMR spectrum (400 MHz, *d*$_6$-DMSO) of PPh_3 (bottom, black) with that of a **P2**/KO*t*Bu mixture (top, blue).

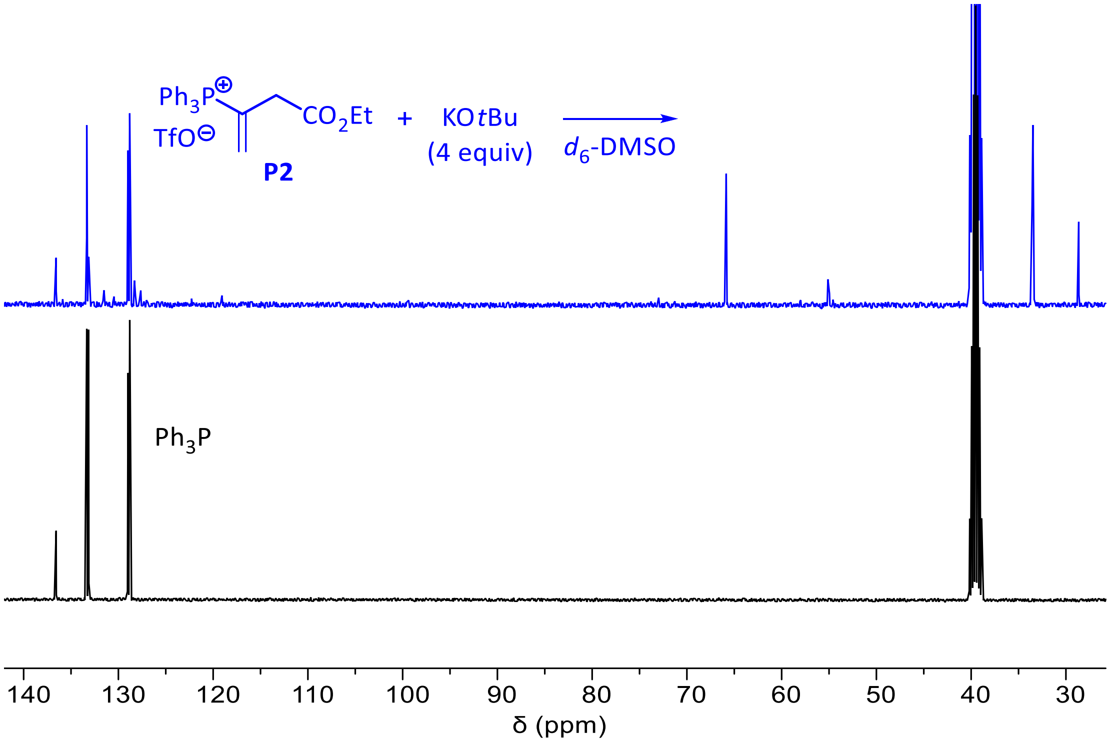

Fig. S23. Comparison of the ^{13}C NMR spectrum (101 MHz, d_6-DMSO) of PPh_3 (bottom, black) with that of a **P2**/KO*t*Bu mixture (top, blue).

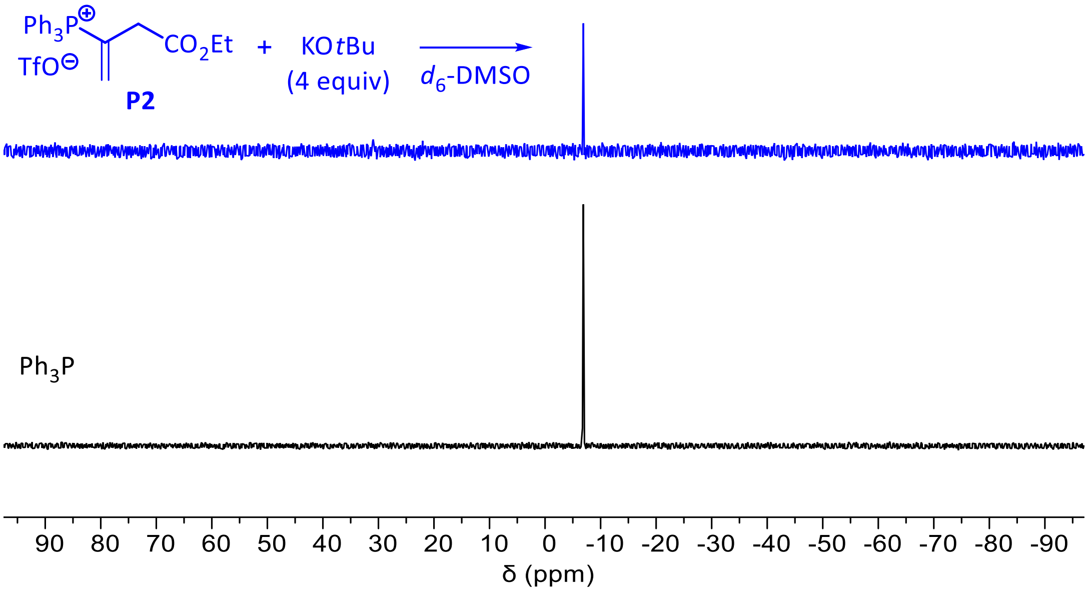

Fig. S24. Comparison of the ^{31}P NMR spectrum (162 MHz, d_6-DMSO) of PPh_3 (bottom, black) with that of a **P2**/KO*t*Bu mixture (top, blue).

4 UV-Vis Absorption Spectra in Dichloromethane

4.1 General

UV-Vis absorption spectra were determined by using J&M TIDAS diode array spectrophotometers controlled by Labcontrol Spectacle or TidasDAQ 3.8.1 software and connected to a Hellma 661.502-QX quartz Suprasil immersion probe (5 mm light path) via fibre optic cables and standard SMA connectors. The temperature of the solutions during the measurements was maintained to 20 ± 0.2 °C by using circulating bath cryostats.

Anhydrous dichloromethane was freshly distilled over calcium hydride under an atmosphere of dry nitrogen.

4.2 UV-Vis Absorption Spectra of Phosphines PR_3

Table S2. UV-vis absorption spectra of phosphines PR_3 in dichloromethane.

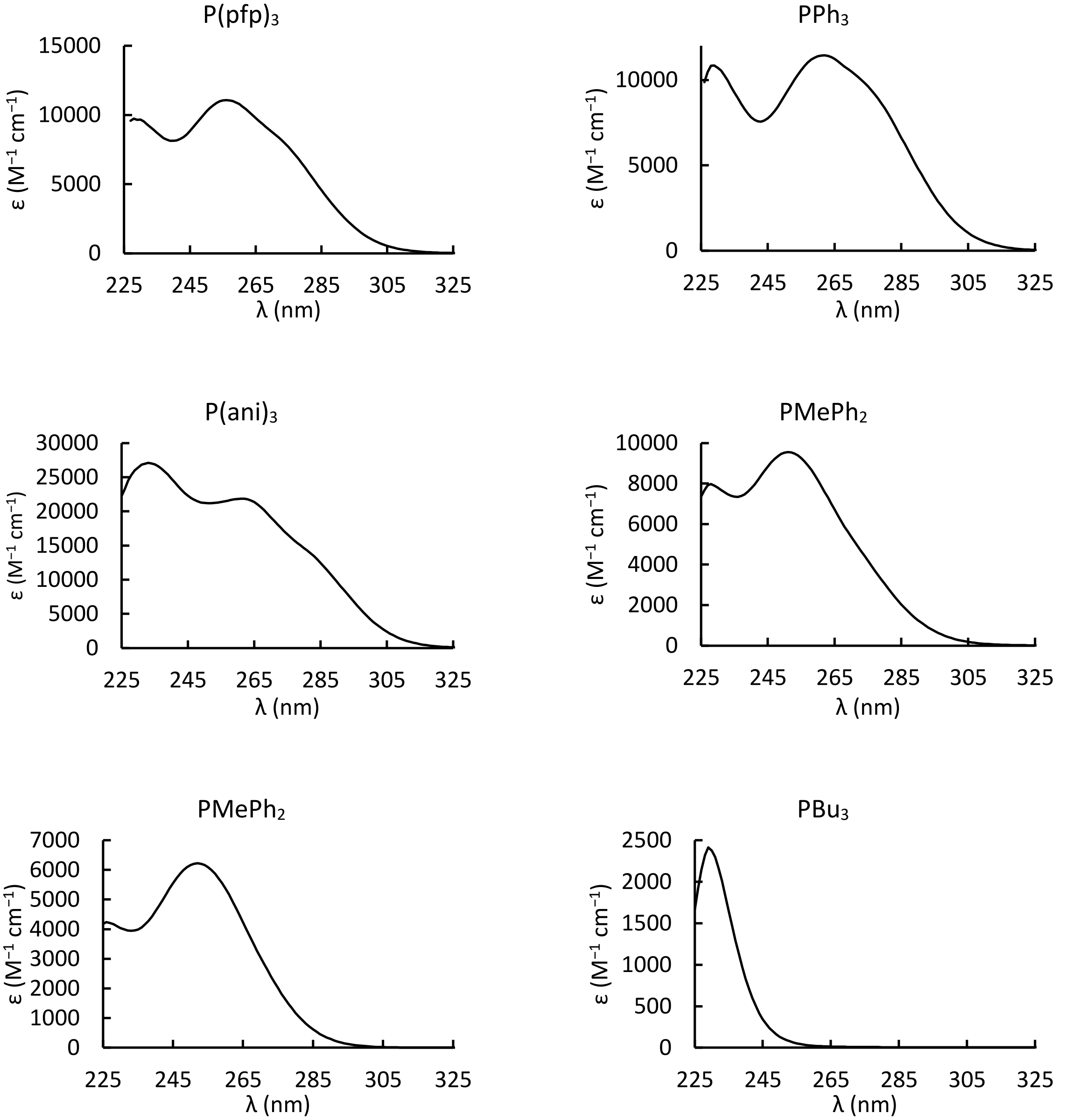

Trialkylphosphonium triflates $[HPR_3]^+[TfO]^-$ with R = Me, Bu, Oct, or Cy have $\varepsilon < 20$ M^{-1} cm^{-1} at λ > 225 nm (in dichloromethane).

4.3 UV-Vis Absorption Spectra of Triethylamine, TEAT, 2,4,6-Collidine and CT

Table S3. UV-Vis absorption spectra of NEt_3, TEAT, 2,4,6-collidine and CT in dichloromethane.

NEt_3

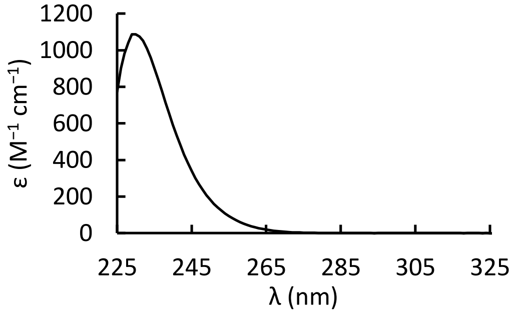

TEAT

(not depicted)

$\varepsilon < 10\ M^{-1}\ cm^{-1}$ at $\lambda > 225$ nm

2,4,6-collidine

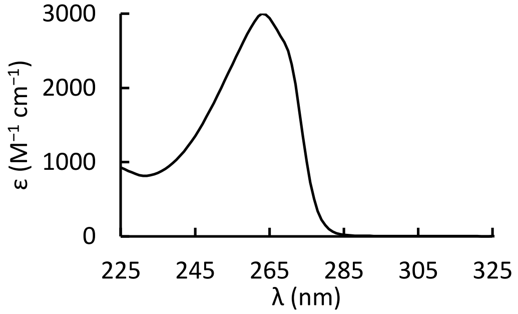

CT

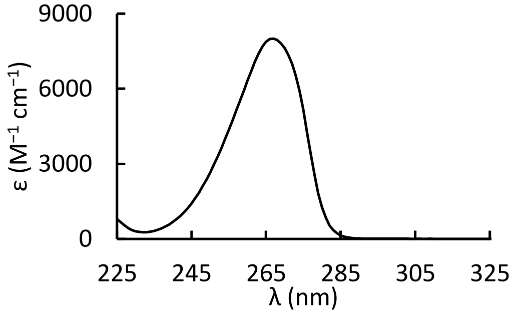

4.4 UV-Vis Absorption Spectra of Electrophiles

Table S4. UV-Vis absorption spectra of electrophiles in dichloromethane.

Ethyl acrylate (**1**) 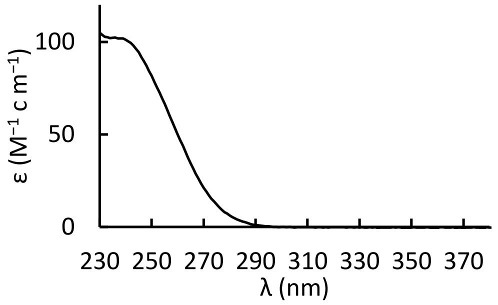	Ethyl butadienoate (**2**) 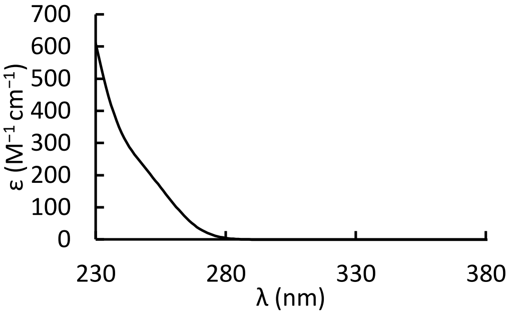
Ethyl propiolate (**3**) 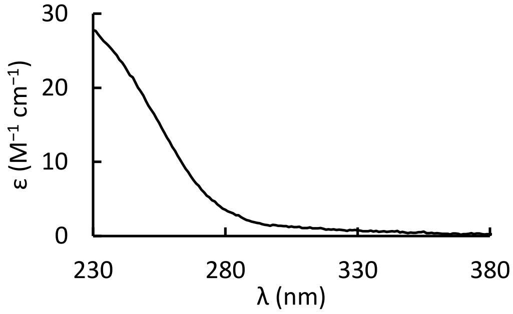	ESF (**4**)<br>(not depicted)<br>ε < 10 M^{-1} cm^{-1} at λ > 225 nm
Ethyl butynoate (**5**)<br>Not determined<br>(reactions of **5** were only studied by NMR kinetic methods)	Benzylidenemalononitrile (BMN) 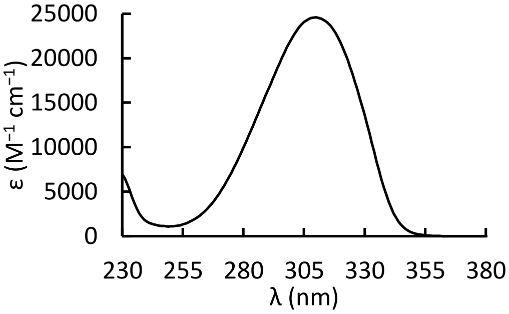

5 Kinetic Measurements

5.1 General

Kinetic Measurements with the Stopped-Flow UV-Vis Spectrometer

The kinetics of fast reactions ($t_{1/2}$ < 40 s) were followed by UV/Vis spectroscopy by using a stopped-flow spectrophotometer system (Applied Photophysics SX20 stopped-flow spectrometer; 5 or 10 mm light path). Stock solutions were prepared in anhydrous dichloromethane, which was freshly distilled from calcium hydride. The kinetic runs were initiated by mixing equal volumes of dichloromethane solutions of the nucleophiles and the electrophiles. The temperature of the solutions during the kinetic studies was maintained to 20 ± 0.2 °C by using circulating bath cryostats.

Kinetic Measurements with Conventional UV-Vis Spectroscopy

The rates of slow reactions ($t_{1/2}$ > 40 s) were determined by using J&M TIDAS diode array spectrophotometers controlled by Labcontrol Spectacle or TidasDAQ 3.8.1 software and connected to a Hellma 661.502-QX quartz Suprasil immersion probe (5 mm light path) via fibre optic cables and standard SMA connectors. The temperature of the solutions during the kinetic studies was maintained to (20 ± 0.1)°C by using circulating bath cryostats. Anhydrous dichloromethane was freshly distilled from calcium hydride under an atmosphere of dry nitrogen.

Kinetic Measurements with NMR Spectroscopy

The kinetics for phosphine-electrophile reactions with the rate constants $k_2 < 4.0 \times 10^{-2}$ M^{-1} s^{-1} were followed by using a Bruker Avance III HD 400 MHz NMR spectrometer, which was equipped with a N_2 cooling unit (20 ± 0.1 °C). The exact 1.0 mL solutions in NMR tubes were prepared freshly under dry argon atmosphere (glove box). The kinetics were measured immediately after complete mixing. Triphenylphosphine oxide ($Ph_3P{=}O$) or triethylphosphine oxide ($Et_3P{=}O$) were used as internal integration standard (IIS) for ^{31}P NMR spectra. For reaction kinetics followed by ^{1}H NMR spectroscopy mesitylene was used as an internal integration standard (IIS).

5.2 Kinetic Experiments in Dichloromethane

5.2.1 Kinetics of the reactions of ethyl acrylate (1) with PR_3

Kinetics of the reactions of ethyl acrylate (**1**) with $P(pfp)_3$ in CD_2Cl_2 (at 20 °C)

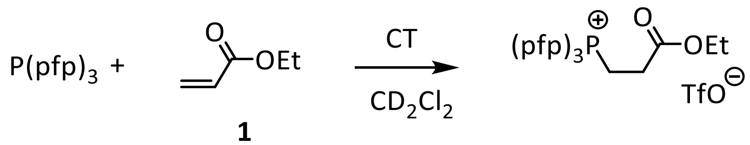

1

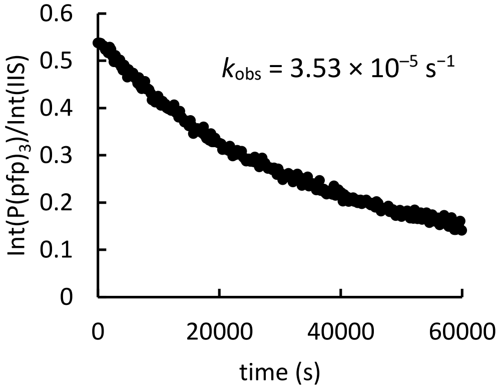

Table S5. Kinetics of the reaction of $P(pfp)_3$ and **1** (proton source: CT, ^{31}P NMR method, monitoring of the decreasing ^{31}P NMR signal of $P(pfp)_3$ at −9.1 ppm, in CD_2Cl_2, Ph_3PO as internal integration standard IIS)

$[P(pfp)_3]$ (M)	[**1**] (M)	[CT] (M)	k_{obs} (s^{-1})
1.02×10^{-1}	**9.74×10^{-1}**	1.89×10^{-1}	**2.19×10^{-5}**
1.02×10^{-1}	**1.60**	1.86×10^{-1}	**3.53×10^{-5}**
1.04×10^{-1}	**2.22**	1.95×10^{-1}	**5.09×10^{-5}**
1.02×10^{-1}	**2.83**	1.91×10^{-1}	**6.25×10^{-5}**
**$k_2 = 2.22 \times 10^{-5}$ M^{-1} s^{-1}**			

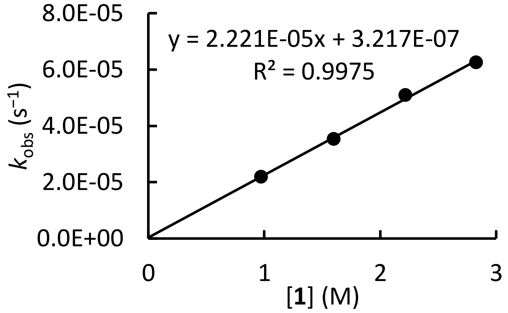

Kinetics of the reactions of ethyl acrylate (**1**) with PPh_3 in CD_2Cl_2 (at 20 °C)

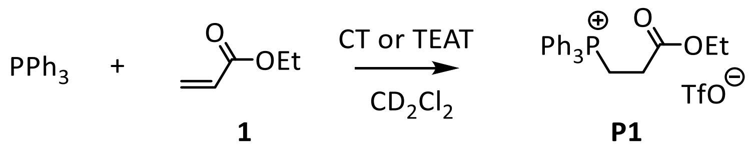

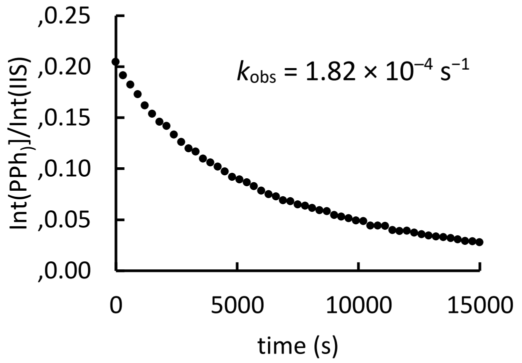

Table S6. Kinetics of the reaction of PPh_3 and **1** (proton source: CT or TEAT, ^{31}P NMR method, monitoring of the decreasing ^{31}P NMR signal of PPh_3 at −5.5 ppm, in CD_2Cl_2, Ph_3PO as internal integration standard IIS)

$[PPh_3]$ (M)	[**1**] (M)	[CT] (M)	k_{obs} (s^{-1})
1.03×10^{-1}	$\mathbf{9.79 \times 10^{-1}}$	2.25×10^{-1}	$\mathbf{1.54 \times 10^{-4}}$
1.20×10^{-1}	**1.16**	2.42×10^{-1}	$\mathbf{1.82 \times 10^{-4}}$
1.13×10^{-1}	**1.40**	2.11×10^{-1}	$\mathbf{2.18 \times 10^{-4}}$
1.13×10^{-1}	**1.59**	2.11×10^{-1}	$\mathbf{2.49 \times 10^{-4}}$
$[PPh_3]$ (M)	[**1**] (M)	[TEAT] (M)	k_{obs} (s^{-1})
8.76×10^{-2}	$\mathbf{8.80 \times 10^{-1}}$	2.70×10^{-1}	$\mathbf{1.38 \times 10^{-4}}$
1.06×10^{-1}	**1.06**	3.08×10^{-1}	$\mathbf{1.66 \times 10^{-4}}$
1.08×10^{-1}	**1.28**	3.07×10^{-1}	$\mathbf{2.00 \times 10^{-4}}$
1.07×10^{-1}	**1.49**	2.88×10^{-1}	$\mathbf{2.33 \times 10^{-4}}$

$\mathbf{k_2 = 1.55 \times 10^{-4}\ M^{-1}\ s^{-1}}$

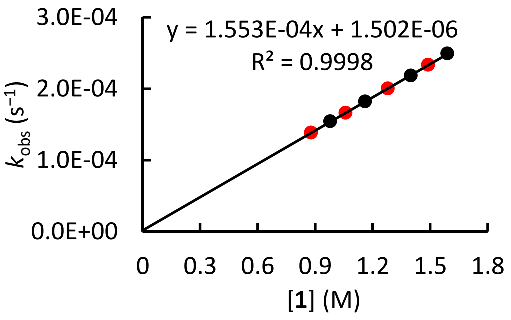

^{31}P NMR spectroscopic analysis of selected samples indicated selective conversion of **1** to **P1** (δ_P = +24.2 ppm in CD_2Cl_2) under the conditions of the kinetic experiments (Figure S25).

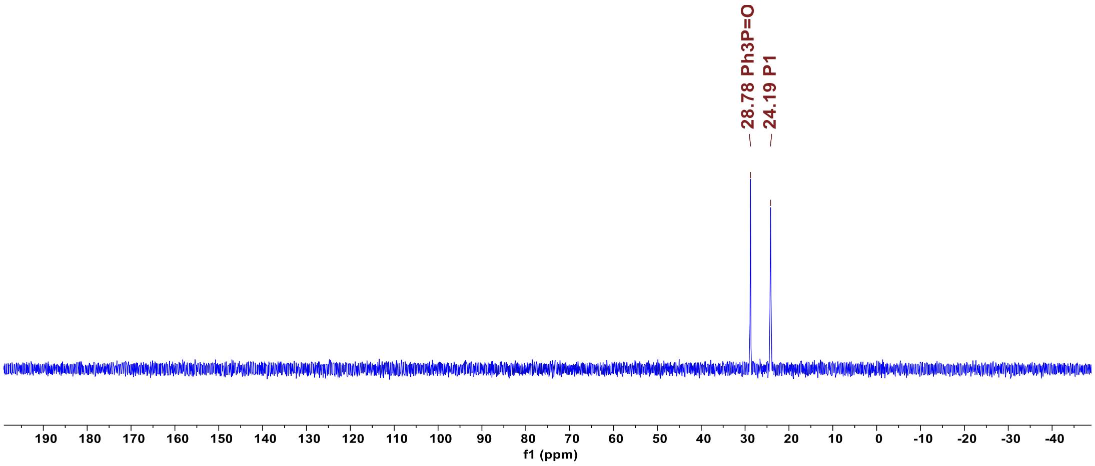

Fig. S25. ^{31}P NMR (81 MHz) spectrum of the sample with $[Ph_3P]_0$ = 0.103 M, $[\mathbf{1}]_0$ = 0.979 M and $[CT]_0$ = 0.225 M in CD_2Cl_2 after completion of their reaction (Ph_3P=O as internal standard).

Kinetics of the reactions of ethyl acrylate (**1**) with P(ani)$_3$ in CD_2Cl_2 (at 20 °C)

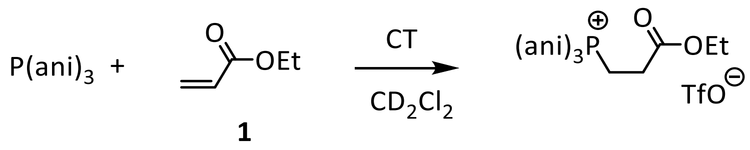

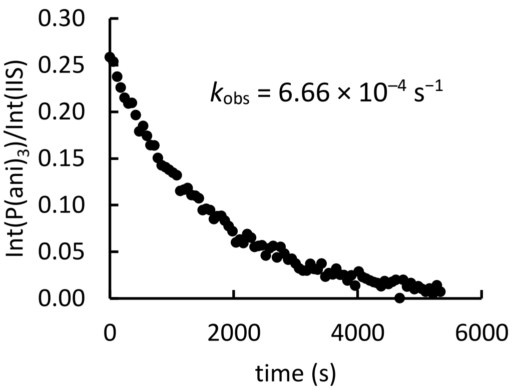

Table S7. Kinetics of the reaction of P(ani)$_3$ and **1** (proton source: CT, ^{31}P NMR method, monitoring of the decreasing ^{31}P NMR signal of P(ani)$_3$ at −10.4 ppm, in CD_2Cl_2, Ph_3PO as internal integration standard IIS)

[P(ani)$_3$] (M)	[**1**] (M)	[CT] (M)	k_{obs} (s^{-1})
4.30×10^{-2}	**4.42×10^{-1}**	7.37×10^{-2}	**4.32×10^{-4}**
4.85×10^{-2}	**7.61×10^{-1}**	8.41×10^{-2}	**6.66×10^{-4}**
4.56×10^{-2}	**9.04×10^{-1}**	7.45×10^{-2}	**8.52×10^{-4}**
4.85×10^{-2}	**1.10**	8.63×10^{-2}	**1.04×10^{-3}**

$k_2 = 9.32 \times 10^{-4}$ M^{-1} s^{-1}

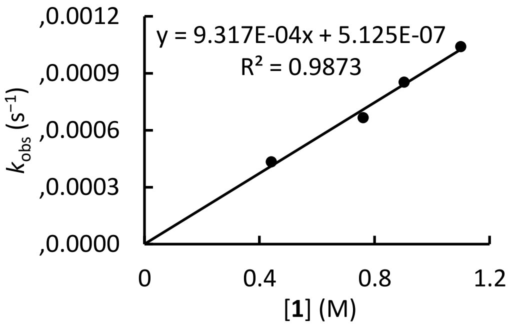

Kinetics of the reactions of ethyl acrylate (**1**) with $PMePh_2$ in CD_2Cl_2 (at 20 °C)

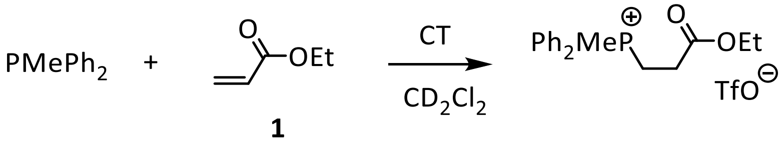

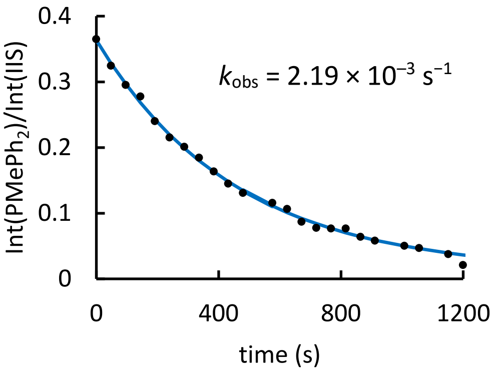

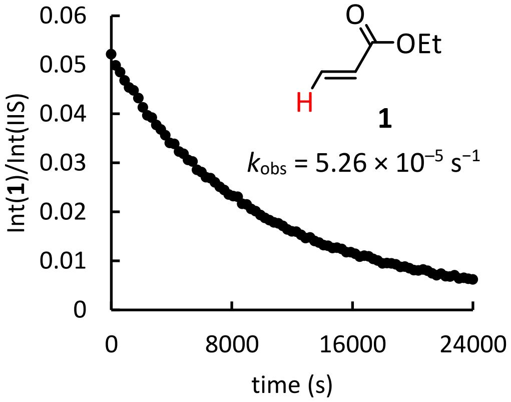

Table S8. Kinetics of the reaction of $PMePh_2$ and **1** (proton source: CT, ^{31}P NMR method, monitoring of the decreasing ^{31}P NMR signal of $P(ani)_3$ at −27.1 ppm, in CD_2Cl_2, Ph_3PO as internal integration standard IIS)

$[PMePh_2]$ (M)	[**1**] (M)	[CT] (M)	k_{obs} (s^{-1})
7.59×10^{-2}	**7.16×10^{-1}**	1.63×10^{-1}	**1.74×10^{-3}**
8.74×10^{-2}	**9.28×10^{-1}**	1.64×10^{-1}	**2.19×10^{-3}**
9.29×10^{-2}	**1.13**	1.92×10^{-1}	**2.67×10^{-3}**
9.09×10^{-2}	**1.34**	2.08×10^{-1}	**3.21×10^{-3}**

$k_2 = 2.36 \times 10^{-3}$ M^{-1} s^{-1}

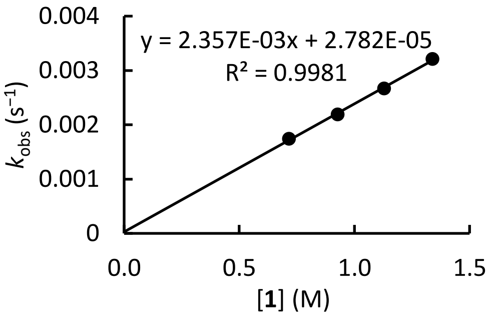

Table S9. Kinetics of the reaction of $PMePh_2$ and **1** (proton source: CT, ^{1}H NMR method, monitoring of the decreasing ^{1}H NMR signal of ethyl acrylate (**1**) at 5.80 ppm, in CD_2Cl_2, mesitylene as internal integration standard IIS)

$[PMePh_2]$ (M)	[**1**] (M)	[CT] (M)	k_{obs} (s^{-1})
**5.59×10^{-2}**	2.66×10^{-3}	5.59×10^{-3}	**5.26×10^{-5}**
**9.59×10^{-2}**	7.99×10^{-3}	1.49×10^{-2}	**1.14×10^{-4}**
**1.29×10^{-1}**	1.33×10^{-2}	2.61×10^{-2}	**1.74×10^{-4}**
**1.69×10^{-1}**	1.60×10^{-2}	2.98×10^{-2}	**2.39×10^{-4}**

$k_2 = 1.66 \times 10^{-3}$ M^{-1} s^{-1}

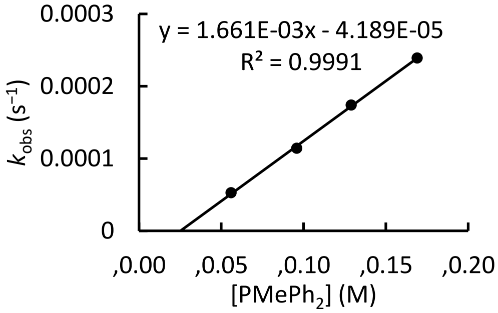

Kinetics of the reactions of ethyl acrylate (**1**) with PMe_2Ph in CD_2Cl_2 (at 20 °C)

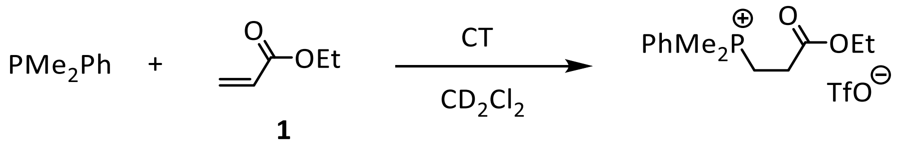

1

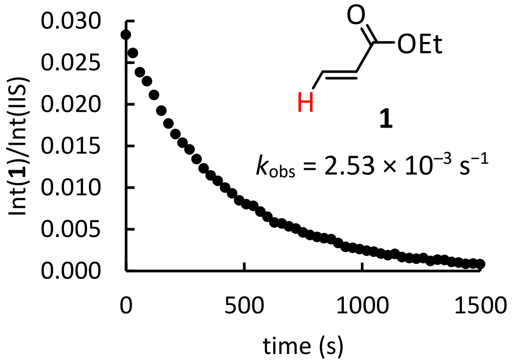

Table S10. Kinetics of the reaction of PMe_2Ph and **1** (proton source: CT, ^1H NMR method, monitoring of the decreasing ^1H NMR signal of **1** at 5.80 ppm, in CD_2Cl_2, mesitylene as internal integration standard IIS)

$[PMe_2Ph]$ (M)	[**1**] (M)	[CT] (M)	k_{obs} (s^{-1})
**9.36×10^{-2}**	9.79×10^{-3}	2.14×10^{-2}	**1.87×10^{-3}**
**1.26×10^{-1}**	1.27×10^{-2}	2.14×10^{-2}	**2.53×10^{-3}**
**1.61×10^{-1}**	1.66×10^{-2}	2.14×10^{-2}	**3.22×10^{-3}**
**1.94×10^{-1}**	1.86×10^{-2}	2.14×10^{-2}	**3.88×10^{-3}**
1.26×10^{-1}	1.26×10^{-2}	3.71×10^{-2}	2.47×10^{-3}
1.26×10^{-1}	1.26×10^{-2}	7.33×10^{-2}	2.55×10^{-3}

$k_2 = 2.00 \times 10^{-2}\ M^{-1}\ s^{-1}$

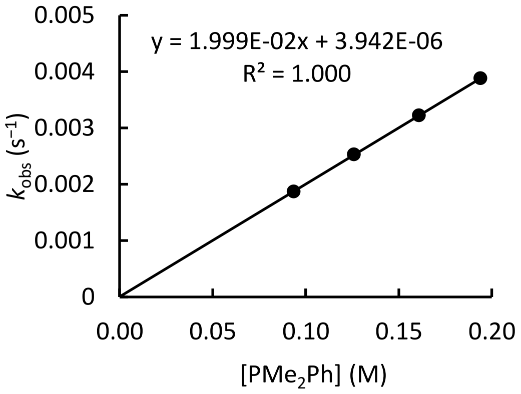

Kinetics of the reactions of ethyl acrylate (**1**) with PBu_3 in CH_2Cl_2 (at 20 °C)

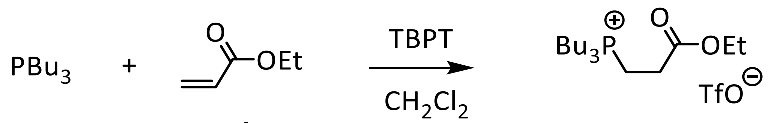

1

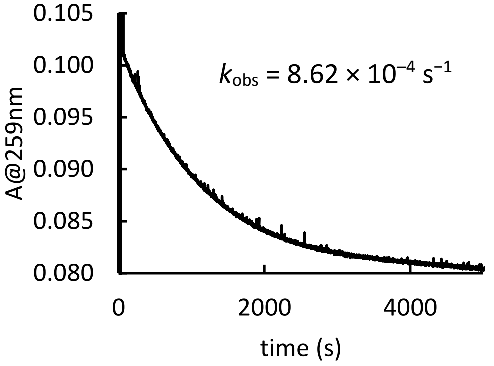

Table S11. Kinetics of the reactions of PBu_3 and **1** (proton source: TBPT, conventional UV-Vis method, detection at 259 nm, in CH_2Cl_2)

$[PBu_3]$ (M)	[**1**] (M)	[TBPT] (M)	k_{obs} (s^{-1})
**1.10×10^{-2}**	1.16×10^{-3}	2.44×10^{-3}	**8.62×10^{-4}**
**1.64×10^{-2}**	1.16×10^{-3}	2.44×10^{-3}	**1.15×10^{-3}**
**2.20×10^{-2}**	1.16×10^{-3}	2.45×10^{-3}	**1.48×10^{-3}**
**2.75×10^{-2}**	1.34×10^{-3}	2.80×10^{-3}	**1.83×10^{-3}**
**3.27×10^{-2}**	1.15×10^{-3}	2.43×10^{-3}	**2.11×10^{-3}**
3.27×10^{-2}	1.19×10^{-3}	3.72×10^{-3}	2.07×10^{-3}

$k_2 = 5.83 \times 10^{-2}$ M^{-1} s^{-1}

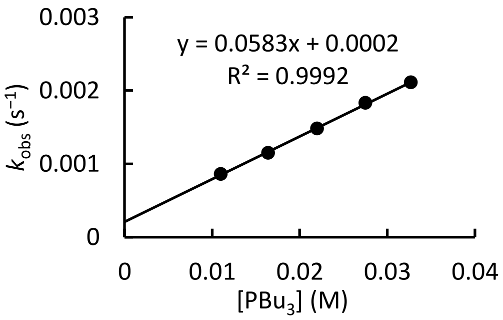

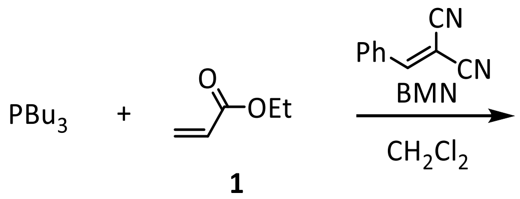

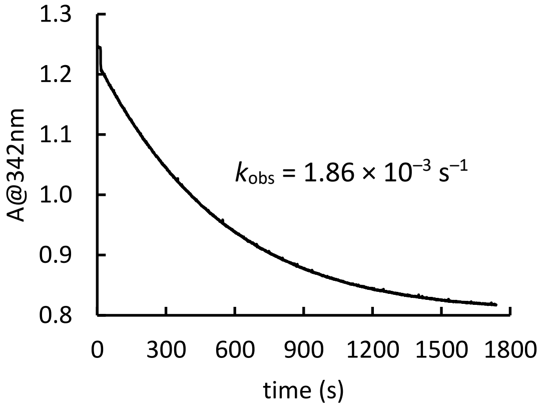

Table S12. Kinetics of the reactions of PBu_3 and **1** (with electrophile BMN as trapping reagent, conventional UV-Vis method, detection at 342 nm, in CH_2Cl_2)

$[PBu_3]$ (M)	[**1**] (M)	[BMN] (M)	k_{obs} (s^{-1})
5.35×10^{-4}	**1.13×10^{-2}**	1.12×10^{-3}	**6.39×10^{-4}**
5.20×10^{-4}	**1.88×10^{-2}**	1.09×10^{-3}	**1.10×10^{-3}**
5.41×10^{-4}	**2.50×10^{-2}**	1.11×10^{-3}	**1.34×10^{-3}**
5.38×10^{-4}	**3.31×10^{-2}**	1.10×10^{-3}	**1.86×10^{-3}**

$k_2 = 5.47 \times 10^{-2}$ M^{-1} s^{-1}

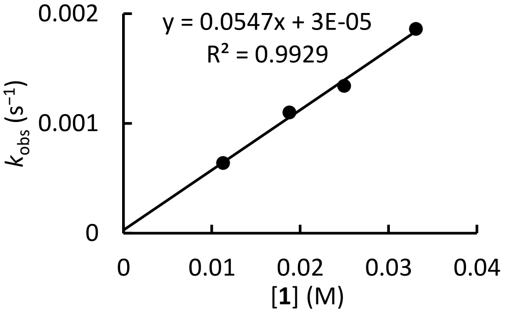

Kinetics of the reactions of ethyl acrylate (**1**) with PMe_3 in CH_2Cl_2 (at 20 °C)

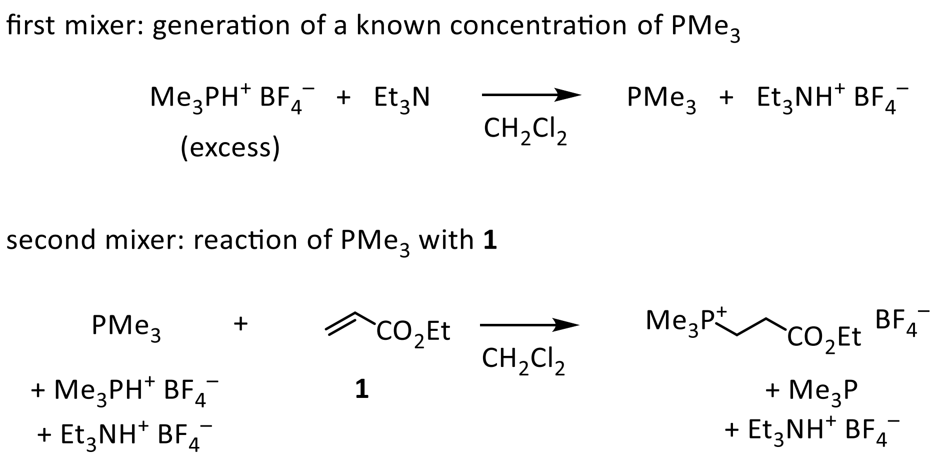

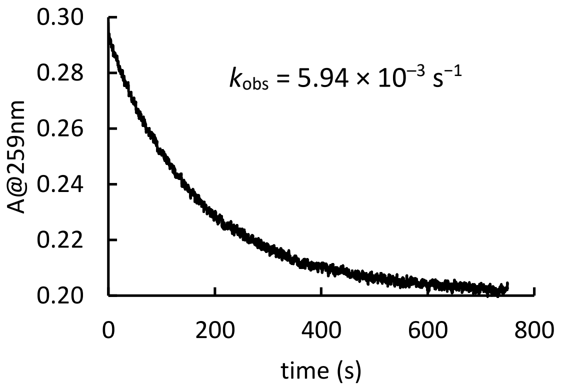

Table S13. Kinetics of the reactions of PMe_3 and **1** (PMe_3 was generated by the deprotonation of an excess of trimethylphosphonium tetrafluoroborate with a known amount of NEt_3 in the first step of the sequential mixing stopped-flow method, detection at 259 nm, in CH_2Cl_2)

$[PMe_3]$ (M)	[**1**] (M)	$[HP^+Me_3]$ (M)	k_{obs} (s^{-1})
**1.40×10^{-2}**	1.37×10^{-3}	5.38×10^{-3}	**1.23×10^{-3}** [a]
**1.73×10^{-2}**	1.82×10^{-3}	1.92×10^{-3}	**1.62×10^{-3}**
**2.59×10^{-2}**	1.82×10^{-3}	2.87×10^{-3}	**2.65×10^{-3}**
**3.46×10^{-2}**	1.82×10^{-3}	3.83×10^{-3}	**3.95×10^{-3}**
**4.32×10^{-2}**	1.82×10^{-3}	4.79×10^{-3}	**4.78×10^{-3}**
**5.19×10^{-2}**	1.82×10^{-3}	5.75×10^{-3}	**5.94×10^{-3}**

[a] Conventional UV-Vis method was used.

$k_2 = 1.24 \times 10^{-1}$ M^{-1} s^{-1}

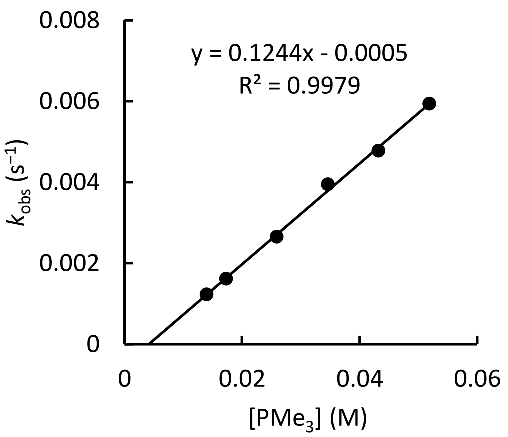

Kinetics of the reactions of ethyl acrylate (**1**) with PCy_3 in CD_2Cl_2 (at 20 °C)

first step: generation of a known concentration of PCy_3

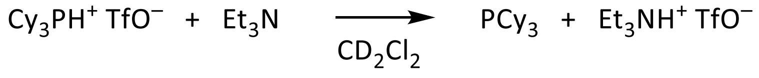

second step: reaction of PCy_3 with **1**

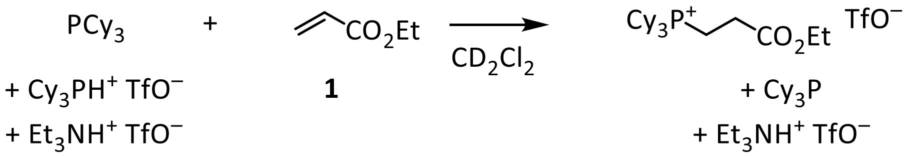

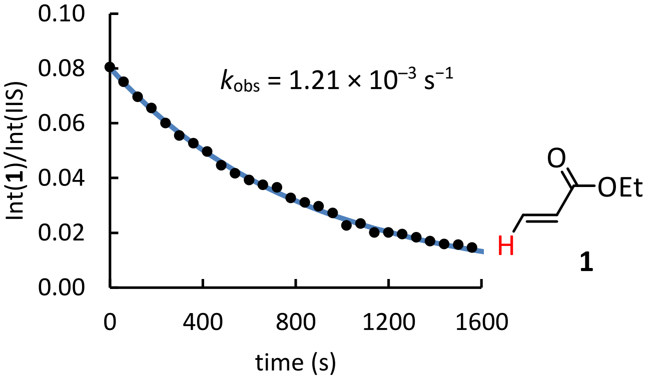

Table S14. Kinetics of the reactions of PCy_3 and **1** (PCy_3 was generated by the deprotonation of an excess of tricyclohexylphosphonium triflate with a known amount of NEt_3 in the first step, ^1H NMR method, monitoring of the decreasing ^1H NMR signal of **1** at 5.80 ppm, in CD_2Cl_2, mesitylene as internal integration standard IIS)

$[PCy_3]$ (M)	[**1**] (M)	$[HP^+Cy_3]$ (M)	k_{obs} (s^{-1})
**1.56×10^{-2}**	1.56×10^{-3}	5.12×10^{-3}	**4.26×10^{-4}**
**2.08×10^{-2}**	2.19×10^{-3}	6.82×10^{-3}	**5.97×10^{-4}**
**2.59×10^{-2}**	2.50×10^{-3}	8.53×10^{-3}	**7.91×10^{-4}**
**3.63×10^{-2}**	3.75×10^{-3}	1.19×10^{-2}	**1.21×10^{-3}**

$k_2 = 3.82 \times 10^{-2}\ M^{-1}\ s^{-1}$

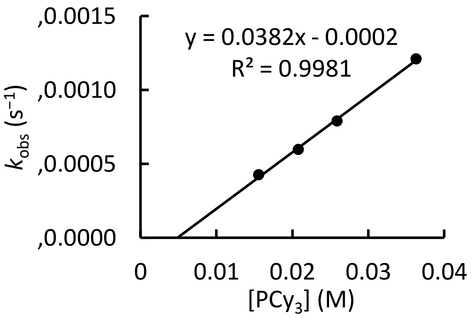

Kinetics of the reactions of ethyl acrylate (**1**) with $POct_3$ in CH_2Cl_2 (at 20 °C)

first step: generation of a known concentration of $POct_3$

$$Oct_3PH^+ \, TfO^- + Et_3N \xrightarrow[CH_2Cl_2]{} POct_3 + Et_3NH^+ \, TfO^-$$

second step: reaction of $POct_3$ with **1**

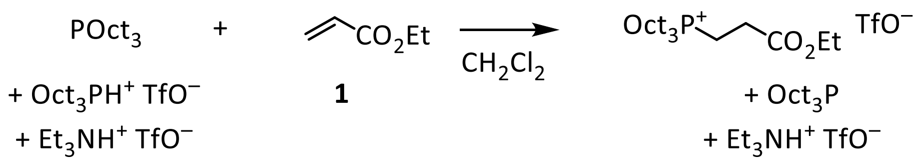

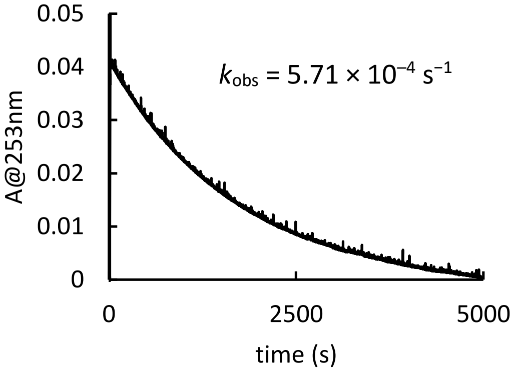

Table S15. Kinetics of the reactions of $POct_3$ and **1** ($POct_3$ was generated by the deprotonation of an excess of trioctylphosphonium triflate with a known amount of NEt_3 in the first step, conventional UV-Vis method, detection at 253 nm, in CH_2Cl_2)

$[POct_3]$ (M)	[**1**] (M)	$[HP^+Oct_3]$ (M)	k_{obs} (s^{-1})
**8.31×10^{-3}**	1.15×10^{-3}	3.03×10^{-3}	**5.71×10^{-4}**
**1.12×10^{-2}**	1.16×10^{-3}	2.88×10^{-3}	**7.09×10^{-4}**
**1.36×10^{-2}**	1.14×10^{-3}	2.94×10^{-3}	**8.69×10^{-4}**
**1.87×10^{-2}**	1.23×10^{-3}	8.59×10^{-3}	**1.13×10^{-3}**

$k_2 = 5.45 \times 10^{-2}$ s^{-1}

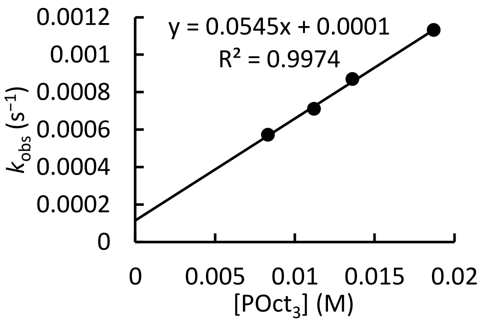

Kinetics of the reactions of ethyl acrylate (**1**) with P(1-Np)$_3$ in CD_2Cl_2 (at 20 °C)

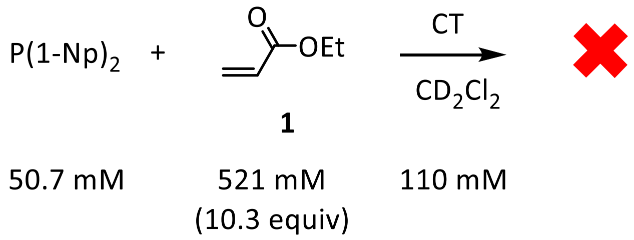

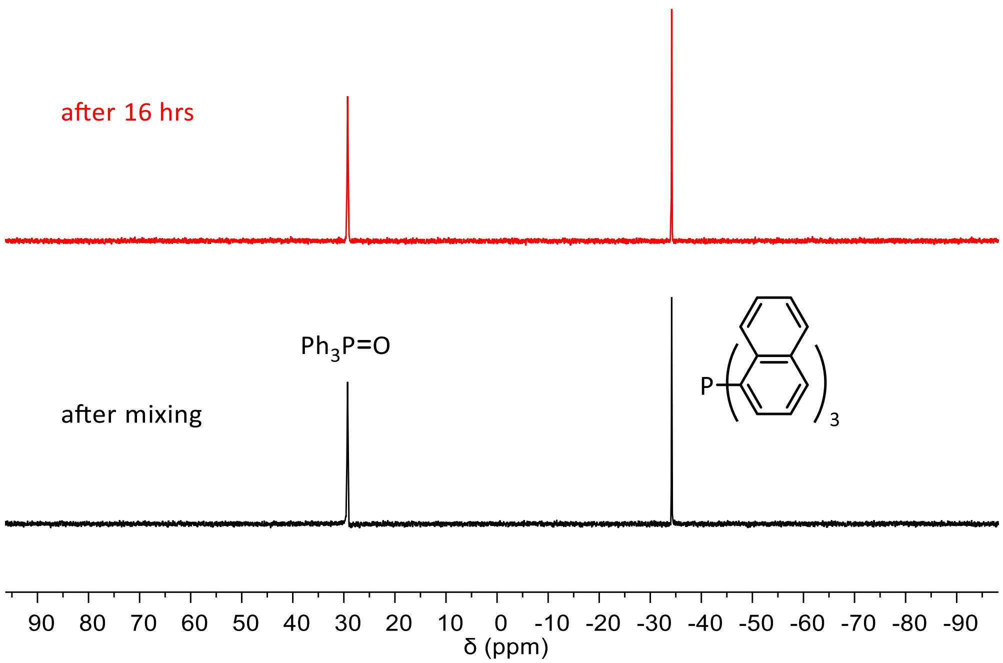

Fig. S26. ^{31}P NMR (162 MHz) spectra of the mixture of P(1-Np)$_3$ with **1** in CD_2Cl_2 (proton source: CT, Ph_3PO as internal integration standard)

5.2.2 Kinetics of the reactions of ethyl 2,3-butadienoate (2) with PR_3

Kinetics of the reactions of ethyl buta-2,3-dienoate (**2**) with $P(pfp)_3$ in CD_2Cl_2 (at 20 °C)

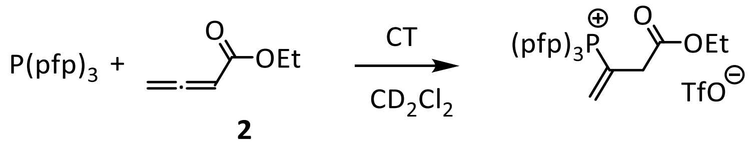

2

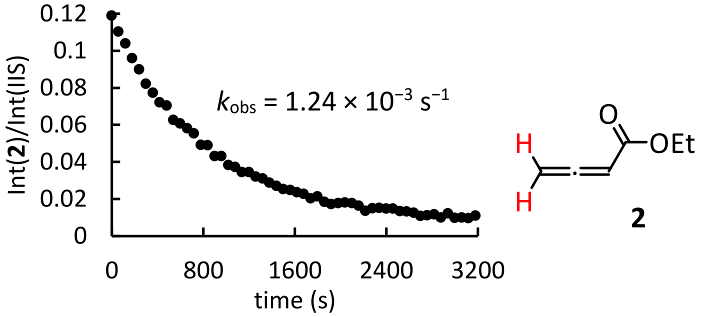

Table S16. Kinetics of the reaction of $P(pfp)_3$ and **2** (proton source: CT, ^1H NMR method, monitoring of the decreasing ^1H NMR signal of **2** at 5.21 ppm, in CD_2Cl_2, mesitylene as internal integration standard)

$[P(pfp)_3]$ (M)	[**2**] (M)	[CT] (M)	k_{obs} (s^{-1})
**1.34×10^{-1}**	1.36×10^{-2}	2.82×10^{-2}	**4.67×10^{-4}**
**1.97×10^{-1}**	2.00×10^{-2}	3.38×10^{-2}	**7.32×10^{-4}**
**2.75×10^{-1}**	2.00×10^{-2}	3.38×10^{-2}	**9.70×10^{-4}**
**3.56×10^{-1}**	2.00×10^{-2}	3.38×10^{-2}	**1.24×10^{-3}**

$k_2 = 3.43 \times 10^{-3}$ M^{-1} s^{-1}

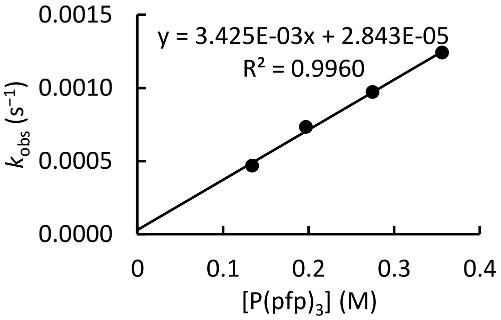

Table S17. Kinetics of the reaction of $P(pfp)_3$ and **2** (proton source: CT, ^{31}P NMR method, monitoring of the decreasing ^{31}P NMR signal of $P(pfp)_3$ at –9.2 ppm, in CD_2Cl_2, Ph_3PO as internal integration standard)

$[P(pfp)_3]$ (M)	[**2**] (M)	[CT] (M)	k_{obs} (s^{-1})
3.36×10^{-2}	**3.21×10^{-1}**	6.78×10^{-2}	1.51×10^{-3}
4.96×10^{-2}	**4.87×10^{-1}**	9.25×10^{-2}	2.05×10^{-3}
6.72×10^{-2}	**6.57×10^{-1}**	1.18×10^{-2}	2.75×10^{-3}
8.00×10^{-2}	**8.23×10^{-1}**	1.33×10^{-2}	3.40×10^{-3}

$k_2 = 3.80 \times 10^{-3}$ M^{-1} s^{-1}

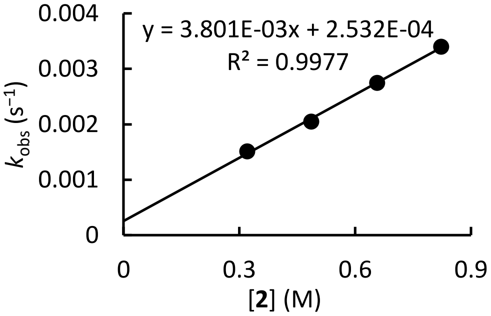

Kinetics of the reactions of ethyl buta-2,3-dienoate (**2**) with PPh_3 in CD_2Cl_2 (at 20 °C)[S6]

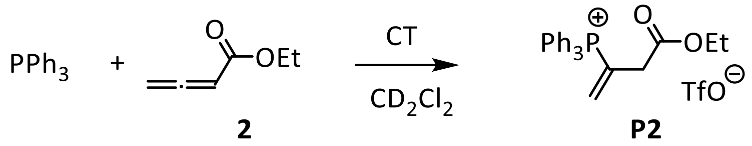

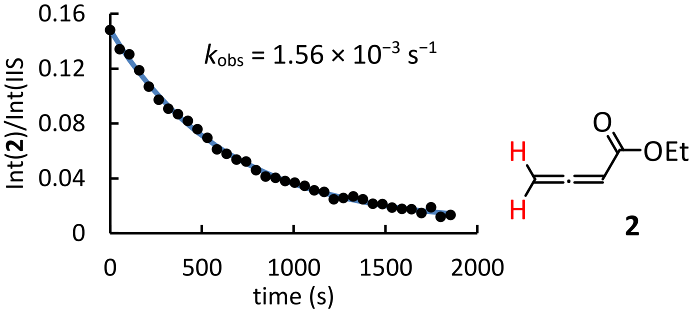

Table S18. Kinetics of the reactions of PPh_3 and **2** (proton source: CT, ^{1}H NMR method, monitoring of the decreasing signal for the $=CH_2$ group in **2** at 5.21 ppm, in CD_2Cl_2, mesitylene as internal integration standard)

$[PPh_3]$ (M)	[**2**] (M)	[CT] (M)	k_{obs} (s^{-1})
**1.75×10^{-1}**	1.82×10^{-2}	3.71×10^{-2}	**1.56×10^{-3}**
**2.34×10^{-1}**	1.82×10^{-2}	3.71×10^{-2}	**1.99×10^{-3}**
**2.90×10^{-1}**	2.90×10^{-2}	5.93×10^{-2}	**2.44×10^{-3}**
**3.48×10^{-1}**	2.90×10^{-2}	5.93×10^{-2}	**2.88×10^{-3}**

$k_2 = 7.67 \times 10^{-3}$ M^{-1} s^{-1} (from ref. S6)

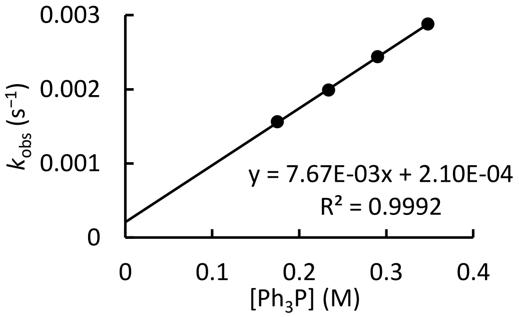

^{31}P NMR spectroscopic analysis of selected samples indicated selective conversion of **2** to **P2** (δ_P = +25.3 ppm in CD_2Cl_2) under the conditions of the kinetic experiments (Figure S27).

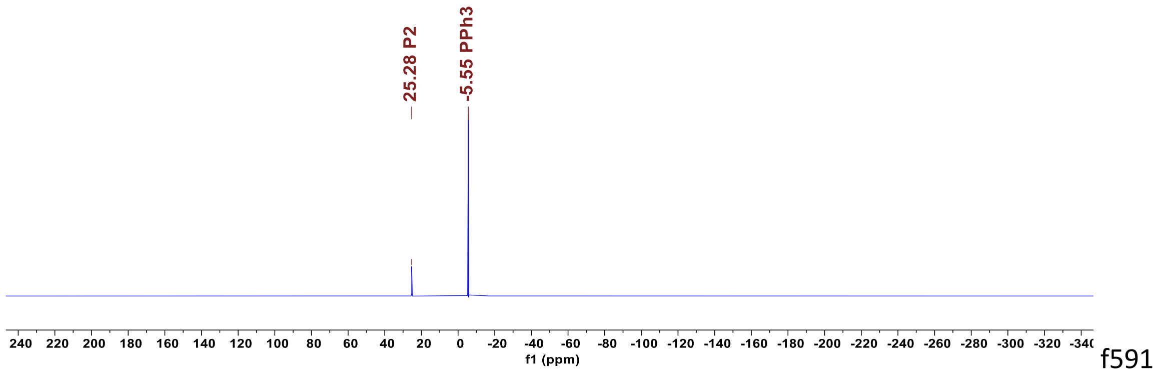

Fig. S27. ^{31}P NMR (162 MHz) spectrum of a sample with $[Ph_3P]_0$ = 250 mM, $[\mathbf{2}]_0$ = 18.7 mM and $[CT]_0$ = 40.6 mM in CD_2Cl_2 after completion of their reaction.

Kinetics of the reactions of ethyl buta-2,3-dienoate (**2**) with $P(ani)_3$ in CH_2Cl_2 (at 20 °C)

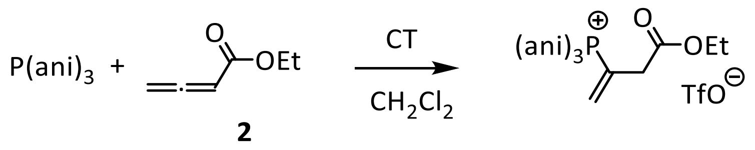

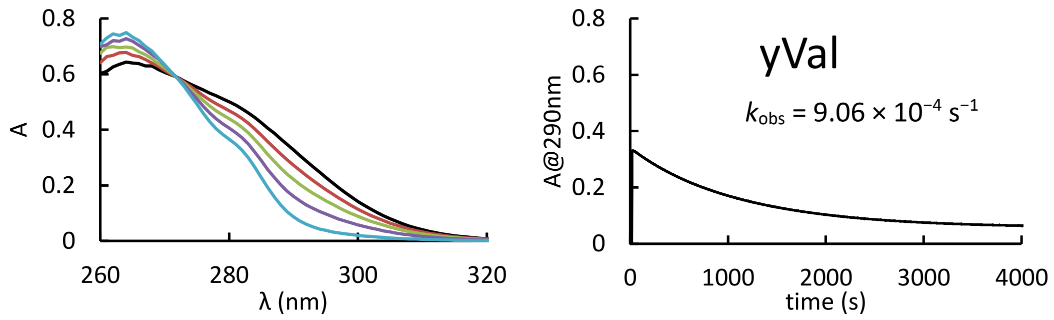

Table S19. Kinetics of the reactions of $P(ani)_3$ and **2** (proton source: CT, conventional UV-Vis method, detection at 290 nm, in CH_2Cl_2)

$[P(ani)_3]$ (M)	[**2**] (M)	[CT] (M)	k_{obs} (s^{-1})
6.83×10^{-5}	$\mathbf{1.18 \times 10^{-2}}$	1.30×10^{-4}	$\mathbf{4.72 \times 10^{-4}}$
6.81×10^{-5}	$\mathbf{1.81 \times 10^{-2}}$	1.29×10^{-4}	$\mathbf{7.18 \times 10^{-4}}$
6.75×10^{-5}	$\mathbf{2.31 \times 10^{-2}}$	1.28×10^{-4}	$\mathbf{9.06 \times 10^{-4}}$
6.74×10^{-5}	$\mathbf{2.93 \times 10^{-2}}$	1.28×10^{-4}	$\mathbf{1.13 \times 10^{-3}}$

$\mathbf{k_2 = 3.76 \times 10^{-2}\ M^{-1}\ s^{-1}}$

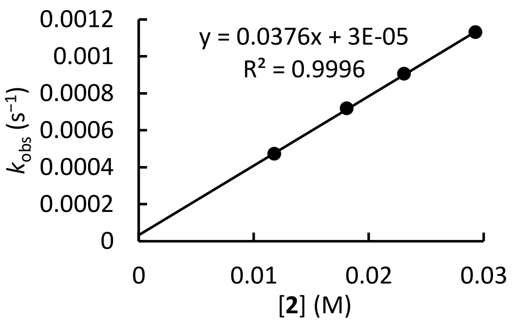

Kinetics of the reactions of ethyl buta-2,3-dienoate (**2**) with $PMePh_2$ in CH_2Cl_2 (at 20 °C)

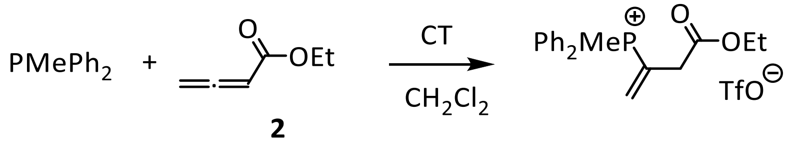

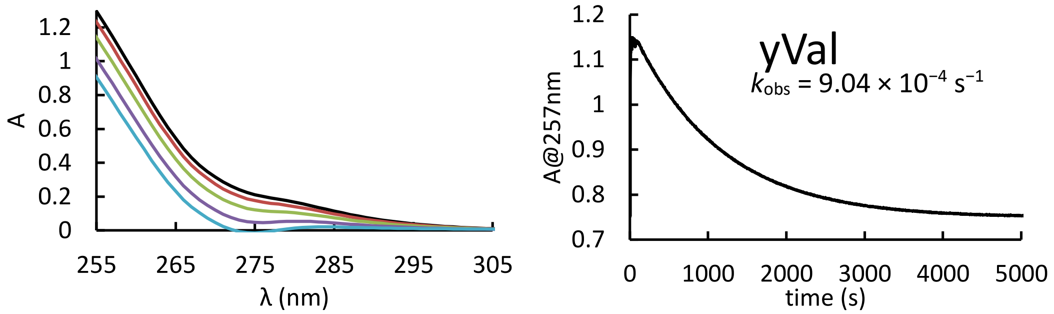

Table S20. Kinetics of the reactions of $PMePh_2$ and **2** (proton source: CT, conventional UV-Vis method, detection at 257 nm, in CH_2Cl_2)

$[PMePh_2]$ (M)	[**2**] (M)	[CT] (M)	k_{obs} (s^{-1})
1.06×10^{-4}	$\mathbf{1.33 \times 10^{-2}}$	2.29×10^{-4}	$\mathbf{9.04 \times 10^{-4}}$
1.03×10^{-4}	$\mathbf{1.57 \times 10^{-2}}$	2.22×10^{-4}	$\mathbf{1.10 \times 10^{-3}}$
1.06×10^{-4}	$\mathbf{1.99 \times 10^{-2}}$	2.28×10^{-4}	$\mathbf{1.37 \times 10^{-3}}$
1.02×10^{-4}	$\mathbf{2.32 \times 10^{-2}}$	2.21×10^{-4}	$\mathbf{1.51 \times 10^{-3}}$

$\mathbf{k_2 = 6.12 \times 10^{-2}\ M^{-1}\ s^{-1}}$

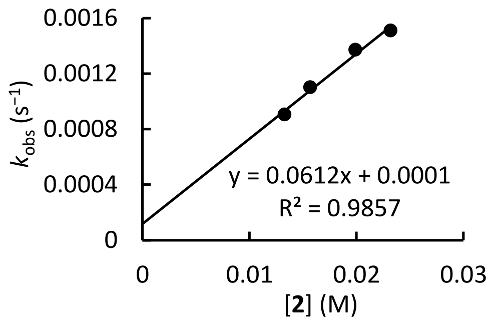

Kinetics of the reactions of ethyl buta-2,3-dienoate (**2**) with PMe_2Ph in CH_2Cl_2 (at 20 °C)

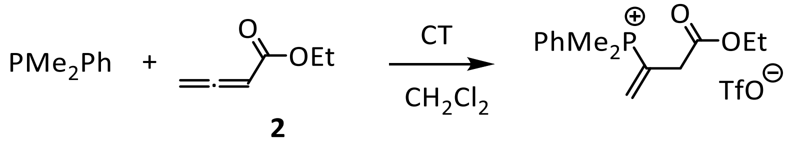

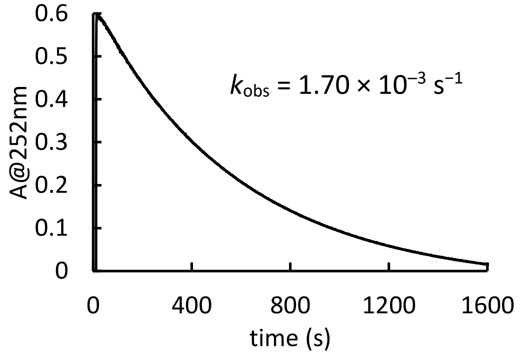

Table S21. Kinetics of the reactions of PMe_2Ph and **2** (proton source: CT, conventional UV-Vis method, detection at 252 nm, in CH_2Cl_2)

$[PMe_2Ph]$ (M)	[**2**] (M)	[CT] (M)	k_{obs} (s^{-1})
2.30×10^{-4}	**5.30×10^{-3}**	2.55×10^{-4}	**1.70×10^{-3}**
2.21×10^{-4}	**7.16×10^{-3}**	2.52×10^{-4}	**2.20×10^{-3}**
2.13×10^{-4}	**8.74×10^{-3}**	2.44×10^{-4}	**2.76×10^{-3}**
2.37×10^{-4}	**1.13×10^{-2}**	2.63×10^{-4}	**3.28×10^{-3}**

$k_2 = 2.68 \times 10^{-1}\ M^{-1}\ s^{-1}$

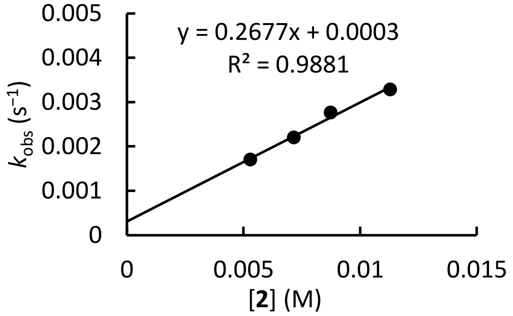

Kinetics of the reactions of ethyl buta-2,3-dienoate (**2**) with PBu_3 in CH_2Cl_2 (at 20 °C)[S6]

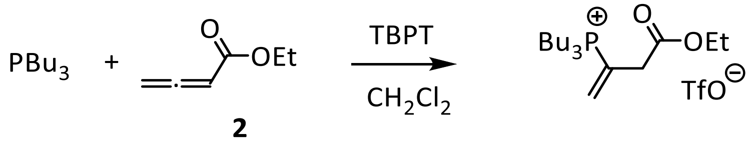

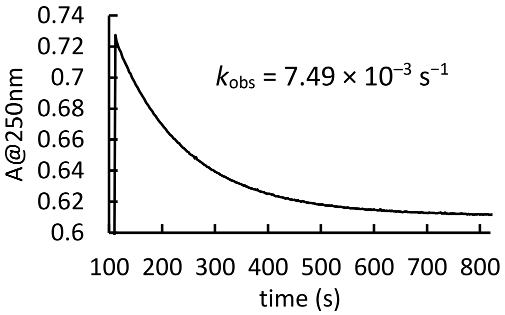

Table S22. Kinetics of the reactions of PBu_3 and **2** (proton source: TBPT, conventional UV-Vis method, detection at 250 nm, in CH_2Cl_2)

$[PBu_3]$ (M)	[**2**] (M)	[TBPT] (M)	k_{obs} (s^{-1})
**3.26×10^{-3}**	3.38×10^{-4}	9.94×10^{-4}	**1.73×10^{-3}**
**5.74×10^{-3}**	5.95×10^{-4}	1.84×10^{-3}	**3.34×10^{-3}**
**8.59×10^{-3}**	8.98×10^{-4}	2.77×10^{-3}	**5.03×10^{-3}**
**1.09×10^{-2}**	8.83×10^{-4}	2.72×10^{-3}	**6.59×10^{-3}**
**1.23×10^{-2}**	1.21×10^{-3}	2.73×10^{-3}	**7.49×10^{-3}**

$k_2 = 6.35 \times 10^{-1}$ M^{-1} s^{-1} (from ref. S6)

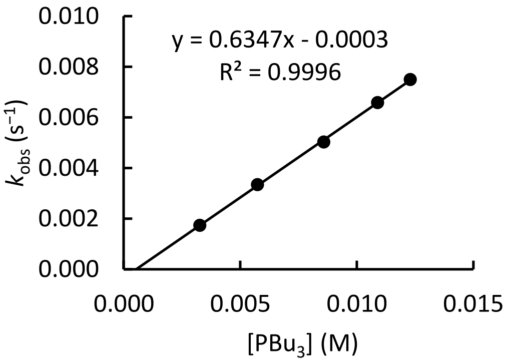

Kinetics of the reactions of ethyl buta-2,3-dienoate (**2**) with PMe_3 in CH_2Cl_2 (at 20 °C)

first mixer: generation of a known concentration of PMe_3

$$\underset{\text{(excess)}}{Me_3PH^+ BF_4^-} + Et_3N \xrightarrow[CH_2Cl_2]{} PMe_3 + Et_3NH^+ TfO^-$$

second step: reaction of PMe_3 with **2**

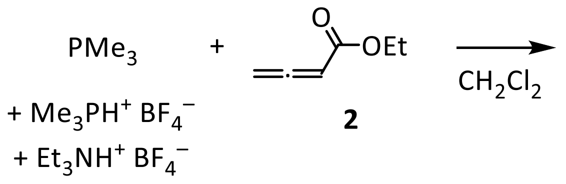

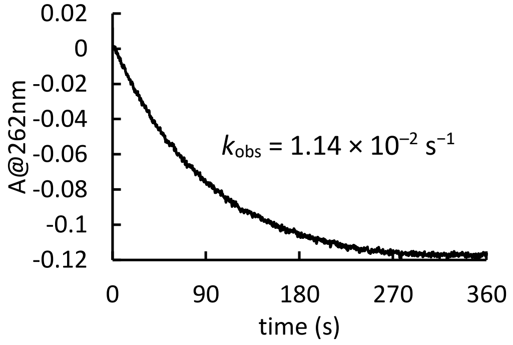

(A solution of electrophile **2** was recorded as the baseline (A_0 = 0). Consumption of **2** then gave rise to negative absorption values.)

Table S23. Kinetics of the reactions of PMe_3 and **2** (PMe_3 was generated by the deprotonation of trimethylphosphonium tetrafluoroborate with NEt_3 in the first step of the sequential mixing stopped-flow method, detection at 262 nm)

$[PMe_3]$ (M)	[**2**] (M)	$[HP^+Me_3]$ (M)	k_{obs} (s^{-1})
**1.50×10^{-2}**	1.52×10^{-3}	2.84×10^{-3}	**1.14×10^{-2}**
**2.25×10^{-2}**	1.52×10^{-3}	4.25×10^{-3}	**1.77×10^{-2}**
**3.00×10^{-2}**	1.52×10^{-3}	5.76×10^{-3}	**2.59×10^{-2}**
**3.75×10^{-2}**	1.52×10^{-3}	7.09×10^{-3}	**3.29×10^{-2}**

$k_2 = 9.69 \times 10^{-1}\ M^{-1}\ s^{-1}$

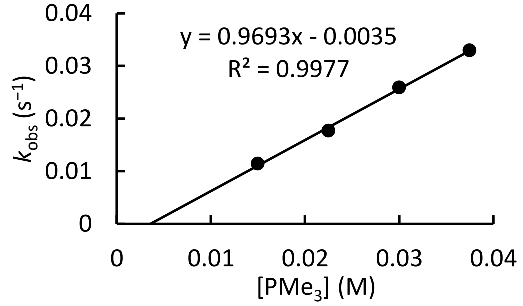

Kinetics of the reactions of ethyl buta-2,3-dienoate (**2**) with PCy_3 in CH_2Cl_2 (at 20 °C)

first step: generation of a known concentration of PCy_3

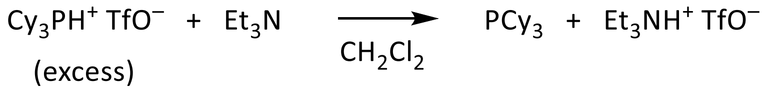

second step: reaction of PCy_3 with **2**

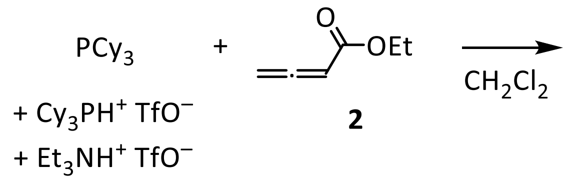

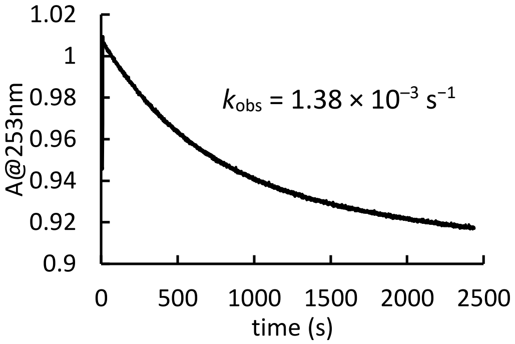

Table S24. Kinetics of the reactions of PCy_3 and **2** (in a first step PCy_3 was generated in CH_2Cl_2 by the deprotonation of tricyclohexylphosphonium triflate with a known amount of NEt_3, in a second step a solution of **2** in dichloromethane was added, conventional UV-Vis method, detection at 253 nm)

$[PCy_3]$ (M)	[**2**] (M)	$[HP^+Cy_3]$ (M)	k_{obs} (s^{-1})
$\mathbf{6.26 \times 10^{-3}}$	6.32×10^{-4}	1.26×10^{-3}	$\mathbf{9.92 \times 10^{-4}}$
$\mathbf{7.79 \times 10^{-3}}$	7.55×10^{-4}	1.57×10^{-3}	$\mathbf{1.16 \times 10^{-3}}$
$\mathbf{9.60 \times 10^{-3}}$	8.66×10^{-4}	1.85×10^{-3}	$\mathbf{1.38 \times 10^{-3}}$
$\mathbf{1.28 \times 10^{-2}}$	8.07×10^{-4}	1.70×10^{-2}	$\mathbf{1.77 \times 10^{-3}}$

$\mathbf{k_2 = 1.20 \times 10^{-1}\ M^{-1}\ s^{-1}}$

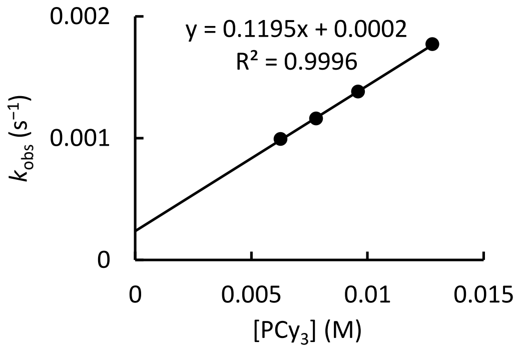

Kinetics of the reactions of ethyl buta-2,3-dienoate (**2**) with $POct_3$ in CH_2Cl_2 (at 20 °C)

first step: generation of a known concentration of $POct_3$

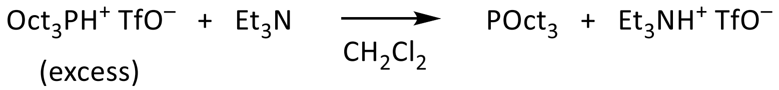

second step: reaction of $POct_3$ with **2**

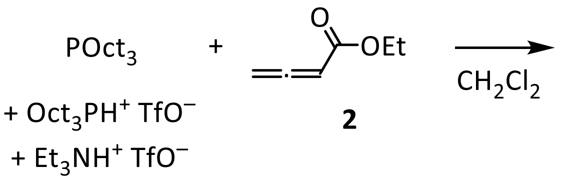

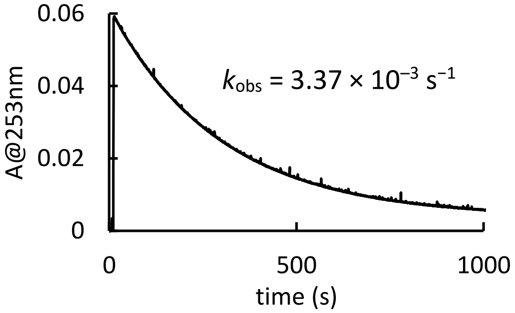

Table S25. Kinetics of the reactions of $POct_3$ and **2** (in a first step $POct_3$ was generated in CH_2Cl_2 by the deprotonation of trioctylphosphonium triflate with a known amount of NEt_3, in a second step a solution of **2** in dichloromethane was added, conventional UV-Vis method, detection at 253 nm)

$[POct_3]$ (M)	[**2**] (M)	$[HP^+Oct_3]$ (M)	k_{obs} (s^{-1})
**3.59×10^{-3}**	3.36×10^{-4}	8.48×10^{-4}	**2.06×10^{-3}**
**4.30×10^{-3}**	3.33×10^{-4}	9.17×10^{-4}	**2.80×10^{-3}**
**5.38×10^{-3}**	5.22×10^{-4}	1.27×10^{-3}	**3.37×10^{-3}**
**7.78×10^{-3}**	5.71×10^{-4}	1.66×10^{-2}	**5.18×10^{-3}**
**$k_2 = 7.24 \times 10^{-1}$ M^{-1} s^{-1}**			

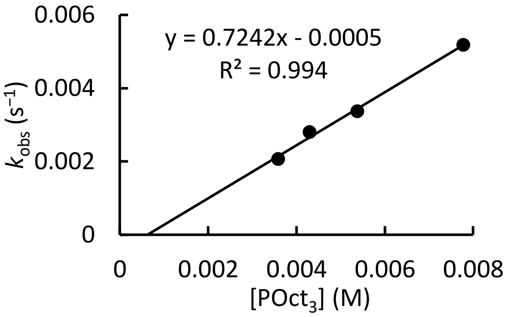

Kinetics of the reactions of ethyl buta-2,3-dienoate (**2**) with $P(1\text{-}Np)_3$ in CD_2Cl_2 (at 20 °C)

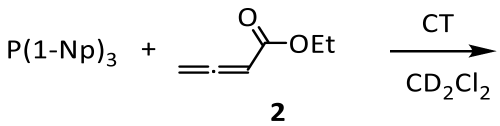

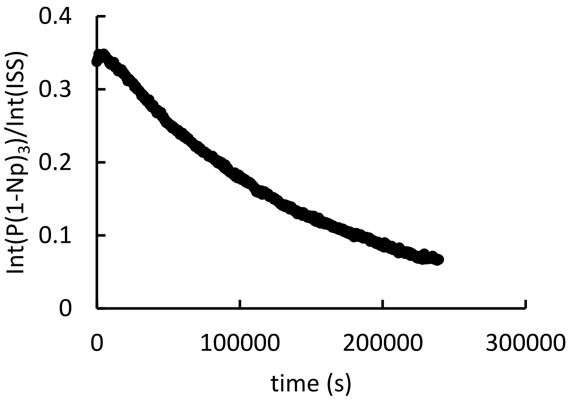

Table S26. Kinetics of the reaction of $P(1\text{-}Np)_3$ and **2** (proton source: CT, ^{31}P NMR method, monitoring of the decreasing ^{31}P NMR signal of $P(1\text{-}Np)_3$ at –34.1 ppm, in CD_2Cl_2, Ph_3PO as internal integration standard)

$[P(1\text{-}Np)_3]$ (M)	[**2**] (M)	[CT] (M)	k_{obs} (s^{-1})
3.18×10^{-2}	$\mathbf{3.15 \times 10^{-1}}$	7.96×10^{-2}	$\mathbf{2.50 \times 10^{-6}}$
3.93×10^{-2}	$\mathbf{5.12 \times 10^{-1}}$	9.14×10^{-2}	$\mathbf{4.04 \times 10^{-6}}$
4.85×10^{-2}	$\mathbf{7.30 \times 10^{-1}}$	1.54×10^{-1}	$\mathbf{5.90 \times 10^{-6}}$
4.52×10^{-2}	$\mathbf{9.08 \times 10^{-1}}$	1.38×10^{-2}	$\mathbf{7.20 \times 10^{-6}}$

$\mathbf{k_2 = 8.00 \times 10^{-6}\ M^{-1}\ s^{-1}}$

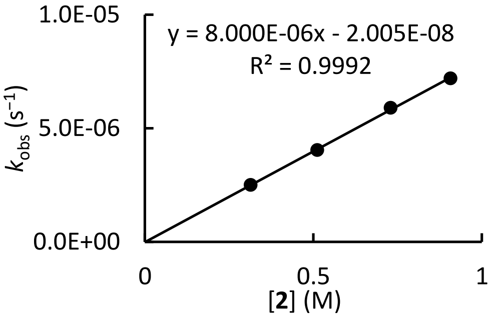

5.2.3 Kinetics of the reactions of ethyl propiolate (3) with PR_3

Kinetics of the reactions of ethyl propiolate (**3**) with $P(pfp)_3$ in CD_2Cl_2 (at 20 °C)

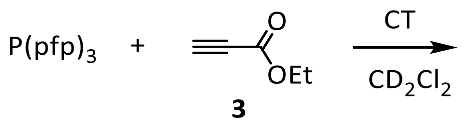

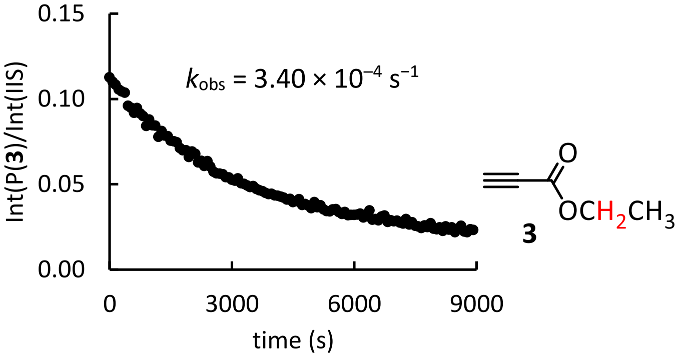

Table S27. Kinetics of the reaction of $P(pfp)_3$ and **3** (proton source: CT, ^1H NMR method, monitoring of the decreasing ^1H NMR signal of **3** at 4.23 ppm, in CD_2Cl_2, mesitylene as internal integration standard IIS)

$[P(pfp)_3]$ (M)	[**3**] (M)	[CT] (M)	k_{obs} (s^{-1})
**1.09×10^{-1}**	1.05×10^{-2}	2.07×10^{-2}	**3.40×10^{-4}**
**1.64×10^{-1}**	1.66×10^{-2}	3.38×10^{-2}	**5.07×10^{-4}**
**2.19×10^{-1}**	1.92×10^{-2}	3.98×10^{-2}	**6.75×10^{-4}**
**2.76×10^{-1}**	1.92×10^{-2}	3.98×10^{-2}	**8.14×10^{-4}**

$k_2 = 2.86 \times 10^{-3}\ M^{-1}\ s^{-1}$

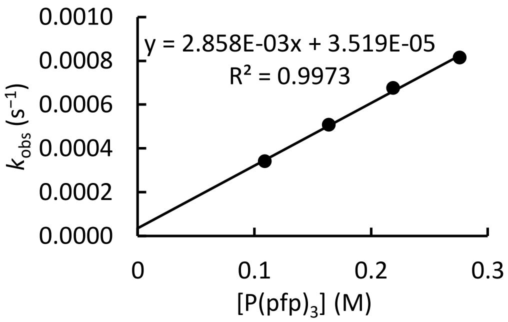

Kinetics of the reactions of ethyl propiolate (**3**) with PPh_3 in CD_2Cl_2 (at 20 °C)

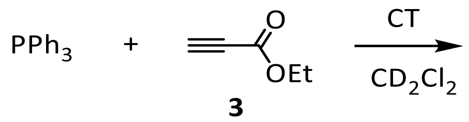

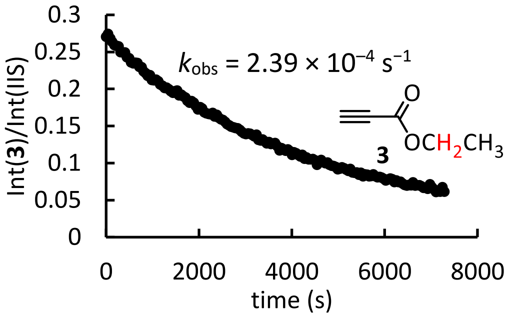

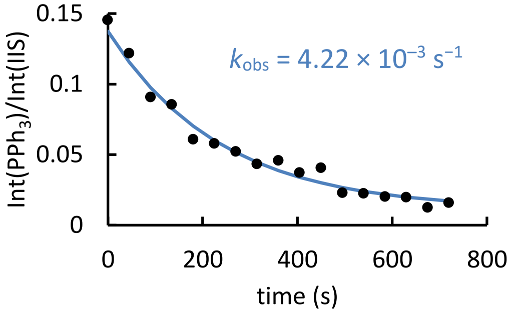

Table S28. Kinetics of the reaction of PPh_3 and **3** (proton source: CT, ^{1}H NMR method, monitoring of the decreasing ^{1}H NMR signal of **3** at 4.23 ppm, in CD_2Cl_2, mesitylene as internal integration standard IIS)

$[PPh_3]$ (M)	[**3**] (M)	[CT] (M)	k_{obs} (s^{-1})
**2.55×10^{-2}**	2.07×10^{-3}	5.59×10^{-3}	**2.39×10^{-4}**
**4.16×10^{-2}**	3.45×10^{-3}	9.31×10^{-3}	**4.09×10^{-4}**
**5.91×10^{-2}**	5.18×10^{-3}	1.30×10^{-3}	**5.82×10^{-4}**
**7.66×10^{-2}**	6.90×10^{-3}	1.86×10^{-3}	**7.72×10^{-4}**

$k_2 = 1.04 \times 10^{-2}$ M^{-1} s^{-1}

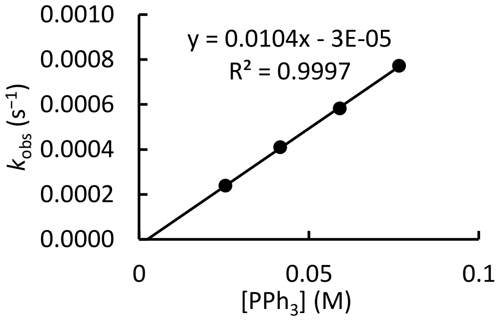

Table S29. Kinetics of the reaction of PPh_3 and **3** (proton source: CT, ^{31}P NMR method, monitoring of the decreasing ^{31}P NMR signal of PPh_3 at –5.5 ppm,[S12] in CD_2Cl_2, Ph_3PO as internal integration standard IIS)

$[PPh_3]$ (M)	[**3**] (M)	[CT] (M)	k_{obs} (s^{-1})
4.04×10^{-2}	**3.87×10^{-1}**	8.59×10^{-2}	**2.63×10^{-3}**
5.16×10^{-2}	**5.18×10^{-1}**	1.15×10^{-1}	**3.50×10^{-3}**
6.29×10^{-2}	**6.14×10^{-1}**	1.36×10^{-1}	**4.22×10^{-3}**
7.41×10^{-2}	**7.33×10^{-1}**	1.63×10^{-1}	**5.06×10^{-3}**

$k_2 = 7.05 \times 10^{-3}$ M^{-1} s^{-1}

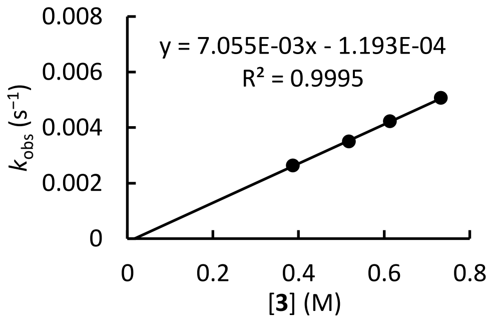

Kinetics of the reactions of ethyl propiolate (**3**) with P(ani)$_3$ in CH_2Cl_2 (at 20 °C)

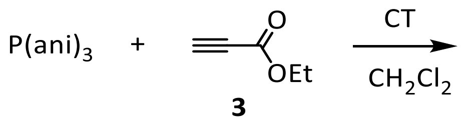

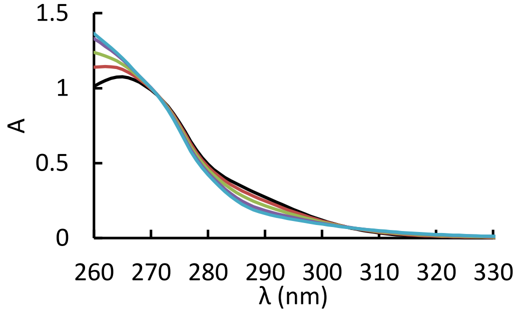

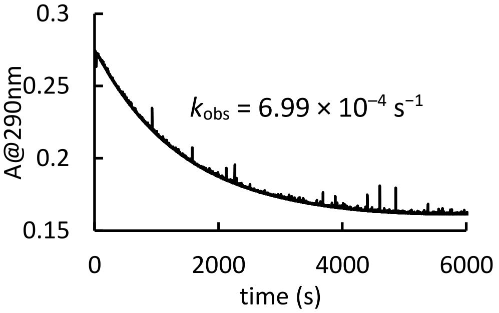

Table S30. Kinetics of the reactions of P(ani)$_3$ and **3** (proton source: CT, conventional UV-Vis method, detection at 290 nm, in CH_2Cl_2)

[P(ani)$_3$] (M)	[**3**] (M)	[CT] (M)	k_{obs} (s^{-1})
5.95×10^{-5}	$\mathbf{1.34 \times 10^{-2}}$	1.13×10^{-4}	$\mathbf{6.99 \times 10^{-4}}$
5.89×10^{-5}	$\mathbf{2.07 \times 10^{-2}}$	1.12×10^{-4}	$\mathbf{1.03 \times 10^{-3}}$
5.95×10^{-5}	$\mathbf{2.57 \times 10^{-2}}$	1.13×10^{-4}	$\mathbf{1.36 \times 10^{-3}}$
6.41×10^{-5}	$\mathbf{3.52 \times 10^{-2}}$	1.22×10^{-4}	$\mathbf{1.87 \times 10^{-3}}$

$\mathbf{k_2 = 5.45 \times 10^{-2}\ M^{-1}\ s^{-1}}$

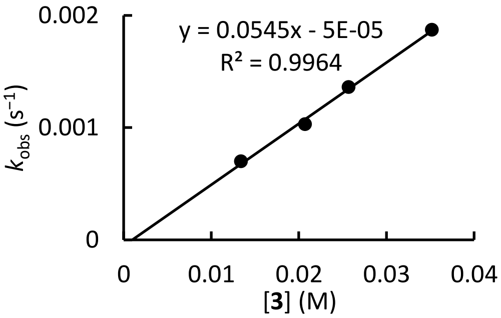

Kinetics of the reactions of ethyl propiolate (**3**) with $PMePh_2$ in CH_2Cl_2 (at 20 °C)

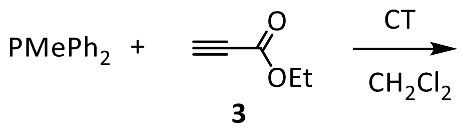

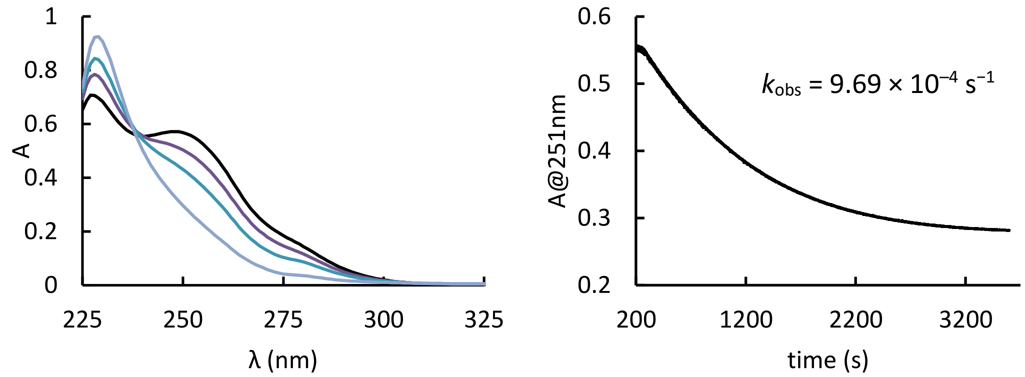

Table S31. Kinetics of the reactions of $PMePh_2$ and **3** (proton source: CT, conventional UV-Vis method, detection at 251 nm, in CH_2Cl_2)

$[PMePh_2]$ (M)	[**3**] (M)	[CT] (M)	k_{obs} (s^{-1})
1.11×10^{-4}	**1.16×10^{-2}**	1.44×10^{-4}	**6.79×10^{-4}**
1.19×10^{-4}	**1.61×10^{-2}**	2.36×10^{-4}	**9.69×10^{-4}**
1.07×10^{-4}	**1.98×10^{-2}**	1.36×10^{-4}	**1.20×10^{-3}**
1.19×10^{-4}	**2.72×10^{-2}**	2.16×10^{-4}	**1.66×10^{-3}**

$k_2 = 6.28 \times 10^{-2}$ M^{-1} s^{-1}

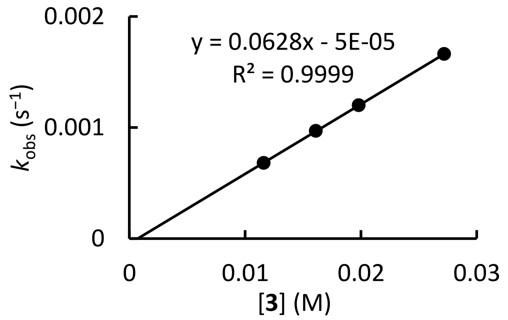

Kinetics of the reactions of ethyl propiolate (**3**) with PMe_2Ph in CH_2Cl_2 (at 20 °C)

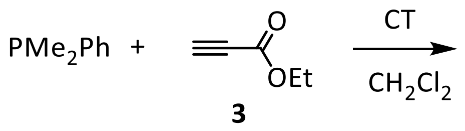

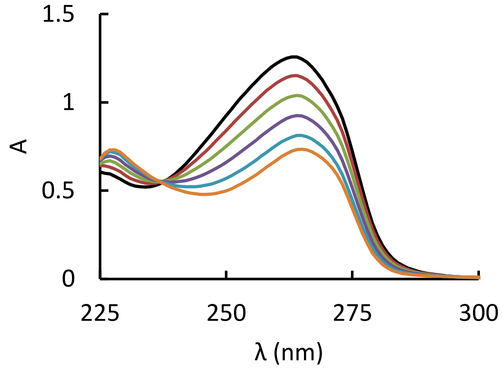

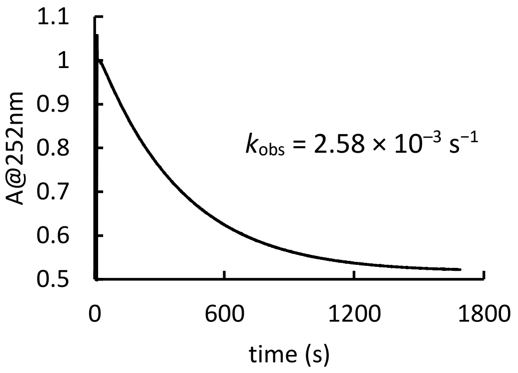

Table S32. Kinetics of the reactions of PMe_2Ph and **3** (proton source: CT, conventional UV-Vis method, detection at 252 nm, in CH_2Cl_2)

$[PMe_2Ph]$ (M)	[**3**] (M)	[CT] (M)	k_{obs} (s^{-1})
2.09×10^{-4}	$\mathbf{8.26 \times 10^{-3}}$	2.32×10^{-4}	$\mathbf{2.58 \times 10^{-3}}$
2.02×10^{-4}	$\mathbf{1.20 \times 10^{-2}}$	2.25×10^{-4}	$\mathbf{3.75 \times 10^{-3}}$
2.08×10^{-4}	$\mathbf{1.69 \times 10^{-2}}$	2.30×10^{-4}	$\mathbf{5.09 \times 10^{-3}}$
2.06×10^{-4}	$\mathbf{2.52 \times 10^{-2}}$	2.29×10^{-4}	$\mathbf{7.55 \times 10^{-3}}$
$\mathbf{k_2 = 2.92 \times 10^{-1}\ M^{-1}\ s^{-1}}$			

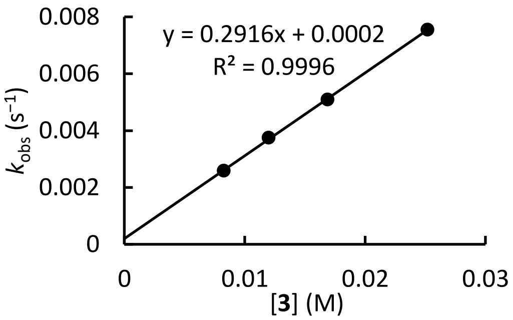

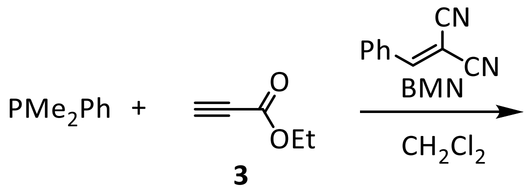

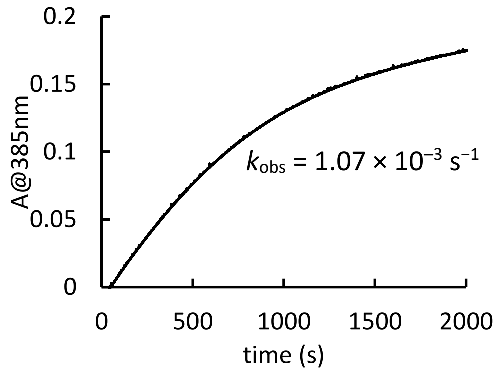

Table S33. Kinetics of the reactions of PMe_2Ph and **3** (with BMN as trapping reagent, conventional UV-Vis method, detection at 385 nm, in CH_2Cl_2)

$[PMe_2Ph]$ (M)	[**3**] (M)	[**6**] (M)	k_{obs} (s^{-1})
3.11×10^{-4}	**4.72×10^{-3}**	1.25×10^{-3}	**1.07×10^{-3}**
3.24×10^{-4}	**7.68×10^{-3}**	1.58×10^{-3}	**1.80×10^{-3}**
3.03×10^{-4}	**8.89×10^{-3}**	1.21×10^{-3}	**2.12×10^{-3}**
3.19×10^{-4}	**1.20×10^{-2}**	1.56×10^{-3}	**2.94×10^{-3}**
**$k_2 = 2.57 \times 10^{-1}\ M^{-1}\ s^{-1}$**			

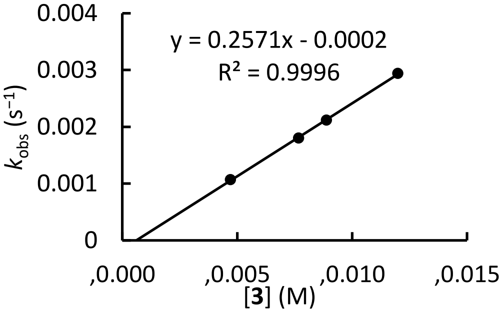

Kinetics of the reactions of ethyl propiolate (**3**) with PBu_3 in CH_2Cl_2 (at 20 °C)

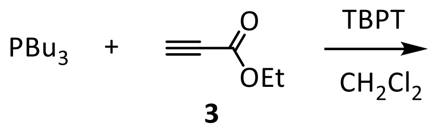

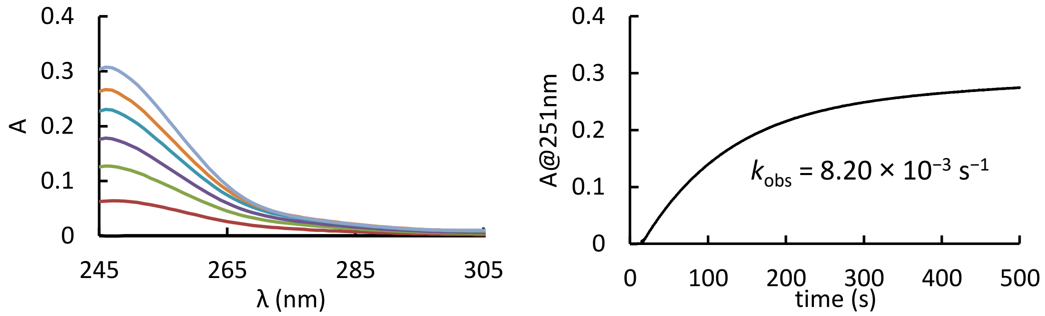

Table S34. Kinetics of the reactions of PBu_3 and **3** (proton source: TBPT, conventional UV-Vis method, detection at 251 nm, in CH_2Cl_2)

$[PBu_3]$ (M)	[**3**] (M)	[TBPT] (M)	k_{obs} (s^{-1})
**3.49×10^{-3}**	5.23×10^{-4}	1.45×10^{-3}	**3.81×10^{-3}**
**4.59×10^{-3}**	8.92×10^{-4}	2.20×10^{-3}	**4.75×10^{-3}**
**8.08×10^{-3}**	8.19×10^{-4}	1.97×10^{-3}	**8.20×10^{-3}**
**1.10×10^{-2}**	1.68×10^{-3}	2.82×10^{-3}	**1.13×10^{-2}**
**1.45×10^{-2}**	1.43×10^{-3}	3.33×10^{-3}	**1.42×10^{-2}**

$k_2 = 9.61 \times 10^{-1}$ M^{-1} s^{-1}

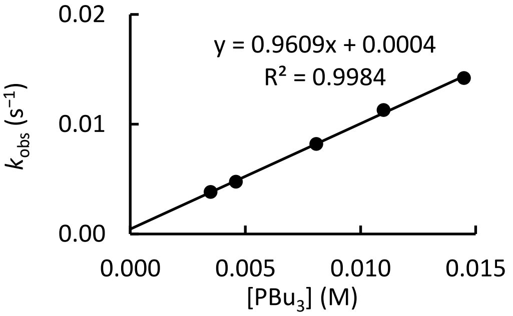

Kinetics of the reactions of ethyl propiolate (**3**) with PMe_3 in CH_2Cl_2 (at 20 °C)

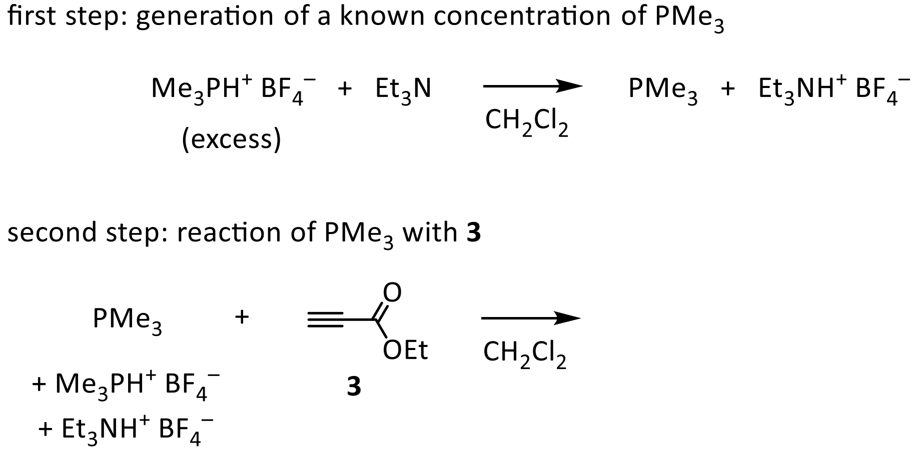

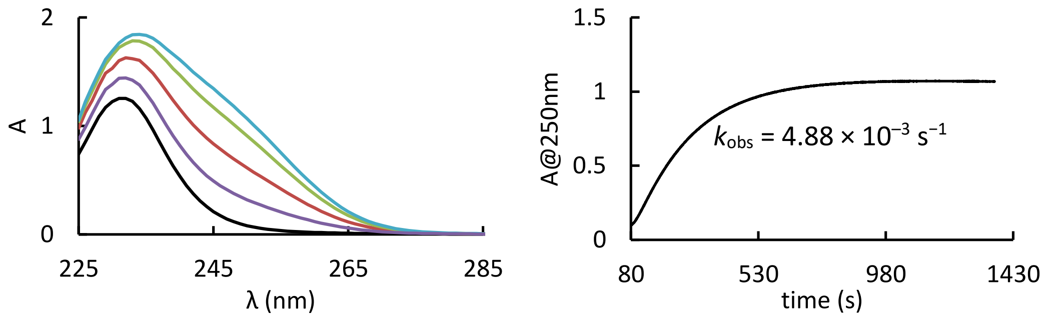

Table S35. Kinetics of the reactions of PMe_3 and **3** (in a first step PMe_3 was generated in CH_2Cl_2 by the deprotonation of trimethylphosphonium tetrafluoroborate with a known amount of NEt_3, in a second step a solution of **3** in dichloromethane was added, conventional UV-Vis method, detection at 250 nm)

$[PMe_3]$ (M)	[**3**] (M)	$[HP^+Me_3]$ (M)	k_{obs} (s^{-1})
**5.17×10^{-3}**	5.16×10^{-4}	6.31×10^{-4}	**4.88×10^{-3}**
**7.63×10^{-3}**	7.93×10^{-4}	9.32×10^{-4}	**7.15×10^{-3}**
**9.86×10^{-3}**	1.08×10^{-3}	1.11×10^{-3}	**9.53×10^{-3}**
**1.29×10^{-2}**	1.43×10^{-3}	1.45×10^{-3}	**1.22×10^{-2}**

$k_2 = 9.56 \times 10^{-1}\ M^{-1}\ s^{-1}$

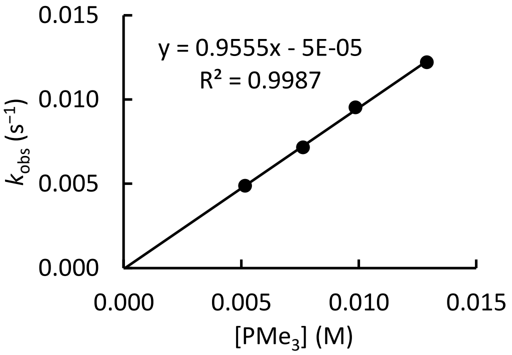

Kinetics of the reactions of ethyl propiolate (**3**) with PCy_3 in CH_2Cl_2 (at 20 °C)

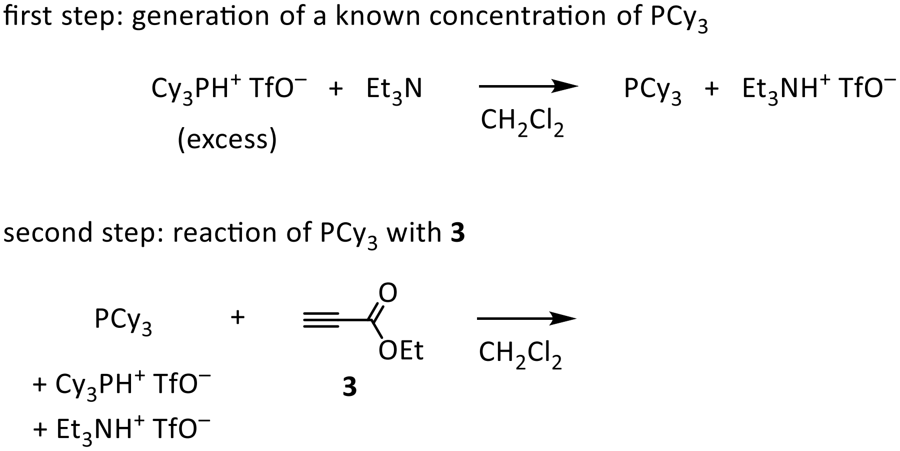

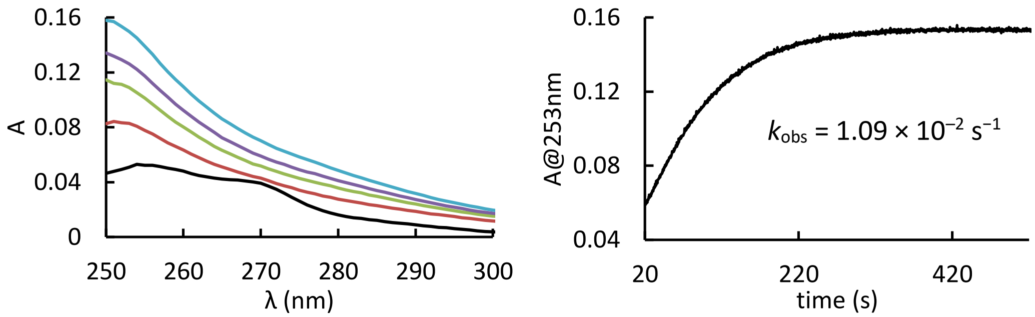

Table S36. Kinetics of the reactions of PCy_3 and **3** (in a first step PCy_3 was generated in CH_2Cl_2 by the deprotonation of tricyclohexylphosphonium triflate with a known amount of NEt_3, in a second step a solution of **3** in dichloromethane was added, conventional UV-Vis method, detection at 253 nm)

$[PCy_3]$ (M)	[**3**] (M)	$[HP^+Cy_3]$ (M)	k_{obs} (s^{-1})
**3.86×10^{-3}**	3.90×10^{-4}	4.62×10^{-3}	**5.22×10^{-3}**
**5.94×10^{-3}**	6.23×10^{-4}	7.13×10^{-3}	**8.17×10^{-3}**
**7.68×10^{-3}**	7.84×10^{-4}	1.08×10^{-2}	**1.09×10^{-2}**
**9.57×10^{-3}**	9.99×10^{-4}	1.34×10^{-2}	**1.33×10^{-2}**

$k_2 = 1.43\ M^{-1}\ s^{-1}$

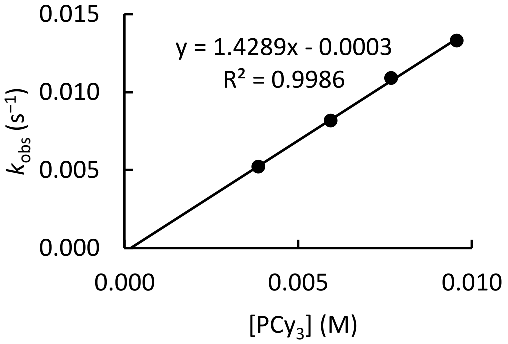

Kinetics of the reactions of ethyl propiolate (**3**) with $POct_3$ in CH_2Cl_2 (at 20 °C)

first step: generation of a known concentration of $POct_3$

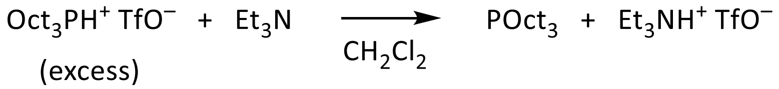

second step: reaction of $POct_3$ with **3**

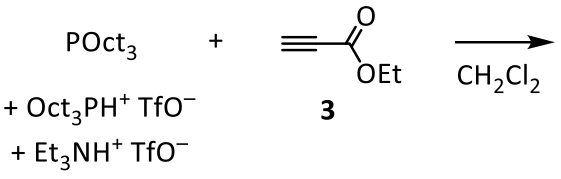

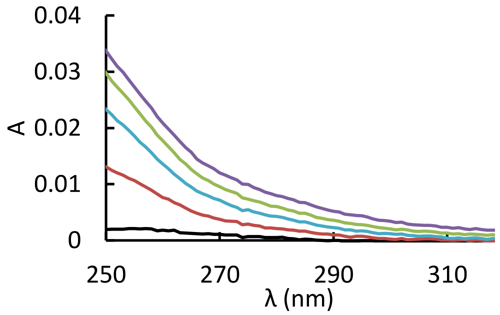

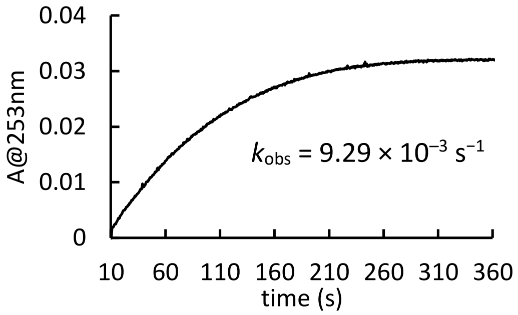

Table S37. Kinetics of the reactions of $POct_3$ and **3** (in a first step $POct_3$ was generated in CH_2Cl_2 by the deprotonation of trioctylphosphonium triflate with a known amount of NEt_3, in a second step a solution of **3** in dichloromethane was added, conventional UV-Vis method, detection at 253 nm)

$[POct_3]$ (M)	[**3**] (M)	$[HP^+Oct_3]$ (M)	k_{obs} (s^{-1})
**2.62×10^{-3}**	2.48×10^{-4}	6.18×10^{-4}	**8.02×10^{-3}**
**2.99×10^{-3}**	2.92×10^{-4}	4.00×10^{-4}	**9.29×10^{-3}**
**4.41×10^{-3}**	5.02×10^{-4}	2.40×10^{-3}	**1.29×10^{-2}**
**4.48×10^{-3}**	4.59×10^{-4}	5.99×10^{-4}	**1.33×10^{-2}**
**5.52×10^{-3}**	6.71×10^{-4}	3.00×10^{-3}	**1.59×10^{-2}**

$k_2 = 2.69\ M^{-1}\ s^{-1}$

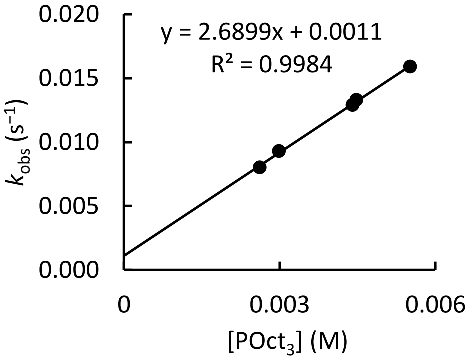

Kinetics of the reactions of ethyl propiolate (**3**) with $P(1\text{-}Np)_3$ in CD_2Cl_2 (at 20 °C)

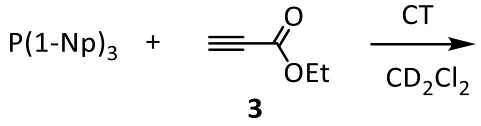

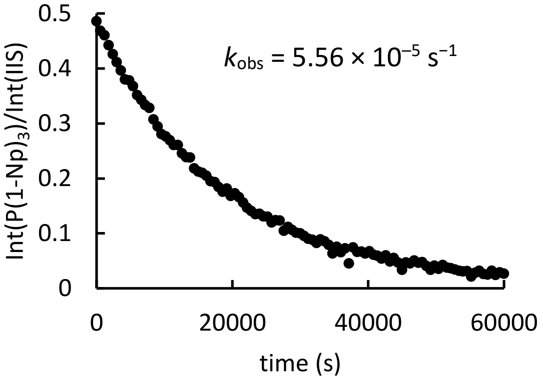

Table S38. Kinetics of the reaction of $P(1\text{-}Np)_3$ and **2** (proton source: CT, ^{31}P NMR method, monitoring of the decreasing ^{31}P NMR signal of $P(1\text{-}Np)_3$ at –34.1 ppm, in CD_2Cl_2, Ph_3PO as internal integration standard, IIS)

$[P(1\text{-}Np)_3]$ (M)	[**3**] (M)	[CT] (M)	k_{obs} (s^{-1})
3.93×10^{-2}	$\mathbf{7.14 \times 10^{-1}}$	8.96×10^{-2}	$\mathbf{5.56 \times 10^{-5}}$
3.59×10^{-2}	**1.35**	8.44×10^{-2}	$\mathbf{1.11 \times 10^{-4}}$
3.42×10^{-2}	**1.82**	7.82×10^{-2}	$\mathbf{1.49 \times 10^{-4}}$
3.59×10^{-2}	**2.19**	8.59×10^{-2}	$\mathbf{1.81 \times 10^{-4}}$

$\mathbf{k_2 = 8.46 \times 10^{-5}\ M^{-1}\ s^{-1}}$

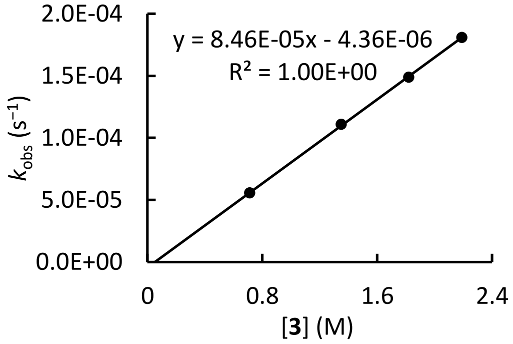

5.2.4 Kinetics of the reactions with ethenesulfonyl fluoride (ESF, 4) with PR_3

Kinetics of the reactions of ESF (**4**) with $P(pfp)_3$ in CH_2Cl_2 (at 20 °C)

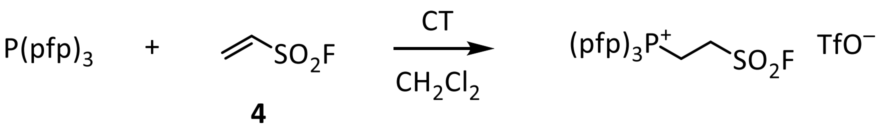

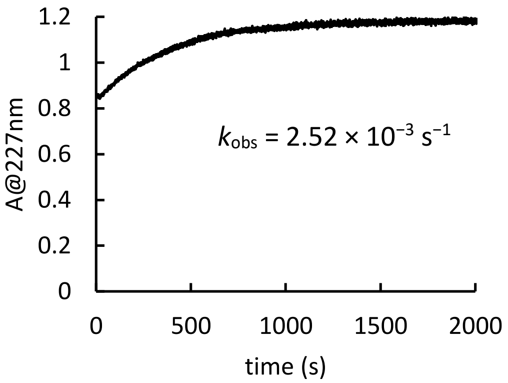

Table S39. Kinetics of the reactions of $P(pfp)_3$ and **4** (proton source: CT, conventional UV-Vis method, detection at 227 nm, in CH_2Cl_2)

$[P(pfp)_3]$ (M)	[CT] (M)	[**4**] (M)	k_{obs} (s^{-1})
1.07×10^{-4}	2.61×10^{-4}	$\mathbf{2.32 \times 10^{-3}}$	$\mathbf{1.74 \times 10^{-3}}$
1.06×10^{-4}	2.59×10^{-4}	$\mathbf{3.90 \times 10^{-3}}$	$\mathbf{2.52 \times 10^{-3}}$
9.83×10^{-5}	2.66×10^{-4}	$\mathbf{5.85 \times 10^{-3}}$	$\mathbf{3.17 \times 10^{-3}}$
9.78×10^{-5}	2.65×10^{-4}	$\mathbf{8.09 \times 10^{-3}}$	$\mathbf{4.24 \times 10^{-3}}$

$\mathbf{k_2 = 4.23 \times 10^{-1}\ M^{-1}\ s^{-1}}$

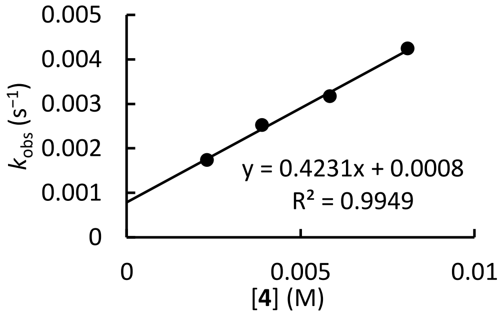

Kinetics of the reactions of ESF (**4**) with PPh_3 in CH_2Cl_2 (at 20 °C)

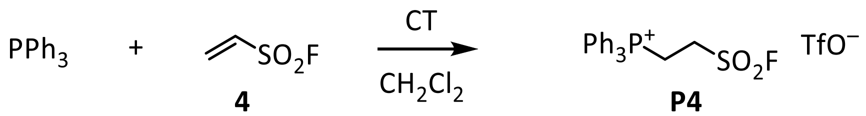

4 **P4**

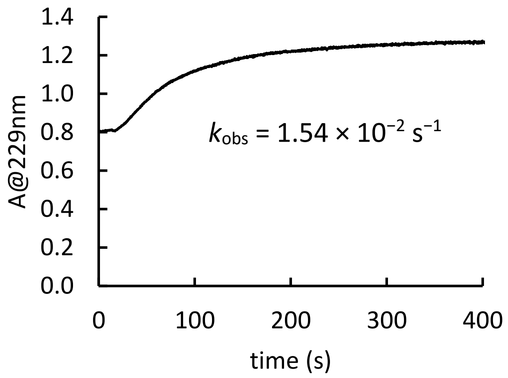

Table S40. Kinetics of the reactions of PPh_3 and **4** (proton source: CT, conventional UV-Vis method, detection at 229 nm, in CH_2Cl_2)

$[PPh_3]$ (M)	[CT] (M)	[**4**] (M)	k_{obs} (s^{-1})
1.37×10^{-4}	3.48×10^{-4}	$\mathbf{1.66 \times 10^{-3}}$	$\mathbf{7.48 \times 10^{-3}}$
1.36×10^{-4}	3.44×10^{-4}	$\mathbf{2.46 \times 10^{-3}}$	$\mathbf{1.02 \times 10^{-2}}$
1.11×10^{-4}	2.81×10^{-4}	$\mathbf{2.70 \times 10^{-3}}$	$\mathbf{1.13 \times 10^{-2}}$
1.07×10^{-4}	2.75×10^{-4}	$\mathbf{3.24 \times 10^{-3}}$	$\mathbf{1.29 \times 10^{-2}}$
1.06×10^{-4}	2.72×10^{-4}	$\mathbf{4.00 \times 10^{-3}}$	$\mathbf{1.54 \times 10^{-2}}$

$\mathbf{k_2 = 3.38\ M^{-1}\ s^{-1}}$

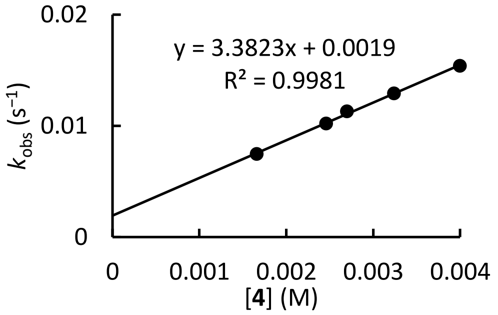

^{31}P NMR spectroscopic analysis indicated selective conversion of **4** to **P4** (δ_P = +23.5 ppm in CD_2Cl_2) under the conditions of the kinetic experiments (Figure S28).

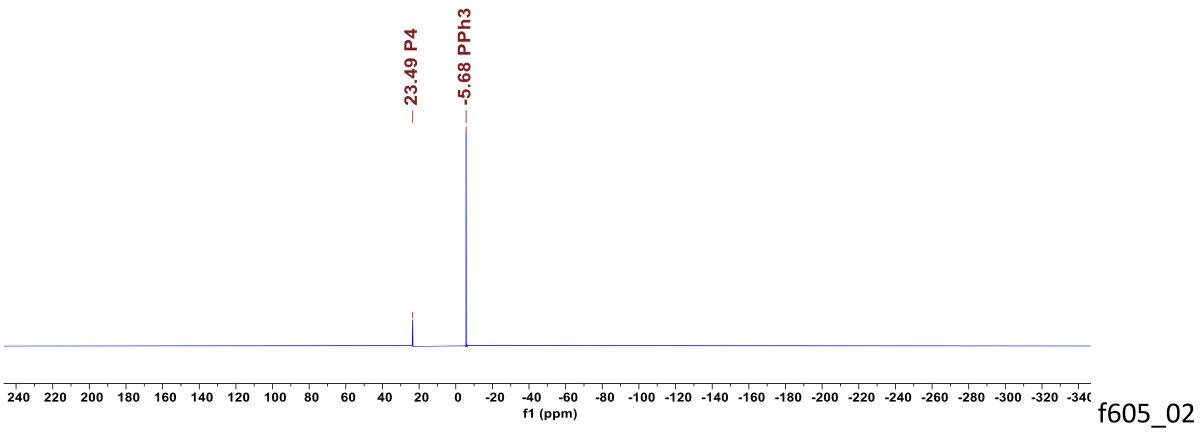

Fig. S28. ^{31}P NMR (162 MHz) spectrum of a sample with $[Ph_3P]_0$ = 108 mM, $[\mathbf{4}]_0$ =10.8 mM and $[CT]_0$ = 22.3 mM in CD_2Cl_2 after completion of their reaction.

Kinetics of the reactions of ESF (**4**) with $PMePh_2$ in CH_2Cl_2 (at 20 °C)

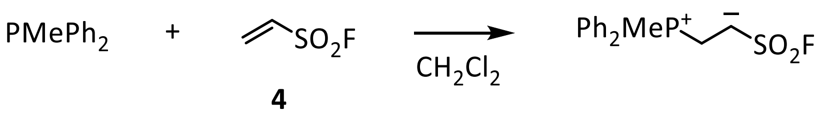

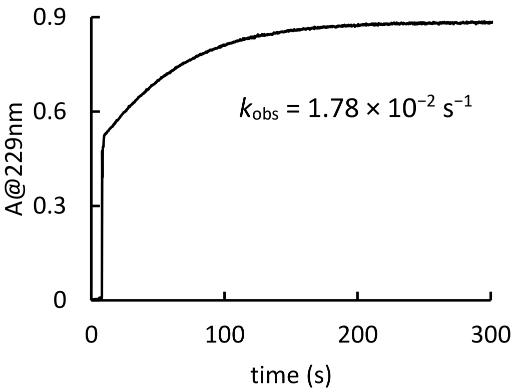

Table S41. Kinetics of the reactions of $PMePh_2$ and **4** (conventional UV-Vis method, detection at 229 nm, in CH_2Cl_2)

$[PMePh_2]$ (M)	[**4**] (M)	k_{obs} (s^{-1})
6.48×10^{-5}	$\mathbf{6.65 \times 10^{-4}}$	$\mathbf{5.38 \times 10^{-3}}$
1.13×10^{-4}	$\mathbf{1.10 \times 10^{-3}}$	$\mathbf{8.27 \times 10^{-3}}$
1.06×10^{-4}	$\mathbf{1.64 \times 10^{-3}}$	$\mathbf{1.33 \times 10^{-2}}$
1.06×10^{-4}	$\mathbf{2.19 \times 10^{-3}}$	$\mathbf{1.78 \times 10^{-2}}$
1.26×10^{-4}	$\mathbf{2.69 \times 10^{-3}}$	$\mathbf{2.31 \times 10^{-2}}$
	$\mathbf{k_2 = 8.76\ M^{-1}{\cdot}s^{-1}}$	

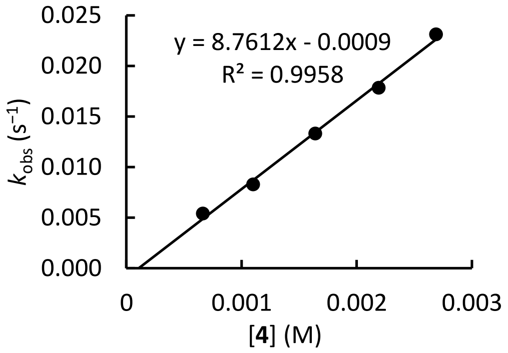

Kinetics of the reactions of ESF (**4**) with PMe_2Ph in CH_2Cl_2 (at 20 °C)

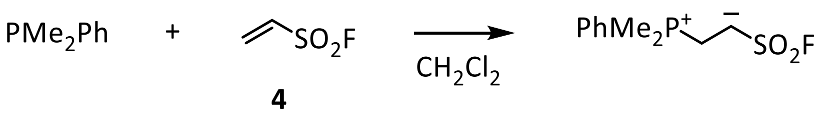

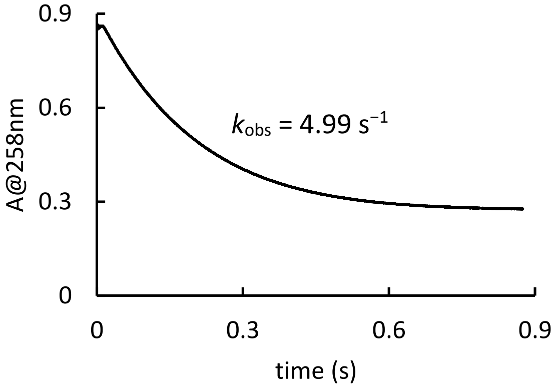

Table S42. Kinetics of the reactions of PMe_2Ph and **5** (stopped-flow method, detection at 258 nm, in CH_2Cl_2)

$[PMe_2Ph]$ (M)	[**4**] (M)	k_{obs} (s^{-1})
	**5.08×10^{-3}**	**2.05**
	**6.77×10^{-3}**	**2.83**
1.63×10^{-4}	**8.47×10^{-3}**	**3.52**
	**1.02×10^{-2}**	**4.23**
	**1.19×10^{-2}**	**4.99**

$k_2 = 4.26 \times 10^{2}\ M^{-1}\cdot s^{-1}$

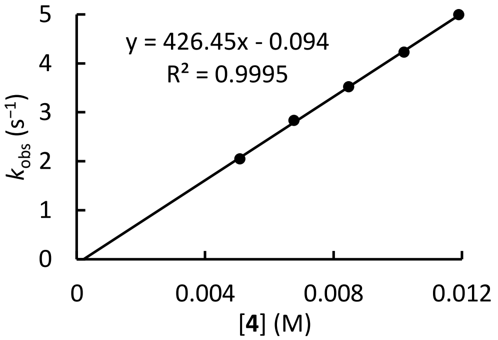

Kinetics of the reactions of ESF (**4**) with PBu_3 in CH_2Cl_2 (at 20 °C)

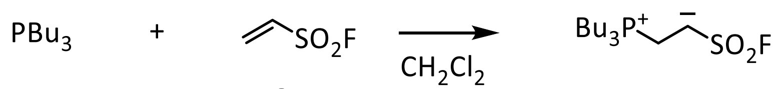

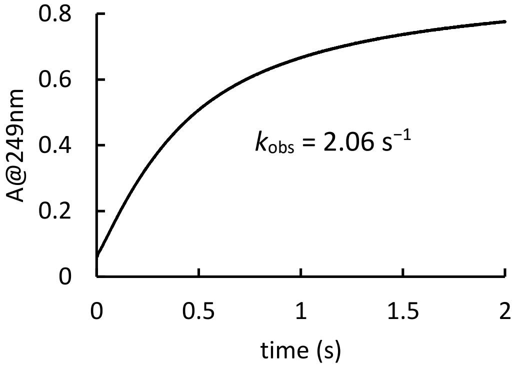

Table S43. Kinetics of the reactions of PBu_3 and **4** (stopped-flow method, detection at 249 nm, in CH_2Cl_2)

$[PBu_3]$ (M)	[**4**] (M)	k_{obs} (s^{-1})
3.21×10^{-4}	**3.11×10^{-3}**	**2.06**
	**4.14×10^{-3}**	**2.88**
	**5.18×10^{-3}**	**3.61**
	**6.21×10^{-3}**	**4.45**
	**8.28×10^{-3}**	**6.20**
	**9.32×10^{-3}**	**6.99**

$k_2 = 7.99 \times 10^2$ $M^{-1} \cdot s^{-1}$

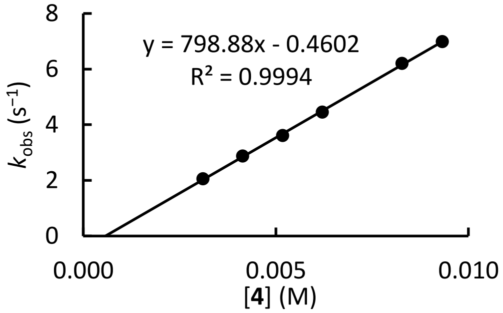

Kinetics of the reactions of ESF (**4**) with PMe_3 in CH_2Cl_2 (at 20 °C)

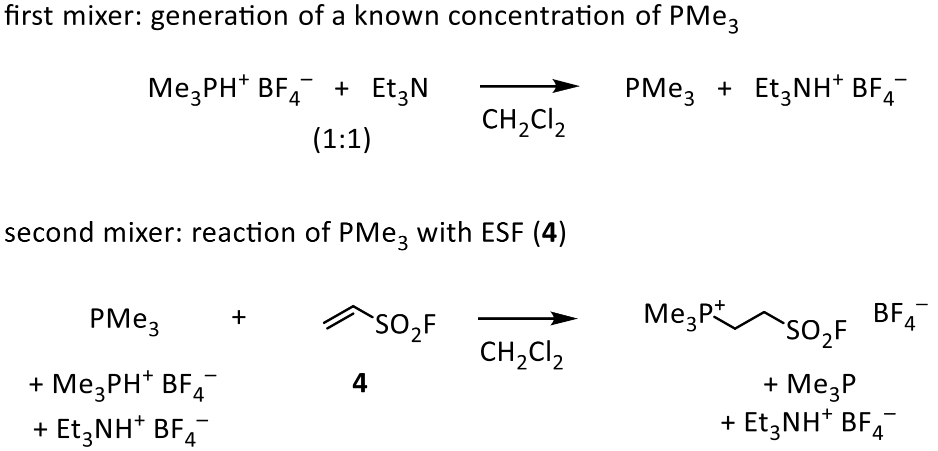

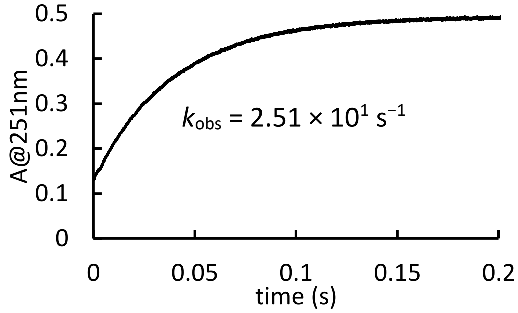

Table S44. Kinetics of the reactions of PMe_3 and **4** (PMe_3 was generated by the deprotonation of trimethylphosphonium tetrafluoroborate with a stoichiometric amount of NEt_3 in the first step of the sequential mixing stopped-flow method, detection at 251 nm, in CH_2Cl_2)

$[PMe_3]$ (M)	[**4**] (M)	k_{obs} (s^{-1})
	**1.37×10^{-2}**	**2.51×10^{1}**
	**2.05×10^{-2}**	**4.01×10^{1}**
1.42×10^{-3}	**2.74×10^{-2}**	**5.37×10^{1}**
	**3.42×10^{-2}**	**6.82×10^{1}**
	**4.11×10^{-2}**	**8.21×10^{1}**

$k_2 = 2.07 \times 10^{3}$ M^{-1} s^{-1}

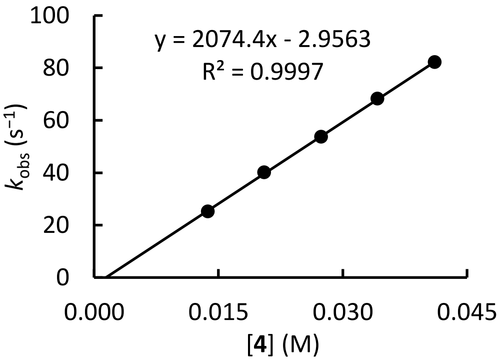

5.2.5 Kinetics of the reactions with ethyl 2-butynoate (5) with PR_3

Kinetics of the reactions of ethyl 2-butynoate (**5**) with PPh_3 in CD_2Cl_2 (at 20 °C)

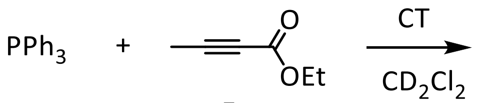

5

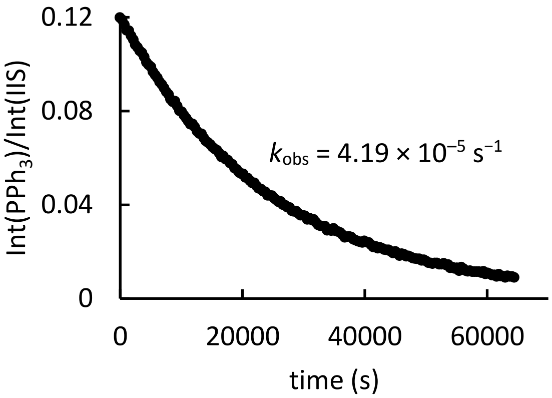

Table S45. Kinetics of the reaction of PPh_3 and **5** (proton source: CT, ^{31}P NMR method, monitoring of the decreasing ^{31}P NMR signal of PPh_3 at –5.5 ppm,[S12] in CD_2Cl_2, Et_3PO as internal integration standard, IIS)

$[PPh_3]$ (M)	[**5**] (M)	[CT] (M)	k_{obs} (s^{-1})
8.77×10^{-2}	**8.92×10^{-1}**	1.14×10^{-1}	**1.71×10^{-5}**
1.35×10^{-1}	**1.35**	1.73×10^{-1}	**2.73×10^{-5}**
1.73×10^{-1}	**1.70**	2.61×10^{-1}	**3.37×10^{-5}**
2.06×10^{-1}	**2.09**	3.49×10^{-1}	**4.19×10^{-5}**

$k_2 = 2.05 \times 10^{-5}$ M^{-1} s^{-1}

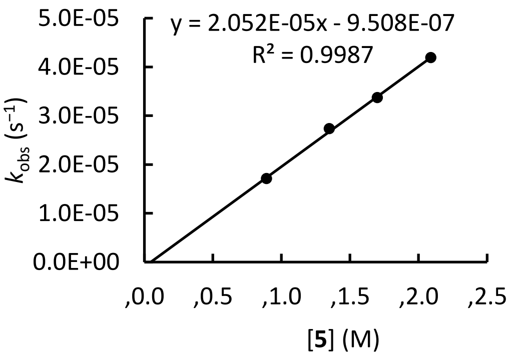

Kinetics of the reactions of ethyl 2-butynoate (**5**) with PBu_3 in CD_2Cl_2 (at 20 °C)

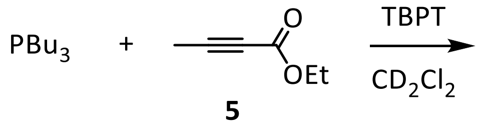

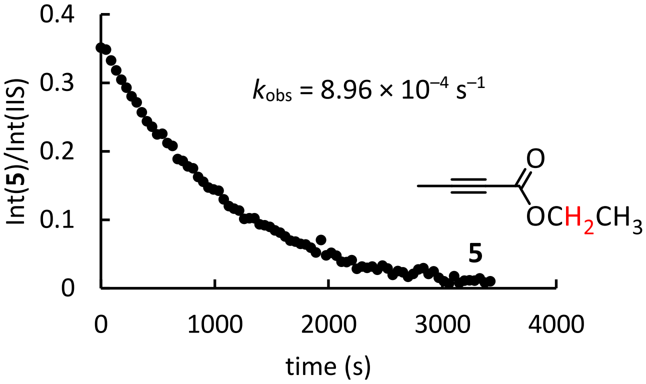

Table S46. Kinetics of the reaction of PBu_3 and **5** (proton source: TBPT, ^1H NMR method, monitoring of the decreasing ^1H NMR signal of 4.16 ppm, residual CH_2Cl_2 as internal integration standard, IIS)

$[PBu_3]$ (M)	[**5**] (M)	[TBPT] (M)	k_{obs} (s^{-1})
**5.34×10^{-2}**	2.23×10^{-3}	5.77×10^{-3}	**3.16×10^{-4}**
**8.50×10^{-2}**	4.46×10^{-3}	1.15×10^{-2}	**4.75×10^{-4}**
**1.16×10^{-1}**	4.46×10^{-3}	1.15×10^{-2}	**6.91×10^{-4}**
**1.57×10^{-1}**	4.46×10^{-3}	1.15×10^{-2}	**8.96×10^{-4}**

$k_2 = 5.71 \times 10^{-3}\ M^{-1}\ s^{-1}$

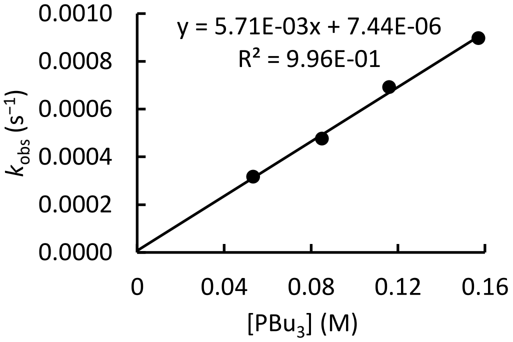

6 Theoretical Analysis

6.1 Prelude: Quantum-Chemical Method Validation

Previous reports on the results of the quantum-chemical investigation of the PMe_3 addition to methyl allenoate, which used the CPCM(benzene)/B3LYP/6-31G(d)//B3LYP/6-31G(d) level of theory[S13] or the CPCM(benzene)/B3LYP/6-31G+(d)//B3LYP/6-31G+(d) level of theory,[S14] have been ambiguous. We, therefore, started by evaluating the influence of various basis sets, electronic structure methods, and solvation models on the results of the quantum-chemical calculations.

As shown in Scheme S1, the addition of PMe_3 to ethyl allenoate (**2**) was used as a model reaction, for which we calculated the reaction energies (ΔG_{add}). Additionally, we analysed the differences in P–O and P–C distances in the zwitterionic product.

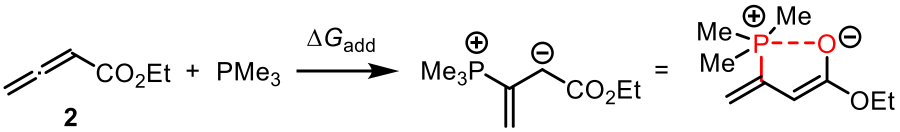

Scheme S1. Reaction of PMe_3 with the allenoate **2** to form a zwitterionic adduct. The considered P-O and P-C atom distances are highlighted in red.

Basis Set. Table S47 gathers ΔG_{add} values computed by using the density functional theory (DFT) functional B3LYP[S15] in gas phase with different basis sets along with the P–O and P–C bond lengths in the optimised zwitterionic product.

Table S47. Reaction energies (ΔG_{add}) for the addition of PMe_3 to ethyl allenoate (as defined in Scheme S1). Computed with the DFT functional B3LYP. P–O and P–C atom distances of the zwitterionic product.

Basis set	ΔG_{add} (kJ mol^{-1})	P–C (Å)	P–O (Å)
6-31G(d)	+47.7	1.833	2.559
6-31G(d)[a]	+44.6[a]	1.824[a]	2.801[a]
6-31G(d,p)	+45.6	1.833	2.574
6-31+G(d)	+47.9	1.833	2.658
6-31+G(d)[b]	+48.1[b]	1.836[b]	2.514[b]
6-311++G(2df,2p)	+40.3	1.821	2.654
def2-SVP	+33.2	1.827	2.800
def2-TZVPP	+38.1	1.818	2.645
def2-QZVPP	+38.2	1.817	2.650

[a] As reported by Dudding *et al*. for the PMe_3 addition to methyl allenoate (ref. S13). [b] As reported by Yu *et al*. for the PMe_3 addition to methyl allenoate (ref. S14).

The studied double-ζ basis sets from the Pople family[S16] yield very similar results for ΔG_{add} with values of +47.7 kJ mol^{-1} for 6-31G(d), +45.6 kJ mol^{-1} for 6-31G(d,p), and +47.9 kJ mol^{-1} for 6-31+G(d). Additionally, all these basis sets predict the same P–C bond length of 1.833 Å. In contrast, using the triple-ζ basis set 6-311++G(2df,2p) from the Pople family results in a noticeably lower ΔG_{add} of +40.3 kJ mol^{-1} and a slightly shorter P–C bond of 1.821 Å. The Karlsruhe[S17] triple-ζ basis set def2-TZVPP gives a ΔG_{add} of +38.1 kJ mol^{-1}, and the (much larger) quadruple-ζ basis set def2-QZVPP yields an almost identical ΔG_{add} of +38.2 kJ mol^{-1}, both of which are close to the results obtained with the 6-311++G(2df,2p) basis set. The P–C bond length for these basis sets are also similar to 6-311++G(2df,2p), with def2-TZVPP giving 1.818 Å and def2-QZVPP showing 1.817 Å. The small double-ζ basis set def2-SVP from the Karlsruhe family predicts a much smaller value at ΔG_{add} = +33.2 kJ mol^{-1}, possibly due to basis set superposition (BSSE) errors.

In general terms, computed ΔG_{add} values as well as key structural features of reactants and products could be reproduced (within ±1 kcal mol^{-1}) consistent with the findings previously reported in refs. S13 and S14. Thus, the difference of PMe_3 reacting with either methyl or ethyl allenoate seems to be minor. That the P–O distance as the most sensitive geometrical parameter shows significant variations for seemingly similar theoretical methods may reflect differences in the default settings of the program releases used for the computations (such as DFT integration grid size or convergence criteria for optimisations).

Electronic structure method. To investigate the influence of the electronic structure method we computed the same parameters as before with different methods (B3LYP, D3[S18]-B3LYP, MN15,[S19] ωb97X-D2,[S20] B2PLYP,[S21] and MP2). The def2-TZVPP basis set was used for all calculations, which were done in gas phase (Table S48).

The B3LYP functional predicts a ΔG_{add} value of +38.1 kJ mol^{-1}, while the D3-corrected B3LYP method provides a significantly different value of –4.5 kJ mol^{-1}. Modern DFT functionals that account for dispersion effects predict a thermoneutral or even exergonic process, with MN15 yielding –13.5 kJ mol^{-1} and ωB97X-D2 giving +0.5 kJ mol^{-1}. B2PLYP, which includes a parametrised combination of MP2 correlation energies and components of the B3LYP hybrid functional, predicts a reaction energy (ΔG_{add}) of +14.5 kJ mol^{-1}. Additional consideration of empirical dispersion corrections as in D3-B2PLYP predicts a slightly exergonic reaction (ΔG_{add} = –6.9 kJ mol^{-1}), in agreement with other dispersion-corrected DFT functionals. MP2 theory, which overestimates dispersion effects, predicts a ΔG_{add} value of –30.0 kJ mol^{-1}.

Table S48. Reaction energies (ΔG_{add}) for the addition of PMe_3 to ethyl allenoate (see Scheme S1). Computed with the different electronic structure methods and the def2-TZVPP basis set. P–O and P–C distances in the zwitterionic product. RHF refers to the reference energy in MP2 calculations.

Method	ΔG_{add} (kJ mol^{-1})	P-C (Å)	P-O (Å)
B3LYP	+38.1	1.818	2.645
D3-B3LYP	–4.5	1.812	2.605
MN15	–13.5	1.802	2.349
ωb97X-D2	+0.5	1.809	2.619
B2PLYP	+14.5	1.817	2.403
D3-B2PLYP	–6.9	1.813	2.410
MP2	–30.0	1.815	2.193
(RHF)[a]	(+81.9)[a]	(1.815)[a]	(2.193)[a]

[a] Based on MP2-optimised geometries.

Solvation Model. We also investigated the impact of two commonly used implicit solvation models, CPCM[S22] and SMD.[S23] For all calculations in Table S49 DCM was chosen as solvent and the def2-TZVPP basis set was used in combination with different electronic structure methods, if not stated otherwise.

Table S49. Reaction energies (ΔG_{add}) for the addition of PMe_3 to ethyl allenoate (see Scheme S1). Computed with the different electronic structure methods and the def2-TZVPP basis set in combination with CPCM(DCM) or SMD(DCM). P–O and P–C distances in the zwitterionic product. RHF refers to the reference energy in MP2 calculations.

Method	CPCM(DCM)			SMD(DCM)		
	ΔG_{add} (kJ mol^{-1})	P-C (Å)	P-O (Å)	ΔG_{add} (kJ mol^{-1})	P-C (Å)	P-O (Å)
B3LYP	+21.2	1.816	3.027	+19.4	1.820	3.036
B3LYP[a]	0.0[a]	1.824[a]	2.801[a]	--	--	--
B3LYP[b]	+40.6[b]	1.836[b]	2.514[b]	--	--	--
D3-B3LYP	–20.1	1.810	2.988	–21.9	1.813	2.995
MN15	–23.2	1.796	2.755	–26.0	1.799	2.779
ωb97X-D2	–16.9	1.807	3.011	–17.6	1.810	3.015
B2PLYP	–2.8	1.808	2.987	–4.8	1.812	2.995
D3-B2PLYP	–23.7	1.806	2.967	–25.1	1.809	2.976
MP2	–45.2	1.798	2.933	–46.2	1.801	2.942
(RHF)[c]	(+48.7)[c]	(1.798)[c]	(2.933)[c]	(+46.5)[c]	(1.801)[c]	(2.942)[c]

[a] As reported by Dudding *et al*. for the PMe_3 addition to methyl allenoate (ref. S13). The 6-31G(d) basis set was used. Structures optimised in gas phase. Solvation energies were obtained by single point energies, using benzene as solvent. Level of theory: CPCM(benzene)/B3LYP/6-31G(d)//B3LYP/6-31G(d). [b] As reported by Yu *et al*. for the PMe_3 addition to methyl allenoate (ref. S14). The 6-31G+(d) basis set was used. Structures optimised in gas phase. Solvation energies were obtained by single point energies, using benzene as solvent. Level of theory: CPCM(benzene)/B3LYP/6-31G+(d)//B3LYP/6-31G+(d). [c] Based on MP2-optimised geometries.

Compared to the ΔG_{add} computed in the gas phase (see Table S48), both CPCM and SMD solvation models exhibit a similar trend: the Gibbs energies for adduct formation, ΔG_{add}, are approx. 10–

20 kJ mol^{-1} lower than in the gas phase, making the reaction more exergonic and highlighting the solvent's stabilising effect on zwitterions. The difference between SMD and CPCM is insignificant, with SMD producing slightly lower ΔG_{add} values by about 1–3 kJ mol^{-1}. Additionally, solvation significantly influences the geometry of the zwitterion and P–O bond distances in the gas phase are approximately 0.4–0.6 Å shorter compared to those in structures optimised with a solvation model. The P–C bond distances are very similar for gas phase and optimisation with a solvation model. For DFT methods, the difference in P–C bond lengths between gas-phase and solvated geometries is only 0.001–0.008 Å. For MP2, the differences are slightly more pronounced, ranging from 0.014–0.017 Å. However, both DFT and MP2 methods exhibit the same trend: P–C bonds are slightly longer in the gas phase compared to those optimised with a solvation model.

Reported ΔG_{add} values by Yu and conworkers obtained at the CPCM(benzene)/B3LYP/6-31G+(d) // B3LYP/6-31G+(d) level theory (ref. S14) are significantly larger (19.4 kJ mol^{-1}) than the ones obtained at the CPMC(DCM)/B3LYP/def2-TZVPP level theory. Regarding the small difference of dielectric constants ε (as implemented in Gaussian16)[S24] of benzene (ε = 2.2706) and DCM (ε = 8.93) the influence of the chosen solvent appears to be minor. Besides the difference in the basis set (see above), the differently calculated ΔG_{add} can be attributed to the differences in the optimised geometries. Dudding and coworkers reported a thermoneutral reaction with ΔG_{add} = 0.0 kJ mol^{-1} in ref. S13, and we were unable to reproduce this ΔG_{add} from data given in the supplemental information of ref. S13.

Conclusions from the quantum-chemical method validation

- A triple-ζ basis set is necessary, at least for energies.
- Dispersion effects play a key role in the studied system. B3LYP without any dispersion corrections does not provide satisfying results. Hence, the use of electronic structure methods which account for dispersion effects is unavoidable.
- Solvation strongly influences the stability of the formed zwitterion and its geometrical properties. Therefore, accounting for solvation effects is already necessary for geometry optimisations. SMD and CPCM provide very similar results.

6.2 Computational Methods

Conformational space of all molecules was initially explored using Conformer-Rotamer Ensemble Sampling Tool (CREST)[S25] with the GFN2-xTB method.[S26] Geometry optimisations have been performed with a combination of the MN15 functional[S19] and the def2-TZVPP basis set.[S17] The nature of all stationary points (minima and transition states) was verified through the computation of the vibrational frequencies. Solvation effects have been considered by applying the SMD (DCM or H_2O for pK_{aH} values) solvation model[S23] to both geometries and energies. Density functional theory (DFT) calculations have been performed with the Gaussian 16 program package.[S24] Reaction barriers and reaction energies of phosphine additions to Michael acceptors and studied pronation reactions have been subject to thermochemical corrections for entropy and enthalpy. The Grimme method for entropy[S27] and the Head-Gordon enthalpy correction[S28] was applied by using Goodvibes.[S29] Free energies in solution have been corrected to a reference state of 1 mol/l at 298.15 K through addition of RTln(24.46) = +7.925 kJ mol^{-1} (= 0.0030185 Hartree) to the gas phase (1 atm) free energies. The value for R = 8.31451 J K^{-1} mol^{-1}. NBO charges[S30,S31] were calculated from the same level of theory as used before. The reaction path for energy strain analysis was explored by employment of the intrinsic reaction coordinate (IRC) method. DLPNO-CCSD(T)[S32,S33] calculations were carried out using ORCA 5.03.[S34] Solvation Energies were determined by subtracting E_{tot} in gas phase and E_{tot} with applied SMD model at the DFT level of theory. Molecular structures were visualised using *CYLview*.[S35]

6.3 Computational Results

6.3.1 Michael acceptors

Table S50. Computed properties of Michael acceptors in dichloromethane. Entropic contributions have been corrected to the reference state. ΔH* refers to thermal correction to enthalpy with the Head-Gordon correction and ΔG* to the thermal correction to Gibbs Free Energy including the Grimme method. Level of theory: SMD(DCM)/MN15/def2-TZVPP. Energies are given in Hartree

Michael acceptor	Conformers	E_{tot}	ΔH	ΔH*	ΔG	ΔG*
	1_1	-345.533372880	0.135829	0.131964	0.094407	0.094171
**1**	1_2	-345.532930913	0.135857	0.132028	0.094699	0.094445
	1_3	-345.532729436	0.135807	0.131940	0.094269	0.093971
	2_1	-383.571065782	0.140830	0.136721	0.096785	0.096446
**2**	2_2	-383.570935499	0.140797	0.136657	0.096596	0.096457
	2_3	-383.570654104	0.140882	0.136906	0.097208	0.096951
	3_1	-344.277940118	0.111749	0.108008	0.070987	0.070717
**3**	3_2	-344.277531935	0.111808	0.108138	0.071468	0.071191
	3_3	-344.273051416	0.111670	0.108003	0.070951	0.070663

6.3.2 Phosphines

Table S51. Computed properties of phosphines in dichloromethane. Entropic contributions have been corrected to the reference state. Δ*H** refers to thermal correction to enthalpy with the Head-Gordon correction and Δ*G** to the thermal correction to Gibbs Free Energy including the Grimme method. Level of theory: SMD(DCM)/MN15/def2-TZVPP. Energies are given in Hartree

Phosphine	Conformers	E_{tot}	Δ*H*	Δ*H**	Δ*G*	Δ*G**
PMe_3	PMe3	-460.878551867	0.123007	0.119852	0.086256	0.086111
$PPhMe_2$	PPhMe2_1	-652.451020029	0.180444	0.176684	0.135409	0.135358
	PPhMe2_2	-652.450587011	0.180476	0.176650	0.135216	0.135118
PPh_2Me	PPh2Me_1	-844.023613292	0.237453	0.232768	0.183276	0.183894
PPh_3	PPh3_1	-1035.59514513	0.294399	0.288508	0.230952	0.232221
$P(ani)_3$	Pani3_1	-1378.95509153	0.400061	0.392757	0.316755	0.319443
	Pani3_2	-1378.95525294	0.400148	0.392890	0.318901	0.320613
	Pani3_3	-1378.95526010	0.400099	0.392856	0.318807	0.320605
$P(pfp)_3$	Ppfp3_1	-1333.22799765	0.271939	0.265699	0.202072	0.203742
PCy_3	PCy3_1	-1046.42327676	0.505120	0.499506	0.438444	0.438044
	PCy3_2	-1046.42522543	0.505001	0.499260	0.437866	0.437488
	PCy3_3	-1046.42324061	0.505103	0.499419	0.438030	0.437734
PBu_3	PBu3_1	-814.353157457	0.391101	0.384320	0.321583	0.321913
	PBu3_2	-814.354991360	0.391511	0.385688	0.326725	0.326638
	PBu3_3	-814.354991340	0.391511	0.385687	0.326721	0.326635

Table S52. Computed properties of phosphines in water. Entropic contributions have been corrected to the reference state. Δ*H** refers to thermal correction to enthalpy with the Head-Gordon correction and Δ*G** to the thermal correction to Gibbs Free Energy including the Grimme method. Level of theory: SMD(H_2O)/MN15/def2-TZVPP. Energies are given in Hartree

Phosphine	Conformers	E_{tot}	Δ*H*	Δ*H**	Δ*G*	Δ*G**
PMe_3	PMe3_H2O	-460.873204485	0.123017	0.119864	0.086300	0.086156
$PPhMe_2$	PPhMe2_1_H2O	-652.441458364	0.180549	0.176795	0.135570	0.135528
	PPhMe2_2_H2O	-652.440766445	0.180469	0.176628	0.135119	0.135012
PPh_2Me	PPh2Me_1_H2O	-844.009476531	0.237571	0.232902	0.183632	0.184171
PPh_3	PPh3_1_H2O	-1035.576260530	0.294548	0.288664	0.230790	0.232236
$P(ani)_3$	Pani3_1_H2O	-1378.937122370	0.400424	0.393182	0.318893	0.320785
	Pani3_2_H2O	-1378.937349850	0.400439	0.393173	0.319154	0.320894
	Pani3_3_H2O	-1378.937314140	0.400390	0.393150	0.319275	0.321016
$P(pfp)_3$	Ppfp3_1_H2O	-1333.208982210	0.272004	0.265777	0.202128	0.203821
PCy_3	PCy3_1_H2O	-1046.404023460	0.505167	0.499578	0.438636	0.43821
	PCy3_2_H2O	-1046.406062040	0.505064	0.499348	0.438054	0.43766
	PCy3_3_H2O	-1046.404132810	0.505178	0.499524	0.438257	0.437945
PBu_3	PBu3_1_H2O	-814.336223256	0.391133	0.384371	0.321801	0.322046
	PBu3_2_H2O	-814.338970788	0.391504	0.385759	0.327033	0.326901
	PBu3_3_H2O	-814.337006698	0.391463	0.385306	0.325251	0.325301
$POct_3$	POct3_1_H2O	-1285.637057420	0.744038	0.73784	0.648090	0.64818
	POct3_2_H2O	-1285.638311120	0.744288	0.738466	0.650005	0.650092
	POct3_3_H2O	-1285.632491300	0.744184	0.737644	0.644270	0.645378

6.3.3 Protonated phosphines

Table S53. Computed properties of protonated phosphines in water. Entropic contributions have been corrected to the reference state. Δ*H** refers to thermal correction to enthalpy with the Head-Gordon correction and Δ*G** to the thermal correction to Gibbs Free Energy including the Grimme method. Level of theory: SMD(H_2O)/MN15/def2-TZVPP. Energies are given in Hartree

PR_3H^+	Conformers	E_{tot}	Δ*H*	Δ*H**	Δ*G*	Δ*G**
PMe_3H^+	PMe3H	-461.338531264	0.131654	0.131497	0.097517	0.097352
$PPhMe_2 H^+$	PPhMe2H_1	-652.899862875	0.188775	0.187987	0.146179	0.146167
	PPhMe2H_2	-652.899917179	0.188739	0.187868	0.146022	0.145865
PPh_2MeH^+	PPh2MeH_1	-844.461706038	0.245841	0.244078	0.193570	0.194464
PPh_3H^+	PPh3H_1	-1036.023781070	0.302949	0.300155	0.242118	0.243429
$P(ani)_3H^+$	Pani3H_1	-1379.390940600	0.408805	0.404676	0.331619	0.332814
	Pani3H_2	-1379.390863520	0.408721	0.404569	0.331487	0.332670
	Pani3H_3	-1379.390197940	0.408544	0.404300	0.330298	0.331897
$P(pfp)_3H^+$	Ppfp3H_1	-1333.654737100	0.280340	0.277072	0.213329	0.214879
PCy_3H^+	PCy3H_1	-1046.873868770	0.514459	0.511686	0.449827	0.449677
	PCy3H_2	-1046.874371110	0.514216	0.511400	0.449422	0.449188
	PCy3H_3	-1046.871214870	0.514320	0.511524	0.449409	0.449281
PBu_3H^+	PBu3H_1	-814.802200230	0.400030	0.396228	0.333545	0.333649
	PBu3H_2	-814.807971194	0.401250	0.398987	0.341536	0.340963
	PBu3H_3	-814.804382801	0.400679	0.397681	0.337504	0.337610
$POct_3H^+$	POct3H_1	-1286.110050360	0.757439	0.752149	0.666137	0.665492
	POct3H_2	-1286.108774210	0.757022	0.751529	0.664767	0.664274
	POct3H_3	-1286.108310240	0.75762	0.752227	0.664960	0.665007

6.3.4 Methylated phosphines

Table S54. Computed properties of the methyl cation and of methylated phosphines in dichloromethane. Level of theory: SMD(DCM)/MN15/def2-TZVPP. Energies are given in Hartree

Cations	Conformers	E_{tot}	ΔH	ΔG
CH_3^+	CH3_1	-39.5523638647	0.038606	0.015734
PMe_4^+	PMe4_1	-500.632957759	0.164440	0.123478
$PPhMe_3^+$	PPhMe3_1	-692.1997858730	0.221835	0.173656
$PPh_2Me_2^+$	PPh2Me2_1	-883.7671592140	0.278831	0.221683
PPh_3Me^+	PPh3Me_1	-1075.33471286	0.336875	0.271439
$P(ani)_3Me^+$	Pani3Me_1	-1418.70181668	0.442824	0.361672
	Pani3Me_2	-1418.70144164	0.442861	0.361453
	Pani3Me_3	-1418.70120633	0.442765	0.361267
$P(pfp)_3Me^+$	Ppfp3Me_1	-1372.96339501	0.314416	0.244398
PCy_3Me^+	PCy3Me_1	-1086.18250515	0.547363	0.475911
	PCy3Me_2	-1086.17989801	0.547649	0.477677
	PCy3Me_3	-1086.17711924	0.548366	0.479758
PBu_3Me^+	PBu3Me_1	-854.10872367	0.433731	0.362408
	PBu3Me_2	-854.10616798	0.434102	0.364755
	PBu3Me_3	-854.10843314	0.433723	0.363633

6.3.5 Zwitterions formed by the addition of PR_3 to ethyl acrylate (1)

Table S55. Computed properties of zwitterionic adducts from the reaction of PR_3 with **1** in dichloromethane. Entropic contributions have been corrected to the reference state. Δ*H** refers to thermal correction to enthalpy with the Head-Gordon correction and Δ*G** to the thermal correction to Gibbs Free Energy including the Grimme method. Level of theory: SMD(DCM)/MN15/def2-TZVPP. Energies are given in Hartree

Zwitterion	Conformers	E_{tot}	Δ*H*	Δ*H**	Δ*G*	Δ*G**
**1**_PMe_3	1_PMe3_1	-806.421429391	0.257208	0.251944	0.198262	0.198177
	1_PMe3_2	-806.421720571	0.257200	0.252108	0.198437	0.198637
	1_PMe3_3	-806.421325783	0.257209	0.251980	0.198539	0.198528
**1**_$PPhMe_2$	1_PPhMe2_1	-997.990027125	0.314354	0.308382	0.247367	0.248074
	1_PPhMe2_2	-997.989581932	0.314406	0.308308	0.247253	0.247768
	1_PPhMe2_3	-997.987637690	0.314367	0.308493	0.248168	0.248619
**1**_PPh_2Me	1_PPh2Me_1	-1189.55673742	0.371729	0.364701	0.296721	0.297566
	1_PPh2Me_2	-1189.55648592	0.371442	0.364582	0.296906	0.297692
	1_PPh2Me_3	-1189.55553959	0.371831	0.364861	0.297678	0.298189
**1**_PPh_3	1_PPh3_1	-1381.12364618	0.428880	0.421069	0.346153	0.347618
	1_PPh3_2	-1381.12381024	0.428759	0.420932	0.347417	0.348143
	1_PPh3_3	-1381.12281149	0.428620	0.420714	0.347039	0.347706
**1**_P(ani_3)	1_Pani3_1	-1724.48895740	0.534613	0.525534	0.436302	0.437361
	1_Pani3_2	-1724.48888097	0.534533	0.525363	0.436382	0.437451
	1_Pani3_3	-1724.48895257	0.534656	0.525616	0.436643	0.437807
**1**_$P(pfp)_3$	1_Ppfp3_1	-1678.75568150	0.406357	0.398114	0.319041	0.320009
	1_Ppfp3_2	-1678.75647043	0.406491	0.398279	0.320279	0.320864
	1_Ppfp3_3	-1678.75461434	0.406331	0.398482	0.320081	0.320966
**1**_PCy_3	1_PCy3_1	-1391.96719807	0.640272	0.632395	0.553451	0.553488
	1_PCy3_2	-1391.96590629	0.640441	0.632688	0.554257	0.554169
	1_PCy3_3	-1391.96417926	0.640144	0.632379	0.553937	0.553828
**1**_PBu_3	1_PBu3_1	-1159.89702071	0.526147	0.517545	0.437763	0.438823
	1_PBu3_2	-1159.89644720	0.526218	0.517655	0.438515	0.439071
	1_PBu3_3	-1159.89728687	0.525781	0.517750	0.440605	0.440506

6.3.6 Zwitterions formed by the addition of PR_3 to ethyl allenoate (2)

Table S56. Computed properties of zwitterionic adducts from the reaction of PR_3 with **2** in dichloromethane. Entropic contributions have been corrected to the reference state. Δ*H** refers to thermal correction to enthalpy with the Head-Gordon correction and Δ*G** to the thermal correction to Gibbs Free Energy including the Grimme method. Level of theory: SMD(DCM)/MN15/def2-TZVPP. Energies are given in Hartree

Zwitterion	Conformers	E_{tot}	Δ*H*	Δ*H**	Δ*G*	Δ*G**
**2**_PMe_3	2_PMe3_1	-844.484097410	0.264124	0.258788	0.204585	0.204236
	2_PMe3_2	-844.483906463	0.264318	0.261298	0.204852	0.204557
	2_PMe3_3	-844.483659612	0.264217	0.261198	0.204778	0.204651
**2**_$PPhMe_2$	2_PPhMe2_1	-1036.05323940	0.320894	0.314854	0.253764	0.254088
	2_PPhMe2_2	-1036.05291661	0.321004	0.314854	0.254027	0.254088
	2_PPhMe2_3	-1036.05272585	0.321037	0.315044	0.253989	0.254205
**2**_PPh_2Me	2_PPh2Me_1	-1227.62047827	0.377967	0.370903	0.302115	0.302969
	2_PPh2Me_2	-1227.62004551	0.378042	0.370770	0.301725	0.302364
	2_PPh2Me_3	-1227.62061987	0.378014	0.371038	0.303485	0.303720
**2**_PPh_3	2_PPh3_1	-1419.18866679	0.434760	0.426829	0.352832	0.353201
	2_PPh3_2	-1419.18783109	0.434887	0.426905	0.352175	0.352914
	2_PPh3_3	-1419.18775721	0.434961	0.426955	0.352349	0.352811
**2**_P(ani_3)	2_Pani3_1	-1762.55174181	0.540694	0.531163	0.439998	0.441188
	2_Pani3_2	-1762.55143839	0.540753	0.531270	0.439937	0.441234
	2_Pani3_3	-1762.55263702	0.540673	0.531437	0.441389	0.442354
**2**_$P(pfp)_3$	2_Ppfp3_1	-1716.82122520	0.412490	0.404224	0.324393	0.325290
	2_Ppfp3_2	-1716.82029678	0.412594	0.404199	0.323881	0.324757
	2_Ppfp3_3	-1716.82029682	0.412592	0.404198	0.323887	0.324759
**2**_PCy_3	2_PCy3_1	-1430.02403460	0.647028	0.639309	0.558502	0.558957
	2_PCy3_2	-1430.02419448	0.646941	0.639188	0.558993	0.559129
	2_PCy3_3	-1430.02392667	0.646937	0.639117	0.558869	0.558763
**2**_PBu_3	2_PBu3_1	-1197.96159391	0.532445	0.523744	0.443088	0.443617
	2_PBu3_2	-1197.96042697	0.532820	0.524236	0.444226	0.444754
	2_PBu3_3	-1197.96015752	0.532792	0.524175	0.444289	0.444831

6.3.7 Zwitterions formed by the addition of PR_3 to ethyl propiolate (3)

Table S57. Computed properties of zwitterionic adducts from the reaction of PR_3 with **3** in dichloromethane. Entropic contributions have been corrected to the reference state. Δ*H** refers to thermal correction to enthalpy with the Head-Gordon correction and Δ*G** to the thermal correction to Gibbs Free Energy including the Grimme method. Level of theory: SMD(DCM)/MN15/def2-TZVPP. Energies are given in Hartree

Zwitterion	Conformers	E_{tot}	Δ*H*	Δ*H**	Δ*G*	Δ*G**
**3**_PMe_3	3_PMe3_1	-805.17899611	0.234492	0.229510	0.177159	0.177106
	3_PMe3_2	-805.17900600	0.234589	0.229764	0.177979	0.177837
	3_PMe3_3	-805.17852743	0.234550	0.229558	0.177715	0.177469
**3**_$PPhMe_2$	3_PPhMe2_1	-996.74761931	0.291576	0.285777	0.225957	0.226496
	3_PPhMe2_2	-996.74854334	0.291842	0.286268	0.227977	0.227978
	3_PPhMe2_3	-996.74792494	0.291786	0.286218	0.227535	0.227773
**3**_PPh_2Me	3_PPh2Me_1	-1188.31723484	0.348748	0.342146	0.277272	0.277182
	3_PPh2Me_2	-1188.31698715	0.348727	0.341891	0.276212	0.276567
	3_PPh2Me_3	-1188.31591222	0.348851	0.341976	0.275419	0.276139
**3**_PPh_3	3_PPh3_1	-1379.88584865	0.405847	0.398077	0.325896	0.326231
	3_PPh3_2	-1379.88482065	0.405783	0.397896	0.325207	0.325834
	3_PPh3_3	-1379.88482080	0.405782	0.397895	0.325215	0.325838
**3**_P(ani_3)	3_Pani3_1	-1723.24975688	0.511504	0.502475	0.413817	0.415050
	3_Pani3_2	-1723.25027535	0.511715	0.502352	0.413145	0.414560
	3_Pani3_3	-1723.24956702	0.511511	0.502321	0.413092	0.414620
**3**_$P(pfp)_3$	3_Ppfp3_1	-1677.51681629	0.382975	0.374704	0.294657	0.296482
	3_Ppfp3_2	-1677.51806945	0.383320	0.375110	0.296869	0.297752
	3_Ppfp3_3	-1677.51604561	0.383245	0.374964	0.295965	0.297241
**3**_PCy_3	3_PCy3_1	-1390.72795325	0.616924	0.608939	0.529846	0.530236
	3_PCy3_2	-1390.72833730	0.617109	0.609158	0.530917	0.530843
	3_PCy3_3	-1390.72761302	0.617301	0.609438	0.531680	0.531502
**3**_PBu_3	3_PBu3_1	-1158.65802050	0.503091	0.494793	0.417834	0.418188
	3_PBu3_2	-1158.65594975	0.503232	0.494877	0.416085	0.417349
	3_PBu3_3	-1158.65890102	0.503460	0.495474	0.420110	0.419841

6.3.8 Transition state (TS) properties for additions of PR_3 to acrylate 1

Table S58. Computed TS properties for additions of PR_3 to **1**. Entropic contributions have been corrected to the reference state. Δ*H** refers to thermal correction to enthalpy with the Head-Gordon correction and Δ*G** to the thermal correction to Gibbs Free Energy including the Grimme method. Level of theory: SMD(DCM)/MN15/def2-TZVPP. Energies are given in Hartree

Zwitterion	Conformers	E_{tot}	Δ*H*	Δ*H**	Δ*G*	Δ*G**
**1**_TS _PMe_3	1_TS_PMe3_1	-806.398954608	0.256299	0.254080	0.197519	0.197474
	1_TS_PMe3_2	-806.399273016	0.256325	0.254228	0.198551	0.198331
	1_TS_PMe3_3	-806.398614487	0.256373	0.254161	0.197771	0.197865
**1**_TS _$PPhMe_2$	1_TS_PPhMe2_1	-997.971291242	0.313627	0.310608	0.246380	0.247145
	1_TS_PPhMe2_2	-997.971646071	0.313749	0.310873	0.247967	0.248321
	1_TS_PPhMe2_3	-997.970967416	0.313852	0.310863	0.247647	0.248154
**1**_TS _PPh_2Me	1_TS_PPh2Me_1	-1189.54149527	0.370714	0.366632	0.294936	0.296335
	1_TS_PPh2Me_2	-1189.54244427	0.370634	0.366652	0.296243	0.297111
	1_TS_PPh2Me_3	-1189.54278607	0.370527	0.366730	0.297213	0.297759
**1**_TS _PPh_3	1_TS_PPh3_1	-1381.11447355	0.427499	0.422502	0.345663	0.346585
	1_TS_PPh3_2	-1381.11357390	0.427683	0.422470	0.344987	0.346294
	1_TS_PPh3_3	-1381.11207388	0.427819	0.422831	0.345640	0.346783
**1**_TS _P(ani_3)	1_TS_Pani3_1	-1724.47753236	0.533328	0.526967	0.433814	0.435516
	1_TS_Pani3_2	-1724.47752156	0.533324	0.527139	0.434804	0.436115
	1_TS_Pani3_3	-1724.47763829	0.533379	0.527268	0.435358	0.43649
**1**_TS _$P(pfp)_3$	1_TS_Ppfp3_1	-1678.74758955	0.405228	0.3968	0.317352	0.318732
	1_TS_Ppfp3_2	-1678.74732263	0.405142	0.396774	0.317576	0.318748
	1_TS_Ppfp3_3	-1678.74627356	0.405254	0.39674	0.316856	0.318418
**1**_TS _PCy_3	1_TS_PCy3_1	-1391.94658338	0.638499	0.633388	0.550105	0.550697
	1_TS_PCy3_2	-1391.94665249	0.638599	0.633509	0.551000	0.551412
	1_TS_PCy3_3	-1391.94677564	0.638517	0.633563	0.551713	0.551838
**1**_TS _PBu_3	1_TS_PBu3_1	-1159.87597217	0.524157	0.518243	0.434511	0.435687
	1_TS_PBu3_2	-1159.87553872	0.524393	0.518547	0.435530	0.436641
	1_TS_PBu3_3	-1159.87489326	0.524299	0.518457	0.435010	0.435893

6.3.9 Transition state (TS) properties for additions of PR_3 to allenoate 2

Table S59. Computed TS properties for additions of PR_3 to **2**. Entropic contributions have been corrected to the reference state. Δ*H** refers to thermal correction to enthalpy with the Head-Gordon correction and Δ*G** to the thermal correction to Gibbs Free Energy including the Grimme method. Level of theory: SMD(DCM)/MN15/def2-TZVPP. Energies are given in Hartree

Zwitterion	Conformers	E_{tot}	Δ*H*	Δ*H**	Δ*G*	Δ*G**
**2**_TS _PMe_3	2_TS_PMe3_1	-844.434359958	0.260185	0.258348	0.198671	0.198802
	2_TS_PMe3_2	-844.433924973	0.261157	0.258334	0.198678	0.198932
	2_TS_PMe3_3	-844.434677659	0.261092	0.258420	0.199582	0.199503
**2**_TS _$PPhMe_2$	2_TS_PPhMe2_1	-1036.00737233	0.318324	0.314759	0.246835	0.248002
	2_TS_PPhMe2_2	-1036.03314643	0.320003	0.316317	0.249537	0.249507
	2_TS_PPhMe2_3	-1036.00788462	0.318751	0.315546	0.250057	0.250466
**2**_TS _PPh_2Me	2_TS_PPh2Me_1	-1227.58123342	0.375584	0.371722	0.368703	0.300774
	2_TS_PPh2Me_2	-1227.58032109	0.375622	0.371468	0.368449	0.300387
	2_TS_PPh2Me_3	-1227.57804168	0.375867	0.371657	0.368638	0.300120
**2**_TS _PPh_3	2_TS_PPh3_1	-1419.15392971	0.432513	0.427501	0.349004	0.349786
	2_TS_PPh3_2	-1419.15196320	0.432645	0.427195	0.347404	0.348739
	2_TS_PPh3_3	-1419.15248671	0.432682	0.427513	0.349940	0.350254
**2**_TS _P(ani_3)	2_TS_Pani3_1	-1762.51547431	0.538256	0.531852	0.437081	0.438541
	2_TS_Pani3_2	-1762.51597003	0.538393	0.532084	0.437765	0.439055
	2_TS_Pani3_3	-1762.51557423	0.538387	0.531954	0.437426	0.438781
**2**_TS _$P(pfp)_3$	2_TS_Ppfp3_1	-1716.78634108	0.410099	0.404686	0.319585	0.321207
	2_TS_Ppfp3_2	-1716.78484260	0.410114	0.404229	0.318162	0.320151
	2_TS_Ppfp3_3	-1716.78596861	0.410171	0.404572	0.319825	0.321134
**2**_TS _PCy_3	2_TS_PCy3_1	-1429.98107662	0.643563	0.635430	0.554017	0.554044
	2_TS_PCy3_2	-1429.98124553	0.643652	0.635471	0.553762	0.554177
	2_TS_PCy3_3	-1429.98435508	0.643512	0.635131	0.552481	0.553169
**2**_TS _PBu_3	2_TS_PBu3_1	-1197.91652972	0.529725	0.524392	0.442208	0.442517
	2_TS_PBu3_2	-1197.91408586	0.529622	0.524933	0.444916	0.444308
	2_TS_PBu3_3	-1197.91323843	0.529701	0.524764	0.444459	0.443782

6.3.10 Transition state (TS) properties for additions of PR_3 to propiolate 3

Table S60. Computed TS properties for additions of PR_3 to **3**. Entropic contributions have been corrected to the reference state. Δ*H** refers to thermal correction to enthalpy with the Head-Gordon correction and Δ*G** to the thermal correction to Gibbs Free Energy including the Grimme method. Level of theory: SMD(DCM)/MN15/def2-TZVPP. Energies are given in Hartree

Zwitterion	Conformers	E_{tot}	Δ*H*	Δ*H**	Δ*G*	Δ*G**
**3**_TS _PMe_3	3_TS_PMe3_1	-805.14255337	0.232306	0.230000	0.173475	0.173423
	3_TS_PMe3_2	-805.14310645	0.232317	0.230181	0.174190	0.174069
	3_TS_PMe3_3	-805.14219484	0.232402	0.230147	0.173870	0.173882
**3**_TS _$PPhMe_2$	3_TS_PPhMe2_1	-996.71475825	0.289782	0.286616	0.222393	0.223173
	3_TS_PPhMe2_2	-996.71435899	0.289655	0.286517	0.222489	0.223283
	3_TS_PPhMe2_3	-996.71530297	0.289829	0.286847	0.223918	0.224291
**3**_TS _PPh_2Me	3_TS_PPh2Me_1	-1188.28829716	0.346681	0.342954	0.274056	0.274376
	3_TS_PPh2Me_2	-1188.28578521	0.346886	0.342872	0.272033	0.273074
	3_TS_PPh2Me_3	-1188.28747819	0.346686	0.342830	0.273793	0.274202
**3**_TS _PPh_3	3_TS_PPh3_1	-1379.85872155	0.403588	0.398484	0.320689	0.322067
	3_TS_PPh3_2	-1379.85795367	0.403678	0.398455	0.321017	0.322246
	3_TS_PPh3_3	-1379.85725242	0.403516	0.398312	0.320698	0.321999
**3**_TS _P(ani_3)	3_TS_Pani3_1	-1723.22035187	0.509123	0.499531	0.406935	0.409711
	3_TS_Pani3_2	-1723.22046259	0.509233	0.499655	0.408725	0.410715
	3_TS_Pani3_3	-1723.22033792	0.509204	0.499597	0.409196	0.410876
**3**_TS _$P(pfp)_3$	3_TS_Ppfp3_1	-1677.49058211	0.380993	0.375400	0.291234	0.293297
	3_TS_Ppfp3_2	-1677.49060670	0.381193	0.375616	0.292189	0.293944
	3_TS_Ppfp3_3	-1677.49025285	0.381235	0.375726	0.293029	0.294459
**3**_TS _PCy_3	3_TS_PCy3_1	-1390.69547386	0.614346	0.609409	0.528224	0.527935
	3_TS_PCy3_2	-1390.69411010	0.614246	0.609110	0.525933	0.526534
	3_TS_PCy3_3	-1390.69353514	0.614439	0.609353	0.527351	0.527407
**3**_TS _PBu_3	3_TS_PBu3_1	-1158.62260571	0.500358	0.495140	0.413535	0.414489
	3_TS_PBu3_2	-1158.62260587	0.500360	0.495142	0.413562	0.414503
	3_TS_PBu3_3	-1158.62232994	0.500390	0.495077	0.413776	0.414582

6.3.11 NBO analysis

Table S61. NBO charges of the transition states of the additions of PPh_3 to **1**, **2**, or **3**. Level of theory: SMD(DCM)/MN15/def2-TZVPP

**1**-PPh_3						**2**-PPh_3						**3**-PPh_3					
**1**			PPh_3			**2**			PPh_3			**3**			PPh_3		
Atom	Number	Charge	Atom	Number	Charge	Atom	Number	Charge	Atom	Number	Charge	Atom	Number	Charge	Atom	Number	Charge
C	35	-0.40169	P	1	1.25963	C	1	-0.46429	P	4	1.17676	C	35	-0.25306	P	1	1.14952
C	36	-0.52669	C	2	-0.38779	C	2	0.08114	C	10	-0.37717	C	36	-0.23967	C	2	-0.37616
C	37	0.71758	C	3	-0.38041	C	3	-0.56226	C	11	-0.38394	C	37	0.69173	C	3	-0.36951
O	38	-0.72086	C	4	-0.38892	C	5	0.74225	C	12	-0.37497	O	38	-0.65176	C	4	-0.37317
O	39	-0.52071	C	5	-0.19926	O	6	-0.67630	C	13	-0.20494	O	39	-0.49811	C	5	-0.19764
C	40	-0.08013	C	6	-0.21254	O	7	-0.52658	C	14	-0.21569	C	40	-0.08697	C	6	-0.21690
C	41	-0.62421	C	7	-0.20086	C	8	-0.08094	C	15	-0.20248	C	41	-0.62617	C	7	-0.20261
H	42	0.22543	C	8	-0.21236	C	9	-0.62741	C	16	-0.21349	H	42	0.23501	C	8	-0.21593
H	43	0.22188	C	9	-0.19633	H	28	0.22240	C	17	-0.20095	H	43	0.19316	C	9	-0.19269
H	44	0.18563	C	10	-0.18966	H	29	0.23269	C	18	-0.19855	H	44	0.19075	C	10	-0.20357
H	45	0.18009	C	11	-0.22199	H	30	0.18846	C	19	-0.21330	H	45	0.22228	C	11	-0.21240
H	46	0.21824	C	12	-0.2062	H	31	0.18738	C	20	-0.20102	H	46	0.21183	C	12	-0.20560
H	47	0.20877	C	13	-0.22025	H	32	0.22236	C	21	-0.21345	H	47	0.21477	C	13	-0.21140
H	48	0.21105	C	14	-0.19423	H	33	0.21239	C	22	-0.19932				C	14	-0.20277
H	49	0.21416	C	15	-0.19789	H	34	0.21109	C	23	-0.20560				C	15	-0.20551
			C	16	-0.21468	H	50	0.24131	C	24	-0.21801				C	16	-0.21481
			C	17	-0.2031				C	25	-0.20929				C	17	-0.20631
			C	18	-0.21298				C	26	-0.21221				C	18	-0.21173
			C	19	-0.21056				C	27	-0.20591				C	19	-0.20810
			H	20	0.23016				H	35	0.23928				H	20	0.23343
			H	21	0.23185				H	36	0.23123				H	21	0.23093
			H	22	0.23021				H	37	0.22945				H	22	0.22982
			H	23	0.23129				H	38	0.23025				H	23	0.23150

	H	24	0.23403		H	39	0.23145		H	24	0.23230
	H	25	0.23170		H	40	0.23013		H	25	0.22895
	H	26	0.22955		H	41	0.23092		H	26	0.23106
	H	27	0.22863		H	42	0.22962		H	27	0.22951
	H	28	0.23070		H	43	0.23067		H	28	0.23034
	H	29	0.23110		H	44	0.23227		H	29	0.23237
	H	30	0.23113		H	45	0.23111		H	30	0.23073
	H	31	0.22990		H	46	0.22967		H	31	0.22915
	H	32	0.22889		H	47	0.22912		H	32	0.22846
	H	33	0.23095		H	48	0.23179		H	33	0.23083
	H	34	0.25175		H	49	0.23289		H	34	0.24411

6.3.12 Thermodynamics of allenoate and butynoate isomerisations

Table S62. Gas phase calculation performed at the DLPNO-CCSD(T)/def2-TZVP//MN15/def2-TZVPP level of theory. Δ*H** refers to enthalpy correction according to Head-Gordon. Δ*G** refers to entropy correction according to Grimme and have been corrected to a reference state of 1 mol L^{-1} at 298.15 K. $\Delta_f H°$ values are according to active thermochemical tables.[S36]

Molecule	E_{tot}	DLPNO-CCSD(T)	Δ*H*	Δ*H**	Δ*G*	Δ*G**	MN15/def2-TZVPP rel. Δ*H*	MN15/def2-TZVPP rel. Δ*H**	MN15/def2-TZVPP rel. Δ*G*	MN15/def2-TZVPP rel. Δ*G**	DLPNO-CCSD(T) rel. Δ*H*	DLPNO-CCSD(T) rel. Δ*H**	DLPNO-CCSD(T) rel. Δ*G*	DLPNO-CCSD(T) rel. Δ*G**	$\Delta_f H°$ (298 K)[16]
	(energies in Hartree)						(energies in kJ mol^{-1})								
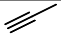	-116.544998510	-116.426243634	0.060937	0.060927	0.031981	0.034987	0	0	0	0	0	0	0	0	0
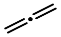	-116.545912301	-116.424131416	0.060108	0.060099	0.031289	0.034297	-4.6	-4.6	-4.2	-4.2	3.4	3.4	3.7	3.7	4.4
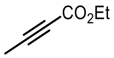	-383.555056757	-383.203408203	0.139109	0.137588	0.091207	0.095025	0	0	0	0	0	0	0	0	-
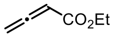	-383.559837777	-383.205891558	0.138106	0.136978	0.093903	0.096611	-15.2	-14.2	-5.5	-8.4	-9.2	-8.1	0.6	-2.4	-

Table S63. Solvation was considered at the DLPNO-CCSD(T)/def2-TZVP//MN15-SMD(DCM)/def2-TZVPP level of theory. Δ*H** refers to enthalpy correction according to Head-Gordon. Δ*G** refers to entropy correction according to Grimme and have been corrected to a reference state of 1 mol/l at 298.15 K. Solv. refers to the solvation energy.

Molecule	E_{tot}	DLPNO-CCSD(T)	Δ*H*	Δ*H**	Δ*G*	Δ*G**	Solv.	MN15/def2-TZVPP rel. Δ*H*	MN15/def2-TZVPP rel. Δ*H**	MN15/def2-TZVPP rel. Δ*G*	MN15/def2-TZVPP rel. Δ*G**	DLPNO-CCSD(T) rel. Δ*H*	DLPNO-CCSD(T) rel. Δ*H**	DLPNO-CCSD(T) rel. Δ*G*	DLPNO-CCSD(T) rel. Δ*G**
	(energies in Hartree)							(energies in kJ mol^{-1})							
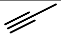	-116.551073181	-116.426334846	0.060742	0.060732	0.031801	0.034807	-0.006095264	0	0	0	0	0	0	0	0
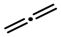	-116.549972995	-116.424191030	0.059911	0.059902	0.031082	0.03409	-0.004067023	-15.2	-14.2	-5.5	-8.4	-9.2	-8.1	0.6	-2.4
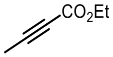	-383.567809949	-383.203408203	0.138776	0.137266	0.092801	0.095655	-0.012904003	0	0	0	0	0	0	0	0
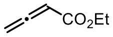	-383.571065782	-383.205824149	0.137811	0.136721	0.093766	0.096446	-0.011354957	-11.1	-10.0	-6.0	-6.5	-4.8	-3.7	0.3	-0.2

6.3.13 Theoretical pK_{aH} value prediction

Theoretical pK_{aH} values were determined according to Equation (S1) based on ΔG_{sol} values as defined in reaction Equation (S2).

$$\mathrm{p}K_{\mathrm{aH}}(\mathrm{PR_3}) = \mathrm{p}K_{\mathrm{aH}}(\mathrm{PPh_3}, 2.73) + \frac{\Delta G_{\mathrm{sol}}}{2.303\mathrm{RT}} \qquad \text{(S1)}$$

$$^{\oplus}\mathrm{PR_3H} + \mathrm{PPh_3} \xrightarrow{\Delta G_{\mathrm{sol}}} \mathrm{PR_3} + {}^{\oplus}\mathrm{PPh_3H} \qquad \text{(S2)}$$

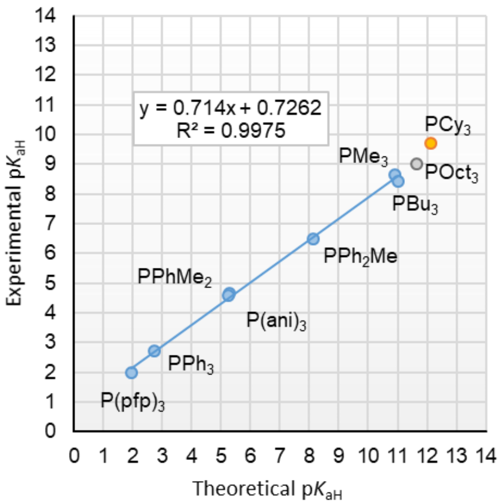

Fig. S29. Correlation between experimental pK_{aH} and quantum-chemically calculated pK_{aH} (level of theory: SMD(H_2O)/MN15/def2-TZVPP).

Table S64. Quantum-chemically calculated and experimental pK_{aH} values in H_2O. Level of theory: SMD(H_2O)/MN15/def2-TZVPP

Phosphine	Experimental pK_{aH}[a]	Theoretical pK_{aH}	Predicted pK_{aH}
PMe_3	8.65	10.92	-
$PPhMe_2$	6.49	8.15	-
PPh_2Me	4.65	5.31	-
PPh_3	2.73	2.73	-
$P(ani)_3$	4.57	5.27	-
$P(pfp)_3$	1.97	1.98	-
PCy_3	9.70	(12.14)[b]	-
PBu_3	8.43	11.03	-
$POCt_3$	-	11.63	9.03[c]

[a] Experimental pK_{aH} of tertiary phosphines from ref. S1. [b] Data for PCy_3 was excluded when calculating the correlation equation in Fig. S29. [c] Extrapolated from the correlation equation in Fig. S29.

6.3.14 Activation strain analysis for reactions of PPh_3 with 1, 2, or 3

Table S65. Energy strain analysis of the PPh_3 addition to **1**. Level of theory: SMD(DCM)/MN15/def2-TZVPP. RC refers to reactant complex and PC to product complex. Energies are given in Hartree

Step	P-C	E_{tot}	$SP_{\mathbf{1}}$	SP_{PPh3}	$SP_{\mathbf{1}+PPh3}$	E_{tot}- $SP_{\mathbf{1}+PPh3}$
OPT RC	4.02059	-1381.13800117	-345.533145772	-1035.59509257	-1381.128238342	-0.00976283
OPT **1**	-	-345.533372803	-	-	-	-
OPT PPh_3	-	-1035.59515116	-	-	-	-
1	3.47720	-1381.13379866	-345.533251402	-1035.59339244	-1381.126643842	-0.00715482
2	3.46990	-1381.13371180	-345.533248024	-1035.59334385	-1381.126591874	-0.00711993
3	3.46208	-1381.13362413	-345.533245127	-1035.59329511	-1381.126540237	-0.00708389
4	3.45447	-1381.13353439	-345.533242139	-1035.59324513	-1381.126487269	-0.00704712
5	3.44692	-1381.13344335	-345.533238457	-1035.59319440	-1381.126432857	-0.00701049
6	3.43971	-1381.13335116	-345.533235656	-1035.59314248	-1381.126378136	-0.00697302
7	3.43201	-1381.13325694	-345.533232337	-1035.59309053	-1381.126322867	-0.00693407
8	3.42399	-1381.13316142	-345.533229353	-1035.59303815	-1381.126267503	-0.00689392
9	3.41537	-1381.13306478	-345.533225470	-1035.59298607	-1381.126211540	-0.00685324
10	3.40705	-1381.13296602	-345.533221158	-1035.59293299	-1381.126154148	-0.00681187
11	3.39881	-1381.13286603	-345.533216855	-1035.59287959	-1381.126096445	-0.00676959
12	3.39102	-1381.13276485	-345.533213787	-1035.59282575	-1381.126039537	-0.00672531
13	3.38272	-1381.13266146	-345.533209903	-1035.59277339	-1381.125983293	-0.00667817
14	3.37406	-1381.13255677	-345.533205778	-1035.59272209	-1381.125927868	-0.00662890
15	3.36477	-1381.13245086	-345.533200241	-1035.59267234	-1381.125872581	-0.00657828
16	3.35583	-1381.13234261	-345.533194656	-1035.59262231	-1381.125816966	-0.00652564
17	3.34697	-1381.13223299	-345.533189141	-1035.59257282	-1381.125761961	-0.00647103
18	3.33857	-1381.13212188	-345.533185130	-1035.59252309	-1381.125708220	-0.00641366
19	3.32961	-1381.13200829	-345.533180062	-1035.59247461	-1381.125654672	-0.00635362
20	3.32027	-1381.13189321	-345.533174154	-1035.59242665	-1381.125600804	-0.00629241
21	3.31025	-1381.13177651	-345.533166580	-1035.59237983	-1381.125546410	-0.00623010
22	3.30056	-1381.13165716	-345.533158805	-1035.59233255	-1381.125491355	-0.00616580
23	3.29090	-1381.13153616	-345.533151741	-1035.59228558	-1381.125437321	-0.00609884
24	3.28164	-1381.13141311	-345.533146267	-1035.59223796	-1381.125384227	-0.00602888
25	3.27173	-1381.13128711	-345.533139920	-1035.59219124	-1381.125331160	-0.00595595
26	3.25035	-1381.13102831	-345.533123012	-1035.59210011	-1381.125223122	-0.00580519
27	3.23963	-1381.13089431	-345.533113571	-1035.59205623	-1381.125169801	-0.00572451
28	3.22907	-1381.13075655	-345.533106837	-1035.59201533	-1381.125122167	-0.00563438
29	3.21710	-1381.13061664	-345.533093116	-1035.59197697	-1381.125070086	-0.00554655
30	3.20549	-1381.13047189	-345.533081516	-1035.59193724	-1381.125018756	-0.00545313
31	3.19378	-1381.13032432	-345.533069915	-1035.59189573	-1381.124965645	-0.00535868
32	3.18248	-1381.13017283	-345.533063097	-1035.59185137	-1381.124914467	-0.00525836
33	3.17040	-1381.13001610	-345.533051035	-1035.59180806	-1381.124859095	-0.00515701
34	3.15783	-1381.12985599	-345.533037368	-1035.59176855	-1381.124805918	-0.00505007
35	3.14458	-1381.12969176	-345.533016645	-1035.59173434	-1381.124750985	-0.00494078
36	3.13164	-1381.12952208	-345.533000246	-1035.59170240	-1381.124702646	-0.00481943
37	3.11861	-1381.12934857	-345.532982473	-1035.59167323	-1381.124655703	-0.00469287
38	3.10578	-1381.12917014	-345.532969439	-1035.59164619	-1381.124615629	-0.00455451
39	3.09241	-1381.12898604	-345.532947630	-1035.59161922	-1381.124566850	-0.00441919

40	3.07871	-1381.12879727	-345.532926328	-1035.59158940	-1381.124515728	-0.00428154
41	3.06464	-1381.12860253	-345.532897107	-1035.59155355	-1381.124450657	-0.00415187
42	3.05065	-1381.12840098	-345.532876796	-1035.59151568	-1381.124392476	-0.00400850
43	3.03652	-1381.12819413	-345.532850702	-1035.59147857	-1381.124329272	-0.00386486
44	3.02221	-1381.12797942	-345.532827929	-1035.59144710	-1381.124275029	-0.00370439
45	3.00768	-1381.12775738	-345.532789762	-1035.59141585	-1381.124205612	-0.00355177
46	2.99286	-1381.12752985	-345.532757618	-1035.59138950	-1381.124147118	-0.00338273
47	2.97813	-1381.12729216	-345.532717165	-1035.59136404	-1381.124081205	-0.00321095
48	2.96317	-1381.12704816	-345.532690220	-1035.59134955	-1381.124039770	-0.00300839
49	2.94812	-1381.12679709	-345.532639531	-1035.59133481	-1381.123974341	-0.00282275
50	2.93292	-1381.12653471	-345.532603331	-1035.59131849	-1381.123921821	-0.00261289
51	2.91758	-1381.12626639	-345.532555836	-1035.59129677	-1381.123852606	-0.00241378
52	2.90167	-1381.12598560	-345.532492593	-1035.59127488	-1381.123767473	-0.00221813
53	2.88605	-1381.12570035	-345.532418852	-1035.59124297	-1381.123661822	-0.00203853
54	2.87018	-1381.12540505	-345.532358206	-1035.59121225	-1381.123570456	-0.00183459
55	2.85416	-1381.12509759	-345.532272254	-1035.59117711	-1381.123449364	-0.00164823
56	2.83784	-1381.12478446	-345.532174871	-1035.59114133	-1381.123316201	-0.00146826
57	2.82244	-1381.12446226	-345.532123452	-1035.59109505	-1381.123218502	-0.00124376
58	2.80611	-1381.12412908	-345.532037387	-1035.59105929	-1381.123096677	-0.00103240
59	2.78974	-1381.12378906	-345.531922487	-1035.59102018	-1381.122942667	-0.00084639
60	2.77329	-1381.12343856	-345.531819041	-1035.59098247	-1381.122801511	-0.00063705
61	2.75669	-1381.12307918	-345.531694283	-1035.59094311	-1381.122637393	-0.00044179
62	2.73997	-1381.12271145	-345.531563703	-1035.59090330	-1381.122467003	-0.00024445
63	2.72313	-1381.12233573	-345.531417304	-1035.59086206	-1381.122279364	-0.00005637
64	2.70617	-1381.12195251	-345.531257753	-1035.59081858	-1381.122076333	0.00012382
65	2.68908	-1381.12156236	-345.531081774	-1035.59077346	-1381.121855234	0.00029287
66	2.67185	-1381.12116595	-345.530887910	-1035.59072754	-1381.121615450	0.00044950
67	2.65447	-1381.12076402	-345.530673291	-1035.59068056	-1381.121353851	0.00058983
68	2.63694	-1381.12035740	-345.530436779	-1035.59063264	-1381.121069419	0.00071202
69	2.61925	-1381.11994702	-345.530172800	-1035.59058405	-1381.120756850	0.00080983
70	2.60138	-1381.11953398	-345.529878428	-1035.59053507	-1381.120413498	0.00087952
71	2.58333	-1381.11911954	-345.529551712	-1035.59048550	-1381.120037212	0.00091767
72	2.56508	-1381.11870516	-345.529186303	-1035.59043669	-1381.119622993	0.00091783
73	2.54662	-1381.11829257	-345.528777450	-1035.59038819	-1381.119165640	0.00087307
74	2.52794	-1381.11788377	-345.528320864	-1035.59034170	-1381.118662564	0.00077879
75	2.50904	-1381.11748111	-345.527808726	-1035.59029721	-1381.118105936	0.00062483
76	2.48991	-1381.11708734	-345.527236617	-1035.59025539	-1381.117492007	0.00040467
77	2.47055	-1381.11670564	-345.526596839	-1035.59021723	-1381.116814069	0.00010843
78	2.45097	-1381.11633960	-345.525884262	-1035.59018250	-1381.116066762	-0.00027284
79	2.43117	-1381.11599321	-345.525091376	-1035.59015246	-1381.115243836	-0.00074937
80	2.41116	-1381.11567084	-345.524210050	-1035.59012591	-1381.114335960	-0.00133488
81	2.39095	-1381.11537711	-345.523236599	-1035.59010432	-1381.113340919	-0.00203619
82	2.37057	-1381.11511682	-345.522166494	-1035.59008587	-1381.112252364	-0.00286446
83	2.35002	-1381.11489473	-345.520993212	-1035.59007079	-1381.111064002	-0.00383073
84	2.32934	-1381.11471539	-345.519717331	-1035.59005959	-1381.109776921	-0.00493847
85	2.30854	-1381.11458303	-345.518335996	-1035.59005126	-1381.108387256	-0.00619577
86	2.28763	-1381.11450138	-345.516852966	-1035.59004570	-1381.106898666	-0.00760271
87	2.26665	-1381.11447355	-345.515263540	-1035.59004135	-1381.105304890	-0.00916866

88	2.24560	-1381.11450185	-345.513574957	-1035.59003882	-1381.103613777	-0.01088807
89	2.22453	-1381.11458747	-345.511798809	-1035.59003591	-1381.101834719	-0.01275275
90	2.20348	-1381.11473038	-345.509936926	-1035.59003345	-1381.099970376	-0.01476000
91	2.18245	-1381.11492931	-345.508005535	-1035.59003014	-1381.098035675	-0.01689364
92	2.16149	-1381.11518182	-345.506011646	-1035.59002433	-1381.096035976	-0.01914584
93	2.14062	-1381.11548439	-345.503969505	-1035.59001667	-1381.093986175	-0.02149821
94	2.11988	-1381.11583242	-345.501894833	-1035.59000566	-1381.091900493	-0.02393193
95	2.09930	-1381.11622005	-345.499800396	-1035.58999105	-1381.089791446	-0.02642860
96	2.07895	-1381.11664009	-345.497705423	-1035.58997064	-1381.087676063	-0.02896403
97	2.05888	-1381.11708400	-345.495623159	-1035.58994386	-1381.085567019	-0.03151698
98	2.03920	-1381.11754213	-345.493584453	-1035.58990776	-1381.083492213	-0.03404992
99	2.01999	-1381.11800415	-345.491604649	-1035.58985994	-1381.081464589	-0.03653956
100	2.00141	-1381.11845979	-345.489719056	-1035.58979909	-1381.079518146	-0.03894164
101	1.98361	-1381.11889937	-345.487948887	-1035.58972042	-1381.077669307	-0.04123006
102	1.96677	-1381.11931430	-345.486330590	-1035.58962349	-1381.075954080	-0.04336022
103	1.95113	-1381.11969772	-345.484890813	-1035.58950409	-1381.074394903	-0.04530282
104	1.93690	-1381.12004499	-345.483651152	-1035.58936230	-1381.073013452	-0.04703154
105	1.92429	-1381.12035394	-345.482616901	-1035.58919944	-1381.071816341	-0.04853760
106	1.91343	-1381.12062480	-345.481794417	-1035.58901977	-1381.070814187	-0.04981061
107	1.90434	-1381.12085990	-345.481153197	-1035.58883377	-1381.069986967	-0.05087293
108	1.89695	-1381.12106329	-345.480703939	-1035.58865361	-1381.069357549	-0.05170574
109	1.89096	-1381.12123998	-345.480301904	-1035.58849303	-1381.068794934	-0.05244505
110	1.88661	-1381.12139487	-345.480237332	-1035.58836122	-1381.068598552	-0.05279632
111	1.88290	-1381.12153245	-345.479795021	-1035.58825836	-1381.068053381	-0.05347907
112	1.87960	-1381.12165696	-345.479784912	-1035.58820481	-1381.067989722	-0.05366724
113	1.87831	-1381.12177052	-345.479617211	-1035.58814918	-1381.067766391	-0.05400413
114	1.87469	-1381.12187589	-345.479272510	-1035.58815290	-1381.067425410	-0.05445048
115	1.87434	-1381.12197282	-345.479284541	-1035.58812703	-1381.067411571	-0.05456125
116	1.87292	-1381.12206156	-345.479105692	-1035.58813790	-1381.067243592	-0.05481797
117	1.87161	-1381.12214224	-345.478938422	-1035.58815161	-1381.067090032	-0.05505221
118	1.87037	-1381.12221530	-345.478751493	-1035.58817330	-1381.066924793	-0.05529051
119	1.86942	-1381.12228106	-345.478598158	-1035.58819189	-1381.066790048	-0.05549101
120	1.86819	-1381.12233988	-345.478375723	-1035.58822807	-1381.066603793	-0.05573609
121	1.86688	-1381.12239275	-345.478169139	-1035.58826038	-1381.066429519	-0.05596323
122	1.86610	-1381.12243944	-345.477983738	-1035.58829659	-1381.066280328	-0.05615911
123	1.86613	-1381.12248132	-345.477904658	-1035.58831469	-1381.066219348	-0.05626197
124	1.86493	-1381.12251856	-345.477652639	-1035.58836397	-1381.066016609	-0.05650195
125	1.86373	-1381.12255169	-345.477438909	-1035.58839453	-1381.065833439	-0.05671825
126	1.86344	-1381.12258153	-345.477289119	-1035.58842797	-1381.065717089	-0.05686444
127	1.86274	-1381.12260849	-345.477113184	-1035.58845806	-1381.065571244	-0.05703725
128	1.86226	-1381.12263283	-345.476954460	-1035.58848687	-1381.065441330	-0.05719150
129	1.86265	-1381.12265509	-345.476907461	-1035.58849650	-1381.065403961	-0.05725113
130	1.86158	-1381.12267501	-345.476697614	-1035.58852505	-1381.065222664	-0.05745235
OPT PC	1.85965	-1381.12281148	-345.476580949	-1035.58856735	-1381.065148299	-0.05766318

Table S66. Energy strain analysis of the PPh_3 addition to **2**. Level of theory: SMD(DCM)/MN15/def2-TZVPP. RC refers to reactant complex and PC to product complex. Energies are given in Hartree

Step	P-C	E_{tot}	$SP_{\mathbf{2}}$	SP_{PPh3}	$SP_{\mathbf{2}+PPh3}$	E_{tot}- $SP_{\mathbf{2}+PPh3}$
OPT RC	3.82985	-1419.17820485	-383.571065763	-1035.59514749	-1419.166213253	-0.011991597
OPT **2**	-	-383.571065763	-	-	-	-
OPT PPh_3	-	-1035.59514749	-	-	-	-
1	3.32166	-1419.17278615	-383.570745979	-1035.59351554	-1419.164261519	-0.008524630
2	3.31181	-1419.17264884	-383.570737171	-1035.59349199	-1419.164229161	-0.008524631
3	3.30168	-1419.17250840	-383.570727372	-1035.59346930	-1419.164196672	-0.008419679
4	3.29149	-1419.17236387	-383.570717476	-1035.59344787	-1419.164165346	-0.008311728
5	3.28134	-1419.17221512	-383.570706816	-1035.59342790	-1419.164134716	-0.008198524
6	3.27020	-1419.17206279	-383.570693819	-1035.59340898	-1419.164102799	-0.008080404
7	3.25935	-1419.17190497	-383.570681348	-1035.59339147	-1419.164072818	-0.007959991
8	3.24824	-1419.17174313	-383.570668393	-1035.59337530	-1419.164043693	-0.007832152
9	3.23761	-1419.17157538	-383.570657346	-1035.59336024	-1419.164017586	-0.007699437
10	3.22622	-1419.17140100	-383.570644004	-1035.59334646	-1419.163990464	-0.007557794
11	3.21462	-1419.17122222	-383.570630451	-1035.59333389	-1419.163964341	-0.007410536
12	3.20227	-1419.17103397	-383.570614416	-1035.59332317	-1419.163937586	-0.007257879
13	3.19026	-1419.17084115	-383.570597950	-1035.59331324	-1419.163911190	-0.007096384
14	3.17803	-1419.17064061	-383.570581487	-1035.59330490	-1419.163886387	-0.006929960
15	3.16553	-1419.17043253	-383.570563285	-1035.59329739	-1419.163860675	-0.006754223
16	3.15295	-1419.17021418	-383.570544667	-1035.59329088	-1419.163835547	-0.006571855
17	3.14003	-1419.16999091	-383.570523464	-1035.59328520	-1419.163808664	-0.006378633
18	3.1273	-1419.16975656	-383.570503267	-1035.59327984	-1419.163783107	-0.006182246
19	3.11409	-1419.16951622	-383.570477351	-1035.59327445	-1419.163751801	-0.005973453
20	3.10088	-1419.16926753	-383.570452342	-1035.59326895	-1419.163721292	-0.005764419
21	3.08734	-1419.16900576	-383.570422567	-1035.59326353	-1419.163686097	-0.005546238
22	3.07368	-1419.16873718	-383.570390692	-1035.59325755	-1419.163648242	-0.005319663
23	3.05963	-1419.16845649	-383.570351459	-1035.59325143	-1419.163602889	-0.005088938
24	3.04583	-1419.16816949	-383.570313959	-1035.59324392	-1419.163557879	-0.004853601
25	3.03168	-1419.16787051	-383.570270527	-1035.59323608	-1419.163506607	-0.004611611
26	3.01739	-1419.16756071	-383.570223068	-1035.59322699	-1419.163450058	-0.004363903
27	3.00278	-1419.16724074	-383.570168118	-1035.59321742	-1419.163385538	-0.004110652
28	2.98794	-1419.16690705	-383.570105396	-1035.59320686	-1419.163312256	-0.003855202
29	2.97272	-1419.16656749	-383.570031653	-1035.59319590	-1419.163227553	-0.003594794
30	2.95767	-1419.16621375	-383.569942368	-1035.59318160	-1419.163123968	-0.003339937
31	2.94231	-1419.16585224	-383.569849706	-1035.59316809	-1419.163017796	-0.003089782
32	2.92655	-1419.16547700	-383.569743594	-1035.59315359	-1419.162897184	-0.002834444
33	2.91058	-1419.16508912	-383.569623615	-1035.59313843	-1419.162762045	-0.002579816
34	2.89435	-1419.16468911	-383.569487239	-1035.59312222	-1419.162609459	-0.002327075
35	2.87789	-1419.16427709	-383.569332793	-1035.59310517	-1419.162437963	-0.002079651
36	2.86118	-1419.16385330	-383.569158658	-1035.59308695	-1419.162245608	-0.001839127
37	2.84423	-1419.16341801	-383.568961892	-1035.59306765	-1419.162029542	-0.001607692
38	2.82703	-1419.16297160	-383.568740281	-1035.59304714	-1419.161787421	-0.001388468
39	2.80958	-1419.16251458	-383.568490554	-1035.59302587	-1419.161516424	-0.001184179
40	2.79188	-1419.16204759	-383.568210283	-1035.59300332	-1419.161213603	-0.000998156
41	2.77393	-1419.16157140	-383.567895995	-1035.59298009	-1419.160876085	-0.000833987
42	2.75573	-1419.16108695	-383.567544257	-1035.59295594	-1419.160500197	-0.000695315

43	2.73728	-1419.16059537	-383.567151379	-1035.59293180	-1419.160083179	-0.000586753
44	2.71858	-1419.16009834	-383.566712678	-1035.59290729	-1419.159619968	-0.000512191
45	2.69964	-1419.15959786	-383.566225337	-1035.59288234	-1419.159107677	-0.000478372
46	2.68046	-1419.15909602	-383.565684667	-1035.59285755	-1419.158542217	-0.000490183
47	2.66105	-1419.15859511	-383.565086939	-1035.59283279	-1419.157919729	-0.000553803
48	2.64143	-1419.15809792	-383.564428941	-1035.59280898	-1419.157237921	-0.000675381
49	2.62159	-1419.15760768	-383.563705274	-1035.59278563	-1419.156490904	-0.000859999
50	2.60156	-1419.15712809	-383.562912727	-1035.59276338	-1419.155676107	-0.001116776
51	2.58135	-1419.15666319	-383.562048273	-1035.59274171	-1419.154789983	-0.001451983
52	2.56096	-1419.15621741	-383.561107998	-1035.59272169	-1419.153829688	-0.001873207
53	2.54042	-1419.15579552	-383.560088150	-1035.59270236	-1419.152790510	-0.002387722
54	2.51972	-1419.15540262	-383.558985219	-1035.59268408	-1419.151669299	-0.003005010
55	2.49889	-1419.15504405	-383.557797103	-1035.59266721	-1419.150464313	-0.003733321
56	2.47793	-1419.15472539	-383.556519327	-1035.59265132	-1419.149170647	-0.004579737
57	2.45686	-1419.15445238	-383.555150391	-1035.59263677	-1419.147787161	-0.005554743
58	2.43569	-1419.15423081	-383.553689166	-1035.59262261	-1419.146311776	-0.006665219
59	2.41443	-1419.15406637	-383.552134595	-1035.59260949	-1419.144744085	-0.007919034
60	2.39312	-1419.15396437	-383.550489767	-1035.59259793	-1419.143087697	-0.009322285
61	2.37175	-1419.15392947	-383.548752632	-1035.59258584	-1419.141338472	-0.010876673
62	2.37175	-1419.15392947	-383.548752632	-1035.59258584	-1419.141338472	-0.012590998
63	2.35034	-1419.15396557	-383.546926460	-1035.59257422	-1419.139500680	-0.014464890
64	2.32893	-1419.15407561	-383.545015619	-1035.59256280	-1419.137578419	-0.016497191
65	2.30752	-1419.15426156	-383.543031134	-1035.59255110	-1419.135582234	-0.018679326
66	2.28612	-1419.15452430	-383.540972163	-1035.59253911	-1419.133511273	-0.021013027
67	2.26476	-1419.15486351	-383.538848043	-1035.59252662	-1419.131374663	-0.023488847
68	2.22224	-1419.15576409	-383.534445022	-1035.59249948	-1419.126944502	-0.028819588
69	2.20111	-1419.15631899	-383.532182732	-1035.59248335	-1419.124666082	-0.031652908
70	2.18012	-1419.15693764	-383.529897810	-1035.59246574	-1419.122363550	-0.034574090
71	2.15928	-1419.15761449	-383.527598655	-1035.59244537	-1419.120044025	-0.037570465
72	2.13861	-1419.15834318	-383.525297177	-1035.59242266	-1419.117719837	-0.040623343
73	2.11816	-1419.15911650	-383.523008496	-1035.59239652	-1419.115405016	-0.043711484
74	2.09795	-1419.15992634	-383.520747399	-1035.59236714	-1419.113114539	-0.046811801
75	2.07804	-1419.16076374	-383.518529093	-1035.59233284	-1419.110861933	-0.049901807
76	2.05848	-1419.16161917	-383.516369419	-1035.59229376	-1419.108663179	-0.052955991
77	2.03932	-1419.16248286	-383.514288743	-1035.59224810	-1419.106536843	-0.055946017
78	2.02064	-1419.16334511	-383.512301639	-1035.59219464	-1419.104496279	-0.058848831
79	2.0025	-1419.16419659	-383.510426550	-1035.59213264	-1419.102559190	-0.061637400
80	1.98501	-1419.16502849	-383.508684756	-1035.59206036	-1419.100745116	-0.064283374
81	1.96824	-1419.16583282	-383.507087538	-1035.59197634	-1419.099063878	-0.066768942
82	1.9523	-1419.16660261	-383.505654465	-1035.59187803	-1419.097532495	-0.069070115
83	1.9373	-1419.16733222	-383.504396245	-1035.59176425	-1419.096160495	-0.071171725
84	1.92333	-1419.16801754	-383.503316234	-1035.59163254	-1419.094948774	-0.073068766
85	1.91046	-1419.16865606	-383.502418265	-1035.59148145	-1419.093899715	-0.074756345
86	1.89876	-1419.16924682	-383.501696369	-1035.59131074	-1419.093007109	-0.076239711
87	1.88822	-1419.16979013	-383.501136130	-1035.59112185	-1419.092257980	-0.077532150
88	1.87883	-1419.17028728	-383.500716299	-1035.59091452	-1419.091630819	-0.078656461
89	1.87052	-1419.17074026	-383.500411492	-1035.59069387	-1419.091105362	-0.079634898
90	1.8632	-1419.17115166	-383.500205043	-1035.59046343	-1419.090668473	-0.080483187

91	1.85679	-1419.17152466	-383.500063984	-1035.59022978	-1419.090293764	-0.081230896
92	1.8512	-1419.17186307	-383.499977695	-1035.58999851	-1419.089976205	-0.081886865
93	1.84636	-1419.17217105	-383.499923578	-1035.58977821	-1419.089701788	-0.082469262
94	1.83866	-1419.17271193	-383.499851228	-1035.58939161	-1419.089242838	-0.083469092
95	1.83576	-1419.17295129	-383.499837762	-1035.58923234	-1419.089070102	-0.083881188
OPT PC	1.80635	-1419.17769006	-383.494003081	-1035.58783087	-1419.081833951	-0.095856109

Table S67. Energy strain analysis of the PPh_3 addition to **3**. Level of theory: SMD(DCM)/MN15/def2-TZVPP. RC refers to reactant complex and PC to product complex. Energies are given in Hartree

Step	P-C	E_{tot}	$S_{\mathbf{3}}$	SP_{PPh3}	$SP_{\mathbf{13}+PPh3}$	E_{tot}- $SP_{\mathbf{3}+PPh3}$
OPT RC	3.72395	-1379.88190702	-344.277896574	-1035.59498328	-1379.872879854	-0.009027166
OPT **3**	-	-344.277940134	-	-	-	-
OPT PPh_3	-	-1035.59514216	-	-	-	-
1	3.60908	-1379.87866994	-344.277844851	-1035.59415865	-1379.872003501	-0.006666499
2	3.59728	-1379.87858417	-344.277840207	-1035.59412369	-1379.871963897	-0.006620103
3	3.58502	-1379.87849524	-344.277834842	-1035.59408980	-1379.871924642	-0.006570358
4	3.57235	-1379.87840248	-344.277830628	-1035.59405576	-1379.871886388	-0.006515612
5	3.55951	-1379.87820774	-344.277823632	-1035.59402307	-1379.871846702	-0.006460298
6	3.54608	-1379.87810514	-344.277817407	-1035.59399058	-1379.871807987	-0.006400013
7	3.53254	-1379.87810500	-344.277810378	-1035.59395838	-1379.871768758	-0.006336242
8	3.51912	-1379.87800057	-344.277805363	-1035.59392621	-1379.871731573	-0.006268997
9	3.50535	-1379.87789205	-344.277798165	-1035.59389562	-1379.871693785	-0.006198265
10	3.49080	-1379.87778064	-344.277788481	-1035.59386495	-1379.871653431	-0.006127209
11	3.47668	-1379.87766689	-344.277781962	-1035.59383343	-1379.871615392	-0.006051498
12	3.46213	-1379.87755108	-344.277771299	-1035.59380271	-1379.871574009	-0.005977071
13	3.44838	-1379.87743242	-344.277767579	-1035.59377051	-1379.871538089	-0.005894331
14	3.43374	-1379.87730888	-344.277753786	-1035.59374061	-1379.871494396	-0.005814484
15	3.41894	-1379.87718190	-344.277746522	-1035.59370960	-1379.871456122	-0.005725778
16	3.40390	-1379.87704915	-344.277734962	-1035.59368057	-1379.871415532	-0.005633618
17	3.38827	-1379.87690969	-344.277724966	-1035.59365156	-1379.871376526	-0.005533164
18	3.37240	-1379.87676469	-344.277714547	-1035.59362362	-1379.871338167	-0.005426523
19	3.35662	-1379.87661377	-344.277707304	-1035.59359542	-1379.871302724	-0.005311046
20	3.34025	-1379.87645564	-344.277693287	-1035.59356911	-1379.871262397	-0.005193243
21	3.32342	-1379.87629189	-344.277677609	-1035.59354318	-1379.871220789	-0.005071101
22	3.30603	-1379.87612118	-344.277657259	-1035.59351863	-1379.871175889	-0.004945291
23	3.28887	-1379.87594414	-344.277640789	-1035.59349435	-1379.871135139	-0.004809001
24	3.27073	-1379.87576012	-344.277605520	-1035.59347166	-1379.871077180	-0.004682940
25	3.25368	-1379.87556881	-344.277596103	-1035.59344755	-1379.871043653	-0.004525157
26	3.23531	-1379.87536941	-344.277553030	-1035.59342727	-1379.870980300	-0.004389110
27	3.21798	-1379.87516006	-344.277542635	-1035.59340430	-1379.870946935	-0.004213125
28	3.19942	-1379.87494004	-344.277502436	-1035.59338557	-1379.870888006	-0.004052034
29	3.18080	-1379.87470978	-344.277474379	-1035.59336611	-1379.870840489	-0.003869291
30	3.16210	-1379.87446955	-344.277442944	-1035.59334727	-1379.870790214	-0.003679336
31	3.14365	-1379.87421889	-344.277415910	-1035.59332732	-1379.870743230	-0.003475660
32	3.12451	-1379.87395854	-344.277376062	-1035.59331050	-1379.870686562	-0.003271978
33	3.10535	-1379.87368676	-344.277337216	-1035.59329625	-1379.870633466	-0.003053294
34	3.08652	-1379.87340501	-344.277296540	-1035.59328865	-1379.870585190	-0.002819820

35	3.06629	-1379.87311228	-344.277224578	-1035.59328455	-1379.870509128	-0.002603152
36	3.04692	-1379.87280754	-344.277183051	-1035.59327238	-1379.870455431	-0.002352109
37	3.02712	-1379.87248910	-344.277113620	-1035.59324927	-1379.870362890	-0.002126210
38	3.00700	-1379.87215651	-344.277047308	-1035.59321975	-1379.870267058	-0.001889452
39	2.98736	-1379.87181005	-344.276991914	-1035.59318715	-1379.870179064	-0.001630986
40	2.96710	-1379.87144989	-344.276907799	-1035.59315683	-1379.870064629	-0.001385261
41	2.94747	-1379.87107288	-344.276852282	-1035.59313889	-1379.869991172	-0.001081708
42	2.92710	-1379.87068197	-344.276741749	-1035.59311625	-1379.869857999	-0.000823971
43	2.90663	-1379.87027196	-344.276656462	-1035.59310444	-1379.869760902	-0.000511058
44	2.88591	-1379.86984726	-344.276529815	-1035.59308118	-1379.869610995	-0.000236265
45	2.86501	-1379.86940437	-344.276405404	-1035.59304024	-1379.869445644	0.000041274
46	2.84392	-1379.86894427	-344.276253873	-1035.59300101	-1379.869254883	0.000310613
47	2.82263	-1379.86846679	-344.276087049	-1035.59298847	-1379.869075519	0.000608729
48	2.80112	-1379.86797211	-344.275893639	-1035.59297776	-1379.868871399	0.000899289
49	2.77941	-1379.86746065	-344.275674028	-1035.59295362	-1379.868627648	0.001166998
50	2.75747	-1379.86693325	-344.275421389	-1035.59292579	-1379.868347179	0.001413929
51	2.73531	-1379.86639108	-344.275133189	-1035.59290558	-1379.868038769	0.001647689
52	2.71292	-1379.86583560	-344.274801590	-1035.59287951	-1379.867681100	0.001845500
53	2.64436	-1379.86469249	-344.273985493	-1035.59282848	-1379.866813973	0.002121483
54	2.62105	-1379.86411017	-344.273487188	-1035.59280367	-1379.866290858	0.002180688
55	2.59751	-1379.86352536	-344.272919768	-1035.59277983	-1379.865699598	0.002174238
56	2.57376	-1379.86294236	-344.272274459	-1035.59275374	-1379.865028199	0.002085839
57	2.54981	-1379.86236633	-344.271544424	-1035.59273017	-1379.864274594	0.001908264
58	2.52567	-1379.86180333	-344.270720707	-1035.59271332	-1379.863434027	0.001630697
59	2.50134	-1379.86126015	-344.269794568	-1035.59270339	-1379.862497958	0.001237808
60	2.47684	-1379.86074450	-344.268757660	-1035.59269840	-1379.861456060	0.000711560
61	2.45220	-1379.86026512	-344.267601335	-1035.59269536	-1379.860296695	0.000031575
62	2.42743	-1379.85983190	-344.266324278	-1035.59269257	-1379.859016848	-0.000815052
63	2.40256	-1379.85945543	-344.264918186	-1035.59268957	-1379.857607756	-0.001847674
64	2.37762	-1379.85914652	-344.263385964	-1035.59268577	-1379.856071734	-0.003074786
65	2.35263	-1379.85891540	-344.261733345	-1035.59268085	-1379.854414195	-0.004501205
66	2.32760	-1379.85872155	-344.258082599	-1035.59266605	-1379.850748649	-0.007972901
67	2.30255	-1379.85877303	-344.256091662	-1035.59265516	-1379.848746822	-0.010026208
68	2.27750	-1379.85893041	-344.254005569	-1035.59264141	-1379.846646979	-0.012283431
69	2.25248	-1379.85919688	-344.251828213	-1035.59262407	-1379.844452283	-0.014744597
70	2.22750	-1379.85957319	-344.249574977	-1035.59260186	-1379.842176837	-0.017396353
71	2.20259	-1379.86005761	-344.247258020	-1035.59257381	-1379.839831830	-0.020225780
72	2.17779	-1379.86064596	-344.244892515	-1035.59253927	-1379.837431785	-0.023214175
73	2.15311	-1379.86133179	-344.242494027	-1035.59249718	-1379.834991207	-0.026340583
74	2.12860	-1379.86210670	-344.240076225	-1035.59244736	-1379.832523585	-0.029583115
75	2.10429	-1379.86296056	-344.237662078	-1035.59238949	-1379.830051568	-0.032908992
76	2.08023	-1379.86388143	-344.235268949	-1035.59232228	-1379.827591229	-0.036290201
77	2.05648	-1379.86485571	-344.232914741	-1035.59224608	-1379.825160821	-0.039694889
78	2.03310	-1379.86586841	-344.230619274	-1035.59215900	-1379.822778274	-0.043090136
79	2.01019	-1379.86690357	-344.228405246	-1035.59206067	-1379.820465916	-0.046437654
80	1.98784	-1379.86794455	-344.226300429	-1035.59194910	-1379.818249529	-0.049695021
81	1.96619	-1379.86897463	-344.224331634	-1035.59182393	-1379.816155564	-0.052819066
82	1.94541	-1379.86997764	-344.222523801	-1035.59168304	-1379.814206841	-0.055770799

83	1.92570	-1379.87093935	-344.220897668	-1035.59152539	-1379.812423058	-0.058516292
84	1.90729	-1379.87184804	-344.219499194	-1035.59135024	-1379.810849434	-0.060998606
85	1.89039	-1379.87269536	-344.218291057	-1035.59115663	-1379.809447687	-0.063247673
86	1.87481	-1379.87347924	-344.217081134	-1035.59094640	-1379.808027534	-0.065451706
87	1.86199	-1379.87419875	-344.216676707	-1035.59072299	-1379.807399697	-0.066799053
88	1.85077	-1379.87485961	-344.216141047	-1035.59048642	-1379.806627467	-0.068232143
89	1.84126	-1379.87546207	-344.215803129	-1035.59024805	-1379.806051179	-0.069410891
90	1.83409	-1379.87601994	-344.215768812	-1035.59001009	-1379.805778902	-0.070241038
91	1.82723	-1379.87653003	-344.215436311	-1035.58978616	-1379.805222471	-0.071307559
92	1.82160	-1379.87700601	-344.215182459	-1035.58957964	-1379.804762099	-0.072243911
93	1.81609	-1379.87744738	-344.214874859	-1035.58939388	-1379.804268739	-0.073178641
94	1.81233	-1379.87785764	-344.214784753	-1035.58922138	-1379.804006133	-0.073851507
95	1.80912	-1379.87824449	-344.214759958	-1035.58907278	-1379.803832738	-0.074411752
96	1.80723	-1379.87860986	-344.214772608	-1035.58893917	-1379.803711778	-0.074898082
97	1.80451	-1379.87895461	-344.214570928	-1035.58884132	-1379.803412248	-0.075542362
98	1.80210	-1379.87928401	-344.214242395	-1035.58876209	-1379.803004485	-0.076279525
99	1.79891	-1379.87959903	-344.213833411	-1035.58870672	-1379.802540131	-0.077058899
100	1.79847	-1379.87989993	-344.213902223	-1035.58863491	-1379.802537133	-0.077362797
101	1.79706	-1379.88018844	-344.213650507	-1035.58859709	-1379.802247597	-0.077940843
102	1.79569	-1379.88046455	-344.213428750	-1035.58857086	-1379.801999610	-0.078464940
103	1.79438	-1379.88072930	-344.213122576	-1035.58855430	-1379.801676876	-0.079052424
104	1.79345	-1379.88098229	-344.213002297	-1035.58853954	-1379.801541837	-0.079440453
105	1.79338	-1379.88122606	-344.212903429	-1035.58852588	-1379.801429309	-0.079796751
106	1.79114	-1379.88145951	-344.212357914	-1035.58854063	-1379.800898544	-0.080560966
107	1.79038	-1379.88168365	-344.212182476	-1035.58853672	-1379.800719196	-0.080964454
108	1.78963	-1379.88189784	-344.211926649	-1035.58853886	-1379.800465509	-0.081432331
109	1.78924	-1379.88210279	-344.211845969	-1035.58853855	-1379.800384519	-0.081718271
110	1.78935	-1379.88229938	-344.211753758	-1035.58854049	-1379.800294248	-0.082005132
111	1.78743	-1379.88248766	-344.211217340	-1035.58856283	-1379.799780170	-0.082707490
112	1.78705	-1379.88266797	-344.211100685	-1035.58856434	-1379.799665025	-0.083002945
113	1.78632	-1379.88283915	-344.210791306	-1035.58857362	-1379.799364926	-0.083474224
114	1.78527	-1379.88300294	-344.210468159	-1035.58858087	-1379.799049029	-0.083953911
115	1.78605	-1379.88315915	-344.210617451	-1035.58857740	-1379.799194851	-0.083964299
116	1.78527	-1379.88330775	-344.210272625	-1035.58859213	-1379.798864755	-0.084442995
117	1.78427	-1379.88344803	-344.209903264	-1035.58860087	-1379.798504134	-0.084943896
118	1.78403	-1379.88358112	-344.209729985	-1035.58860656	-1379.798336545	-0.085244575
119	1.78434	-1379.88370675	-344.209777900	-1035.58860981	-1379.798387710	-0.085319040
120	1.78360	-1379.88382433	-344.209391731	-1035.58862388	-1379.798015611	-0.085808719
121	1.78376	-1379.88393482	-344.209403868	-1035.58862117	-1379.798025038	-0.085909782
122	1.78326	-1379.88403794	-344.209040577	-1035.58863788	-1379.797678457	-0.086359483
123	1.78243	-1379.88413445	-344.208676183	-1035.58864057	-1379.797316753	-0.086817697
124	1.78268	-1379.88422504	-344.208585739	-1035.58864572	-1379.797231459	-0.086993581
125	1.78237	-1379.88431024	-344.208394727	-1035.58865235	-1379.797047077	-0.087263163
126	1.78304	-1379.88439035	-344.208537963	-1035.58865137	-1379.797189333	-0.087201017
OPT PC	1.78009	-1379.88584857	-344.204581444	-1035.58845444	-1379.793035884	-0.092812686

6.3.15 Intrinsic barriers

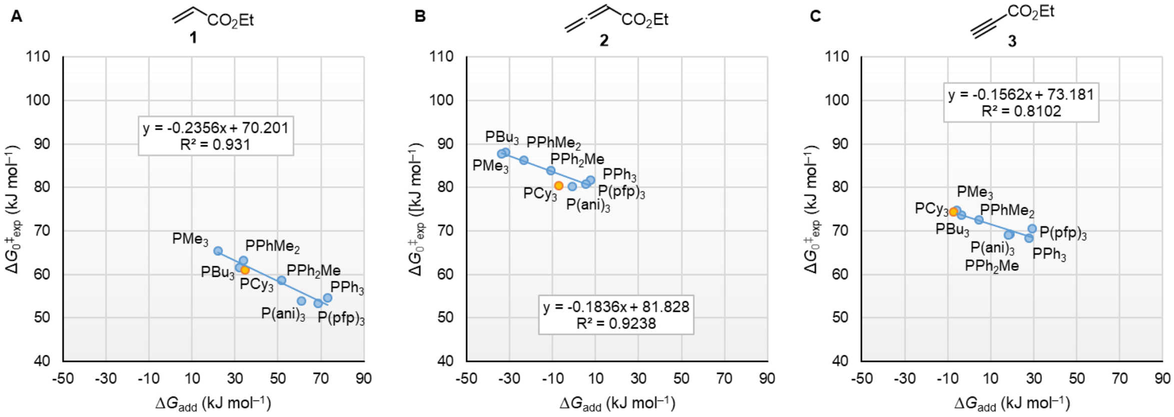

Fig. S30. Correlations of $\Delta G_0^{\ddagger}{}_{exp}$ with ΔG_{add} for phosphine additions to (A) **1**, (B) **2**, and (C) **3** (with ΔG_{add} from Table 4 and $\Delta G_0^{\ddagger}{}_{exp}$ from Table 5).

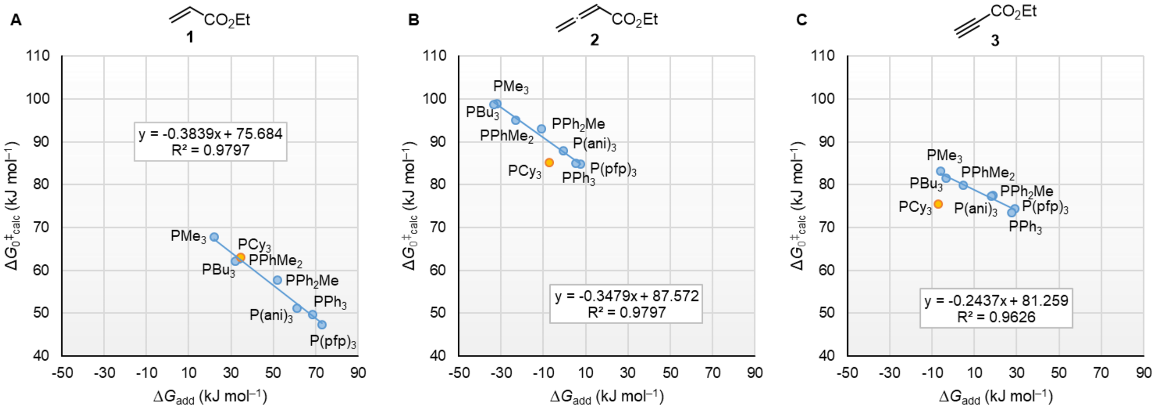

Fig. S31. Correlations of $\Delta G_0^{\ddagger}{}_{calc}$ with ΔG_{add} for phosphine additions to (A) **1**, (B) **2**, and (C) **3** (with ΔG_{add} from Table 4 and $\Delta G_0^{\ddagger}{}_{calc}$ from Table 5).

6.4 Cartesian Coordinates

Michael-acceptors

1_1

6	-2.982423000	-0.320524000	-0.000036000
6	-1.728742000	-0.756650000	-0.000007000
6	-0.599579000	0.202251000	0.000054000
8	-0.705334000	1.409762000	0.000017000
8	0.576243000	-0.426928000	0.000017000
6	1.744375000	0.408834000	0.000002000
6	2.951852000	-0.491906000	-0.000027000
1	-3.820041000	-1.005077000	-0.000073000
1	-3.193402000	0.742143000	-0.000020000
1	-1.477315000	-1.809394000	-0.000020000
1	1.714308000	1.049445000	0.882216000
1	1.714276000	1.049459000	-0.882201000
1	3.858742000	0.112372000	-0.000040000
1	2.961645000	-1.126826000	0.885885000
1	2.961612000	-1.126814000	-0.885947000

1_2

6	2.885530000	0.098832000	0.260907000
6	1.765469000	-0.607025000	0.164677000
6	0.483202000	0.058849000	-0.163986000
8	0.344407000	1.246463000	-0.362278000
8	-0.517865000	-0.821760000	-0.223940000
6	-1.826121000	-0.302536000	-0.515907000
6	-2.474939000	0.251787000	0.730400000
1	3.831109000	-0.369665000	0.498716000
1	2.873502000	1.170221000	0.100155000
1	1.735531000	-1.678041000	0.317829000
1	-2.381930000	-1.150512000	-0.909879000
1	-1.737125000	0.457085000	-1.291734000
1	-1.904752000	1.093783000	1.121451000
1	-3.482310000	0.597199000	0.496609000
1	-2.545196000	-0.517131000	1.500061000

1_3

6	-2.176396000	-1.380293000	0.000007000
6	-2.006767000	-0.062923000	-0.000009000
6	-0.681970000	0.595386000	0.000022000
8	-0.542565000	1.801006000	-0.000032000
8	0.343587000	-0.254023000	0.000021000
6	1.654674000	0.332217000	0.000000000
6	2.657164000	-0.792191000	0.000000000
1	-3.167593000	-1.814547000	-0.000014000
1	-1.331219000	-2.056411000	0.000043000
1	-2.842653000	0.625109000	-0.000045000
1	1.753671000	0.965638000	0.882298000
1	1.753652000	0.965621000	-0.882312000
1	2.539435000	-1.416124000	0.885970000
1	2.539415000	-1.416142000	-0.885955000
1	3.666881000	-0.382180000	-0.000015000

2_1

6	-2.770591000	-1.469120000	0.000020000
6	-2.168561000	-0.320401000	0.000018000
6	-1.570627000	0.845370000	-0.000001000
6	-0.102242000	1.003021000	-0.000023000
8	0.446282000	2.084627000	-0.000037000
8	0.558455000	-0.152747000	0.000001000
6	1.992110000	-0.062743000	-0.000007000
6	2.536492000	-1.467296000	0.000025000
1	-3.026710000	-1.964356000	-0.929612000
1	-3.026704000	-1.964356000	0.929654000
1	-2.138556000	1.767609000	-0.000006000
1	2.307063000	0.495348000	0.882479000
1	2.307055000	0.495304000	-0.882524000
1	3.625884000	-1.436982000	0.000019000
1	2.207292000	-2.010322000	-0.885846000
1	2.207300000	-2.010278000	0.885927000

2_2

6	-3.733272000	-0.007267000	0.000304000
6	-2.498877000	-0.403276000	0.000132000
6	-1.249596000	-0.797580000	-0.000095000
6	-0.145572000	0.185748000	-0.000457000
8	-0.271778000	1.390401000	-0.000114000
8	1.039089000	-0.427423000	-0.000203000
6	2.193864000	0.427082000	-0.000002000
6	3.415015000	-0.454900000	0.000276000
1	-4.263524000	0.163920000	0.930132000
1	-4.263696000	0.164204000	-0.929374000
1	-0.989784000	-1.849062000	-0.000144000
1	2.153743000	1.066964000	0.882231000
1	2.154093000	1.066895000	-0.882302000
1	3.434645000	-1.089555000	-0.885613000
1	3.434291000	-1.089486000	0.886222000
1	4.312365000	0.163461000	0.000430000

2_3

6	3.076147000	-1.059472000	0.065909000
6	2.237727000	-0.071444000	0.119424000
6	1.399820000	0.934120000	0.177310000
6	-0.048926000	0.798931000	-0.076820000
8	-0.816523000	1.735998000	-0.018621000
8	-0.419281000	-0.444323000	-0.380421000
6	-1.819708000	-0.664953000	-0.618830000
6	-2.570944000	-0.820335000	0.682100000
1	3.578242000	-1.314938000	-0.860231000
1	3.292231000	-1.655560000	0.945125000
1	1.738545000	1.933669000	0.420934000
1	-1.860503000	-1.575501000	-1.212500000
1	-2.210440000	0.162716000	-1.209638000
1	-2.159630000	-1.643996000	1.266049000
1	-2.516784000	0.094170000	1.271835000
1	-3.619926000	-1.035047000	0.476208000

3_1

6	2.953204000	-0.918783000	0.000080000
6	1.907471000	-0.329698000	-0.000169000
6	0.669868000	0.430206000	0.000008000
8	0.630990000	1.638161000	0.000005000
8	-0.393487000	-0.359674000	0.000032000
6	-1.673336000	0.306695000	0.000034000
6	-2.738409000	-0.757078000	-0.000023000
1	3.882301000	-1.444585000	0.000123000
1	-1.728625000	0.943076000	-0.883354000
1	-1.728653000	0.943009000	0.883468000
1	-3.720662000	-0.285393000	-0.000061000
1	-2.658549000	-1.386017000	-0.886332000
1	-2.658630000	-1.386031000	0.886282000

3_2

6	-3.026975000	-0.468345000	0.278450000
6	-1.889636000	-0.134924000	0.089034000
6	-0.528346000	0.321521000	-0.131778000
8	-0.211226000	1.487632000	-0.117501000
8	0.296405000	-0.691851000	-0.354329000
6	1.684870000	-0.359699000	-0.571295000
6	2.392855000	-0.135831000	0.742835000
1	-4.038673000	-0.766279000	0.444899000
1	1.735505000	0.518879000	-1.212822000
1	2.089655000	-1.216166000	-1.104486000
1	3.448147000	0.063976000	0.556097000
1	1.970630000	0.717453000	1.272479000
1	2.316700000	-1.020439000	1.374996000

3_3

6	2.056657000	1.633837000	0.000000000
6	1.457045000	0.593495000	-0.000008000
6	0.783743000	-0.696192000	0.000003000
8	1.397134000	-1.736091000	-0.000018000
8	-0.542160000	-0.682260000	0.000038000
6	-1.261793000	0.568821000	0.000012000
6	-2.730729000	0.238313000	-0.000021000
1	2.593019000	2.557084000	-0.000010000
1	-0.979737000	1.136517000	-0.887200000
1	-0.979777000	1.136526000	0.887231000
1	-3.309933000	1.161220000	-0.000052000
1	-2.996430000	-0.337106000	-0.886323000
1	-2.996480000	-0.337080000	0.886281000

Phosphines

PMe3_1

6	2.953204000	-0.918783000	0.000080000
6	1.907471000	-0.329698000	-0.000169000
6	0.669868000	0.430206000	0.000008000
8	0.630990000	1.638161000	0.000005000
8	-0.393487000	-0.359674000	0.000032000
6	-1.673336000	0.306695000	0.000034000
6	-2.738409000	-0.757078000	-0.000023000
1	3.882301000	-1.444585000	0.000123000
1	-1.728625000	0.943076000	-0.883354000
1	-1.728653000	0.943009000	0.883468000
1	-3.720662000	-0.285393000	-0.000061000
1	-2.658549000	-1.386017000	-0.886332000
1	-2.658630000	-1.386031000	0.886282000

PPhMe2_1

15	1.642440000	-0.171555000	-0.565896000
6	2.127633000	-1.287635000	0.796042000
6	-0.138403000	-0.022981000	-0.229314000
6	-0.920188000	-1.181999000	-0.298399000

6	-2.289979000	-1.133418000	-0.084413000
6	-2.910899000	0.083043000	0.186488000
6	-2.149285000	1.242693000	0.242594000
6	-0.772882000	1.190591000	0.039366000
6	2.292240000	1.398674000	0.084432000
1	1.696240000	-2.277250000	0.643594000
1	3.213880000	-1.394071000	0.796301000
1	1.804486000	-0.901286000	1.765341000
1	-0.447556000	-2.132042000	-0.525748000
1	-2.875544000	-2.042884000	-0.134105000
1	-3.980306000	0.124516000	0.348909000
1	-2.623964000	2.193836000	0.449473000
1	-0.197774000	2.106583000	0.092497000
1	2.032410000	2.230209000	-0.571068000
1	1.931196000	1.606589000	1.094689000
1	3.380910000	1.325329000	0.107785000

PPhMe2_2

15	1.625943000	-0.648305000	-0.000021000
6	2.224085000	0.373922000	1.389093000
6	-0.152144000	-0.235719000	-0.000022000
6	-1.079668000	-1.277431000	-0.000073000
6	-2.446982000	-1.016795000	-0.000070000
6	-2.901214000	0.295410000	-0.000017000
6	-1.986236000	1.345377000	0.000035000
6	-0.623728000	1.080020000	0.000033000
6	2.224141000	0.374097000	-1.388982000
1	1.821290000	-0.003181000	2.329799000
1	3.312514000	0.303672000	1.434634000
1	1.946765000	1.424793000	1.276708000
1	-0.724319000	-2.301948000	-0.000116000
1	-3.153542000	-1.837167000	-0.000110000
1	-3.963783000	0.502974000	-0.000015000
1	-2.337257000	2.369734000	0.000077000
1	0.078881000	1.906504000	0.000076000
1	1.821381000	-0.002884000	-2.329752000
1	1.946829000	1.424956000	-1.276476000
1	3.312571000	0.303842000	-1.434490000

PPh2Me_1

15	-0.018558000	1.382310000	-0.705232000
6	1.400440000	0.311023000	-0.321262000
6	-1.402963000	0.308157000	-0.201509000
6	0.006470000	2.555373000	0.689845000
6	-1.377233000	-0.424994000	0.988251000
6	-2.462888000	-1.204911000	1.359237000
6	-3.590847000	-1.261893000	0.542969000
6	-3.626874000	-0.539129000	-0.641854000
6	-2.534605000	0.241051000	-1.012301000
6	1.472730000	-0.942087000	-0.940471000
6	2.556646000	-1.779982000	-0.726480000
6	3.600051000	-1.373999000	0.102304000
6	3.545041000	-0.128735000	0.713071000
6	2.451549000	0.708395000	0.505008000
1	0.822831000	3.268540000	0.571069000
1	-0.929719000	3.114914000	0.673108000
1	0.102950000	2.049975000	1.653511000
1	-0.497252000	-0.385778000	1.622396000
1	-2.432900000	-1.770231000	2.282183000
1	-4.437065000	-1.872191000	0.831979000
1	-4.500247000	-0.583511000	-1.280042000
1	-2.558109000	0.802479000	-1.939527000
1	0.665417000	-1.262902000	-1.591051000
1	2.590886000	-2.749414000	-1.207477000
1	4.448399000	-2.025529000	0.268142000
1	4.351600000	0.195444000	1.358799000
1	2.426471000	1.673948000	0.994546000

PPh3_1

15	0.000659000	0.001392000	-1.231171000
6	-0.050698000	-1.634047000	-0.436473000
6	1.442281000	0.773932000	-0.435775000
6	-1.389925000	0.862097000	-0.434737000
6	-1.768306000	2.090386000	-0.984832000
6	-2.797126000	2.833147000	-0.421812000
6	-3.477206000	2.346046000	0.690084000
6	-3.118612000	1.119384000	1.236039000
6	-2.077770000	0.382379000	0.680241000
6	2.695041000	0.477718000	-0.981224000
6	3.853966000	0.994788000	-0.418509000
6	3.773508000	1.835120000	0.687786000
6	2.532066000	2.147905000	1.228315000
6	1.372033000	1.617098000	0.673200000
6	0.719649000	-1.997901000	0.668102000
6	0.598714000	-3.267950000	1.223064000
6	-0.298391000	-4.183819000	0.686840000
6	-1.071063000	-3.830416000	-0.415206000
6	-0.939015000	-2.568247000	-0.977696000
1	-1.248263000	2.463829000	-1.860841000
1	-3.075001000	3.785352000	-0.855500000
1	-4.286407000	2.918412000	1.125343000
1	-3.646333000	0.734954000	2.099780000
1	-1.801532000	-0.568347000	1.120690000
1	2.757170000	-0.165629000	-1.852900000
1	4.817483000	0.751276000	-0.847798000
1	4.674790000	2.247823000	1.122723000
1	2.464083000	2.803343000	2.087467000
1	0.410606000	1.860880000	1.109632000
1	1.415833000	-1.289581000	1.101419000
1	1.204095000	-3.538844000	2.078907000
1	-0.392083000	-5.170697000	1.121751000
1	-1.767891000	-4.540818000	-0.841494000
1	-1.531308000	-2.298545000	-1.846044000

Pani3_1

15	0.004573000	-0.000035000	1.480450000
6	-0.752235000	-1.450780000	0.691724000
6	1.637079000	0.070425000	0.687635000
6	-0.876449000	1.377422000	0.689362000
6	-0.755727000	2.638239000	1.270624000
6	-1.359618000	3.761782000	0.716261000
6	-2.124745000	3.624824000	-0.441109000
6	-2.268883000	2.365339000	-1.028637000
6	-1.649569000	1.261038000	-0.471166000
6	2.670618000	-0.655725000	1.276437000
6	3.945805000	-0.696624000	0.722504000
6	4.207127000	0.022988000	-0.442820000
6	3.186444000	0.768976000	-1.037702000
6	1.920526000	0.787166000	-0.480208000
6	-0.255875000	-2.079052000	-0.455504000
6	-0.907234000	-3.166208000	-1.010925000
6	-2.083220000	-3.652420000	-0.434636000
6	-2.593102000	-3.041728000	0.710584000
6	-1.917998000	-1.958534000	1.262751000
8	5.411835000	0.059293000	-1.053795000
8	-2.761616000	4.649135000	-1.050483000
8	-2.656442000	-4.714194000	-1.042993000
6	6.469551000	-0.685912000	-0.482610000
6	-2.638525000	5.939404000	-0.484290000
6	-3.849091000	-5.233980000	-0.488467000
1	-0.173475000	2.751005000	2.179342000
1	-1.239135000	4.723693000	1.193702000
1	-2.870297000	2.276249000	-1.924559000
1	-1.768963000	0.294141000	-0.946338000
1	2.478754000	-1.206466000	2.191628000
1	4.721076000	-1.273451000	1.206769000
1	3.408549000	1.325729000	-1.939444000
1	1.141541000	1.367518000	-0.960979000
1	0.651072000	-1.713428000	-0.923205000
1	-0.522944000	-3.654680000	-1.897505000
1	-3.496790000	-3.403727000	1.180160000
1	-2.314025000	-1.497156000	2.161602000
1	7.336840000	-0.531001000	-1.118709000
1	6.697342000	-0.338199000	0.527698000
1	6.230641000	-1.751553000	-0.451985000
1	-3.209768000	6.611662000	-1.118826000
1	-3.046334000	5.966871000	0.529018000
1	-1.595355000	6.263626000	-0.462852000
1	-3.687763000	-5.591058000	0.531510000
1	-4.643697000	-4.484000000	-0.486730000
1	-4.145337000	-6.068957000	-1.117734000

Pani3_2

15	-0.095693000	0.040753000	1.527373000
6	0.738510000	-1.423874000	0.848028000
6	0.754304000	1.407822000	0.690348000
6	-1.701784000	-0.021862000	0.678557000
6	-2.753949000	0.706414000	1.230024000
6	-4.010323000	0.746869000	0.633503000
6	-4.232323000	0.025800000	-0.538509000
6	-3.192217000	-0.721280000	-1.098670000
6	-1.947140000	-0.740463000	-0.497982000
6	1.740318000	2.092724000	1.395544000
6	2.472028000	3.126059000	0.816400000
6	2.202476000	3.497271000	-0.499097000
6	1.208147000	2.826369000	-1.219044000
6	0.499594000	1.796525000	-0.630823000
6	1.799206000	-1.388478000	-0.049166000
6	2.401064000	-2.558682000	-0.513252000
6	1.929483000	-3.793623000	-0.079780000
6	0.863839000	-3.846517000	0.825954000
6	0.290139000	-2.679064000	1.286260000
8	2.844900000	4.490932000	-1.149938000
8	-5.415356000	-0.011486000	-1.189688000
8	2.440088000	-4.981882000	-0.473943000
6	3.855994000	5.194567000	-0.454674000
6	-6.492242000	0.731839000	-0.653096000
6	3.520934000	-4.967763000	-1.385142000
1	-2.592641000	1.257840000	2.150397000
1	-4.801179000	1.324849000	1.090456000
1	-3.384137000	-1.278184000	-2.007184000

1	-1.151957000	-1.322477000	-0.950169000
1	1.945806000	1.816056000	2.424077000
1	3.230857000	3.633637000	1.394873000
1	1.010808000	3.132050000	-2.238835000
1	-0.264067000	1.284868000	-1.205667000
1	2.176988000	-0.437143000	-0.404670000
1	3.225747000	-2.490834000	-1.208896000
1	0.511813000	-4.814548000	1.159330000
1	-0.528778000	-2.734210000	1.996626000
1	4.242928000	5.941268000	-1.142722000
1	3.452783000	5.693367000	0.429762000
1	4.666853000	4.526489000	-0.154753000
1	-7.338251000	0.574992000	-1.316793000
1	-6.751304000	0.384117000	0.349742000
1	-6.256116000	1.797956000	-0.615575000
1	3.237581000	-4.492318000	-2.327452000
1	4.385240000	-4.447782000	-0.964817000
1	3.783016000	-6.005728000	-1.572059000

Pani3_3

15	0.000358000	-0.000419000	-1.710164000
6	-1.155592000	-1.155492000	-0.918840000
6	-0.422644000	1.578205000	-0.919290000
6	1.578976000	-0.424164000	-0.919729000
6	2.747118000	0.103984000	-1.486342000
6	3.987482000	-0.139631000	-0.929783000
6	4.097224000	-0.944278000	0.208720000
6	2.949870000	-1.490917000	0.779073000
6	1.705421000	-1.222146000	0.212951000
6	-1.479713000	2.313797000	-1.472913000
6	-1.890407000	3.509147000	-0.916306000
6	-1.234153000	4.018315000	0.208911000
6	-0.171341000	3.310624000	0.765245000
6	0.219393000	2.098482000	0.199751000
6	-1.923523000	-0.860153000	0.202937000
6	-2.778314000	-1.803776000	0.769116000
6	-2.864271000	-3.076765000	0.210359000
6	-2.098627000	-3.389960000	-0.917352000
6	-1.267960000	-2.437513000	-1.474324000
8	-1.687803000	5.198700000	0.685135000
8	5.346569000	-1.144491000	0.682846000
8	-3.661181000	-4.059073000	0.685655000
6	-1.041774000	5.749203000	1.816233000
6	5.499188000	-1.958691000	1.828897000
6	-4.455426000	-3.778017000	1.821405000
1	2.675699000	0.717746000	-2.378310000
1	4.889238000	0.271252000	-1.365738000
1	3.010380000	-2.119684000	1.656270000
1	0.822060000	-1.648981000	0.673975000
1	-1.986762000	1.935575000	-2.354608000
1	-2.709487000	4.074983000	-1.341799000
1	0.354412000	3.687284000	1.631354000
1	1.043282000	1.557088000	0.650395000
1	-1.863423000	0.123288000	0.654875000
1	-3.364489000	-1.536646000	1.637171000
1	-2.182500000	-4.381183000	-1.344555000
1	-0.689896000	-2.687282000	-2.358062000
1	-1.548377000	6.684575000	2.038195000
1	-1.120033000	5.083492000	2.679083000
1	0.012320000	5.949958000	1.610202000
1	6.563757000	-1.994252000	2.044055000
1	4.971889000	-1.535650000	2.687316000
1	5.134534000	-2.972089000	1.644886000
1	-3.835110000	-3.521708000	2.683680000
1	-5.153226000	-2.960926000	1.623256000
1	-5.015895000	-4.683152000	2.039410000

Ppfp3_1

15	0.001774000	-0.000195000	1.410656000
6	-0.749795000	1.452724000	0.616063000
6	-0.223359000	2.097757000	-0.503068000
6	-1.939390000	1.932282000	1.173202000
6	-0.868161000	3.194544000	-1.063164000
1	0.698150000	1.746797000	-0.950976000
6	-2.605143000	3.015855000	0.619457000
1	-2.350753000	1.448370000	2.052376000
6	-2.048781000	3.625871000	-0.490457000
1	-0.470183000	3.706601000	-1.928867000
1	-3.528157000	3.393675000	1.038088000
6	-0.882086000	-1.375625000	0.614359000
6	-0.722632000	-2.642696000	1.183143000
6	-1.685298000	-1.243003000	-0.519116000
6	-1.330116000	-3.759925000	0.628569000
1	-0.112745000	-2.755822000	2.072562000
6	-2.314310000	-2.348013000	-1.080040000
1	-1.826113000	-0.270655000	-0.975309000
6	-2.118000000	-3.583817000	-0.494528000
1	-1.211023000	-4.746116000	1.056391000
1	-2.942918000	-2.260318000	-1.956143000
6	1.634732000	-0.076656000	0.614353000
6	1.917790000	-0.827997000	-0.526690000
6	2.656215000	0.683626000	1.191602000
6	3.188876000	-0.819133000	-1.088747000
1	1.143218000	-1.427430000	-0.989520000
6	3.927227000	0.717099000	0.636554000
1	2.452831000	1.258506000	2.088124000
6	4.164866000	-0.041711000	-0.495176000
1	3.423939000	-1.398669000	-1.971322000
1	4.724784000	1.304717000	1.070734000
9	-2.722190000	-4.657880000	-1.033282000
9	-2.679529000	4.685085000	-1.028204000
9	5.396891000	-0.027630000	-1.034500000

PCy3_1

15	0.106151000	0.386465000	-0.645503000
6	-1.685455000	0.139043000	-0.290204000
6	0.660744000	1.430814000	0.780386000
6	0.845271000	-1.278370000	-0.276487000
6	-0.208044000	2.674906000	0.987396000
6	-0.081751000	3.666532000	-0.163183000
6	1.372845000	4.095145000	-0.323625000
6	2.272413000	2.885585000	-0.551469000
6	2.119536000	1.862334000	0.571119000
6	-2.036825000	-0.454262000	1.071172000
6	-3.549121000	-0.503230000	1.268932000
6	-4.208147000	-1.315549000	0.160784000
6	-3.857272000	-0.743667000	-1.207560000
6	-2.345273000	-0.680614000	-1.401583000
6	1.292491000	-1.564263000	1.157574000
6	1.781985000	-3.003111000	1.296874000
6	2.933459000	-3.277957000	0.338349000
6	2.509068000	-2.994628000	-1.096715000
6	1.999477000	-1.565519000	-1.243407000
1	-2.113686000	1.150119000	-0.347988000
1	0.602032000	0.826357000	1.689066000
1	0.038784000	-1.986670000	-0.518169000
1	-1.250928000	2.397296000	1.156724000
1	0.136787000	3.168606000	1.904735000
1	-0.429379000	3.201921000	-1.093185000
1	-0.718236000	4.535221000	0.018717000
1	1.477398000	4.807048000	-1.145221000
1	1.689193000	4.610769000	0.591378000
1	2.007688000	2.419803000	-1.505754000
1	3.316982000	3.196150000	-0.626154000
1	2.480092000	2.307456000	1.506528000
1	2.749927000	0.990525000	0.371044000
1	-1.641351000	-1.476566000	1.118975000
1	-1.561910000	0.113678000	1.876185000
1	-3.791393000	-0.922501000	2.248165000
1	-3.943818000	0.519611000	1.249331000
1	-3.849147000	-2.350012000	0.217883000
1	-5.291373000	-1.344341000	0.297269000
1	-4.316024000	-1.338153000	-2.000834000
1	-4.266484000	0.270081000	-1.287078000
1	-2.098993000	-0.254188000	-2.377783000
1	-1.942663000	-1.701100000	-1.386784000
1	2.114869000	-0.888426000	1.419601000
1	0.484370000	-1.370288000	1.865413000
1	2.085623000	-3.198050000	2.328049000
1	0.954666000	-3.686094000	1.069898000
1	3.775860000	-2.625956000	0.598119000
1	3.281883000	-4.308089000	0.440077000
1	3.336419000	-3.174787000	-1.786881000
1	1.703614000	-3.685636000	-1.372513000
1	1.676920000	-1.376654000	-2.270347000
1	2.817595000	-0.863250000	-1.036057000

PCy3_2

15	0.027058000	-0.128919000	-1.038806000
6	-0.263882000	1.546478000	-0.291171000
6	1.422384000	-0.823160000	-0.057364000
6	-1.430520000	-1.096262000	-0.457973000
6	2.726623000	-0.184851000	-0.544024000
6	3.929271000	-0.725642000	0.219709000
6	4.013320000	-2.239284000	0.068184000
6	2.723049000	-2.895181000	0.542810000
6	1.509918000	-2.342780000	-0.200309000
6	0.220387000	2.649654000	-1.238139000
6	-0.170029000	4.032106000	-0.727317000
6	0.363849000	4.264840000	0.680126000
6	-0.125986000	3.172850000	1.622439000
6	0.286203000	1.794060000	1.114476000
6	-2.689840000	-0.679348000	-1.221564000
6	-3.863088000	-1.590063000	-0.872762000
6	-4.122412000	-1.592978000	0.628953000
6	-2.868723000	-2.004988000	1.391174000
6	-1.702586000	-1.084234000	1.043349000
1	-1.359338000	1.628642000	-0.234090000
1	1.294856000	-0.591452000	1.007027000
1	-1.208764000	-2.129192000	-0.759489000

1	2.843743000	-0.414293000	-1.610851000
1	2.673353000	0.904346000	-0.462077000
1	4.847274000	-0.251440000	-0.134322000
1	3.824443000	-0.473900000	1.281676000
1	4.170882000	-2.481698000	-0.989232000
1	4.868670000	-2.635764000	0.619530000
1	2.775210000	-3.979337000	0.420233000
1	2.598074000	-2.699478000	1.614277000
1	0.601809000	-2.815440000	0.181204000
1	1.582611000	-2.596885000	-1.266207000
1	1.312742000	2.587954000	-1.325097000
1	-0.183913000	2.481856000	-2.239536000
1	0.191876000	4.804296000	-1.409951000
1	-1.263955000	4.105168000	-0.709571000
1	1.459890000	4.247555000	0.654830000
1	0.066879000	5.250358000	1.045443000
1	0.262254000	3.333013000	2.630958000
1	-1.219739000	3.217891000	1.687157000
1	-0.049107000	1.019932000	1.807026000
1	1.381061000	1.744230000	1.088475000
1	-2.950809000	0.352325000	-0.955412000
1	-2.497327000	-0.692999000	-2.297798000
1	-4.758090000	-1.278887000	-1.416125000
1	-3.627678000	-2.610330000	-1.196863000
1	-4.413118000	-0.583392000	0.942816000
1	-4.955346000	-2.256218000	0.872161000
1	-3.053284000	-1.994828000	2.467826000
1	-2.603923000	-3.033573000	1.118987000
1	-0.805685000	-1.370836000	1.600531000
1	-1.959039000	-0.061132000	1.344981000

PCy3_3

15	0.094641000	0.062457000	-0.923813000
6	-0.809117000	-1.457741000	-0.393284000
6	-0.688850000	1.415066000	0.062295000
6	1.724604000	-0.080145000	-0.039423000
6	-2.121069000	1.668193000	-0.409021000
6	-2.777384000	2.810163000	0.359558000
6	-1.957869000	4.087046000	0.236775000
6	-0.539017000	3.846528000	0.732743000
6	0.125741000	2.707168000	-0.033621000
6	-1.985356000	-1.819666000	-1.305191000
6	-2.520971000	-3.202938000	-0.947024000
6	-2.931628000	-3.266182000	0.519880000
6	-1.764637000	-2.893462000	1.427437000
6	-1.233032000	-1.508682000	1.072422000
6	2.786817000	0.655495000	-0.867568000
6	4.134145000	0.685959000	-0.157845000
6	4.613710000	-0.732680000	0.118431000
6	3.569825000	-1.498191000	0.920451000
6	2.212375000	-1.505719000	0.221211000
1	-0.066575000	-2.251169000	-0.553823000
1	-0.710123000	1.109717000	1.117548000
1	1.624465000	0.422133000	0.934095000
1	-2.098151000	1.914728000	-1.478503000
1	-2.719075000	0.761367000	-0.304600000
1	-3.796134000	2.967159000	-0.001672000
1	-2.850399000	2.532533000	1.417762000
1	-1.923661000	4.391198000	-0.815879000
1	-2.427140000	4.901867000	0.792286000
1	0.061217000	4.754782000	0.643952000
1	-0.572956000	3.586804000	1.797272000
1	1.135336000	2.550285000	0.351965000
1	0.224056000	2.987101000	-1.090927000
1	-2.791450000	-1.087677000	-1.194341000
1	-1.668238000	-1.787661000	-2.350778000
1	-3.363588000	-3.462397000	-1.591888000
1	-1.734669000	-3.944874000	-1.129509000
1	-3.752752000	-2.559780000	0.690736000
1	-3.308173000	-4.260663000	0.768914000
1	-2.068191000	-2.929101000	2.476208000
1	-0.959536000	-3.627155000	1.298681000
1	-0.397648000	-1.232779000	1.723961000
1	-2.025728000	-0.770226000	1.242323000
1	2.893056000	0.122980000	-1.821204000
1	2.454619000	1.665853000	-1.116360000
1	4.866365000	1.231046000	-0.757652000
1	4.028946000	1.224803000	0.791395000
1	4.777532000	-1.243845000	-0.837317000
1	5.569803000	-0.723822000	0.646293000
1	3.900408000	-2.523637000	1.100745000
1	3.455026000	-1.021829000	1.901204000
1	1.497474000	-2.059504000	0.833055000
1	2.296536000	-2.036374000	-0.736107000

PBu3_1

15	-0.000566000	0.001305000	-0.642906000
6	-0.322303000	-1.584637000	0.217882000
6	-1.213186000	1.071207000	0.219191000
6	1.532631000	0.514424000	0.220653000
6	-2.643472000	0.884946000	-0.274277000
6	-3.633627000	1.789435000	0.447770000
6	-5.058367000	1.597090000	-0.049381000
6	0.555739000	-2.729441000	-0.273723000
6	0.267454000	-4.038865000	0.448776000
6	1.143654000	-5.177641000	-0.050559000
6	2.088643000	1.845087000	-0.273240000
6	3.367218000	2.249658000	0.449221000
6	3.916221000	3.576934000	-0.051336000
1	-0.187842000	-1.435479000	1.296820000
1	-1.374592000	-1.840086000	0.056860000
1	-1.151165000	0.878571000	1.297781000
1	-0.906933000	2.109828000	0.060441000
1	1.333972000	0.558038000	1.299145000
1	2.278887000	-0.270543000	0.061974000
1	-2.687428000	1.085681000	-1.350127000
1	-2.951998000	-0.158097000	-0.143188000
1	-3.582394000	1.589294000	1.522417000
1	-3.328134000	2.831498000	0.312332000
1	-5.385013000	0.565577000	0.100836000
1	-5.761122000	2.249640000	0.470622000
1	-5.128252000	1.813071000	-1.117871000
1	0.405118000	-2.867993000	-1.349719000
1	1.612935000	-2.475231000	-0.141216000
1	0.418282000	-3.894655000	1.522991000
1	-0.788540000	-4.293726000	0.315989000
1	2.201156000	-4.945353000	0.094682000
1	0.931967000	-6.111564000	0.471824000
1	0.986564000	-5.347991000	-1.118048000
1	2.285565000	1.780838000	-1.348864000
1	1.341497000	2.635572000	-0.143935000
1	3.166676000	2.309842000	1.523300000
1	4.115401000	1.461816000	0.317805000
1	4.141931000	3.525114000	-1.118861000
1	3.186973000	4.377206000	0.093400000
1	4.831349000	3.860383000	0.470344000

PBu3_2

15	-0.158176000	-0.115620000	-0.103043000
6	0.783827000	-1.529855000	-0.803576000
6	0.555126000	1.242318000	-1.112626000
6	-1.742422000	-0.342103000	-1.002991000
6	-0.286640000	2.519933000	-1.103188000
6	-0.656358000	3.012750000	0.294115000
6	0.558783000	3.229379000	1.184118000
6	2.299753000	-1.387207000	-0.672418000
6	2.776142000	-1.103613000	0.746713000
6	4.293084000	-1.133438000	0.850849000
6	-2.438215000	-1.669650000	-0.697772000
6	-2.641979000	-1.938541000	0.791546000
6	-3.393915000	-0.818952000	1.496439000
1	0.454800000	-2.433707000	-0.280180000
1	0.506505000	-1.646455000	-1.858213000
1	1.556871000	1.450064000	-0.723686000
1	0.679975000	0.885953000	-2.142002000
1	-2.395981000	0.493808000	-0.735070000
1	-1.546359000	-0.262723000	-2.078913000
1	0.267853000	3.308355000	-1.622082000
1	-1.200574000	2.353283000	-1.679826000
1	-1.217518000	3.945689000	0.200942000
1	-1.331420000	2.291493000	0.769564000
1	0.280086000	3.681454000	2.136792000
1	1.060903000	2.283537000	1.402901000
1	1.283524000	3.887906000	0.697752000
1	2.775299000	-2.307017000	-1.028132000
1	2.652964000	-0.587467000	-1.331612000
1	2.403225000	-0.125075000	1.069028000
1	2.334802000	-1.836983000	1.429473000
1	4.740870000	-0.397811000	0.178379000
1	4.635346000	-0.912878000	1.862685000
1	4.681732000	-2.115368000	0.571164000
1	-3.412986000	-1.676123000	-1.195878000
1	-1.860657000	-2.488364000	-1.136232000
1	-3.185882000	-2.879158000	0.907270000
1	-1.669051000	-2.081577000	1.273912000
1	-2.803317000	0.100505000	1.517605000
1	-4.334661000	-0.599876000	0.984063000
1	-3.626829000	-1.083289000	2.528695000

PBu3_2

15	-0.158157000	-0.115638000	-0.102944000
6	-1.742296000	-0.342451000	-1.003027000
6	0.784083000	-1.529759000	-0.803421000
6	0.554960000	1.242392000	-1.112566000
6	2.300002000	-1.386916000	-0.672375000
6	2.776490000	-1.103729000	0.746807000
6	4.293460000	-1.132955000	0.850717000
6	-2.437998000	-1.670027000	-0.697745000
6	-2.641953000	-1.938762000	0.791579000
6	-3.394108000	-0.819158000	1.496218000

6	-0.286962000	2.519905000	-1.103210000
6	-0.656787000	3.012764000	0.294051000
6	0.558294000	3.229685000	1.184061000
1	-2.395967000	0.493428000	-0.735287000
1	-1.546119000	-0.263168000	-2.078935000
1	0.455194000	-2.433623000	-0.279950000
1	0.506705000	-1.646468000	-1.858028000
1	1.556668000	1.450271000	-0.723604000
1	0.679892000	0.885999000	-2.141919000
1	2.775652000	-2.306533000	-1.028447000
1	2.653032000	-0.586901000	-1.331338000
1	2.403196000	-0.125492000	1.069579000
1	2.335571000	-1.837590000	1.429309000
1	4.635763000	-0.912723000	1.862609000
1	4.682489000	-2.114587000	0.570520000
1	4.740833000	-0.396818000	0.178533000
1	-3.412701000	-1.676641000	-1.195986000
1	-1.860307000	-2.488743000	-1.136025000
1	-3.185779000	-2.879418000	0.907338000
1	-1.669084000	-2.081635000	1.274114000
1	-4.334809000	-0.600254000	0.983684000
1	-3.627128000	-1.083381000	2.528478000
1	-2.803608000	0.100360000	1.517333000
1	0.267469000	3.308361000	-1.622120000
1	-1.200865000	2.353145000	-1.679865000
1	-1.218118000	3.945592000	0.200795000
1	-1.331714000	2.291416000	0.769550000
1	1.060567000	2.283954000	1.402949000
1	1.282928000	3.888298000	0.697651000
1	0.279484000	3.681800000	2.136683000

Phosphines (SMD Water)

PMe3_H2O

15	0.000144000	0.000045000	-0.595819000
6	-1.409698000	0.762036000	0.272357000
6	1.364772000	0.839604000	0.272751000
6	0.044732000	-1.601665000	0.272691000
1	-2.336698000	0.259368000	-0.006681000
1	-1.280054000	0.692047000	1.356069000
1	-1.497323000	1.812870000	-0.006824000
1	2.318495000	0.389657000	-0.006277000
1	1.393532000	1.893760000	-0.006177000
1	1.239058000	0.762193000	1.356421000
1	0.942872000	-2.154060000	-0.006752000
1	0.041444000	-1.454154000	1.356367000
1	-0.822319000	-2.202208000	-0.005664000

PPhMe2_1_H2O

15	1.644328000	-0.154079000	-0.570801000
6	2.130611000	-1.302345000	0.761862000
6	-0.137150000	-0.021416000	-0.232371000
6	-0.912250000	-1.185948000	-0.281674000
6	-2.282278000	-1.140785000	-0.066532000
6	-2.909293000	0.076089000	0.187675000
6	-2.153642000	1.240620000	0.225632000
6	-0.777545000	1.192900000	0.020017000
6	2.280406000	1.400635000	0.126438000
1	1.708102000	-2.290779000	0.582055000
1	3.217470000	-1.397438000	0.766533000
1	1.797870000	-0.939885000	1.736865000
1	-0.435860000	-2.137581000	-0.492315000
1	-2.862828000	-2.053872000	-0.100708000
1	-3.978247000	0.114071000	0.352199000
1	-2.632640000	2.191826000	0.420518000
1	-0.207059000	2.112360000	0.058426000
1	2.024405000	2.249130000	-0.507743000
1	1.902253000	1.575635000	1.136394000
1	3.368454000	1.329226000	0.163512000

PPhMe2_2_H2O

15	1.626512000	-0.650236000	-0.000018000
6	2.220857000	0.374904000	1.387022000
6	-0.151554000	-0.239242000	-0.000019000
6	-1.081686000	-1.278699000	-0.000077000
6	-2.448397000	-1.014656000	-0.000077000
6	-2.899170000	0.298765000	-0.000020000
6	-1.981391000	1.346359000	0.000037000
6	-0.619425000	1.077927000	0.000038000
6	2.220907000	0.375071000	-1.386913000
1	1.821257000	-0.001744000	2.328917000
1	3.309393000	0.311321000	1.429825000
1	1.936483000	1.422872000	1.268984000
1	-0.730594000	-2.304419000	-0.000121000
1	-3.156832000	-1.833019000	-0.000121000
1	-3.960889000	0.509038000	-0.000020000
1	-2.329662000	2.371317000	0.000081000
1	0.085001000	1.902557000	0.000084000
1	1.821339000	-0.001462000	-2.328867000
1	1.936536000	1.423026000	-1.268759000
1	3.309444000	0.311487000	-1.429685000

PPh2Me_1_H2O

15	-0.016320000	1.393785000	-0.707289000
6	1.395761000	0.312251000	-0.327051000
6	-1.399480000	0.318495000	-0.204495000
6	0.016876000	2.555133000	0.696008000
6	-1.371644000	-0.401732000	0.993292000
6	-2.451368000	-1.187735000	1.368233000
6	-3.575390000	-1.264015000	0.547826000
6	-3.613360000	-0.553826000	-0.644431000
6	-2.526962000	0.233042000	-1.018697000
6	1.448939000	-0.950227000	-0.928654000
6	2.525806000	-1.796689000	-0.711770000
6	3.579942000	-1.390300000	0.103126000
6	3.542982000	-0.135994000	0.696894000
6	2.457149000	0.710254000	0.485615000
1	0.835855000	3.265350000	0.581226000
1	-0.917647000	3.116976000	0.688482000
1	0.117109000	2.037265000	1.652370000
1	-0.495465000	-0.345947000	1.631387000
1	-2.420239000	-1.742402000	2.297259000
1	-4.416895000	-1.878878000	0.839819000
1	-4.483553000	-0.612593000	-1.285290000
1	-2.553874000	0.784883000	-1.951249000
1	0.632647000	-1.272275000	-1.567097000
1	2.545636000	-2.773105000	-1.178690000
1	4.422070000	-2.048640000	0.271982000
1	4.357506000	0.188006000	1.332161000
1	2.446155000	1.682644000	0.961595000

PPh3_1_H2O

15	0.013885000	-0.001529000	-1.245028000
6	1.518882000	-0.618259000	-0.432416000
6	-0.215304000	1.617069000	-0.449025000
6	-1.281741000	-1.000308000	-0.450804000
6	-2.568371000	-0.906714000	-0.989283000
6	-3.626802000	-1.603058000	-0.421789000
6	-3.407656000	-2.420197000	0.683110000
6	-2.129591000	-2.530584000	1.217469000
6	-1.071979000	-1.821211000	0.657164000
6	0.470107000	2.698876000	-1.008532000
6	0.384371000	3.963021000	-0.440439000
6	-0.405485000	4.165115000	0.686939000
6	-1.100799000	3.098124000	1.243849000
6	-1.003643000	1.829298000	0.682257000
6	2.115191000	-0.018272000	0.676492000
6	3.253230000	-0.575866000	1.250813000
6	3.800902000	-1.741893000	0.729690000
6	3.212699000	-2.347858000	-0.376388000
6	2.085781000	-1.783044000	-0.957838000
1	-2.738504000	-0.280341000	-1.858592000
1	-4.618875000	-1.516040000	-0.845571000
1	-4.229143000	-2.971090000	1.122230000
1	-1.953647000	-3.165901000	2.076005000
1	-0.082563000	-1.907460000	1.090438000
1	1.075108000	2.545371000	-1.895726000
1	0.924282000	4.790626000	-0.881901000
1	-0.482161000	5.151240000	1.126095000
1	-1.717458000	3.251122000	2.120193000
1	-1.542124000	1.003440000	1.131956000
1	1.692010000	0.885228000	1.099182000
1	3.708397000	-0.099739000	2.109765000
1	4.684734000	-2.174852000	1.179758000
1	3.637031000	-3.253424000	-0.790204000
1	1.635604000	-2.249212000	-1.827850000

Pani3_1_H2O

15	0.054887000	0.001461000	1.495063000
6	-0.303640000	-1.597221000	0.707646000
6	1.594487000	0.497140000	0.672934000
6	-1.178837000	1.082399000	0.714765000
6	-1.463220000	2.296177000	1.336907000
6	-2.361586000	3.207077000	0.790269000
6	-3.007586000	2.890886000	-0.402302000
6	-2.746392000	1.672906000	-1.033940000
6	-1.841808000	0.784675000	-0.481705000
6	2.796708000	0.210101000	1.314904000
6	4.026380000	0.499321000	0.730691000
6	4.056144000	1.106451000	-0.521956000
6	2.860269000	1.414122000	-1.175685000
6	1.648348000	1.109058000	-0.584785000
6	0.481034000	-2.183139000	-0.289006000
6	0.139057000	-3.409278000	-0.836068000
6	-1.006118000	-4.076190000	-0.403283000
6	-1.803620000	-3.509568000	0.590203000
6	-1.437537000	-2.286453000	1.138262000
8	5.200808000	1.437371000	-1.174171000
8	-3.906821000	3.705831000	-1.013305000

8	-1.270991000	-5.272274000	-0.993903000
6	6.431832000	1.131811000	-0.539702000
6	-4.183775000	4.955341000	-0.402505000
6	-2.426863000	-5.974325000	-0.567964000
1	-0.972992000	2.542513000	2.272666000
1	-2.555947000	4.139390000	1.300589000
1	-3.260574000	1.444596000	-1.958872000
1	-1.645029000	-0.152246000	-0.990425000
1	2.779395000	-0.253334000	2.295279000
1	4.939012000	0.260496000	1.257423000
1	2.905032000	1.889422000	-2.147277000
1	0.731224000	1.347030000	-1.111167000
1	1.372218000	-1.681248000	-0.646448000
1	0.748848000	-3.864939000	-1.605838000
1	-2.692958000	-4.010282000	0.945239000
1	-2.058077000	-1.857978000	1.918767000
1	7.216594000	1.463071000	-1.213425000
1	6.522809000	1.659366000	0.411544000
1	6.527731000	0.057756000	-0.369754000
1	-4.907929000	5.455838000	-1.038634000
1	-4.608832000	4.818224000	0.593556000
1	-3.280352000	5.563898000	-0.331222000
1	-2.366096000	-6.220617000	0.493796000
1	-3.330312000	-5.390430000	-0.753907000
1	-2.463052000	-6.890666000	-1.149893000

Pani3_2_H2O

15	0.100207000	-0.057388000	1.538295000
6	-0.666649000	1.443497000	0.858701000
6	-0.814825000	-1.377700000	0.696425000
6	1.702692000	-0.061512000	0.680724000
6	2.733538000	-0.817863000	1.234374000
6	3.984835000	-0.904838000	0.631903000
6	4.219954000	-0.202891000	-0.547574000
6	3.202566000	0.569111000	-1.113290000
6	1.962227000	0.634659000	-0.506201000
6	-1.807332000	-2.044876000	1.408920000
6	-2.589623000	-3.036074000	0.822092000
6	-2.364655000	-3.377890000	-0.508219000
6	-1.366705000	-2.724622000	-1.237635000
6	-0.607143000	-1.736969000	-0.640994000
6	-1.762506000	1.453377000	0.004102000
6	-2.310968000	2.648066000	-0.463919000
6	-1.747154000	3.858096000	-0.077071000
6	-0.646197000	3.867463000	0.785193000
6	-0.125456000	2.677042000	1.249996000
8	-3.063852000	-4.334750000	-1.171218000
8	5.407874000	-0.208584000	-1.206802000
8	-2.202952000	5.073429000	-0.483475000
6	-4.083690000	-5.016998000	-0.459826000
6	6.463166000	-0.978436000	-0.655104000
6	-3.319077000	5.096289000	-1.357264000
1	2.561293000	-1.354153000	2.161276000
1	4.759444000	-1.502368000	1.090702000
1	3.405871000	1.108270000	-2.029789000
1	1.182921000	1.234799000	-0.961985000
1	-1.979334000	-1.788531000	2.448416000
1	-3.351981000	-3.532196000	1.405207000
1	-1.207099000	-3.007125000	-2.270411000
1	0.157394000	-1.235192000	-1.223123000
1	-2.211487000	0.520421000	-0.315033000
1	-3.164749000	2.616782000	-1.125732000
1	-0.222168000	4.818379000	1.081446000
1	0.724222000	2.697997000	1.924793000
1	-4.520177000	-5.729177000	-1.153906000
1	-3.671093000	-5.551201000	0.398017000
1	-4.853823000	-4.322452000	-0.119053000
1	7.314276000	-0.855456000	-1.318444000
1	6.725646000	-0.623020000	0.343074000
1	6.191640000	-2.034484000	-0.604639000
1	-3.095270000	4.576782000	-2.291103000
1	-4.192310000	4.638670000	-0.888333000
1	-3.526882000	6.141733000	-1.565838000

Pani3_3_H2O

15	0.001259000	0.005077000	-1.739006000
6	-1.154212000	-1.144854000	-0.940655000
6	-0.419443000	1.583784000	-0.948275000
6	1.574149000	-0.419122000	-0.937801000
6	2.744997000	0.125875000	-1.481324000
6	3.979766000	-0.118341000	-0.912046000
6	4.077229000	-0.939027000	0.214652000
6	2.928436000	-1.499884000	0.764909000
6	1.689932000	-1.230479000	0.186542000
6	-1.467615000	2.327100000	-1.506704000
6	-1.880281000	3.518472000	-0.942218000
6	-1.234902000	4.010645000	0.195127000
6	-0.181191000	3.296294000	0.758066000
6	0.211552000	2.089098000	0.184268000
6	-1.919435000	-0.840227000	0.180606000
6	-2.763986000	-1.783124000	0.762643000
6	-2.838601000	-3.062528000	0.219912000
6	-2.078005000	-3.386004000	-0.906780000
6	-1.257328000	-2.433995000	-1.479820000
8	-1.693732000	5.193313000	0.682610000
8	5.328379000	-1.143428000	0.704438000
8	-3.627554000	-4.050839000	0.717723000
6	-1.060798000	5.718952000	1.837487000
6	5.461941000	-1.989098000	1.834853000
6	-4.404321000	-3.755900000	1.866743000
1	2.682689000	0.752848000	-2.364414000
1	4.883822000	0.304998000	-1.330540000
1	2.981980000	-2.138926000	1.634683000
1	0.804007000	-1.667522000	0.632484000
1	-1.967515000	1.960362000	-2.396899000
1	-2.692968000	4.090513000	-1.371141000
1	0.334143000	3.662014000	1.634744000
1	1.026408000	1.539257000	0.640813000
1	-1.864173000	0.148120000	0.621952000
1	-3.347432000	-1.511489000	1.630683000
1	-2.153921000	-4.383552000	-1.320048000
1	-0.679598000	-2.691933000	-2.361072000
1	-1.567764000	6.650957000	2.069853000
1	-1.155672000	5.033238000	2.681538000
1	-0.004136000	5.917438000	1.648162000
1	6.522732000	-2.032910000	2.063722000
1	4.921863000	-1.584096000	2.692719000
1	5.095202000	-2.993866000	1.616516000
1	-3.766777000	-3.480241000	2.708935000
1	-5.109045000	-2.946947000	1.665263000
1	-4.952837000	-4.661368000	2.109337000

Ppfp3_1_H2O

15	-0.001524000	0.001010000	1.429522000
6	0.525539000	-1.543281000	0.628411000
6	-0.094633000	-2.096971000	-0.491707000
6	1.637168000	-2.191753000	1.174316000
6	0.382441000	-3.271271000	-1.062166000
1	-0.957807000	-1.613633000	-0.932530000
6	2.136812000	-3.356227000	0.610163000
1	2.120463000	-1.778485000	2.052559000
6	1.491566000	-3.871557000	-0.499210000
1	-0.089725000	-3.713665000	-1.928818000
1	2.998605000	-3.864804000	1.020159000
6	1.073909000	1.227722000	0.627763000
6	1.094283000	2.510855000	1.181824000
6	1.849739000	0.969152000	-0.502481000
6	1.854707000	3.524049000	0.616541000
1	0.505169000	2.721200000	2.067384000
6	2.629400000	1.967424000	-1.074434000
1	1.849738000	-0.017267000	-0.949899000
6	2.610011000	3.224566000	-0.502473000
1	1.876047000	4.521711000	1.033266000
1	3.238117000	1.781389000	-1.948838000
6	-1.601828000	0.317629000	0.627675000
6	-1.765809000	1.111956000	-0.507560000
6	-2.723635000	-0.301195000	1.186664000
6	-3.020441000	1.285995000	-1.079384000
1	-0.911497000	1.601351000	-0.958909000
6	-3.981637000	-0.151334000	0.621505000
1	-2.611296000	-0.911027000	2.076093000
6	-4.099790000	0.645533000	-0.502469000
1	-3.163809000	1.901035000	-1.957373000
1	-4.856578000	-0.627877000	1.042026000
9	3.360911000	4.196296000	-1.052449000
9	1.960299000	-5.007324000	-1.047865000
9	-5.316919000	0.808491000	-1.052555000

PCy3_1_H2O

15	0.109153000	0.379639000	-0.638462000
6	-1.684374000	0.154220000	-0.286071000
6	0.673614000	1.411936000	0.790973000
6	0.827213000	-1.293813000	-0.272740000
6	-0.183634000	2.661709000	1.007220000
6	-0.047382000	3.660892000	-0.134899000
6	1.411194000	4.076580000	-0.290045000
6	2.299811000	2.861241000	-0.527481000
6	2.136338000	1.829504000	0.585372000
6	-2.038360000	-0.437755000	1.074969000
6	-3.550298000	-0.469253000	1.274978000
6	-4.219093000	-1.274971000	0.168457000
6	-3.865327000	-0.705544000	-1.199658000
6	-2.353464000	-0.658768000	-1.396080000
6	1.265972000	-1.584556000	1.162790000
6	1.736506000	-3.029123000	1.302638000
6	2.889691000	-3.315785000	0.350392000
6	2.475038000	-3.026846000	-1.085919000
6	1.983474000	-1.591921000	-1.233187000
1	-2.101881000	1.169811000	-0.341552000
1	0.607967000	0.801545000	1.694801000

1	0.013853000	-1.992809000	-0.518265000
1	-1.228889000	2.392534000	1.174378000
1	0.166803000	3.144869000	1.927736000
1	-0.400131000	3.207422000	-1.068503000
1	-0.675218000	4.534066000	0.053602000
1	1.524176000	4.795472000	-1.104107000
1	1.730766000	4.579187000	0.630696000
1	2.033007000	2.407357000	-1.486954000
1	3.346948000	3.162972000	-0.598096000
1	2.500544000	2.263763000	1.524101000
1	2.757371000	0.952677000	0.378301000
1	-1.655002000	-1.464725000	1.118426000
1	-1.554973000	0.122822000	1.879826000
1	-3.795779000	-0.885021000	2.254596000
1	-3.933054000	0.557891000	1.254228000
1	-3.868625000	-2.312180000	0.224204000
1	-5.302057000	-1.293769000	0.306741000
1	-4.331806000	-1.293971000	-1.992584000
1	-4.263064000	0.312780000	-1.276288000
1	-2.105677000	-0.235408000	-2.373169000
1	-1.961052000	-1.682976000	-1.380297000
1	2.096681000	-0.919489000	1.426135000
1	0.458330000	-1.380334000	1.867890000
1	2.031806000	-3.229203000	2.334958000
1	0.902308000	-3.701055000	1.069066000
1	3.737008000	-2.672362000	0.614637000
1	3.226472000	-4.349444000	0.452728000
1	3.302787000	-3.216403000	-1.772686000
1	1.661863000	-3.707146000	-1.364721000
1	1.669828000	-1.399390000	-2.262157000
1	2.808202000	-0.899826000	-1.018182000

PCy3_2_H2O

15	0.023606000	-0.129162000	-1.046490000
6	-0.257727000	1.545613000	-0.296909000
6	1.414903000	-0.829549000	-0.066347000
6	-1.436474000	-1.089565000	-0.464361000
6	2.722828000	-0.195904000	-0.547496000
6	3.917905000	-0.739599000	0.225195000
6	3.997218000	-2.253365000	0.075780000
6	2.702367000	-2.903713000	0.544327000
6	1.496277000	-2.349111000	-0.207632000
6	0.236935000	2.649085000	-1.237404000
6	-0.144427000	4.031303000	-0.720220000
6	0.390495000	4.252766000	0.688157000
6	-0.109467000	3.160271000	1.623821000
6	0.294485000	1.781860000	1.109711000
6	-2.696903000	-0.663180000	-1.219916000
6	-3.873335000	-1.566565000	-0.864654000
6	-4.123833000	-1.566654000	0.638099000
6	-2.868780000	-1.988106000	1.392018000
6	-1.699529000	-1.074375000	1.038355000
1	-1.352456000	1.635640000	-0.239981000
1	1.284841000	-0.597683000	0.997424000
1	-1.223110000	-2.124107000	-0.765962000
1	2.847304000	-0.430095000	-1.612529000
1	2.672680000	0.893566000	-0.468388000
1	4.839952000	-0.269125000	-0.122407000
1	3.805014000	-0.486675000	1.285776000
1	4.158770000	-2.497168000	-0.980593000
1	4.848029000	-2.652255000	0.631844000
1	2.751605000	-3.988119000	0.425291000
1	2.571145000	-2.703429000	1.613920000
1	0.583850000	-2.817099000	0.168752000
1	1.576252000	-2.605438000	-1.272403000
1	1.329067000	2.579760000	-1.320996000
1	-0.167363000	2.491008000	-2.240329000
1	0.222813000	4.804213000	-1.398779000
1	-1.237658000	4.110552000	-0.701805000
1	1.486123000	4.225346000	0.663176000
1	0.101597000	5.238769000	1.057830000
1	0.277543000	3.312448000	2.633746000
1	-1.202856000	3.212483000	1.685226000
1	-0.045267000	1.006349000	1.798134000
1	1.388964000	1.726446000	1.082225000
1	-2.948907000	0.369639000	-0.950542000
1	-2.512373000	-0.677782000	-2.297479000
1	-4.769165000	-1.250337000	-1.403184000
1	-3.645041000	-2.588285000	-1.188623000
1	-4.403605000	-0.554504000	0.952801000
1	-4.960318000	-2.223056000	0.886493000
1	-3.046607000	-1.978107000	2.469527000
1	-2.612149000	-3.017530000	1.116021000
1	-0.801243000	-1.365631000	1.590417000
1	-1.949158000	-0.049636000	1.339616000

PCy3_3_H2O

15	0.093105000	0.062372000	-0.934209000
6	-0.810830000	-1.455363000	-0.403154000
6	-0.689022000	1.414265000	0.051460000
6	1.719066000	-0.082641000	-0.046300000
6	-2.120456000	1.670332000	-0.418987000
6	-2.774585000	2.808609000	0.356000000
6	-1.953552000	4.084494000	0.237999000
6	-0.535943000	3.839933000	0.734308000
6	0.128320000	2.704555000	-0.037490000
6	-1.995509000	-1.817374000	-1.303003000
6	-2.522919000	-3.202023000	-0.939169000
6	-2.918918000	-3.265354000	0.531360000
6	-1.744706000	-2.888226000	1.427094000
6	-1.223452000	-1.501858000	1.065679000
6	2.785151000	0.657261000	-0.864611000
6	4.126154000	0.687202000	-0.143970000
6	4.605440000	-0.731880000	0.128238000
6	3.557364000	-1.501760000	0.919787000
6	2.205446000	-1.508997000	0.210899000
1	-0.071010000	-2.250401000	-0.567925000
1	-0.710921000	1.104599000	1.105144000
1	1.612657000	0.415429000	0.928499000
1	-2.097671000	1.925023000	-1.486587000
1	-2.719288000	0.763634000	-0.319595000
1	-3.793511000	2.968301000	-0.002702000
1	-2.845089000	2.525553000	1.412635000
1	-1.917790000	4.391455000	-0.813680000
1	-2.421890000	4.897985000	0.795696000
1	0.065383000	4.747733000	0.651943000
1	-0.572099000	3.572902000	1.796672000
1	1.136680000	2.543472000	0.349057000
1	0.228270000	2.991216000	-1.092841000
1	-2.802042000	-1.087801000	-1.181425000
1	-1.690659000	-1.785896000	-2.352214000
1	-3.370771000	-3.464688000	-1.575380000
1	-1.735261000	-3.940532000	-1.128202000
1	-3.739621000	-2.560610000	0.709373000
1	-3.290070000	-4.260587000	0.784350000
1	-2.037781000	-2.924660000	2.478509000
1	-0.937137000	-3.617441000	1.289815000
1	-0.385110000	-1.219040000	1.710048000
1	-2.019398000	-0.767309000	1.236973000
1	2.901474000	0.126666000	-1.818175000
1	2.453751000	1.668191000	-1.112064000
1	4.862030000	1.236923000	-0.734533000
1	4.010612000	1.219993000	0.807145000
1	4.775015000	-1.238034000	-0.829002000
1	5.557947000	-0.724583000	0.662007000
1	3.888128000	-2.527029000	1.098990000
1	3.434206000	-1.028190000	1.900600000
1	1.487176000	-2.066318000	0.815330000
1	2.297215000	-2.034972000	-0.748142000

PBu3_1_H2O

15	0.000485000	-0.002081000	-0.670930000
6	0.931336000	-1.321201000	0.193823000
6	-1.608430000	-0.146667000	0.192011000
6	0.678121000	1.464337000	0.192126000
6	-2.445266000	-1.330637000	-0.276362000
6	-3.775981000	-1.428267000	0.456931000
6	-4.607939000	-2.608424000	-0.019255000
6	2.375910000	-1.450597000	-0.273190000
6	3.127317000	-2.554208000	0.458570000
6	4.566534000	-2.679426000	-0.015460000
6	0.066896000	2.780045000	-0.273526000
6	0.651599000	3.982533000	0.454684000
6	0.040209000	5.292014000	-0.017730000
1	0.894551000	-1.124572000	1.272720000
1	0.407979000	-2.266541000	0.020351000
1	-1.420896000	-0.213927000	1.271071000
1	-2.163114000	0.780671000	0.018469000
1	0.528498000	1.334064000	1.271329000
1	1.758126000	1.484101000	0.016447000
1	-2.631181000	-1.244731000	-1.352337000
1	-1.890574000	-2.264011000	-0.132720000
1	-3.585827000	-1.513364000	1.530943000
1	-4.330556000	-0.496124000	0.313262000
1	-4.071929000	-3.547093000	0.137080000
1	-5.559602000	-2.674471000	0.509135000
1	-4.822152000	-2.524351000	-1.086890000
1	2.396152000	-1.652355000	-1.349568000
1	2.904774000	-0.502743000	-0.127011000
1	3.103527000	-2.350046000	1.533118000
1	2.600206000	-3.501597000	0.311329000
1	5.109012000	-1.745342000	0.145864000
1	5.100264000	-3.471744000	0.510449000
1	4.603252000	-2.902294000	-1.084008000
1	0.226986000	2.897329000	-1.350707000
1	-1.017698000	2.765427000	-0.122366000
1	0.491006000	3.860887000	1.530007000
1	1.734984000	3.998094000	0.302742000
1	0.211498000	5.434890000	-1.086862000

1	-1.039404000	5.296045000	0.147338000
1	0.462215000	6.150081000	0.506533000

PBu3_2_H2O

15	-0.158642000	-0.122393000	-0.096939000
6	0.772425000	-1.549700000	-0.781876000
6	0.571157000	1.221995000	-1.110750000
6	-1.739852000	-0.336791000	-1.002689000
6	-0.264613000	2.502975000	-1.113571000
6	-0.641392000	3.000353000	0.279604000
6	0.570703000	3.210632000	1.174937000
6	2.288631000	-1.406254000	-0.661120000
6	2.771847000	-1.089028000	0.748026000
6	4.288727000	-1.123699000	0.843750000
6	-2.448038000	-1.656145000	-0.694163000
6	-2.662379000	-1.910941000	0.795566000
6	-3.400496000	-0.773722000	1.486142000
1	0.443157000	-2.445073000	-0.244608000
1	0.488070000	-1.677517000	-1.832914000
1	1.572356000	1.425714000	-0.718871000
1	0.696482000	0.855012000	-2.135913000
1	-2.386390000	0.506985000	-0.743213000
1	-1.533603000	-0.265106000	-2.076884000
1	0.297425000	3.287366000	-1.629765000
1	-1.174949000	2.337885000	-1.695829000
1	-1.195108000	3.936886000	0.181935000
1	-1.325640000	2.284403000	0.750452000
1	0.290633000	3.661118000	2.127678000
1	1.071850000	2.263466000	1.391042000
1	1.297850000	3.868107000	0.691489000
1	2.762177000	-2.334453000	-0.996245000
1	2.639221000	-0.621969000	-1.339607000
1	2.406556000	-0.099662000	1.045702000
1	2.331044000	-1.803338000	1.450887000
1	4.734123000	-0.408212000	0.148782000
1	4.639152000	-0.878548000	1.846793000
1	4.669502000	-2.114509000	0.586614000
1	-3.419924000	-1.657654000	-1.197208000
1	-1.874092000	-2.482316000	-1.122784000
1	-3.221474000	-2.841519000	0.916648000
1	-1.693629000	-2.066640000	1.282404000
1	-2.798992000	0.138999000	1.497201000
1	-4.335694000	-0.548371000	0.967041000
1	-3.640789000	-1.023221000	2.520122000

PBu3_3_H2O

15	-0.285449000	0.284894000	0.325086000
6	-1.705386000	0.245960000	-0.839953000
6	0.390896000	-1.388955000	0.020441000
6	0.880407000	1.279880000	-0.688492000
6	-0.586431000	-2.488556000	0.427180000
6	0.052542000	-3.873583000	0.458627000
6	0.609542000	-4.296647000	-0.893011000
6	-2.232489000	1.631678000	-1.214666000
6	-2.574771000	2.514981000	-0.018039000
6	-3.582558000	1.869503000	0.921027000
6	2.346171000	1.111567000	-0.295485000
6	2.628319000	1.392136000	1.174128000
6	4.117853000	1.365439000	1.477180000
1	-1.386848000	-0.287003000	-1.743663000
1	-2.500358000	-0.347491000	-0.380597000
1	0.656572000	-1.472933000	-1.039788000
1	1.316630000	-1.490478000	0.598320000
1	0.738336000	1.002031000	-1.739432000
1	0.591187000	2.331106000	-0.589281000
1	-1.001469000	-2.256411000	1.413708000
1	-1.429835000	-2.499932000	-0.270680000
1	0.853295000	-3.879184000	1.204322000
1	-0.691025000	-4.601701000	0.791434000
1	1.441145000	-3.659595000	-1.198307000
1	0.972417000	-5.324804000	-0.870160000
1	-0.161213000	-4.228870000	-1.665061000
1	-1.493703000	2.140964000	-1.838937000
1	-3.128807000	1.509009000	-1.830555000
1	-1.659286000	2.753454000	0.534427000
1	-2.969078000	3.465366000	-0.384842000
1	-4.474147000	1.554665000	0.372709000
1	-3.161837000	0.986080000	1.406272000
1	-3.895912000	2.559075000	1.705540000
1	2.678032000	0.095714000	-0.533316000
1	2.957033000	1.783388000	-0.906794000
1	2.204303000	2.365073000	1.442796000
1	2.112620000	0.650596000	1.792580000
1	4.644231000	2.125055000	0.895163000
1	4.322178000	1.549105000	2.532423000
1	4.545755000	0.394708000	1.216814000

POct3_1_H2O

15	-2.376038000	0.968416000	-0.637404000
6	-1.995936000	0.888337000	-2.426510000
6	-2.306079000	2.779709000	-0.351046000
6	-4.188438000	0.700226000	-0.641076000
6	-2.174593000	3.150046000	1.129774000
6	-1.070835000	2.385473000	1.862897000
6	0.287526000	2.427045000	1.175793000
6	-0.502362000	1.063379000	-2.703697000
6	0.375779000	-0.008491000	-2.068807000
6	1.849252000	0.185839000	-2.391022000
6	-4.598432000	-0.701718000	-1.101277000
6	-3.787706000	-1.829372000	-0.467547000
6	-3.810474000	-1.810597000	1.054500000
6	2.743687000	-0.807472000	-1.665644000
6	4.222452000	-0.468982000	-1.759086000
6	5.109433000	-1.496930000	-1.071665000
6	6.561239000	-1.050782000	-1.004757000
6	-3.204151000	-3.067493000	1.676191000
6	-1.787767000	-3.379504000	1.199879000
6	-0.784635000	-2.262615000	1.457458000
6	0.613193000	-2.645704000	0.995239000
6	1.290933000	1.485145000	1.825530000
6	2.677982000	1.585575000	1.205047000
6	3.673564000	0.565723000	1.747828000
6	3.973414000	0.755316000	3.228494000
1	-2.585811000	1.636290000	-2.967938000
1	-2.316317000	-0.097724000	-2.780074000
1	-1.439964000	3.154027000	-0.903071000
1	-3.187636000	3.256481000	-0.791552000
1	-4.525491000	0.875421000	0.386110000
1	-4.670123000	1.457527000	-1.268595000
1	-1.987120000	4.225061000	1.208471000
1	-3.126148000	2.964167000	1.636517000
1	-0.970993000	2.781871000	2.877572000
1	-1.369631000	1.336140000	1.970620000
1	0.188336000	2.141595000	0.121030000
1	0.675995000	3.452174000	1.182407000
1	-0.332259000	1.066063000	-3.784724000
1	-0.169909000	2.043352000	-2.340898000
1	0.256573000	0.010405000	-0.977542000
1	0.043098000	-1.002534000	-2.394687000
1	2.011486000	0.117218000	-3.472022000
1	2.137790000	1.204291000	-2.100392000
1	-5.658585000	-0.851393000	-0.875725000
1	-4.500960000	-0.761834000	-2.188465000
1	-4.171824000	-2.792291000	-0.820801000
1	-2.749060000	-1.766508000	-0.816990000
1	-3.276897000	-0.923501000	1.413869000
1	-4.846214000	-1.703911000	1.394609000
1	2.459127000	-0.829334000	-0.606555000
1	2.565831000	-1.818183000	-2.051379000
1	4.520406000	-0.372244000	-2.809274000
1	4.387781000	0.514373000	-1.299688000
1	4.729482000	-1.673505000	-0.059752000
1	5.031651000	-2.451091000	-1.601096000
1	6.649337000	-0.122470000	-0.434272000
1	7.197753000	-1.797958000	-0.529462000
1	6.955829000	-0.861490000	-2.005615000
1	-3.198364000	-2.963805000	2.765659000
1	-3.850122000	-3.920540000	1.446111000
1	-1.436355000	-4.289151000	1.697433000
1	-1.797326000	-3.604939000	0.127804000
1	-1.101557000	-1.352339000	0.936984000
1	-0.777283000	-2.022286000	2.526490000
1	0.946571000	-3.565132000	1.482524000
1	0.626743000	-2.819031000	-0.085000000
1	1.345004000	-1.865476000	1.215527000
1	1.339327000	1.689468000	2.899969000
1	0.921726000	0.453737000	1.720007000
1	2.583222000	1.460911000	0.120071000
1	3.071490000	2.595688000	1.368151000
1	3.279349000	-0.442937000	1.578993000
1	4.605263000	0.631626000	1.176582000
1	4.319049000	1.773650000	3.423221000
1	3.087606000	0.583133000	3.841634000
1	4.748415000	0.067776000	3.569283000

POct3_2_H2O

15	-1.560797000	-1.553524000	0.442455000
6	-1.036967000	-2.855267000	-0.744704000
6	-3.380556000	-1.765924000	0.407771000
6	-1.207218000	-2.375623000	2.045043000
6	-4.108255000	-0.684957000	1.211257000
6	-3.790632000	0.744128000	0.769277000
6	-4.017144000	0.986094000	-0.717757000
6	0.345824000	-2.621979000	-1.358191000
6	0.435831000	-1.297564000	-2.111071000
6	1.626987000	-1.215480000	-3.061427000
6	0.286076000	-2.548307000	2.323591000
6	1.085977000	-1.259830000	2.159952000
6	0.578404000	-0.120231000	3.032875000
6	2.978879000	-1.408880000	-2.377887000

6	3.202738000	-0.430846000	-1.235469000
6	4.601300000	-0.478625000	-0.641539000
6	4.735446000	0.470503000	0.540381000
6	1.429676000	1.138911000	2.926629000
6	1.357148000	1.821089000	1.566036000
6	2.179504000	3.102083000	1.530219000
6	2.216301000	3.742099000	0.151939000
6	-3.751362000	2.437734000	-1.150340000
6	-2.533193000	2.573187000	-2.059559000
6	-1.227767000	2.204741000	-1.368265000
6	-0.039517000	2.237681000	-2.317623000
1	-1.778322000	-2.849473000	-1.551137000
1	-1.095245000	-3.835860000	-0.259880000
1	-3.690546000	-1.727162000	-0.639675000
1	-3.643762000	-2.761353000	0.780932000
1	-1.674474000	-1.769964000	2.827564000
1	-1.707515000	-3.350065000	2.050526000
1	-5.186710000	-0.849466000	1.123379000
1	-3.863460000	-0.795461000	2.272072000
1	-4.415293000	1.437770000	1.339867000
1	-2.750991000	0.994187000	1.015198000
1	-3.362566000	0.323301000	-1.296437000
1	-5.041310000	0.690056000	-0.965706000
1	1.116114000	-2.655369000	-0.579802000
1	0.570788000	-3.441250000	-2.050042000
1	-0.491025000	-1.147191000	-2.679299000
1	0.478392000	-0.473985000	-1.390614000
1	1.507020000	-1.966336000	-3.848489000
1	1.620479000	-0.237025000	-3.554132000
1	0.418021000	-2.932223000	3.339836000
1	0.687667000	-3.309144000	1.647185000
1	2.140648000	-1.447716000	2.390816000
1	1.047376000	-0.954756000	1.109067000
1	-0.451961000	0.128239000	2.750210000
1	0.542625000	-0.462017000	4.072792000
1	3.061748000	-2.431801000	-1.994601000
1	3.776122000	-1.291367000	-3.118406000
1	2.988253000	0.591213000	-1.578533000
1	2.484121000	-0.639030000	-0.437122000
1	4.822618000	-1.502851000	-0.325366000
1	5.335221000	-0.225741000	-1.412505000
1	5.738753000	0.457026000	0.967796000
1	4.512387000	1.496694000	0.234807000
1	4.027641000	0.202401000	1.331263000
1	1.116860000	1.859376000	3.689058000
1	2.475766000	0.890776000	3.145248000
1	1.714581000	1.147549000	0.776979000
1	0.308887000	2.043913000	1.326653000
1	1.774549000	3.807867000	2.261792000
1	3.199527000	2.872006000	1.857149000
1	2.798816000	4.664305000	0.148537000
1	2.662736000	3.059443000	-0.577056000
1	1.207213000	3.979905000	-0.194786000
1	-4.626503000	2.843929000	-1.661075000
1	-3.599043000	3.059219000	-0.260205000
1	-2.667805000	1.923472000	-2.932708000
1	-2.459698000	3.596747000	-2.440603000
1	-1.062124000	2.891247000	-0.530081000
1	-1.313995000	1.203401000	-0.932554000
1	0.881278000	1.912980000	-1.826828000
1	-0.213046000	1.572802000	-3.168474000
1	0.124419000	3.244648000	-2.709455000

POct3_3_H2O

15	0.792347000	-2.160503000	-0.028433000
6	0.888913000	-1.842967000	1.783533000
6	2.303597000	-3.179544000	-0.257559000
6	-0.464825000	-3.496158000	-0.040591000
6	3.584015000	-2.451509000	0.144734000
6	3.804719000	-1.127382000	-0.573163000
6	5.081981000	-0.435791000	-0.124470000
6	1.401847000	-0.453560000	2.171842000
6	0.638392000	0.691473000	1.510826000
6	-0.861671000	0.684381000	1.777732000
6	-1.881366000	-3.006312000	0.253967000
6	-2.399789000	-1.947234000	-0.709214000
6	-3.809181000	-1.501753000	-0.355660000
6	-1.578533000	1.795411000	1.023529000
6	-3.030915000	1.982753000	1.434898000
6	-3.724388000	3.077883000	0.637780000
6	-5.144030000	3.345550000	1.113207000
6	-4.323607000	-0.371653000	-1.233913000
6	-5.724243000	0.070933000	-0.834366000
6	-6.270159000	1.237905000	-1.650278000
6	-6.479719000	0.889593000	-3.117665000
6	5.313371000	0.898668000	-0.816271000
6	6.590076000	1.590174000	-0.364444000
6	6.820795000	2.927353000	-1.052569000
6	8.103413000	3.600569000	-0.590101000
1	1.507247000	-2.623540000	2.240138000
1	-0.119494000	-1.978835000	2.181698000
1	2.195584000	-4.096323000	0.332665000
1	2.351351000	-3.471312000	-1.311053000
1	-0.429400000	-3.956541000	-1.033377000
1	-0.170594000	-4.265147000	0.682407000
1	3.571362000	-2.269088000	1.225008000
1	4.442657000	-3.103757000	-0.042126000
1	3.830255000	-1.294816000	-1.655980000
1	2.956136000	-0.457598000	-0.386887000
1	5.038100000	-0.278405000	0.960368000
1	5.938548000	-1.094770000	-0.306462000
1	1.334000000	-0.347812000	3.259298000
1	2.463204000	-0.365622000	1.918355000
1	1.050119000	1.643713000	1.858616000
1	0.804118000	0.660582000	0.428200000
1	-1.298773000	-0.278315000	1.485238000
1	-1.039600000	0.791054000	2.854425000
1	-2.564716000	-3.861171000	0.240839000
1	-1.927441000	-2.600752000	1.271009000
1	-1.739330000	-1.072244000	-0.690707000
1	-2.372087000	-2.332438000	-1.734887000
1	-4.493558000	-2.355319000	-0.421370000
1	-3.823446000	-1.176634000	0.692836000
1	-1.043287000	2.740098000	1.175655000
1	-1.529535000	1.584420000	-0.053113000
1	-3.578000000	1.039803000	1.308502000
1	-3.075416000	2.225558000	2.502780000
1	-3.133282000	3.996592000	0.707069000
1	-3.729074000	2.795102000	-0.420438000
1	-5.641505000	4.099254000	0.500926000
1	-5.747226000	2.435396000	1.077465000
1	-5.143786000	3.699736000	2.146438000
1	-3.638489000	0.484204000	-1.161173000
1	-4.309479000	-0.694114000	-2.279765000
1	-6.407976000	-0.781220000	-0.927516000
1	-5.708958000	0.340001000	0.227738000
1	-7.218951000	1.564437000	-1.216238000
1	-5.584764000	2.087710000	-1.568210000
1	-6.947103000	1.712417000	-3.659911000
1	-5.535480000	0.662112000	-3.614528000
1	-7.125715000	0.013579000	-3.215877000
1	5.349099000	0.743909000	-1.900549000
1	4.457659000	1.556877000	-0.626630000
1	6.556157000	1.743331000	0.720345000
1	7.446233000	0.933116000	-0.555885000
1	6.850993000	2.771681000	-2.135287000
1	5.965441000	3.581617000	-0.858393000
1	8.262743000	4.558726000	-1.086037000
1	8.078496000	3.780840000	0.486869000
1	8.968675000	2.967445000	-0.798557000

Protonated Phosphines

PMe3H

15	0.000184000	0.000411000	0.344615000
6	-1.360097000	0.988221000	-0.215965000
6	-0.176640000	-1.671446000	-0.215878000
6	1.536487000	0.682674000	-0.216165000
1	-1.246127000	2.006589000	0.151440000
1	-1.359492000	0.988531000	-1.305742000
1	-2.293208000	0.563851000	0.150663000
1	0.656687000	-2.268111000	0.151163000
1	-1.116021000	-2.080439000	0.152153000
1	-0.177335000	-1.671610000	-1.305576000
1	2.360284000	0.073726000	0.152378000
1	1.536755000	0.681086000	-1.305893000
1	1.636746000	1.703008000	0.149786000
1	0.000443000	0.000507000	1.728447000

PPhMe2H_1

15	1.563616000	-0.114953000	-0.346283000
6	2.234073000	-1.343778000	0.742954000
6	-0.201810000	-0.021937000	-0.158903000
6	-0.952705000	-1.192190000	-0.293057000
6	-2.329737000	-1.143187000	-0.144248000
6	-2.955070000	0.067581000	0.139784000
6	-2.207216000	1.230663000	0.273498000
6	-0.826698000	1.191225000	0.123303000
6	2.322579000	1.459561000	-0.061058000
1	1.747496000	-2.299860000	0.554723000
1	3.304449000	-1.433163000	0.563129000
1	2.052273000	-1.032025000	1.770802000
1	-0.462394000	-2.133014000	-0.513152000
1	-2.914714000	-2.046820000	-0.248372000
1	-4.029969000	0.102514000	0.257847000
1	-2.696045000	2.169546000	0.495384000

1	-0.246107000	2.098311000	0.228520000
1	1.940353000	2.193310000	-0.768332000
1	2.111328000	1.776490000	0.959648000
1	3.397717000	1.346712000	-0.193721000
1	1.840873000	-0.505340000	-1.645873000

PPhMe2H_2

15	1.551031000	-0.410103000	0.000011000
6	2.283542000	0.301388000	1.450098000
6	-0.206266000	-0.170602000	-0.000005000
6	-1.062740000	-1.269580000	-0.000119000
6	-2.436278000	-1.063744000	-0.000135000
6	-2.943969000	0.229995000	-0.000037000
6	-2.085812000	1.326285000	0.000077000
6	-0.713495000	1.130238000	0.000093000
6	2.283577000	0.301472000	-1.450019000
1	1.851774000	-0.155492000	2.338390000
1	3.359807000	0.136730000	1.426747000
1	2.075565000	1.371751000	1.445698000
1	-0.657211000	-2.273621000	-0.000194000
1	-3.107503000	-1.911786000	-0.000223000
1	-4.014410000	0.387447000	-0.000048000
1	-2.486518000	2.330809000	0.000154000
1	-0.039120000	1.979012000	0.000180000
1	1.851824000	-0.155349000	-2.338349000
1	2.075604000	1.371836000	-1.445555000
1	3.359840000	0.136808000	-1.426657000
1	1.753530000	-1.779311000	-0.000027000

PPh2MeH_1

15	1.551031000	-0.410103000	0.000011000
6	2.283542000	0.301388000	1.450098000
6	-0.206266000	-0.170602000	-0.000005000
6	-1.062740000	-1.269580000	-0.000119000
6	-2.436278000	-1.063744000	-0.000135000
6	-2.943969000	0.229995000	-0.000037000
6	-2.085812000	1.326285000	0.000077000
6	-0.713495000	1.130238000	0.000093000
6	2.283577000	0.301472000	-1.450019000
1	1.851774000	-0.155492000	2.338390000
1	3.359807000	0.136730000	1.426747000
1	2.075565000	1.371751000	1.445698000
1	-0.657211000	-2.273621000	-0.000194000
1	-3.107503000	-1.911786000	-0.000223000
1	-4.014410000	0.387447000	-0.000048000
1	-2.486518000	2.330809000	0.000154000
1	-0.039120000	1.979012000	0.000180000
1	1.851824000	-0.155349000	-2.338349000
1	2.075604000	1.371836000	-1.445555000
1	3.359840000	0.136808000	-1.426657000
1	1.753530000	-1.779311000	-0.000027000

PPh3H_1

15	0.092876000	-0.018651000	-0.818379000
6	-0.069798000	-1.687638000	-0.226193000
6	1.552511000	0.742696000	-0.158205000
6	-1.382771000	0.900443000	-0.463739000
6	-1.879101000	1.779278000	-1.426175000
6	-3.003957000	2.539298000	-1.137695000
6	-3.621204000	2.421215000	0.102458000
6	-3.123105000	1.542997000	1.060584000
6	-2.002251000	0.776097000	0.781455000
6	2.588628000	1.092286000	-1.021669000
6	3.732534000	1.687028000	-0.504423000
6	3.831925000	1.927098000	0.860343000
6	2.793499000	1.575750000	1.719965000
6	1.648818000	0.981310000	1.214567000
6	1.028613000	-2.346846000	0.324931000
6	0.891968000	-3.661086000	0.750970000
6	-0.330897000	-4.308959000	0.624614000
6	-1.425269000	-3.649811000	0.072177000
6	-1.300428000	-2.336712000	-0.353508000
1	-1.392216000	1.861132000	-2.390046000
1	-3.399553000	3.218406000	-1.880506000
1	-4.499387000	3.013264000	0.323515000
1	-3.610945000	1.452709000	2.021444000
1	-1.613758000	0.083358000	1.518804000
1	2.498631000	0.901271000	-2.083764000
1	4.542227000	1.962050000	-1.166368000
1	4.723451000	2.391755000	1.260115000
1	2.878360000	1.766214000	2.781092000
1	0.835525000	0.702522000	1.875233000
1	1.980404000	-1.840185000	0.421482000
1	1.739205000	-4.175619000	1.183178000
1	-0.434050000	-5.332316000	0.960258000
1	-2.375343000	-4.157446000	-0.022851000
1	-2.150940000	-1.818511000	-0.778912000
1	0.251551000	0.010661000	-2.196549000

Pani3H_1

15	0.132196000	-0.114360000	0.993670000
6	-0.783874000	-1.551726000	0.511923000
6	1.767773000	-0.189377000	0.328415000
6	-0.745894000	1.351423000	0.541966000
6	-0.924618000	2.366284000	1.477234000
6	-1.566034000	3.545671000	1.124439000
6	-2.026991000	3.706154000	-0.181847000
6	-1.849342000	2.686648000	-1.126634000
6	-1.214826000	1.518627000	-0.768182000
6	2.865649000	0.000183000	1.162310000
6	4.154203000	-0.016491000	0.646212000
6	4.337863000	-0.221632000	-0.720752000
6	3.234797000	-0.412937000	-1.563851000
6	1.960064000	-0.397981000	-1.044054000
6	-0.116987000	-2.755222000	0.246905000
6	-0.833502000	-3.882755000	-0.090087000
6	-2.229766000	-3.828662000	-0.168307000
6	-2.903610000	-2.635108000	0.096407000
6	-2.175432000	-1.505237000	0.434527000
8	5.548253000	-0.253391000	-1.315101000
8	-2.659635000	4.813973000	-0.619700000
8	-2.850190000	-4.975192000	-0.510033000
6	6.697851000	-0.058412000	-0.503126000
6	-2.859368000	5.877623000	0.300929000
6	-4.268348000	-4.968221000	-0.602834000
1	-0.564180000	2.238397000	2.490587000
1	-1.702285000	4.321049000	1.863517000
1	-2.220895000	2.836853000	-2.131336000
1	-1.083196000	0.728353000	-1.498698000
1	2.718499000	0.159979000	2.223593000
1	4.996183000	0.125774000	1.307219000
1	3.408028000	-0.573786000	-2.619400000
1	1.107771000	-0.551491000	-1.696610000
1	0.963200000	-2.805055000	0.303925000
1	-0.334799000	-4.818822000	-0.302418000
1	-3.980354000	-2.577436000	0.038480000
1	-2.701758000	-0.580844000	0.638471000
1	7.554228000	-0.112683000	-1.168133000
1	6.670484000	0.920036000	-0.021458000
1	6.774117000	-0.838966000	0.255347000
1	-3.370444000	6.663600000	-0.246413000
1	-3.478421000	5.554055000	1.138965000
1	-1.904823000	6.252485000	0.672958000
1	-4.718333000	-4.714251000	0.358019000
1	-4.603342000	-4.261683000	-1.363467000
1	-4.560411000	-5.974462000	-0.886834000
1	0.303330000	-0.031668000	2.369250000

Pani3H_2

15	0.087704000	-0.015385000	1.019078000
6	-0.790791000	1.459539000	0.583460000
6	-0.777835000	-1.441178000	0.435535000
6	1.754676000	0.087953000	0.440647000
6	2.797893000	-0.315221000	1.269781000
6	4.109061000	-0.292617000	0.816401000
6	4.373004000	0.135669000	-0.484641000
6	3.326799000	0.544329000	-1.321718000
6	2.028337000	0.522878000	-0.863142000
6	-1.260979000	-2.379908000	1.341447000
6	-1.939499000	-3.506014000	0.894869000
6	-2.130418000	-3.687511000	-0.474084000
6	-1.643726000	-2.744695000	-1.390112000
6	-0.973485000	-1.630106000	-0.939452000
6	-2.147824000	1.408640000	0.271609000
6	-2.845450000	2.566042000	-0.040962000
6	-2.174720000	3.789494000	-0.039743000
6	-0.811921000	3.847556000	0.276814000
6	-0.126048000	2.693779000	0.584209000
8	-2.775611000	-4.747120000	-1.003334000
8	5.611576000	0.192329000	-1.015336000
8	-2.764551000	4.964425000	-0.336673000
6	-3.289063000	-5.728572000	-0.113429000
6	6.705562000	-0.216643000	-0.206092000
6	-4.146801000	4.955549000	-0.666614000
1	2.590077000	-0.646507000	2.279976000
1	4.906999000	-0.601786000	1.475027000
1	3.561246000	0.877368000	-2.323782000
1	1.221755000	0.847170000	-1.510767000
1	-1.109435000	-2.234349000	2.404019000
1	-2.309917000	-4.225427000	1.609897000
1	-1.806774000	-2.912652000	-2.446176000
1	-0.598459000	-0.899725000	-1.647874000
1	-2.671487000	0.460817000	0.269123000
1	-3.895699000	2.505783000	-0.284953000
1	-0.314899000	4.808181000	0.268381000
1	0.928878000	2.744611000	0.823897000
1	-3.764178000	-6.483480000	-0.732252000
1	-2.485251000	-6.184272000	0.466575000
1	-4.026577000	-5.291741000	0.561479000
1	7.596547000	-0.100022000	-0.815229000
1	6.785836000	0.410423000	0.683018000

1	6.598848000	-1.261297000	0.089781000
1	-4.329022000	4.350550000	-1.555925000
1	-4.740991000	4.574290000	0.165115000
1	-4.419583000	5.987203000	-0.865949000
1	0.174579000	-0.185212000	2.394514000

Pani3H_3

15	0.008653000	-0.003203000	-1.242654000
6	-1.176810000	-1.200238000	-0.709449000
6	-0.446416000	1.623870000	-0.722288000
6	1.637622000	-0.416650000	-0.693410000
6	2.737247000	0.249854000	-1.251905000
6	4.009394000	-0.031801000	-0.808103000
6	4.203892000	-0.982079000	0.203430000
6	3.114837000	-1.648688000	0.763297000
6	1.834700000	-1.360257000	0.309422000
6	-1.542748000	2.250336000	-1.330676000
6	-1.944352000	3.496832000	-0.906691000
6	-1.259395000	4.136357000	0.135274000
6	-0.169304000	3.517920000	0.746714000
6	0.231171000	2.261593000	0.311897000
6	-2.002881000	-0.946025000	0.381128000
6	-2.897299000	-1.909777000	0.825937000
6	-2.960495000	-3.137856000	0.167514000
6	-2.130068000	-3.397132000	-0.930562000
6	-1.244475000	-2.436538000	-1.365051000
8	-1.720274000	5.354506000	0.484869000
8	5.482157000	-1.194402000	0.576895000
8	-3.799382000	-4.132338000	0.521892000
6	-1.058337000	6.044311000	1.536060000
6	5.729689000	-2.145412000	1.603283000
6	-4.668435000	-3.913069000	1.624353000
1	2.588492000	0.986511000	-2.032668000
1	4.872580000	0.467458000	-1.227218000
1	3.251117000	-2.384977000	1.541342000
1	0.988538000	-1.878832000	0.743533000
1	-2.073918000	1.755541000	-2.135122000
1	-2.784211000	4.001227000	-1.365155000
1	0.368584000	3.999988000	1.549410000
1	1.078623000	1.781063000	0.785477000
1	-1.954841000	0.009712000	0.889061000
1	-3.534601000	-1.696423000	1.671256000
1	-2.204330000	-4.356103000	-1.425359000
1	-0.603830000	-2.636495000	-2.215670000
1	-1.580320000	6.988261000	1.658436000
1	-1.109266000	5.475153000	2.465341000
1	-0.015215000	6.233700000	1.278553000
1	6.804836000	-2.161018000	1.752612000
1	5.235975000	-1.851810000	2.530810000
1	5.387479000	-3.136892000	1.303276000
1	-4.098975000	-3.726741000	2.536032000
1	-5.335492000	-3.072093000	1.430012000
1	-5.251619000	-4.821511000	1.738986000
1	0.009479000	-0.017491000	-2.631044000

Ppfp3H_1

15	0.000726000	0.001606000	1.016483000
6	1.349606000	-1.016187000	0.479900000
6	1.183910000	-1.920301000	-0.567912000
6	2.593993000	-0.855080000	1.095203000
6	2.264107000	-2.671112000	-1.006130000
1	0.218069000	-2.042491000	-1.041580000
6	3.676272000	-1.601201000	0.664803000
1	2.713924000	-0.149481000	1.908095000
6	3.482844000	-2.491973000	-0.378689000
1	2.170846000	-3.382285000	-1.814628000
1	4.651760000	-1.505145000	1.120070000
6	0.206221000	1.673644000	0.465339000
6	-0.544703000	2.677790000	1.081929000
6	1.059969000	1.970314000	-0.595806000
6	-0.439964000	3.984421000	0.640136000
1	-1.205927000	2.438220000	1.905499000
6	1.169247000	3.277245000	-1.045796000
1	1.639478000	1.188182000	-1.069609000
6	0.416194000	4.250825000	-0.416113000
1	-1.001264000	4.787106000	1.096944000
1	1.822351000	3.543583000	-1.864634000
6	-1.553415000	-0.658345000	0.474956000
6	-2.247230000	-0.063482000	-0.577685000
6	-2.043428000	-1.812496000	1.091950000
6	-3.438563000	-0.619410000	-1.018378000
1	-1.864243000	0.830819000	-1.052679000
6	-3.232161000	-2.372186000	0.659590000
1	-1.498260000	-2.270167000	1.908120000
6	-3.900646000	-1.759738000	-0.388054000
1	-4.003158000	-0.183010000	-1.830053000
1	-3.642686000	-3.260976000	1.117327000
9	0.520891000	5.514175000	-0.844716000
9	4.527642000	-3.216306000	-0.796705000
9	-5.051911000	-2.298344000	-0.806827000
1	-0.000646000	0.009103000	2.403760000

PCy3H_1

15	-0.001119000	0.292594000	-0.328258000
6	-1.764305000	-0.029833000	-0.213500000
6	0.506237000	1.507720000	0.910071000
6	0.975248000	-1.224157000	-0.231664000
6	-0.476496000	2.680702000	0.993101000
6	-0.418963000	3.545045000	-0.260456000
6	0.996914000	4.066906000	-0.482277000
6	2.004309000	2.925284000	-0.569198000
6	1.927742000	2.032111000	0.663473000
6	-2.131119000	-0.644919000	1.138495000
6	-3.640675000	-0.838075000	1.221358000
6	-4.139528000	-1.704027000	0.071547000
6	-3.756911000	-1.093690000	-1.270996000
6	-2.246778000	-0.908598000	-1.369895000
6	1.467712000	-1.535003000	1.184834000
6	2.220551000	-2.860184000	1.185556000
6	3.387685000	-2.816677000	0.208219000
6	2.895624000	-2.504791000	-1.198997000
6	2.136773000	-1.184387000	-1.232391000
1	-2.241146000	0.953935000	-0.308130000
1	0.479300000	0.944933000	1.848943000
1	0.284993000	-2.020352000	-0.537781000
1	-1.489718000	2.328910000	1.194117000
1	-0.170655000	3.282154000	1.854587000
1	-0.737063000	2.961963000	-1.133511000
1	-1.120856000	4.374742000	-0.167771000
1	1.040647000	4.680222000	-1.383590000
1	1.266558000	4.711361000	0.361714000
1	1.813708000	2.335390000	-1.472104000
1	3.016984000	3.319426000	-0.665684000
1	2.196518000	2.619161000	1.546895000
1	2.643500000	1.211452000	0.595554000
1	-1.633713000	-1.618148000	1.225584000
1	-1.773810000	-0.016222000	1.958327000
1	-3.904166000	-1.282794000	2.182036000
1	-4.125426000	0.142868000	1.171883000
1	-3.688766000	-2.699346000	0.152812000
1	-5.221124000	-1.833490000	0.135471000
1	-4.105701000	-1.720765000	-2.092792000
1	-4.242764000	-0.117927000	-1.376188000
1	-1.969360000	-0.461430000	-2.326833000
1	-1.763332000	-1.888894000	-1.308084000
1	2.144796000	-0.740925000	1.514298000
1	0.631107000	-1.565061000	1.885085000
1	2.569527000	-3.082517000	2.195028000
1	1.534224000	-3.662220000	0.893469000
1	4.089435000	-2.036725000	0.524416000
1	3.930069000	-3.763446000	0.219877000
1	3.729237000	-2.468421000	-1.901920000
1	2.225225000	-3.305483000	-1.529701000
1	1.756333000	-0.982170000	-2.235579000
1	2.814275000	-0.364858000	-0.968699000
1	0.206095000	0.798677000	-1.604621000

PCy3H_2

15	-0.003520000	-0.130457000	-0.681797000
6	-0.201430000	1.599288000	-0.198859000
6	1.423041000	-0.909650000	0.088614000
6	-1.513310000	-1.055536000	-0.391301000
6	2.705852000	-0.319947000	-0.510414000
6	3.932400000	-0.970600000	0.115943000
6	3.897697000	-2.479721000	-0.079505000
6	2.627984000	-3.062353000	0.525063000
6	1.382313000	-2.430485000	-0.085556000
6	0.402703000	2.547771000	-1.239507000
6	0.122329000	3.991808000	-0.843750000
6	0.657370000	4.289658000	0.551081000
6	0.051892000	3.336784000	1.573721000
6	0.347775000	1.888748000	1.202185000
6	-2.667284000	-0.526373000	-1.246205000
6	-3.910225000	-1.374666000	-1.000831000
6	-4.281775000	-1.375870000	0.476786000
6	-3.124341000	-1.886262000	1.325940000
6	-1.874659000	-1.044922000	1.095972000
1	-1.287564000	1.757122000	-0.178099000
1	1.371898000	-0.670212000	1.157429000
1	-1.293331000	-2.082523000	-0.705325000
1	2.703380000	-0.511190000	-1.589715000
1	2.732533000	0.762392000	-0.371105000
1	4.836272000	-0.542811000	-0.320180000
1	3.948723000	-0.744613000	1.187444000
1	3.921419000	-2.702675000	-1.151943000
1	4.778649000	-2.943545000	0.367091000
1	2.592898000	-4.143875000	0.386105000
1	2.625443000	-2.873104000	1.603882000
1	0.487661000	-2.842284000	0.384661000
1	1.334463000	-2.666892000	-1.155082000

1	1.484038000	2.383539000	-1.290981000
1	-0.008625000	2.329056000	-2.226818000
1	0.561453000	4.667844000	-1.578972000
1	-0.960477000	4.157082000	-0.855459000
1	1.746267000	4.167678000	0.550987000
1	0.448154000	5.325011000	0.824876000
1	0.438210000	3.545881000	2.572373000
1	-1.033132000	3.482970000	1.606376000
1	-0.083074000	1.202581000	1.934857000
1	1.431613000	1.733824000	1.204424000
1	-2.881777000	0.510545000	-0.967488000
1	-2.390774000	-0.534906000	-2.302500000
1	-4.737195000	-1.000091000	-1.605826000
1	-3.709037000	-2.401667000	-1.323378000
1	-4.525960000	-0.352992000	0.784320000
1	-5.172018000	-1.984321000	0.643914000
1	-3.387941000	-1.875041000	2.384465000
1	-2.906847000	-2.924852000	1.054694000
1	-1.038315000	-1.412579000	1.697227000
1	-2.072125000	-0.010579000	1.401384000
1	0.227928000	-0.153948000	-2.052949000

PCy3H_3

15	0.093041000	0.033528000	-0.479269000
6	-0.788315000	-1.520728000	-0.257606000
6	-0.728613000	1.449067000	0.271958000
6	1.803830000	-0.056332000	0.108926000
6	-2.145730000	1.637341000	-0.277518000
6	-2.799549000	2.870421000	0.335551000
6	-1.960553000	4.116359000	0.095274000
6	-0.565752000	3.924619000	0.671566000
6	0.116710000	2.710356000	0.053869000
6	-1.885896000	-1.756839000	-1.299439000
6	-2.481206000	-3.144132000	-1.086760000
6	-3.021671000	-3.292399000	0.330482000
6	-1.925014000	-3.042123000	1.358357000
6	-1.319937000	-1.656244000	1.172170000
6	2.730833000	0.613704000	-0.914921000
6	4.155247000	0.658163000	-0.378847000
6	4.650388000	-0.744008000	-0.050474000
6	3.717099000	-1.423191000	0.942861000
6	2.290058000	-1.475881000	0.408900000
1	-0.021099000	-2.288703000	-0.415939000
1	-0.775318000	1.227776000	1.345890000
1	1.818404000	0.520877000	1.041520000
1	-2.089446000	1.751653000	-1.366528000
1	-2.754944000	0.757564000	-0.069361000
1	-3.802979000	2.990580000	-0.075415000
1	-2.906938000	2.713146000	1.414147000
1	-1.883147000	4.297702000	-0.982413000
1	-2.440327000	4.990849000	0.537698000
1	0.049859000	4.809903000	0.505765000
1	-0.640309000	3.777577000	1.754255000
1	1.112016000	2.584746000	0.483157000
1	0.238932000	2.869630000	-1.024305000
1	-2.673783000	-1.007342000	-1.193613000
1	-1.472757000	-1.659853000	-2.305253000
1	-3.268509000	-3.324099000	-1.820276000
1	-1.701039000	-3.894554000	-1.254332000
1	-3.826878000	-2.565138000	0.483717000
1	-3.452586000	-4.285165000	0.469909000
1	-2.315721000	-3.142389000	2.371974000
1	-1.136791000	-3.792976000	1.236305000
1	-0.520585000	-1.477057000	1.896247000
1	-2.090997000	-0.897171000	1.341378000
1	2.701973000	0.021955000	-1.836621000
1	2.378467000	1.616133000	-1.164889000
1	4.808912000	1.138065000	-1.108798000
1	4.174166000	1.271851000	0.528315000
1	4.683342000	-1.335486000	-0.972030000
1	5.666270000	-0.706828000	0.346181000
1	4.061398000	-2.433954000	1.166659000
1	3.721882000	-0.862354000	1.883720000
1	1.633121000	-1.965657000	1.130021000
1	2.267853000	-2.065289000	-0.513978000
1	0.141575000	0.284458000	-1.847221000

PBu3H_1

15	0.001142000	0.000929000	0.329518000
6	0.302161000	-1.671703000	-0.211147000
6	1.300207000	1.096392000	-0.212710000
6	-1.597241000	0.576513000	-0.214615000
6	2.693131000	0.568285000	0.121239000
6	3.768205000	1.585932000	-0.232607000
6	5.161518000	1.064316000	0.077986000
6	-0.853696000	-2.612850000	0.118630000
6	-0.507525000	-4.054338000	-0.225471000
6	-1.661095000	-4.996917000	0.075395000
6	-1.839916000	2.045690000	0.122787000
6	-3.258814000	2.465279000	-0.233092000
6	-3.507908000	3.931661000	0.079757000
1	0.488193000	-1.630445000	-1.287660000
1	1.224951000	-2.002539000	0.272352000
1	1.171497000	1.234029000	-1.289644000
1	1.124834000	2.062657000	0.267289000
1	-1.647803000	0.400868000	-1.292805000
1	-2.347136000	-0.062097000	0.259732000
1	2.752518000	0.328007000	1.187200000
1	2.877285000	-0.360885000	-0.424329000
1	3.686798000	1.834267000	-1.294376000
1	3.579215000	2.509688000	0.320673000
1	5.367761000	0.152497000	-0.486018000
1	5.930016000	1.795684000	-0.172932000
1	5.257133000	0.827225000	1.139500000
1	-1.100307000	-2.539675000	1.182593000
1	-1.746370000	-2.310820000	-0.435538000
1	-0.238976000	-4.112226000	-1.283865000
1	0.379931000	-4.351930000	0.339355000
1	-2.545879000	-4.721284000	-0.501838000
1	-1.410391000	-6.029742000	-0.167099000
1	-1.928679000	-4.955330000	1.133131000
1	-1.663499000	2.214450000	1.189666000
1	-1.127668000	2.672684000	-0.420591000
1	-3.429754000	2.271758000	-1.295616000
1	-3.964115000	1.836743000	0.317202000
1	-3.354927000	4.131856000	1.142205000
1	-2.820689000	4.568767000	-0.480543000
1	-4.525066000	4.229831000	-0.174988000
1	-0.000161000	0.002470000	1.717247000

PBu3H_2

15	-0.168876000	-0.143389000	-0.510003000
6	0.819173000	-1.577259000	-0.913216000
6	0.531632000	1.326597000	-1.243329000
6	-1.854901000	-0.374377000	-1.053125000
6	-0.350462000	2.565275000	-1.067998000
6	-0.711943000	2.862623000	0.384532000
6	0.506907000	2.985440000	1.287289000
6	2.314739000	-1.368014000	-0.677468000
6	2.654305000	-0.980432000	0.755456000
6	4.156433000	-0.957445000	0.984993000
6	-2.462398000	-1.698707000	-0.583494000
6	-2.410406000	-1.899123000	0.928317000
6	-3.054747000	-0.757864000	1.701676000
1	0.436624000	-2.403297000	-0.307869000
1	0.604319000	-1.805991000	-1.959903000
1	1.518001000	1.466754000	-0.794135000
1	0.684623000	1.094176000	-2.299930000
1	-2.427392000	0.483927000	-0.693022000
1	-1.830311000	-0.315085000	-2.143806000
1	0.192242000	3.414738000	-1.487760000
1	-1.260602000	2.444036000	-1.658761000
1	-1.287368000	3.789408000	0.411343000
1	-1.380061000	2.082688000	0.769525000
1	0.224870000	3.313505000	2.287630000
1	1.031089000	2.031636000	1.391798000
1	1.216549000	3.709646000	0.880454000
1	2.825560000	-2.299671000	-0.930504000
1	2.693982000	-0.605838000	-1.363679000
1	2.237438000	0.007683000	0.983611000
1	2.175080000	-1.685405000	1.441568000
1	4.638495000	-0.250551000	0.306656000
1	4.403878000	-0.666125000	2.005699000
1	4.588798000	-1.942756000	0.800388000
1	-3.502310000	-1.716918000	-0.915887000
1	-1.952854000	-2.524465000	-1.084361000
1	-2.913876000	-2.837596000	1.166326000
1	-1.371003000	-2.024714000	1.252310000
1	-2.492124000	0.173047000	1.591833000
1	-4.070342000	-0.574422000	1.343205000
1	-3.107416000	-0.983318000	2.766674000
1	-0.172730000	0.015465000	0.868841000

PBu3H_3

15	-0.209090000	0.218185000	-0.192352000
6	-1.763634000	0.305765000	-1.069440000
6	0.429363000	-1.445833000	-0.207671000
6	0.957796000	1.375721000	-0.894983000
6	-0.599320000	-2.450486000	0.315716000
6	0.020037000	-3.822625000	0.556330000
6	0.600663000	-4.437431000	-0.708344000
6	-2.287960000	1.735146000	-1.226167000
6	-2.454342000	2.483153000	0.093689000
6	-3.339000000	1.746844000	1.089085000
6	2.379494000	1.207335000	-0.361980000
6	2.478088000	1.368178000	1.148439000
6	3.923365000	1.349254000	1.618033000
1	-1.588091000	-0.159413000	-2.042575000
1	-2.472409000	-0.325694000	-0.529494000
1	0.727878000	-1.659256000	-1.236804000

1	1.332442000	-1.451993000	0.410300000
1	0.911995000	1.224352000	-1.976402000
1	0.574031000	2.377755000	-0.685354000
1	-1.033889000	-2.074280000	1.246349000
1	-1.415465000	-2.538888000	-0.406325000
1	0.799820000	-3.731963000	1.317507000
1	-0.747094000	-4.481764000	0.967188000
1	1.447152000	-3.859463000	-1.082999000
1	0.951770000	-5.453423000	-0.527228000
1	-0.152633000	-4.477109000	-1.498672000
1	-1.620202000	2.292456000	-1.886305000
1	-3.254747000	1.673338000	-1.730063000
1	-1.472915000	2.675077000	0.542067000
1	-2.880460000	3.464218000	-0.122777000
1	-4.295290000	1.482063000	0.631964000
1	-2.872699000	0.823537000	1.440726000
1	-3.542511000	2.362765000	1.964961000
1	2.769638000	0.228363000	-0.653804000
1	3.010226000	1.954473000	-0.848511000
1	1.994681000	2.304975000	1.441383000
1	1.924014000	0.563684000	1.644228000
1	4.486796000	2.165127000	1.161105000
1	3.997175000	1.452815000	2.700509000
1	4.407622000	0.412250000	1.335553000
1	-0.445121000	0.554206000	1.133133000

POct3H_1

15	-1.608069000	-1.590700000	0.395036000
6	-1.410408000	-2.734375000	-0.967309000
6	-3.361894000	-1.494937000	0.766036000
6	-0.622683000	-2.162930000	1.774208000
6	-3.910130000	-0.168627000	1.303038000
6	-3.520255000	1.057887000	0.481427000
6	-3.754509000	0.913205000	-1.016828000
6	0.040475000	-3.059183000	-1.313923000
6	0.809608000	-1.825307000	-1.763406000
6	2.172523000	-2.146815000	-2.362360000
6	-0.784595000	-1.361199000	3.065721000
6	-0.585616000	0.139154000	2.888715000
6	0.722128000	0.566471000	2.234921000
6	3.121581000	-2.856526000	-1.399824000
6	3.369834000	-2.091397000	-0.103262000
6	3.863721000	-0.667075000	-0.328017000
6	4.405671000	-0.044199000	0.949006000
6	0.676750000	2.040564000	1.858433000
6	1.951584000	2.545381000	1.203967000
6	1.796288000	3.922490000	0.576057000
6	3.012719000	4.302972000	-0.252691000
6	-3.338267000	2.157658000	-1.801163000
6	-1.842243000	2.463876000	-1.739227000
6	-0.967891000	1.442834000	-2.459840000
6	0.502273000	1.616495000	-2.105065000
1	-1.138053000	-0.338923000	0.006859000
1	-1.969936000	-3.626803000	-0.674797000
1	-1.929400000	-2.283913000	-1.819333000
1	-3.857054000	-1.759585000	-0.171548000
1	-3.557216000	-2.311418000	1.465879000
1	-0.905457000	-3.208920000	1.919119000
1	0.413766000	-2.157398000	1.426111000
1	-4.997405000	-0.268965000	1.316611000
1	-3.594214000	-0.032889000	2.339156000
1	-4.085435000	1.918694000	0.849346000
1	-2.468349000	1.296556000	0.662567000
1	-3.210334000	0.044227000	-1.404790000
1	-4.813912000	0.704039000	-1.194241000
1	0.530025000	-3.531321000	-0.458098000
1	0.036717000	-3.798007000	-2.119262000
1	0.207734000	-1.294352000	-2.506624000
1	0.932852000	-1.133091000	-0.918289000
1	2.027537000	-2.772783000	-3.247803000
1	2.634342000	-1.218182000	-2.711046000
1	-1.782000000	-1.543068000	3.473621000
1	-0.067934000	-1.755632000	3.788555000
1	-1.410268000	0.541658000	2.290609000
1	-0.671461000	0.624962000	3.863760000
1	1.563872000	0.362453000	2.905095000
1	0.912550000	-0.010032000	1.317162000
1	2.734090000	-3.852722000	-1.168229000
1	4.080355000	-3.007253000	-1.904876000
1	2.455835000	-2.062663000	0.503850000
1	4.111546000	-2.632966000	0.492082000
1	4.643371000	-0.676571000	-1.097322000
1	3.047630000	-0.050901000	-0.725379000
1	4.728429000	0.987196000	0.795591000
1	3.643751000	-0.040422000	1.732986000
1	5.260009000	-0.614664000	1.319914000
1	-0.159699000	2.181552000	1.159517000
1	0.441954000	2.643657000	2.742146000
1	2.769801000	2.558960000	1.932515000
1	2.255768000	1.838247000	0.422732000
1	0.902609000	3.920231000	-0.059219000
1	1.618429000	4.666977000	1.357427000
1	2.915832000	5.294686000	-0.695747000
1	3.917973000	4.294701000	0.359024000
1	3.156935000	3.583624000	-1.064088000
1	-3.629773000	2.038431000	-2.849142000
1	-3.898869000	3.014620000	-1.415781000
1	-1.655784000	3.450304000	-2.174108000
1	-1.517294000	2.534864000	-0.695096000
1	-1.279852000	0.427368000	-2.194383000
1	-1.117757000	1.537913000	-3.539178000
1	1.150962000	0.953945000	-2.681980000
1	0.828677000	2.645736000	-2.279311000
1	0.654815000	1.394499000	-1.043716000

POct3H_2

15	2.169930000	-1.308596000	-0.178207000
6	2.018544000	-2.201301000	1.364509000
6	3.856850000	-0.747201000	-0.370831000
6	1.730517000	-2.349353000	-1.563075000
6	4.183491000	0.522158000	0.421217000
6	3.272712000	1.707425000	0.104376000
6	3.080032000	1.965784000	-1.385137000
6	0.625847000	-2.238229000	2.020741000
6	0.560232000	-1.333900000	3.246661000
6	0.691269000	0.151574000	2.930563000
6	0.379014000	-3.041218000	-1.405437000
6	-0.800227000	-2.081052000	-1.462131000
6	-2.118630000	-2.797040000	-1.207903000
6	-0.479032000	0.700984000	2.122749000
6	-0.536915000	2.222857000	2.132265000
6	-1.739686000	2.786798000	1.380885000
6	-3.058011000	2.503288000	2.088567000
6	-3.329608000	-1.870033000	-1.202656000
6	-3.465184000	-1.043444000	0.073165000
6	-4.732980000	-0.192511000	0.088656000
6	-4.666483000	0.987921000	-0.871568000
6	2.030306000	3.041237000	-1.650528000
6	0.600180000	2.539513000	-1.463400000
6	0.117939000	1.594905000	-2.559080000
6	-1.366933000	1.297635000	-2.411844000
1	1.314763000	-0.213313000	-0.157282000
1	2.382058000	-3.205767000	1.130859000
1	2.750841000	-1.754732000	2.044901000
1	4.478311000	-1.584774000	-0.043923000
1	4.033337000	-0.615872000	-1.439318000
1	1.758529000	-1.716499000	-2.455228000
1	2.545181000	-3.073812000	-1.644909000
1	4.139142000	0.302175000	1.491038000
1	5.219360000	0.781325000	0.194191000
1	2.296315000	1.560716000	0.578286000
1	3.691495000	2.603853000	0.568717000
1	4.041643000	2.246295000	-1.824390000
1	2.770063000	1.047997000	-1.896580000
1	-0.161529000	-1.958566000	1.313875000
1	0.416122000	-3.265925000	2.316670000
1	-0.391465000	-1.498618000	3.759503000
1	1.352441000	-1.635991000	3.937573000
1	0.751444000	0.705177000	3.872333000
1	1.636115000	0.348582000	2.405799000
1	0.279407000	-3.781135000	-2.202221000
1	0.359257000	-3.599483000	-0.463333000
1	-0.673625000	-1.273132000	-0.730430000
1	-0.824183000	-1.597263000	-2.444528000
1	-2.243702000	-3.569373000	-1.972597000
1	-2.059676000	-3.319576000	-0.244816000
1	-0.430762000	0.353528000	1.081078000
1	-1.407858000	0.289533000	2.534684000
1	-0.569145000	2.571872000	3.170948000
1	0.391934000	2.615266000	1.703315000
1	-1.618722000	3.866058000	1.257973000
1	-1.766090000	2.359947000	0.371488000
1	-3.902269000	2.947560000	1.558703000
1	-3.044469000	2.911641000	3.102053000
1	-3.247181000	1.430899000	2.165747000
1	-3.279113000	-1.211632000	-2.076956000
1	-4.242800000	-2.463003000	-1.313667000
1	-3.469616000	-1.730015000	0.926760000
1	-2.592900000	-0.390021000	0.204410000
1	-5.587503000	-0.828789000	-0.163245000
1	-4.912544000	0.180643000	1.099791000
1	-5.585290000	1.575556000	-0.845688000
1	-3.837756000	1.649502000	-0.606380000
1	-4.510489000	0.662280000	-1.902460000
1	2.215757000	3.887405000	-0.981419000
1	2.133023000	3.420048000	-2.671678000
1	0.501262000	2.029189000	-0.497608000
1	-0.082157000	3.395372000	-1.423043000

1	0.316536000	2.044399000	-3.537054000
1	0.683997000	0.655698000	-2.525036000
1	-1.949878000	2.221716000	-2.449615000
1	-1.567665000	0.823229000	-1.447138000
1	-1.735662000	0.631518000	-3.194181000

POct3H_3

15	1.043141000	-1.992305000	-0.067747000
6	0.621796000	-2.546753000	1.579170000
6	2.764912000	-2.398624000	-0.364590000
6	-0.090116000	-2.686670000	-1.263883000
6	3.580706000	-1.406471000	-1.200545000
6	3.513997000	0.041283000	-0.716238000
6	3.729654000	0.207345000	0.782217000
6	-0.835320000	-2.312713000	1.967571000
6	-1.230808000	-0.842586000	1.903142000
6	-2.568447000	-0.562517000	2.578535000
6	0.169948000	-2.265186000	-2.713296000
6	0.359088000	-0.762331000	-2.897701000
6	-0.745738000	0.118247000	-2.323858000
6	-3.722869000	-1.392022000	2.017636000
6	-3.912220000	-1.252935000	0.510382000
6	-4.141009000	0.183378000	0.059736000
6	-4.534096000	0.264073000	-1.406959000
6	-0.289637000	1.562638000	-2.162274000
6	-1.232830000	2.401000000	-1.315876000
6	-0.800155000	3.856196000	-1.217592000
6	-1.626763000	4.632485000	-0.205306000
6	3.909620000	1.667197000	1.189593000
6	2.733584000	2.574965000	0.826313000
6	1.391753000	2.120950000	1.396985000
6	1.429483000	1.906047000	2.903994000
1	0.898809000	-0.613150000	-0.101547000
1	0.892081000	-3.604527000	1.620112000
1	1.298917000	-2.006276000	2.249140000
1	3.199989000	-2.498607000	0.633429000
1	2.773165000	-3.393307000	-0.815733000
1	-0.027726000	-3.771195000	-1.147853000
1	-1.087978000	-2.385752000	-0.931548000
1	4.616265000	-1.750426000	-1.156506000
1	3.275477000	-1.465274000	-2.246994000
1	4.272909000	0.618880000	-1.252283000
1	2.553063000	0.487477000	-0.990673000
1	2.882749000	-0.220301000	1.336363000
1	4.610751000	-0.366852000	1.086684000
1	-1.487824000	-2.912171000	1.327628000
1	-0.973634000	-2.680203000	2.987679000
1	-0.446340000	-0.245246000	2.380198000
1	-1.273138000	-0.512573000	0.856715000
1	-2.471989000	-0.769316000	3.648410000
1	-2.800143000	0.502139000	2.486640000
1	1.062562000	-2.780189000	-3.076107000
1	-0.669542000	-2.621493000	-3.313005000
1	1.305639000	-0.468470000	-2.432018000
1	0.482204000	-0.548116000	-3.961777000
1	-1.634988000	0.069538000	-2.959050000
1	-1.062682000	-0.247805000	-1.338621000
1	-3.569034000	-2.445337000	2.268562000
1	-4.647405000	-1.088001000	2.517477000
1	-3.040084000	-1.657877000	-0.019899000
1	-4.766515000	-1.862721000	0.199995000
1	-4.921108000	0.634097000	0.682490000
1	-3.232829000	0.769771000	0.230377000
1	-4.660310000	1.295172000	-1.740883000
1	-3.771447000	-0.200553000	-2.036839000
1	-5.473524000	-0.264296000	-1.584267000
1	0.697650000	1.567948000	-1.679490000
1	-0.152995000	2.025492000	-3.145489000
1	-2.253069000	2.343860000	-1.714652000
1	-1.273019000	1.969329000	-0.305837000
1	0.259221000	3.892236000	-0.938201000
1	-0.874480000	4.322185000	-2.204376000
1	-1.328659000	5.679971000	-0.149743000
1	-2.687724000	4.598296000	-0.463331000
1	-1.515695000	4.197760000	0.791671000
1	4.084028000	1.719850000	2.267684000
1	4.813555000	2.055257000	0.710745000
1	2.948374000	3.580757000	1.200286000
1	2.651289000	2.661837000	-0.262334000
1	0.633906000	2.871601000	1.154097000
1	1.070646000	1.197297000	0.900492000
1	0.428105000	1.763255000	3.312326000
1	2.023811000	1.028335000	3.168934000
1	1.874396000	2.770300000	3.404203000

Methyl cations

CH3_1

6	0.000002000	0.000202000	-0.000018000
1	-0.009766000	-1.087570000	0.000035000
1	-0.936682000	0.551633000	0.000036000
1	0.946435000	0.534726000	0.000036000

PMe4_1

15	0.000201000	0.000009000	0.000243000
6	-1.237612000	0.694604000	1.066799000
6	-0.196053000	0.655318000	-1.638148000
6	-0.179784000	-1.765570000	-0.043529000
1	-2.227250000	0.441011000	0.687700000
1	-1.118328000	0.289971000	2.071603000
1	-1.125351000	1.778192000	1.095154000
1	-1.186360000	0.399704000	-2.014247000
1	-0.085425000	1.739115000	-1.611072000
1	0.563381000	0.228256000	-2.292792000
1	-1.169990000	-2.019656000	-0.421012000
1	0.579556000	-2.191461000	-0.699005000
1	-0.059249000	-2.167764000	0.962070000
6	1.613126000	0.415720000	0.614515000
1	1.734513000	0.009891000	1.618520000
1	2.371987000	-0.007408000	-0.043271000
1	1.721440000	1.499581000	0.644900000

PPh2Me2_1

15	-0.000059000	1.174936000	0.000154000
6	1.447583000	0.138107000	-0.054697000
6	-1.447549000	0.137864000	0.054702000
6	0.051229000	2.202282000	1.448728000
6	-1.527421000	-0.833091000	1.056713000
6	-2.647675000	-1.644350000	1.139093000
6	-3.684115000	-1.491689000	0.221811000
6	-3.603275000	-0.529395000	-0.776524000
6	-2.484570000	0.290436000	-0.863851000
6	1.528031000	-0.831832000	-1.057631000
6	2.648342000	-1.642991000	-1.140218000
6	3.684255000	-1.491253000	-0.222191000
6	3.602848000	-0.529970000	0.777071000
6	2.484083000	0.289759000	0.864602000
1	0.927919000	2.849083000	1.416023000
1	-0.850671000	2.814102000	1.460667000
1	0.078224000	1.581165000	2.343872000
1	-0.713706000	-0.955043000	1.763778000
1	-2.712625000	-2.397033000	1.913332000
1	-4.556376000	-2.128908000	0.285376000
1	-4.408759000	-0.415581000	-1.489540000
1	-2.425929000	1.038801000	-1.643912000
1	0.714687000	-0.953087000	-1.765244000
1	2.713719000	-2.394902000	-1.915169000
1	4.556562000	-2.128396000	-0.285900000
1	4.407935000	-0.416895000	1.490652000
1	2.425000000	1.037347000	1.645367000
6	-0.051528000	2.202697000	-1.448121000
1	0.850347000	2.814554000	-1.459995000
1	-0.078657000	1.581834000	-2.343435000
1	-0.928228000	2.849470000	-1.415112000

PPhMe3_1

15	-1.386410000	0.010813000	0.001121000
6	0.393331000	0.029682000	-0.007851000
6	-2.023069000	1.662360000	-0.108728000
6	-1.973906000	-0.941294000	-1.379639000
6	-1.955783000	-0.756368000	1.499637000
6	1.105413000	1.227710000	-0.000946000
6	2.494552000	1.200418000	0.002535000
6	3.166698000	-0.015330000	0.000899000
6	2.456098000	-1.212243000	-0.004397000
6	1.070148000	-1.192970000	-0.009044000
1	-3.111853000	1.607282000	-0.115666000
1	-1.699349000	2.247600000	0.751466000
1	-1.679934000	2.132989000	-1.029501000
1	-3.063317000	-0.972867000	-1.352498000
1	-1.640499000	-0.479124000	-2.308107000
1	-1.579776000	-1.955492000	-1.314416000
1	-3.045538000	-0.788382000	1.496692000
1	-1.562369000	-1.771761000	1.553658000
1	-1.605092000	-0.184768000	2.358245000
1	0.585838000	2.177230000	0.000956000
1	3.049730000	2.128774000	0.005962000
1	4.248741000	-0.032475000	0.003207000
1	2.982222000	-2.157336000	-0.005922000
1	0.516461000	-2.125657000	-0.015686000

Pani3Me_1

15	-0.101735000	-0.047483000	1.039104000
6	1.011706000	-1.340872000	0.555548000
6	0.470843000	1.519838000	0.437492000
6	-1.723981000	-0.381449000	0.404593000

6	-2.813416000	0.362013000	0.882852000
6	-4.077396000	0.140898000	0.384376000
6	-4.282127000	-0.828739000	-0.607687000
6	-3.203076000	-1.566200000	-1.096232000
6	-1.931858000	-1.336485000	-0.586545000
6	1.573390000	2.139386000	1.045132000
6	2.053657000	3.334495000	0.558569000
6	1.443279000	3.939279000	-0.549312000
6	0.344765000	3.331817000	-1.158909000
6	-0.131217000	2.125944000	-0.662152000
6	2.027381000	-1.120183000	-0.369696000
6	2.880534000	-2.149463000	-0.746775000
6	2.712742000	-3.418194000	-0.190892000
6	1.684839000	-3.648628000	0.735087000
6	0.845005000	-2.622364000	1.103180000
8	1.977136000	5.099612000	-0.957058000
8	-5.545360000	-0.983153000	-1.030113000
8	3.486651000	-4.473233000	-0.484428000
6	1.404800000	5.748011000	-2.081689000
6	-5.808847000	-1.956387000	-2.027994000
6	4.550810000	-4.297518000	-1.405926000
1	-2.665917000	1.120310000	1.643050000
1	-4.928455000	0.703350000	0.744790000
1	-3.342097000	-2.314448000	-1.862944000
1	-1.099086000	-1.913401000	-0.969628000
1	2.058014000	1.680228000	1.898446000
1	2.902174000	3.825163000	1.016660000
1	-0.142600000	3.787011000	-2.008701000
1	-0.983699000	1.659045000	-1.140491000
1	2.163036000	-0.136963000	-0.803660000
1	3.664455000	-1.954667000	-1.463978000
1	1.571584000	-4.641973000	1.148564000
1	0.048212000	-2.812340000	1.813676000
1	1.988153000	6.649310000	-2.245740000
1	1.459426000	5.112892000	-2.968031000
1	0.364710000	6.018656000	-1.889361000
1	-6.878178000	-1.922616000	-2.214555000
1	-5.272173000	-1.725817000	-2.950466000
1	-5.531740000	-2.954370000	-1.682598000
1	4.174556000	-3.993868000	-2.384623000
1	5.264197000	-3.555761000	-1.041714000
1	5.044054000	-5.261256000	-1.492096000
6	-0.174587000	0.001132000	2.820659000
1	0.822921000	0.186868000	3.218963000
1	-0.851353000	0.793662000	3.139372000
1	-0.536522000	-0.958755000	3.188899000

Pani3Me_2

15	-0.082792000	-0.025570000	1.060504000
6	1.006371000	-1.339072000	0.575763000
6	0.516184000	1.529163000	0.451015000
6	-1.711513000	-0.332196000	0.426344000
6	-2.783416000	0.424910000	0.901512000
6	-4.062610000	0.235198000	0.404285000
6	-4.277835000	-0.725663000	-0.588690000
6	-3.205588000	-1.481458000	-1.074623000
6	-1.935283000	-1.285728000	-0.572898000
6	1.603302000	2.146092000	1.071346000
6	2.115042000	3.338893000	0.585834000
6	1.535863000	3.926404000	-0.542904000
6	0.448115000	3.310178000	-1.171678000
6	-0.055405000	2.123800000	-0.680077000
6	2.011216000	-1.138837000	-0.366003000
6	2.840671000	-2.183674000	-0.752720000
6	2.660493000	-3.447428000	-0.189308000
6	1.644856000	-3.656556000	0.755155000
6	0.827382000	-2.615311000	1.131662000
8	1.960809000	5.077258000	-1.084097000
8	-5.478521000	-0.983883000	-1.127253000
8	3.410609000	-4.516864000	-0.492123000
6	3.063434000	5.740435000	-0.486412000
6	-6.600194000	-0.248424000	-0.665238000
6	4.453584000	-4.366914000	-1.442033000
1	-2.624135000	1.178668000	1.664104000
1	-4.878317000	0.830013000	0.788480000
1	-3.394568000	-2.217494000	-1.844629000
1	-1.113116000	-1.876881000	-0.956858000
1	2.064698000	1.694895000	1.941806000
1	2.953577000	3.801698000	1.085378000
1	0.014252000	3.783241000	-2.042598000
1	-0.898817000	1.655824000	-1.173030000
1	2.156343000	-0.159688000	-0.806074000
1	3.615338000	-2.004634000	-1.483919000
1	1.522189000	-4.646180000	1.174807000
1	0.039032000	-2.788983000	1.855500000
1	3.241851000	6.633011000	-1.078897000
1	2.836495000	6.027186000	0.542270000
1	3.954304000	5.109608000	-0.502604000
1	-7.457735000	-0.617462000	-1.220168000
1	-6.761606000	-0.411150000	0.402085000
1	-6.475223000	0.818571000	-0.859256000
1	4.057704000	-4.059571000	-2.411829000
1	5.191436000	-3.638591000	-1.100111000
1	4.924345000	-5.341046000	-1.536624000
6	-0.154893000	0.028560000	2.841697000
1	0.845576000	0.192001000	3.242005000
1	-0.814284000	0.836557000	3.157874000
1	-0.539296000	-0.922368000	3.210340000

Pani3Me_3

15	-0.108901000	-0.024961000	1.085045000
6	0.971825000	-1.351848000	0.616907000
6	0.504869000	1.517465000	0.458966000
6	-1.737637000	-0.324097000	0.446690000
6	-2.802429000	0.457954000	0.896999000
6	-4.080257000	0.275340000	0.393697000
6	-4.301536000	-0.703820000	-0.579985000
6	-3.236502000	-1.485198000	-1.040459000
6	-1.967344000	-1.296060000	-0.533202000
6	1.586468000	2.138710000	1.084425000
6	2.109376000	3.322636000	0.589165000
6	1.546521000	3.897082000	-0.554347000
6	0.466517000	3.274556000	-1.190437000
6	-0.047769000	2.096785000	-0.689523000
6	1.970300000	-1.170950000	-0.346136000
6	2.776047000	-2.227701000	-0.717726000
6	2.597105000	-3.487123000	-0.135581000
6	1.599270000	-3.678265000	0.825708000
6	0.794842000	-2.611614000	1.192046000
8	1.979848000	5.041048000	-1.103466000
8	-5.501559000	-0.956502000	-1.122758000
8	3.422990000	-4.456956000	-0.554619000
6	3.072044000	5.712928000	-0.496392000
6	-6.614272000	-0.188254000	-0.693601000
6	3.281907000	-5.755008000	0.000089000
1	-2.638385000	1.224998000	1.645203000
1	-4.890441000	0.889604000	0.758518000
1	-3.429731000	-2.234695000	-1.796292000
1	-1.150432000	-1.906116000	-0.898289000
1	2.034098000	1.698686000	1.967622000
1	2.943655000	3.788632000	1.092798000
1	0.046651000	3.736637000	-2.074004000
1	-0.885385000	1.624542000	-1.188251000
1	2.116677000	-0.200136000	-0.803577000
1	3.554574000	-2.103609000	-1.458672000
1	1.447245000	-4.644135000	1.285016000
1	0.015140000	-2.769232000	1.929040000
1	3.256766000	6.600175000	-1.094922000
1	2.829348000	6.008988000	0.526037000
1	3.964696000	5.084505000	-0.493712000
1	-7.471703000	-0.553660000	-1.251089000
1	-6.793215000	-0.321697000	0.375016000
1	-6.465202000	0.870992000	-0.911620000
1	3.450584000	-5.739785000	1.078674000
1	2.292203000	-6.165183000	-0.210525000
1	4.037383000	-6.374247000	-0.474579000
6	-0.188045000	0.055365000	2.864622000
1	0.811404000	0.216501000	3.268159000
1	-0.842437000	0.874237000	3.163104000
1	-0.583418000	-0.885982000	3.245726000

PCy3Me_1

15	0.028825000	-0.088655000	0.690263000
6	0.073642000	1.641062000	0.143457000
6	-1.303620000	-0.995271000	-0.124069000
6	1.606686000	-0.887655000	0.330678000
6	-2.675776000	-0.514256000	0.364597000
6	-3.781662000	-1.229808000	-0.402136000
6	-3.667155000	-2.736851000	-0.214585000
6	-2.298271000	-3.230386000	-0.662780000
6	-1.173108000	-2.509313000	0.073514000
6	-0.635846000	2.598416000	1.108288000
6	-0.451103000	4.037666000	0.638921000
6	-0.959045000	4.225817000	-0.784231000
6	-0.256088000	3.264891000	-1.733602000
6	-0.457786000	1.822436000	-1.284362000
6	2.788131000	-0.231772000	1.052906000
6	4.058719000	-1.034708000	0.788959000
6	4.336501000	-1.144328000	-0.704587000
6	3.150221000	-1.765869000	-1.430313000
6	1.880076000	-0.962266000	-1.174302000
1	1.142167000	1.895027000	0.145109000
1	-1.207134000	-0.772066000	-1.193962000
1	1.494385000	-1.906241000	0.724820000
1	-2.772859000	-0.752813000	1.429186000
1	-2.769603000	0.569249000	0.263434000
1	-4.755375000	-0.871435000	-0.064613000
1	-3.696498000	-0.986263000	-1.466812000
1	-3.806900000	-2.975068000	0.845831000

1	-4.454963000	-3.252290000	-0.766356000
1	-2.205751000	-4.306443000	-0.507342000
1	-2.185217000	-3.048610000	-1.737247000
1	-0.208555000	-2.867198000	-0.290895000
1	-1.226537000	-2.733586000	1.145321000
1	-1.703525000	2.355365000	1.138987000
1	-0.244750000	2.482579000	2.120278000
1	-0.962024000	4.715195000	1.324925000
1	0.615688000	4.285191000	0.674675000
1	-2.036946000	4.029978000	-0.810690000
1	-0.811496000	5.257729000	-1.107015000
1	-0.627453000	3.386949000	-2.752314000
1	0.816156000	3.489135000	-1.749503000
1	0.030671000	1.133054000	-1.975652000
1	-1.528265000	1.592844000	-1.299512000
1	2.924078000	0.785433000	0.670672000
1	2.603699000	-0.163007000	2.126176000
1	4.900082000	-0.568386000	1.303984000
1	3.937602000	-2.038804000	1.209565000
1	4.516860000	-0.142197000	-1.109516000
1	5.240394000	-1.729610000	-0.881121000
1	3.341555000	-1.824040000	-2.502877000
1	3.000060000	-2.789922000	-1.070847000
1	1.031375000	-1.402081000	-1.704809000
1	2.015112000	0.056520000	-1.556120000
6	-0.232022000	-0.176832000	2.451404000
1	-1.180606000	0.286657000	2.717693000
1	-0.243259000	-1.226453000	2.749890000
1	0.570500000	0.339596000	2.976164000

PCy3Me_2

15	-0.075633000	0.026669000	0.536746000
6	0.831112000	-1.509983000	0.240881000
6	0.715015000	1.440483000	-0.267049000
6	-1.757124000	-0.117404000	-0.145916000
6	2.117242000	1.706441000	0.289075000
6	2.768955000	2.879766000	-0.434646000
6	1.909978000	4.130977000	-0.325421000
6	0.523184000	3.866022000	-0.892938000
6	-0.153638000	2.701836000	-0.176832000
6	1.964219000	-1.817084000	1.225308000
6	2.533736000	-3.198430000	0.914321000
6	3.018381000	-3.285919000	-0.527483000
6	1.888376000	-2.965366000	-1.497751000
6	1.315206000	-1.583239000	-1.210507000
6	-2.773600000	0.616802000	0.741373000
6	-4.144590000	0.626007000	0.077230000
6	-4.622382000	-0.795813000	-0.182040000
6	-3.610011000	-1.549292000	-1.032926000
6	-2.227496000	-1.555113000	-0.387611000
1	0.071541000	-2.288046000	0.390858000
1	0.795242000	1.153027000	-1.323949000
1	-1.704441000	0.390368000	-1.118072000
1	2.038430000	1.940637000	1.356688000
1	2.741486000	0.817195000	0.190930000
1	3.764323000	3.053800000	-0.022959000
1	2.896189000	2.620578000	-1.491393000
1	1.820584000	4.416043000	0.728914000
1	2.382209000	4.965577000	-0.846146000
1	-0.105361000	4.754512000	-0.815322000
1	0.610986000	3.623930000	-1.957719000
1	-1.137277000	2.524976000	-0.615485000
1	-0.303457000	2.961510000	0.877779000
1	2.758738000	-1.071202000	1.137383000
1	1.597262000	-1.787307000	2.252364000
1	3.345169000	-3.424685000	1.607863000
1	1.751284000	-3.948149000	1.076021000
1	3.832214000	-2.567238000	-0.676793000
1	3.424671000	-4.277989000	-0.731120000
1	2.238955000	-3.014915000	-2.529734000
1	1.093079000	-3.711242000	-1.387738000
1	0.499343000	-1.346810000	-1.900438000
1	2.096351000	-0.829593000	-1.359936000
1	-2.841845000	0.088203000	1.698486000
1	-2.446659000	1.634486000	0.960822000
1	-4.854166000	1.163965000	0.707913000
1	-4.078703000	1.168101000	-0.872676000
1	-4.743134000	-1.312284000	0.776711000
1	-5.598611000	-0.789559000	-0.669604000
1	-3.936035000	-2.576556000	-1.203452000
1	-3.536026000	-1.067479000	-2.014144000
1	-1.528589000	-2.091253000	-1.031889000
1	-2.269402000	-2.087133000	0.569814000
6	-0.143455000	0.311427000	2.295030000
1	-0.682970000	1.233832000	2.506668000
1	-0.647159000	-0.524626000	2.781910000
1	0.871620000	0.392566000	2.683660000

PCy3Me_3

15	0.156349000	-0.185373000	0.424017000
6	-1.547398000	-0.756492000	0.244490000
6	1.186194000	-1.036174000	-0.803542000
6	0.217599000	1.610568000	0.122727000
6	0.842864000	-2.507596000	-1.065678000
6	1.402063000	-3.449806000	-0.010133000
6	2.914692000	-3.291243000	0.069772000
6	3.276718000	-1.859479000	0.440717000
6	2.691656000	-0.851668000	-0.547043000
6	-2.110076000	-0.405587000	-1.137368000
6	-3.499618000	-1.009167000	-1.305102000
6	-4.435705000	-0.506865000	-0.214029000
6	-3.872590000	-0.825585000	1.164331000
6	-2.476613000	-0.235860000	1.345677000
6	0.527544000	1.975497000	-1.333745000
6	0.380373000	3.478751000	-1.538373000
6	1.310037000	4.242098000	-0.605469000
6	1.034982000	3.865379000	0.843422000
6	1.166805000	2.361675000	1.063913000
1	-1.487484000	-1.848605000	0.342476000
1	0.930123000	-0.484107000	-1.713216000
1	-0.806077000	1.942319000	0.347206000
1	-0.232044000	-2.642024000	-1.197166000
1	1.305383000	-2.759136000	-2.025962000
1	0.956923000	-3.232649000	0.967690000
1	1.133857000	-4.477433000	-0.259999000
1	3.339886000	-3.985940000	0.795879000
1	3.346927000	-3.537535000	-0.906603000
1	2.913764000	-1.664121000	1.451955000
1	4.360270000	-1.731944000	0.468753000
1	3.190456000	-0.984531000	-1.511816000
1	2.903226000	0.172311000	-0.224481000
1	-2.183428000	0.685459000	-1.216405000
1	-1.442392000	-0.746822000	-1.932545000
1	-3.891573000	-0.764475000	-2.293473000
1	-3.423864000	-2.100600000	-1.246767000
1	-4.549985000	0.578367000	-0.313726000
1	-5.427699000	-0.946605000	-0.329618000
1	-4.532606000	-0.449271000	1.947514000
1	-3.813621000	-1.912635000	1.286366000
1	-2.092715000	-0.495415000	2.332995000
1	-2.533479000	0.857011000	1.285908000
1	1.559856000	1.683950000	-1.557266000
1	-0.121290000	1.432300000	-2.023493000
1	0.586787000	3.728537000	-2.580280000
1	-0.656906000	3.767209000	-1.334728000
1	2.348059000	3.992152000	-0.851778000
1	1.195547000	5.318030000	-0.747334000
1	1.712300000	4.393817000	1.516288000
1	0.015798000	4.170070000	1.106521000
1	0.951066000	2.125265000	2.105861000
1	2.197143000	2.048638000	0.861938000
6	0.653330000	-0.469323000	2.113992000
1	1.686859000	-0.158015000	2.257338000
1	0.019287000	0.129335000	2.768211000
1	0.542255000	-1.522835000	2.368393000

PBu3Me_1

15	0.000205000	0.000170000	-0.226714000
6	-0.330093000	-1.656547000	0.359866000
6	-1.270119000	1.102379000	0.380287000
6	1.596862000	0.537850000	0.369494000
6	-2.643911000	0.870195000	-0.246705000
6	-3.687692000	1.805912000	0.349308000
6	-5.057187000	1.600111000	-0.277634000
6	0.585343000	-2.725741000	-0.233042000
6	0.261281000	-4.102868000	0.331938000
6	1.151477000	-5.182595000	-0.261956000
6	2.060201000	1.870874000	-0.214485000
6	3.418330000	2.276373000	0.343562000
6	3.895969000	3.598509000	-0.234950000
1	-0.244447000	-1.626469000	1.450088000
1	-1.375691000	-1.874801000	0.123271000
1	-1.305738000	0.969512000	1.465777000
1	-0.930782000	2.124146000	0.188699000
1	1.527216000	0.587900000	1.460130000
1	2.309524000	-0.254728000	0.124250000
1	-2.587570000	1.032257000	-1.327048000
1	-2.958479000	-0.166825000	-0.095810000
1	-3.739382000	1.640584000	1.429063000
1	-3.360583000	2.839802000	0.207209000
1	-5.404622000	0.576083000	-0.125116000
1	-5.799574000	2.272832000	0.152323000
1	-5.023251000	1.782641000	-1.353799000
1	0.474235000	-2.747362000	-1.320821000
1	1.631789000	-2.485180000	-0.023918000
1	0.374326000	-4.077844000	1.419336000
1	-0.789713000	-4.330274000	0.132396000
1	2.203549000	-4.977064000	-0.053440000
1	0.915454000	-6.165631000	0.146041000

1	1.031184000	-5.232039000	-1.346218000
1	2.125904000	1.794870000	-1.303753000
1	1.329177000	2.654107000	0.006032000
1	3.350502000	2.344810000	1.432856000
1	4.142581000	1.486666000	0.124816000
1	3.986553000	3.536902000	-1.321489000
1	3.191314000	4.400515000	-0.005255000
1	4.868886000	3.883178000	0.166200000
6	0.000974000	0.009318000	-2.006746000
1	0.896823000	-0.492149000	-2.373653000
1	-0.882440000	-0.513123000	-2.374924000
1	-0.011092000	1.037692000	-2.368336000

PBu3Me_1

15	-0.211239000	-0.276681000	0.573873000
6	0.232965000	-1.858781000	-0.131811000
6	0.970031000	0.981535000	0.111327000
6	-1.833712000	0.197253000	-0.006402000
6	0.586597000	2.381321000	0.595367000
6	1.701936000	3.392275000	0.343786000
6	2.053718000	3.538598000	-1.129463000
6	1.683798000	-2.284830000	0.139338000
6	2.635127000	-1.925109000	-0.995009000
6	4.062839000	-2.341336000	-0.679951000
6	-2.884020000	-0.901570000	0.168125000
6	-4.291540000	-0.397524000	-0.136848000
6	-4.453873000	0.084056000	-1.571195000
1	-0.479220000	-2.569748000	0.294630000
1	0.030040000	-1.810782000	-1.205943000
1	1.938710000	0.678407000	0.523294000
1	1.050807000	0.945623000	-0.978044000
1	-2.118813000	1.098778000	0.545648000
1	-1.718321000	0.480349000	-1.056197000
1	-0.320900000	2.705904000	0.078439000
1	0.356341000	2.353111000	1.663185000
1	1.387347000	4.358589000	0.742846000
1	2.588870000	3.092536000	0.909261000
1	2.778285000	4.338659000	-1.282377000
1	2.489049000	2.623044000	-1.533930000
1	1.164267000	3.775098000	-1.718794000
1	2.048951000	-1.846441000	1.074911000
1	1.706210000	-3.366160000	0.287039000
1	2.291828000	-2.418929000	-1.908335000
1	2.594793000	-0.850273000	-1.190898000
1	4.120423000	-3.416031000	-0.494412000
1	4.740677000	-2.105944000	-1.500623000
1	4.427947000	-1.831016000	0.214053000
1	-2.645680000	-1.737225000	-0.495968000
1	-2.848771000	-1.285399000	1.191190000
1	-4.997817000	-1.206680000	0.059361000
1	-4.538714000	0.410861000	0.557241000
1	-3.847062000	0.968615000	-1.772566000
1	-4.154601000	-0.694940000	-2.276737000
1	-5.490907000	0.345487000	-1.782719000
6	-0.239159000	-0.441180000	2.345834000
1	-0.558386000	0.495943000	2.801145000
1	-0.933549000	-1.233699000	2.624869000
1	0.758530000	-0.695772000	2.703751000

PBu3Me_3

15	-0.372265000	-0.435633000	0.674798000
6	-0.220606000	1.007396000	-0.368664000
6	-1.352584000	-1.687877000	-0.156458000
6	1.252803000	-1.113640000	0.998442000
6	-2.557343000	-1.188666000	-0.954484000
6	-3.627224000	-0.488736000	-0.126998000
6	-4.890662000	-0.253949000	-0.940413000
6	0.709773000	2.072429000	0.209125000
6	0.814457000	3.278386000	-0.715193000
6	1.744413000	4.342403000	-0.154727000
6	2.103789000	-1.319227000	-0.251680000
6	3.456197000	-1.930310000	0.091580000
6	4.315352000	-2.133374000	-1.146056000
1	0.138003000	0.659755000	-1.343061000
1	-1.228069000	1.407653000	-0.516486000
1	-0.658056000	-2.212767000	-0.817927000
1	-1.660273000	-2.398889000	0.615776000
1	1.748147000	-0.432157000	1.696525000
1	1.096047000	-2.058385000	1.528209000
1	-2.220314000	-0.523975000	-1.754812000
1	-3.001437000	-2.058575000	-1.444790000
1	-3.853665000	-1.091033000	0.758509000
1	-3.248697000	0.471966000	0.234901000
1	-5.652562000	0.267061000	-0.360421000
1	-4.673338000	0.347099000	-1.826050000
1	-5.313595000	-1.201918000	-1.278748000
1	0.343367000	2.394640000	1.188377000
1	1.707275000	1.649676000	0.366083000
1	1.170462000	2.945229000	-1.694232000
1	-0.184227000	3.695657000	-0.871216000
1	2.751172000	3.943338000	-0.014160000
1	1.815557000	5.202826000	-0.820375000
1	1.389047000	4.697270000	0.814990000
1	1.583923000	-1.969616000	-0.961417000
1	2.258355000	-0.359563000	-0.755123000
1	3.970463000	-1.278983000	0.803868000
1	3.297668000	-2.885348000	0.600088000
1	3.821872000	-2.800026000	-1.856443000
1	4.496882000	-1.183319000	-1.653059000
1	5.282774000	-2.568990000	-0.895355000
6	-1.075213000	0.011498000	2.249000000
1	-2.037344000	0.504365000	2.122668000
1	-1.205294000	-0.890986000	2.846863000
1	-0.389078000	0.684485000	2.763994000

Zwitterions

1_PMe3_1

15	-2.161957000	0.043359000	0.113024000
6	-2.429000000	1.690469000	0.731329000
6	-3.747693000	-0.649977000	-0.320722000
6	-1.451137000	-0.943233000	1.408303000
1	-3.096159000	1.657320000	1.592449000
1	-1.472572000	2.120322000	1.028121000
1	-3.607113000	-1.662753000	-0.698945000
1	-4.211626000	-0.039437000	-1.095697000
1	-2.093382000	-0.898401000	2.288369000
1	-1.356018000	-1.973993000	1.070198000
1	-4.398567000	-0.678589000	0.553112000
1	-0.461783000	-0.554180000	1.646441000
1	-2.875464000	2.304523000	-0.051233000
6	-1.055981000	0.114213000	-1.298619000
6	0.267752000	0.690439000	-0.911177000
6	1.268132000	-0.169804000	-0.489772000
8	1.192739000	-1.410444000	-0.345730000
8	2.472394000	0.458010000	-0.209574000
6	3.526857000	-0.380548000	0.224257000
6	4.732157000	0.494299000	0.478444000
1	-1.578324000	0.689637000	-2.069253000
1	-0.956733000	-0.915066000	-1.660506000
1	3.233900000	-0.915586000	1.131753000
1	3.745531000	-1.135216000	-0.535888000
1	5.574444000	-0.110404000	0.816880000
1	5.028169000	1.016606000	-0.432385000
1	4.513438000	1.239003000	1.245125000
1	0.454027000	1.750160000	-1.013711000

1_PMe3_2

15	-1.985767000	0.143235000	0.023740000
6	-1.070212000	1.552917000	-0.552363000
6	-3.547707000	0.093056000	-0.837194000
6	-2.315451000	0.356993000	1.759309000
1	-1.623912000	2.460398000	-0.309519000
1	-0.925049000	1.481854000	-1.629032000
1	-4.100953000	1.016744000	-0.667876000
1	-4.134698000	-0.751228000	-0.474961000
1	-1.369848000	0.407325000	2.298853000
1	-2.872963000	1.280221000	1.916179000
1	-3.366110000	-0.028679000	-1.905268000
1	-2.898705000	-0.487557000	2.126844000
1	-0.097198000	1.565980000	-0.060942000
6	-1.044110000	-1.365729000	-0.215650000
6	0.273692000	-1.277978000	0.484735000
6	1.359495000	-0.773434000	-0.208541000
8	1.371997000	-0.328635000	-1.378174000
8	2.549564000	-0.801348000	0.510806000
6	3.602739000	-0.004023000	-0.000090000
6	3.381291000	1.463929000	0.303888000
1	-0.918750000	-1.481441000	-1.297566000
1	-1.680132000	-2.183678000	0.136982000
1	4.509908000	-0.365002000	0.486722000
1	3.702003000	-0.160371000	-1.075206000
1	3.290001000	1.623377000	1.379684000
1	4.214881000	2.064917000	-0.063943000
1	2.467027000	1.809720000	-0.181724000
1	0.380089000	-1.635628000	1.499061000

1_PMe3_2

15	-2.139220000	-0.039226000	0.013542000
6	-1.501586000	-1.021378000	1.349687000
6	-2.639549000	1.541118000	0.660506000
6	-3.583240000	-0.850197000	-0.649808000
1	-2.250118000	-1.080819000	2.140457000
1	-1.262753000	-2.020219000	0.987998000
1	-1.774060000	2.038337000	1.097954000
1	-3.403040000	1.399782000	1.425273000
1	-4.002712000	-0.244077000	-1.453150000
1	-4.332666000	-0.984237000	0.130150000

1	-3.041497000	2.154557000	-0.146248000
1	-3.298284000	-1.824559000	-1.047289000
1	-0.593694000	-0.553447000	1.728933000
6	-0.879344000	0.206704000	-1.240422000
6	0.326961000	0.875076000	-0.663545000
6	1.339358000	0.087538000	-0.143716000
8	1.370722000	-1.161764000	-0.076877000
8	2.413790000	0.813681000	0.357779000
6	3.570358000	0.071170000	0.699802000
6	4.389999000	-0.280006000	-0.524939000
1	-1.354876000	0.775061000	-2.045778000
1	-0.639688000	-0.788218000	-1.630992000
1	3.285313000	-0.833526000	1.238905000
1	4.142820000	0.710299000	1.374110000
1	3.805401000	-0.910664000	-1.195266000
1	4.684514000	0.623606000	-1.061330000
1	5.294318000	-0.821161000	-0.240290000
1	0.415484000	1.952191000	-0.679169000

1_PPhMe2_1

6	-0.263679000	0.197783000	-1.358202000
6	-1.692799000	0.615147000	-1.279645000
6	-2.613854000	-0.282260000	-0.765198000
8	-2.381988000	-1.420616000	-0.301725000
8	-3.925813000	0.172315000	-0.805701000
6	-4.859969000	-0.552697000	-0.025308000
6	-4.735092000	-0.208158000	1.444886000
1	0.293570000	0.652747000	-2.182572000
1	-0.162495000	-0.889634000	-1.445042000
1	-4.723269000	-1.623870000	-0.179831000
1	-5.843362000	-0.271135000	-0.404426000
1	-4.882351000	0.861739000	1.601981000
1	-5.479657000	-0.748024000	2.032675000
1	-3.743547000	-0.483890000	1.808069000
1	-2.004899000	1.579651000	-1.653991000
15	0.616970000	0.656954000	0.145937000
6	2.300071000	0.058854000	0.109686000
6	2.498510000	-1.324655000	0.075769000
6	3.784368000	-1.838907000	0.002288000
6	4.875832000	-0.976398000	-0.041190000
6	4.681928000	0.399240000	-0.013317000
6	3.395644000	0.919994000	0.060331000
1	1.646858000	-1.995950000	0.107634000
1	3.936129000	-2.909895000	-0.021583000
1	5.878965000	-1.378653000	-0.097866000
1	5.531209000	1.068918000	-0.047748000
1	3.250604000	1.992686000	0.081604000
6	-0.194343000	-0.032686000	1.570417000
1	0.292543000	0.340991000	2.471300000
1	-1.241447000	0.271905000	1.551803000
1	-0.138189000	-1.119372000	1.538438000
6	0.619950000	2.427715000	0.303652000
1	1.134409000	2.725852000	1.216496000
1	1.101754000	2.882607000	-0.561659000
1	-0.418381000	2.757596000	0.350058000

1_PPhMe2_2

6	0.238155000	0.670969000	-0.971224000
6	1.652892000	0.984037000	-0.619436000
6	2.569104000	-0.054845000	-0.555795000
8	2.349534000	-1.272960000	-0.732824000
8	3.861039000	0.351219000	-0.263655000
6	4.836683000	-0.672691000	-0.195949000
6	6.160383000	-0.021990000	0.130826000
1	0.160479000	-0.227841000	-1.591619000
1	-0.281842000	1.481778000	-1.491390000
1	4.888625000	-1.206973000	-1.148216000
1	4.561395000	-1.405006000	0.567919000
1	6.436511000	0.697972000	-0.640788000
1	6.107126000	0.502255000	1.086088000
1	6.946620000	-0.775170000	0.195361000
1	1.960784000	2.005342000	-0.445315000
15	-0.726591000	0.330557000	0.514004000
6	-0.684683000	1.769460000	1.562685000
6	-2.434875000	-0.015527000	0.120364000
6	-3.181011000	0.989852000	-0.501468000
6	-4.498230000	0.753989000	-0.865023000
6	-5.075814000	-0.486845000	-0.612748000
6	-4.336171000	-1.490471000	0.000712000
6	-3.015735000	-1.259065000	0.367001000
1	-2.729375000	1.955716000	-0.699441000
1	-5.073411000	1.534899000	-1.344629000
1	-6.104035000	-0.670590000	-0.896082000
1	-4.785874000	-2.455223000	0.195369000
1	-2.445526000	-2.046001000	0.843825000
6	-0.029068000	-1.039266000	1.401552000
1	-0.064902000	-1.940649000	0.792604000
1	1.013516000	-0.798553000	1.609644000
1	-0.569873000	-1.187744000	2.336232000
1	0.354370000	1.996509000	1.802120000
1	-1.130349000	2.617631000	1.042007000
1	-1.239740000	1.571589000	2.479095000

1_PPhMe2_3

6	-0.518558000	-0.904441000	-1.249347000
6	-1.884718000	-1.464204000	-1.031006000
6	-2.924618000	-0.681389000	-0.548986000
8	-4.090748000	-1.007063000	-0.256705000
8	-2.555357000	0.656180000	-0.393019000
6	-3.447498000	1.468896000	0.344797000
6	-2.845080000	2.852348000	0.424936000
1	-0.519559000	0.136455000	-1.584447000
1	0.072262000	-1.475157000	-1.972509000
1	-3.592763000	1.047161000	1.345409000
1	-4.426346000	1.494183000	-0.138463000
1	-1.872810000	2.823627000	0.921719000
1	-2.707751000	3.269027000	-0.573899000
1	-3.497489000	3.519600000	0.989235000
1	-2.065620000	-2.515825000	-1.205552000
15	0.449650000	-0.949250000	0.276570000
6	1.999188000	-0.084283000	0.068547000
6	3.234309000	-0.726507000	0.146219000
6	4.403037000	-0.005677000	-0.067977000
6	4.341411000	1.351054000	-0.361497000
6	3.110127000	1.994623000	-0.445013000
6	1.940817000	1.279954000	-0.233151000
1	3.289932000	-1.783359000	0.374576000
1	5.360824000	-0.505038000	-0.004303000
1	5.253720000	1.909169000	-0.527302000
1	3.062322000	3.050524000	-0.676165000
1	0.979847000	1.780168000	-0.302408000
6	0.758922000	-2.646975000	0.703758000
1	1.318695000	-2.709080000	1.636318000
1	-0.207466000	-3.136650000	0.831056000
1	1.308810000	-3.142185000	-0.096265000
6	-0.423442000	-0.194968000	1.633113000
1	-1.405045000	-0.661602000	1.723431000
1	0.150263000	-0.359015000	2.545744000
1	-0.541125000	0.873223000	1.456709000

1_PPh2Me_1

6	0.437952000	-0.823370000	-1.238983000
6	1.814583000	-0.269229000	-1.113473000
6	2.776786000	-1.014484000	-0.448264000
8	2.623925000	-2.119932000	0.115460000
8	4.031546000	-0.429015000	-0.434823000
6	5.057361000	-1.162288000	0.209894000
6	6.328864000	-0.352243000	0.112502000
1	0.429469000	-1.917785000	-1.200154000
1	-0.077597000	-0.509348000	-2.149860000
1	5.178810000	-2.138409000	-0.267163000
1	4.789938000	-1.346240000	1.253893000
1	6.597259000	-0.180461000	-0.930746000
1	6.207497000	0.616346000	0.599581000
1	7.151423000	-0.879789000	0.596895000
1	2.064730000	0.678319000	-1.568663000
15	-0.583301000	-0.277160000	0.152729000
6	-0.696693000	1.506186000	0.123738000
6	-2.237410000	-0.956597000	0.088635000
6	-2.827473000	-1.216136000	-1.150368000
6	-4.110938000	-1.742880000	-1.210747000
6	-4.806182000	-2.014896000	-0.038270000
6	-4.220741000	-1.760937000	1.197503000
6	-2.939009000	-1.232903000	1.263777000
6	-1.737915000	2.133024000	-0.562600000
6	-1.783621000	3.518192000	-0.637730000
6	-0.792817000	4.280580000	-0.028462000
6	0.244723000	3.659903000	0.657272000
6	0.297137000	2.274042000	0.734933000
1	-2.289220000	-1.007784000	-2.066692000
1	-4.565013000	-1.943637000	-2.172058000
1	-5.805175000	-2.428298000	-0.087007000
1	-4.761112000	-1.976274000	2.109864000
1	-2.486826000	-1.039096000	2.228384000
1	-2.515575000	1.543021000	-1.032609000
1	-2.593726000	4.001297000	-1.167819000
1	-0.831642000	5.360692000	-0.085729000
1	1.014858000	4.253046000	1.132344000
1	1.108155000	1.794051000	1.267443000
6	0.175145000	-0.774608000	1.682510000
1	0.082035000	-1.854589000	1.789255000
1	1.232354000	-0.515018000	1.642782000
1	-0.303411000	-0.269195000	2.520831000

1_PPh2Me_2

6	-0.022542000	-2.072358000	0.217971000
6	-1.346487000	-2.321681000	-0.428218000
6	-2.498260000	-1.642670000	-0.047048000
8	-3.629054000	-1.674026000	-0.569124000
8	-2.314028000	-0.850775000	1.081285000
6	-3.403934000	-0.030249000	1.458838000

6	-3.029742000	0.667847000	2.745684000
1	0.658070000	-2.928571000	0.180112000
1	-0.111935000	-1.759789000	1.259699000
1	-4.302975000	-0.636286000	1.589559000
1	-3.614497000	0.694816000	0.666164000
1	-2.842892000	-0.059637000	3.537149000
1	-2.129090000	1.269736000	2.611710000
1	-3.837835000	1.325771000	3.068168000
1	-1.395158000	-2.950054000	-1.307308000
15	0.797359000	-0.736821000	-0.681997000
6	-0.229406000	0.720697000	-0.728367000
6	2.383949000	-0.274714000	0.005579000
6	3.156715000	0.699346000	-0.631974000
6	4.392255000	1.051180000	-0.111385000
6	4.859014000	0.433683000	1.046514000
6	4.091858000	-0.532661000	1.683926000
6	2.851580000	-0.889257000	1.165736000
6	-0.087528000	1.701630000	0.253451000
6	-0.936017000	2.800409000	0.260337000
6	-1.924820000	2.920813000	-0.710727000
6	-2.067102000	1.944754000	-1.690848000
6	-1.220596000	0.843644000	-1.703233000
1	2.785927000	1.182277000	-1.529274000
1	4.990925000	1.805674000	-0.604412000
1	5.823878000	0.709761000	1.451721000
1	4.455530000	-1.009582000	2.584394000
1	2.254498000	-1.642457000	1.664561000
1	0.679334000	1.604183000	1.013178000
1	-0.826457000	3.559937000	1.023390000
1	-2.586422000	3.777538000	-0.702903000
1	-2.838463000	2.037380000	-2.443904000
1	-1.340824000	0.080545000	-2.461474000
6	1.084735000	-1.286838000	-2.353078000
1	1.795399000	-2.113489000	-2.325901000
1	0.149390000	-1.628342000	-2.794722000
1	1.495835000	-0.470135000	-2.946184000

1_PPh2Me_3

6	-0.587024000	-0.402438000	-1.386989000
6	-1.870663000	-1.083917000	-1.083915000
6	-2.874240000	-0.311014000	-0.529332000
8	-2.794945000	0.910285000	-0.244463000
8	-4.057265000	-0.976471000	-0.277950000
6	-5.108602000	-0.200803000	0.270677000
6	-6.303303000	-1.108187000	0.446475000
1	-0.743060000	0.604367000	-1.789080000
1	0.035298000	-0.951506000	-2.096969000
1	-5.345542000	0.633580000	-0.394279000
1	-4.799194000	0.228055000	1.227578000
1	-6.615957000	-1.523924000	-0.512286000
1	-6.065278000	-1.934289000	1.117982000
1	-7.140662000	-0.551890000	0.869364000
1	-1.998087000	-2.142942000	-1.254722000
15	0.479359000	-0.135968000	0.083267000
6	1.857028000	-1.271580000	0.066902000
6	1.109281000	1.538280000	0.082982000
6	0.163562000	2.561534000	-0.041249000
6	0.579476000	3.885395000	-0.022761000
6	1.930578000	4.189511000	0.112932000
6	2.869439000	3.171699000	0.235721000
6	2.462248000	1.843343000	0.222930000
6	2.656154000	-1.324663000	-1.079222000
6	3.733987000	-2.194547000	-1.132908000
6	4.014290000	-3.019449000	-0.046882000
6	3.218968000	-2.973279000	1.090974000
6	2.139785000	-2.099227000	1.151868000
1	-0.888060000	2.307846000	-0.146771000
1	-0.149636000	4.679425000	-0.117159000
1	2.252272000	5.222922000	0.122929000
1	3.919433000	3.410282000	0.343263000
1	3.194675000	1.052162000	0.324406000
1	2.433975000	-0.681414000	-1.923986000
1	4.352951000	-2.232051000	-2.019462000
1	4.853765000	-3.701141000	-0.090814000
1	3.435993000	-3.617380000	1.932783000
1	1.523071000	-2.068625000	2.040811000
6	-0.443848000	-0.362449000	1.585272000
1	-1.295887000	0.317621000	1.546565000
1	-0.804874000	-1.388114000	1.649736000
1	0.189389000	-0.123219000	2.439561000

1_PPh3_1

6	-0.286121000	0.040401000	-1.771814000
6	-1.579672000	0.745952000	-1.580655000
6	-2.765656000	0.039591000	-1.697912000
8	-2.914968000	-1.187947000	-1.893119000
8	-3.895331000	0.840253000	-1.597549000
6	-5.114496000	0.164805000	-1.341750000
6	-5.213745000	-0.253869000	0.111124000
1	0.376707000	0.472213000	-2.531067000
1	-0.458403000	-1.003919000	-2.051798000
1	-5.204881000	-0.702348000	-1.997353000
1	-5.904229000	0.873809000	-1.594018000
1	-4.404009000	-0.944487000	0.355150000
1	-5.139660000	0.615259000	0.766834000
1	-6.164676000	-0.753123000	0.305532000
1	-1.602108000	1.819522000	-1.454826000
15	0.715949000	-0.003783000	-0.264222000
6	-0.166972000	-0.800419000	1.061360000
6	2.242278000	-0.881973000	-0.586280000
6	1.088369000	1.665741000	0.260548000
6	2.704438000	-1.881469000	0.270676000
6	3.873571000	-2.567529000	-0.032181000
6	4.581829000	-2.263298000	-1.188305000
6	4.122194000	-1.271657000	-2.048557000
6	2.954960000	-0.582809000	-1.752747000
6	0.076162000	2.404712000	0.882017000
6	0.310078000	3.721270000	1.251901000
6	1.550819000	4.302477000	1.011513000
6	2.561402000	3.566878000	0.404285000
6	2.333953000	2.249175000	0.028189000
6	-1.228429000	-1.659689000	0.776853000
6	-1.871218000	-2.315488000	1.820308000
6	-1.458541000	-2.117208000	3.132920000
6	-0.402040000	-1.256608000	3.414861000
6	0.242399000	-0.592876000	2.381020000
1	2.151854000	-2.130410000	1.167467000
1	4.227381000	-3.342529000	0.634755000
1	5.491135000	-2.801587000	-1.422207000
1	4.670393000	-1.035908000	-2.951032000
1	2.603873000	0.189934000	-2.426226000
1	-0.885720000	1.946611000	1.077694000
1	-0.474483000	4.290660000	1.732266000
1	1.731844000	5.328388000	1.305051000
1	3.529942000	4.015242000	0.226600000
1	3.129301000	1.680062000	-0.435074000
1	-1.574938000	-1.794843000	-0.242510000
1	-2.700425000	-2.976792000	1.604450000
1	-1.964854000	-2.629778000	3.940750000
1	-0.084762000	-1.099121000	4.437178000
1	1.060507000	0.084778000	2.596836000

1_PPh3_2

6	0.235954000	-0.133744000	-1.902902000
6	1.113648000	-1.333541000	-1.861958000
6	2.451121000	-1.271858000	-1.481629000
8	3.252303000	-2.211640000	-1.322169000
8	2.907337000	0.024447000	-1.271873000
6	4.250499000	0.151506000	-0.842119000
6	4.522872000	1.620185000	-0.617509000
1	-0.543846000	-0.154783000	-2.670483000
1	0.802639000	0.788107000	-2.046309000
1	4.926211000	-0.262094000	-1.595238000
1	4.403680000	-0.420478000	0.077081000
1	5.550163000	1.767952000	-0.282147000
1	4.377622000	2.186136000	-1.538973000
1	3.851436000	2.022040000	0.143890000
1	0.676847000	-2.314184000	-1.998108000
15	-0.626018000	0.057543000	-0.313592000
6	-1.615239000	-1.397600000	-0.010566000
6	0.571882000	0.211346000	0.998900000
6	-1.703853000	1.489790000	-0.318346000
6	0.866555000	1.453483000	1.560942000
6	1.888709000	1.564495000	2.494752000
6	2.619071000	0.440307000	2.863247000
6	2.315886000	-0.802770000	2.316483000
6	1.288839000	-0.923335000	1.391227000
6	-2.992805000	1.442964000	0.214483000
6	-3.802339000	2.570336000	0.168804000
6	-3.331981000	3.743611000	-0.409594000
6	-2.050046000	3.793688000	-0.946559000
6	-1.236636000	2.670071000	-0.905608000
6	-1.920429000	-1.774389000	1.298391000
6	-2.748557000	-2.864485000	1.529812000
6	-3.269907000	-3.579734000	0.457841000
6	-2.968793000	-3.205275000	-0.847327000
6	-2.143933000	-2.114736000	-1.084934000
1	0.304667000	2.332754000	1.272548000
1	2.115601000	2.528643000	2.930407000
1	3.420447000	0.530757000	3.585377000
1	2.877635000	-1.679630000	2.610478000
1	1.049297000	-1.889587000	0.966395000
1	-3.365855000	0.529209000	0.659738000
1	-4.801391000	2.530306000	0.582305000
1	-3.966407000	4.619720000	-0.445424000
1	-1.684365000	4.705295000	-1.400167000
1	-0.237806000	2.713511000	-1.323906000
1	-1.511621000	-1.217233000	2.132815000
1	-2.982339000	-3.156086000	2.545099000

1	-3.910303000	-4.433075000	0.639646000
1	-3.372092000	-3.764824000	-1.680778000
1	-1.910190000	-1.829941000	-2.103016000

1_PPh3_3

6	0.025822000	0.016706000	-2.087151000
6	1.405062000	-0.511121000	-2.154458000
6	2.362197000	0.251553000	-1.506234000
8	2.151127000	1.370021000	-0.979768000
8	3.620196000	-0.301366000	-1.431860000
6	4.552433000	0.377039000	-0.606333000
6	5.757325000	-0.519001000	-0.445935000
1	-0.677027000	-0.537240000	-2.710958000
1	-0.037488000	1.075076000	-2.361330000
1	4.095510000	0.596205000	0.362921000
1	4.830709000	1.333296000	-1.058105000
1	6.511578000	-0.031707000	0.172989000
1	5.472746000	-1.458079000	0.032648000
1	6.202775000	-0.747807000	-1.415042000
1	1.602086000	-1.529219000	-2.457023000
15	-0.713511000	-0.014549000	-0.381194000
6	-1.471722000	1.559651000	-0.002140000
6	-2.002951000	-1.259920000	-0.316302000
6	0.496489000	-0.418428000	0.862891000
6	-2.934192000	-1.307105000	-1.358199000
6	-3.980436000	-2.216567000	-1.315319000
6	-4.103162000	-3.084693000	-0.234871000
6	-3.184401000	-3.036551000	0.806068000
6	-2.137019000	-2.123823000	0.770250000
6	1.175000000	-1.634069000	0.746602000
6	2.172760000	-1.958031000	1.658005000
6	2.484697000	-1.076400000	2.686794000
6	1.794323000	0.125282000	2.815347000
6	0.803718000	0.458218000	1.901878000
6	-0.725649000	2.716530000	-0.249972000
6	-1.275140000	3.959681000	0.032070000
6	-2.561309000	4.051497000	0.554311000
6	-3.301545000	2.900867000	0.802350000
6	-2.759785000	1.652265000	0.525820000
1	-2.851298000	-0.626368000	-2.197763000
1	-4.698244000	-2.248471000	-2.124256000
1	-4.917549000	-3.796912000	-0.204663000
1	-3.281153000	-3.707813000	1.649124000
1	-1.428533000	-2.085151000	1.587597000
1	0.917021000	-2.324659000	-0.046159000
1	2.701986000	-2.897324000	1.564858000
1	3.263731000	-1.328760000	3.394893000
1	2.032918000	0.806681000	3.621216000
1	0.282644000	1.403148000	1.989814000
1	0.281369000	2.622004000	-0.643987000
1	-0.699989000	4.856561000	-0.157102000
1	-2.988252000	5.022828000	0.768692000
1	-4.301072000	2.974315000	1.210225000
1	-3.337494000	0.756929000	0.719515000

1_Pani3_1

6	0.176575000	-0.288675000	-2.199702000
6	0.908631000	-1.582547000	-2.278068000
6	2.286967000	-1.684087000	-2.119947000
8	2.983768000	-2.716375000	-2.073769000
8	2.930230000	-0.455056000	-2.013071000
6	4.332407000	-0.502537000	-1.822307000
6	4.828267000	0.918083000	-1.686773000
15	-0.391998000	0.019675000	-0.500874000
6	1.015422000	0.028926000	0.582643000
6	-1.258766000	1.577462000	-0.391086000
6	-1.479885000	-1.293661000	0.001720000
6	1.651097000	-1.183937000	0.888242000
6	2.848521000	-1.189302000	1.571360000
6	3.436049000	0.017492000	1.968723000
6	2.791241000	1.227166000	1.707620000
6	1.588771000	1.222077000	1.012333000
6	-0.748060000	2.693138000	-1.072418000
6	-1.401097000	3.906519000	-1.021290000
6	-2.588092000	4.034886000	-0.290706000
6	-3.110879000	2.931918000	0.385173000
6	-2.443950000	1.714917000	0.327844000
6	-2.239895000	-1.986808000	-0.938003000
6	-3.133812000	-2.973402000	-0.545728000
6	-3.275266000	-3.272729000	0.810519000
6	-2.518905000	-2.578756000	1.761612000
6	-1.630296000	-1.603413000	1.359585000
8	4.618280000	-0.078036000	2.603859000
8	-4.109960000	-4.212617000	1.285950000
8	-3.160601000	5.251225000	-0.297028000
6	5.274956000	1.118769000	2.980861000
6	-4.883652000	-4.955579000	0.359505000
6	-4.363647000	5.431686000	0.429720000
1	0.817800000	0.559224000	-2.449872000
1	-0.708842000	-0.218030000	-2.839586000
1	0.345053000	-2.506017000	-2.316288000
1	4.568649000	-1.085519000	-0.927648000
1	4.809779000	-1.003781000	-2.668453000
1	5.908081000	0.928162000	-1.532052000
1	4.604678000	1.494915000	-2.585589000
1	4.353456000	1.410029000	-0.835402000
1	1.209081000	-2.121224000	0.575146000
1	3.356677000	-2.116777000	1.801038000
1	3.218106000	2.166889000	2.027214000
1	1.104490000	2.167347000	0.800824000
1	0.170109000	2.610749000	-1.642112000
1	-1.014324000	4.772198000	-1.542612000
1	-4.029148000	3.008770000	0.949307000
1	-2.864134000	0.863952000	0.849930000
1	-2.136742000	-1.768741000	-1.993727000
1	-3.704307000	-3.502351000	-1.295242000
1	-2.640919000	-2.828691000	2.807267000
1	-1.044036000	-1.075870000	2.102924000
1	6.212670000	0.823515000	3.443233000
1	5.481970000	1.742619000	2.107944000
1	4.679976000	1.685400000	3.700443000
1	-5.473419000	-5.656194000	0.943725000
1	-5.551991000	-4.303034000	-0.206147000
1	-4.243522000	-5.508241000	-0.331123000
1	-5.154443000	4.783390000	0.046683000
1	-4.212939000	5.233185000	1.492908000
1	-4.651772000	6.470236000	0.294259000

1_Pani3_2

6	-0.155894000	0.196657000	2.456845000
6	-1.549065000	-0.225254000	2.724288000
6	-2.540317000	0.599167000	2.225081000
8	-2.345547000	1.681183000	1.620480000
8	-3.835000000	0.165015000	2.438284000
6	-4.867435000	0.939703000	1.851405000
6	-5.034496000	0.623623000	0.379690000
15	0.396591000	0.033574000	0.695245000
6	1.622738000	-1.261385000	0.584183000
6	-0.937024000	-0.382469000	-0.399672000
6	1.161210000	1.550907000	0.161024000
6	2.671005000	-1.284802000	1.516449000
6	3.669004000	-2.231021000	1.428279000
6	3.643886000	-3.184527000	0.402766000
6	2.611604000	-3.168891000	-0.534219000
6	1.613942000	-2.205682000	-0.437961000
6	-1.263583000	0.410972000	-1.503548000
6	-2.306947000	0.056138000	-2.334903000
6	-3.055343000	-1.095276000	-2.068795000
6	-2.728642000	-1.905329000	-0.980430000
6	-1.673695000	-1.541646000	-0.153466000
6	0.483622000	2.757361000	0.397726000
6	1.033836000	3.952901000	-0.014498000
6	2.273316000	3.970810000	-0.665733000
6	2.953416000	2.776129000	-0.905049000
6	2.390775000	1.575156000	-0.490869000
8	4.651704000	-4.074301000	0.395053000
8	2.737457000	5.180821000	-1.026383000
8	-4.078346000	-1.346546000	-2.907468000
6	4.668741000	-5.059971000	-0.622865000
6	3.994751000	5.251975000	-1.674903000
6	-4.895480000	-2.473640000	-2.648254000
1	0.003517000	1.260389000	2.665911000
1	0.576443000	-0.370918000	3.033845000
1	-1.773771000	-1.192648000	3.149192000
1	-5.773539000	0.690113000	2.405530000
1	-4.658197000	2.001549000	1.990174000
1	-5.851102000	1.208741000	-0.048052000
1	-5.257944000	-0.436407000	0.237353000
1	-4.114370000	0.862446000	-0.155014000
1	2.711748000	-0.547838000	2.310454000
1	4.481504000	-2.256168000	2.142457000
1	2.576076000	-3.892963000	-1.335265000
1	0.821612000	-2.197998000	-1.175860000
1	-0.702195000	1.313693000	-1.710071000
1	-2.574812000	0.665916000	-3.187718000
1	-3.287830000	-2.804507000	-0.765558000
1	-1.413613000	-2.179046000	0.682352000
1	-0.482568000	2.732046000	0.893079000
1	0.524174000	4.892087000	0.157336000
1	3.910247000	2.770638000	-1.406647000
1	2.924303000	0.651793000	-0.681490000
1	5.548686000	-5.671292000	-0.444130000
1	3.776117000	-5.687408000	-0.576272000
1	4.740634000	-4.601808000	-1.611591000
1	4.180037000	6.303911000	-1.872931000
1	4.788369000	4.858541000	-1.035967000
1	3.982012000	4.703068000	-2.618854000
1	-5.668359000	-2.476499000	-3.411765000
1	-4.321069000	-3.400402000	-2.712156000

1	-5.360677000	-2.401666000	-1.662157000

1_Pani3_3

6	0.029169000	0.063044000	-2.453877000
6	-1.169762000	-0.739434000	-2.785093000
6	-2.381992000	-0.233905000	-2.351931000
8	-2.542512000	0.863632000	-1.765222000
8	-3.480377000	-1.031103000	-2.613204000
6	-4.733719000	-0.562950000	-2.143941000
6	-4.934171000	-0.874504000	-0.675257000
15	0.511479000	0.053291000	-0.665140000
6	-0.719168000	-0.719337000	0.354626000
6	2.037796000	-0.854429000	-0.474994000
6	0.797461000	1.719978000	-0.107578000
6	-1.078959000	-2.049501000	0.092739000
6	-2.059175000	-2.667456000	0.841624000
6	-2.705505000	-1.965332000	1.864548000
6	-2.335054000	-0.651711000	2.153367000
6	-1.349311000	-0.038142000	1.390459000
6	2.233548000	-1.776756000	0.548510000
6	3.450396000	-2.431111000	0.701350000
6	4.495475000	-2.152777000	-0.178657000
6	4.313461000	-1.215241000	-1.203219000
6	3.100410000	-0.577679000	-1.348232000
6	-0.151708000	2.694967000	-0.420396000
6	0.011971000	4.005891000	0.005795000
6	1.141432000	4.350475000	0.751407000
6	2.094364000	3.376297000	1.069216000
6	1.922919000	2.074206000	0.644791000
8	-3.681283000	-2.627807000	2.514000000
8	1.391078000	5.593121000	1.202071000
8	5.705825000	-2.734515000	-0.115014000
6	-4.440310000	-1.920645000	3.477998000
6	0.457960000	6.617048000	0.904086000
6	5.935790000	-3.696460000	0.899765000
1	0.921245000	-0.270284000	-2.987051000
1	-0.111090000	1.128307000	-2.667633000
1	-1.084108000	-1.733782000	-3.198411000
1	-4.816700000	0.510894000	-2.318817000
1	-5.483809000	-1.071528000	-2.751604000
1	-5.917407000	-0.534795000	-0.342614000
1	-4.861265000	-1.949269000	-0.496047000
1	-4.168799000	-0.368279000	-0.086018000
1	-0.579904000	-2.599680000	-0.695074000
1	-2.354974000	-3.689449000	0.643813000
1	-2.816018000	-0.097760000	2.946382000
1	-1.082182000	0.988881000	1.606503000
1	1.431892000	-1.995236000	1.242726000
1	3.571820000	-3.146468000	1.501817000
1	5.138783000	-1.008150000	-1.871672000
1	2.978316000	0.152291000	-2.140608000
1	-1.033364000	2.411062000	-0.987868000
1	-0.735817000	4.744211000	-0.245573000
1	2.960970000	3.666566000	1.648740000
1	2.667317000	1.328518000	0.896495000
1	-5.198646000	-2.609390000	3.839699000
1	-3.816915000	-1.599115000	4.315044000
1	-4.924468000	-1.050103000	3.028477000
1	0.847073000	7.527182000	1.351702000
1	0.359928000	6.757086000	-0.174447000
1	-0.521321000	6.393082000	1.332342000
1	5.826824000	-3.253923000	1.892188000
1	5.251408000	-4.541676000	0.801000000
1	6.957257000	-4.042413000	0.769923000

1_PCy3_1

6	0.337879000	0.258852000	-1.816572000
6	1.708971000	0.794849000	-1.561294000
6	2.781888000	-0.057042000	-1.380572000
8	2.793514000	-1.308627000	-1.423636000
8	3.978927000	0.603760000	-1.127776000
6	5.124153000	-0.214928000	-0.987492000
6	6.305716000	0.690729000	-0.729110000
15	-0.577745000	-0.019703000	-0.280086000
6	-2.305413000	-0.528111000	-0.603441000
6	0.216241000	-1.330366000	0.668038000
6	-0.598326000	1.502207000	0.700903000
6	-3.324214000	0.023239000	0.400317000
6	-4.703499000	-0.567327000	0.129090000
6	-5.151976000	-0.263474000	-1.294049000
6	-4.138348000	-0.798959000	-2.295987000
6	-2.750270000	-0.223416000	-2.038920000
6	-0.459501000	-1.539510000	2.027265000
6	0.337450000	-2.547603000	2.848805000
6	0.458804000	-3.873265000	2.107042000
6	1.073636000	-3.670463000	0.727713000
6	0.273688000	-2.660671000	-0.089064000
6	0.670106000	1.811453000	1.503807000
6	0.421084000	3.040942000	2.372608000
6	0.024659000	4.239997000	1.519020000
6	-1.211450000	3.927970000	0.684091000
6	-0.977787000	2.696195000	-0.182649000
1	-0.275131000	0.936426000	-2.417894000
1	0.362874000	-0.701447000	-2.342767000
1	1.854243000	1.863459000	-1.481538000
1	4.985927000	-0.920500000	-0.163632000
1	5.277897000	-0.807871000	-1.893341000
1	7.216560000	0.101799000	-0.613895000
1	6.447873000	1.385018000	-1.558462000
1	6.154378000	1.271330000	0.182088000
1	-2.287629000	-1.620038000	-0.488759000
1	1.246850000	-0.978058000	0.812032000
1	-1.405864000	1.347147000	1.427504000
1	-3.015330000	-0.192370000	1.425489000
1	-3.377647000	1.113117000	0.299306000
1	-4.661497000	-1.653182000	0.270765000
1	-5.420733000	-0.175152000	0.852510000
1	-6.138648000	-0.690183000	-1.483630000
1	-5.240545000	0.822024000	-1.417276000
1	-4.089254000	-1.890198000	-2.207827000
1	-4.447600000	-0.572831000	-3.318071000
1	-2.770263000	0.862247000	-2.188516000
1	-2.037105000	-0.634668000	-2.754999000
1	-0.566505000	-0.598449000	2.572234000
1	-1.469602000	-1.933478000	1.858136000
1	1.338126000	-2.140217000	3.030837000
1	-0.135154000	-2.693232000	3.822087000
1	1.050525000	-4.581998000	2.689809000
1	-0.541166000	-4.307617000	1.991547000
1	2.098017000	-3.296300000	0.831771000
1	1.130726000	-4.619335000	0.190737000
1	-0.743271000	-3.043764000	-0.232106000
1	0.739515000	-2.518290000	-1.062784000
1	0.956893000	0.959301000	2.123518000
1	1.497442000	2.004005000	0.818177000
1	-0.384883000	2.821537000	3.082923000
1	1.314188000	3.266789000	2.958114000
1	-0.152178000	5.114742000	2.147874000
1	0.854271000	4.487378000	0.847172000
1	-2.060575000	3.737075000	1.350829000
1	-1.477938000	4.780811000	0.057160000
1	-0.156690000	2.891000000	-0.880493000
1	-1.865397000	2.470667000	-0.781350000

1_PCy3_2

6	0.185098000	0.137620000	-1.794731000
6	1.611030000	0.572062000	-1.686912000
6	2.605414000	-0.363517000	-1.471317000
8	2.484513000	-1.605169000	-1.355276000
8	3.878835000	0.186568000	-1.382493000
6	4.942695000	-0.730233000	-1.211744000
6	6.225858000	0.064368000	-1.139501000
15	-0.638371000	0.102640000	-0.187041000
6	-2.396095000	-0.358416000	-0.349564000
6	0.126678000	-1.134634000	0.879324000
6	-0.483317000	1.720792000	0.618226000
6	-3.400960000	0.793200000	-0.433376000
6	-4.821556000	0.236861000	-0.409741000
6	-5.047629000	-0.729303000	-1.565852000
6	-4.018356000	-1.851644000	-1.540722000
6	-2.601791000	-1.288968000	-1.554797000
6	-0.394096000	-1.072779000	2.321015000
6	0.431457000	-2.009460000	3.198140000
6	0.364402000	-3.439693000	2.676358000
6	0.795685000	-3.509930000	1.216462000
6	-0.033241000	-2.564389000	0.354308000
6	0.851970000	1.974923000	1.333868000
6	0.761101000	3.282744000	2.114227000
6	0.447948000	4.449200000	1.185202000
6	-0.831719000	4.189753000	0.400312000
6	-0.743243000	2.872241000	-0.362393000
1	-0.420693000	0.789414000	-2.433621000
1	0.104766000	-0.875536000	-2.201772000
1	1.862233000	1.621318000	-1.754181000
1	4.794622000	-1.315856000	-0.300255000
1	4.969111000	-1.437393000	-2.045472000
1	7.078199000	-0.603855000	-1.010166000
1	6.375280000	0.639099000	-2.054626000
1	6.202859000	0.758688000	-0.298265000
1	-2.615899000	-0.929554000	0.563337000
1	1.195726000	-0.882921000	0.858369000
1	-1.277460000	1.722317000	1.379123000
1	-3.260030000	1.505898000	0.381738000
1	-3.248535000	1.332685000	-1.374448000
1	-4.980979000	-0.288453000	0.538617000
1	-5.540681000	1.057122000	-0.449004000
1	-6.059698000	-1.136837000	-1.530343000
1	-4.953351000	-0.181906000	-2.510483000
1	-4.153230000	-2.448980000	-0.631645000

1	-4.159566000	-2.521885000	-2.390508000
1	-2.450408000	-0.707997000	-2.471154000
1	-1.869221000	-2.097029000	-1.564476000
1	-0.374462000	-0.058751000	2.722856000
1	-1.439144000	-1.402687000	2.334080000
1	1.472742000	-1.668589000	3.194183000
1	0.078790000	-1.961145000	4.230223000
1	0.982359000	-4.097896000	3.290345000
1	-0.668192000	-3.798397000	2.759678000
1	1.848428000	-3.221009000	1.125726000
1	0.707250000	-4.531154000	0.840617000
1	-1.090650000	-2.850436000	0.418229000
1	0.287721000	-2.625767000	-0.685354000
1	1.117837000	1.153984000	2.000190000
1	1.643852000	2.045614000	0.584783000
1	-0.030072000	3.195569000	2.868428000
1	1.696750000	3.459836000	2.647840000
1	0.366517000	5.379143000	1.751396000
1	1.277436000	4.571821000	0.479897000
1	-1.680645000	4.142632000	1.092409000
1	-1.027897000	5.008428000	-0.294548000
1	0.092292000	2.915742000	-1.068562000
1	-1.649177000	2.709133000	-0.946168000

1_PCy3_3

6	0.190127000	0.241154000	-2.005893000
6	0.770638000	-1.072415000	-2.419766000
6	2.039163000	-1.470218000	-2.035756000
8	2.596915000	-2.577771000	-2.180728000
8	2.764825000	-0.451066000	-1.403015000
6	3.998377000	-0.835441000	-0.826236000
6	4.521144000	0.336566000	-0.028376000
15	-0.503332000	0.292114000	-0.318714000
6	-2.277474000	-0.042286000	-0.443535000
6	0.372823000	-0.843474000	0.790401000
6	-0.290514000	1.972754000	0.331827000
6	-2.627258000	-1.262966000	-1.302739000
6	-4.139066000	-1.329035000	-1.497796000
6	-4.859380000	-1.388592000	-0.156472000
6	-4.494529000	-0.186533000	0.705904000
6	-2.985358000	-0.107112000	0.911331000
6	0.280410000	-0.489438000	2.281288000
6	1.287434000	-1.337527000	3.053676000
6	1.019295000	-2.824050000	2.857102000
6	1.012035000	-3.182914000	1.376957000
6	0.012594000	-2.324218000	0.609444000
6	1.200999000	2.304007000	0.481498000
6	1.377101000	3.697307000	1.073932000
6	0.699714000	4.741880000	0.197339000
6	-0.777607000	4.415611000	0.025644000
6	-0.973589000	3.018723000	-0.555862000
1	0.930964000	1.043328000	-2.041560000
1	-0.637629000	0.555970000	-2.650504000
1	0.122498000	-1.823064000	-2.853294000
1	3.853436000	-1.708382000	-0.181451000
1	4.706436000	-1.128511000	-1.605622000
1	5.499122000	0.101425000	0.393425000
1	4.621529000	1.221277000	-0.659508000
1	3.843076000	0.576367000	0.793853000
1	-2.651290000	0.838830000	-0.983956000
1	1.416681000	-0.712355000	0.472368000
1	-0.758860000	1.998185000	1.323580000
1	-2.120238000	-1.212383000	-2.267893000
1	-2.290647000	-2.175469000	-0.805214000
1	-4.467726000	-0.437845000	-2.044359000
1	-4.394232000	-2.195535000	-2.110347000
1	-5.939746000	-1.436249000	-0.305328000
1	-4.565067000	-2.305786000	0.366476000
1	-4.834754000	0.729689000	0.210403000
1	-4.999257000	-0.237698000	1.672326000
1	-2.647565000	-1.002828000	1.443614000
1	-2.725821000	0.757287000	1.528561000
1	0.472030000	0.567699000	2.465459000
1	-0.729936000	-0.703622000	2.643799000
1	2.295111000	-1.099549000	2.689370000
1	1.257812000	-1.077946000	4.113611000
1	1.761014000	-3.419462000	3.393033000
1	0.040291000	-3.066726000	3.286324000
1	2.009544000	-3.018714000	0.951442000
1	0.775728000	-4.239527000	1.238485000
1	-0.993788000	-2.493331000	1.009575000
1	0.009808000	-2.595949000	-0.445555000
1	1.707658000	1.560229000	1.098863000
1	1.679220000	2.271872000	-0.503393000
1	0.933078000	3.720236000	2.075768000
1	2.440317000	3.917397000	1.186360000
1	0.822240000	5.739160000	0.623879000
1	1.182737000	4.749087000	-0.786242000
1	-1.271766000	4.463188000	1.002548000
1	-1.263473000	5.152068000	-0.616923000
1	-0.534993000	2.976602000	-1.559602000
1	-2.039946000	2.809339000	-0.655192000

1_PBu3_1

6	-0.042068000	0.317351000	-1.712526000
6	1.413791000	0.446014000	-1.402874000
15	-1.012669000	0.501745000	-0.203627000
6	2.130316000	-0.701342000	-1.105787000
8	1.695305000	-1.874082000	-1.044047000
8	3.476047000	-0.481824000	-0.855963000
6	4.236314000	-1.620988000	-0.497416000
6	5.659333000	-1.168365000	-0.268169000
6	-0.590616000	2.082803000	0.529816000
6	-2.798640000	0.510664000	-0.476327000
6	-0.592772000	-0.781049000	0.967688000
6	-3.465507000	-0.863742000	-0.535886000
6	-0.760882000	3.249890000	-0.441604000
6	-1.184835000	-0.543379000	2.357543000
6	-0.473334000	4.596961000	0.214894000
6	0.961816000	4.717657000	0.708140000
6	-1.046475000	-1.777736000	3.244172000
6	0.401239000	-2.206585000	3.436756000
6	-2.984311000	-1.748232000	-1.678679000
6	-3.815506000	-3.016903000	-1.789143000
1	-0.418148000	1.066139000	-2.416598000
1	-0.269857000	-0.667987000	-2.126572000
1	1.893277000	1.416111000	-1.407965000
1	3.822371000	-2.080387000	0.405140000
1	4.187955000	-2.372016000	-1.289937000
1	6.285368000	-2.016246000	0.012603000
1	6.071885000	-0.721636000	-1.173858000
1	5.704963000	-0.427515000	0.531333000
1	-1.225037000	2.214651000	1.411517000
1	0.443254000	1.996078000	0.872544000
1	-3.233241000	1.111279000	0.327358000
1	-2.967893000	1.057004000	-1.409496000
1	-0.932836000	-1.734982000	0.553082000
1	0.498322000	-0.819156000	0.986344000
1	-3.322127000	-1.387047000	0.415218000
1	-4.542493000	-0.706326000	-0.640254000
1	-0.080700000	3.114786000	-1.288113000
1	-1.777996000	3.246445000	-0.844368000
1	-0.674765000	0.302199000	2.827964000
1	-2.242280000	-0.271989000	2.274648000
1	-0.676206000	5.386487000	-0.512001000
1	-1.168059000	4.749375000	1.046257000
1	1.175454000	5.727901000	1.058739000
1	1.164869000	4.034762000	1.534559000
1	1.665370000	4.484303000	-0.095420000
1	-1.500845000	-1.566563000	4.214770000
1	-1.618711000	-2.598117000	2.799853000
1	0.477378000	-3.032140000	4.145099000
1	0.850507000	-2.536185000	2.497840000
1	1.002179000	-1.378065000	3.820240000
1	-3.028552000	-1.183281000	-2.615240000
1	-1.934190000	-2.016723000	-1.527494000
1	-3.463327000	-3.659953000	-2.595965000
1	-3.769899000	-3.589951000	-0.860291000
1	-4.863947000	-2.778287000	-1.980639000

1_PBu3_2

6	0.392893000	-0.677491000	1.527085000
6	1.825482000	-0.245349000	1.492392000
15	-0.588531000	-0.213239000	0.071822000
6	2.572771000	-0.622692000	0.402114000
8	2.125499000	-1.250903000	-0.594902000
8	3.902492000	-0.250507000	0.415963000
6	4.665684000	-0.607223000	-0.722854000
6	6.083815000	-0.144102000	-0.485725000
6	0.084630000	1.248254000	-0.710318000
6	-0.776108000	-1.470034000	-1.203946000
6	-2.217018000	0.240481000	0.700519000
6	-0.813535000	-2.917514000	-0.723888000
6	-0.036146000	2.499678000	0.154979000
6	-3.228148000	0.675270000	-0.355148000
6	0.844552000	3.626655000	-0.366168000
6	0.706501000	4.891048000	0.466566000
6	-4.475178000	1.279299000	0.278043000
6	-5.502940000	1.688263000	-0.765089000
6	-1.971409000	-3.238620000	0.212157000
6	-2.084265000	-4.732566000	0.473172000
1	0.250396000	-1.763652000	1.623430000
1	-0.134956000	-0.224893000	2.371222000
1	2.244270000	0.321509000	2.311255000
1	4.627579000	-1.688507000	-0.877030000
1	4.247480000	-0.139452000	-1.618575000
1	6.712301000	-0.397745000	-1.340234000
1	6.117919000	0.936776000	-0.342376000
1	6.500958000	-0.621401000	0.402014000

1	1.130843000	1.012070000	-0.919163000
1	-0.424935000	1.374935000	-1.670077000
1	-1.692815000	-1.218384000	-1.746557000
1	0.069020000	-1.317453000	-1.874305000
1	-2.599831000	-0.598273000	1.287240000
1	-2.034933000	1.054427000	1.410579000
1	-0.884725000	-3.558000000	-1.607601000
1	0.137463000	-3.156650000	-0.241213000
1	-1.079396000	2.831515000	0.187477000
1	0.256187000	2.265261000	1.185094000
1	-3.516331000	-0.181239000	-0.971559000
1	-2.775935000	1.410840000	-1.029846000
1	1.883800000	3.284656000	-0.362435000
1	0.586105000	3.831547000	-1.409428000
1	1.348194000	5.690520000	0.094679000
1	-0.323392000	5.254927000	0.454287000
1	0.978623000	4.699798000	1.506874000
1	-4.183385000	2.146357000	0.878247000
1	-4.910732000	0.553574000	0.971081000
1	-5.084180000	2.425082000	-1.454104000
1	-5.821032000	0.826113000	-1.355278000
1	-6.390361000	2.125617000	-0.306530000
1	-1.833829000	-2.715204000	1.163836000
1	-2.903913000	-2.858343000	-0.219895000
1	-2.899987000	-4.964005000	1.158868000
1	-2.262171000	-5.274846000	-0.457931000
1	-1.159744000	-5.117752000	0.908791000

1_PBu3_3

6	0.274001000	-1.240589000	1.255976000
6	0.824300000	-2.322386000	0.382163000
15	-0.532060000	0.022984000	0.258034000
6	2.126882000	-2.321067000	-0.094000000
8	2.682165000	-3.135784000	-0.857596000
8	2.883885000	-1.247104000	0.382411000
6	4.230931000	-1.189876000	-0.048710000
6	4.936671000	-0.141148000	0.778906000
6	-1.278728000	1.279133000	1.300857000
6	0.652206000	0.802294000	-0.833757000
6	-1.777695000	-0.747684000	-0.768235000
6	1.542767000	1.834186000	-0.149648000
6	-2.027120000	2.385231000	0.544613000
6	-2.790416000	-1.569761000	0.023989000
6	-3.525071000	2.123933000	0.436419000
6	-4.235163000	3.221471000	-0.339411000
6	-3.962839000	-2.001552000	-0.845651000
6	-4.960108000	-2.853795000	-0.077503000
6	2.649536000	2.310647000	-1.080367000
6	3.480372000	3.421124000	-0.457380000
1	-0.480721000	-1.592426000	1.967707000
1	1.050004000	-0.734560000	1.836878000
1	0.164465000	-3.113273000	0.048653000
1	4.703925000	-2.166104000	0.071870000
1	4.273372000	-0.942647000	-1.115077000
1	5.976739000	-0.045924000	0.464158000
1	4.455167000	0.832180000	0.668722000
1	4.920639000	-0.413452000	1.835286000
1	-1.942082000	0.768354000	2.005346000
1	-0.452187000	1.680842000	1.893489000
1	0.096559000	1.252467000	-1.661411000
1	1.248675000	-0.016852000	-1.248661000
1	-2.265672000	0.052210000	-1.334875000
1	-1.245244000	-1.372345000	-1.490751000
1	0.939780000	2.691332000	0.168198000
1	1.990341000	1.401692000	0.752307000
1	-1.874523000	3.335421000	1.061714000
1	-1.606170000	2.517140000	-0.459344000
1	-2.294057000	-2.451415000	0.440743000
1	-3.168274000	-0.990169000	0.874179000
1	-3.702377000	1.155565000	-0.041486000
1	-3.939288000	2.047038000	1.445949000
1	-5.309277000	3.043294000	-0.397237000
1	-4.081084000	4.192981000	0.135343000
1	-3.850535000	3.286977000	-1.359596000
1	-4.456584000	-1.107000000	-1.239442000
1	-3.582085000	-2.553056000	-1.709957000
1	-5.801685000	-3.151389000	-0.703785000
1	-4.484632000	-3.761863000	0.298967000
1	-5.357542000	-2.307145000	0.780611000
1	2.204472000	2.657638000	-2.017781000
1	3.284678000	1.455804000	-1.335516000
1	4.294798000	3.731929000	-1.112572000
1	2.862124000	4.297627000	-0.251130000
1	3.917624000	3.096508000	0.489895000

2_PMe3_1

6	-1.897065000	2.321977000	0.013545000
6	-1.045523000	1.263055000	-0.006297000
6	0.376992000	1.346493000	-0.029074000
15	-1.829242000	-0.355719000	-0.007264000
6	1.222543000	0.234492000	0.005109000
8	0.881668000	-0.964710000	0.050089000
8	2.551970000	0.550874000	-0.015855000
6	3.461524000	-0.541136000	0.025413000
6	4.859469000	0.027139000	-0.006876000
6	-3.605984000	-0.153706000	-0.097851000
6	-1.421851000	-1.320063000	-1.462145000
6	-1.605641000	-1.223456000	1.537159000
1	-2.971113000	2.221669000	0.010509000
1	-1.497688000	3.330266000	0.027984000
1	0.825504000	2.332115000	-0.059928000
1	3.294251000	-1.125823000	0.932855000
1	3.284224000	-1.201697000	-0.826235000
1	5.591949000	-0.779933000	0.025126000
1	5.021328000	0.602711000	-0.918915000
1	5.030429000	0.681011000	0.849000000
1	-3.891471000	0.373714000	-1.006627000
1	-4.032338000	-1.158333000	-0.124414000
1	-3.989860000	0.369314000	0.776204000
1	-1.149186000	-2.331984000	-1.169819000
1	-2.291103000	-1.344178000	-2.119447000
1	-0.577772000	-0.867123000	-1.975769000
1	-0.552616000	-1.369322000	1.752612000
1	-2.078611000	-0.627863000	2.319315000
1	-2.113182000	-2.186846000	1.468731000

2_PMe3_2

6	-1.951309000	2.310669000	0.000493000
6	-1.069717000	1.277892000	0.000178000
6	0.351289000	1.388385000	-0.000211000
15	-1.787165000	-0.371969000	-0.000049000
6	1.190277000	0.272487000	0.000027000
8	0.828307000	-0.923216000	0.000442000
8	2.522328000	0.568062000	-0.000237000
6	3.415637000	-0.538875000	0.000102000
6	4.821772000	0.009437000	0.000049000
6	-3.572974000	-0.226462000	-0.000415000
6	-1.438587000	-1.265361000	-1.515699000
6	-1.439400000	-1.265866000	1.515481000
1	-3.021869000	2.176480000	0.000597000
1	-1.583473000	3.330950000	0.000573000
1	0.794162000	2.376637000	-0.000475000
1	3.233386000	-1.158806000	0.880705000
1	3.233498000	-1.159259000	-0.880210000
1	4.997317000	0.621977000	-0.884958000
1	4.997188000	0.622449000	0.884756000
1	5.542055000	-0.809119000	0.000320000
1	-3.925262000	0.286517000	-0.893753000
1	-3.965901000	-1.245554000	-0.000657000
1	-3.925634000	0.286192000	0.892965000
1	-1.185642000	-2.297991000	-1.283312000
1	-2.323923000	-1.235598000	-2.150927000
1	-0.596460000	-0.806224000	-2.026491000
1	-0.597339000	-0.807159000	2.026752000
1	-2.324987000	-1.236077000	2.150354000
1	-1.186638000	-2.298486000	1.282830000

2_PMe3_3

6	2.042669000	2.205324000	-0.351331000
6	1.081712000	1.270662000	-0.136148000
6	-0.317951000	1.517371000	-0.024794000
15	1.652113000	-0.429990000	0.001243000
6	-1.239461000	0.502635000	0.237031000
8	-0.974332000	-0.706978000	0.403659000
8	-2.535125000	0.929317000	0.316740000
6	-3.534289000	-0.073276000	0.464793000
6	-3.877176000	-0.710228000	-0.864600000
6	1.023278000	-1.461417000	-1.324775000
6	3.426925000	-0.467041000	-0.244355000
6	1.448256000	-1.095579000	1.654184000
1	3.091046000	1.967545000	-0.442286000
1	1.765329000	3.249805000	-0.442957000
1	-0.675652000	2.533147000	-0.138738000
1	-3.197456000	-0.824385000	1.179672000
1	-4.399777000	0.440977000	0.882269000
1	-4.215223000	0.045449000	-1.575016000
1	-3.004193000	-1.215831000	-1.278174000
1	-4.674542000	-1.444318000	-0.738361000
1	0.723682000	-2.429332000	-0.927689000
1	0.157388000	-0.986584000	-1.779064000
1	1.809940000	-1.593866000	-2.067625000
1	3.694291000	-0.101227000	-1.234324000
1	3.941782000	0.108906000	0.522763000
1	3.729528000	-1.513142000	-0.160225000
1	1.110327000	-2.128571000	1.593437000
1	2.408324000	-1.051131000	2.168396000
1	0.705392000	-0.517013000	2.196045000

2_PPhMe2_1

6	-2.379448000	1.934110000	-1.573316000
6	-1.330033000	1.573521000	-0.791342000

6	0.002127000	2.068675000	-0.888656000
15	-1.649947000	0.245717000	0.386885000
6	0.977142000	1.681193000	0.028323000
8	0.769218000	0.978851000	1.040962000
8	2.238937000	2.123869000	-0.241963000
6	3.289450000	1.509883000	0.498840000
6	3.574746000	0.114139000	-0.015173000
6	-3.296776000	-0.395955000	0.071055000
6	-0.592866000	-1.156988000	0.036295000
6	-1.714946000	0.728665000	2.111638000
6	-0.197292000	-1.366820000	-1.283223000
6	0.593114000	-2.465941000	-1.600511000
6	0.980872000	-3.354893000	-0.605488000
6	0.573049000	-3.150776000	0.709477000
6	-0.213787000	-2.053613000	1.032735000
1	-3.355242000	1.479998000	-1.504810000
1	-2.251073000	2.714844000	-2.314924000
1	0.265058000	2.717731000	-1.713973000
1	4.153947000	2.159086000	0.363536000
1	3.031446000	1.489716000	1.558026000
1	3.837014000	0.143226000	-1.073821000
1	4.406789000	-0.329542000	0.533729000
1	2.696892000	-0.523448000	0.111444000
1	-3.444463000	-1.225315000	0.765470000
1	-4.054655000	0.362373000	0.266029000
1	-3.385687000	-0.766052000	-0.948924000
1	-1.511131000	1.795936000	2.175938000
1	-0.952956000	0.207131000	2.683667000
1	-2.706259000	0.514891000	2.509609000
1	-0.499023000	-0.670829000	-2.057814000
1	0.907413000	-2.623036000	-2.624090000
1	1.600009000	-4.207519000	-0.852991000
1	0.872651000	-3.843479000	1.485097000
1	-0.518876000	-1.894432000	2.059609000

2_PPhMe2_2

6	-0.561468000	2.841394000	-1.638159000
6	-0.004153000	1.895854000	-0.837031000
6	1.339177000	1.429245000	-0.897928000
15	-1.101542000	1.187489000	0.398743000
6	1.806455000	0.392662000	-0.085686000
8	1.145955000	-0.215882000	0.780289000
8	3.110426000	0.054823000	-0.296509000
6	3.629401000	-0.995967000	0.510485000
6	5.070230000	-1.206036000	0.113531000
6	-2.694270000	2.006703000	0.308147000
6	-1.509652000	-0.515578000	0.033192000
6	-0.568495000	1.474683000	2.085005000
6	-1.257761000	-1.030744000	-1.235730000
6	-1.645848000	-2.329624000	-1.538996000
6	-2.284123000	-3.109352000	-0.580875000
6	-2.540327000	-2.590325000	0.683695000
6	-2.157171000	-1.291567000	0.991779000
1	-1.583460000	3.172745000	-1.543419000
1	0.037896000	3.308459000	-2.411906000
1	2.009611000	1.873062000	-1.623273000
1	3.545308000	-0.725462000	1.565361000
1	3.040170000	-1.902524000	0.356727000
1	5.144279000	-1.480444000	-0.939296000
1	5.651672000	-0.297775000	0.275611000
1	5.509356000	-2.006563000	0.709445000
1	-2.595800000	3.071666000	0.515316000
1	-3.163929000	1.848901000	-0.661155000
1	-3.318256000	1.551080000	1.080222000
1	-1.228587000	2.209764000	2.544545000
1	0.454578000	1.844114000	2.073077000
1	-0.591189000	0.544936000	2.649015000
1	-0.750357000	-0.423897000	-1.976664000
1	-1.444835000	-2.733857000	-2.522554000
1	-2.581554000	-4.122369000	-0.819236000
1	-3.037397000	-3.196009000	1.430189000
1	-2.357387000	-0.887779000	1.978015000

2_PPhMe2_3

6	0.235120000	-2.913647000	-1.552513000
6	-0.260012000	-1.844938000	-0.875696000
6	-1.497194000	-1.188770000	-1.132993000
15	0.766405000	-1.242001000	0.472040000
6	-1.873423000	-0.021770000	-0.464210000
8	-1.210848000	0.560557000	0.418442000
8	-3.078981000	0.485579000	-0.856330000
6	-3.572049000	1.605318000	-0.128421000
6	-4.232955000	1.174013000	1.162888000
6	2.167652000	-2.342314000	0.677010000
6	1.546051000	0.321404000	0.085551000
6	-0.045892000	-1.284250000	2.069483000
6	1.492276000	0.829705000	-1.209585000
6	2.165024000	2.005818000	-1.513995000
6	2.886714000	2.672245000	-0.529374000
6	2.941739000	2.161317000	0.762666000
6	2.275952000	0.981915000	1.071299000
1	1.165261000	-3.397756000	-1.300201000
1	-0.325130000	-3.326971000	-2.383949000
1	-2.147787000	-1.590702000	-1.899550000
1	-2.757602000	2.303462000	0.065906000
1	-4.292302000	2.085578000	-0.790435000
1	-4.641797000	2.039683000	1.686307000
1	-5.048090000	0.477525000	0.961698000
1	-3.507389000	0.686971000	1.814942000
1	2.796689000	-2.349656000	-0.211249000
1	2.747980000	-1.950169000	1.514789000
1	1.835770000	-3.351627000	0.917532000
1	0.386579000	-2.086688000	2.666423000
1	-1.109638000	-1.458499000	1.921112000
1	0.080781000	-0.329377000	2.575318000
1	0.914450000	0.315820000	-1.969069000
1	2.119916000	2.405202000	-2.518773000
1	3.406072000	3.591178000	-0.768970000
1	3.502802000	2.679120000	1.529639000
1	2.321989000	0.581562000	2.078365000

2_PPh2Me_1

6	-1.069043000	-2.232140000	-1.577921000
6	-0.087618000	-1.707942000	-0.795668000
6	1.303363000	-1.979851000	-0.930723000
15	-0.645648000	-0.500831000	0.425368000
6	2.277731000	-1.466890000	-0.070970000
8	2.075167000	-0.785524000	0.951786000
8	3.558854000	-1.776544000	-0.436533000
6	4.597495000	-1.129354000	0.290117000
6	4.787795000	0.297963000	-0.178176000
6	0.157349000	1.071792000	0.163086000
6	-2.403737000	-0.217796000	0.154577000
6	-0.548111000	-1.033194000	2.129678000
6	-2.841931000	0.793782000	-0.699998000
6	-4.200349000	0.978404000	-0.922325000
6	-5.127357000	0.153498000	-0.296094000
6	-4.696373000	-0.857619000	0.555422000
6	-3.339368000	-1.043807000	0.781903000
6	0.594020000	1.407157000	-1.117189000
6	1.205796000	2.635868000	-1.334017000
6	1.379051000	3.522863000	-0.277493000
6	0.935443000	3.187720000	0.998107000
6	0.322702000	1.962399000	1.221498000
1	-2.108645000	-1.957795000	-1.494100000
1	-0.807544000	-2.960938000	-2.337445000
1	1.619265000	-2.587281000	-1.769984000
1	4.375468000	-1.161316000	1.357241000
1	5.492921000	-1.721792000	0.103080000
1	5.615086000	0.766060000	0.357613000
1	5.011253000	0.323003000	-1.245761000
1	3.882707000	0.879823000	0.005360000
1	-0.864249000	-2.075433000	2.180307000
1	0.475103000	-0.949807000	2.479503000
1	-1.224340000	-0.425897000	2.731299000
1	-2.125558000	1.439523000	-1.192074000
1	-4.533059000	1.767320000	-1.583926000
1	-6.185637000	0.299545000	-0.469882000
1	-5.415526000	-1.502234000	1.043416000
1	-3.012641000	-1.838687000	1.440569000
1	0.461694000	0.708646000	-1.935496000
1	1.550645000	2.896740000	-2.325971000
1	1.861487000	4.476806000	-0.447219000
1	1.072166000	3.878332000	1.819896000
1	-0.009195000	1.697287000	2.218141000

2_PPh2Me_2

6	0.676192000	-1.943965000	-1.876766000
6	-0.204530000	-1.425680000	-0.980033000
6	-1.617516000	-1.591237000	-1.016655000
15	0.521449000	-0.427786000	0.337232000
6	-2.478903000	-0.911408000	-0.151943000
8	-2.139531000	-0.119647000	0.749208000
8	-3.801547000	-1.173698000	-0.355150000
6	-4.718174000	-0.501296000	0.500461000
6	-6.109829000	-0.929884000	0.103788000
6	2.310598000	-0.594887000	0.199268000
6	0.200312000	1.312213000	0.096689000
6	0.131203000	-0.983461000	1.999603000
6	-0.274587000	1.771055000	-1.128710000
6	-0.459490000	3.134024000	-1.324506000
6	-0.168793000	4.030918000	-0.302551000
6	0.311286000	3.569370000	0.918852000
6	0.500628000	2.208797000	1.120033000
6	3.085602000	0.374593000	-0.434602000
6	4.457687000	0.191381000	-0.567283000
6	5.057545000	-0.959654000	-0.073464000
6	4.287058000	-1.933945000	0.554888000
6	2.918999000	-1.753047000	0.691175000
1	1.744688000	-1.816730000	-1.804312000

1	0.300835000	-2.527509000	-2.710435000
1	-2.039174000	-2.230862000	-1.782029000
1	-4.506772000	-0.758394000	1.540735000
1	-4.592439000	0.578708000	0.397726000
1	-6.847804000	-0.436618000	0.736938000
1	-6.314236000	-0.664351000	-0.933971000
1	-6.227606000	-2.008437000	0.214056000
1	-0.470995000	-1.889002000	1.933154000
1	1.047533000	-1.182215000	2.551692000
1	-0.456110000	-0.218837000	2.499606000
1	-0.506891000	1.065898000	-1.918272000
1	-0.834198000	3.494355000	-2.273558000
1	-0.316953000	5.091846000	-0.457512000
1	0.538698000	4.267844000	1.713430000
1	0.880835000	1.848011000	2.069098000
1	2.623120000	1.271715000	-0.826727000
1	5.054674000	0.949156000	-1.057414000
1	6.125702000	-1.100271000	-0.177260000
1	4.751929000	-2.833478000	0.936375000
1	2.320332000	-2.520616000	1.168274000

2_PPh2Me_3

6	0.405069000	-0.212785000	3.417464000
6	-0.062488000	0.064705000	2.171639000
6	-1.239884000	0.802995000	1.866317000
15	0.895396000	-0.643732000	0.820022000
6	-1.585468000	1.136307000	0.554746000
8	-0.921321000	0.860298000	-0.464018000
8	-2.753738000	1.826631000	0.432171000
6	-3.135138000	2.192061000	-0.889389000
6	-4.434462000	2.953783000	-0.794453000
6	-0.076425000	-1.770185000	-0.177961000
6	1.861554000	0.548519000	-0.096514000
6	2.145802000	-1.726507000	1.522077000
6	1.757029000	1.905767000	0.201462000
6	2.577064000	2.817154000	-0.447465000
6	3.488614000	2.378651000	-1.403202000
6	3.589230000	1.025911000	-1.704807000
6	2.783424000	0.106331000	-1.044484000
6	-0.793277000	-2.750365000	0.505884000
6	-1.526433000	-3.692462000	-0.205700000
6	-1.530555000	-3.660833000	-1.594823000
6	-0.813508000	-2.680779000	-2.275154000
6	-0.092186000	-1.728315000	-1.569565000
1	1.281437000	-0.811134000	3.607383000
1	-0.122773000	0.174354000	4.281997000
1	-1.872990000	1.129211000	2.681649000
1	-2.350546000	2.801961000	-1.342472000
1	-3.246468000	1.293999000	-1.500876000
1	-5.212512000	2.335331000	-0.345708000
1	-4.312415000	3.850878000	-0.186485000
1	-4.764858000	3.254530000	-1.789031000
1	1.681389000	-2.507467000	2.122791000
1	2.673161000	-2.193055000	0.689049000
1	2.858807000	-1.160611000	2.119726000
1	1.029638000	2.240147000	0.931932000
1	2.499732000	3.870832000	-0.213577000
1	4.121622000	3.093549000	-1.912935000
1	4.296998000	0.683767000	-2.448302000
1	2.870332000	-0.949766000	-1.273542000
1	-0.785957000	-2.769889000	1.591207000
1	-2.092399000	-4.446908000	0.324753000
1	-2.098591000	-4.396371000	-2.149777000
1	-0.827540000	-2.650587000	-3.356863000
1	0.435702000	-0.941951000	-2.092894000

2_PPh3_1

6	0.831064000	-0.262643000	2.982913000
6	-0.080677000	0.061990000	2.029849000
6	-1.458562000	0.344875000	2.255199000
15	0.501958000	-0.024656000	0.321274000
6	-2.310806000	0.727605000	1.218086000
8	-1.955748000	0.973800000	0.048304000
8	-3.627516000	0.824748000	1.563667000
6	-4.548444000	0.953103000	0.484252000
6	-4.747786000	-0.371613000	-0.221760000
6	2.170972000	-0.712746000	0.335050000
6	-0.492426000	-1.196877000	-0.597666000
6	0.750017000	1.572741000	-0.447049000
6	-0.745412000	-1.049989000	-1.959310000
6	-1.497936000	-2.007430000	-2.624938000
6	-2.000951000	-3.106328000	-1.934145000
6	-1.751513000	-3.249298000	-0.574874000
6	-0.995687000	-2.295423000	0.097813000
6	1.467115000	1.652305000	-1.640389000
6	1.718222000	2.892614000	-2.213208000
6	1.282729000	4.049626000	-1.577680000
6	0.590199000	3.970036000	-0.373315000
6	0.313705000	2.732518000	0.189801000
6	3.238152000	0.123078000	0.681779000
6	4.530082000	-0.377774000	0.733302000
6	4.770428000	-1.714450000	0.429177000
6	3.716578000	-2.547403000	0.077186000
6	2.418621000	-2.050736000	0.031957000
1	1.844366000	-0.557210000	2.761456000
1	0.534605000	-0.246876000	4.025960000
1	-1.862729000	0.187457000	3.247110000
1	-4.196430000	1.715154000	-0.211706000
1	-5.477391000	1.297480000	0.937758000
1	-3.807265000	-0.711082000	-0.660979000
1	-5.101447000	-1.128903000	0.479666000
1	-5.485623000	-0.269242000	-1.019054000
1	-0.379938000	-0.181884000	-2.492894000
1	-1.700176000	-1.890778000	-3.681617000
1	-2.591702000	-3.847958000	-2.456215000
1	-2.145547000	-4.099960000	-0.034456000
1	-0.796460000	-2.404543000	1.157966000
1	1.836956000	0.750109000	-2.114542000
1	2.263435000	2.954158000	-3.145750000
1	1.488324000	5.016594000	-2.018604000
1	0.259220000	4.872237000	0.124007000
1	-0.240732000	2.658908000	1.117613000
1	3.053891000	1.164920000	0.916813000
1	5.348956000	0.274593000	1.006529000
1	5.779968000	-2.103239000	0.463156000
1	3.900567000	-3.585862000	-0.164899000
1	1.605777000	-2.709311000	-0.244357000

2_PPh3_2

6	-0.767139000	0.110972000	-2.978188000
6	0.155349000	0.154287000	-1.980999000
6	1.554893000	0.351711000	-2.153637000
15	-0.504007000	0.024235000	-0.305249000
6	2.450465000	0.363804000	-1.081872000
8	2.153966000	0.180453000	0.114687000
8	3.748186000	0.600368000	-1.434803000
6	4.724534000	0.438289000	-0.410693000
6	5.050555000	-1.023599000	-0.191846000
6	-0.166824000	1.525468000	0.609225000
6	-2.302745000	-0.070788000	-0.419066000
6	-0.081628000	-1.506512000	0.522750000
6	-2.890656000	-1.290644000	-0.770129000
6	-4.267376000	-1.389941000	-0.900485000
6	-5.069813000	-0.275352000	-0.673701000
6	-4.492456000	0.936832000	-0.319805000
6	-3.111537000	1.042023000	-0.194199000
6	0.846335000	-2.381668000	-0.038823000
6	1.103976000	-3.598363000	0.576446000
6	0.449446000	-3.936255000	1.756941000
6	-0.480247000	-3.066617000	2.314803000
6	-0.759005000	-1.857433000	1.690606000
6	-0.107360000	2.717713000	-0.110292000
6	0.104624000	3.916476000	0.560467000
6	0.253781000	3.923452000	1.941848000
6	0.193925000	2.730976000	2.656557000
6	-0.015330000	1.530022000	1.993404000
1	-1.825610000	-0.000203000	-2.805957000
1	-0.437559000	0.202223000	-4.007382000
1	1.929552000	0.511912000	-3.156852000
1	4.370059000	0.900369000	0.510824000
1	5.601182000	0.983868000	-0.758940000
1	4.163095000	-1.562647000	0.143362000
1	5.410202000	-1.477798000	-1.116211000
1	5.826272000	-1.129582000	0.568068000
1	-2.267484000	-2.159510000	-0.946870000
1	-4.714538000	-2.336035000	-1.175702000
1	-6.144902000	-0.355135000	-0.770314000
1	-5.113721000	1.803999000	-0.138508000
1	-2.671796000	1.991337000	0.082338000
1	1.361374000	-2.106224000	-0.951354000
1	1.819368000	-4.280964000	0.136809000
1	0.659350000	-4.882577000	2.238698000
1	-0.997764000	-3.332352000	3.227106000
1	-1.510043000	-1.194929000	2.106020000
1	-0.227159000	2.708645000	-1.187843000
1	0.155453000	4.841773000	0.001765000
1	0.421769000	4.857262000	2.462711000
1	0.319128000	2.734914000	3.731425000
1	-0.037955000	0.602443000	2.550621000

2_PPh3_3

6	0.663983000	-0.159494000	-2.973155000
6	-0.206309000	-0.035007000	-1.936118000
6	-1.624931000	0.006264000	-2.044751000
15	0.542263000	-0.021143000	-0.293274000
6	-2.464974000	0.170460000	-0.939470000
8	-2.098204000	0.351286000	0.237325000
8	-3.795931000	0.124664000	-1.232646000
6	-4.688299000	0.294952000	-0.137230000
6	-6.094911000	0.222701000	-0.679175000

6	-0.046741000	-1.413966000	0.663209000
6	2.317012000	-0.287402000	-0.483341000
6	0.467023000	1.571333000	0.521047000
6	3.121062000	0.788576000	-0.874089000
6	4.482633000	0.609087000	-1.065046000
6	5.054664000	-0.643099000	-0.859987000
6	4.262522000	-1.713570000	-0.466478000
6	2.895738000	-1.539098000	-0.279825000
6	-0.295621000	2.604178000	-0.020098000
6	-0.274505000	3.857982000	0.574109000
6	0.491823000	4.077309000	1.714324000
6	1.255364000	3.048199000	2.253214000
6	1.256887000	1.797969000	1.648595000
6	-0.134958000	-1.371330000	2.052118000
6	-0.547874000	-2.498840000	2.747935000
6	-0.873261000	-3.664377000	2.060901000
6	-0.786478000	-3.704216000	0.674694000
6	-0.371804000	-2.579545000	-0.028828000
1	1.731623000	-0.242526000	-2.848163000
1	0.278111000	-0.190730000	-3.986235000
1	-2.065495000	-0.123899000	-3.025388000
1	-4.498698000	1.256444000	0.344972000
1	-4.509849000	-0.485007000	0.606163000
1	-6.265927000	1.008704000	-1.415570000
1	-6.276230000	-0.741798000	-1.154675000
1	-6.814229000	0.347607000	0.130617000
1	2.677870000	1.764537000	-1.034271000
1	5.097255000	1.445510000	-1.370698000
1	6.118424000	-0.781394000	-1.004312000
1	4.705056000	-2.687256000	-0.302340000
1	2.287498000	-2.379845000	0.026856000
1	-0.899653000	2.421069000	-0.900380000
1	-0.860475000	4.662681000	0.149961000
1	0.499324000	5.054645000	2.179485000
1	1.858981000	3.220326000	3.134520000
1	1.880588000	1.005176000	2.045767000
1	0.093648000	-0.459308000	2.588253000
1	-0.623854000	-2.464908000	3.826874000
1	-1.198819000	-4.540100000	2.607387000
1	-1.043639000	-4.607941000	0.137913000
1	-0.300640000	-2.606850000	-1.110397000

2_Pani3_1

6	-0.675410000	-0.064040000	-3.131281000
6	0.302029000	-0.006717000	-2.187003000
6	1.700185000	-0.074415000	-2.447535000
15	-0.285855000	0.062452000	-0.480554000
6	2.687902000	0.015668000	-1.459098000
8	2.512360000	0.224677000	-0.246405000
8	3.959174000	-0.144190000	-1.938721000
6	5.006455000	-0.057995000	-0.981089000
6	6.312352000	-0.243274000	-1.715048000
6	0.310719000	-1.362576000	0.408604000
6	0.018181000	1.637417000	0.295702000
6	-2.075562000	-0.065020000	-0.478346000
6	-0.630951000	1.957392000	1.494382000
6	-0.448374000	3.193589000	2.080309000
6	0.368311000	4.146612000	1.462239000
6	0.996998000	3.846293000	0.252788000
6	0.823472000	2.592305000	-0.315925000
6	-2.728155000	-1.263818000	-0.208793000
6	-4.115022000	-1.354002000	-0.252294000
6	-4.866519000	-0.225855000	-0.576947000
6	-4.220730000	0.987182000	-0.847821000
6	-2.845959000	1.064495000	-0.795305000
6	0.563477000	-1.332301000	1.781766000
6	0.982780000	-2.472371000	2.435897000
6	1.162695000	-3.665598000	1.726794000
6	0.915378000	-3.703303000	0.355512000
6	0.490162000	-2.549111000	-0.293682000
8	1.580176000	-4.727242000	2.444276000
8	-6.209393000	-0.212565000	-0.647042000
8	0.486966000	5.328802000	2.094441000
6	1.799806000	-5.948515000	1.762580000
6	-6.906625000	-1.418442000	-0.388401000
6	1.305054000	6.324327000	1.505767000
1	-1.728004000	-0.066865000	-2.896662000
1	-0.398559000	-0.121965000	-4.178480000
1	2.011620000	-0.235124000	-3.472756000
1	4.968796000	0.911532000	-0.479280000
1	4.870693000	-0.826318000	-0.216592000
1	6.343319000	-1.215431000	-2.208342000
1	6.443543000	0.531666000	-2.471168000
1	7.146649000	-0.185894000	-1.015345000
1	-1.290353000	1.237434000	1.966087000
1	-0.937625000	3.450041000	3.010781000
1	1.621622000	4.573514000	-0.245280000
1	1.321177000	2.355276000	-1.248397000
1	-2.157792000	-2.148505000	0.044170000
1	-4.593341000	-2.297401000	-0.032121000
1	-4.821463000	1.852898000	-1.094181000
1	-2.357233000	2.008188000	-1.008286000
1	0.454040000	-0.409616000	2.337800000
1	1.191044000	-2.462705000	3.497814000
1	1.054680000	-4.611913000	-0.212116000
1	0.299522000	-2.580737000	-1.360808000
1	0.878582000	-6.315466000	1.304368000
1	2.567504000	-5.836438000	0.993717000
1	2.139096000	-6.661874000	2.508497000
1	-6.620423000	-2.197212000	-1.098440000
1	-6.721779000	-1.768247000	0.629545000
1	-7.963038000	-1.194208000	-0.505945000
1	2.337790000	5.980881000	1.415598000
1	0.929513000	6.609746000	0.520795000
1	1.268086000	7.184528000	2.168310000

2_Pani3_2

6	-0.629752000	-0.187534000	-3.269219000
6	0.343250000	-0.017206000	-2.334067000
6	1.738798000	0.061635000	-2.604539000
15	-0.234692000	0.009240000	-0.623118000
6	2.713691000	0.276805000	-1.623195000
8	2.517567000	0.484233000	-0.412973000
8	3.993427000	0.252395000	-2.104115000
6	5.025510000	0.475465000	-1.151864000
6	6.343316000	0.421439000	-1.885856000
6	0.514678000	-1.333504000	0.279287000
6	-0.099099000	1.617600000	0.133458000
6	-2.000614000	-0.312018000	-0.604223000
6	-0.771438000	1.872341000	1.325800000
6	-0.733507000	3.130092000	1.914758000
6	-0.033925000	4.158207000	1.282339000
6	0.621147000	3.914946000	0.069582000
6	0.596227000	2.656454000	-0.493439000
6	-2.517632000	-1.570451000	-0.312968000
6	-3.887303000	-1.809503000	-0.336991000
6	-4.759789000	-0.772614000	-0.662971000
6	-4.251066000	0.498412000	-0.958653000
6	-2.891927000	0.723226000	-0.926006000
6	0.783904000	-1.252692000	1.639513000
6	1.320859000	-2.335876000	2.322878000
6	1.594422000	-3.517860000	1.632913000
6	1.331499000	-3.601521000	0.262648000
6	0.797027000	-2.518005000	-0.407823000
8	2.113327000	-4.619628000	2.210234000
8	-6.097461000	-0.903436000	-0.711111000
8	0.052917000	5.411985000	1.764037000
6	2.401944000	-4.576084000	3.595559000
6	-6.657554000	-2.172366000	-0.422140000
6	-0.587539000	5.703950000	2.992922000
1	-1.673992000	-0.301087000	-3.025164000
1	-0.356399000	-0.226994000	-4.318167000
1	2.060712000	-0.077706000	-3.629558000
1	4.879824000	1.445421000	-0.670966000
1	4.976684000	-0.286620000	-0.370821000
1	6.482142000	-0.551649000	-2.358265000
1	6.386790000	1.189777000	-2.658623000
1	7.166422000	0.586525000	-1.190024000
1	-1.344467000	1.086067000	1.804585000
1	-1.256188000	3.298854000	2.845055000
1	1.148048000	4.730469000	-0.408227000
1	1.113291000	2.469688000	-1.426576000
1	-1.853607000	-2.386327000	-0.057482000
1	-4.258339000	-2.796203000	-0.100221000
1	-4.943819000	1.291687000	-1.207187000
1	-2.510590000	1.711029000	-1.156830000
1	0.597550000	-0.332311000	2.178839000
1	1.530268000	-2.247198000	3.379077000
1	1.558196000	-4.523550000	-0.256562000
1	0.595509000	-2.589429000	-1.470991000
1	3.144080000	-3.806639000	3.819014000
1	1.498413000	-4.389621000	4.180561000
1	2.804824000	-5.550534000	3.857345000
1	-6.302804000	-2.927529000	-1.126704000
1	-6.417961000	-2.484162000	0.596660000
1	-7.733821000	-2.063763000	-0.521883000
1	-1.665578000	5.543692000	2.922355000
1	-0.181455000	5.092842000	3.801909000
1	-0.392334000	6.752247000	3.200848000

2_Pani3_3

6	0.793089000	-0.354162000	3.150646000
6	-0.135433000	0.120279000	2.278537000
6	-1.437112000	0.582666000	2.626938000
15	0.315313000	0.005826000	0.531224000
6	-2.328980000	1.128991000	1.698371000
8	-2.078436000	1.402329000	0.511324000
8	-3.581014000	1.365217000	2.200517000
6	-4.582183000	1.700831000	1.246295000

6	-5.046020000	0.476020000	0.485497000
6	-0.924620000	-0.938612000	-0.335162000
6	0.722376000	1.583275000	-0.189897000
6	1.844496000	-0.923840000	0.391159000
6	1.376942000	1.633744000	-1.425948000
6	1.742116000	2.846470000	-1.974763000
6	1.481874000	4.033503000	-1.281950000
6	0.853817000	3.990667000	-0.035984000
6	0.471153000	2.767148000	0.495486000
6	1.855899000	-2.278326000	0.072062000
6	3.046439000	-2.993106000	0.006947000
6	4.252213000	-2.344608000	0.269566000
6	4.253758000	-0.981870000	0.591834000
6	3.066698000	-0.283582000	0.647532000
6	-1.265005000	-0.679718000	-1.664785000
6	-2.227051000	-1.441057000	-2.296182000
6	-2.873834000	-2.473322000	-1.606121000
6	-2.540776000	-2.737594000	-0.278625000
6	-1.567461000	-1.965408000	0.347670000
8	-3.807872000	-3.156746000	-2.296874000
8	5.453656000	-2.947752000	0.229043000
8	1.875189000	5.171783000	-1.882919000
6	-4.512551000	-4.185843000	-1.627312000
6	5.501263000	-4.324959000	-0.100121000
6	1.624786000	6.399686000	-1.222497000
1	1.733312000	-0.782224000	2.840425000
1	0.586076000	-0.325739000	4.214983000
1	-1.769512000	0.440768000	3.648038000
1	-5.399101000	2.132494000	1.824246000
1	-4.200405000	2.459314000	0.561934000
1	-4.217351000	0.049146000	-0.083155000
1	-5.426187000	-0.280285000	1.174376000
1	-5.844129000	0.740453000	-0.210066000
1	1.608268000	0.717438000	-1.957444000
1	2.240025000	2.900226000	-2.933910000
1	0.654616000	4.896839000	0.517384000
1	-0.031307000	2.733851000	1.454671000
1	0.928274000	-2.795851000	-0.136332000
1	3.022469000	-4.042443000	-0.249236000
1	5.199638000	-0.494591000	0.789273000
1	3.081237000	0.770858000	0.897132000
1	-0.795461000	0.136332000	-2.199868000
1	-2.508756000	-1.246267000	-3.322777000
1	-3.031164000	-3.525763000	0.274186000
1	-1.314184000	-2.169197000	1.382235000
1	-3.835897000	-4.978204000	-1.299106000
1	-5.056376000	-3.792734000	-0.765188000
1	-5.220332000	-4.592451000	-2.344310000
1	4.949593000	-4.922021000	0.629124000
1	5.095955000	-4.503910000	-1.098366000
1	6.549227000	-4.610348000	-0.080329000
1	0.554580000	6.549863000	-1.064847000
1	2.143522000	6.442815000	-0.262426000
1	2.006126000	7.181970000	-1.872874000

2_Ppfp3_1

6	-0.801946000	-0.380533000	3.085220000
6	0.127487000	0.043392000	2.189660000
6	1.464778000	0.428809000	2.493295000
15	-0.365483000	-0.037328000	0.453958000
6	2.337059000	0.911062000	1.515224000
8	2.025843000	1.167533000	0.335217000
8	3.620746000	1.099007000	1.936577000
6	4.584314000	1.349732000	0.917300000
6	4.938202000	0.076810000	0.177589000
6	-0.701094000	1.559318000	-0.276277000
6	0.769385000	-1.088357000	-0.444594000
6	-1.966504000	-0.860158000	0.358846000
6	1.341347000	-2.156845000	0.245117000
6	2.219528000	-3.015851000	-0.403324000
6	2.502711000	-2.778252000	-1.734670000
6	1.951327000	-1.724611000	-2.443613000
6	1.077928000	-0.872549000	-1.785752000
6	-2.080568000	-2.205361000	0.009180000
6	-3.323918000	-2.821895000	-0.030682000
6	-4.438959000	-2.071592000	0.288682000
6	-4.366580000	-0.734759000	0.641207000
6	-3.120437000	-0.131437000	0.669951000
6	-0.367977000	2.727541000	0.407899000
6	-0.701439000	3.962811000	-0.123310000
6	-1.349079000	3.999201000	-1.346115000
6	-1.694320000	2.859904000	-2.049258000
6	-1.377278000	1.628502000	-1.494252000
9	-1.663409000	5.193765000	-1.868548000
9	-5.642824000	-2.659448000	0.250134000
9	3.351689000	-3.602085000	-2.368869000
1	-1.774847000	-0.750004000	2.803212000
1	-0.561620000	-0.370896000	4.142591000
1	1.828527000	0.275103000	3.501306000
1	4.204903000	2.106303000	0.229888000
1	5.453056000	1.753949000	1.435890000
1	4.056698000	-0.324143000	-0.327262000
1	5.706410000	0.274174000	-0.571599000
1	5.318498000	-0.674966000	0.870889000
1	1.102648000	-2.321194000	1.289505000
1	2.679987000	-3.849698000	0.108264000
1	2.214176000	-1.577116000	-3.482081000
1	0.659308000	-0.027620000	-2.316837000
1	-1.201069000	-2.783712000	-0.240403000
1	-3.432394000	-3.862051000	-0.305241000
1	-5.268670000	-0.188911000	0.880944000
1	-3.045213000	0.914297000	0.942936000
1	0.153382000	2.666445000	1.355102000
1	-0.462315000	4.884939000	0.388237000
1	-2.208313000	2.942686000	-2.996760000
1	-1.666513000	0.720640000	-2.010683000

2_Ppfp3_2

6	0.657584000	-0.199197000	-3.078860000
6	-0.268815000	-0.021773000	-2.099760000
6	-1.674025000	0.090391000	-2.296954000
15	0.374649000	-0.034641000	-0.413879000
6	-2.571500000	0.310250000	-1.247816000
8	-2.267763000	0.487664000	-0.052221000
8	-3.881509000	0.327563000	-1.622231000
6	-4.831477000	0.554509000	-0.586771000
6	-6.201613000	0.563548000	-1.219132000
6	-0.350926000	-1.385106000	0.505186000
6	2.138517000	-0.395101000	-0.483852000
6	0.331616000	1.564626000	0.384167000
6	3.021414000	0.631421000	-0.837825000
6	4.380970000	0.389690000	-0.939793000
6	4.840030000	-0.889526000	-0.674999000
6	4.000390000	-1.926467000	-0.317296000
6	2.638736000	-1.670797000	-0.225180000
6	-0.352963000	2.627965000	-0.202877000
6	-0.313878000	3.885598000	0.377302000
6	0.401231000	4.046109000	1.551176000
6	1.092771000	3.013750000	2.157680000
6	1.065821000	1.765298000	1.553583000
6	-0.547241000	-1.323406000	1.882739000
6	-1.074852000	-2.412454000	2.558466000
6	-1.398267000	-3.544717000	1.831110000
6	-1.218231000	-3.634701000	0.464096000
6	-0.687430000	-2.538370000	-0.202325000
9	-1.912169000	-4.599524000	2.482853000
9	0.433077000	5.259700000	2.120905000
9	6.155694000	-1.128754000	-0.764197000
1	1.708895000	-0.339892000	-2.886344000
1	0.335355000	-0.217389000	-4.114135000
1	-2.058909000	-0.027731000	-3.302133000
1	-4.748514000	-0.232515000	0.165960000
1	-4.616610000	1.504603000	-0.093003000
1	-6.963254000	0.732348000	-0.457466000
1	-6.277914000	1.356446000	-1.963936000
1	-6.408099000	-0.389813000	-1.706595000
1	2.642447000	1.625829000	-1.040961000
1	5.078475000	1.168866000	-1.214257000
1	4.408202000	-2.907302000	-0.115501000
1	1.972088000	-2.475773000	0.054008000
1	-0.913686000	2.469372000	-1.115564000
1	-0.828770000	4.729461000	-0.060657000
1	1.645462000	3.193640000	3.069311000
1	1.628661000	0.950197000	1.993299000
1	-0.313041000	-0.420903000	2.432055000
1	-1.246120000	-2.389843000	3.625853000
1	-1.491853000	-4.540000000	-0.060070000
1	-0.534994000	-2.583828000	-1.274505000

2_Ppfp3_3

6	-0.657933000	-0.198884000	-3.078886000
6	0.268479000	-0.021883000	-2.099706000
6	1.673688000	0.090117000	-2.296782000
15	-0.375150000	-0.034768000	-0.413906000
6	2.571338000	0.310065000	-1.247732000
8	2.267986000	0.487868000	-0.052171000
8	3.881278000	0.327030000	-1.622558000
6	4.831462000	0.554652000	-0.587448000
6	6.201452000	0.563155000	-1.220133000
6	-0.331971000	1.564527000	0.383963000
6	-2.138923000	-0.395424000	-0.483797000
6	0.350874000	-1.384827000	0.505354000
6	-2.638985000	-1.671131000	-0.224875000
6	-4.000621000	-1.926916000	-0.316772000
6	-4.840386000	-0.890072000	-0.674517000
6	-4.381472000	0.389146000	-0.939579000
6	-3.021925000	0.630992000	-0.837796000
6	0.548450000	-1.322448000	1.882709000
6	1.076646000	-2.411181000	2.558460000

6	1.399388000	-3.543780000	1.831316000
6	1.218133000	-3.634461000	0.464503000
6	0.686753000	-2.538425000	-0.201927000
6	0.352608000	2.627795000	-0.203220000
6	0.313738000	3.885442000	0.376927000
6	-0.401145000	4.046004000	1.550943000
6	-1.092706000	3.013728000	2.157584000
6	-1.065946000	1.765267000	1.553522000
9	-0.432801000	5.259606000	2.120664000
9	1.913948000	-4.598247000	2.483105000
9	-6.156043000	-1.129426000	-0.763539000
1	-1.709259000	-0.339604000	-2.886419000
1	-0.335702000	-0.216742000	-4.114170000
1	2.058639000	-0.028219000	-3.301921000
1	4.616749000	1.505122000	-0.094309000
1	4.748618000	-0.231824000	0.165865000
1	6.277609000	1.355483000	-1.965563000
1	6.963324000	0.732490000	-0.458814000
1	6.407766000	-0.390591000	-1.706927000
1	-1.972250000	-2.476014000	0.054402000
1	-4.408338000	-2.907755000	-0.114801000
1	-5.079083000	1.168219000	-1.214069000
1	-2.643039000	1.625403000	-1.041098000
1	0.314799000	-0.419650000	2.431770000
1	1.248916000	-2.388106000	3.625677000
1	1.491358000	-4.539995000	-0.059460000
1	0.533373000	-2.584314000	-1.273963000
1	0.913175000	2.469111000	-1.115999000
1	0.828628000	4.729265000	-0.061108000
1	-1.645298000	3.193722000	3.069257000
1	-1.628763000	0.950181000	1.993320000

2_PCy3_1

6	0.393988000	-0.161179000	-2.986622000
6	-0.425374000	0.082293000	-1.930404000
6	-1.831604000	0.286540000	-2.077856000
15	0.384121000	-0.011071000	-0.316578000
6	-2.754532000	0.543715000	-1.063377000
8	-2.538671000	0.681710000	0.155462000
8	-4.040866000	0.664482000	-1.534718000
6	-5.070128000	0.752149000	-0.559146000
6	-5.424480000	-0.614054000	-0.010809000
6	-0.359112000	-1.373050000	0.635628000
6	2.177418000	-0.306107000	-0.433761000
6	0.204923000	1.521577000	0.669578000
6	2.677997000	-1.676512000	-0.905786000
6	4.138700000	-1.830771000	-0.486164000
6	5.003114000	-0.709738000	-1.051578000
6	4.458336000	0.660359000	-0.663183000
6	3.006008000	0.797730000	-1.108588000
6	1.346576000	1.830438000	1.647158000
6	0.958146000	3.026236000	2.512902000
6	0.660184000	4.247260000	1.653174000
6	-0.450779000	3.938155000	0.658875000
6	-0.094322000	2.739019000	-0.211984000
6	-0.775904000	-2.571850000	-0.223889000
6	-1.535281000	-3.578650000	0.633839000
6	-0.697136000	-4.024892000	1.824572000
6	-0.295694000	-2.824852000	2.671654000
6	0.480891000	-1.806424000	1.841898000
1	1.447925000	-0.350556000	-2.893912000
1	-0.021205000	-0.178534000	-3.989070000
1	-2.217942000	0.213067000	-3.088429000
1	-4.762368000	1.423095000	0.243386000
1	-5.922839000	1.194869000	-1.074255000
1	-4.559843000	-1.055691000	0.486902000
1	-6.237189000	-0.535659000	0.713131000
1	-5.742852000	-1.278689000	-0.815201000
1	-1.279018000	-0.911240000	1.009834000
1	2.420734000	-0.258137000	0.639784000
1	-0.695326000	1.322035000	1.257092000
1	2.085594000	-2.487814000	-0.485713000
1	2.600705000	-1.752801000	-1.992888000
1	4.191424000	-1.812349000	0.609096000
1	4.516326000	-2.802471000	-0.809690000
1	6.035513000	-0.820764000	-0.714487000
1	5.011718000	-0.786156000	-2.144710000
1	4.510767000	0.784014000	0.425343000
1	5.063504000	1.451652000	-1.109252000
1	2.620713000	1.792044000	-0.884456000
1	2.961402000	0.681953000	-2.193194000
1	1.566698000	0.971079000	2.281974000
1	2.264437000	2.075310000	1.105167000
1	0.066919000	2.770357000	3.096518000
1	1.759560000	3.239927000	3.222917000
1	0.388052000	5.098607000	2.280278000
1	1.566626000	4.527625000	1.103911000
1	-0.658696000	4.804931000	0.028538000
1	-1.371718000	3.708420000	1.207195000
1	0.784105000	2.977011000	-0.822874000
1	-0.914307000	2.510084000	-0.893279000
1	-1.400665000	-2.232838000	-1.052273000
1	0.103542000	-3.059039000	-0.655151000
1	-2.456332000	-3.109051000	0.999008000
1	-1.829853000	-4.435109000	0.024499000
1	-1.245135000	-4.750958000	2.428274000
1	0.207339000	-4.525786000	1.459417000
1	-1.198833000	-2.346873000	3.066666000
1	0.305644000	-3.138036000	3.527312000
1	1.419443000	-2.261181000	1.508791000
1	0.738293000	-0.944177000	2.459726000

2_PCy3_2

6	-0.397734000	0.201540000	-2.977481000
6	0.439045000	0.239795000	-1.907556000
6	1.837808000	0.501070000	-2.032295000
15	-0.383577000	0.042837000	-0.309504000
6	2.768768000	0.588416000	-0.996881000
8	2.571692000	0.454022000	0.225468000
8	4.040520000	0.857159000	-1.445739000
6	5.085315000	0.753623000	-0.488171000
6	5.476403000	-0.691214000	-0.259019000
6	-0.213866000	1.588902000	0.635617000
6	-2.156767000	-0.338810000	-0.468344000
6	0.313694000	-1.310811000	0.709497000
6	-3.121029000	0.744376000	-0.966546000
6	-4.540962000	0.347023000	-0.567945000
6	-4.916581000	-1.016078000	-1.138186000
6	-3.908177000	-2.085445000	-0.732410000
6	-2.499799000	-1.672923000	-1.148009000
6	-0.666522000	-1.998371000	1.669646000
6	0.102462000	-2.956175000	2.575918000
6	0.852409000	-3.998407000	1.757546000
6	1.804004000	-3.321085000	0.780929000
6	1.060577000	-2.352255000	-0.131737000
6	-0.247099000	2.847082000	-0.238665000
6	0.061626000	4.073661000	0.613131000
6	-0.912449000	4.190411000	1.778329000
6	-0.860171000	2.936825000	2.641358000
6	-1.183650000	1.693619000	1.817747000
1	-1.454491000	0.020180000	-2.903257000
1	0.002817000	0.368073000	-3.972168000
1	2.205768000	0.647571000	-3.041715000
1	5.919211000	1.318910000	-0.904685000
1	4.778453000	1.224446000	0.446265000
1	5.795696000	-1.154031000	-1.193884000
1	6.298467000	-0.755838000	0.455641000
1	4.628297000	-1.251822000	0.137843000
1	0.802068000	1.500905000	1.034380000
1	-2.390972000	-0.469374000	0.600138000
1	1.063232000	-0.790365000	1.312041000
1	-2.879665000	1.722395000	-0.553319000
1	-3.062163000	0.831876000	-2.053796000
1	-4.600820000	0.312562000	0.526517000
1	-5.247378000	1.107796000	-0.905087000
1	-5.921709000	-1.297312000	-0.818106000
1	-4.934586000	-0.948619000	-2.231804000
1	-3.932885000	-2.222578000	0.355459000
1	-4.166875000	-3.044113000	-1.185713000
1	-1.777593000	-2.452151000	-0.905471000
1	-2.475134000	-1.550402000	-2.232685000
1	-1.202091000	-1.268205000	2.277954000
1	-1.416890000	-2.568300000	1.114291000
1	0.819958000	-2.383329000	3.173802000
1	-0.588663000	-3.435565000	3.271997000
1	1.396948000	-4.680073000	2.413954000
1	0.129086000	-4.600448000	1.195177000
1	2.335495000	-4.061825000	0.180269000
1	2.558704000	-2.758956000	1.344338000
1	0.347140000	-2.907321000	-0.751408000
1	1.760283000	-1.855646000	-0.804710000
1	0.481106000	2.754297000	-1.046562000
1	-1.232018000	2.967622000	-0.699476000
1	1.080923000	3.984127000	1.005384000
1	0.032320000	4.971054000	-0.007723000
1	-0.688424000	5.075004000	2.377660000
1	-1.928954000	4.314699000	1.386370000
1	0.146019000	2.832791000	3.061859000
1	-1.555253000	3.013910000	3.479704000
1	-2.215997000	1.763404000	1.459775000
1	-1.117320000	0.804035000	2.446629000

2_PCy3_3

6	-0.268631000	0.217485000	-2.948817000
6	0.507817000	0.090514000	-1.840622000
6	1.935035000	0.113411000	-1.893442000
15	-0.413085000	0.031670000	-0.286872000
6	2.826172000	0.002748000	-0.823375000
8	2.569566000	-0.147479000	0.385815000

8	4.139520000	0.066619000	-1.217833000
6	5.111698000	-0.046466000	-0.189000000
6	6.472967000	0.032115000	-0.837231000
6	0.012202000	1.511814000	0.680622000
6	-2.219227000	-0.016234000	-0.521680000
6	-0.021157000	-1.438424000	0.733507000
6	-2.944891000	1.239041000	-1.019788000
6	-4.435055000	1.093956000	-0.716635000
6	-5.015959000	-0.161886000	-1.355443000
6	-4.246083000	-1.405421000	-0.925481000
6	-2.765103000	-1.251398000	-1.254766000
6	-1.148964000	-1.946964000	1.641897000
6	-0.608014000	-3.049674000	2.548343000
6	-0.035800000	-4.198423000	1.728905000
6	1.063088000	-3.694607000	0.803363000
6	0.550213000	-2.585741000	-0.107725000
6	0.201907000	2.770326000	-0.174031000
6	0.694440000	3.917903000	0.701756000
6	-0.267196000	4.178534000	1.853347000
6	-0.429787000	2.924119000	2.700969000
6	-0.936962000	1.757903000	1.857793000
1	-1.343262000	0.228516000	-2.927198000
1	0.203715000	0.324720000	-3.919920000
1	2.368180000	0.234712000	-2.880281000
1	4.975769000	0.756414000	0.539600000
1	4.981677000	-0.992646000	0.341559000
1	6.598482000	0.982513000	-1.357263000
1	7.254229000	-0.052163000	-0.081202000
1	6.603616000	-0.775456000	-1.558505000
1	0.995003000	1.243492000	1.083654000
1	-2.519246000	-0.127690000	0.532400000
1	0.788501000	-1.076056000	1.372975000
1	-2.560407000	2.139179000	-0.542760000
1	-2.801864000	1.359474000	-2.096145000
1	-4.567041000	1.040864000	0.370685000
1	-4.970261000	1.980279000	-1.062271000
1	-6.073828000	-0.260333000	-1.104374000
1	-4.951454000	-0.068332000	-2.445377000
1	-4.358472000	-1.553494000	0.155427000
1	-4.648126000	-2.292792000	-1.417734000
1	-2.212922000	-2.154798000	-0.995917000
1	-2.658613000	-1.118068000	-2.333074000
1	-1.561551000	-1.140608000	2.250345000
1	-1.970412000	-2.354765000	1.045652000
1	0.180569000	-2.633559000	3.185063000
1	-1.402764000	-3.403942000	3.207838000
1	0.345060000	-4.982989000	2.385813000
1	-0.837283000	-4.642335000	1.126837000
1	1.467065000	-4.511090000	0.201657000
1	1.888416000	-3.296924000	1.405349000
1	-0.229894000	-2.984638000	-0.766006000
1	1.355154000	-2.215331000	-0.742639000
1	0.918420000	2.568901000	-0.972400000
1	-0.740376000	3.059774000	-0.647777000
1	1.677958000	3.656549000	1.108306000
1	0.824333000	4.815428000	0.094124000
1	0.085998000	5.009669000	2.466837000
1	-1.244041000	4.468420000	1.448235000
1	0.540600000	2.655452000	3.132532000
1	-1.115391000	3.102758000	3.531566000
1	-1.942153000	1.993399000	1.493111000
1	-1.016029000	0.861534000	2.475710000

2_PBu3_1

6	-0.160167000	-0.238482000	2.817798000
6	0.422187000	-0.003903000	1.613458000
6	1.758366000	0.468666000	1.438411000
15	-0.645851000	-0.228295000	0.176347000
6	2.449130000	0.458376000	0.223738000
8	2.027889000	0.061103000	-0.880287000
8	3.726475000	0.941725000	0.315923000
6	4.490675000	0.935464000	-0.882262000
6	5.858097000	1.484470000	-0.554436000
6	-2.331320000	-0.527586000	0.749395000
6	-0.714045000	1.284855000	-0.784696000
6	-0.239431000	-1.680687000	-0.811175000
6	-1.248742000	2.460312000	0.030088000
6	-3.372872000	-0.455018000	-0.366954000
6	0.556313000	-2.706364000	-0.009215000
6	-4.743085000	-0.898725000	0.130496000
6	-5.802515000	-0.795817000	-0.955175000
6	0.833245000	-3.962316000	-0.823175000
6	1.633481000	-4.985558000	-0.032429000
6	-1.218817000	3.753441000	-0.774122000
6	-1.751138000	4.931756000	0.025932000
1	-1.186085000	-0.549377000	2.936419000
1	0.420922000	-0.110155000	3.724825000
1	2.300247000	0.768050000	2.327915000
1	4.557202000	-0.082709000	-1.272500000
1	3.993434000	1.543623000	-1.641444000
1	5.783572000	2.502912000	-0.171544000
1	6.351014000	0.868069000	0.198166000
1	6.480712000	1.498189000	-1.449499000
1	-2.339378000	-1.519267000	1.212843000
1	-2.574057000	0.200060000	1.525508000
1	0.296452000	1.479364000	-1.143340000
1	-1.344427000	1.086421000	-1.656980000
1	-1.186858000	-2.112851000	-1.144276000
1	0.322539000	-1.354122000	-1.683393000
1	-2.274661000	2.263601000	0.357987000
1	-0.644589000	2.580809000	0.935668000
1	-3.442722000	0.570793000	-0.740847000
1	-3.082268000	-1.077405000	-1.218690000
1	1.499892000	-2.253266000	0.307767000
1	0.010559000	-2.971731000	0.903883000
1	-4.674526000	-1.928500000	0.492514000
1	-5.025489000	-0.284762000	0.990635000
1	-5.540895000	-1.418978000	-1.813163000
1	-6.780287000	-1.116686000	-0.594535000
1	-5.897034000	0.232959000	-1.309373000
1	-0.115933000	-4.398624000	-1.148777000
1	1.375782000	-3.682641000	-1.731020000
1	1.094086000	-5.288733000	0.867657000
1	1.834898000	-5.881879000	-0.620244000
1	2.592127000	-4.567501000	0.281684000
1	-0.191160000	3.949010000	-1.092573000
1	-1.808596000	3.621174000	-1.686180000
1	-1.725764000	5.855711000	-0.552811000
1	-2.784545000	4.757447000	0.333836000
1	-1.157485000	5.085488000	0.929533000

2_PBu3_2

6	0.836358000	0.611711000	2.456218000
6	0.027022000	0.237612000	1.430608000
6	-1.319289000	-0.204120000	1.586724000
15	0.756573000	0.361891000	-0.223212000
6	-2.165986000	-0.402735000	0.498875000
8	-1.862054000	-0.237029000	-0.701377000
8	-3.426649000	-0.813682000	0.821682000
6	-4.324725000	-1.009477000	-0.263686000
6	-5.650005000	-1.443569000	0.313543000
6	2.514398000	0.737132000	-0.040392000
6	0.759365000	-1.158108000	-1.195397000
6	0.120829000	1.761226000	-1.169960000
6	0.591814000	-2.425203000	-0.361504000
6	3.390107000	-0.383187000	0.524288000
6	-0.572801000	2.804148000	-0.299833000
6	4.790598000	0.112286000	0.873877000
6	5.535945000	0.689545000	-0.321001000
6	-1.025494000	4.005250000	-1.118152000
6	-1.727707000	5.044999000	-0.258748000
6	0.851405000	-3.673959000	-1.192443000
6	0.648963000	-4.946560000	-0.385107000
1	1.860167000	0.927729000	2.333495000
1	0.448938000	0.597857000	3.469404000
1	-1.705586000	-0.334772000	2.590044000
1	-4.425516000	-0.081115000	-0.830422000
1	-3.923516000	-1.765209000	-0.942583000
1	-6.371808000	-1.605892000	-0.487402000
1	-5.541283000	-2.373576000	0.872618000
1	-6.046675000	-0.681333000	0.985036000
1	2.828844000	0.976440000	-1.060997000
1	2.618196000	1.660600000	0.536877000
1	-0.034434000	-1.079773000	-1.935629000
1	1.721343000	-1.173145000	-1.718416000
1	0.979084000	2.203285000	-1.683957000
1	-0.567765000	1.367136000	-1.915484000
1	1.273320000	-2.410054000	0.496240000
1	-0.421526000	-2.451166000	0.046127000
1	2.925558000	-0.815817000	1.412141000
1	3.464599000	-1.186930000	-0.215732000
1	-1.434734000	2.342529000	0.190428000
1	0.102168000	3.135743000	0.497232000
1	4.711206000	0.868786000	1.661135000
1	5.360656000	-0.718713000	1.294892000
1	5.569360000	-0.032255000	-1.141210000
1	5.056406000	1.596094000	-0.694640000
1	6.562971000	0.946831000	-0.059852000
1	-0.157918000	4.452078000	-1.613185000
1	-1.694735000	3.661761000	-1.912498000
1	-2.052507000	5.903181000	-0.848280000
1	-2.608364000	4.616589000	0.224365000
1	-1.063964000	5.410398000	0.527975000
1	0.184875000	-3.669677000	-2.060123000
1	1.872931000	-3.636663000	-1.584196000
1	1.319676000	-4.969461000	0.476650000
1	-0.374389000	-5.007621000	-0.009021000
1	0.840663000	-5.838499000	-0.982757000

2_PBu3_3

6	0.709015000	-1.258461000	2.052654000
6	-0.052950000	-0.607283000	1.133450000
6	-1.327741000	-0.032398000	1.409790000
15	0.656675000	-0.531541000	-0.529340000
6	-2.196673000	0.398204000	0.407946000
8	-1.979375000	0.368024000	-0.820332000
8	-3.384231000	0.893306000	0.867376000
6	-4.312466000	1.325846000	-0.118960000
6	-5.546769000	1.814025000	0.599701000
6	2.400177000	-1.001316000	-0.434150000
6	-0.025733000	-1.732632000	-1.685019000
6	0.717841000	1.101596000	-1.290202000
6	-0.198824000	-3.129012000	-1.079984000
6	3.326014000	-0.012146000	0.269732000
6	0.662610000	2.259867000	-0.297171000
6	4.751113000	-0.545085000	0.345099000
6	5.696108000	0.449467000	1.000614000
6	1.053635000	3.573653000	-0.959308000
6	0.958097000	4.746851000	0.003239000
6	-1.547048000	-3.303283000	-0.392253000
6	-1.641096000	-4.636503000	0.331650000
1	1.692052000	-1.652551000	1.849924000
1	0.331762000	-1.382446000	3.062152000
1	-1.663911000	-0.011954000	2.439330000
1	-3.866785000	2.120001000	-0.722217000
1	-4.548167000	0.497905000	-0.791476000
1	-6.288558000	2.158520000	-0.121376000
1	-5.989549000	1.013475000	1.193284000
1	-5.303099000	2.642889000	1.265245000
1	2.486921000	-1.999873000	-0.000679000
1	2.698940000	-1.094051000	-1.483906000
1	-0.973805000	-1.343118000	-2.052379000
1	0.686855000	-1.740366000	-2.514828000
1	1.662861000	1.123719000	-1.844536000
1	-0.100463000	1.168569000	-2.004416000
1	-0.099865000	-3.873033000	-1.874263000
1	0.602699000	-3.339840000	-0.362295000
1	3.326962000	0.940382000	-0.270425000
1	2.962228000	0.201005000	1.278554000
1	-0.348091000	2.333118000	0.109690000
1	1.327375000	2.068550000	0.552573000
1	4.749063000	-1.485522000	0.904250000
1	5.098556000	-0.784363000	-0.664516000
1	6.713494000	0.061176000	1.058646000
1	5.726022000	1.384996000	0.437709000
1	5.368290000	0.684211000	2.015548000
1	2.074070000	3.488173000	-1.346930000
1	0.405666000	3.743660000	-1.824439000
1	1.612539000	4.595524000	0.864503000
1	1.243716000	5.684893000	-0.474239000
1	-0.061394000	4.857585000	0.378435000
1	-1.705433000	-2.482699000	0.312550000
1	-2.337421000	-3.220446000	-1.144272000
1	-0.885344000	-4.698352000	1.118788000
1	-2.618234000	-4.776694000	0.795037000
1	-1.473093000	-5.468902000	-0.356245000

3_PMe3_1

6	1.133973000	-0.764767000	-1.284748000
6	-0.111214000	-1.259990000	-1.292494000
6	-1.065883000	-1.031912000	-0.250047000
8	-1.142885000	-1.663024000	0.803577000
8	-1.990081000	-0.091429000	-0.566597000
6	-2.997454000	0.156649000	0.414633000
6	-3.869623000	1.273747000	-0.101247000
15	1.800258000	0.241815000	0.012620000
6	3.175231000	1.185082000	-0.616070000
6	0.603253000	1.376189000	0.692342000
6	2.392922000	-0.762859000	1.362317000
1	1.868143000	-0.997136000	-2.057915000
1	-2.522384000	0.426766000	1.361319000
1	-3.571057000	-0.756286000	0.583128000
1	-4.655818000	1.495546000	0.620610000
1	-3.282634000	2.179202000	-0.260470000
1	-4.337562000	0.993935000	-1.045297000
1	3.637497000	1.757416000	0.187729000
1	3.910281000	0.502301000	-1.042997000
1	2.822718000	1.862840000	-1.393537000
1	-0.143213000	0.833815000	1.272244000
1	0.118560000	1.931380000	-0.109428000
1	1.125820000	2.066865000	1.355544000
1	2.802557000	-0.132792000	2.152382000
1	1.556334000	-1.347108000	1.747435000
1	3.163388000	-1.441181000	0.995996000

3_PMe3_2

6	1.119463000	-1.349051000	-0.629387000
6	-0.160495000	-1.726131000	-0.545937000
6	-1.248514000	-1.023658000	0.050046000
8	-1.575185000	-1.091470000	1.235538000
8	-2.016687000	-0.359790000	-0.854497000
6	-3.255405000	0.175741000	-0.383299000
6	-3.075836000	1.534648000	0.255501000
15	1.799692000	0.169461000	-0.024662000
6	3.030980000	0.773276000	-1.163992000
6	0.560643000	1.427210000	0.193552000
6	2.605902000	-0.079553000	1.547658000
1	1.891479000	-1.996543000	-1.051505000
1	-3.704937000	-0.526982000	0.318261000
1	-3.890187000	0.243689000	-1.266084000
1	-4.048409000	1.947269000	0.528065000
1	-2.469186000	1.457861000	1.157401000
1	-2.594352000	2.226106000	-0.438090000
1	3.504870000	1.669428000	-0.763678000
1	3.787073000	0.002129000	-1.314397000
1	2.558249000	1.001975000	-2.118931000
1	-0.118164000	1.143485000	0.996925000
1	0.002211000	1.558365000	-0.732446000
1	1.059975000	2.359900000	0.458820000
1	3.053799000	0.851952000	1.894811000
1	1.865949000	-0.426722000	2.269286000
1	3.380799000	-0.838637000	1.438300000

3_PMe3_3

6	1.072106000	-0.998327000	-1.110879000
6	-0.241690000	-1.261577000	-1.097237000
6	-1.163957000	-0.671044000	-0.175527000
8	-1.369681000	-1.056267000	0.975071000
8	-1.894311000	0.332284000	-0.724840000
6	-2.902426000	0.922044000	0.099289000
6	-4.175322000	0.106394000	0.077620000
15	1.880421000	0.112352000	0.008285000
6	3.409499000	0.673202000	-0.715042000
6	0.892288000	1.543915000	0.402884000
6	2.265731000	-0.699147000	1.549716000
1	1.769749000	-1.503821000	-1.780535000
1	-3.066154000	1.914677000	-0.318306000
1	-2.522871000	1.027199000	1.116845000
1	-4.944043000	0.596429000	0.676847000
1	-4.546881000	0.005291000	-0.942660000
1	-3.997410000	-0.887298000	0.487423000
1	3.948395000	1.305714000	-0.010053000
1	4.023714000	-0.191472000	-0.967847000
1	3.194443000	1.236487000	-1.623097000
1	0.047230000	1.261984000	1.030480000
1	0.533544000	2.011560000	-0.512779000
1	1.518932000	2.246755000	0.953726000
1	2.767339000	-0.006820000	2.226637000
1	1.332710000	-1.045792000	1.995772000
1	2.909542000	-1.557053000	1.356480000

3_PPhMe2_1

6	-0.328357000	-1.507062000	-1.364552000
6	-1.608351000	-1.155683000	-1.523785000
6	-2.498201000	-0.644455000	-0.533321000
8	-3.328183000	-1.287710000	0.104293000
8	-2.436913000	0.712845000	-0.427898000
6	-3.376666000	1.318593000	0.460540000
6	-3.089455000	2.799260000	0.482424000
15	0.620782000	-1.374272000	0.126965000
6	-0.401280000	-1.075834000	1.555452000
6	1.545898000	-2.871531000	0.402494000
6	1.770057000	-0.006640000	0.005150000
6	1.240467000	1.247066000	-0.312554000
6	2.077274000	2.348908000	-0.401529000
6	3.444516000	2.201310000	-0.184059000
6	3.974080000	0.954171000	0.122630000
6	3.138761000	-0.153005000	0.220003000
1	0.281755000	-1.885678000	-2.187621000
1	-4.390154000	1.109846000	0.113188000
1	-3.271206000	0.877076000	1.454404000
1	-3.784735000	3.304205000	1.153218000
1	-3.197172000	3.229174000	-0.513633000
1	-2.073939000	2.989219000	0.834245000
1	0.243249000	-1.085100000	2.435131000
1	-0.877790000	-0.098378000	1.476318000
1	-1.160987000	-1.851555000	1.640103000
1	0.833346000	-3.690314000	0.503884000
1	2.193380000	-3.067728000	-0.452255000
1	2.143819000	-2.798211000	1.310355000
1	0.175419000	1.350407000	-0.497925000
1	1.666678000	3.319463000	-0.646800000
1	4.098083000	3.060785000	-0.258613000
1	5.037602000	0.840357000	0.286531000
1	3.559231000	-1.121170000	0.460487000

3_PPhMe2_2

6	-0.308734000	1.037348000	-1.590426000
6	-1.423138000	0.339056000	-1.824896000
6	-2.192772000	-0.451094000	-0.924993000

8	-1.991887000	-1.641323000	-0.674801000
8	-3.295186000	0.190149000	-0.455177000
6	-4.240214000	-0.594693000	0.276117000
6	-3.860956000	-0.715152000	1.735068000
15	0.506184000	1.195269000	-0.024257000
6	1.079566000	2.865585000	0.205477000
6	-0.578391000	0.771501000	1.322666000
6	1.913014000	0.091750000	0.055727000
6	3.193913000	0.530175000	0.384762000
6	4.240436000	-0.383191000	0.442304000
6	4.007004000	-1.726802000	0.176702000
6	2.727604000	-2.165568000	-0.153470000
6	1.680972000	-1.258566000	-0.219775000
1	0.242938000	1.546623000	-2.383314000
1	-5.190070000	-0.073118000	0.164360000
1	-4.323625000	-1.577883000	-0.186740000
1	-4.641645000	-1.250173000	2.277929000
1	-3.747163000	0.271990000	2.186282000
1	-2.926105000	-1.264163000	1.845472000
1	1.749289000	3.139506000	-0.609828000
1	0.211340000	3.524620000	0.190108000
1	1.596770000	2.970665000	1.158574000
1	-0.820625000	-0.290087000	1.276757000
1	-0.059734000	0.980589000	2.258882000
1	-1.491065000	1.363345000	1.259645000
1	3.381878000	1.575579000	0.594558000
1	5.236591000	-0.043832000	0.694183000
1	4.824307000	-2.434948000	0.222562000
1	2.548607000	-3.211435000	-0.365138000
1	0.682299000	-1.589022000	-0.492184000

3_PPhMe2_3

6	-0.328880000	1.384416000	1.389076000
6	-1.579269000	0.971701000	1.611547000
6	-2.524236000	0.396652000	0.716952000
8	-3.411618000	1.001854000	0.117189000
8	-2.458736000	-0.965773000	0.688840000
6	-3.525360000	-1.647392000	0.024006000
6	-3.276328000	-1.755601000	-1.463525000
15	0.555644000	1.272342000	-0.141989000
6	-0.511088000	0.789792000	-1.482066000
6	1.304577000	2.837930000	-0.543756000
6	1.846859000	0.038129000	-0.016165000
6	3.176304000	0.304537000	-0.337077000
6	4.126940000	-0.704159000	-0.229131000
6	3.750961000	-1.973024000	0.193595000
6	2.423292000	-2.240734000	0.515911000
6	1.472405000	-1.236754000	0.416256000
1	0.290934000	1.822597000	2.174464000
1	-3.566619000	-2.633955000	0.484310000
1	-4.461569000	-1.126587000	0.224521000
1	-4.075525000	-2.329724000	-1.934662000
1	-2.330643000	-2.264784000	-1.658944000
1	-3.246783000	-0.765526000	-1.919014000
1	0.071077000	0.803197000	-2.403862000
1	-0.877006000	-0.221427000	-1.305357000
1	-1.351417000	1.478940000	-1.557367000
1	1.860727000	2.773519000	-1.478449000
1	0.506112000	3.572929000	-0.647385000
1	1.970506000	3.146408000	0.262158000
1	3.476560000	1.290923000	-0.667144000
1	5.160246000	-0.496952000	-0.474463000
1	4.493891000	-2.755681000	0.277148000
1	2.132703000	-3.227840000	0.850341000
1	0.437915000	-1.432768000	0.681580000

3_PPh2Me_1

6	0.343303000	-1.960357000	-0.777755000
6	-0.917622000	-2.394067000	-0.912468000
6	-2.005793000	-1.837886000	-0.155845000
8	-2.239782000	-2.054940000	1.031722000
8	-2.853697000	-1.106278000	-0.913776000
6	-3.886664000	-0.416463000	-0.208854000
6	-4.706179000	0.341123000	-1.223090000
15	0.827404000	-0.698745000	0.379990000
6	0.932682000	-1.379828000	2.027360000
6	2.465280000	-0.111093000	-0.042492000
6	-0.303667000	0.680842000	0.383696000
6	3.483196000	-1.062862000	-0.153463000
6	4.770901000	-0.664356000	-0.480326000
6	5.047353000	0.682212000	-0.696584000
6	4.037477000	1.629919000	-0.585152000
6	2.745013000	1.236899000	-0.258761000
6	-0.729427000	1.263376000	1.577266000
6	-1.558877000	2.377683000	1.543412000
6	-1.958905000	2.909593000	0.322974000
6	-1.538636000	2.326318000	-0.868871000
6	-0.716056000	1.209801000	-0.841219000
1	1.190337000	-2.401781000	-1.303335000
1	-4.496086000	-1.135799000	0.339741000
1	-3.426774000	0.257537000	0.520401000
1	-5.497500000	0.897599000	-0.720103000
1	-4.083847000	1.047655000	-1.773845000
1	-5.166381000	-0.343531000	-1.936145000
1	-0.055316000	-1.731455000	2.324937000
1	1.617126000	-2.228497000	1.986313000
1	1.316351000	-0.642881000	2.732229000
1	3.268556000	-2.112390000	0.015836000
1	5.557257000	-1.402489000	-0.567179000
1	6.052430000	0.991632000	-0.952341000
1	4.253413000	2.676940000	-0.752258000
1	1.960363000	1.978230000	-0.172055000
1	-0.422325000	0.851411000	2.529923000
1	-1.893012000	2.827027000	2.469107000
1	-2.604378000	3.778223000	0.299414000
1	-1.856351000	2.737388000	-1.817984000
1	-0.397352000	0.740975000	-1.765036000

3_PPh2Me_2

6	-0.206512000	-0.734904000	2.180599000
6	0.830057000	-1.527850000	1.881839000
6	1.538887000	-1.519795000	0.637653000
8	1.170373000	-2.086301000	-0.390508000
8	2.753291000	-0.925456000	0.722476000
6	3.534515000	-0.885649000	-0.472988000
6	4.314298000	-2.167232000	-0.663461000
15	-0.905535000	0.514354000	1.131434000
6	-1.529429000	1.832699000	2.162527000
6	0.307400000	1.231318000	0.029655000
6	-2.261036000	-0.133512000	0.160609000
6	1.381064000	1.918089000	0.601351000
6	2.316417000	2.537823000	-0.212972000
6	2.186215000	2.468004000	-1.597379000
6	1.125626000	1.774728000	-2.167706000
6	0.182701000	1.154939000	-1.355893000
6	-3.375621000	0.651898000	-0.138313000
6	-4.398806000	0.128682000	-0.917349000
6	-4.308968000	-1.171952000	-1.401488000
6	-3.197911000	-1.954369000	-1.106582000
6	-2.173470000	-1.440190000	-0.322938000
1	-0.766840000	-0.821957000	3.113773000
1	2.879880000	-0.691484000	-1.324312000
1	4.204849000	-0.035480000	-0.347225000
1	4.959278000	-2.353383000	0.196171000
1	3.635751000	-3.011024000	-0.783241000
1	4.940760000	-2.095654000	-1.553837000
1	-1.912502000	2.654272000	1.559228000
1	-2.317112000	1.443381000	2.808236000
1	-0.706143000	2.195467000	2.778886000
1	1.487173000	1.955179000	1.679707000
1	3.148053000	3.069856000	0.229898000
1	2.917936000	2.951221000	-2.232035000
1	1.030058000	1.714190000	-3.243711000
1	-0.642932000	0.612854000	-1.800036000
1	-3.451472000	1.665210000	0.234773000
1	-5.265425000	0.735331000	-1.144855000
1	-5.108884000	-1.577376000	-2.007572000
1	-3.129689000	-2.966666000	-1.482416000
1	-1.301586000	-2.041552000	-0.091665000

3_PPh2Me_3

6	0.558447000	0.280897000	1.656280000
6	1.868976000	0.107980000	1.852308000
6	2.830737000	-0.239176000	0.855898000
8	3.103240000	-1.384475000	0.496789000
8	3.531677000	0.823849000	0.390618000
6	4.539646000	0.536714000	-0.579715000
6	5.164003000	1.847640000	-0.987394000
15	-0.274406000	0.151583000	0.091102000
6	0.807786000	0.471516000	-1.289134000
6	-0.954789000	-1.492769000	-0.126050000
6	-1.637822000	1.303413000	0.048865000
6	-2.108421000	-1.694652000	-0.885782000
6	-2.581640000	-2.983266000	-1.089736000
6	-1.904902000	-4.067448000	-0.539758000
6	-0.752941000	-3.867350000	0.212287000
6	-0.275124000	-2.580291000	0.423159000
6	-2.660635000	1.142243000	0.987976000
6	-3.713425000	2.043545000	1.024230000
6	-3.749280000	3.105608000	0.124896000
6	-2.733489000	3.267420000	-0.808878000
6	-1.674172000	2.368437000	-0.848887000
1	-0.143064000	0.485262000	2.467242000
1	5.276226000	-0.142355000	-0.147366000
1	4.083643000	0.029439000	-1.433917000
1	5.940559000	1.674197000	-1.732511000
1	4.415800000	2.514005000	-1.418560000
1	5.615508000	2.343769000	-0.128040000
1	0.213499000	0.460117000	-2.203682000
1	1.305550000	1.433050000	-1.170267000

1	1.547464000	-0.327547000	-1.347633000
1	-2.635940000	-0.849194000	-1.311491000
1	-3.478650000	-3.141437000	-1.673849000
1	-2.278814000	-5.071079000	-0.696184000
1	-0.228216000	-4.711591000	0.639532000
1	0.623658000	-2.416082000	1.006817000
1	-2.632605000	0.308827000	1.681838000
1	-4.505507000	1.918208000	1.750475000
1	-4.572603000	3.807519000	0.153170000
1	-2.763311000	4.093120000	-1.507483000
1	-0.884176000	2.500267000	-1.577246000

3_PPh3_1

6	0.014769000	0.117466000	-2.125723000
6	-1.217319000	0.515683000	-2.463502000
6	-2.236459000	0.893045000	-1.528410000
8	-2.305255000	1.974411000	-0.947602000
8	-3.229632000	-0.020310000	-1.426961000
6	-4.238379000	0.263502000	-0.456749000
6	-5.238566000	-0.864490000	-0.492087000
15	0.625346000	-0.025670000	-0.459721000
6	-0.642101000	-0.563406000	0.677722000
6	1.963434000	-1.218354000	-0.427467000
6	1.324986000	1.518152000	0.119668000
6	-0.901975000	0.123076000	1.862524000
6	-1.842820000	-0.376989000	2.755260000
6	-2.515633000	-1.558375000	2.467011000
6	-2.263431000	-2.239447000	1.279047000
6	-1.333351000	-1.740873000	0.379780000
6	3.019492000	-1.019989000	-1.322579000
6	4.083475000	-1.908635000	-1.345188000
6	4.101956000	-2.993072000	-0.472643000
6	3.059293000	-3.185773000	0.424521000
6	1.987809000	-2.300248000	0.450242000
6	2.290582000	1.502315000	1.129937000
6	2.810258000	2.697025000	1.605090000
6	2.371717000	3.905477000	1.070902000
6	1.413753000	3.921358000	0.064540000
6	0.886092000	2.728250000	-0.414749000
1	0.790547000	-0.100540000	-2.860577000
1	-3.768107000	0.353867000	0.526892000
1	-4.708261000	1.220046000	-0.689870000
1	-4.755933000	-1.817423000	-0.271191000
1	-6.018687000	-0.692847000	0.250074000
1	-5.707152000	-0.933942000	-1.474233000
1	-0.377383000	1.043628000	2.086080000
1	-2.048467000	0.157030000	3.673419000
1	-3.243969000	-1.948153000	3.166526000
1	-2.793748000	-3.155145000	1.053033000
1	-1.138681000	-2.259345000	-0.552459000
1	3.012371000	-0.167351000	-1.992948000
1	4.898059000	-1.755067000	-2.040482000
1	4.933441000	-3.685648000	-0.491140000
1	3.077207000	-4.024797000	1.107555000
1	1.180037000	-2.452111000	1.154708000
1	2.633636000	0.559155000	1.539833000
1	3.558239000	2.686477000	2.386898000
1	2.782250000	4.836816000	1.439206000
1	1.076347000	4.861757000	-0.350684000
1	0.131660000	2.731647000	-1.191197000

3_PPh3_2

6	-0.043446000	0.053703000	-2.082733000
6	-1.335060000	0.251045000	-2.369246000
6	-2.376765000	0.446297000	-1.405516000
8	-2.607102000	1.499465000	-0.813695000
8	-3.196329000	-0.625543000	-1.288966000
6	-4.253763000	-0.523501000	-0.333610000
6	-5.463938000	0.165790000	-0.922668000
15	0.644635000	-0.015834000	-0.442412000
6	-0.500737000	-0.725958000	0.728959000
6	2.134516000	-1.011129000	-0.469690000
6	1.136906000	1.610049000	0.125463000
6	-0.840205000	-0.068240000	1.909886000
6	-1.700466000	-0.678530000	2.815024000
6	-2.215017000	-1.940295000	2.542526000
6	-1.882376000	-2.595137000	1.359779000
6	-1.031048000	-1.988449000	0.449284000
6	2.337847000	-2.087538000	0.391340000
6	3.519951000	-2.815689000	0.318383000
6	4.495259000	-2.471573000	-0.608803000
6	4.296942000	-1.392846000	-1.465867000
6	3.121284000	-0.661397000	-1.396822000
6	2.168724000	1.739114000	1.058623000
6	2.519631000	2.996485000	1.527775000
6	1.844866000	4.122839000	1.066067000
6	0.819151000	3.994711000	0.137072000
6	0.461193000	2.738583000	-0.337075000
1	0.729639000	-0.022311000	-2.848344000
1	-4.487033000	-1.551280000	-0.055846000
1	-3.889579000	0.003323000	0.550421000
1	-5.811698000	-0.365908000	-1.809223000
1	-6.275266000	0.184883000	-0.193467000
1	-5.221466000	1.190971000	-1.199770000
1	-0.442183000	0.916696000	2.119877000
1	-1.967997000	-0.166640000	3.729832000
1	-2.883283000	-2.413846000	3.250234000
1	-2.291929000	-3.573291000	1.144796000
1	-0.779371000	-2.486641000	-0.480330000
1	1.583199000	-2.356833000	1.119086000
1	3.676953000	-3.650186000	0.988965000
1	5.413734000	-3.041432000	-0.662853000
1	5.058462000	-1.121115000	-2.184721000
1	2.971752000	0.186479000	-2.055749000
1	2.695767000	0.860649000	1.412595000
1	3.319841000	3.098553000	2.248921000
1	2.123309000	5.103518000	1.429921000
1	0.297493000	4.871975000	-0.222021000
1	-0.343111000	2.628567000	-1.053756000

3_PPh3_3

6	0.043442000	0.053730000	-2.082687000
6	1.335027000	0.251133000	-2.369234000
6	2.376778000	0.446501000	-1.405542000
8	2.607082000	1.499624000	-0.813663000
8	3.196454000	-0.625284000	-1.289153000
6	4.253843000	-0.523293000	-0.333758000
6	5.463967000	0.166226000	-0.922684000
15	-0.644616000	-0.015897000	-0.442301000
6	-1.137252000	1.609914000	0.125494000
6	-2.134313000	-1.011454000	-0.469535000
6	0.500949000	-0.725739000	0.729059000
6	-2.169367000	1.738927000	1.058322000
6	-2.520506000	2.996302000	1.527327000
6	-1.845691000	4.122692000	1.065796000
6	-0.819652000	3.994608000	0.137139000
6	-0.461462000	2.738487000	-0.336844000
6	-3.121308000	-0.661358000	-1.396310000
6	-4.296893000	-1.392911000	-1.465501000
6	-4.494882000	-2.472099000	-0.608931000
6	-3.519345000	-2.816583000	0.317873000
6	-2.337304000	-2.088328000	0.390973000
6	0.840095000	-0.068071000	1.910105000
6	1.700418000	-0.678249000	2.815272000
6	2.215297000	-1.939863000	2.542696000
6	1.882963000	-2.594660000	1.359833000
6	1.031606000	-1.988070000	0.449293000
1	-0.729748000	-0.022290000	-2.848206000
1	3.889596000	0.003367000	0.550345000
1	4.487216000	-1.551083000	-0.056138000
1	5.811811000	-0.365310000	-1.809302000
1	5.221367000	1.191415000	-1.199652000
1	6.275254000	0.185317000	-0.193440000
1	-2.696473000	0.860465000	1.412195000
1	-3.320950000	3.098325000	2.248223000
1	-2.124337000	5.103367000	1.429512000
1	-0.297939000	4.871902000	-0.221805000
1	0.343073000	2.628523000	-1.053288000
1	-2.971978000	0.186885000	-2.054819000
1	-5.058609000	-1.120933000	-2.184054000
1	-5.413304000	-3.042041000	-0.663066000
1	-3.676130000	-3.651455000	0.988040000
1	-1.582461000	-2.357898000	1.118417000
1	0.441803000	0.916739000	2.120165000
1	1.967730000	-0.166399000	3.730169000
1	2.883579000	-2.413340000	3.250441000
1	2.292794000	-3.572689000	1.144803000
1	0.780196000	-2.486192000	-0.480428000

3_TS_Pani3_1

6	0.203921000	-0.046273000	2.435002000
6	-0.951604000	-0.174200000	3.098199000
6	-2.214093000	-0.459937000	2.472254000
8	-2.856049000	-1.497116000	2.582781000
8	-2.715147000	0.608151000	1.798012000
6	-3.969847000	0.415545000	1.144846000
6	-4.221292000	1.634009000	0.291720000
15	0.437059000	-0.073344000	0.667517000
6	0.397482000	1.592342000	0.027025000
6	2.066025000	-0.712763000	0.307467000
6	-0.777370000	-1.056169000	-0.180065000
6	1.043859000	1.899465000	-1.178788000
6	0.970526000	3.170694000	-1.706459000
6	0.250388000	4.168047000	-1.035765000
6	-0.390767000	3.874994000	0.168061000
6	-0.312333000	2.589513000	0.689742000
6	2.283597000	-1.854067000	-0.469183000
6	3.567279000	-2.316743000	-0.686634000
6	4.660751000	-1.647896000	-0.129657000
6	4.456146000	-0.501684000	0.642503000

6	3.163828000	-0.044773000	0.851722000
6	-1.016684000	-2.361318000	0.248136000
6	-1.923983000	-3.173918000	-0.414654000
6	-2.610949000	-2.672955000	-1.522794000
6	-2.379667000	-1.362463000	-1.956268000
6	-1.471910000	-0.563683000	-1.291052000
8	0.233816000	5.379408000	-1.618088000
8	-3.512488000	-3.379969000	-2.225492000
8	5.874181000	-2.168252000	-0.387708000
6	-0.481871000	6.422812000	-0.979217000
6	-3.796262000	-4.706191000	-1.813614000
6	7.011030000	-1.524804000	0.161188000
1	1.144344000	0.192698000	2.937359000
1	-3.919663000	-0.492913000	0.539347000
1	-4.753106000	0.275595000	1.892639000
1	-4.252026000	2.536714000	0.903255000
1	-5.173839000	1.537204000	-0.229689000
1	-3.430194000	1.748024000	-0.452701000
1	1.604874000	1.134002000	-1.703010000
1	1.465054000	3.422071000	-2.635577000
1	-0.942407000	4.633568000	0.704317000
1	-0.811920000	2.362011000	1.622899000
1	1.447392000	-2.381709000	-0.910385000
1	3.748448000	-3.198247000	-1.287589000
1	5.288022000	0.034022000	1.076086000
1	3.012412000	0.852943000	1.441831000
1	-0.498905000	-2.744546000	1.120206000
1	-2.098733000	-4.178473000	-0.057824000
1	-2.927246000	-0.992986000	-2.813409000
1	-1.306506000	0.451074000	-1.631750000
1	-0.373746000	7.300523000	-1.610018000
1	-1.540505000	6.172031000	-0.885429000
1	-0.068804000	6.633827000	0.009277000
1	-4.543320000	-5.088829000	-2.503202000
1	-2.902971000	-5.332132000	-1.863535000
1	-4.196845000	-4.723663000	-0.798126000
1	6.966380000	-1.512087000	1.252292000
1	7.874677000	-2.101729000	-0.157123000
1	7.101535000	-0.502118000	-0.211221000

3_TS_Pani3_2

6	-0.133783000	-0.093238000	-2.370968000
6	1.081473000	0.034150000	-2.915248000
6	2.305851000	0.216682000	-2.196375000
8	2.713614000	1.287138000	-1.746891000
8	3.073695000	-0.900080000	-2.166905000
6	4.315713000	-0.808147000	-1.468263000
6	5.405609000	-0.246074000	-2.353453000
15	-0.485675000	-0.079064000	-0.624193000
6	-1.992757000	-0.988243000	-0.323321000
6	0.834852000	-0.818147000	0.308823000
6	-0.762679000	1.584530000	-0.043732000
6	-2.085683000	-2.021099000	0.614098000
6	-3.281692000	-2.685377000	0.806155000
6	-4.411271000	-2.330294000	0.063463000
6	-4.333303000	-1.293362000	-0.869402000
6	-3.128312000	-0.631869000	-1.052310000
6	1.224125000	-2.130762000	0.004013000
6	2.227980000	-2.743608000	0.719785000
6	2.879062000	-2.050007000	1.749155000
6	2.505445000	-0.741742000	2.055951000
6	1.484141000	-0.136634000	1.332776000
6	-0.143865000	2.661073000	-0.673195000
6	-0.317651000	3.955921000	-0.200869000
6	-1.120993000	4.177334000	0.917937000
6	-1.746217000	3.098498000	1.556329000
6	-1.568868000	1.817378000	1.079077000
8	-5.532375000	-3.031539000	0.309267000
8	-1.352230000	5.392362000	1.445387000
8	3.858715000	-2.717050000	2.383582000
6	-6.696714000	-2.718809000	-0.435283000
6	-0.742861000	6.516515000	0.834055000
6	4.561473000	-2.052219000	3.419129000
1	-1.043618000	-0.164540000	-2.968998000
1	4.180902000	-0.198249000	-0.573114000
1	4.548091000	-1.828586000	-1.163044000
1	6.355122000	-0.233025000	-1.816119000
1	5.162853000	0.772132000	-2.655312000
1	5.525353000	-0.859298000	-3.247446000
1	-1.220785000	-2.305817000	1.199836000
1	-3.365201000	-3.486698000	1.528624000
1	-5.196889000	-0.998470000	-1.447724000
1	-3.079436000	0.180790000	-1.769121000
1	0.738204000	-2.662302000	-0.806634000
1	2.541554000	-3.754494000	0.494386000
1	3.000640000	-0.189655000	2.841415000
1	1.203218000	0.882333000	1.569870000
1	0.489961000	2.486753000	-1.534147000
1	0.169794000	4.776225000	-0.707406000
1	-2.368354000	3.295010000	2.419671000
1	-2.060816000	0.988839000	1.575625000
1	-7.471960000	-3.398549000	-0.093287000
1	-7.013480000	-1.689218000	-0.255910000
1	-6.530106000	-2.867687000	-1.504233000
1	-1.052688000	7.384647000	1.408873000
1	0.345717000	6.434614000	0.859177000
1	-1.075404000	6.628758000	-0.199950000
1	5.304276000	-2.754318000	3.786839000
1	5.062689000	-1.158687000	3.041041000
1	3.889696000	-1.775912000	4.234506000

3_TS_Pani3_3

6	0.132342000	0.038499000	2.411373000
6	-1.041160000	0.011671000	3.047234000
6	-2.352913000	-0.163574000	2.513147000
8	-3.030054000	-1.185332000	2.587537000
8	-2.878272000	0.996809000	2.028204000
6	-4.241663000	0.953613000	1.602765000
6	-4.342420000	0.433518000	0.186996000
15	0.423381000	-0.024444000	0.653949000
6	0.507036000	1.629469000	-0.013759000
6	2.018676000	-0.772874000	0.362706000
6	-0.811285000	-0.951788000	-0.223460000
6	1.307498000	1.914765000	-1.128169000
6	1.322361000	3.182513000	-1.670813000
6	0.536111000	4.195838000	-1.108877000
6	-0.262834000	3.923010000	0.002372000
6	-0.270251000	2.643093000	0.541320000
6	2.175555000	-1.937813000	-0.393438000
6	3.428468000	-2.492430000	-0.570573000
6	4.551327000	-1.894055000	0.007734000
6	4.407345000	-0.727840000	0.763794000
6	3.145429000	-0.178207000	0.932512000
6	-1.257052000	-2.165384000	0.298696000
6	-2.133532000	-2.968223000	-0.415788000
6	-2.568860000	-2.556098000	-1.677634000
6	-2.120273000	-1.342395000	-2.211615000
6	-1.251661000	-0.549725000	-1.490819000
8	0.614271000	5.401982000	-1.697388000
8	-3.419444000	-3.262011000	-2.441491000
8	5.731305000	-2.500624000	-0.214014000
6	-0.167095000	6.459712000	-1.168642000
6	-3.923520000	-4.486204000	-1.934448000
6	6.897126000	-1.932423000	0.356532000
1	1.077365000	0.184870000	2.940287000
1	-4.813739000	0.332098000	2.291529000
1	-4.600127000	1.980012000	1.672967000
1	-3.764289000	1.063826000	-0.491642000
1	-5.382006000	0.431868000	-0.144270000
1	-3.955866000	-0.585657000	0.133179000
1	1.920234000	1.137452000	-1.569627000
1	1.937428000	3.417348000	-2.529611000
1	-0.870417000	4.693771000	0.454165000
1	-0.889060000	2.433273000	1.405311000
1	1.315201000	-2.412339000	-0.849120000
1	3.562606000	-3.392703000	-1.155754000
1	5.262807000	-0.247658000	1.216348000
1	3.042764000	0.733134000	1.511625000
1	-0.926629000	-2.482658000	1.280197000
1	-2.475483000	-3.897467000	0.015967000
1	-2.474902000	-1.040722000	-3.188424000
1	-0.917287000	0.391195000	-1.910669000
1	0.033523000	7.329360000	-1.787892000
1	-1.231628000	6.220556000	-1.212952000
1	0.114577000	6.677585000	-0.136455000
1	-4.590885000	-4.881655000	-2.694895000
1	-3.116237000	-5.199162000	-1.755125000
1	-4.481327000	-4.326705000	-1.009438000
1	6.827533000	-1.904151000	1.445938000
1	7.725251000	-2.572253000	0.065153000
1	7.068912000	-0.923142000	-0.023670000

3_Ppfp3_1

6	0.122310000	-0.169324000	2.355872000
6	-1.107998000	-0.411733000	2.827409000
6	-2.265829000	-0.686832000	2.020787000
8	-2.844193000	-1.761367000	1.918681000
8	-2.760501000	0.439064000	1.441538000
6	-4.006933000	0.321246000	0.754480000
6	-4.226330000	1.611093000	0.002990000
15	0.520554000	-0.078699000	0.626744000
6	-0.449846000	-1.249059000	-0.301699000
6	0.246325000	1.565555000	-0.027131000
6	2.259595000	-0.423349000	0.372129000
6	-1.351530000	-0.845873000	-1.283891000
6	-2.122669000	-1.788556000	-1.949791000
6	-1.966068000	-3.119489000	-1.611212000
6	-1.080462000	-3.552758000	-0.637800000
6	-0.324934000	-2.603213000	0.025105000

6	0.753130000	1.885266000	-1.291084000
6	0.542912000	3.142533000	-1.830152000
6	-0.168041000	4.064821000	-1.078621000
6	-0.672359000	3.784741000	0.177073000
6	-0.461430000	2.517496000	0.703964000
6	3.176370000	0.293192000	1.148759000
6	4.536893000	0.089564000	0.992556000
6	4.956683000	-0.831894000	0.048076000
6	4.079049000	-1.548473000	-0.742989000
6	2.717255000	-1.337679000	-0.575536000
9	-2.706500000	-4.033275000	-2.251545000
9	6.272155000	-1.033009000	-0.108106000
9	-0.368406000	5.285381000	-1.593064000
1	0.970948000	0.063412000	2.999751000
1	-3.968166000	-0.534570000	0.078357000
1	-4.801278000	0.136542000	1.480921000
1	-3.435655000	1.763762000	-0.735118000
1	-5.182886000	1.583254000	-0.519048000
1	-4.228984000	2.462343000	0.684976000
1	-1.469288000	0.203815000	-1.523048000
1	-2.835318000	-1.505063000	-2.711854000
1	-1.006913000	-4.605742000	-0.404262000
1	0.357928000	-2.910293000	0.809007000
1	1.312631000	1.147960000	-1.856344000
1	0.920501000	3.416814000	-2.805426000
1	-1.214394000	4.544391000	0.723309000
1	-0.851377000	2.266476000	1.681351000
1	2.828213000	1.020739000	1.872941000
1	5.265127000	0.629114000	1.582074000
1	4.459073000	-2.252047000	-1.470769000
1	2.017289000	-1.887522000	-1.191067000

3_Ppfp3_2

6	-0.000882000	0.078189000	-2.292972000
6	1.201537000	0.485963000	-2.715356000
6	2.268718000	0.910513000	-1.857614000
8	2.346325000	2.009174000	-1.310720000
8	3.288586000	0.024122000	-1.796149000
6	4.360001000	0.367859000	-0.915958000
6	5.377013000	-0.743879000	-0.975054000
15	-0.501923000	-0.023757000	-0.589116000
6	0.850256000	-0.488190000	0.477426000
6	-1.202957000	1.522612000	-0.025591000
6	-1.795938000	-1.251160000	-0.421930000
6	1.176254000	0.247867000	1.615762000
6	2.191360000	-0.183973000	2.457977000
6	2.855615000	-1.353233000	2.136230000
6	2.560606000	-2.104145000	1.010114000
6	1.552842000	-1.658020000	0.172451000
6	-2.120880000	1.527584000	1.028769000
6	-2.646549000	2.722931000	1.490146000
6	-2.239876000	3.895817000	0.876104000
6	-1.336366000	3.924845000	-0.169896000
6	-0.814124000	2.721905000	-0.622623000
6	-2.913166000	-1.134862000	-1.256119000
6	-3.947492000	-2.051494000	-1.174339000
6	-3.844950000	-3.073900000	-0.246089000
6	-2.760580000	-3.213533000	0.598597000
6	-1.728575000	-2.289543000	0.505481000
9	3.835056000	-1.777148000	2.945721000
9	-4.843713000	-3.963162000	-0.162185000
9	-2.751195000	5.054183000	1.313911000
1	-0.816882000	-0.170525000	-2.972430000
1	4.792249000	1.320525000	-1.225218000
1	3.961652000	0.496342000	0.094571000
1	5.772395000	-0.851750000	-1.985456000
1	6.206460000	-0.524342000	-0.302366000
1	4.931864000	-1.693402000	-0.675019000
1	0.644715000	1.162336000	1.846367000
1	2.469083000	0.369816000	3.344016000
1	3.116545000	-3.007898000	0.802090000
1	1.308635000	-2.218001000	-0.723011000
1	-2.427966000	0.595858000	1.489121000
1	-3.359486000	2.757504000	2.302355000
1	-1.056044000	4.869150000	-0.615724000
1	-0.097492000	2.713376000	-1.433881000
1	-2.982320000	-0.321076000	-1.968703000
1	-4.819746000	-1.983933000	-1.809617000
1	-2.731525000	-4.027027000	1.310345000
1	-0.875109000	-2.382747000	1.164125000

3_Ppfp3_3

6	0.011784000	0.099366000	-2.309548000
6	1.246311000	0.347423000	-2.753140000
6	2.431968000	0.641878000	-2.015933000
8	2.735091000	1.763937000	-1.606445000
8	3.307765000	-0.389836000	-1.950722000
6	4.614214000	-0.084757000	-1.453520000
6	4.658267000	-0.099243000	0.057488000
15	-0.474186000	0.012536000	-0.601336000

6	0.745269000	-0.812922000	0.403127000
6	-0.812318000	1.630493000	0.083321000
6	-2.003153000	-0.911464000	-0.487162000
6	1.090850000	-0.337780000	1.667661000
6	1.928919000	-1.081800000	2.486674000
6	2.393317000	-2.296014000	2.016968000
6	2.078269000	-2.791574000	0.762033000
6	1.252269000	-2.035196000	-0.050738000
6	-1.717005000	1.764839000	1.140714000
6	-1.959477000	3.009618000	1.697675000
6	-1.285985000	4.101434000	1.176262000
6	-0.387274000	4.002539000	0.130408000
6	-0.151026000	2.751138000	-0.420160000
6	-3.077770000	-0.479075000	-1.271625000
6	-4.287117000	-1.152014000	-1.234342000
6	-4.398233000	-2.252225000	-0.400494000
6	-3.359196000	-2.701911000	0.391635000
6	-2.150541000	-2.020471000	0.343953000
9	3.197508000	-3.021466000	2.804672000
9	-5.566713000	-2.906470000	-0.358430000
9	-1.521801000	5.308589000	1.707663000
1	-0.842060000	-0.030891000	-2.975784000
1	5.263938000	-0.853543000	-1.869960000
1	4.924094000	0.884826000	-1.842888000
1	4.399983000	-1.086629000	0.440242000
1	5.663679000	0.148034000	0.402092000
1	3.962143000	0.633627000	0.466159000
1	0.708129000	0.611778000	2.019416000
1	2.218350000	-0.735359000	3.469063000
1	2.482871000	-3.740635000	0.438622000
1	0.992622000	-2.394415000	-1.039363000
1	-2.236386000	0.896989000	1.529248000
1	-2.656042000	3.143247000	2.513728000
1	0.108967000	4.888394000	-0.241266000
1	0.556533000	2.644370000	-1.233234000
1	-2.972249000	0.391463000	-1.908729000
1	-5.132313000	-0.839030000	-1.831577000
1	-3.499807000	-3.564271000	1.028443000
1	-1.327519000	-2.358372000	0.960495000

3_PCy3_1

6	-0.158760000	0.355089000	-1.957260000
6	-1.397399000	0.046759000	-2.351400000
6	-2.445542000	-0.563546000	-1.606243000
8	-2.728090000	-1.760816000	-1.604976000
8	-3.227359000	0.342942000	-0.950481000
6	-4.375234000	-0.170796000	-0.273380000
6	-5.534801000	-0.374993000	-1.222108000
15	0.567845000	0.213569000	-0.336782000
6	0.575228000	1.845432000	0.460174000
6	-0.376521000	-0.899644000	0.730090000
6	2.292949000	-0.266895000	-0.603689000
6	1.425750000	2.864938000	-0.302234000
6	1.380655000	4.222007000	0.393417000
6	-0.049713000	4.720547000	0.544641000
6	-0.886096000	3.704633000	1.310562000
6	-0.863785000	2.352506000	0.607637000
6	-0.196888000	-2.370113000	0.344935000
6	-1.189816000	-3.227592000	1.119191000
6	-0.997849000	-3.048071000	2.619782000
6	-1.125571000	-1.580603000	3.007902000
6	-0.138957000	-0.711550000	2.232637000
6	3.100275000	-0.435210000	0.683295000
6	4.580086000	-0.592212000	0.347456000
6	4.800634000	-1.765089000	-0.600405000
6	3.970998000	-1.603823000	-1.868887000
6	2.489202000	-1.470206000	-1.531674000
1	0.566336000	0.795263000	-2.648647000
1	-4.111052000	-1.103805000	0.229418000
1	-4.616186000	0.576336000	0.482546000
1	-5.281207000	-1.116983000	-1.978231000
1	-6.411340000	-0.723028000	-0.673657000
1	-5.790059000	0.561544000	-1.719320000
1	1.011736000	1.706074000	1.457007000
1	-1.419872000	-0.620053000	0.532813000
1	2.686946000	0.605850000	-1.145146000
1	2.461488000	2.527212000	-0.373267000
1	1.037623000	2.962385000	-1.322869000
1	1.832612000	4.123844000	1.387053000
1	1.984141000	4.940072000	-0.164796000
1	-0.064243000	5.688550000	1.049090000
1	-0.487083000	4.866165000	-0.449328000
1	-0.481260000	3.592020000	2.322984000
1	-1.916574000	4.049952000	1.411338000
1	-1.480064000	1.629590000	1.148112000
1	-1.301429000	2.451873000	-0.391528000
1	-0.324402000	-2.502856000	-0.731299000
1	0.821633000	-2.675363000	0.610223000
1	-2.203987000	-2.925348000	0.835081000

1	-1.074106000	-4.276058000	0.838319000
1	-1.720059000	-3.651048000	3.173519000
1	0.000512000	-3.405054000	2.898336000
1	-2.142784000	-1.236696000	2.783665000
1	-0.970196000	-1.449517000	4.080409000
1	0.885388000	-1.004165000	2.483575000
1	-0.262382000	0.332208000	2.524975000
1	2.947433000	0.412064000	1.357473000
1	2.749189000	-1.334532000	1.201924000
1	4.932985000	0.329079000	-0.129829000
1	5.158748000	-0.723547000	1.263581000
1	5.860007000	-1.860076000	-0.846163000
1	4.502756000	-2.691633000	-0.096232000
1	4.298881000	-0.705990000	-2.404773000
1	4.123871000	-2.452740000	-2.537614000
1	2.158110000	-2.383088000	-1.032121000
1	1.890736000	-1.358441000	-2.437870000

3_PCy3_2

6	0.188024000	0.433096000	-1.884963000
6	1.487001000	0.589668000	-2.148799000
6	2.613583000	0.572730000	-1.283526000
8	3.094345000	1.547306000	-0.702711000
8	3.237861000	-0.639981000	-1.249474000
6	4.534092000	-0.675714000	-0.650764000
6	5.590292000	-0.154435000	-1.599819000
15	-0.595893000	0.144424000	-0.311675000
6	-1.153047000	1.722937000	0.389919000
6	-2.069681000	-0.842514000	-0.676331000
6	0.548721000	-0.601131000	0.873779000
6	0.055679000	2.641005000	0.602975000
6	-0.379092000	3.964292000	1.221049000
6	-1.409321000	4.654492000	0.337719000
6	-2.612863000	3.747722000	0.120791000
6	-2.200195000	2.411707000	-0.489334000
6	-1.816843000	-2.089558000	-1.530390000
6	-3.155653000	-2.665260000	-1.981546000
6	-4.026180000	-3.009418000	-0.778612000
6	-4.247080000	-1.785615000	0.102538000
6	-2.916707000	-1.186706000	0.548782000
6	0.137143000	-0.456743000	2.343334000
6	1.257440000	-0.982331000	3.236736000
6	1.596751000	-2.430790000	2.909725000
6	1.971965000	-2.574446000	1.440278000
6	0.845053000	-2.067365000	0.549232000
1	-0.567849000	0.512007000	-2.671808000
1	4.523895000	-0.103044000	0.278065000
1	4.708902000	-1.724469000	-0.412603000
1	6.577409000	-0.231149000	-1.141610000
1	5.593302000	-0.734999000	-2.523087000
1	5.401697000	0.891008000	-1.842207000
1	-1.608526000	1.494609000	1.361495000
1	-2.647212000	-0.151415000	-1.308151000
1	1.474468000	-0.025889000	0.735688000
1	0.811277000	2.155985000	1.225523000
1	0.526973000	2.828172000	-0.367635000
1	-0.816362000	3.776614000	2.208603000
1	0.492854000	4.603531000	1.370031000
1	-1.721923000	5.602418000	0.779882000
1	-0.951818000	4.883690000	-0.631088000
1	-3.098014000	3.559999000	1.085486000
1	-3.351365000	4.230996000	-0.521508000
1	-3.081460000	1.777765000	-0.607406000
1	-1.774287000	2.578410000	-1.485891000
1	-1.186439000	-1.848217000	-2.388410000
1	-1.291631000	-2.843806000	-0.941068000
1	-3.672474000	-1.926372000	-2.604145000
1	-2.989580000	-3.549156000	-2.599933000
1	-4.983902000	-3.419406000	-1.104688000
1	-3.526774000	-3.787338000	-0.189690000
1	-4.801053000	-1.028176000	-0.463691000
1	-4.851463000	-2.043189000	0.974183000
1	-2.374028000	-1.919864000	1.156424000
1	-3.083222000	-0.303709000	1.171894000
1	-0.072768000	0.584054000	2.593808000
1	-0.777605000	-1.029502000	2.526465000
1	2.147316000	-0.361390000	3.080724000
1	0.972114000	-0.879714000	4.285319000
1	2.408190000	-2.784048000	3.548816000
1	0.724026000	-3.060048000	3.119324000
1	2.874299000	-1.987384000	1.236677000
1	2.199153000	-3.614470000	1.198785000
1	-0.056429000	-2.656665000	0.750554000
1	1.096557000	-2.184534000	-0.507168000

3_PCy3_3

6	0.200804000	0.464983000	-1.818955000
6	1.489872000	0.736500000	-2.035437000
6	2.636752000	0.714819000	-1.202060000
8	3.028574000	1.644173000	-0.491377000
8	3.399576000	-0.404237000	-1.367529000
6	4.708966000	-0.378251000	-0.799236000
6	5.673456000	0.377294000	-1.686259000
15	-0.544672000	-0.003081000	-0.267639000
6	-1.947983000	-1.145742000	-0.506059000
6	0.667807000	-0.806774000	0.797912000
6	-1.140343000	1.504920000	0.542807000
6	-3.081281000	-0.942294000	0.507116000
6	-4.137417000	-2.028634000	0.337990000
6	-4.693447000	-2.020365000	-1.079624000
6	-3.571439000	-2.214396000	-2.090500000
6	-2.493909000	-1.146637000	-1.938195000
6	0.133999000	-1.064133000	2.209774000
6	1.259937000	-1.598354000	3.089691000
6	1.858991000	-2.869543000	2.499423000
6	2.336843000	-2.634144000	1.071206000
6	1.198403000	-2.113699000	0.200793000
6	0.017650000	2.358713000	1.063550000
6	-0.521984000	3.612338000	1.745453000
6	-1.404948000	4.420138000	0.802967000
6	-2.545789000	3.563139000	0.269551000
6	-1.996399000	2.325314000	-0.429593000
1	-0.547450000	0.624935000	-2.595841000
1	4.666707000	0.062795000	0.197481000
1	5.000638000	-1.424204000	-0.706672000
1	6.676770000	0.351821000	-1.258444000
1	5.711152000	-0.071615000	-2.679519000
1	5.364640000	1.418007000	-1.782252000
1	-1.514094000	-2.137311000	-0.323006000
1	1.500634000	-0.094252000	0.864756000
1	-1.759762000	1.189969000	1.391281000
1	-2.694176000	-0.941181000	1.528138000
1	-3.548868000	0.033250000	0.337906000
1	-3.684551000	-3.005192000	0.543217000
1	-4.935878000	-1.883068000	1.067906000
1	-5.452102000	-2.796231000	-1.198131000
1	-5.183872000	-1.057932000	-1.265577000
1	-3.115587000	-3.198646000	-1.933888000
1	-3.961456000	-2.199631000	-3.109822000
1	-1.686379000	-1.328094000	-2.648925000
1	-2.915268000	-0.161686000	-2.169079000
1	-0.293559000	-0.157235000	2.644256000
1	-0.666753000	-1.810935000	2.152003000
1	2.039360000	-0.831737000	3.164363000
1	0.888855000	-1.781671000	4.099726000
1	2.680198000	-3.226721000	3.123743000
1	1.094071000	-3.654541000	2.493636000
1	3.143653000	-1.892487000	1.074604000
1	2.743294000	-3.553344000	0.644881000
1	0.394638000	-2.857883000	0.191851000
1	1.530234000	-1.964305000	-0.826458000
1	0.636654000	1.791190000	1.762522000
1	0.661641000	2.634944000	0.221636000
1	-1.110035000	3.315285000	2.621594000
1	0.309659000	4.219602000	2.107384000
1	-1.794698000	5.304584000	1.310681000
1	-0.800273000	4.770923000	-0.040595000
1	-3.188202000	3.252197000	1.101368000
1	-3.166683000	4.135231000	-0.422279000
1	-1.362792000	2.638991000	-1.266290000
1	-2.806718000	1.721748000	-0.846237000

3_PBu3_1

6	-0.382995000	-0.564310000	-1.818879000
6	-1.690663000	-0.320454000	-1.977606000
6	-2.520497000	0.314247000	-0.997425000
8	-2.947877000	1.463938000	-1.039899000
8	-2.903196000	-0.533609000	-0.002899000
6	-3.832098000	-0.017285000	0.951511000
6	-4.088609000	-1.106233000	1.963310000
15	0.559405000	-0.199152000	-0.356296000
6	0.339696000	-1.368867000	0.999299000
6	2.294538000	-0.200143000	-0.809765000
6	0.126156000	1.392023000	0.347227000
6	1.043659000	-2.714657000	0.829958000
6	0.583757000	-3.522924000	-0.379141000
6	-0.904660000	-3.839868000	-0.351712000
6	0.288856000	2.557131000	-0.623613000
6	0.128041000	3.907596000	0.069442000
6	-1.239828000	4.089730000	0.712676000
6	3.232747000	0.232556000	0.311942000
6	4.695091000	0.052708000	-0.072976000
6	5.634537000	0.510362000	1.031354000
1	0.217841000	-1.057981000	-2.585185000
1	-3.408716000	0.874749000	1.420717000
1	-4.748435000	0.282541000	0.440009000
1	-4.792168000	-0.756781000	2.719063000
1	-4.509553000	-1.988889000	1.481150000
1	-3.161998000	-1.393708000	2.463279000

1	0.711759000	-0.857943000	1.892451000
1	-0.739947000	-1.486361000	1.125870000
1	2.521056000	-1.219948000	-1.140710000
1	2.399930000	0.445589000	-1.686138000
1	-0.899289000	1.314166000	0.719784000
1	0.770112000	1.517395000	1.224722000
1	2.123560000	-2.549379000	0.777352000
1	0.862575000	-3.297687000	1.737035000
1	0.831459000	-2.986448000	-1.301448000
1	1.156214000	-4.452870000	-0.406904000
1	-1.173115000	-4.343323000	0.580931000
1	-1.511828000	-2.935852000	-0.432389000
1	-1.182929000	-4.494631000	-1.178167000
1	1.277355000	2.505044000	-1.090446000
1	-0.453069000	2.458652000	-1.420166000
1	0.912201000	4.017793000	0.825297000
1	0.291793000	4.696505000	-0.667933000
1	-2.034088000	3.910863000	-0.014715000
1	-1.391304000	3.394781000	1.541626000
1	-1.356896000	5.099527000	1.107982000
1	3.048617000	1.283316000	0.558854000
1	3.023699000	-0.344734000	1.219509000
1	4.873316000	-1.001292000	-0.305535000
1	4.893437000	0.612233000	-0.991581000
1	5.483150000	1.568388000	1.255857000
1	5.456596000	-0.052479000	1.950350000
1	6.679806000	0.373743000	0.752686000

3_PBu3_2

6	0.328366000	-0.221039000	1.598155000
6	1.659371000	-0.111572000	1.658513000
6	2.571470000	0.192422000	0.606884000
8	2.932386000	1.320546000	0.267342000
8	3.147615000	-0.919628000	0.067301000
6	4.266859000	-0.709958000	-0.796843000
6	5.532330000	-0.478971000	-0.002127000
15	-0.609230000	-0.010775000	0.105128000
6	-0.122947000	-1.138809000	-1.208545000
6	-2.347562000	-0.252442000	0.464619000
6	-0.375039000	1.638064000	-0.561003000
6	-0.651676000	-2.564983000	-1.065769000
6	-0.199922000	-3.279203000	0.201436000
6	-0.604591000	-4.744784000	0.187971000
6	-0.794016000	2.737837000	0.410422000
6	-0.638338000	4.133185000	-0.187854000
6	0.805170000	4.479861000	-0.523676000
6	-3.254324000	0.063810000	-0.723471000
6	-4.694585000	-0.379767000	-0.486212000
6	-5.329193000	0.295121000	0.721114000
1	-0.289797000	-0.421220000	2.474832000
1	4.339117000	-1.618153000	-1.393864000
1	4.062454000	0.130571000	-1.460179000
1	5.728937000	-1.323811000	0.658846000
1	6.382526000	-0.363204000	-0.675883000
1	5.443111000	0.425160000	0.599793000
1	-0.465777000	-0.689303000	-2.144624000
1	0.970984000	-1.128304000	-1.225712000
1	-2.464387000	-1.293429000	0.785696000
1	-2.580118000	0.374197000	1.329658000
1	0.687760000	1.708250000	-0.808051000
1	-0.940531000	1.697031000	-1.496742000
1	-1.746392000	-2.559025000	-1.110620000
1	-0.312730000	-3.134600000	-1.935351000
1	0.885538000	-3.181906000	0.303372000
1	-0.630183000	-2.787595000	1.079733000
1	-0.149147000	-5.264414000	-0.657848000
1	-0.297500000	-5.255574000	1.100910000
1	-1.688392000	-4.847188000	0.094733000
1	-1.836277000	2.583479000	0.706995000
1	-0.186728000	2.661418000	1.317930000
1	-1.259295000	4.210186000	-1.085775000
1	-1.030240000	4.860928000	0.526179000
1	1.199783000	3.846060000	-1.319701000
1	0.894689000	5.515208000	-0.854700000
1	1.446429000	4.347479000	0.350927000
1	-3.231831000	1.141888000	-0.913591000
1	-2.866031000	-0.424595000	-1.622659000
1	-5.280141000	-0.158671000	-1.381116000
1	-4.714807000	-1.466265000	-0.357252000
1	-5.254202000	1.382653000	0.639704000
1	-6.385236000	0.038096000	0.808089000
1	-4.841393000	-0.003691000	1.650520000

3_PBu3_3

6	0.273381000	0.903455000	1.549976000
6	1.598373000	1.083478000	1.482246000
6	2.417117000	0.795605000	0.345343000
8	2.561964000	1.524038000	-0.637508000
8	3.151701000	-0.339495000	0.488132000
6	4.141683000	-0.590309000	-0.512830000
6	5.385817000	0.236104000	-0.274614000
15	-0.703329000	0.232812000	0.226816000
6	-0.870155000	1.335983000	-1.188347000
6	0.021772000	-1.271889000	-0.427839000
6	-2.340518000	-0.135210000	0.859139000
6	-1.863974000	2.483798000	-1.006692000
6	-1.522349000	3.446861000	0.126015000
6	-0.183979000	4.143971000	-0.069472000
6	-3.238932000	-0.858162000	-0.139112000
6	-4.658795000	-1.014896000	0.388505000
6	-5.552017000	-1.757538000	-0.592390000
6	0.166807000	-2.369885000	0.621656000
6	0.648717000	-3.687310000	0.020995000
6	2.068265000	-3.608484000	-0.520633000
1	-0.320107000	1.197276000	2.417225000
1	4.362747000	-1.653582000	-0.435082000
1	3.720103000	-0.391261000	-1.499509000
1	5.797092000	0.028310000	0.713876000
1	6.143819000	-0.008342000	-1.020234000
1	5.160064000	1.299347000	-0.345993000
1	0.139648000	1.696744000	-1.402630000
1	-1.171408000	0.703743000	-2.028860000
1	-0.626376000	-1.596166000	-1.248878000
1	0.983142000	-0.999537000	-0.872569000
1	-2.210917000	-0.723151000	1.772153000
1	-2.778253000	0.822212000	1.161596000
1	-1.901180000	3.040429000	-1.947054000
1	-2.864273000	2.069170000	-0.851894000
1	-2.317972000	4.192397000	0.194248000
1	-1.524062000	2.911575000	1.081667000
1	-0.000258000	4.871853000	0.721730000
1	0.646742000	3.435310000	-0.063228000
1	-0.164399000	4.674496000	-1.025018000
1	-3.262868000	-0.310058000	-1.087642000
1	-2.821158000	-1.846312000	-0.357839000
1	-4.626792000	-1.545692000	1.344328000
1	-5.071345000	-0.023301000	0.596392000
1	-6.567663000	-1.864033000	-0.210219000
1	-5.606561000	-1.227198000	-1.545649000
1	-5.162295000	-2.758214000	-0.791243000
1	0.867626000	-2.037402000	1.393241000
1	-0.798706000	-2.527798000	1.111735000
1	0.598386000	-4.460517000	0.790740000
1	-0.039957000	-3.990978000	-0.773556000
1	2.397055000	-4.573093000	-0.909410000
1	2.153589000	-2.880723000	-1.330570000
1	2.759877000	-3.307467000	0.269240000

Transition States (TS)

1_TS_PMe3_1

15	-2.279081000	-0.062984000	-0.025281000
6	-2.559284000	0.390179000	1.696762000
6	-3.906815000	-0.642408000	-0.578199000
6	-1.309842000	-1.575923000	0.100152000
1	-2.959245000	-0.451267000	2.265442000
1	-1.613238000	0.700411000	2.144670000
1	-3.827783000	-1.047417000	-1.587292000
1	-4.607246000	0.192975000	-0.595223000
1	-1.798523000	-2.280963000	0.775816000
1	-1.198849000	-2.033989000	-0.882354000
1	-4.291867000	-1.417248000	0.089138000
1	-0.319909000	-1.342324000	0.495016000
1	-3.261160000	1.223575000	1.750572000
6	-0.787793000	1.576478000	-0.628965000
6	0.379238000	1.334278000	0.083615000
6	1.373210000	0.472812000	-0.477902000
8	1.306977000	-0.107352000	-1.559868000
8	2.465834000	0.332481000	0.316988000
6	3.508312000	-0.498886000	-0.187490000
6	4.619614000	-0.509959000	0.832739000
1	-1.439927000	2.386311000	-0.325009000
1	-0.785587000	1.374797000	-1.692484000
1	3.117773000	-1.503432000	-0.362960000
1	3.851695000	-0.108012000	-1.147294000
1	5.439259000	-1.138304000	0.483603000
1	5.004041000	0.497276000	0.996139000
1	4.265862000	-0.904481000	1.785811000
1	0.528587000	1.716396000	1.084381000

1_TS_PMe3_2

15	2.079499000	0.097371000	-0.103422000
6	2.581402000	-0.214489000	1.599751000
6	3.554314000	0.844635000	-0.849921000
6	0.938494000	1.483332000	0.059782000
1	2.917418000	0.702765000	2.086822000
1	1.731493000	-0.617373000	2.154303000

1	3.322269000	1.180033000	-1.861013000
1	4.352371000	0.103946000	-0.906761000
1	1.409942000	2.286660000	0.629742000
1	0.651083000	1.855858000	-0.922997000
1	3.899934000	1.697440000	-0.260765000
1	0.043772000	1.158103000	0.593813000
1	3.389969000	-0.946478000	1.619820000
6	0.765530000	-1.747001000	-0.468432000
6	-0.383249000	-1.554344000	0.288347000
6	-1.463916000	-0.810645000	-0.279652000
8	-1.502552000	-0.345241000	-1.417043000
8	-2.501559000	-0.639063000	0.582973000
6	-3.576816000	0.183755000	0.133462000
6	-3.238821000	1.650932000	0.284922000
1	1.518218000	-2.448436000	-0.128582000
1	0.688674000	-1.654554000	-1.543908000
1	-3.811523000	-0.058153000	-0.903145000
1	-4.427070000	-0.087177000	0.758130000
1	-4.090125000	2.266318000	-0.009559000
1	-2.992319000	1.880074000	1.322831000
1	-2.388783000	1.912597000	-0.346061000
1	-0.436543000	-1.834798000	1.331679000

1_TS_PMe3_3

15	-2.270184000	-0.066299000	-0.125968000
6	-2.723000000	1.056175000	1.209859000
6	-3.855392000	-0.808414000	-0.603642000
6	-1.417339000	-1.401908000	0.730134000
1	-3.239288000	0.525058000	2.011601000
1	-1.819669000	1.515227000	1.615931000
1	-3.686380000	-1.592129000	-1.342348000
1	-4.497797000	-0.047701000	-1.047944000
1	-2.025118000	-1.765365000	1.561299000
1	-1.218151000	-2.222592000	0.041410000
1	-4.360153000	-1.238740000	0.264734000
1	-0.468979000	-1.034075000	1.124545000
1	-3.373464000	1.843661000	0.826626000
6	-0.615526000	1.164805000	-1.138072000
6	0.431828000	1.243258000	-0.229645000
6	1.439007000	0.227885000	-0.232143000
8	1.483516000	-0.749937000	-0.976037000
8	2.397263000	0.436020000	0.710580000
6	3.481972000	-0.490089000	0.740955000
6	4.525264000	-0.148151000	-0.299437000
1	-1.250408000	2.028107000	-1.296058000
1	-0.488287000	0.533641000	-2.008259000
1	3.895199000	-0.415734000	1.746238000
1	3.099096000	-1.499929000	0.592221000
1	5.365629000	-0.840344000	-0.230147000
1	4.101093000	-0.218833000	-1.300730000
1	4.900656000	0.864483000	-0.146467000
1	0.471658000	2.009719000	0.532468000

1_TS_PPhMe2_1

15	0.826605000	0.565179000	-0.033849000
6	0.846227000	2.258472000	-0.641977000
6	0.004697000	-0.344858000	-1.353453000
1	1.332563000	2.333223000	-1.615404000
1	-0.192048000	2.582326000	-0.739748000
1	0.465726000	-0.115162000	-2.315117000
1	0.054289000	-1.417048000	-1.170093000
1	-1.043612000	-0.041538000	-1.381160000
1	1.344514000	2.918597000	0.068226000
6	-0.717855000	0.760474000	1.633635000
6	-1.947713000	0.979655000	1.025929000
6	-2.752795000	-0.144076000	0.657478000
8	-2.465807000	-1.328402000	0.820348000
8	-3.928683000	0.211348000	0.079053000
6	-4.791515000	-0.858087000	-0.301737000
6	-6.029593000	-0.252043000	-0.914636000
1	-0.205238000	1.586672000	2.112193000
1	-0.543903000	-0.203789000	2.094599000
1	-4.272346000	-1.507459000	-1.009656000
1	-5.034495000	-1.458348000	0.577229000
1	-6.717661000	-1.040621000	-1.219906000
1	-6.540231000	0.391372000	-0.197537000
1	-5.775476000	0.341620000	-1.793329000
1	-2.280655000	1.970725000	0.748736000
6	2.539037000	0.003369000	-0.082295000
6	2.797573000	-1.367821000	0.031831000
6	3.615717000	0.891300000	-0.141335000
6	4.101260000	-1.839258000	0.064453000
6	4.921881000	0.415953000	-0.100225000
6	5.168563000	-0.947559000	-0.000668000
1	1.971994000	-2.067939000	0.096932000
1	3.440916000	1.956427000	-0.226183000
1	4.286229000	-2.902857000	0.144393000
1	5.747295000	1.114656000	-0.151571000
1	6.186083000	-1.315236000	0.027923000

1_TS_PPhMe2_2

15	-0.692000000	0.676424000	0.126696000
6	-0.815314000	2.188062000	1.094539000
6	0.222179000	-0.436134000	1.211113000
1	-1.270641000	2.010652000	2.069751000
1	0.200053000	2.563078000	1.241016000
1	-0.240178000	-0.459446000	2.198941000
1	0.251557000	-1.441990000	0.795312000
1	1.243722000	-0.062413000	1.309168000
1	-1.384999000	2.945887000	0.556373000
6	0.794758000	1.344016000	-1.470626000
6	2.036480000	1.409727000	-0.851154000
6	2.824830000	0.219401000	-0.768260000
8	2.523598000	-0.878774000	-1.231944000
8	3.990427000	0.400586000	-0.092475000
6	4.781819000	-0.763524000	0.141033000
6	4.249451000	-1.553961000	1.316293000
1	0.260961000	2.260057000	-1.696711000
1	0.616419000	0.535676000	-2.168156000
1	4.806945000	-1.373475000	-0.761916000
1	5.786197000	-0.393209000	0.342270000
1	4.892812000	-2.411906000	1.516427000
1	4.218657000	-0.931530000	2.211882000
1	3.243654000	-1.918785000	1.105292000
1	2.366532000	2.291265000	-0.318272000
6	-2.356410000	-0.012073000	0.059059000
6	-2.512331000	-1.329840000	-0.387335000
6	-3.494692000	0.742927000	0.350343000
6	-3.776351000	-1.884369000	-0.519107000
6	-4.761355000	0.186365000	0.209031000
6	-4.905926000	-1.126286000	-0.222120000
1	-1.637009000	-1.921251000	-0.634144000
1	-3.399031000	1.764978000	0.694987000
1	-3.881998000	-2.907020000	-0.857981000
1	-5.635865000	0.780369000	0.442485000
1	-5.892819000	-1.557664000	-0.328680000

1_TS_PPhMe2_3

15	-0.837833000	0.611308000	0.048886000
6	-0.971145000	2.349059000	0.494634000
6	-0.082796000	-0.138491000	1.502261000
1	-1.523843000	2.487537000	1.424733000
1	0.043545000	2.730379000	0.626899000
1	-0.618202000	0.162609000	2.403847000
1	-0.087935000	-1.224251000	1.419845000
1	0.951079000	0.204856000	1.572160000
1	-1.448900000	2.917224000	-0.303709000
6	0.820091000	0.732640000	-1.514435000
6	1.990786000	1.058729000	-0.841477000
6	2.811221000	0.009816000	-0.318851000
8	2.582496000	-1.195701000	-0.395657000
8	3.922306000	0.475416000	0.311335000
6	4.839490000	-0.500359000	0.804047000
6	5.749363000	-1.001383000	-0.295373000
1	0.312568000	1.491658000	-2.098211000
1	0.719209000	-0.273891000	-1.900538000
1	5.409647000	0.007675000	1.580977000
1	4.284263000	-1.322283000	1.256338000
1	6.470280000	-1.713231000	0.109125000
1	5.169132000	-1.500281000	-1.071251000
1	6.299006000	-0.173142000	-0.744342000
1	2.262135000	2.084099000	-0.629553000
6	-2.524488000	-0.025550000	0.028219000
6	-2.711321000	-1.412493000	-0.011494000
6	-3.642423000	0.809412000	-0.033319000
6	-3.987158000	-1.950025000	-0.088922000
6	-4.920166000	0.267268000	-0.119674000
6	-5.096563000	-1.110409000	-0.144398000
1	-1.850618000	-2.072104000	0.016465000
1	-3.522985000	1.885257000	-0.007583000
1	-4.117576000	-3.024408000	-0.110555000
1	-5.778785000	0.925376000	-0.161986000
1	-6.092288000	-1.529694000	-0.208691000

1_TS_PPh2Me_1

6	-0.832261000	-0.766378000	1.712661000
6	-2.038942000	-0.217630000	1.278625000
6	-2.957000000	-1.030202000	0.546694000
8	-2.804705000	-2.211411000	0.235963000
8	-4.092549000	-0.367195000	0.200735000
6	-5.071819000	-1.122929000	-0.507043000
6	-6.239246000	-0.209783000	-0.788912000
1	-0.269386000	-0.261121000	2.488348000
1	-0.756432000	-1.847697000	1.740010000
1	-5.374181000	-1.981401000	0.096038000
1	-4.635055000	-1.508674000	-1.430555000
1	-5.926913000	0.641619000	-1.394501000
1	-7.013915000	-0.751955000	-1.331598000
1	-6.668887000	0.165605000	0.140329000
1	-2.278011000	0.825083000	1.436634000
15	0.670800000	-0.395767000	0.090591000

6	0.714121000	1.395304000	0.027943000
6	2.391661000	-0.921448000	-0.097090000
6	3.244965000	-0.326084000	-1.030457000
6	4.548921000	-0.778896000	-1.169174000
6	5.010257000	-1.828268000	-0.377786000
6	4.167904000	-2.423506000	0.552248000
6	2.860885000	-1.968958000	0.694331000
6	1.659861000	2.085548000	0.794539000
6	1.644607000	3.470606000	0.847273000
6	0.676547000	4.184889000	0.144710000
6	-0.271646000	3.508033000	-0.609925000
6	-0.255226000	2.117417000	-0.670942000
1	2.885437000	0.492502000	-1.644169000
1	5.206841000	-0.315733000	-1.893273000
1	6.028593000	-2.178659000	-0.486987000
1	4.526494000	-3.237015000	1.169421000
1	2.201812000	-2.427497000	1.422736000
1	2.412045000	1.532615000	1.346445000
1	2.385970000	3.994942000	1.436383000
1	0.664566000	5.266387000	0.187313000
1	-1.026125000	4.059059000	-1.156502000
1	-0.995250000	1.600225000	-1.268809000
6	-0.140689000	-0.907832000	-1.430764000
1	-0.027224000	-1.986072000	-1.540305000
1	-1.203947000	-0.672058000	-1.379194000
1	0.302204000	-0.404108000	-2.291784000

1_TS_PPh2Me_2

6	-0.857756000	-0.774090000	-1.880238000
6	-1.969898000	-1.462544000	-1.408857000
6	-3.045757000	-0.726417000	-0.831912000
8	-3.109195000	0.500819000	-0.727898000
8	-4.049304000	-1.518710000	-0.371049000
6	-5.115012000	-0.870509000	0.320976000
6	-4.734263000	-0.582485000	1.756665000
1	-0.106278000	-1.303699000	-2.453427000
1	-0.987973000	0.264697000	-2.167833000
1	-5.953714000	-1.563831000	0.271775000
1	-5.386001000	0.045654000	-0.203600000
1	-3.896510000	0.114420000	1.795632000
1	-5.576677000	-0.138313000	2.288711000
1	-4.451254000	-1.503415000	2.268586000
1	-1.981677000	-2.541185000	-1.331566000
15	0.518170000	-0.199289000	-0.133492000
6	2.083109000	-1.052557000	0.064166000
6	0.829754000	1.565456000	0.030885000
6	2.076686000	2.120019000	0.324120000
6	2.221458000	3.499050000	0.414599000
6	1.126663000	4.333473000	0.218059000
6	-0.118720000	3.786269000	-0.073059000
6	-0.267653000	2.409721000	-0.176079000
6	2.310327000	-1.976647000	1.085101000
6	3.512404000	-2.673714000	1.142160000
6	4.494743000	-2.454527000	0.184869000
6	4.272637000	-1.539419000	-0.841089000
6	3.071054000	-0.851216000	-0.907344000
1	2.932940000	1.478726000	0.491876000
1	3.191028000	3.921389000	0.645937000
1	1.244054000	5.407078000	0.290434000
1	-0.973689000	4.431852000	-0.227840000
1	-1.236938000	1.978930000	-0.412162000
1	1.554275000	-2.155339000	1.839296000
1	3.679933000	-3.387455000	1.938500000
1	5.429586000	-2.997794000	0.232851000
1	5.032928000	-1.369706000	-1.592452000
1	2.892353000	-0.148033000	-1.714150000
6	-0.506829000	-0.628500000	1.274592000
1	-1.391144000	0.010518000	1.240853000
1	-0.833514000	-1.666968000	1.188838000
1	0.016034000	-0.473239000	2.217996000

1_TS_PPh2Me_3

6	-0.430019000	-2.296004000	-0.087740000
6	-1.650484000	-1.926302000	0.483156000
6	-2.529318000	-1.065097000	-0.241929000
8	-2.352159000	-0.634660000	-1.380453000
8	-3.651455000	-0.745162000	0.461782000
6	-4.597934000	0.098287000	-0.189603000
6	-5.520245000	-0.698708000	-1.085842000
1	0.122375000	-3.124314000	0.342858000
1	-0.336817000	-2.213486000	-1.164127000
1	-4.068184000	0.864061000	-0.757726000
1	-5.156120000	0.580532000	0.612901000
1	-6.044606000	-1.464275000	-0.512333000
1	-6.262771000	-0.042050000	-1.541703000
1	-4.951652000	-1.182093000	-1.879982000
1	-1.903342000	-2.185503000	1.502512000
15	1.026218000	-0.732996000	0.510742000
6	2.723312000	-0.380012000	-0.004611000
6	0.139029000	0.811334000	0.360835000
6	-0.875820000	1.134257000	1.264002000
6	-1.680128000	2.248659000	1.041990000
6	-1.466600000	3.051070000	-0.070237000
6	-0.440764000	2.745961000	-0.963651000
6	0.352105000	1.630100000	-0.753589000
6	3.326372000	-1.207477000	-0.950111000
6	4.638688000	-0.974325000	-1.349428000
6	5.348283000	0.088511000	-0.806350000
6	4.750035000	0.921691000	0.136582000
6	3.442343000	0.689594000	0.536715000
1	-1.046662000	0.519426000	2.138491000
1	-2.472743000	2.485583000	1.740501000
1	-2.092605000	3.917450000	-0.241778000
1	-0.268098000	3.374767000	-1.827516000
1	1.134909000	1.382399000	-1.461635000
1	2.766796000	-2.032771000	-1.375145000
1	5.101967000	-1.619384000	-2.084634000
1	6.368522000	0.272598000	-1.117722000
1	5.304057000	1.751386000	0.556459000
1	2.972325000	1.340539000	1.265762000
6	1.136811000	-1.063695000	2.276882000
1	0.160133000	-1.377918000	2.648814000
1	1.846464000	-1.876987000	2.432190000
1	1.467682000	-0.183509000	2.829609000

1_TS_PPh3_1

15	-0.819154000	-0.016985000	-0.376930000
6	-1.510147000	1.585816000	0.030209000
6	-2.151393000	-1.216132000	-0.268365000
6	0.396924000	-0.449444000	0.853480000
6	-3.014568000	-1.311603000	-1.363597000
6	-4.083080000	-2.197493000	-1.338082000
6	-4.286902000	-3.005719000	-0.224532000
6	-3.425520000	-2.922406000	0.864199000
6	-2.362139000	-2.028463000	0.846886000
6	0.815579000	0.413473000	1.868156000
6	1.856019000	0.044489000	2.708462000
6	2.492724000	-1.183018000	2.538394000
6	2.078890000	-2.048193000	1.533284000
6	1.037729000	-1.681478000	0.688212000
6	-2.819277000	1.761205000	0.482574000
6	-3.299025000	3.039666000	0.737507000
6	-2.477882000	4.145822000	0.546317000
6	-1.173556000	3.975956000	0.093072000
6	-0.690821000	2.702168000	-0.175547000
1	-2.848135000	-0.686586000	-2.234543000
1	-4.750137000	-2.262656000	-2.187761000
1	-5.115306000	-3.702140000	-0.206376000
1	-3.584466000	-3.550478000	1.731227000
1	-1.697793000	-1.959836000	1.699584000
1	0.332364000	1.373887000	1.998119000
1	2.172459000	0.715188000	3.496822000
1	3.308341000	-1.464142000	3.192543000
1	2.568810000	-3.004258000	1.400644000
1	0.709375000	-2.357091000	-0.093354000
1	-3.460653000	0.902090000	0.638029000
1	-4.313907000	3.171266000	1.090116000
1	-2.855576000	5.140226000	0.746854000
1	-0.534561000	4.836138000	-0.059365000
1	0.323399000	2.557519000	-0.536573000
6	0.311025000	-0.057588000	-2.341299000
6	1.594755000	-0.574094000	-2.167178000
6	2.550183000	0.241993000	-1.496925000
8	2.360991000	1.402431000	-1.122191000
8	3.735406000	-0.376527000	-1.260372000
6	4.682217000	0.353212000	-0.485407000
6	5.825464000	-0.577808000	-0.165909000
1	-0.416203000	-0.627083000	-2.910813000
1	0.212695000	1.018686000	-2.450020000
1	4.198957000	0.716792000	0.424439000
1	5.022492000	1.225113000	-1.048752000
1	6.580214000	-0.055202000	0.422182000
1	5.471481000	-1.434378000	0.410543000
1	6.294331000	-0.945390000	-1.079276000
1	1.814767000	-1.622296000	-2.313282000

1_TS_PPh3_2

15	-0.797449000	0.003856000	-0.353242000
6	-1.317689000	1.671546000	0.045039000
6	-2.263999000	-1.031872000	-0.361930000
6	0.274102000	-0.586653000	0.943836000
6	-3.048628000	-1.016485000	-1.518460000
6	-4.208936000	-1.775522000	-1.587995000
6	-4.584345000	-2.567583000	-0.507993000
6	-3.802603000	-2.594288000	0.642081000
6	-2.647115000	-1.826926000	0.719405000
6	0.716741000	0.204893000	2.005709000
6	1.652047000	-0.293645000	2.900676000
6	2.159165000	-1.581175000	2.740011000
6	1.718155000	-2.376772000	1.690093000

6	0.781898000	-1.880795000	0.790272000
6	-2.626476000	2.001150000	0.401690000
6	-2.963438000	3.325046000	0.653882000
6	-1.999977000	4.323154000	0.554764000
6	-0.694941000	3.999571000	0.196972000
6	-0.353467000	2.680316000	-0.068125000
1	-2.747246000	-0.405373000	-2.362599000
1	-4.813614000	-1.755961000	-2.485291000
1	-5.484438000	-3.166138000	-0.563350000
1	-4.095041000	-3.209953000	1.482873000
1	-2.044379000	-1.843630000	1.619185000
1	0.335338000	1.210838000	2.129273000
1	1.987589000	0.322715000	3.724722000
1	2.893332000	-1.963821000	3.437513000
1	2.105492000	-3.379739000	1.565822000
1	0.432648000	-2.502305000	-0.026436000
1	-3.378846000	1.226161000	0.485621000
1	-3.978809000	3.576390000	0.932256000
1	-2.266822000	5.353297000	0.752971000
1	0.055139000	4.775729000	0.116464000
1	0.660486000	2.415807000	-0.354378000
6	0.447270000	-0.134142000	-2.245283000
6	1.642752000	-0.813692000	-2.015452000
6	2.657745000	-0.141582000	-1.276377000
8	2.587762000	1.018996000	-0.863538000
8	3.749539000	-0.910695000	-1.023493000
6	4.779599000	-0.324453000	-0.230972000
6	5.714049000	0.515621000	-1.073211000
1	-0.308586000	-0.592213000	-2.874891000
1	0.491658000	0.948405000	-2.323569000
1	5.309460000	-1.163582000	0.219613000
1	4.327534000	0.271263000	0.563473000
1	6.517690000	0.918952000	-0.455274000
1	6.159697000	-0.086347000	-1.866278000
1	5.173216000	1.347009000	-1.524503000
1	1.740360000	-1.876005000	-2.190140000

1_TS_PPh3_3

15	-0.767170000	-0.027110000	-0.326852000
6	-2.482845000	-0.520713000	-0.503723000
6	0.008481000	-1.095602000	0.872210000
6	-0.761041000	1.638352000	0.342090000
6	1.348639000	-0.826964000	1.178888000
6	2.043760000	-1.655809000	2.049991000
6	1.416759000	-2.766363000	2.603743000
6	0.089672000	-3.043834000	2.292396000
6	-0.614064000	-2.214195000	1.429169000
6	-0.576877000	1.913671000	1.698008000
6	-0.595237000	3.227403000	2.148629000
6	-0.803894000	4.270839000	1.253170000
6	-0.992329000	4.002146000	-0.098285000
6	-0.964324000	2.691324000	-0.554298000
6	-2.741239000	-1.709768000	-1.194813000
6	-4.047047000	-2.134735000	-1.392957000
6	-5.106384000	-1.365730000	-0.920970000
6	-4.856327000	-0.176401000	-0.245916000
6	-3.549766000	0.246913000	-0.034553000
1	1.830808000	0.049769000	0.758484000
1	3.074744000	-1.433643000	2.294242000
1	1.960706000	-3.414419000	3.278862000
1	-0.401754000	-3.904268000	2.727907000
1	-1.649365000	-2.432495000	1.198670000
1	-0.420842000	1.103398000	2.399524000
1	-0.449980000	3.435705000	3.200769000
1	-0.816824000	5.293267000	1.608024000
1	-1.150914000	4.812432000	-0.797814000
1	-1.101718000	2.482321000	-1.609670000
1	-1.913927000	-2.302603000	-1.570906000
1	-4.238575000	-3.059205000	-1.922019000
1	-6.125624000	-1.691971000	-1.082707000
1	-5.679642000	0.423059000	0.120548000
1	-3.360753000	1.172507000	0.495500000
6	0.411361000	-0.238683000	-2.248640000
6	1.664335000	-0.826612000	-2.077323000
6	2.756085000	-0.014441000	-1.659852000
8	2.699815000	1.185409000	-1.381092000
8	3.931965000	-0.695645000	-1.578013000
6	5.063672000	0.029057000	-1.102069000
6	5.087289000	0.074977000	0.409949000
1	-0.365873000	-0.799985000	-2.757672000
1	0.387965000	0.828383000	-2.448132000
1	5.055051000	1.034486000	-1.522684000
1	5.931821000	-0.504672000	-1.487399000
1	5.979861000	0.597639000	0.757175000
1	4.210452000	0.602015000	0.788337000
1	5.099139000	-0.935647000	0.820882000
1	1.806492000	-1.896518000	-2.143358000

1_TS_Pani3_1

15	0.621727000	-0.031088000	0.562654000
6	-0.660995000	-0.711748000	-0.470140000
6	0.878887000	1.671746000	0.075669000
6	2.127647000	-0.930495000	0.193550000
6	-0.094485000	2.602807000	0.444479000
6	0.036082000	3.946892000	0.116938000
6	1.170261000	4.377602000	-0.572372000
6	2.157849000	3.455293000	-0.932251000
6	2.012001000	2.118850000	-0.613729000
6	2.330471000	-1.639604000	-0.996965000
6	3.520966000	-2.300211000	-1.227970000
6	4.544383000	-2.262492000	-0.274896000
6	4.358860000	-1.559553000	0.915645000
6	3.151337000	-0.908993000	1.139639000
6	-1.041999000	-2.039492000	-0.225295000
6	-2.094005000	-2.612207000	-0.914042000
6	-2.805459000	-1.860573000	-1.851871000
6	-2.429119000	-0.543767000	-2.118292000
6	-1.366504000	0.019439000	-1.422498000
1	-0.974311000	2.265465000	0.985715000
1	-0.737266000	4.643602000	0.407338000
1	3.030068000	3.810512000	-1.466070000
1	2.783283000	1.415300000	-0.904202000
1	1.547723000	-1.672298000	-1.745441000
1	3.686034000	-2.852347000	-2.144253000
1	5.133653000	-1.522623000	1.667982000
1	3.007814000	-0.372792000	2.071835000
1	-0.498468000	-2.628321000	0.505088000
1	-2.398118000	-3.633814000	-0.725585000
1	-2.960889000	0.049948000	-2.848195000
1	-1.092669000	1.047271000	-1.627619000
6	-0.169074000	-0.267500000	2.718937000
6	-1.344606000	-1.008242000	2.759456000
6	-2.563523000	-0.379274000	2.370760000
8	-2.686475000	0.795315000	2.016472000
8	-3.639930000	-1.209364000	2.431218000
6	-4.900506000	-0.663501000	2.049721000
6	-5.085180000	-0.696846000	0.548136000
1	0.740014000	-0.691626000	3.131125000
1	-0.245995000	0.810525000	2.822113000
1	-4.982736000	0.355614000	2.428216000
1	-5.645746000	-1.283152000	2.547640000
1	-5.011915000	-1.719858000	0.174447000
1	-6.067026000	-0.300911000	0.282162000
1	-4.320024000	-0.090273000	0.062538000
1	-1.342258000	-2.076886000	2.923157000
8	5.671354000	-2.933900000	-0.583691000
6	6.735017000	-2.919530000	0.350418000
1	7.536661000	-3.508992000	-0.085833000
1	7.089862000	-1.901584000	0.526670000
1	6.432153000	-3.367698000	1.299354000
8	-3.851825000	-2.476752000	-2.441941000
6	-4.698140000	-1.700851000	-3.268577000
1	-5.512096000	-2.351453000	-3.576710000
1	-5.104070000	-0.847159000	-2.718611000
1	-4.169773000	-1.342261000	-4.154800000
8	1.394253000	5.660108000	-0.925123000
6	0.417446000	6.626122000	-0.584477000
1	0.784352000	7.579773000	-0.953906000
1	-0.541244000	6.400311000	-1.057080000
1	0.282375000	6.685564000	0.497805000

1_TS_Pani3_2

15	0.490469000	0.022166000	0.680329000
6	1.172646000	1.579979000	0.124313000
6	1.783536000	-1.211582000	0.535945000
6	-0.809552000	-0.443595000	-0.445098000
6	2.728092000	-1.299504000	1.568385000
6	3.754951000	-2.220039000	1.512975000
6	3.854023000	-3.093065000	0.424311000
6	2.918355000	-3.023754000	-0.607676000
6	1.895618000	-2.083433000	-0.544213000
6	-1.181261000	0.302163000	-1.570409000
6	-2.244980000	-0.094582000	-2.356182000
6	-2.974785000	-1.242101000	-2.027355000
6	-2.613347000	-2.000718000	-0.915980000
6	-1.540680000	-1.590535000	-0.131613000
6	2.446396000	1.714027000	-0.421250000
6	2.938972000	2.959663000	-0.796346000
6	2.144140000	4.092901000	-0.628696000
6	0.862531000	3.969768000	-0.080540000
6	0.388016000	2.731148000	0.301036000
1	2.655585000	-0.633788000	2.421793000
1	4.487922000	-2.292042000	2.306054000
1	2.977689000	-3.688827000	-1.457500000
1	1.176374000	-2.033862000	-1.353096000
1	-0.632136000	1.198445000	-1.832513000
1	-2.539459000	0.476086000	-3.227577000
1	-3.160704000	-2.892759000	-0.646667000
1	-1.258913000	-2.186927000	0.728691000

1	3.070572000	0.839154000	-0.560696000
1	3.931817000	3.034229000	-1.216474000
1	0.262879000	4.862135000	0.044929000
1	-0.604141000	2.637540000	0.734109000
6	-0.505966000	0.176532000	2.761485000
6	-1.818652000	-0.278822000	2.747696000
6	-2.825169000	0.556281000	2.180292000
8	-2.645199000	1.681510000	1.710017000
8	-4.064780000	-0.004481000	2.201620000
6	-5.120349000	0.743971000	1.603008000
6	-5.143320000	0.552878000	0.102634000
1	0.248369000	-0.392072000	3.294104000
1	-0.342486000	1.249814000	2.764458000
1	-5.007824000	1.797643000	1.858789000
1	-6.035865000	0.368794000	2.059399000
1	-5.965617000	1.119436000	-0.337379000
1	-4.206721000	0.899723000	-0.335552000
1	-5.277240000	-0.501453000	-0.148265000
1	-2.073979000	-1.298012000	3.002590000
8	-4.019559000	-1.534723000	-2.830078000
6	-4.798712000	-2.673857000	-2.518595000
1	-4.199237000	-3.586281000	-2.562464000
1	-5.248035000	-2.584489000	-1.526247000
1	-5.585659000	-2.724703000	-3.266072000
8	2.533622000	5.340636000	-0.961766000
6	3.825628000	5.511863000	-1.513021000
1	4.599028000	5.185311000	-0.813986000
1	3.930235000	4.961913000	-2.451180000
1	3.941132000	6.575012000	-1.705380000
8	4.877278000	-3.969852000	0.454745000
6	5.009491000	-4.879410000	-0.621804000
1	4.127952000	-5.518896000	-0.706982000
1	5.167807000	-4.352570000	-1.565478000
1	5.878991000	-5.492831000	-0.402455000

1_TS_Pani3_3

15	0.659659000	-0.046916000	0.564794000
6	-0.674421000	-0.638412000	-0.459785000
6	1.041026000	1.638064000	0.091821000
6	2.096867000	-1.047874000	0.185141000
6	0.183979000	2.649530000	0.530025000
6	0.424487000	3.981991000	0.215446000
6	1.552829000	4.316844000	-0.533241000
6	2.426827000	3.312417000	-0.962077000
6	2.172617000	1.990383000	-0.654161000
6	2.296951000	-1.666111000	-1.056348000
6	3.441955000	-2.397002000	-1.301450000
6	4.421834000	-2.524597000	-0.310124000
6	4.237055000	-1.917292000	0.931491000
6	3.074954000	-1.191523000	1.167103000
6	-1.033277000	-1.989215000	-0.338898000
6	-2.166520000	-2.481228000	-0.956785000
6	-2.989570000	-1.622119000	-1.688621000
6	-2.642171000	-0.277770000	-1.826872000
6	-1.493663000	0.201218000	-1.208241000
1	-0.692845000	2.385825000	1.114555000
1	-0.260576000	4.742828000	0.561622000
1	3.297998000	3.594307000	-1.539553000
1	2.857239000	1.223586000	-0.996945000
1	1.548056000	-1.569137000	-1.833457000
1	3.605369000	-2.878543000	-2.257027000
1	4.977207000	-2.010001000	1.713219000
1	2.932070000	-0.726359000	2.136555000
1	-0.412911000	-2.659230000	0.246696000
1	-2.449911000	-3.521651000	-0.862934000
1	-3.258455000	0.399955000	-2.400331000
1	-1.249081000	1.252090000	-1.303722000
6	-0.199542000	-0.308676000	2.672555000
6	-1.443785000	-0.930958000	2.592760000
6	-2.545297000	-0.162152000	2.116556000
8	-2.534137000	1.051120000	1.891424000
8	-3.667048000	-0.895568000	1.892693000
6	-4.780120000	-0.203215000	1.337149000
6	-5.815654000	-1.234760000	0.960890000
1	0.639331000	-0.847254000	3.099982000
1	-0.178722000	0.761325000	2.854955000
1	-4.452919000	0.368003000	0.465217000
1	-5.171662000	0.508295000	2.068318000
1	-5.405517000	-1.940738000	0.236176000
1	-6.144123000	-1.793549000	1.837907000
1	-6.685702000	-0.748824000	0.517586000
1	-1.546877000	-2.006284000	2.639040000
8	5.507751000	-3.252147000	-0.636712000
6	6.528088000	-3.402099000	0.333330000
1	6.946818000	-2.433807000	0.616585000
1	6.152227000	-3.909639000	1.224400000
1	7.303622000	-4.008721000	-0.125821000
8	-4.101473000	-2.168548000	-2.227109000
6	-5.043274000	-1.298203000	-2.824409000
1	-4.634526000	-0.823263000	-3.719211000
1	-5.898174000	-1.909034000	-3.101862000
1	-5.363112000	-0.524842000	-2.119816000
8	1.877564000	5.578378000	-0.882143000
6	1.020831000	6.626748000	-0.469322000
1	1.453580000	7.547505000	-0.850701000
1	0.017319000	6.501108000	-0.882138000
1	0.960098000	6.679970000	0.619984000

1_TS_Ppfp3_1

15	-0.651331000	-0.004618000	-0.465816000
6	-1.109768000	1.660381000	0.011606000
6	-2.131413000	-1.014885000	-0.368404000
6	0.511054000	-0.642007000	0.725797000
6	-2.994543000	-0.997576000	-1.467973000
6	-4.170631000	-1.732405000	-1.456727000
6	-4.454385000	-2.491634000	-0.335895000
6	-3.621148000	-2.541399000	0.767198000
6	-2.453907000	-1.791616000	0.746328000
6	1.080925000	0.131873000	1.738527000
6	2.085600000	-0.387255000	2.539763000
6	2.513161000	-1.683346000	2.304056000
6	1.971629000	-2.482799000	1.316545000
6	0.969213000	-1.947577000	0.518118000
6	-2.372804000	1.994081000	0.504949000
6	-2.679223000	3.311319000	0.815302000
6	-1.705608000	4.275393000	0.627149000
6	-0.445397000	3.983205000	0.137149000
6	-0.153829000	2.665367000	-0.181950000
1	-2.745343000	-0.402269000	-2.339264000
1	-4.852817000	-1.731098000	-2.295829000
1	-3.890312000	-3.151023000	1.619013000
1	-1.792498000	-1.811099000	1.603541000
1	0.746261000	1.148301000	1.901967000
1	2.537891000	0.193550000	3.332004000
1	2.336045000	-3.490998000	1.174737000
1	0.528505000	-2.556484000	-0.262613000
1	-3.122271000	1.226182000	0.651176000
1	-3.649722000	3.593380000	1.200373000
1	0.279142000	4.774998000	0.003716000
1	0.823486000	2.401570000	-0.575114000
6	0.448712000	-0.143197000	-2.443488000
6	1.663499000	-0.809584000	-2.285088000
6	2.710370000	-0.123111000	-1.606657000
8	2.646759000	1.038730000	-1.194183000
8	3.822360000	-0.877380000	-1.407881000
6	4.888348000	-0.274956000	-0.676426000
6	5.761859000	0.574273000	-1.572898000
1	-0.342422000	-0.616077000	-3.016120000
1	0.475327000	0.938757000	-2.535828000
1	5.453467000	-1.105943000	-0.254750000
1	4.474682000	0.317656000	0.141001000
1	6.595532000	0.987710000	-1.003423000
1	6.167234000	-0.024243000	-2.389783000
1	5.186060000	1.398669000	-1.992716000
1	1.761999000	-1.871426000	-2.461514000
9	-5.585350000	-3.213901000	-0.319387000
9	-1.998696000	5.550804000	0.928836000
9	3.492543000	-2.185446000	3.075140000

1_TS_Ppfp3_2

15	-0.683262000	-0.022797000	-0.494727000
6	-1.345860000	1.577589000	-0.038852000
6	-1.997113000	-1.225486000	-0.277324000
6	0.624789000	-0.446635000	0.638719000
6	-2.920920000	-1.364402000	-1.317297000
6	-3.978829000	-2.254781000	-1.210920000
6	-4.082761000	-3.009049000	-0.056148000
6	-3.183644000	-2.906368000	0.990053000
6	-2.137208000	-2.002739000	0.874232000
6	1.143670000	0.439362000	1.585025000
6	2.265600000	0.100521000	2.324622000
6	2.861872000	-1.128050000	2.092089000
6	2.373862000	-2.035926000	1.173131000
6	1.250076000	-1.681810000	0.437413000
6	-2.614683000	1.747143000	0.519039000
6	-3.090247000	3.018502000	0.806483000
6	-2.277564000	4.103025000	0.530305000
6	-1.016913000	3.974016000	-0.024553000
6	-0.556457000	2.699545000	-0.319873000
1	-2.814023000	-0.768398000	-2.216681000
1	-4.705450000	-2.375172000	-2.002699000
1	-3.310760000	-3.519417000	1.871917000
1	-1.427760000	-1.902508000	1.686043000
1	0.676962000	1.403424000	1.742559000
1	2.683016000	0.771097000	3.063354000
1	2.872191000	-2.985404000	1.031912000
1	0.847922000	-2.377470000	-0.289730000
1	-3.235793000	0.886397000	0.733944000
1	-4.068666000	3.174638000	1.240165000

1	-0.422843000	4.854965000	-0.226249000
1	0.425417000	2.562341000	-0.763012000
6	0.312340000	-0.085970000	-2.525864000
6	1.609549000	-0.587783000	-2.414293000
6	2.586835000	0.247307000	-1.801698000
8	2.401448000	1.412395000	-1.438183000
8	3.784129000	-0.359053000	-1.601405000
6	4.752219000	0.377568000	-0.859105000
6	5.862881000	-0.573359000	-0.487135000
1	-0.440337000	-0.673702000	-3.041342000
1	0.196921000	0.986885000	-2.650695000
1	4.278908000	0.801506000	0.029095000
1	5.122345000	1.208814000	-1.463968000
1	6.632033000	-0.047127000	0.078664000
1	5.477517000	-1.387166000	0.130311000
1	6.323244000	-1.002611000	-1.377633000
1	1.832122000	-1.636044000	-2.555402000
9	-5.097678000	-3.880377000	0.052390000
9	-2.734806000	5.334750000	0.808368000
9	3.962748000	-1.449166000	2.793549000

1_TS_Ppfp3_3

15	-0.626959000	-0.007178000	-0.494547000
6	0.602035000	-0.603950000	0.651140000
6	-1.110044000	1.642399000	0.011484000
6	-2.071749000	-1.058935000	-0.329090000
6	-0.195574000	2.674337000	-0.233564000
6	-0.503147000	3.982023000	0.111115000
6	-1.737998000	4.236555000	0.680362000
6	-2.670126000	3.244448000	0.924458000
6	-2.347379000	1.937656000	0.587141000
6	-2.267461000	-1.936176000	0.738774000
6	-3.407876000	-2.725023000	0.801475000
6	-4.342635000	-2.612497000	-0.211099000
6	-4.185562000	-1.752774000	-1.283820000
6	-3.035309000	-0.981054000	-1.340012000
6	1.058271000	0.129641000	1.748519000
6	2.054765000	-0.377930000	2.568266000
6	2.589717000	-1.617485000	2.263578000
6	2.167166000	-2.372865000	1.186654000
6	1.168652000	-1.853464000	0.373767000
1	0.762855000	2.440757000	-0.688133000
1	0.189501000	4.794301000	-0.062309000
1	-3.621395000	3.497144000	1.372777000
1	-3.063679000	1.148215000	0.777594000
1	-1.529506000	-2.005608000	1.528090000
1	-3.578546000	-3.412479000	1.618742000
1	-4.944018000	-1.703995000	-2.053174000
1	-2.886205000	-0.306210000	-2.175920000
1	0.632742000	1.100249000	1.970006000
1	2.420210000	0.171218000	3.425318000
1	2.617209000	-3.336782000	0.991769000
1	0.811517000	-2.434146000	-0.468108000
6	0.331511000	-0.075898000	-2.552889000
6	1.590374000	-0.672514000	-2.527138000
6	2.675262000	0.075849000	-1.989115000
8	2.597710000	1.224956000	-1.545353000
8	3.857595000	-0.593965000	-1.997116000
6	4.979294000	0.073378000	-1.421865000
6	4.985274000	-0.065689000	0.084205000
1	-0.483569000	-0.586017000	-3.055949000
1	0.291107000	1.006810000	-2.629649000
1	4.965491000	1.123095000	-1.715613000
1	5.854884000	-0.402145000	-1.862411000
1	5.859406000	0.433638000	0.505170000
1	4.088184000	0.388422000	0.506635000
1	5.017064000	-1.117621000	0.373201000
1	1.733006000	-1.722159000	-2.742081000
9	-2.047427000	5.502014000	1.006609000
9	3.568828000	-2.103251000	3.044931000
9	-5.447964000	-3.371779000	-0.152881000

1_TS_PCy3_1

6	-0.764886000	-0.085835000	-2.201295000
6	-1.984483000	-0.648981000	-1.855762000
6	-3.057308000	0.190181000	-1.426514000
8	-3.046073000	1.418884000	-1.353074000
8	-4.172273000	-0.508137000	-1.083989000
6	-5.293825000	0.264896000	-0.664491000
6	-6.414048000	-0.691681000	-0.338683000
15	0.714249000	-0.024215000	-0.345454000
6	0.437036000	-1.671467000	0.378176000
6	2.535275000	0.134495000	-0.574715000
6	0.226560000	1.177132000	0.946547000
6	0.742087000	-2.787899000	-0.623563000
6	0.358549000	-4.143010000	-0.036423000
6	1.082988000	-4.392527000	1.280944000
6	0.799093000	-3.273393000	2.275596000
6	1.186454000	-1.921031000	1.686312000
6	2.838711000	0.865856000	-1.887604000
6	4.336497000	0.885383000	-2.169017000
6	5.098879000	1.517162000	-1.011268000
6	4.801312000	0.781919000	0.289533000
6	3.303595000	0.778762000	0.580360000
6	0.163458000	2.574854000	0.321172000
6	-0.209445000	3.624042000	1.361307000
6	-1.546321000	3.278242000	2.004886000
6	-1.492852000	1.890287000	2.630452000
6	-1.115226000	0.831921000	1.598235000
1	-0.049143000	-0.660255000	-2.775385000
1	-0.727880000	0.982094000	-2.381504000
1	-2.130674000	-1.721451000	-1.836810000
1	-5.577900000	0.955518000	-1.461021000
1	-5.015615000	0.864296000	0.205344000
1	-6.685185000	-1.281789000	-1.214648000
1	-7.294020000	-0.137164000	-0.012029000
1	-6.120627000	-1.372946000	0.460626000
1	-0.644912000	-1.701922000	0.574197000
1	2.891587000	-0.899861000	-0.679799000
1	0.994186000	1.174637000	1.731075000
1	0.209304000	-2.616735000	-1.561903000
1	1.814370000	-2.788779000	-0.852386000
1	-0.723274000	-4.156500000	0.139235000
1	0.578516000	-4.938229000	-0.751662000
1	0.793158000	-5.358900000	1.698515000
1	2.162157000	-4.434366000	1.091980000
1	-0.271128000	-3.263427000	2.512326000
1	1.335031000	-3.445923000	3.211337000
1	2.263232000	-1.918211000	1.474925000
1	0.997366000	-1.116999000	2.402814000
1	2.298440000	0.393597000	-2.712330000
1	2.467389000	1.895405000	-1.813018000
1	4.684064000	-0.145347000	-2.305270000
1	4.537827000	1.418226000	-3.100626000
1	6.171767000	1.517639000	-1.214181000
1	4.790050000	2.563927000	-0.907847000
1	5.151868000	-0.253339000	0.205989000
1	5.341737000	1.238517000	1.121514000
1	2.965509000	1.814304000	0.703400000
1	3.096369000	0.259904000	1.519011000
1	1.117038000	2.822840000	-0.154618000
1	-0.599623000	2.561328000	-0.466502000
1	0.566817000	3.657628000	2.134930000
1	-0.247960000	4.612756000	0.899544000
1	-1.817446000	4.024995000	2.754166000
1	-2.326137000	3.292720000	1.234692000
1	-0.744443000	1.887580000	3.431941000
1	-2.452359000	1.636967000	3.086514000
1	-1.888494000	0.787702000	0.824534000
1	-1.070514000	-0.149051000	2.076127000

1_TS_PCy3_2

6	0.824943000	0.832376000	-1.865029000
6	2.024494000	1.195692000	-1.258400000
6	3.135687000	0.301323000	-1.235919000
8	3.219254000	-0.794807000	-1.790323000
8	4.182623000	0.794802000	-0.512580000
6	5.367844000	0.004366000	-0.475907000
6	6.217785000	0.226745000	-1.707659000
15	-0.673622000	0.008445000	-0.285525000
6	0.465918000	-0.438360000	1.051953000
6	-1.616043000	1.453606000	0.313303000
6	-1.856130000	-1.385968000	-0.472649000
6	-0.086467000	-0.523455000	2.472793000
6	1.071412000	-0.693592000	3.454441000
6	1.906551000	-1.924703000	3.116216000
6	2.401443000	-1.882085000	1.673775000
6	1.226215000	-1.723612000	0.715878000
6	-0.673242000	2.582796000	0.741579000
6	-1.469636000	3.773131000	1.265363000
6	-2.443091000	4.281613000	0.209662000
6	-3.372376000	3.163721000	-0.244985000
6	-2.575632000	1.971195000	-0.763209000
6	-3.063023000	-1.347705000	0.467896000
6	-3.904135000	-2.610059000	0.303114000
6	-4.362330000	-2.767100000	-1.141545000
6	-3.165060000	-2.804408000	-2.083132000
6	-2.308834000	-1.552335000	-1.927297000
1	0.124671000	1.603963000	-2.161794000
1	0.834263000	-0.030513000	-2.521536000
1	2.120469000	2.127913000	-0.716860000
1	5.098881000	-1.047806000	-0.372826000
1	5.896706000	0.319752000	0.423225000
1	6.481660000	1.280692000	-1.804877000
1	5.678655000	-0.084809000	-2.601944000
1	7.139310000	-0.353227000	-1.638991000
1	1.205980000	0.376640000	1.021877000
1	-2.205522000	1.145378000	1.187317000
1	-1.259612000	-2.271703000	-0.217515000

1	-0.673906000	0.362811000	2.726536000
1	-0.753741000	-1.390156000	2.545262000
1	1.706886000	0.198083000	3.401279000
1	0.694501000	-0.759481000	4.477419000
1	2.746691000	-2.013606000	3.808178000
1	1.287023000	-2.819613000	3.247800000
1	3.080437000	-1.030247000	1.541225000
1	2.967685000	-2.785450000	1.436214000
1	0.554539000	-2.581247000	0.837100000
1	1.567043000	-1.710248000	-0.321716000
1	0.031503000	2.235966000	1.501031000
1	-0.082294000	2.899535000	-0.125928000
1	-2.031206000	3.461365000	2.153628000
1	-0.790097000	4.568844000	1.577047000
1	-3.018850000	5.126059000	0.593813000
1	-1.874051000	4.645336000	-0.653686000
1	-3.986206000	2.837946000	0.602797000
1	-4.055585000	3.521423000	-1.017929000
1	-1.991301000	2.279325000	-1.639032000
1	-3.252624000	1.181212000	-1.093727000
1	-2.740594000	-1.231663000	1.504850000
1	-3.683442000	-0.477842000	0.226300000
1	-3.302388000	-3.481206000	0.586303000
1	-4.762274000	-2.577984000	0.977872000
1	-4.966005000	-3.669780000	-1.255463000
1	-5.001260000	-1.916709000	-1.407417000
1	-2.552845000	-3.682598000	-1.847695000
1	-3.492459000	-2.909205000	-3.119522000
1	-2.891972000	-0.674862000	-2.229056000
1	-1.437122000	-1.598001000	-2.584938000

1_TS_PCy3_3

6	0.516599000	0.384151000	2.341777000
6	1.778170000	-0.206080000	2.318587000
6	2.870333000	0.449661000	1.682182000
8	2.874598000	1.585699000	1.206669000
8	3.995358000	-0.323962000	1.636375000
6	5.093405000	0.198085000	0.893813000
6	4.896151000	-0.021153000	-0.591284000
15	-0.689205000	-0.022941000	0.379054000
6	0.513914000	-1.015093000	-0.549575000
6	-2.266844000	-0.963253000	0.407393000
6	-0.965303000	1.499858000	-0.590183000
6	0.653487000	-2.419492000	0.044904000
6	1.910320000	-3.079722000	-0.509894000
6	1.837793000	-3.159909000	-2.031845000
6	1.589422000	-1.789924000	-2.655629000
6	0.345334000	-1.130645000	-2.063936000
6	-3.053365000	-0.710601000	1.698558000
6	-4.280252000	-1.613331000	1.765017000
6	-5.167517000	-1.418413000	0.541748000
6	-4.382201000	-1.681322000	-0.737181000
6	-3.162229000	-0.768675000	-0.818595000
6	-1.889547000	2.451365000	0.176790000
6	-2.120530000	3.737894000	-0.608109000
6	-0.799456000	4.426980000	-0.922681000
6	0.126107000	3.483579000	-1.679960000
6	0.361759000	2.202450000	-0.887428000
1	-0.242486000	-0.010670000	3.004999000
1	0.459544000	1.458688000	2.197109000
1	1.929935000	-1.223976000	2.651250000
1	5.970232000	-0.339032000	1.253956000
1	5.215288000	1.257998000	1.117331000
1	3.994043000	0.491156000	-0.930894000
1	4.803507000	-1.085756000	-0.813670000
1	5.746861000	0.373116000	-1.149212000
1	1.463610000	-0.491421000	-0.349372000
1	-1.950455000	-2.014102000	0.428220000
1	-1.443010000	1.230207000	-1.541912000
1	0.673347000	-2.383458000	1.136605000
1	-0.217742000	-3.016419000	-0.247299000
1	2.779603000	-2.481870000	-0.209765000
1	2.039418000	-4.076571000	-0.083279000
1	2.752831000	-3.599040000	-2.435081000
1	1.013561000	-3.827822000	-2.308298000
1	2.452048000	-1.141262000	-2.459714000
1	1.493719000	-1.878887000	-3.739886000
1	-0.532450000	-1.751480000	-2.277029000
1	0.173097000	-0.156465000	-2.526486000
1	-2.409581000	-0.877138000	2.565471000
1	-3.372679000	0.336713000	1.733852000
1	-3.949304000	-2.657670000	1.802997000
1	-4.839767000	-1.419636000	2.682440000
1	-6.041155000	-2.071164000	0.595203000
1	-5.536834000	-0.386238000	0.527710000
1	-4.049782000	-2.725662000	-0.746592000
1	-5.015981000	-1.536982000	-1.614739000
1	-3.502441000	0.272153000	-0.855717000
1	-2.601017000	-0.954333000	-1.737292000
1	-2.844612000	1.969902000	0.396787000
1	-1.420260000	2.690040000	1.140056000
1	-2.631282000	3.492977000	-1.546724000
1	-2.779974000	4.403740000	-0.047637000
1	-0.970771000	5.340520000	-1.495850000
1	-0.317031000	4.720473000	0.016679000
1	-0.328595000	3.231623000	-2.645413000
1	1.080812000	3.969603000	-1.891121000
1	0.860314000	2.438631000	0.058101000
1	1.040563000	1.541277000	-1.432550000

1_TS_PBu3_1

6	-0.059291000	-0.481346000	1.602698000
6	0.992510000	-1.322685000	1.274439000
15	-0.966927000	0.227168000	-0.411298000
6	2.307445000	-0.784004000	1.099432000
8	2.644287000	0.389546000	1.243179000
8	3.219580000	-1.726338000	0.746326000
6	4.557080000	-1.269075000	0.560422000
6	5.399883000	-2.460617000	0.178779000
6	0.462113000	0.864992000	-1.319775000
6	-1.300581000	-1.359838000	-1.220231000
6	-2.356923000	1.302434000	-0.935080000
6	-2.373725000	-2.163553000	-0.494329000
6	0.825884000	2.291351000	-0.925179000
6	-2.742181000	2.322226000	0.134861000
6	2.123960000	2.751464000	-1.574180000
6	2.486028000	4.176581000	-1.186688000
6	-3.388946000	1.668078000	1.348502000
6	-3.752436000	2.679587000	2.424014000
6	-2.763589000	-3.429039000	-1.246173000
6	-3.813093000	-4.239153000	-0.501391000
1	-0.986923000	-0.894894000	1.977066000
1	0.174132000	0.508983000	1.976586000
1	0.836606000	-2.375189000	1.076248000
1	4.572185000	-0.503649000	-0.218927000
1	4.914295000	-0.806022000	1.482193000
1	5.379602000	-3.215649000	0.965222000
1	5.035551000	-2.912132000	-0.744501000
1	6.433979000	-2.151369000	0.025620000
1	1.298737000	0.191369000	-1.107489000
1	0.254955000	0.796112000	-2.393385000
1	-0.359028000	-1.917297000	-1.245251000
1	-1.588882000	-1.168680000	-2.259356000
1	-2.069916000	1.789351000	-1.872187000
1	-3.212548000	0.655797000	-1.153376000
1	-2.010618000	-2.432905000	0.503414000
1	-3.266496000	-1.544622000	-0.340937000
1	0.015048000	2.973960000	-1.204318000
1	0.932512000	2.348367000	0.163494000
1	-1.853817000	2.878940000	0.454721000
1	-3.434278000	3.055427000	-0.289716000
1	2.923030000	2.066815000	-1.272931000
1	2.032240000	2.668712000	-2.661745000
1	2.604442000	4.262936000	-0.104320000
1	3.418714000	4.498377000	-1.651767000
1	1.702048000	4.873887000	-1.491216000
1	-4.283091000	1.125961000	1.023575000
1	-2.705089000	0.916401000	1.759612000
1	-4.447780000	3.426652000	2.034623000
1	-4.221210000	2.203281000	3.285846000
1	-2.862870000	3.207015000	2.775226000
1	-1.867579000	-4.035543000	-1.408709000
1	-3.135903000	-3.153937000	-2.237508000
1	-4.089933000	-5.151783000	-1.047401000
1	-3.443988000	-4.542086000	0.480937000
1	-4.720001000	-3.650186000	-0.346644000

1_TS_PBu3_2

6	0.031446000	0.532890000	1.522302000
6	-0.972810000	1.386157000	1.089284000
15	0.979540000	-0.346459000	-0.395101000
6	-2.299926000	0.880773000	0.910191000
8	-2.686362000	-0.257360000	1.167225000
8	-3.151944000	1.812532000	0.403974000
6	-4.514721000	1.420170000	0.245472000
6	-5.272715000	1.522150000	1.550315000
6	-0.422268000	-0.931772000	-1.380698000
6	1.456735000	1.179290000	-1.250175000
6	2.337342000	-1.517788000	-0.781645000
6	2.546193000	1.949061000	-0.511925000
6	-0.885292000	-2.332727000	-0.995105000
6	2.641530000	-2.463144000	0.378621000
6	-1.984129000	-2.863126000	-1.913574000
6	-3.261640000	-2.035761000	-1.863042000
6	3.274486000	-1.739226000	1.559399000
6	3.549116000	-2.671556000	2.728685000
6	3.058236000	3.142410000	-1.307446000
6	4.124206000	3.920439000	-0.551863000
1	0.962969000	0.937899000	1.896991000

1	-0.257874000	-0.411521000	1.968209000
1	-0.765586000	2.408243000	0.800221000
1	-4.924883000	2.103453000	-0.497314000
1	-4.554350000	0.405075000	-0.152295000
1	-6.321360000	1.263970000	1.396031000
1	-4.853262000	0.839427000	2.288685000
1	-5.223480000	2.538910000	1.942175000
1	-1.224890000	-0.201100000	-1.242965000
1	-0.131710000	-0.897471000	-2.437596000
1	0.555890000	1.793023000	-1.348151000
1	1.780215000	0.919359000	-2.263811000
1	2.059880000	-2.066662000	-1.686675000
1	3.227675000	-0.927588000	-1.019845000
1	2.155648000	2.298151000	0.449765000
1	3.386455000	1.282543000	-0.280422000
1	-0.032830000	-3.019074000	-1.024368000
1	-1.249200000	-2.311772000	0.037105000
1	1.719196000	-2.955060000	0.708606000
1	3.314876000	-3.256009000	0.039792000
1	-1.604321000	-2.896223000	-2.939833000
1	-2.210452000	-3.894461000	-1.632061000
1	-4.043129000	-2.479656000	-2.481608000
1	-3.632447000	-1.965393000	-0.838457000
1	-3.096542000	-1.017624000	-2.222575000
1	4.204841000	-1.267001000	1.227104000
1	2.613093000	-0.925367000	1.879239000
1	4.010850000	-2.146345000	3.565515000
1	2.622513000	-3.124766000	3.087626000
1	4.219251000	-3.480942000	2.430276000
1	2.215221000	3.797342000	-1.547206000
1	3.457954000	2.789086000	-2.262650000
1	4.980144000	3.282032000	-0.321313000
1	4.487941000	4.772171000	-1.127815000
1	3.730339000	4.299424000	0.393771000

1_TS_PBu3_3

6	-0.357669000	0.650216000	1.762795000
6	-1.598840000	1.063503000	1.297674000
15	0.922584000	0.269566000	-0.116312000
6	-2.618152000	0.093475000	1.039664000
8	-2.535516000	-1.121209000	1.213858000
8	-3.764499000	0.645984000	0.563730000
6	-4.833074000	-0.258055000	0.293577000
6	-5.999514000	0.550728000	-0.217398000
6	-0.093694000	-0.849728000	-1.111212000
6	0.734543000	1.842477000	-0.997623000
6	2.647736000	-0.234683000	-0.459692000
6	1.350631000	3.021726000	-0.254773000
6	0.034097000	-2.314694000	-0.713834000
6	3.233169000	-1.147712000	0.613928000
6	-0.968252000	-3.193285000	-1.449731000
6	-0.838159000	-4.658902000	-1.066023000
6	4.638564000	-1.619556000	0.268414000
6	5.229264000	-2.506913000	1.353186000
6	1.265915000	4.317015000	-1.051129000
6	1.863467000	5.494626000	-0.297004000
1	0.320963000	1.369316000	2.202054000
1	-0.275175000	-0.350617000	2.170175000
1	-1.800027000	2.099986000	1.059951000
1	-4.505327000	-0.995435000	-0.442884000
1	-5.091453000	-0.798620000	1.206199000
1	-6.320012000	1.279429000	0.527772000
1	-5.730525000	1.083812000	-1.129854000
1	-6.840205000	-0.107114000	-0.438688000
1	-1.130047000	-0.517238000	-0.992731000
1	0.171571000	-0.707021000	-2.164753000
1	-0.339024000	2.002923000	-1.139051000
1	1.175467000	1.732825000	-1.994240000
1	2.675433000	-0.721328000	-1.441890000
1	3.245168000	0.679047000	-0.534932000
1	0.838105000	3.153427000	0.704372000
1	2.400139000	2.810500000	-0.019513000
1	1.050415000	-2.669203000	-0.919761000
1	-0.128385000	-2.414299000	0.364913000
1	3.250497000	-0.614118000	1.569825000
1	2.584444000	-2.017667000	0.761827000
1	-1.976592000	-2.833990000	-1.221378000
1	-0.828195000	-3.072687000	-2.528595000
1	-0.999926000	-4.792233000	0.005915000
1	-1.562223000	-5.281415000	-1.593232000
1	0.160351000	-5.035483000	-1.300031000
1	4.609102000	-2.160159000	-0.682616000
1	5.278592000	-0.746382000	0.108195000
1	4.608861000	-3.392050000	1.510213000
1	6.234536000	-2.844036000	1.097614000
1	5.287035000	-1.971295000	2.303355000
1	0.217446000	4.518276000	-1.290558000
1	1.782000000	4.182808000	-2.006507000
1	1.799953000	6.418384000	-0.873318000
1	1.341873000	5.653880000	0.649349000
1	2.916332000	5.314656000	-0.068134000

2_TS_PMe3_1

6	-1.670044000	2.603444000	0.284075000
6	-0.762353000	1.738578000	-0.108436000
6	0.482571000	1.449867000	-0.556996000
15	-1.976968000	-0.421095000	-0.034711000
6	1.327103000	0.593207000	0.231444000
8	1.021621000	0.098428000	1.310443000
8	2.514720000	0.336709000	-0.354728000
6	3.393608000	-0.535149000	0.359551000
6	4.656464000	-0.676517000	-0.451960000
6	-0.973840000	-1.433799000	-1.142983000
6	-2.164072000	-1.483518000	1.420534000
6	-3.622034000	-0.508591000	-0.793899000
1	-2.709514000	2.349436000	0.435023000
1	-1.362043000	3.628203000	0.469603000
1	3.594238000	-0.112810000	1.345591000
1	2.900393000	-1.498243000	0.505900000
1	5.354298000	-1.337812000	0.061698000
1	4.440407000	-1.099500000	-1.433353000
1	5.137820000	0.292020000	-0.589133000
1	0.773278000	1.687813000	-1.572439000
1	-3.610151000	0.003580000	-1.756941000
1	-3.936746000	-1.544655000	-0.943939000
1	-4.349864000	-0.009985000	-0.151838000
1	-1.445065000	-2.403451000	-1.317082000
1	-0.846043000	-0.922342000	-2.098452000
1	0.012671000	-1.596616000	-0.705860000
1	-1.188020000	-1.626079000	1.884549000
1	-2.584338000	-2.454851000	1.146916000
1	-2.821954000	-1.004493000	2.146726000

2_TS_PMe3_2

6	-1.365385000	2.616811000	0.538626000
6	-0.602472000	1.718441000	-0.041669000
6	0.538331000	1.370980000	-0.683832000
15	-1.986125000	-0.337674000	0.044201000
6	1.402445000	0.385696000	-0.091228000
8	1.199475000	-0.177465000	0.978302000
8	2.469631000	0.092799000	-0.865193000
6	3.397070000	-0.865835000	-0.348581000
6	4.351809000	-0.227460000	0.634550000
6	-1.229331000	-1.334323000	-1.257416000
6	-2.073648000	-1.491851000	1.437796000
6	-3.718126000	-0.239656000	-0.485781000
1	-2.393847000	2.436421000	0.816881000
1	-0.941620000	3.593393000	0.753508000
1	2.845596000	-1.684929000	0.113323000
1	3.927351000	-1.245860000	-1.220264000
1	5.077747000	-0.964237000	0.980613000
1	4.893238000	0.593522000	0.163288000
1	3.811109000	0.156649000	1.499069000
1	0.709006000	1.673415000	-1.709356000
1	-3.788194000	0.337623000	-1.408647000
1	-4.139775000	-1.234233000	-0.653210000
1	-4.308114000	0.267563000	0.279226000
1	-1.801249000	-2.248514000	-1.429943000
1	-1.186717000	-0.760315000	-2.184572000
1	-0.211050000	-1.605406000	-0.974091000
1	-2.587586000	-1.020725000	2.276598000
1	-1.061993000	-1.745185000	1.754877000
1	-2.609035000	-2.403566000	1.160027000

2_TS_PMe3_3

6	-1.769371000	2.569526000	0.363344000
6	-0.797527000	1.804199000	-0.079107000
6	0.447302000	1.644461000	-0.589884000
15	-1.771994000	-0.468255000	0.007765000
6	1.401217000	0.853878000	0.140174000
8	1.198085000	0.338617000	1.233558000
8	2.565804000	0.689693000	-0.523388000
6	3.545006000	-0.148628000	0.096805000
6	3.240793000	-1.611792000	-0.134761000
6	-0.773288000	-1.303313000	-1.242983000
6	-1.708251000	-1.618304000	1.406607000
6	-3.462064000	-0.698182000	-0.608628000
1	-2.770098000	2.210135000	0.556572000
1	-1.558657000	3.618784000	0.547703000
1	4.490520000	0.131949000	-0.364237000
1	3.590632000	0.079534000	1.161648000
1	2.304401000	-1.890716000	0.348876000
1	3.165398000	-1.821939000	-1.202623000
1	4.039450000	-2.227804000	0.280529000
1	0.661945000	1.914363000	-1.616231000
1	-3.594736000	-0.139608000	-1.536132000
1	-3.679103000	-1.753568000	-0.792698000
1	-4.174223000	-0.316017000	0.124465000
1	-1.118799000	-2.327305000	-1.399608000
1	-0.832669000	-0.757272000	-2.185892000

1	0.272570000	-1.330057000	-0.929864000
1	-2.324102000	-1.243392000	2.224924000
1	-0.679176000	-1.691666000	1.759128000
1	-2.067449000	-2.609140000	1.116195000

2_TS_PPhMe2_1

6	-0.213758000	2.588449000	-1.004836000
6	0.596514000	1.558144000	-0.918866000
6	1.749512000	0.923941000	-1.241390000
15	-0.661722000	0.001494000	0.521078000
6	2.665337000	0.564648000	-0.192082000
8	2.499303000	0.799110000	0.999990000
8	3.743637000	-0.110182000	-0.638142000
6	4.684089000	-0.524270000	0.355645000
6	5.812194000	-1.233208000	-0.350066000
6	-0.642359000	0.125376000	2.328141000
6	-2.399253000	-0.267242000	0.125406000
6	0.139923000	-1.579548000	0.190053000
6	-3.377251000	0.529286000	0.732279000
6	-4.714098000	0.398265000	0.380627000
6	-5.095339000	-0.524484000	-0.588004000
6	-4.130537000	-1.314637000	-1.202710000
6	-2.792290000	-1.186730000	-0.851538000
1	0.153317000	3.499991000	-1.466881000
1	-1.231843000	2.578167000	-0.639587000
1	5.038127000	0.352084000	0.900978000
1	4.182960000	-1.179704000	1.070834000
1	6.551224000	-1.568300000	0.377684000
1	5.442961000	-2.104440000	-0.891634000
1	6.304296000	-0.566331000	-1.058343000
1	-1.074095000	1.073180000	2.648608000
1	-1.189533000	-0.696608000	2.793642000
1	0.399350000	0.107168000	2.649250000
1	1.128751000	-1.561664000	0.651201000
1	-0.429225000	-2.413729000	0.603155000
1	0.271263000	-1.723790000	-0.883202000
1	-3.092581000	1.254501000	1.486270000
1	-5.459357000	1.016971000	0.864317000
1	-6.137528000	-0.626786000	-0.861558000
1	-4.419866000	-2.035831000	-1.956609000
1	-2.052232000	-1.809616000	-1.339350000
1	1.900201000	0.522962000	-2.235692000

2_TS_PPhMe2_2

6	1.898876000	-2.665705000	-1.006922000
6	1.256460000	-2.008677000	-0.065575000
6	0.417605000	-2.037181000	1.006751000
15	1.966344000	0.223397000	-0.264532000
6	-0.984952000	-1.719714000	0.901680000
8	-1.775313000	-1.683737000	1.835296000
8	-1.377231000	-1.467315000	-0.366073000
6	-2.727113000	-1.035080000	-0.534316000
6	-2.953173000	-0.803182000	-2.007232000
6	2.945390000	1.204658000	-1.437938000
6	0.509268000	1.226042000	0.056122000
6	2.928126000	0.338021000	1.258378000
6	-0.161377000	1.842115000	-1.006518000
6	-1.322313000	2.567378000	-0.782260000
6	-1.841130000	2.675807000	0.506052000
6	-1.191990000	2.055372000	1.564912000
6	-0.020133000	1.337634000	1.343528000
1	2.563967000	-2.190578000	-1.713982000
1	1.751124000	-3.739041000	-1.084344000
1	-3.403368000	-1.795935000	-0.140893000
1	-2.880066000	-0.120876000	0.044202000
1	-2.265340000	-0.047331000	-2.389013000
1	-2.804730000	-1.724501000	-2.571359000
1	-3.973171000	-0.457057000	-2.175832000
1	3.115397000	2.217481000	-1.067404000
1	3.906357000	0.707620000	-1.578999000
1	2.445905000	1.251444000	-2.404771000
1	2.468731000	-0.268544000	2.039539000
1	3.926250000	-0.056786000	1.063283000
1	3.009954000	1.369262000	1.606164000
1	0.224290000	1.751029000	-2.015513000
1	-1.825820000	3.046233000	-1.612620000
1	-2.749052000	3.238971000	0.680113000
1	-1.592575000	2.129367000	2.567889000
1	0.480651000	0.867064000	2.180742000
1	0.805422000	-2.185289000	2.007020000

2_TS_PPhMe2_3

6	-0.296611000	-3.188972000	-1.419275000
6	0.043884000	-1.943159000	-1.170595000
6	0.917985000	-0.918078000	-1.370054000
15	-1.526819000	-1.174213000	0.407941000
6	2.074992000	-0.737353000	-0.532416000
8	2.460483000	-1.477766000	0.364277000
8	2.747582000	0.393534000	-0.854173000
6	3.865335000	0.732761000	-0.031674000
6	3.412441000	1.409059000	1.243306000
6	-0.575858000	-1.374985000	1.928754000
6	-1.557182000	0.597521000	0.124272000
6	-3.217802000	-1.596239000	0.915518000
6	-0.463572000	1.392688000	0.478999000
6	-0.443739000	2.747339000	0.164673000
6	-1.516251000	3.323893000	-0.503263000
6	-2.609964000	2.540386000	-0.861317000
6	-2.627711000	1.186784000	-0.556550000
1	-1.158553000	-3.668935000	-0.977484000
1	0.318834000	-3.769863000	-2.100207000
1	4.442915000	-0.165896000	0.183673000
1	4.471917000	1.404682000	-0.636760000
1	2.807986000	0.728189000	1.843907000
1	4.277379000	1.712735000	1.834421000
1	2.823375000	2.299060000	1.014537000
1	-0.944176000	-0.719831000	2.720735000
1	-0.664565000	-2.414460000	2.248731000
1	0.479176000	-1.177536000	1.739837000
1	-3.551933000	-0.970806000	1.745798000
1	-3.908840000	-1.492265000	0.080047000
1	-3.225398000	-2.641181000	1.229062000
1	0.375335000	0.958833000	1.010817000
1	0.410329000	3.350551000	0.447050000
1	-1.502970000	4.379124000	-0.743909000
1	-3.449165000	2.985206000	-1.380688000
1	-3.479415000	0.585541000	-0.852198000
1	0.679851000	-0.125973000	-2.069586000

2_TS_PPh2Me_1

6	0.935248000	3.194713000	-0.706077000
6	-0.013592000	2.283104000	-0.696947000
6	-1.241014000	1.858612000	-1.099983000
15	0.891951000	0.503506000	0.669284000
6	-2.181837000	1.464632000	-0.089923000
8	-2.009960000	1.611873000	1.116986000
8	-3.280821000	0.857416000	-0.581382000
6	-4.230820000	0.394773000	0.379887000
6	-5.274249000	-0.404134000	-0.359080000
6	-0.150802000	-0.888873000	0.213842000
6	2.572618000	0.018625000	0.198805000
6	0.897163000	0.554384000	2.472414000
6	3.479891000	-0.537410000	1.103865000
6	4.760765000	-0.884349000	0.691805000
6	5.149889000	-0.685141000	-0.628023000
6	4.257352000	-0.124851000	-1.534952000
6	2.981936000	0.236081000	-1.120390000
6	-0.236669000	-1.231213000	-1.138723000
6	-1.105820000	-2.228190000	-1.561873000
6	-1.914974000	-2.881845000	-0.638856000
6	-1.846589000	-2.537914000	0.707359000
6	-0.973580000	-1.543672000	1.131961000
1	0.709194000	4.181334000	-1.098516000
1	1.936578000	3.012061000	-0.338910000
1	-1.416842000	1.573343000	-2.129261000
1	-3.716373000	-0.213630000	1.126119000
1	-4.668370000	1.253499000	0.893607000
1	-5.773363000	0.207949000	-1.110716000
1	-4.816825000	-1.261278000	-0.856322000
1	-6.025220000	-0.771507000	0.340480000
1	-0.088347000	0.878745000	2.804020000
1	1.137364000	-0.408103000	2.926385000
1	1.628625000	1.296400000	2.794801000
1	3.192030000	-0.706566000	2.133731000
1	5.454552000	-1.314059000	1.403137000
1	6.147884000	-0.957946000	-0.946215000
1	4.557725000	0.043917000	-2.561067000
1	2.298624000	0.702657000	-1.821654000
1	0.380072000	-0.712512000	-1.864694000
1	-1.158959000	-2.486565000	-2.611652000
1	-2.598401000	-3.654521000	-0.967330000
1	-2.477556000	-3.041082000	1.429232000
1	-0.944895000	-1.273035000	2.180062000

2_TS_PPh2Me_2

6	-0.630238000	-3.175893000	-0.740827000
6	0.212242000	-2.165742000	-0.776649000
6	1.366932000	-1.613140000	-1.234885000
15	-0.819763000	-0.493217000	0.639144000
6	2.310049000	-1.123912000	-0.269324000
8	2.204433000	-1.280509000	0.943795000
8	3.319884000	-0.415064000	-0.816907000
6	4.290951000	0.124356000	0.083281000
6	5.333274000	-0.909452000	0.444821000
6	0.054810000	1.004221000	0.164270000
6	-2.560563000	-0.173236000	0.251639000
6	-0.739898000	-0.573837000	2.439679000
6	-3.470250000	0.303947000	1.198334000
6	-4.796713000	0.525804000	0.849273000
6	-5.229989000	0.279164000	-0.448742000
6	-4.334391000	-0.202075000	-1.396918000

6	-3.011724000	-0.438144000	-1.044736000
6	0.054684000	1.362860000	-1.186591000
6	0.799075000	2.448619000	-1.629481000
6	1.568899000	3.176226000	-0.728856000
6	1.585440000	2.818198000	0.615424000
6	0.836857000	1.735588000	1.059983000
1	-0.315748000	-4.133397000	-1.144393000
1	-1.627235000	-3.103148000	-0.326703000
1	1.464083000	-1.314385000	-2.270745000
1	4.735808000	0.964783000	-0.448036000
1	3.783908000	0.499538000	0.972722000
1	4.873395000	-1.747980000	0.967454000
1	5.830325000	-1.282520000	-0.451296000
1	6.087060000	-0.467126000	1.097413000
1	0.287991000	-0.796998000	2.722428000
1	-1.060768000	0.348706000	2.925614000
1	-1.374304000	-1.395308000	2.775098000
1	-3.148537000	0.508850000	2.211538000
1	-5.491884000	0.895695000	1.592175000
1	-6.263676000	0.454293000	-0.717916000
1	-4.667344000	-0.406222000	-2.406413000
1	-2.321710000	-0.841662000	-1.777747000
1	-0.529475000	0.786189000	-1.895854000
1	0.787079000	2.718354000	-2.677670000
1	2.155895000	4.018151000	-1.072997000
1	2.185903000	3.380098000	1.319656000
1	0.874460000	1.455619000	2.105241000

2_TS_PPh2Me_3

6	0.198942000	-2.749702000	-1.662642000
6	-0.480641000	-2.261808000	-0.647739000
6	-1.630682000	-2.217891000	0.077201000
15	0.911290000	-0.653114000	0.341947000
6	-2.746114000	-1.407616000	-0.350439000
8	-2.896122000	-0.878687000	-1.441634000
8	-3.676681000	-1.297728000	0.629090000
6	-4.766473000	-0.409834000	0.374004000
6	-4.345152000	1.023386000	0.611123000
6	2.657320000	-0.214334000	0.161120000
6	0.010371000	0.896474000	0.117510000
6	0.726358000	-1.059136000	2.090560000
6	-0.229808000	1.786730000	1.166547000
6	-0.882803000	2.989805000	0.928209000
6	-1.296485000	3.315526000	-0.359380000
6	-1.073006000	2.429251000	-1.407037000
6	-0.431880000	1.220261000	-1.166622000
6	3.061866000	0.599747000	-0.902138000
6	4.406875000	0.865586000	-1.115008000
6	5.370586000	0.311592000	-0.277782000
6	4.979240000	-0.509120000	0.773192000
6	3.632361000	-0.773020000	0.992522000
1	-0.241123000	-3.556695000	-2.241083000
1	1.177949000	-2.390412000	-1.947466000
1	-1.671916000	-2.645911000	1.071056000
1	-5.119783000	-0.553298000	-0.646964000
1	-5.552059000	-0.712104000	1.064726000
1	-3.994007000	1.153914000	1.636105000
1	-3.540087000	1.296492000	-0.072549000
1	-5.185619000	1.698558000	0.445132000
1	1.124131000	-2.056961000	2.277253000
1	1.238358000	-0.344400000	2.734834000
1	-0.336413000	-1.061108000	2.333120000
1	0.097431000	1.548171000	2.171087000
1	-1.065601000	3.673946000	1.746980000
1	-1.802308000	4.254846000	-0.543354000
1	-1.408936000	2.671822000	-2.406894000
1	-0.282407000	0.513431000	-1.975128000
1	2.318575000	1.033963000	-1.561423000
1	4.703557000	1.506389000	-1.935517000
1	6.419706000	0.518754000	-0.444590000
1	5.722937000	-0.943432000	1.429232000
1	3.345438000	-1.413330000	1.817397000

2_TS_PPh3_1

6	0.112207000	-0.582409000	-3.177087000
6	-0.490295000	0.321633000	-2.431212000
6	-1.438307000	1.298335000	-2.361074000
15	0.631031000	0.029011000	-0.361994000
6	-2.646585000	1.138130000	-1.600970000
8	-3.512925000	1.988262000	-1.441202000
8	-2.760536000	-0.095071000	-1.046842000
6	-3.862296000	-0.274073000	-0.156737000
6	-3.810832000	-1.685115000	0.373258000
6	-0.399937000	0.749158000	0.915631000
6	0.874946000	-1.697315000	0.075238000
6	2.237171000	0.831429000	-0.181310000
6	-0.161438000	-2.579250000	-0.249908000
6	-0.058475000	-3.926681000	0.067908000
6	1.090027000	-4.407092000	0.689508000
6	2.132102000	-3.538533000	0.995629000
6	2.027055000	-2.186183000	0.692651000
6	3.096796000	0.830189000	-1.282149000
6	4.360163000	1.399506000	-1.186998000
6	4.768703000	1.986335000	0.005466000
6	3.914429000	1.999866000	1.103208000
6	2.653860000	1.423412000	1.013116000
6	-0.925281000	0.021841000	1.984808000
6	-1.760429000	0.641747000	2.906083000
6	-2.082551000	1.987822000	2.763765000
6	-1.563002000	2.717746000	1.700185000
6	-0.724429000	2.102855000	0.779086000
1	0.959819000	-1.168042000	-2.845817000
1	-0.267721000	-0.766149000	-4.177655000
1	-4.793687000	-0.083716000	-0.692341000
1	-3.785378000	0.460188000	0.649718000
1	-4.659995000	-1.861037000	1.034182000
1	-3.856134000	-2.407230000	-0.443407000
1	-2.894060000	-1.856585000	0.939554000
1	-1.046128000	-2.198603000	-0.750023000
1	-0.867647000	-4.602132000	-0.178581000
1	1.175321000	-5.459117000	0.929651000
1	3.027086000	-3.913166000	1.475511000
1	2.838764000	-1.512359000	0.939802000
1	2.769776000	0.387082000	-2.216316000
1	5.020064000	1.393524000	-2.044834000
1	5.750097000	2.437016000	0.078610000
1	4.230680000	2.457677000	2.031658000
1	1.993256000	1.431783000	1.871841000
1	-0.678296000	-1.026622000	2.101521000
1	-2.158060000	0.073094000	3.737091000
1	-2.736034000	2.467216000	3.481252000
1	-1.810996000	3.765023000	1.585826000
1	-0.311736000	2.675362000	-0.044461000
1	-1.238391000	2.285991000	-2.757087000

2_TS_PPh3_2

6	-0.157245000	0.862615000	-3.092596000
6	0.575934000	0.112050000	-2.296903000
6	1.746001000	-0.559699000	-2.102190000
15	-0.769472000	0.009158000	-0.345029000
6	2.700460000	-0.017808000	-1.175898000
8	2.598085000	1.063251000	-0.604170000
8	3.757805000	-0.836026000	-0.971969000
6	4.738181000	-0.395778000	-0.030468000
6	5.735163000	0.540907000	-0.674768000
6	-2.213738000	-1.074380000	-0.346356000
6	0.305742000	-0.648392000	0.927936000
6	-1.349840000	1.618856000	0.204192000
6	0.857189000	-1.909528000	0.683090000
6	1.771203000	-2.459741000	1.573541000
6	2.143321000	-1.752197000	2.710572000
6	1.591671000	-0.498739000	2.962183000
6	0.678790000	0.053948000	2.074414000
6	-0.440608000	2.680010000	0.120365000
6	-0.823400000	3.956988000	0.506752000
6	-2.120049000	4.189419000	0.955889000
6	-3.031502000	3.141824000	1.024165000
6	-2.650051000	1.858155000	0.651530000
6	-2.967903000	-1.171711000	-1.517451000
6	-4.110122000	-1.961996000	-1.553552000
6	-4.499762000	-2.670539000	-0.422790000
6	-3.748741000	-2.585283000	0.745270000
6	-2.611535000	-1.789166000	0.786084000
1	0.265711000	1.184962000	-4.039384000
1	-1.159242000	1.191955000	-2.850715000
1	5.224535000	-1.304903000	0.321582000
1	4.235728000	0.082538000	0.811248000
1	6.227462000	0.055127000	-1.518111000
1	5.236352000	1.442361000	-1.029581000
1	6.498678000	0.829814000	0.048733000
1	0.564598000	-2.461761000	-0.203519000
1	2.194761000	-3.436130000	1.376179000
1	2.858987000	-2.177074000	3.402870000
1	1.873908000	0.049341000	3.851989000
1	0.260961000	1.033691000	2.270961000
1	0.564353000	2.488084000	-0.242845000
1	-0.114002000	4.772418000	0.448162000
1	-2.420694000	5.187558000	1.247703000
1	-4.041004000	3.322028000	1.370859000
1	-3.363120000	1.044685000	0.711347000
1	-2.654604000	-0.631459000	-2.403710000
1	-4.688973000	-2.030865000	-2.465423000
1	-5.385442000	-3.292160000	-0.451807000
1	-4.050267000	-3.137088000	1.626341000
1	-2.032040000	-1.720761000	1.699038000
1	1.883368000	-1.566426000	-2.475434000

2_TS_PPh3_3

6	-0.309528000	-0.049012000	-3.219658000
6	0.408046000	0.603085000	-2.327388000

6	1.455839000	1.453043000	-2.108952000
15	-0.672996000	0.033121000	-0.316383000
6	2.715508000	0.993748000	-1.594431000
8	3.694239000	1.688539000	-1.352371000
8	2.723712000	-0.345245000	-1.378471000
6	3.906280000	-0.919461000	-0.822453000
6	3.942448000	-0.760602000	0.681164000
6	0.075738000	-1.372283000	0.527965000
6	-0.398943000	1.441458000	0.756633000
6	-2.447097000	-0.281305000	-0.351863000
6	0.925987000	1.715449000	1.115141000
6	1.235049000	2.850331000	1.852715000
6	0.225191000	3.726516000	2.234795000
6	-1.093523000	3.460950000	1.881418000
6	-1.406694000	2.326652000	1.142763000
6	-3.230919000	0.543572000	-1.166729000
6	-4.601734000	0.346808000	-1.253650000
6	-5.199923000	-0.691680000	-0.546531000
6	-4.425069000	-1.527107000	0.249569000
6	-3.053680000	-1.324181000	0.349955000
6	0.573265000	-2.417148000	-0.251854000
6	1.141554000	-3.532010000	0.351918000
6	1.223495000	-3.604048000	1.738501000
6	0.729490000	-2.565386000	2.521913000
6	0.154869000	-1.453195000	1.920082000
1	-0.008254000	-0.004922000	-4.262136000
1	-1.187710000	-0.631950000	-2.973733000
1	4.781412000	-0.465398000	-1.287672000
1	3.867527000	-1.972882000	-1.099616000
1	4.814541000	-1.273656000	1.089073000
1	4.004758000	0.293066000	0.955065000
1	3.046119000	-1.193556000	1.132144000
1	1.712329000	1.021851000	0.838359000
1	2.263332000	3.047829000	2.127441000
1	0.464512000	4.612094000	2.809305000
1	-1.883096000	4.136491000	2.184580000
1	-2.437324000	2.128945000	0.875275000
1	-2.758060000	1.339029000	-1.733478000
1	-5.200606000	0.994779000	-1.880389000
1	-6.267544000	-0.853135000	-0.621772000
1	-4.888220000	-2.338456000	0.796181000
1	-2.457171000	-1.977636000	0.974703000
1	0.532167000	-2.343241000	-1.332712000
1	1.528756000	-4.337560000	-0.258646000
1	1.674768000	-4.468031000	2.209344000
1	0.791504000	-2.621766000	3.601037000
1	-0.230226000	-0.645056000	2.530943000
1	1.313920000	2.524836000	-2.166955000

2_TS_Pani3_1

6	0.159401000	0.796024000	3.561112000
6	-0.523096000	-0.161778000	2.968912000
6	-1.472678000	-1.130729000	3.063637000
15	0.403095000	-0.045848000	0.744460000
6	-2.749896000	-1.022751000	2.412736000
8	-3.623234000	-1.880385000	2.404438000
8	-2.922605000	0.163162000	1.778658000
6	-4.102487000	0.271789000	0.982361000
6	-4.098142000	1.627982000	0.322444000
6	-0.745686000	-0.862302000	-0.360093000
6	0.572321000	1.641449000	0.157896000
6	1.991216000	-0.842871000	0.454366000
6	-0.418220000	2.559196000	0.539323000
6	-0.367909000	3.871862000	0.114886000
6	0.693163000	4.307953000	-0.686421000
6	1.696454000	3.412774000	-1.056782000
6	1.625661000	2.089036000	-0.634740000
6	2.980375000	-0.730845000	1.440565000
6	4.229497000	-1.291003000	1.261437000
6	4.518354000	-1.996446000	0.088802000
6	3.542056000	-2.127989000	-0.898540000
6	2.292005000	-1.548537000	-0.708412000
6	-1.373839000	-0.229060000	-1.428619000
6	-2.306324000	-0.896737000	-2.216650000
6	-2.623511000	-2.224478000	-1.931212000
6	-1.998282000	-2.872771000	-0.861438000
6	-1.074600000	-2.198362000	-0.086794000
1	0.970560000	1.335754000	3.090343000
1	-0.113739000	1.077065000	4.574136000
1	-4.980379000	0.140692000	1.617154000
1	-4.103043000	-0.533848000	0.242950000
1	-5.006168000	1.752446000	-0.268315000
1	-4.063457000	2.422438000	1.069466000
1	-3.239042000	1.736616000	-0.341450000
1	-1.234385000	2.227040000	1.172821000
1	-1.131881000	4.583733000	0.400382000
1	2.527542000	3.732311000	-1.669255000
1	2.407285000	1.400331000	-0.933909000
1	2.760008000	-0.200659000	2.360878000
1	4.996311000	-1.207014000	2.020729000
1	3.743262000	-2.672047000	-1.810503000
1	1.542849000	-1.652951000	-1.484567000
1	-1.137854000	0.803093000	-1.660149000
1	-2.773327000	-0.377467000	-3.041619000
1	-2.256757000	-3.903246000	-0.654345000
1	-0.593266000	-2.713010000	0.737880000
1	-1.235396000	-2.088186000	3.510337000
8	-3.515907000	-2.953876000	-2.633956000
6	-4.182331000	-2.330490000	-3.714669000
1	-4.764335000	-1.470010000	-3.375758000
1	-3.474992000	-2.008670000	-4.482713000
1	-4.853789000	-3.074314000	-4.134832000
8	5.757166000	-2.520730000	-0.003110000
6	6.088669000	-3.251189000	-1.169264000
1	5.437360000	-4.119975000	-1.287899000
1	6.021151000	-2.623134000	-2.060569000
1	7.114621000	-3.585815000	-1.042965000
8	0.673126000	5.606718000	-1.048693000
6	1.726629000	6.089469000	-1.861427000
1	2.690897000	5.987979000	-1.358336000
1	1.759490000	5.562334000	-2.817652000
1	1.522865000	7.141953000	-2.038333000

2_TS_Pani3_2

6	0.350542000	0.632085000	3.467474000
6	-0.482249000	-0.181065000	2.851542000
6	-1.566673000	-0.998137000	2.926726000
15	0.396811000	-0.093932000	0.606397000
6	-2.830490000	-0.659198000	2.330272000
8	-3.826057000	-1.371531000	2.312628000
8	-2.837122000	0.573044000	1.765933000
6	-4.016663000	0.913711000	1.036799000
6	-3.822408000	2.288663000	0.448485000
6	0.805544000	1.575149000	0.089521000
6	1.825820000	-1.116217000	0.215364000
6	-0.902554000	-0.665647000	-0.485208000
6	2.791148000	-1.320658000	1.198566000
6	3.940386000	-2.061413000	0.944685000
6	4.126239000	-2.622217000	-0.318162000
6	3.157981000	-2.436744000	-1.311352000
6	2.025441000	-1.692780000	-1.046329000
6	-1.421566000	-1.940806000	-0.259176000
6	-2.477730000	-2.437127000	-1.015707000
6	-3.032211000	-1.644313000	-2.019725000
6	-2.515984000	-0.367061000	-2.260629000
6	-1.468025000	0.115283000	-1.499637000
6	1.905240000	1.904823000	-0.697749000
6	2.163173000	3.223980000	-1.057277000
6	1.303687000	4.235168000	-0.628739000
6	0.198452000	3.917629000	0.168688000
6	-0.037040000	2.606771000	0.531355000
8	1.467656000	5.539730000	-0.928453000
8	-4.061860000	-2.030772000	-2.802484000
8	5.201434000	-3.358289000	-0.663532000
6	2.577784000	5.908429000	-1.725211000
6	-4.634198000	-3.303130000	-2.566727000
6	6.211786000	-3.559985000	0.307199000
1	1.222559000	1.068384000	2.998288000
1	0.146463000	0.898578000	4.500501000
1	-1.468380000	-2.004214000	3.314949000
1	-4.178425000	0.163836000	0.257817000
1	-4.876305000	0.886267000	1.708508000
1	-4.722926000	2.589531000	-0.087604000
1	-3.628592000	3.021988000	1.232792000
1	-2.986531000	2.298983000	-0.252672000
1	2.644876000	-0.898054000	2.186851000
1	4.669195000	-2.202542000	1.729881000
1	3.319039000	-2.885116000	-2.283422000
1	1.284808000	-1.553627000	-1.825421000
1	-0.992960000	-2.565616000	0.517230000
1	-2.859378000	-3.427621000	-0.812824000
1	-2.954956000	0.230269000	-3.049729000
1	-1.083091000	1.108902000	-1.696950000
1	2.576634000	1.127133000	-1.042790000
1	3.025787000	3.449634000	-1.667978000
1	-0.451271000	4.717500000	0.500506000
1	-0.885708000	2.364776000	1.163094000
1	2.525914000	6.986831000	-1.846955000
1	3.518299000	5.645095000	-1.235974000
1	2.533956000	5.431812000	-2.707108000
1	-3.904395000	-4.100361000	-2.726185000
1	-5.031255000	-3.373698000	-1.551415000
1	-5.447054000	-3.413611000	-3.279366000
1	6.982065000	-4.160223000	-0.168982000
1	6.642281000	-2.609295000	0.630008000
1	5.822195000	-4.095167000	1.175974000

2_TS_Pani3_3

6	0.442037000	0.512433000	3.516129000

6	-0.292249000	-0.398067000	2.911326000
6	-1.230052000	-1.377917000	3.007181000
15	0.436107000	-0.099564000	0.629854000
6	-2.557575000	-1.227938000	2.475551000
8	-3.425723000	-2.090673000	2.467028000
8	-2.786457000	0.005444000	1.961716000
6	-4.028408000	0.168649000	1.276847000
6	-4.084313000	1.576883000	0.739992000
6	-0.803772000	-0.828065000	-0.438980000
6	0.569913000	1.626220000	0.156810000
6	1.980136000	-0.891208000	0.150633000
6	-0.387984000	2.508666000	0.679380000
6	-0.366590000	3.850214000	0.354781000
6	0.633944000	4.350356000	-0.485975000
6	1.606808000	3.490474000	-0.994747000
6	1.564596000	2.137727000	-0.671930000
6	2.986735000	-1.030391000	1.103209000
6	4.213792000	-1.602246000	0.784373000
6	4.438321000	-2.056612000	-0.514313000
6	3.431927000	-1.933279000	-1.479145000
6	2.221200000	-1.358206000	-1.148826000
6	-1.523926000	-0.114343000	-1.392561000
6	-2.515473000	-0.725067000	-2.154339000
6	-2.799437000	-2.076343000	-1.958709000
6	-2.082849000	-2.804895000	-1.004253000
6	-1.100788000	-2.186583000	-0.254979000
1	1.211442000	1.090124000	3.020767000
1	0.259160000	0.710976000	4.568244000
1	-4.850358000	-0.027387000	1.967314000
1	-4.086026000	-0.569575000	0.472134000
1	-5.037662000	1.741526000	0.237012000
1	-3.992876000	2.303709000	1.548546000
1	-3.282090000	1.752550000	0.021613000
1	-1.155119000	2.125171000	1.344575000
1	-1.105788000	4.535585000	0.749475000
1	2.391929000	3.859608000	-1.639208000
1	2.322499000	1.478180000	-1.078397000
1	2.810716000	-0.691991000	2.118629000
1	4.973328000	-1.698076000	1.547088000
1	3.625386000	-2.297128000	-2.480166000
1	1.449537000	-1.267304000	-1.904817000
1	-1.313325000	0.936350000	-1.554995000
1	-3.052416000	-0.144583000	-2.891264000
1	-2.316577000	-3.852596000	-0.865546000
1	-0.547920000	-2.763297000	0.478877000
1	-0.955072000	-2.366609000	3.352835000
8	-3.742751000	-2.754897000	-2.646027000
6	-4.497823000	-2.050046000	-3.612224000
1	-5.052280000	-1.226754000	-3.155408000
1	-3.857163000	-1.658371000	-4.406020000
1	-5.199439000	-2.762788000	-4.036938000
8	5.587851000	-2.630289000	-0.922285000
6	6.630884000	-2.780054000	0.022952000
1	6.956546000	-1.810648000	0.406908000
1	6.319217000	-3.413890000	0.856162000
1	7.456097000	-3.254964000	-0.500321000
8	0.588979000	5.672794000	-0.746313000
6	1.585291000	6.221106000	-1.588792000
1	2.581152000	6.087867000	-1.159987000
1	1.554886000	5.767208000	-2.581969000
1	1.369358000	7.282711000	-1.672254000

2_TS_Ppfp3_1

6	0.191696000	0.750881000	3.295816000
6	-0.449307000	-0.198280000	2.643909000
6	-1.391128000	-1.182328000	2.689811000
15	0.527229000	-0.018542000	0.495292000
6	-2.650532000	-1.069829000	2.008091000
8	-3.519911000	-1.930011000	1.959836000
8	-2.812370000	0.128087000	1.390993000
6	-3.990078000	0.258122000	0.593671000
6	-3.980863000	1.628936000	-0.034942000
6	-0.571443000	-0.855929000	-0.644519000
6	0.674521000	1.680163000	-0.068774000
6	2.141865000	-0.780367000	0.244669000
6	-0.355795000	2.554909000	0.294365000
6	-0.330473000	3.882710000	-0.105186000
6	0.751585000	4.319515000	-0.848773000
6	1.797800000	3.489922000	-1.209254000
6	1.751943000	2.159543000	-0.815627000
6	3.073662000	-0.698767000	1.282306000
6	4.345900000	-1.233215000	1.138488000
6	4.659387000	-1.859715000	-0.053576000
6	3.761794000	-1.970533000	-1.100354000
6	2.496815000	-1.422447000	-0.944338000
6	-1.209763000	-0.218587000	-1.709795000
6	-2.105327000	-0.912001000	-2.511865000
6	-2.352034000	-2.242806000	-2.224857000
6	-1.739600000	-2.908829000	-1.179413000
6	-0.844423000	-2.203729000	-0.387012000
1	1.008830000	1.322098000	2.874580000
1	-0.122843000	0.990457000	4.307144000
1	-4.869236000	0.115011000	1.223891000
1	-3.992552000	-0.530035000	-0.163774000
1	-4.887894000	1.769470000	-0.623349000
1	-3.944693000	2.405958000	0.730167000
1	-3.121154000	1.750548000	-0.695914000
1	-1.182018000	2.186373000	0.892802000
1	-1.119691000	4.573368000	0.159345000
1	2.622611000	3.883828000	-1.787459000
1	2.560238000	1.494970000	-1.094961000
1	2.798896000	-0.217983000	2.214110000
1	5.079972000	-1.180193000	1.930886000
1	4.055620000	-2.473456000	-2.011695000
1	1.782867000	-1.495585000	-1.755521000
1	-1.007760000	0.824329000	-1.920976000
1	-2.605413000	-0.438234000	-3.345811000
1	-1.964630000	-3.950481000	-0.995695000
1	-0.347356000	-2.708013000	0.433965000
1	-1.158235000	-2.144913000	3.127317000
9	5.884498000	-2.388581000	-0.199515000
9	0.791979000	5.606238000	-1.230159000
9	-3.220138000	-2.916326000	-2.998338000

2_TS_Ppfp3_2

6	0.223798000	0.762127000	3.283411000
6	-0.566854000	0.045350000	2.512067000
6	-1.749850000	-0.615135000	2.364683000
15	0.634273000	0.012596000	0.467034000
6	-2.751281000	-0.034637000	1.514260000
8	-2.671235000	1.067567000	0.979712000
8	-3.821213000	-0.840344000	1.334269000
6	-4.845299000	-0.365711000	0.457431000
6	-5.812058000	0.540635000	1.185302000
6	-0.532874000	-0.619770000	-0.735418000
6	1.165188000	1.635780000	-0.089267000
6	2.068860000	-1.072902000	0.335504000
6	0.262114000	2.690289000	0.095216000
6	0.598484000	3.980924000	-0.283500000
6	1.854240000	4.198693000	-0.822493000
6	2.778627000	3.186495000	-1.001386000
6	2.424718000	1.895703000	-0.631736000
6	2.846791000	-1.294428000	1.473409000
6	3.976143000	-2.099938000	1.416003000
6	4.300752000	-2.682759000	0.205677000
6	3.550810000	-2.495188000	-0.942411000
6	2.429647000	-1.682492000	-0.869751000
6	-1.027569000	0.125333000	-1.806722000
6	-2.018987000	-0.392215000	-2.626635000
6	-2.508402000	-1.657393000	-2.350841000
6	-2.039837000	-2.429182000	-1.305179000
6	-1.047371000	-1.897161000	-0.492633000
1	1.210051000	1.094192000	2.986432000
1	-0.131671000	1.051574000	4.267801000
1	-5.345608000	-1.261572000	0.091129000
1	-4.383213000	0.148795000	-0.386058000
1	-6.608714000	0.856539000	0.510530000
1	-6.263865000	0.019333000	2.030028000
1	-5.298158000	1.428336000	1.553284000
1	-0.710262000	2.485608000	0.532045000
1	-0.086397000	4.808469000	-0.157713000
1	3.748780000	3.411030000	-1.423546000
1	3.137060000	1.091774000	-0.769051000
1	2.563078000	-0.840451000	2.415688000
1	4.589367000	-2.285636000	2.287176000
1	3.846495000	-2.977339000	-1.864319000
1	1.830708000	-1.520171000	-1.757850000
1	-0.645914000	1.119557000	-2.001597000
1	-2.414319000	0.167741000	-3.463295000
1	-2.450908000	-3.414521000	-1.132522000
1	-0.665675000	-2.484628000	0.334942000
1	-1.872297000	-1.633210000	2.711145000
9	5.385915000	-3.470307000	0.140792000
9	2.192246000	5.448485000	-1.180864000
9	-3.478362000	-2.156695000	-3.136767000

2_TS_Ppfp3_3

6	0.492335000	0.696038000	3.337406000
6	-0.424555000	0.124207000	2.584792000
6	-1.696233000	-0.356257000	2.476129000
15	0.658247000	-0.009195000	0.484634000
6	-2.636461000	0.393346000	1.690778000
8	-2.430786000	1.504308000	1.210393000
8	-3.806746000	-0.252679000	1.500535000
6	-4.750342000	0.388925000	0.641197000
6	-5.833568000	-0.610585000	0.322213000
6	-0.647241000	-0.446793000	-0.659339000
6	1.379297000	1.539546000	-0.069667000
6	1.923636000	-1.274559000	0.256522000

6	0.655879000	2.706778000	0.204762000
6	1.151998000	3.947023000	-0.167223000
6	2.385836000	3.998149000	-0.791429000
6	3.136262000	2.869016000	-1.062279000
6	2.622436000	1.631776000	-0.698040000
6	2.756028000	-1.586417000	1.333482000
6	3.765676000	-2.529785000	1.201449000
6	3.915760000	-3.158303000	-0.020375000
6	3.106297000	-2.884117000	-1.108749000
6	2.106983000	-1.933590000	-0.962442000
6	-1.098839000	0.387160000	-1.683253000
6	-2.201320000	0.031496000	-2.445679000
6	-2.843932000	-1.160785000	-2.158842000
6	-2.420229000	-2.018899000	-1.162509000
6	-1.314824000	-1.649291000	-0.407861000
1	1.500821000	0.900052000	3.001477000
1	0.225835000	0.994167000	4.346979000
1	-4.238838000	0.724195000	-0.262930000
1	-5.152220000	1.272262000	1.142260000
1	-6.579017000	-0.154514000	-0.329513000
1	-5.414734000	-1.478971000	-0.189734000
1	-6.331734000	-0.950236000	1.230718000
1	-0.303463000	2.630855000	0.706815000
1	0.607436000	4.860697000	0.028765000
1	4.097171000	2.964682000	-1.549373000
1	3.196235000	0.737407000	-0.906929000
1	2.610258000	-1.093037000	2.287174000
1	4.418458000	-2.785556000	2.024761000
1	3.264080000	-3.405595000	-2.043104000
1	1.465651000	-1.702198000	-1.804143000
1	-0.596135000	1.325033000	-1.883594000
1	-2.568581000	0.663328000	-3.243043000
1	-2.952706000	-2.942537000	-0.979790000
1	-0.965871000	-2.304533000	0.382483000
1	-1.945719000	-1.363571000	2.783662000
9	4.883809000	-4.078281000	-0.156179000
9	2.879245000	5.196944000	-1.143236000
9	-3.929237000	-1.495497000	-2.879044000

2_TS_PCy3_1

6	0.080107000	-0.028574000	-3.242567000
6	0.716489000	0.397418000	-2.173503000
6	1.828460000	0.924717000	-1.604286000
15	-0.742736000	-0.026101000	-0.277781000
6	3.052362000	0.162583000	-1.493187000
8	3.319742000	-0.896458000	-2.041144000
8	3.948293000	0.783288000	-0.690440000
6	5.225263000	0.154162000	-0.569039000
6	6.059519000	0.990621000	0.368057000
6	0.498904000	-0.825456000	0.803898000
6	-0.893667000	1.671939000	0.439483000
6	-2.377628000	-0.824613000	-0.016342000
6	-1.106737000	2.742213000	-0.638442000
6	-1.026711000	4.140957000	-0.036707000
6	-2.046312000	4.315979000	1.080840000
6	-1.854459000	3.246748000	2.148128000
6	-1.945069000	1.850236000	1.540019000
6	-3.382392000	-0.184588000	-0.982111000
6	-4.762527000	-0.817450000	-0.853401000
6	-4.687678000	-2.315828000	-1.112143000
6	-3.703225000	-2.966027000	-0.150298000
6	-2.318718000	-2.333593000	-0.255673000
6	0.098309000	-1.085218000	2.254767000
6	1.302810000	-1.574473000	3.057289000
6	1.945492000	-2.799885000	2.418950000
6	2.330474000	-2.515044000	0.971759000
6	1.104758000	-2.083855000	0.175986000
1	0.509606000	0.184553000	-4.217369000
1	-0.850737000	-0.575904000	-3.199531000
1	1.789952000	1.880837000	-1.093971000
1	5.091580000	-0.860807000	-0.189701000
1	5.682548000	0.079802000	-1.557450000
1	5.590332000	1.054089000	1.350384000
1	7.045687000	0.541684000	0.486642000
1	6.185770000	2.000609000	-0.022647000
1	1.301766000	-0.072188000	0.817780000
1	0.093910000	1.851255000	0.889623000
1	-2.706012000	-0.648718000	1.016872000
1	-0.373153000	2.634735000	-1.439108000
1	-2.093037000	2.599460000	-1.095183000
1	-0.019481000	4.291634000	0.369792000
1	-1.174221000	4.892762000	-0.814913000
1	-1.969601000	5.313687000	1.518073000
1	-3.055238000	4.224172000	0.661895000
1	-0.868511000	3.371696000	2.610426000
1	-2.597860000	3.356851000	2.940727000
1	-2.943082000	1.720262000	1.106627000
1	-1.838956000	1.092949000	2.317619000
1	-3.442835000	0.893161000	-0.818024000
1	-3.013769000	-0.328633000	-2.006391000
1	-5.143498000	-0.644704000	0.159878000
1	-5.458663000	-0.338763000	-1.545205000
1	-5.674406000	-2.773776000	-1.017825000
1	-4.350239000	-2.484077000	-2.141443000
1	-4.071506000	-2.839937000	0.874441000
1	-3.633660000	-4.040002000	-0.335193000
1	-1.900689000	-2.520953000	-1.254238000
1	-1.649880000	-2.803599000	0.465708000
1	-0.299607000	-0.178107000	2.712345000
1	-0.696577000	-1.840234000	2.283006000
1	2.043582000	-0.767361000	3.097507000
1	1.005760000	-1.788224000	4.086467000
1	2.817292000	-3.114072000	2.996898000
1	1.231197000	-3.631524000	2.437294000
1	3.072103000	-1.705771000	0.942994000
1	2.793338000	-3.391684000	0.513437000
1	0.375398000	-2.898490000	0.189841000
1	1.365200000	-1.897607000	-0.867558000

2_TS_PCy3_2

6	-0.684440000	0.619139000	-3.189867000
6	0.322423000	0.180994000	-2.465357000
6	1.603977000	-0.264492000	-2.405306000
15	-0.694655000	0.061184000	-0.213338000
6	2.590751000	0.468528000	-1.662883000
8	2.467769000	1.605403000	-1.222503000
8	3.716632000	-0.251499000	-1.448095000
6	4.706677000	0.364682000	-0.625327000
6	5.850305000	-0.607432000	-0.480157000
6	-1.221719000	1.639018000	0.567914000
6	-2.051572000	-1.171947000	0.018650000
6	0.620788000	-0.580035000	0.892912000
6	-3.474095000	-0.617403000	0.078685000
6	-4.461808000	-1.744255000	0.368527000
6	-4.379574000	-2.831809000	-0.695888000
6	-2.956957000	-3.364660000	-0.815928000
6	-1.980587000	-2.227446000	-1.091777000
6	0.292303000	-0.541760000	2.388856000
6	1.527301000	-0.917804000	3.203321000
6	2.029092000	-2.304939000	2.821387000
6	2.297701000	-2.390987000	1.324225000
6	1.056879000	-2.002890000	0.526056000
6	-0.018083000	2.524783000	0.907099000
6	-0.478058000	3.813035000	1.583105000
6	-1.444403000	4.587369000	0.696177000
6	-2.624125000	3.710808000	0.297121000
6	-2.136397000	2.431768000	-0.374416000
1	-0.500896000	0.871572000	-4.230544000
1	-1.687287000	0.743638000	-2.804987000
1	1.830588000	-1.285722000	-2.686773000
1	5.029167000	1.301167000	-1.083703000
1	4.262074000	0.607616000	0.344199000
1	5.511382000	-1.536438000	-0.019420000
1	6.284275000	-0.841403000	-1.452591000
1	6.628088000	-0.174600000	0.149026000
1	-1.769136000	1.411675000	1.493610000
1	-1.853988000	-1.671172000	0.977397000
1	1.478451000	0.083013000	0.712925000
1	-3.561834000	0.166061000	0.834825000
1	-3.724766000	-0.167715000	-0.889660000
1	-4.226141000	-2.178450000	1.346924000
1	-5.476673000	-1.345514000	0.430135000
1	-5.076011000	-3.641854000	-0.469640000
1	-4.681468000	-2.407880000	-1.660577000
1	-2.673893000	-3.857530000	0.121705000
1	-2.894153000	-4.116302000	-1.605586000
1	-2.241862000	-1.742873000	-2.040422000
1	-0.963149000	-2.608407000	-1.206437000
1	-0.076419000	0.438209000	2.695825000
1	-0.505799000	-1.263981000	2.596950000
1	2.315725000	-0.181098000	3.005947000
1	1.303960000	-0.871644000	4.271393000
1	2.927962000	-2.557290000	3.387860000
1	1.263881000	-3.043791000	3.086706000
1	3.112586000	-1.705276000	1.059403000
1	2.622829000	-3.396975000	1.050201000
1	0.243358000	-2.696985000	0.771500000
1	1.248849000	-2.089941000	-0.544329000
1	0.697055000	2.006859000	1.548059000
1	0.511225000	2.757433000	-0.023049000
1	-0.978009000	3.560486000	2.525900000
1	0.387446000	4.429710000	1.834855000
1	-1.789072000	5.491917000	1.201926000
1	-0.917253000	4.904851000	-0.210572000
1	-3.201086000	3.447093000	1.191422000
1	-3.296655000	4.252925000	-0.371086000
1	-1.554189000	2.693444000	-1.266039000
1	-2.982820000	1.832600000	-0.711187000

2_TS_PCy3_3

6	0.166594000	0.967692000	-3.142424000
6	-0.631290000	1.031970000	-2.099879000
6	-1.828403000	1.356300000	-1.555149000
15	0.807392000	0.103682000	-0.302242000
6	-2.916752000	0.409263000	-1.571699000
8	-2.949109000	-0.658071000	-2.168645000
8	-3.966145000	0.832289000	-0.829536000
6	-5.100630000	-0.034701000	-0.791220000
6	-6.075343000	0.535194000	0.208185000
6	-0.439401000	-0.220382000	0.990085000
6	1.950582000	-1.316508000	-0.495370000
6	1.828661000	1.458586000	0.390102000
6	2.552760000	-1.886488000	0.788010000
6	3.629679000	-2.915706000	0.455069000
6	3.062544000	-4.028421000	-0.419037000
6	2.448714000	-3.458854000	-1.692992000
6	1.365706000	-2.437842000	-1.359726000
6	3.009787000	1.808453000	-0.517633000
6	3.802570000	2.983706000	0.046396000
6	2.914069000	4.203622000	0.250369000
6	1.738682000	3.859346000	1.155609000
6	0.942766000	2.695905000	0.576924000
6	-1.191346000	-1.528730000	0.737181000
6	-2.437114000	-1.591139000	1.613845000
6	-2.056539000	-1.484246000	3.086097000
6	-1.263127000	-0.209731000	3.349707000
6	-0.024096000	-0.132699000	2.459744000
1	1.150600000	0.520736000	-3.115423000
1	-0.180936000	1.381695000	-4.085138000
1	-1.941415000	2.253364000	-0.957667000
1	-5.537387000	-0.098132000	-1.789953000
1	-4.777037000	-1.038008000	-0.507664000
1	-5.624405000	0.581298000	1.200679000
1	-6.383860000	1.540516000	-0.079655000
1	-6.962804000	-0.095344000	0.262612000
1	-1.166093000	0.589937000	0.810461000
1	2.770703000	-0.878453000	-1.082813000
1	2.223989000	1.155134000	1.368833000
1	2.961248000	-1.086400000	1.412142000
1	1.757383000	-2.373702000	1.363097000
1	4.443903000	-2.415915000	-0.082530000
1	4.055145000	-3.327388000	1.372911000
1	3.839391000	-4.756859000	-0.660682000
1	2.285768000	-4.560905000	0.142315000
1	3.232984000	-2.969053000	-2.281860000
1	2.034924000	-4.259243000	-2.309896000
1	0.561349000	-2.942110000	-0.817407000
1	0.924585000	-2.021233000	-2.269770000
1	3.670899000	0.948197000	-0.641308000
1	2.624409000	2.069651000	-1.512155000
1	4.231909000	2.689187000	1.010967000
1	4.637246000	3.221368000	-0.616447000
1	3.491430000	5.031938000	0.666141000
1	2.529578000	4.533577000	-0.721663000
1	2.114851000	3.578508000	2.146301000
1	1.089440000	4.726697000	1.291554000
1	0.545976000	2.985563000	-0.404721000
1	0.083716000	2.468190000	1.213439000
1	-1.451710000	-1.624819000	-0.320485000
1	-0.535755000	-2.369722000	0.991664000
1	-3.097075000	-0.755133000	1.348752000
1	-2.990332000	-2.513744000	1.424020000
1	-2.947430000	-1.515480000	3.716960000
1	-1.440632000	-2.350142000	3.356224000
1	-1.899732000	0.657825000	3.138733000
1	-0.975063000	-0.148655000	4.401514000
1	0.648247000	-0.962144000	2.700915000
1	0.518703000	0.792691000	2.662911000

2_TS_PBu3_1

6	0.087306000	1.348958000	-2.843577000
6	0.796576000	0.652960000	-1.983738000
6	1.899529000	-0.070447000	-1.674079000
15	-0.696541000	0.518433000	0.008636000
6	2.734924000	0.326507000	-0.573468000
8	2.571043000	1.325008000	0.117986000
8	3.729884000	-0.555724000	-0.332413000
6	4.538710000	-0.314245000	0.822121000
6	3.847005000	-0.790467000	2.080002000
6	-0.731798000	1.671523000	1.424618000
6	-2.454257000	0.126040000	-0.282837000
6	-0.033491000	-1.008491000	0.727008000
6	-3.243040000	-0.350741000	0.934650000
6	-1.579001000	2.917841000	1.166511000
6	-0.313803000	-2.227003000	-0.149439000
6	-1.239240000	3.645464000	-0.131722000
6	0.222107000	4.058954000	-0.219324000
6	0.631509000	-3.380525000	0.156663000
6	0.325978000	-4.615752000	-0.675224000
6	-4.646612000	-0.835582000	0.576510000
6	-4.637328000	-2.136738000	-0.214458000
1	0.561043000	1.670929000	-3.766309000
1	-0.947711000	1.617797000	-2.684350000
1	2.044039000	-1.053870000	-2.105660000
1	5.457694000	-0.870544000	0.645813000
1	4.775317000	0.747986000	0.880694000
1	3.582258000	-1.845949000	1.994072000
1	4.510505000	-0.671860000	2.937609000
1	2.940884000	-0.212633000	2.263666000
1	-1.105334000	1.137179000	2.303873000
1	0.308975000	1.938745000	1.625179000
1	-2.922321000	1.020634000	-0.708974000
1	-2.463048000	-0.626462000	-1.077051000
1	1.048646000	-0.867808000	0.822263000
1	-0.419262000	-1.156023000	1.739893000
1	-2.703678000	-1.166961000	1.428317000
1	-3.308200000	0.464380000	1.660016000
1	-2.635810000	2.634811000	1.152739000
1	-1.451467000	3.606072000	2.007477000
1	-1.352203000	-2.550318000	-0.011739000
1	-0.215800000	-1.956164000	-1.207896000
1	-1.485678000	2.999743000	-0.983099000
1	-1.882483000	4.524407000	-0.220216000
1	0.497612000	4.683143000	0.635407000
1	0.882634000	3.189428000	-0.226485000
1	0.417354000	4.630853000	-1.128111000
1	1.657917000	-3.044520000	-0.030615000
1	0.573490000	-3.619417000	1.223276000
1	1.016180000	-5.431744000	-0.457683000
1	-0.687757000	-4.973586000	-0.481242000
1	0.398832000	-4.390862000	-1.741781000
1	-5.218285000	-0.979712000	1.496560000
1	-5.162224000	-0.055705000	0.007386000
1	-5.650450000	-2.484126000	-0.421261000
1	-4.128001000	-2.026205000	-1.173529000
1	-4.123415000	-2.921955000	0.346329000

2_TS_PBu3_2

6	-1.892207000	-0.220136000	2.399254000
6	-0.734522000	-0.526318000	1.853129000
6	0.533170000	-0.977610000	2.005101000
15	-1.012679000	0.200544000	-0.536911000
6	1.138704000	-1.855178000	1.044980000
8	0.585900000	-2.310416000	0.049291000
8	2.439674000	-2.098390000	1.315813000
6	3.170524000	-2.859975000	0.351534000
6	3.621943000	-1.987020000	-0.797959000
6	-2.011149000	1.719138000	-0.782999000
6	0.550807000	0.599509000	-1.395549000
6	-1.759839000	-0.898881000	-1.795947000
6	1.679944000	1.178658000	-0.548006000
6	-1.324915000	3.015147000	-0.358785000
6	-3.270434000	-1.090422000	-1.690995000
6	-1.017260000	3.108828000	1.129422000
6	-0.249249000	4.378343000	1.460975000
6	-3.739438000	-1.555648000	-0.317119000
6	-3.055113000	-2.832997000	0.145273000
6	2.844752000	1.650527000	-1.407339000
6	3.996349000	2.176102000	-0.564965000
1	-2.168808000	-0.692205000	3.336605000
1	-2.587349000	0.484780000	1.962483000
1	1.174576000	-0.503009000	2.738074000
1	4.022477000	-3.264573000	0.895289000
1	2.551975000	-3.685568000	-0.000295000
1	4.213488000	-1.146300000	-0.429760000
1	4.237395000	-2.565684000	-1.487969000
1	2.760867000	-1.599662000	-1.343615000
1	-2.265011000	1.765323000	-1.848447000
1	-2.952541000	1.592446000	-0.236034000
1	0.882819000	-0.338129000	-1.852483000
1	0.304938000	1.284086000	-2.218385000
1	-1.510301000	-0.466203000	-2.771799000
1	-1.234924000	-1.853093000	-1.718643000
1	1.322237000	2.016858000	0.059132000
1	2.040804000	0.424631000	0.157832000
1	-0.394967000	3.137429000	-0.926043000
1	-1.960743000	3.860049000	-0.640684000
1	-3.774890000	-0.154836000	-1.947634000
1	-3.575248000	-1.823872000	-2.443973000
1	-1.953627000	3.069358000	1.695305000
1	-0.435961000	2.236509000	1.443999000
1	-0.807728000	5.265205000	1.152216000
1	-0.045297000	4.463811000	2.528807000
1	0.709420000	4.392444000	0.934329000
1	-3.550629000	-0.760292000	0.411792000
1	-4.822542000	-1.699028000	-0.340694000
1	-1.979647000	-2.683783000	0.266088000

1	-3.451823000	-3.170008000	1.104552000
1	-3.203376000	-3.636317000	-0.581868000
1	3.185870000	0.819488000	-2.033895000
1	2.491328000	2.429105000	-2.091222000
1	4.369681000	1.398441000	0.106372000
1	4.829855000	2.516510000	-1.180564000
1	3.670875000	3.015976000	0.053926000

2_TS_PBu3_3

6	0.965764000	1.680642000	2.377976000
6	-0.079039000	1.035427000	1.906524000
6	-1.303047000	0.491830000	2.077312000
15	0.668542000	0.446885000	-0.451983000
6	-2.329139000	0.615616000	1.077896000
8	-2.216619000	1.213528000	0.014060000
8	-3.452653000	-0.056845000	1.406300000
6	-4.499078000	-0.078634000	0.431115000
6	-4.253386000	-1.157522000	-0.599119000
6	2.452037000	0.059991000	-0.529366000
6	-0.100572000	-1.098816000	-1.045257000
6	0.467381000	1.508592000	-1.926855000
6	-0.465024000	-2.112099000	0.046498000
6	2.858088000	-1.120543000	0.345214000
6	1.399348000	2.718321000	-1.970687000
6	4.367716000	-1.317027000	0.391600000
6	4.764694000	-2.515586000	1.239349000
6	1.372261000	3.566307000	-0.702507000
6	-0.024023000	4.043446000	-0.333168000
6	-0.439090000	-3.545650000	-0.474692000
6	-1.433932000	-3.779247000	-1.602848000
1	0.838597000	2.316095000	3.248768000
1	1.952566000	1.615837000	1.938641000
1	-1.451902000	-0.231627000	2.869979000
1	-5.410066000	-0.274322000	0.994169000
1	-4.575892000	0.903176000	-0.035195000
1	-4.182321000	-2.133755000	-0.117412000
1	-5.075750000	-1.185843000	-1.315145000
1	-3.327385000	-0.963913000	-1.143273000
1	2.726060000	-0.130493000	-1.574754000
1	2.997525000	0.957184000	-0.214167000
1	-0.986397000	-0.806478000	-1.616056000
1	0.601194000	-1.551980000	-1.759547000
1	0.634695000	0.878528000	-2.807813000
1	-0.579999000	1.817985000	-1.940690000
1	0.221754000	-2.020151000	0.892832000
1	-1.461866000	-1.886781000	0.438702000
1	2.478142000	-0.975832000	1.364477000
1	2.389073000	-2.036918000	-0.031198000
1	2.422481000	2.380285000	-2.156751000
1	1.117647000	3.340495000	-2.825760000
1	4.743327000	-1.439232000	-0.628933000
1	4.832549000	-0.408359000	0.786010000
1	4.326612000	-3.433765000	0.841495000
1	5.846785000	-2.648399000	1.271931000
1	4.410279000	-2.398514000	2.265916000
1	1.784704000	2.983335000	0.128741000
1	2.038015000	4.422770000	-0.834876000
1	-0.471512000	4.606880000	-1.156828000
1	-0.681994000	3.201763000	-0.105875000
1	-0.000889000	4.692665000	0.543929000
1	0.574767000	-3.771192000	-0.824559000
1	-0.648321000	-4.234087000	0.347565000
1	-1.410569000	-4.813524000	-1.948646000
1	-2.451758000	-3.559655000	-1.271272000
1	-1.219842000	-3.139205000	-2.461445000

3_TS_PMe3_1

15	2.196639000	0.158225000	0.162847000
6	1.181438000	1.630976000	-0.079934000
6	3.886898000	0.810893000	0.038617000
6	1.990863000	-0.729032000	-1.396295000
1	1.435580000	2.123756000	-1.020832000
1	0.126649000	1.352872000	-0.100717000
1	4.601104000	-0.012767000	0.057144000
1	4.016193000	1.373977000	-0.889256000
1	2.651490000	-1.596650000	-1.418303000
1	0.960763000	-1.078016000	-1.484650000
1	4.093687000	1.466073000	0.885180000
1	1.338014000	2.329047000	0.743151000
1	2.223484000	-0.080780000	-2.244119000
6	0.818645000	-1.133576000	1.540016000
6	-0.318284000	-1.185253000	1.042895000
6	-1.367249000	-0.988738000	0.108968000
8	-1.568509000	-1.682252000	-0.877481000
8	-2.164857000	0.044327000	0.439178000
6	-3.268658000	0.290884000	-0.442186000
6	-4.020008000	1.481423000	0.096016000
1	1.472940000	-1.432928000	2.332977000
1	-2.887248000	0.474987000	-1.447630000
1	-3.897127000	-0.600015000	-0.480261000
1	-4.871129000	1.701023000	-0.548396000
1	-4.391346000	1.282334000	1.101346000
1	-3.377573000	2.361646000	0.129512000

3_TS_PMe3_2

15	-2.058392000	-0.008482000	-0.122888000
6	-1.102920000	1.348667000	-0.833533000
6	-3.751414000	0.648992000	-0.120027000
6	-1.599330000	0.058977000	1.622193000
1	-1.243012000	2.265135000	-0.255814000
1	-0.043149000	1.089211000	-0.834389000
1	-4.413915000	-0.043785000	0.399584000
1	-3.786953000	1.619860000	0.380908000
1	-2.192643000	-0.657579000	2.191424000
1	-0.545544000	-0.200879000	1.734377000
1	-4.106549000	0.763079000	-1.144481000
1	-1.415630000	1.523226000	-1.863686000
1	-1.765021000	1.060761000	2.025045000
6	-0.793216000	-1.857512000	-0.805243000
6	0.395354000	-1.667576000	-0.497655000
6	1.510024000	-1.006451000	0.079695000
8	1.844313000	-1.094710000	1.252005000
8	2.198161000	-0.279571000	-0.823262000
6	3.270796000	0.522101000	-0.308627000
6	2.738029000	1.810227000	0.276900000
1	-1.521404000	-2.506274000	-1.247149000
1	3.823998000	-0.053440000	0.432156000
1	3.916267000	0.712364000	-1.163660000
1	3.561443000	2.415224000	0.657877000
1	2.209686000	2.387834000	-0.482662000
1	2.054575000	1.602208000	1.101627000

3_TS_PMe3_3

15	-2.273519000	0.009618000	-0.158757000
6	-1.494591000	1.636729000	-0.095966000
6	-4.043323000	0.405982000	-0.065122000
6	-1.921563000	-0.663460000	1.479131000
1	-1.814160000	2.183973000	0.793439000
1	-0.409600000	1.524354000	-0.070676000
1	-4.622756000	-0.514152000	0.014776000
1	-4.253610000	1.036395000	0.802660000
1	-2.438630000	-1.614998000	1.608437000
1	-0.848985000	-0.837836000	1.578652000
1	-4.352125000	0.930656000	-0.969665000
1	-1.761972000	2.209906000	-0.984403000
1	-2.245338000	0.028500000	2.259764000
6	-0.722290000	-1.196739000	-1.426404000
6	0.411116000	-1.026788000	-0.948398000
6	1.430017000	-0.590956000	-0.063919000
8	1.736443000	-1.150827000	0.978901000
8	2.059134000	0.513233000	-0.512353000
6	3.158800000	0.990319000	0.276648000
6	4.420600000	0.217390000	-0.029341000
1	-1.327743000	-1.668883000	-2.172733000
1	3.262374000	2.039152000	0.005319000
1	2.897571000	0.922046000	1.332125000
1	5.252723000	0.626186000	0.544972000
1	4.301910000	-0.832734000	0.235818000
1	4.665923000	0.289056000	-1.089280000

3_TS_PPhMe2_1

15	0.725235000	-0.258647000	0.133709000
6	2.494183000	0.086306000	0.013490000
6	0.372356000	-1.294207000	-1.303295000
6	3.386176000	-0.979491000	-0.151841000
6	4.755294000	-0.756772000	-0.179413000
6	5.256946000	0.532630000	-0.028783000
6	4.380051000	1.595939000	0.148376000
6	3.007580000	1.376459000	0.170251000
1	3.007345000	-1.989592000	-0.258997000
1	5.432430000	-1.590200000	-0.316592000
1	6.325064000	0.706213000	-0.048604000
1	4.763074000	2.601786000	0.265279000
1	2.337773000	2.216612000	0.304775000
6	-0.783585000	-1.146623000	1.667576000
6	-1.929532000	-1.109086000	1.189817000
6	-2.961559000	-0.895392000	0.240208000
8	-3.285991000	-1.685786000	-0.633892000
8	-3.587219000	0.282866000	0.417397000
6	-4.663827000	0.563711000	-0.487589000
6	-5.211032000	1.922120000	-0.131707000
1	-0.157382000	-1.419511000	2.492860000
1	-5.420949000	-0.215470000	-0.389106000
1	-4.285090000	0.536088000	-1.510332000
1	-6.036621000	2.171768000	-0.797976000
1	-4.441994000	2.688052000	-0.234370000
1	-5.580245000	1.933842000	0.893796000
1	0.692467000	-0.806972000	-2.225587000
1	0.870315000	-2.258900000	-1.210443000
1	-0.704201000	-1.471038000	-1.341873000
6	-0.084140000	1.298203000	-0.281247000

1	0.051324000	2.030833000	0.513756000
1	0.293714000	1.701844000	-1.222025000
1	-1.153343000	1.104921000	-0.383293000

3_TS_PPhMe2_2

15	0.772132000	-0.143260000	0.139805000
6	2.563783000	0.049758000	0.016602000
6	0.341630000	-1.201804000	-1.258687000
6	3.363682000	-1.092807000	-0.098741000
6	4.746645000	-0.986906000	-0.128995000
6	5.354173000	0.261422000	-0.031323000
6	4.569028000	1.400863000	0.095349000
6	3.182895000	1.298042000	0.119854000
1	2.900802000	-2.070706000	-0.165932000
1	5.351535000	-1.879335000	-0.226975000
1	6.433098000	0.344037000	-0.053201000
1	5.034645000	2.375318000	0.170879000
1	2.585879000	2.196504000	0.213307000
6	-0.812672000	-0.841011000	1.692946000
6	-1.946815000	-0.751479000	1.194405000
6	-2.959409000	-0.530774000	0.226131000
8	-3.326354000	-1.353395000	-0.600317000
8	-3.503763000	0.697453000	0.326571000
6	-4.592420000	0.990888000	-0.561835000
6	-5.890918000	0.430286000	-0.030234000
1	-0.218436000	-1.108887000	2.543173000
1	-4.363660000	0.593755000	-1.550218000
1	-4.623111000	2.077353000	-0.615475000
1	-6.710929000	0.703619000	-0.695204000
1	-6.103645000	0.831141000	0.961144000
1	-5.844109000	-0.656597000	0.030331000
1	0.706203000	-0.779235000	-2.196209000
1	0.757961000	-2.199729000	-1.123955000
1	-0.745381000	-1.291840000	-1.300714000
6	0.095192000	1.458432000	-0.336558000
1	0.292666000	2.206078000	0.431119000
1	0.503278000	1.794527000	-1.291147000
1	-0.986598000	1.350987000	-0.431511000

3_TS_PPhMe2_3

15	-0.618360000	-0.286764000	-0.166559000
6	-2.392220000	0.024409000	-0.026042000
6	-0.129053000	-0.854264000	1.476917000
6	-3.217356000	-0.949911000	0.546901000
6	-4.591764000	-0.767214000	0.603319000
6	-5.166141000	0.386015000	0.077646000
6	-4.356391000	1.354426000	-0.503682000
6	-2.979048000	1.176359000	-0.556985000
1	-2.783268000	-1.856540000	0.952199000
1	-5.215792000	-1.526317000	1.057551000
1	-6.238213000	0.528012000	0.120934000
1	-4.795888000	2.254827000	-0.913805000
1	-2.362121000	1.942469000	-1.009933000
6	0.841848000	-1.523168000	-1.499144000
6	2.010182000	-1.335598000	-1.120075000
6	3.062396000	-0.842783000	-0.305454000
8	3.447038000	-1.365667000	0.729985000
8	3.626519000	0.269070000	-0.816735000
6	4.629456000	0.902351000	-0.009283000
6	3.990102000	1.758536000	1.060087000
1	0.182137000	-2.016273000	-2.184199000
1	5.211266000	1.504657000	-0.703990000
1	5.274110000	0.138953000	0.424055000
1	4.761304000	2.245217000	1.658048000
1	3.375879000	1.147997000	1.723572000
1	3.365445000	2.531434000	0.610449000
1	-0.441883000	-0.144494000	2.244374000
1	-0.557922000	-1.832799000	1.690268000
1	0.958169000	-0.951381000	1.495698000
6	0.135356000	1.349256000	-0.263567000
1	-0.077927000	1.820934000	-1.222260000
1	-0.209346000	1.992209000	0.548121000
1	1.216784000	1.224845000	-0.182531000

3_TS_PPh2Me_1

6	-0.323162000	-2.048616000	-1.582740000
6	-1.490487000	-2.005208000	-1.145730000
6	-2.475897000	-1.607461000	-0.199572000
8	-2.653176000	-2.143684000	0.885719000
8	-3.244324000	-0.598471000	-0.639649000
6	-4.266279000	-0.151111000	0.260014000
6	-5.033850000	0.946369000	-0.431323000
15	1.024886000	-0.847983000	-0.187527000
6	0.882894000	-1.921547000	1.254994000
6	2.712500000	-0.195060000	-0.095363000
6	-0.053316000	0.559445000	0.140516000
6	3.737406000	-0.969940000	0.455517000
6	5.049254000	-0.511697000	0.446588000
6	5.355921000	0.722066000	-0.114804000
6	4.342819000	1.496194000	-0.671879000
6	3.032319000	1.039842000	-0.667988000
6	-0.460810000	1.341611000	-0.942609000
6	-1.285527000	2.440281000	-0.746304000
6	-1.730165000	2.753002000	0.534500000
6	-1.346430000	1.967280000	1.615209000
6	-0.509232000	0.873907000	1.420257000
1	0.338107000	-2.455505000	-2.322410000
1	-4.910484000	-0.994482000	0.512812000
1	-3.797299000	0.201446000	1.180980000
1	-5.496531000	0.576996000	-1.346732000
1	-5.820162000	1.315741000	0.227090000
1	-4.376119000	1.778711000	-0.684510000
1	-0.174634000	-2.138505000	1.414054000
1	1.392211000	-2.863788000	1.051449000
1	1.308525000	-1.469844000	2.151560000
1	3.514452000	-1.932563000	0.898983000
1	5.831295000	-1.119525000	0.883561000
1	6.377534000	1.079274000	-0.118283000
1	4.573365000	2.459295000	-1.109160000
1	2.252087000	1.652569000	-1.104118000
1	-0.139811000	1.075024000	-1.943903000
1	-1.594308000	3.041818000	-1.591587000
1	-2.382825000	3.603066000	0.688395000
1	-1.699057000	2.202958000	2.611167000
1	-0.217391000	0.268152000	2.269304000

3_TS_PPh2Me_2

6	1.081366000	-0.202933000	2.129713000
6	2.257884000	-0.183457000	1.723771000
6	3.287484000	-0.138680000	0.749565000
8	3.677944000	0.882673000	0.200662000
8	3.822925000	-1.349113000	0.508049000
6	4.843438000	-1.410089000	-0.498664000
6	4.238537000	-1.451633000	-1.883201000
15	-0.454152000	-0.214502000	0.377049000
6	0.657749000	-0.669837000	-0.970549000
6	-1.971346000	-1.153736000	0.083187000
6	-0.870882000	1.508029000	0.039778000
6	-2.519059000	-1.881770000	1.138467000
6	-3.680337000	-2.625226000	0.952372000
6	-4.297341000	-2.643972000	-0.291345000
6	-3.757571000	-1.918202000	-1.350819000
6	-2.600796000	-1.175680000	-1.165263000
6	0.180062000	2.425528000	-0.081257000
6	-0.083829000	3.774151000	-0.273236000
6	-1.397971000	4.228502000	-0.331089000
6	-2.445966000	3.326959000	-0.191769000
6	-2.186290000	1.973821000	-0.005783000
1	0.428701000	-0.168247000	2.978909000
1	5.510906000	-0.556998000	-0.382322000
1	5.395125000	-2.322622000	-0.281616000
1	5.028207000	-1.559445000	-2.627709000
1	3.558308000	-2.298943000	-1.979404000
1	3.691279000	-0.532642000	-2.093330000
1	1.449622000	0.074033000	-1.056934000
1	1.106232000	-1.639944000	-0.750490000
1	0.124945000	-0.719115000	-1.920176000
1	-2.031778000	-1.868931000	2.106536000
1	-4.098488000	-3.188669000	1.776330000
1	-5.199902000	-3.223007000	-0.439148000
1	-4.241034000	-1.930911000	-2.319186000
1	-2.189663000	-0.601912000	-1.988442000
1	1.209503000	2.086729000	-0.013668000
1	0.737821000	4.472030000	-0.371186000
1	-1.602555000	5.281086000	-0.478652000
1	-3.470505000	3.674502000	-0.229947000
1	-3.012168000	1.281408000	0.101752000

3_TS_PPh2Me_3

6	-0.419294000	-1.939002000	-1.591043000
6	-1.587173000	-1.791435000	-1.178522000
6	-2.550065000	-1.294479000	-0.256057000
8	-2.795678000	-1.803908000	0.828797000
8	-3.201581000	-0.215839000	-0.721513000
6	-4.206045000	0.348016000	0.132302000
6	-5.514613000	-0.395968000	-0.003753000
15	0.994191000	-0.830359000	-0.183829000
6	0.733669000	-1.863038000	1.271778000
6	2.726260000	-0.311314000	-0.064640000
6	0.022734000	0.662607000	0.096426000
6	3.673566000	-1.144623000	0.537163000
6	5.017297000	-0.790228000	0.548872000
6	5.433572000	0.396367000	-0.042490000
6	4.498081000	1.228059000	-0.650185000
6	3.156209000	0.875227000	-0.666886000
6	-0.286038000	1.463225000	-1.005387000
6	-1.037311000	2.619477000	-0.846994000
6	-1.507139000	2.972804000	0.413782000
6	-1.221405000	2.170537000	1.512694000
6	-0.457378000	1.019383000	1.356149000
1	0.219822000	-2.408504000	-2.312864000

1	-3.846498000	0.336834000	1.161435000
1	-4.303030000	1.381890000	-0.195380000
1	-5.857155000	-0.381767000	-1.038845000
1	-5.403376000	-1.431191000	0.317245000
1	-6.275853000	0.078256000	0.616839000
1	-0.341219000	-1.996981000	1.404297000
1	1.175653000	-2.844480000	1.098361000
1	1.167975000	-1.427974000	2.172455000
1	3.366154000	-2.071970000	1.004310000
1	5.738344000	-1.442435000	1.025017000
1	6.479922000	0.672626000	-0.030037000
1	4.814011000	2.155206000	-1.111135000
1	2.437438000	1.531945000	-1.142635000
1	0.052026000	1.165656000	-1.992365000
1	-1.269285000	3.234839000	-1.706655000
1	-2.102417000	3.868483000	0.538270000
1	-1.594162000	2.438768000	2.492995000
1	-0.242709000	0.400478000	2.218611000

3_TS_PPh3_1

15	0.828685000	-0.030383000	-0.568793000
6	-0.404345000	-0.613014000	0.601656000
6	2.227501000	-1.159088000	-0.428042000
6	1.412459000	1.555886000	0.054503000
6	-0.662855000	0.000390000	1.827007000
6	-1.622955000	-0.529367000	2.681902000
6	-2.320551000	-1.677420000	2.322848000
6	-2.067755000	-2.292838000	1.100681000
6	-1.123471000	-1.754814000	0.238017000
6	3.225708000	-1.060155000	-1.403131000
6	4.329458000	-1.899829000	-1.365633000
6	4.438489000	-2.858929000	-0.363061000
6	3.444837000	-2.970159000	0.602346000
6	2.343029000	-2.122542000	0.574566000
6	2.698295000	1.745841000	0.562443000
6	3.097449000	3.003231000	1.001560000
6	2.216466000	4.076153000	0.944391000
6	0.930600000	3.891469000	0.444986000
6	0.530739000	2.641415000	-0.005565000
1	-0.115814000	0.889448000	2.115933000
1	-1.821349000	-0.047092000	3.630336000
1	-3.064923000	-2.089480000	2.992388000
1	-2.613266000	-3.183224000	0.815637000
1	-0.939365000	-2.218845000	-0.725408000
1	3.135894000	-0.318331000	-2.189981000
1	5.099415000	-1.812072000	-2.121216000
1	5.295096000	-3.520075000	-0.337373000
1	3.527997000	-3.715038000	1.383382000
1	1.576626000	-2.209880000	1.335007000
1	3.389886000	0.914422000	0.619871000
1	4.097254000	3.140622000	1.393083000
1	2.529567000	5.053761000	1.287839000
1	0.239235000	4.723135000	0.400864000
1	-0.473554000	2.502896000	-0.393264000
6	-0.471062000	0.387545000	-2.453925000
6	-1.665345000	0.634817000	-2.193382000
6	-2.729769000	0.867381000	-1.279856000
8	-2.877378000	1.900210000	-0.641005000
8	-3.605954000	-0.148442000	-1.221138000
6	-4.668362000	0.000663000	-0.271097000
6	-5.502032000	-1.253829000	-0.319951000
1	0.272302000	0.316394000	-3.223728000
1	-5.254729000	0.884058000	-0.527760000
1	-4.235866000	0.160223000	0.718969000
1	-6.325650000	-1.176096000	0.389818000
1	-4.902792000	-2.126910000	-0.058719000
1	-5.918751000	-1.403158000	-1.316214000

3_TS_PPh3_2

15	0.847769000	-0.022527000	-0.550575000
6	-0.281684000	-0.673948000	0.683490000
6	2.330349000	-1.044462000	-0.467834000
6	1.329862000	1.613457000	0.026712000
6	-0.453888000	-0.110065000	1.947901000
6	-1.344183000	-0.681130000	2.848635000
6	-2.060892000	-1.819822000	2.495148000
6	-1.890981000	-2.388326000	1.237295000
6	-1.010729000	-1.812212000	0.331332000
6	3.267079000	-0.904016000	-1.496968000
6	4.425289000	-1.667972000	-1.500680000
6	4.649724000	-2.593631000	-0.485970000
6	3.716763000	-2.747405000	0.532722000
6	2.561073000	-1.974882000	0.546240000
6	2.637470000	1.945698000	0.381092000
6	2.943931000	3.241181000	0.783559000
6	1.949112000	4.209055000	0.842684000
6	0.641180000	3.881718000	0.495872000
6	0.332134000	2.594349000	0.082021000
1	0.104547000	0.774341000	2.229023000
1	-1.475944000	-0.238586000	3.827517000
1	-2.755057000	-2.261215000	3.198907000
1	-2.453086000	-3.269993000	0.957030000
1	-0.891795000	-2.238497000	-0.659092000
1	3.085773000	-0.190328000	-2.293533000
1	5.147454000	-1.548238000	-2.297891000
1	5.548890000	-3.196098000	-0.492296000
1	3.889019000	-3.467341000	1.322449000
1	1.841561000	-2.095038000	1.346951000
1	3.418256000	1.195892000	0.348867000
1	3.961461000	3.489987000	1.056486000
1	2.190211000	5.215999000	1.158243000
1	-0.138346000	4.631144000	0.543668000
1	-0.688930000	2.340921000	-0.186668000
6	-0.557967000	0.343448000	-2.355258000
6	-1.766457000	0.417628000	-2.054584000
6	-2.840283000	0.432718000	-1.123562000
8	-3.082801000	1.361257000	-0.365106000
8	-3.606833000	-0.669972000	-1.196921000
6	-4.698762000	-0.753012000	-0.271776000
6	-5.912763000	-0.021838000	-0.797046000
1	0.161449000	0.440279000	-3.145096000
1	-4.380908000	-0.354111000	0.691857000
1	-4.895977000	-1.818644000	-0.164253000
1	-6.744091000	-0.136911000	-0.100468000
1	-6.215350000	-0.426060000	-1.763483000
1	-5.700871000	1.040938000	-0.909518000

3_TS_PPh3_3

15	0.793745000	0.022390000	-0.569867000
6	2.342186000	-0.897526000	-0.499962000
6	1.138135000	1.632304000	0.160682000
6	-0.319453000	-0.812632000	0.566079000
6	2.590373000	-1.922483000	0.413811000
6	3.801375000	-2.604956000	0.382969000
6	4.771879000	-2.267847000	-0.553014000
6	4.530496000	-1.246975000	-1.467669000
6	3.318821000	-0.571777000	-1.447319000
6	0.097818000	2.568573000	0.174514000
6	0.300895000	3.829577000	0.716709000
6	1.545181000	4.175030000	1.235827000
6	2.582963000	3.251256000	1.219018000
6	2.381900000	1.982118000	0.687653000
6	-0.529346000	-0.395448000	1.880381000
6	-1.316045000	-1.159212000	2.735522000
6	-1.889252000	-2.342687000	2.284970000
6	-1.697124000	-2.755144000	0.969966000
6	-0.926457000	-1.986483000	0.109328000
1	1.842003000	-2.186604000	1.151149000
1	3.986857000	-3.398571000	1.095320000
1	5.713469000	-2.801194000	-0.572811000
1	5.282238000	-0.983523000	-2.200336000
1	3.126655000	0.217960000	-2.165986000
1	-0.874133000	2.305846000	-0.231266000
1	-0.511763000	4.544482000	0.728623000
1	1.703991000	5.161625000	1.651887000
1	3.551265000	3.513974000	1.625414000
1	3.194498000	1.266183000	0.689298000
1	-0.068073000	0.515994000	2.240919000
1	-1.472525000	-0.833464000	3.755767000
1	-2.496009000	-2.938640000	2.954836000
1	-2.156295000	-3.668067000	0.613176000
1	-0.779481000	-2.299934000	-0.918878000
6	-0.495370000	0.369280000	-2.473764000
6	-1.715908000	0.492471000	-2.257758000
6	-2.883805000	0.635393000	-1.465763000
8	-3.166378000	1.650655000	-0.842557000
8	-3.698538000	-0.433755000	-1.522666000
6	-4.932149000	-0.327142000	-0.797826000
6	-4.743357000	-0.684020000	0.658193000
1	0.279154000	0.394409000	-3.215520000
1	-5.609902000	-1.021769000	-1.290669000
1	-5.319882000	0.684507000	-0.909952000
1	-5.679012000	-0.537125000	1.199415000
1	-3.978156000	-0.050713000	1.110289000
1	-4.444290000	-1.726439000	0.763825000

3_TS_Pani3_1

6	-0.402981000	0.099078000	2.906024000
6	-1.618345000	-0.178946000	2.903225000
6	-2.816816000	-0.621705000	2.277278000
8	-3.358652000	-1.700677000	2.453909000
8	-3.334585000	0.327287000	1.471966000
6	-4.498442000	-0.040766000	0.723356000
6	-4.790190000	1.090589000	-0.229337000
15	0.637382000	-0.037979000	0.813671000
6	0.605559000	1.643109000	0.172558000
6	2.256051000	-0.693378000	0.377886000
6	-0.556514000	-0.951215000	-0.160789000
6	1.672018000	2.224295000	-0.521392000
6	1.592669000	3.527463000	-0.977976000

6	0.438145000	4.282287000	-0.758324000
6	-0.637071000	3.716089000	-0.071479000
6	-0.539981000	2.410871000	0.392871000
6	2.487517000	-1.491921000	-0.748123000
6	3.757697000	-1.955646000	-1.034088000
6	4.832295000	-1.626205000	-0.202802000
6	4.617726000	-0.834551000	0.925835000
6	3.333653000	-0.385096000	1.207518000
6	-0.931093000	-2.213308000	0.294932000
6	-1.842235000	-2.995503000	-0.404411000
6	-2.408396000	-2.498961000	-1.577913000
6	-2.044439000	-1.231058000	-2.044628000
6	-1.130041000	-0.469082000	-1.343342000
8	0.444142000	5.542956000	-1.238908000
8	-3.320049000	-3.166231000	-2.313926000
8	6.037202000	-2.115526000	-0.559415000
6	-0.708005000	6.339164000	-1.035324000
6	-3.748843000	-4.434542000	-1.853472000
6	7.154152000	-1.791106000	0.247182000
1	0.416099000	0.474001000	3.488162000
1	-4.295151000	-0.972073000	0.190709000
1	-5.327323000	-0.218371000	1.410806000
1	-4.992956000	2.013761000	0.314280000
1	-5.661994000	0.849406000	-0.837463000
1	-3.940189000	1.256936000	-0.894192000
1	2.572789000	1.653017000	-0.710810000
1	2.416019000	3.981165000	-1.514710000
1	-1.541050000	4.279655000	0.111072000
1	-1.380513000	1.979078000	0.926286000
1	1.666500000	-1.750384000	-1.406255000
1	3.944408000	-2.573727000	-1.902966000
1	5.432038000	-0.575131000	1.587010000
1	3.172588000	0.223181000	2.091290000
1	-0.513247000	-2.592108000	1.221956000
1	-2.119219000	-3.965650000	-0.017866000
1	-2.495515000	-0.864131000	-2.957889000
1	-0.858660000	0.511110000	-1.717072000
1	-0.500928000	7.304097000	-1.489897000
1	-1.583801000	5.895421000	-1.514394000
1	-0.908781000	6.477383000	0.029492000
1	-4.483067000	-4.791402000	-2.570486000
1	-2.915958000	-5.139830000	-1.808175000
1	-4.212783000	-4.357619000	-0.867419000
1	7.030799000	-2.171956000	1.263498000
1	8.015705000	-2.267376000	-0.212557000
1	7.314468000	-0.710985000	0.281946000

3_TS_Pani3_2

6	-0.399718000	0.106597000	2.942479000
6	-1.628396000	-0.086079000	2.973290000
6	-2.897193000	-0.396893000	2.416360000
8	-3.438090000	-1.490554000	2.448391000
8	-3.480282000	0.693862000	1.874700000
6	-4.712057000	0.478348000	1.174063000
6	-4.432277000	-0.022323000	-0.224105000
15	0.583551000	0.014738000	0.788462000
6	0.494611000	1.665460000	0.075266000
6	2.240088000	-0.585429000	0.422758000
6	-0.536175000	-0.982685000	-0.193310000
6	1.516604000	2.242877000	-0.685310000
6	1.376904000	3.516677000	-1.205922000
6	0.205313000	4.244331000	-0.984072000
6	-0.826001000	3.680493000	-0.231538000
6	-0.668853000	2.404701000	0.294569000
6	2.513794000	-1.580979000	-0.521364000
6	3.808376000	-2.021381000	-0.727727000
6	4.865298000	-1.472933000	0.002876000
6	4.609405000	-0.480588000	0.950439000
6	3.303196000	-0.056274000	1.155909000
6	-1.013608000	-2.172809000	0.351206000
6	-1.801735000	-3.043745000	-0.392651000
6	-2.127257000	-2.715561000	-1.708239000
6	-1.661410000	-1.518336000	-2.264934000
6	-0.878432000	-0.664395000	-1.514775000
8	0.152315000	5.478022000	-1.528158000
8	-2.890700000	-3.486087000	-2.507755000
8	6.095429000	-1.958151000	-0.264457000
6	-1.016627000	6.248281000	-1.320265000
6	-3.404147000	-4.694280000	-1.977264000
6	7.192347000	-1.428644000	0.456109000
1	0.480488000	0.355222000	3.501821000
1	-5.326461000	-0.224505000	1.735463000
1	-5.205234000	1.448478000	1.161165000
1	-3.814969000	0.694506000	-0.769011000
1	-5.365969000	-0.161421000	-0.770401000
1	-3.906077000	-0.977491000	-0.184780000
1	2.428895000	1.691203000	-0.877306000
1	2.165124000	3.967833000	-1.795059000
1	-1.741110000	4.224268000	-0.045015000
1	-1.474355000	1.975895000	0.882179000
1	1.707812000	-2.014985000	-1.100997000
1	4.025544000	-2.791327000	-1.456992000
1	5.409776000	-0.044462000	1.530888000
1	3.112619000	0.710283000	1.900040000
1	-0.770254000	-2.427187000	1.376657000
1	-2.163223000	-3.955110000	0.061493000
1	-1.930251000	-1.281656000	-3.286502000
1	-0.520553000	0.258044000	-1.957695000
1	-0.858325000	7.193952000	-1.831366000
1	-1.895335000	5.753825000	-1.740789000
1	-1.181150000	6.436053000	-0.256705000
1	-3.987985000	-5.155455000	-2.769039000
1	-2.597552000	-5.369385000	-1.682875000
1	-4.048613000	-4.502206000	-1.116487000
1	7.083724000	-1.608383000	1.528129000
1	8.077634000	-1.944053000	0.093900000
1	7.300860000	-0.356340000	0.277494000

3_TS_Pani3_3

6	0.600635000	0.194038000	-3.054784000
6	1.827083000	-0.013308000	-3.025013000
6	3.065186000	-0.362750000	-2.425308000
8	3.588899000	-1.464317000	-2.470374000
8	3.643721000	0.700063000	-1.825069000
6	4.840689000	0.439373000	-1.081028000
6	4.492942000	-0.104355000	0.285674000
15	-0.490875000	0.048514000	-0.964932000
6	-0.416636000	1.682494000	-0.214879000
6	-2.165518000	-0.545913000	-0.684449000
6	0.578344000	-0.980971000	0.040263000
6	-1.485273000	2.287244000	0.438649000
6	-1.370173000	3.563795000	0.983979000
6	-0.161333000	4.248269000	0.883907000
6	0.923678000	3.647770000	0.234486000
6	0.792798000	2.388463000	-0.312881000
6	-2.484855000	-1.549795000	0.224982000
6	-3.793618000	-2.001649000	0.371046000
6	-4.807256000	-1.437983000	-0.400803000
6	-4.499377000	-0.428756000	-1.320590000
6	-3.196315000	0.000412000	-1.464430000
6	1.046628000	-2.184365000	-0.502877000
6	1.782986000	-3.066977000	0.262761000
6	2.080098000	-2.758973000	1.594130000
6	1.630184000	-1.559141000	2.147811000
6	0.885785000	-0.683551000	1.366774000
8	0.049857000	5.485364000	1.379540000
8	2.813458000	-3.666819000	2.267993000
8	-6.103267000	-1.805352000	-0.329710000
6	-1.026810000	6.136152000	2.026773000
6	3.174136000	-3.375424000	3.605333000
6	-6.456878000	-2.828510000	0.581870000
1	-0.242374000	0.473296000	-3.655434000
1	5.467268000	-0.254677000	-1.639876000
1	5.348242000	1.399770000	-1.013299000
1	3.867844000	0.605909000	0.830303000
1	5.400247000	-0.283104000	0.864183000
1	3.950486000	-1.046074000	0.188621000
1	-2.428858000	1.763979000	0.535418000
1	-2.220770000	4.006092000	1.482953000
1	1.855676000	4.193950000	0.165035000
1	1.639489000	1.936634000	-0.819839000
1	-1.707728000	-1.994461000	0.835298000
1	-4.007829000	-2.784159000	1.085019000
1	-5.298168000	-0.005149000	-1.915624000
1	-2.969132000	0.777363000	-2.186946000
1	0.831388000	-2.424347000	-1.538057000
1	2.154963000	-3.994411000	-0.152894000
1	1.854049000	-1.300847000	3.172993000
1	0.534412000	0.242847000	1.806630000
1	-0.658421000	7.110492000	2.335951000
1	-1.872275000	6.268402000	1.347781000
1	-1.353666000	5.578400000	2.907381000
1	3.770908000	-4.213934000	3.953573000
1	3.768861000	-2.460641000	3.660738000
1	2.290834000	-3.273520000	4.239797000
1	-6.221860000	-2.539739000	1.608935000
1	-7.529647000	-2.971382000	0.485308000
1	-5.944928000	-3.763461000	0.342612000

3_TS_Ppfp3_1

15	-0.676816000	-0.026662000	0.752459000
6	0.451539000	-0.945495000	-0.297085000
6	-2.324310000	-0.665426000	0.399186000
6	-0.662317000	1.661902000	0.125762000
6	0.961292000	-0.449742000	-1.498515000
6	1.813117000	-1.223294000	-2.274583000
6	2.137808000	-2.492091000	-1.827683000
6	1.656871000	-3.015341000	-0.641642000
6	0.813747000	-2.226328000	0.126683000

6	-3.361614000	-0.279487000	1.254907000
6	-4.655124000	-0.733896000	1.052147000
6	-4.887100000	-1.593169000	-0.007673000
6	-3.886867000	-2.007222000	-0.866676000
6	-2.597989000	-1.533763000	-0.658557000
6	-1.748795000	2.242012000	-0.529813000
6	-1.683350000	3.553756000	-0.982595000
6	-0.516389000	4.263688000	-0.776371000
6	0.583848000	3.722215000	-0.134409000
6	0.500190000	2.417087000	0.324217000
1	0.692283000	0.543255000	-1.836832000
1	2.219376000	-0.859502000	-3.208680000
1	1.953784000	-4.006285000	-0.326908000
1	0.439336000	-2.605393000	1.071145000
1	-3.155915000	0.386110000	2.085772000
1	-5.470308000	-0.442729000	1.700330000
1	-4.119317000	-2.681014000	-1.680116000
1	-1.805537000	-1.844206000	-1.327894000
1	-2.654466000	1.672773000	-0.696656000
1	-2.515828000	4.019957000	-1.491843000
1	1.476094000	4.317427000	0.004885000
1	1.350878000	1.975561000	0.831661000
6	0.421262000	0.045748000	2.780866000
6	1.638202000	-0.234088000	2.736653000
6	2.794533000	-0.658934000	2.022980000
8	3.334961000	-1.747896000	2.121033000
8	3.268727000	0.318854000	1.225619000
6	4.374069000	-0.027829000	0.383147000
6	4.595272000	1.128433000	-0.558561000
1	-0.371935000	0.397971000	3.412197000
1	4.130836000	-0.945610000	-0.155829000
1	5.251339000	-0.222393000	1.002338000
1	4.833731000	2.038407000	-0.007327000
1	3.700823000	1.308910000	-1.158529000
1	5.422983000	0.906144000	-1.231936000
9	-6.135447000	-2.046098000	-0.206166000
9	-0.446151000	5.530948000	-1.215409000
9	2.963511000	-3.242577000	-2.574001000

3_TS_Ppfp3_2

15	0.704948000	-0.027786000	-0.678587000
6	-0.501632000	-0.682287000	0.476520000
6	2.190544000	-1.029036000	-0.483972000
6	1.131540000	1.614519000	-0.077323000
6	-0.799141000	-0.088551000	1.703393000
6	-1.758977000	-0.641448000	2.539689000
6	-2.402407000	-1.794376000	2.127735000
6	-2.129453000	-2.416429000	0.922932000
6	-1.178890000	-1.842875000	0.092498000
6	3.190611000	-0.893252000	-1.452816000
6	4.358748000	-1.635321000	-1.376442000
6	4.498654000	-2.526744000	-0.327214000
6	3.528372000	-2.698614000	0.641861000
6	2.368993000	-1.938621000	0.559510000
6	2.404069000	1.950062000	0.385919000
6	2.685791000	3.244709000	0.803707000
6	1.675416000	4.185372000	0.756911000
6	0.399309000	3.889424000	0.309969000
6	0.134522000	2.597540000	-0.115691000
1	-0.285389000	0.813092000	2.011886000
1	-2.009341000	-0.194383000	3.492058000
1	-2.667417000	-3.311775000	0.642154000
1	-0.967650000	-2.295079000	-0.870219000
1	3.055765000	-0.196274000	-2.272549000
1	5.144667000	-1.539803000	-2.113203000
1	3.685121000	-3.409432000	1.441821000
1	1.603335000	-2.057248000	1.315870000
1	3.185862000	1.202342000	0.427971000
1	3.666456000	3.522949000	1.165129000
1	-0.360461000	4.659074000	0.294810000
1	-0.861477000	2.345734000	-0.465938000
6	-0.593649000	0.311669000	-2.555997000
6	-1.815825000	0.392027000	-2.315105000
6	-2.928523000	0.425201000	-1.431113000
8	-3.200344000	1.368702000	-0.701158000
8	-3.693130000	-0.677430000	-1.515223000
6	-4.824749000	-0.741840000	-0.636678000
6	-6.013657000	-0.022561000	-1.230825000
1	0.160912000	0.395709000	-3.314001000
1	-4.550106000	-0.321757000	0.331338000
1	-5.026527000	-1.805104000	-0.515020000
1	-6.273505000	-0.448157000	-2.200495000
1	-5.795746000	1.037458000	-1.357285000
1	-6.874940000	-0.122101000	-0.569232000
9	1.941190000	5.438218000	1.162382000
9	5.622357000	-3.257621000	-0.250363000
9	-3.338434000	-2.329491000	2.927975000

3_TS_Ppfp3_3

6	0.527622000	0.352341000	-2.642742000
6	1.754931000	0.476278000	-2.460913000
6	2.953176000	0.623386000	-1.717224000
8	3.244964000	1.633383000	-1.088545000
8	3.784894000	-0.427863000	-1.831826000
6	5.076031000	-0.286669000	-1.220731000
6	5.043451000	-0.653552000	0.244528000
15	-0.678093000	0.004966000	-0.711684000
6	0.534444000	-0.750106000	0.373188000
6	-1.021949000	1.631183000	-0.020224000
6	-2.192072000	-0.949836000	-0.501439000
6	0.843071000	-0.262556000	1.643103000
6	1.747709000	-0.934708000	2.454722000
6	2.323534000	-2.094975000	1.972657000
6	2.044153000	-2.608479000	0.718628000
6	1.152979000	-1.917133000	-0.086152000
6	-2.250838000	1.971890000	0.546401000
6	-2.471044000	3.250970000	1.041404000
6	-1.443480000	4.171108000	0.966323000
6	-0.209414000	3.869174000	0.417554000
6	-0.006024000	2.593020000	-0.083509000
6	-3.223643000	-0.729459000	-1.420610000
6	-4.415690000	-1.431024000	-1.332084000
6	-4.549020000	-2.367150000	-0.321567000
6	-3.548923000	-2.622287000	0.597462000
6	-2.364778000	-1.902778000	0.503729000
9	3.211697000	-2.741149000	2.744849000
9	-5.696545000	-3.058743000	-0.233685000
9	-1.649845000	5.408837000	1.446061000
1	-0.261344000	0.392501000	-3.368752000
1	5.726066000	-0.956852000	-1.780350000
1	5.419749000	0.737349000	-1.361481000
1	4.785826000	-1.704501000	0.376335000
1	6.026254000	-0.484358000	0.686653000
1	4.314585000	-0.039758000	0.775850000
1	0.373560000	0.642140000	2.008735000
1	2.005963000	-0.572845000	3.440588000
1	2.534995000	-3.511904000	0.383039000
1	0.930654000	-2.287423000	-1.080900000
1	-3.046106000	1.240142000	0.610008000
1	-3.417103000	3.532397000	1.483592000
1	0.566116000	4.622314000	0.382779000
1	0.956653000	2.340838000	-0.516395000
1	-3.093976000	0.001918000	-2.210754000
1	-5.225203000	-1.270760000	-2.031011000
1	-3.701848000	-3.365111000	1.368573000
1	-1.575438000	-2.087056000	1.221681000

3_TS_PCy3_1

6	0.603407000	0.579376000	2.486613000
6	1.823735000	0.411083000	2.339530000
6	3.029825000	-0.000307000	1.715337000
8	3.543851000	-1.101900000	1.837160000
8	3.585466000	0.988717000	0.986941000
6	4.782383000	0.662874000	0.268495000
6	4.457771000	-0.059899000	-1.018718000
15	-0.684053000	0.009954000	0.514487000
6	-2.351359000	-0.763691000	0.327595000
6	-0.711490000	1.538034000	-0.494002000
6	0.472911000	-1.120252000	-0.327391000
6	-3.170808000	-0.580526000	1.609585000
6	-4.487256000	-1.344519000	1.532421000
6	-5.293201000	-0.918018000	0.312003000
6	-4.477617000	-1.110097000	-0.960335000
6	-3.169343000	-0.327757000	-0.889405000
6	-1.480001000	2.615765000	0.278079000
6	-1.564790000	3.908983000	-0.523152000
6	-0.168440000	4.411286000	-0.867419000
6	0.611780000	3.345144000	-1.626320000
6	0.691285000	2.045303000	-0.830876000
6	0.632869000	-2.425357000	0.456500000
6	1.685486000	-3.316658000	-0.194512000
6	1.347942000	-3.592950000	-1.654674000
6	1.188933000	-2.290190000	-2.429542000
6	0.120324000	-1.413191000	-1.785135000
1	-0.199026000	0.906381000	3.114767000
1	5.262107000	1.620272000	0.074549000
1	5.431537000	0.064036000	0.906572000
1	3.987210000	-1.020746000	-0.808506000
1	3.783073000	0.539090000	-1.633686000
1	5.371432000	-0.240262000	-1.586332000
1	-2.140397000	-1.837058000	0.221966000
1	-1.234789000	1.336227000	-1.436985000
1	1.443139000	-0.601988000	-0.302556000
1	-2.586292000	-0.907411000	2.473485000
1	-3.376743000	0.488060000	1.749459000
1	-4.269507000	-2.416412000	1.459644000
1	-5.063909000	-1.195296000	2.447677000
1	-6.228859000	-1.478118000	0.255849000
1	-5.557242000	0.141607000	0.409239000

1 -4.251021000 -2.175320000 -1.085238000
1 -5.051434000 -0.798546000 -1.835874000
1 -2.596042000 -0.458292000 -1.809730000
1 -3.401622000 0.740096000 -0.804900000
1 -2.478907000 2.258844000 0.544378000
1 -0.947489000 2.807686000 1.218550000
1 -2.120412000 3.720563000 -1.449239000
1 -2.119266000 4.664346000 0.037527000
1 -0.224009000 5.332277000 -1.451482000
1 0.362586000 4.649391000 0.061358000
1 0.110143000 3.146888000 -2.580773000
1 1.617926000 3.699659000 -1.860261000
1 1.247336000 2.215569000 0.098166000
1 1.245700000 1.295374000 -1.401718000
1 0.904945000 -2.208186000 1.492339000
1 -0.325729000 -2.957660000 0.471586000
1 2.655353000 -2.811095000 -0.133632000
1 1.778124000 -4.252057000 0.361532000
1 2.117007000 -4.219106000 -2.111855000
1 0.405965000 -4.151933000 -1.706015000
1 2.142473000 -1.748175000 -2.427292000
1 0.935009000 -2.491065000 -3.472616000
1 -0.838915000 -1.945031000 -1.812114000
1 -0.009263000 -0.483067000 -2.346016000

3_TS_PCy3_2

6 0.575420000 0.496903000 2.503790000
6 1.782740000 0.227192000 2.396646000
6 2.936953000 -0.320929000 1.776585000
8 3.402995000 -1.426137000 2.004575000
8 3.497948000 0.533880000 0.899357000
6 4.608814000 0.029206000 0.149624000
6 4.961264000 1.069170000 -0.883092000
15 -0.709973000 -0.012171000 0.518832000
6 -2.387776000 -0.745750000 0.279382000
6 -0.693028000 1.557938000 -0.425426000
6 0.439313000 -1.120397000 -0.359650000
6 -3.229336000 -0.586478000 1.550224000
6 -4.557343000 -1.323205000 1.422335000
6 -5.330301000 -0.844622000 0.199862000
6 -4.492523000 -1.012340000 -1.061558000
6 -3.172463000 -0.256963000 -0.939454000
6 -1.452226000 2.620289000 0.376553000
6 -1.493669000 3.946636000 -0.372443000
6 -0.080522000 4.431833000 -0.669855000
6 0.689845000 3.381475000 -1.460015000
6 0.725859000 2.047479000 -0.720317000
6 0.570748000 -2.468967000 0.352230000
6 1.649778000 -3.316743000 -0.314261000
6 1.360984000 -3.509003000 -1.798177000
6 1.214663000 -2.164235000 -2.500138000
6 0.120343000 -1.332822000 -1.838788000
1 -0.207523000 0.922843000 3.096588000
1 5.439835000 -0.170399000 0.827916000
1 4.320916000 -0.916644000 -0.315615000
1 5.234300000 2.011172000 -0.407362000
1 5.805567000 0.726601000 -1.481047000
1 4.116015000 1.248179000 -1.550197000
1 -2.194477000 -1.819311000 0.145406000
1 -1.205027000 1.405625000 -1.383877000
1 1.414640000 -0.617344000 -0.290388000
1 -2.668435000 -0.950588000 2.414769000
1 -3.418694000 0.480963000 1.719125000
1 -4.357060000 -2.396149000 1.320713000
1 -5.150188000 -1.191723000 2.329935000
1 -6.274498000 -1.385277000 0.107733000
1 -5.577187000 0.216227000 0.324845000
1 -4.282695000 -2.077157000 -1.215595000
1 -5.042624000 -0.662860000 -1.937995000
1 -2.582025000 -0.368704000 -1.851322000
1 -3.387994000 0.811688000 -0.825674000
1 -2.463667000 2.275405000 0.609510000
1 -0.934193000 2.763592000 1.333496000
1 -2.034982000 3.808434000 -1.315699000
1 -2.042837000 4.690490000 0.208452000
1 -0.105330000 5.377817000 -1.214735000
1 0.438081000 4.618118000 0.277595000
1 0.202366000 3.236367000 -2.431195000
1 1.708612000 3.721367000 -1.659957000
1 1.271842000 2.163502000 0.222847000
1 1.272151000 1.310637000 -1.315093000
1 0.801743000 -2.317026000 1.409622000
1 -0.386293000 -3.001004000 0.296755000
1 2.613921000 -2.809801000 -0.193822000
1 1.732524000 -4.282126000 0.189543000
1 2.149654000 -4.101797000 -2.266238000
1 0.426190000 -4.070431000 -1.912607000
1 2.163345000 -1.615797000 -2.434178000
1 0.997064000 -2.304550000 -3.561092000
1 -0.833523000 -1.868993000 -1.917901000
1 -0.002669000 -0.374732000 -2.351543000

3_TS_PCy3_3

6 -0.787348000 0.884048000 -2.094243000
6 -1.979776000 0.883378000 -1.751223000
6 -3.140491000 0.701906000 -0.958288000
8 -3.497944000 1.449972000 -0.058687000
8 -3.847601000 -0.384054000 -1.328425000
6 -5.092194000 -0.603119000 -0.650520000
6 -6.194597000 0.223805000 -1.270933000
15 0.706401000 0.006744000 -0.421970000
6 2.137722000 -1.157670000 -0.500344000
6 1.301296000 1.501725000 0.452952000
6 -0.498536000 -0.770153000 0.698192000
6 2.725773000 -1.198330000 -1.914925000
6 3.803282000 -2.270502000 -2.028173000
6 4.900553000 -2.050871000 -0.994531000
6 4.312419000 -2.018571000 0.410475000
6 3.247945000 -0.931475000 0.527404000
6 2.144500000 2.332677000 -0.520035000
6 2.694073000 3.581106000 0.158859000
6 1.555452000 4.426592000 0.715043000
6 0.703361000 3.609342000 1.677879000
6 0.156812000 2.352705000 1.006526000
6 -1.139859000 -2.005089000 0.060467000
6 -2.272891000 -2.512802000 0.945943000
6 -1.760930000 -2.847932000 2.342104000
6 -1.086576000 -1.636144000 2.975350000
6 0.038725000 -1.116786000 2.085740000
1 -0.060667000 1.155028000 -2.832079000
1 -5.286337000 -1.669140000 -0.756931000
1 -4.972155000 -0.369419000 0.407091000
1 -6.303553000 -0.013835000 -2.329416000
1 -7.141057000 0.014180000 -0.771292000
1 -5.978907000 1.287151000 -1.168169000
1 1.691128000 -2.142132000 -0.301174000
1 1.932868000 1.196220000 1.296669000
1 -1.290178000 -0.016785000 0.812289000
1 1.929767000 -1.373001000 -2.643253000
1 3.161561000 -0.218700000 -2.146879000
1 3.344236000 -3.251481000 -1.858898000
1 4.219905000 -2.280737000 -3.037552000
1 5.662373000 -2.829163000 -1.073464000
1 5.395907000 -1.093702000 -1.195523000
1 3.856918000 -2.990616000 0.632492000
1 5.096979000 -1.854023000 1.152264000
1 2.839614000 -0.903583000 1.539832000
1 3.717332000 0.041865000 0.343604000
1 2.956183000 1.729173000 -0.936072000
1 1.504458000 2.631409000 -1.360079000
1 3.355515000 3.280044000 0.979671000
1 3.296324000 4.159103000 -0.545115000
1 1.945183000 5.317530000 1.211797000
1 0.927688000 4.768663000 -0.115759000
1 1.318234000 3.313682000 2.536047000
1 -0.121336000 4.210538000 2.066067000
1 -0.512788000 2.636308000 0.185869000
1 -0.438156000 1.781041000 1.722734000
1 -1.506149000 -1.770493000 -0.941730000
1 -0.384752000 -2.793687000 -0.041555000
1 -3.033667000 -1.726565000 1.023936000
1 -2.752129000 -3.382136000 0.490028000
1 -2.577474000 -3.203590000 2.973930000
1 -1.032112000 -3.663669000 2.268596000
1 -1.828273000 -0.839549000 3.106439000
1 -0.701604000 -1.885286000 3.966573000
1 0.802580000 -1.897269000 1.980359000
1 0.522585000 -0.248922000 2.542785000

3_TS_PBu3_1

6 0.088614000 -0.662152000 -1.598956000
6 1.283359000 -0.988887000 -1.558709000
6 2.549355000 -1.278005000 -0.984676000
8 2.866216000 -2.343431000 -0.478197000
8 3.396651000 -0.234498000 -1.085123000
6 4.671850000 -0.376222000 -0.444655000
6 4.561939000 -0.087390000 1.034968000
15 -0.745053000 -0.194834000 0.589663000
6 0.703204000 0.552246000 1.396903000
6 -2.191942000 0.617177000 1.366546000
6 -0.722699000 -1.857364000 1.333274000
6 0.803181000 2.067498000 1.246472000
6 0.805542000 2.545104000 -0.200067000
6 0.830587000 4.062109000 -0.292018000
6 -1.967798000 -2.696259000 1.050632000
6 -2.363606000 -2.742778000 -0.419552000
6 -3.531943000 -3.685487000 -0.660495000
6 -2.665713000 1.879914000 0.646002000
6 -3.077396000 1.632122000 -0.799417000

6	-3.488083000	2.920323000	-1.495353000
1	-0.803055000	-0.510137000	-2.174596000
1	5.055779000	-1.379028000	-0.627741000
1	5.316741000	0.345979000	-0.941322000
1	4.150991000	0.910865000	1.198306000
1	3.918131000	-0.819213000	1.523689000
1	5.548430000	-0.133311000	1.497494000
1	0.685862000	0.269766000	2.453880000
1	1.582541000	0.070696000	0.956350000
1	-2.999678000	-0.121981000	1.351457000
1	-1.960287000	0.819683000	2.417805000
1	0.165442000	-2.344741000	0.914566000
1	-0.560107000	-1.762654000	2.411355000
1	-0.022427000	2.543672000	1.786492000
1	1.720360000	2.411622000	1.735291000
1	1.664697000	2.108982000	-0.720334000
1	-0.082730000	2.162783000	-0.717734000
1	1.709853000	4.470489000	0.211694000
1	0.846371000	4.405920000	-1.326887000
1	-0.053524000	4.489198000	0.190213000
1	-1.790329000	-3.716211000	1.404172000
1	-2.811845000	-2.315919000	1.634674000
1	-2.627734000	-1.732283000	-0.758850000
1	-1.498471000	-3.046897000	-1.016940000
1	-3.273649000	-4.704049000	-0.362496000
1	-4.402698000	-3.380784000	-0.075098000
1	-3.825435000	-3.707752000	-1.710549000
1	-3.516912000	2.299038000	1.191051000
1	-1.885544000	2.647205000	0.663205000
1	-2.242289000	1.174149000	-1.341525000
1	-3.897203000	0.906422000	-0.822245000
1	-3.792337000	2.746067000	-2.527916000
1	-4.321518000	3.397498000	-0.974822000
1	-2.655278000	3.629180000	-1.506426000

3_TS_PBu3_2

6	0.088126000	-0.662758000	-1.598768000
6	1.282732000	-0.990018000	-1.558525000
6	2.548639000	-1.279496000	-0.984477000
8	2.865051000	-2.344847000	-0.477557000
8	3.396408000	-0.236426000	-1.085455000
6	4.671555000	-0.378387000	-0.444942000
6	4.561795000	-0.088689000	1.034523000
15	-0.745364000	-0.194710000	0.589739000
6	0.703427000	0.551076000	1.397208000
6	-2.191616000	0.618625000	1.366418000
6	-0.724629000	-1.857265000	1.333354000
6	0.804799000	2.066237000	1.246805000
6	0.807899000	2.543842000	-0.199731000
6	0.834373000	4.060823000	-0.291679000
6	-1.970477000	-2.694997000	1.050559000
6	-2.366142000	-2.741109000	-0.419675000
6	-3.535320000	-3.682728000	-0.660797000
6	-2.664132000	1.881775000	0.645775000
6	-3.075896000	1.634277000	-0.799671000
6	-3.485412000	2.922792000	-1.495715000
1	-0.803485000	-0.510487000	-2.174434000
1	5.055023000	-1.381469000	-0.627484000
1	5.316768000	0.343248000	-0.942014000
1	5.548287000	-0.134646000	1.497043000
1	4.151152000	0.909786000	1.197302000
1	3.917779000	-0.820033000	1.523685000
1	0.685632000	0.268605000	2.454178000
1	1.582409000	0.068722000	0.956826000
1	-3.000032000	-0.119787000	1.351254000
1	-1.959899000	0.820945000	2.417698000
1	0.163100000	-2.345464000	0.914731000
1	-0.562060000	-1.762715000	2.411453000
1	-0.020490000	2.543157000	1.786656000
1	1.722187000	2.409528000	1.735820000
1	1.666756000	2.106915000	-0.719814000
1	-0.080621000	2.162350000	-0.717582000
1	-0.049450000	4.488736000	0.190350000
1	1.713906000	4.468389000	0.212227000
1	0.850708000	4.404615000	-1.326546000
1	-1.794007000	-3.715120000	1.404105000
1	-2.814237000	-2.313870000	1.634502000
1	-2.629287000	-1.730356000	-0.758972000
1	-1.501211000	-3.046009000	-1.016960000
1	-4.405866000	-3.377240000	-0.075497000
1	-3.828702000	-3.704688000	-1.710887000
1	-3.278004000	-4.701538000	-0.362799000
1	-3.514993000	2.301723000	1.190717000
1	-1.883242000	2.648333000	0.663020000
1	-2.241127000	1.175556000	-1.341666000
1	-3.896324000	0.909281000	-0.822537000
1	-3.789697000	2.748739000	-2.528303000
1	-4.318505000	3.400693000	-0.975302000
1	-2.652012000	3.630948000	-1.506731000

3_TS_PBu3_3

6	-0.084268000	-0.368849000	-1.601804000
6	-1.289480000	-0.652825000	-1.555000000
6	-2.550184000	-0.921277000	-0.959249000
8	-2.890460000	-1.997556000	-0.491703000
8	-3.363159000	0.153040000	-0.990439000
6	-4.626918000	0.019894000	-0.324779000
6	-4.472976000	0.235103000	1.163506000
15	0.738433000	0.038691000	0.600253000
6	0.370881000	-1.552089000	1.409561000
6	2.262769000	0.625457000	1.430867000
6	-0.582367000	1.072115000	1.306585000
6	1.482157000	-2.592395000	1.272740000
6	1.786979000	-2.998724000	-0.166889000
6	0.658122000	-3.797971000	-0.802844000
6	-0.464749000	2.568295000	1.025606000
6	-0.328661000	2.913846000	-0.451087000
6	-0.324262000	4.416755000	-0.681043000
6	3.535494000	0.321138000	0.640529000
6	3.591387000	1.056749000	-0.692206000
6	4.878727000	0.771197000	-1.449603000
1	0.807278000	-0.229043000	-2.180016000
1	-5.261501000	0.782654000	-0.771489000
1	-5.044824000	-0.961738000	-0.544108000
1	-4.057868000	1.223576000	1.366874000
1	-5.445409000	0.163459000	1.651823000
1	-3.815166000	-0.520412000	1.594960000
1	0.178377000	-1.348947000	2.467978000
1	-0.568273000	-1.912426000	0.976276000
1	2.167707000	1.709486000	1.548505000
1	2.299705000	0.192702000	2.436210000
1	-1.515346000	0.686458000	0.880791000
1	-0.627778000	0.883843000	2.383800000
1	2.387831000	-2.204441000	1.748409000
1	1.197461000	-3.485194000	1.838001000
1	1.993139000	-2.101993000	-0.764911000
1	2.704494000	-3.592430000	-0.180939000
1	0.486310000	-4.722119000	-0.245453000
1	-0.278660000	-3.237506000	-0.813124000
1	0.891420000	-4.067140000	-1.834091000
1	-1.353718000	3.066035000	1.426188000
1	0.386935000	2.986313000	1.571652000
1	0.596175000	2.475041000	-0.845928000
1	-1.146920000	2.444318000	-1.006732000
1	0.499583000	4.889443000	-0.140938000
1	-0.217469000	4.664432000	-1.737703000
1	-1.253817000	4.865576000	-0.323547000
1	4.405919000	0.604661000	1.240032000
1	3.621983000	-0.755193000	0.458320000
1	2.729023000	0.767222000	-1.303965000
1	3.490496000	2.132023000	-0.510017000
1	5.749538000	1.073159000	-0.863392000
1	4.974796000	-0.296924000	-1.658117000
1	4.915122000	1.302361000	-2.401352000

7 References

(S1) (a) W. A. Henderson Jr. and C. A. Streuli, *J. Am. Chem. Soc.*, 1960, **82**, 5791–5794. (b) T. Allman and R. G. Goel, *Can. J. Chem.*, 1982, **60**, 716–722. (c) P. M. Zizelman, C. Amatore and J. K. Kochi, *J. Am. Chem. Soc.*, 1984, **106**, 3771–3784.

(S2) S. Tshepelevitsh, A. Kütt, M. Lõkov, I. Kaljurand, J. Saame, A. Heering, P. G. Plieger, R. Vianello and I. Leito, *Eur. J. Org. Chem.*, 2019, **2019**, 6735–6748.

(S3) (a) J. G. Atton and L. A. P. Kane-Maguire, *J. Chem. Soc., Dalton Trans.*, 1982, 1491–1498. (b) L. A. P. Kane-Maguire, E. D. Honig and D. A Sweigart, *Chem. Rev.*, 1984, **84**, 525–543.

(S4) G. R. Fulmer, A. J. M. Miller, N. H. Sherden, H. E. Gottlieb, A. Nudelman, B. M. Stoltz, J. E. Bercaw and K. I. Goldberg, *Organometallics*, 2010, **29**, 2176–2179.

(S5) R. W. Lang and H. J. Hansen, *Org. Synth.*, 1984, **6**, 202.

(S6) F. An, H. Jangra, Y. Wei, M. Shi, H. Zipse and A. R. Ofial, *Chem. Commun.*, 2022, **58**, 3358–3361.

(S7) P. Bhattacharya, J. A. Krause and H. Guan, *Organometallics*, 2014, **33**, 6113–6121.

(S8) A ^{31}P NMR chemical shift of +25.5 ppm for **P2** with a phenyltetrazole counteranion was reported in: D. Virieux, A. F. Guillouzic and H.-J. Cristau, *Tetrahedron*, 2006, **62**, 3710–3720.

(S9) J. R. Van Wazer, C. F. Callis, J. N. Shoolery and R. C. Jones, *J. Am. Chem. Soc.*, 1956, **78**, 5709–5962.

(S10) Ł. Kapuśniak, P. N. Plessow, D. Tryzbiński, K. Woźniak, P. Hofmann and P. I. Jolly, *Organometallics*, 2021, **40**, 693–701.

(S11) A. K. Sharma, H. Joshi, R. Bhaskar and A. K. Singh, *Dalton Trans.*, 2019, **48**, 10962–10970.

(S12) The ^{31}P NMR chemical shift of PPh_3 in this work is consistent with data reported in: T. Li, A. J. Lough and R. H. Morris, *Chem. Eur. J.*, 2007, **13**, 3796–3803.

(S13) E. Mercier, B. Fonovic, C. Henry, O. Kwon and T. Dudding, *Tetrahedron Lett.*, 2007, **48**, 3617–3620.

(S14) Y. Liang, S. Liu, Y. Xia, Y. Li and Z-X. Yu, *Chem. Eur. J.*, 2008, **14**, 4361–4373.

(S15) (*a*) A. D. Becke, *J. Chem. Phys.*, 1993, **98**, 5648–5656. (b) P. J. Stephens, F. J. Devlin, C. F. Chabalowski and M. J. Frisch, *J. Phys. Chem.*, 1994, **98**, 11623–11627.

(S16) (*a*) T. Clark, J. Chandrasekhar, G. W.Spitznagel and P. v. R. Schleyer, *J. Comput. Chem.*, 1983, **4**, 294–301. (*b*) M. J. Frisch, J. A. Pople and J. S. S. Binkley, *J. Chem. Phys*. 1984, **80**, 3265–3269. (*c*) R. Ditchfield, W. J. Hehre and J. A. Pople, *J. Chem. Phys.*, 1971, **54**, 724–728. (d) R. Krishnan, J. S. Binkley, R. Seeger and J. A. Pople, *J. Chem. Phys.*, 1980, **72**, 650-654.

(S17) F. Weigend and R. Ahlrichs, *Phys. Chem. Chem. Phys.*, 2005, **7**, 3297–3305.

(S18) (a) S. Grimme, J. Antony, S. Ehrlich and H. Krieg, *J. Chem. Phys.*, 2010, **132**, 154104. (b) S. Grimme, *J. Comput. Chem.*, 2006, **27**, 1787–1799.

(S19) H. S. Yu, X. He, S. L. Li and D. G. Truhlar, *Chem. Sci.,* 2019, **7**, 5032–5051.

(S20) J.-D. Chai and M. Head-Gordon, *Phys. Chem. Chem. Phys.*, 2008, **10**, 6615–6620.

(S21) S. Grimme, *J. Chem. Phys.*, 2006, **124**, 034108.

(S22) (*a*) V. Barone and M. Cossi, *J. Phys. Chem. A*, 1998, **102**, 1995–2001. (*b*) M. Cossi, N. Rega, G. Scalmani and V. Barone, *J. Comput. Chem*., 2003, **24**, 669–681.

(S23) A. V. Marenich, C. J. Cramer and D. G. Truhlar, *J. Phys. Chem. B.*, 2009, **113**, 6378–6396.

(S24) Gaussian 16, Revision B.01., M. J. Frisch, G. W. Trucks, H. B. Schlegel, G. E. Scuseria, M. A. Robb, J. R. Cheeseman, G. Scalmani, V. Barone, G. A. Petersson, H. Nakatsuji, X. Li, M. Caricato, A. V. Marenich, J. Bloino, B. G. Janesko, R. Gomperts, B. Mennucci, H. P. Hratchian, J. V. Ortiz, A. F. Izmaylov, J. L. Sonnenberg, D. Williams-Young, F. Ding, F. Lipparini, F. Egidi, J. Goings, B. Peng, A. Petrone, T. Henderson, D. Ranasinghe, V. G. Zakrzewski, J. Gao, N. Rega, G. Zheng, W. Liang, M. Hada, M. Ehara, K. Toyota, R. Fukuda, J. Hasegawa, M. Ishida, T. Nakajima, Y. Honda, O. Kitao, H. Nakai, T. Vreven, K. Throssell, J. A. Montgomery, Jr., J. E. Peralta, F. Ogliaro, M. J. Bearpark, J. J. Heyd, E. N. Brothers, K. N. Kudin, V. N. Staroverov, T. A. Keith, R. Kobayashi, J. Normand, K. Raghavachari, A. P. Rendell, J. C. Burant, S. S. Iyengar, J. Tomasi, M. Cossi, J. M. Millam, M. Klene, C. Adamo, R. Cammi, J. W. Ochterski, R. L. Martin, K. Morokuma, O. Farkas, J. B. Foresman, and D. J. Fox, Gaussian, Inc., Wallingford CT, 2016.

(S25) S. Grimme, *J. Chem. Theory Comput.,* 2019, **15**, 2847–2862.

(S26) C. Bannwarth, S. Ehlert and S. Grimme, *J. Chem. Theory Comput.,* 2019, **15**, 1652–1671.

(S27) S. Grimme, *Chem. Eur. J.,* 2012, **18**, 9955–9964.

(S28) P. Yi, J. Gomes, S. Mallikarjun Sharada, A. T. Bell and M. Head-Gordon, *Chem. Eur. J.,* 2012, **18**, 9955–9964.

(S29) G. Luchini, J. V. Alegre-Requena, I. Funes-Ardoiz and R. S. Paton*, F1000Research,* 2020, **9** (Chem Inf Sci), 291.

(S30) A. J. Foster and F. Weinhold, *J. Am. Chem. Soc.*, 1980, **102**, 7211–7218.

(S31) A. E. Reed, L. A. Curtiss and F. Weinhold, *Chem. Rev.*, 1988, **88**, 899–926.

(S32) C. Riplinger and F. Neese, *J. Chem. Phys.,* 2013*,* **138**, 034106.

(S33) C. Riplinger, B. Sandhoefer, A. Hansen and F. Neese, *J. Chem. Phys.,* 2013*,* **139**, 134101.

(S34) (a) F. Neese, *WIREs Comput. Mol. Sci.*, 2012, **2**, 73–78. (b) F. Neese, *WIREs Comput. Mol. Sci.*, 2018, **8**, e1327. (c) F. Neese, *WIREs Comput. Mol. Sci.*, 2022, **12**, e1606.

(S35) C. Y. Legault, *CYLview20*, Université de Sherbrooke, 2020 (http://www.cylview.org).

(S36) Values according to Active Thermochemical Tables (ATcT) Thermochemical Values ver.1.130: (a) Ruscic, R. E. Pinzon, M. L. Morton, G. von Laszewski, S. Bittner, S. G. Nijsure, K. A. Amin, M. Minkoff, and A. F. Wagner, *J. Phys. Chem. A*, 2004, **108**, 9979–9997 (b) B. Ruscic, R. E. Pinzon, G. von Laszewski, D. Kodeboyina, A. Burcat, D. Leahy, D. Montoya, and A. F. Wagner, *J. Phys. Conf. Ser.*, 2005, **16**, 561–570. (c) B. Ruscic and D. H. Bross, Argonne National Laboratory, Lemont, Illinois 2023; available at https://atct.anl.gov/.
